# From Isocyanides to Iminonitriles via Silver-mediated Sequential Insertion of C(sp^3^)–H Bond

**DOI:** 10.1016/j.isci.2019.10.057

**Published:** 2019-11-01

**Authors:** Huiwen Chi, Hao Li, Bingxin Liu, Rongxuan Ye, Haoyang Wang, Yin-Long Guo, Qitao Tan, Bin Xu

**Affiliations:** 1 Department of Chemistry, Innovative Drug Research Center, Shanghai University, Shanghai 200444, China; 2 State Key Laboratory of Organometallic Chemistry, Shanghai Institute of Organic Chemistry, Chinese Academy of Sciences, Shanghai 200032, China; 3 Qianweichang College, Shanghai University, Shanghai 200444, China

**Keywords:** Materials Chemistry, Optical Property, Organic Synthesis

## Abstract

Heterocycles are prevalent constituents of many marketing drugs and biologically active molecules to meet modern medical challenges. Isocyanide insertion into C(sp^3^)–H bonds is challenging especially for the construction of quaternary carbon centers. Herein, we describe an efficient strategy for the synthesis of α-iminonitrile substituted isochromans and tetrahydroisoquinolines (THIQs) with quaternary carbon centers through silver-triflate-mediated sequential isocyanide insertion of C(sp^3^)–H bonds, where isocyanide acts as the crucial “CN” and “imine” sources. The produced α-iminonitriles have extensive applications as valuable synthetic building blocks for pharmacologically interesting heterocycles. This protocol could be further applied for the synthesis of iminonitrile-decorated phenanthridines and azapyrene. Interestingly, a remarkable aggregation-induced emission (AIE) effect was first observed for an iminonitrile-decorated pyrene derivative, which may open a particular area for iminonitrile applications in materials science.

## Highlights

• Iminonitrile formation via sequential C(sp^3^)-H bond isocyanide insertion
• Construction of quaternary center
• Isocyanide as both "imine" and "CN" sources
• Valuable synthetic building blocks and novel AIEgen

## Graphical Abstract

**Figure:**
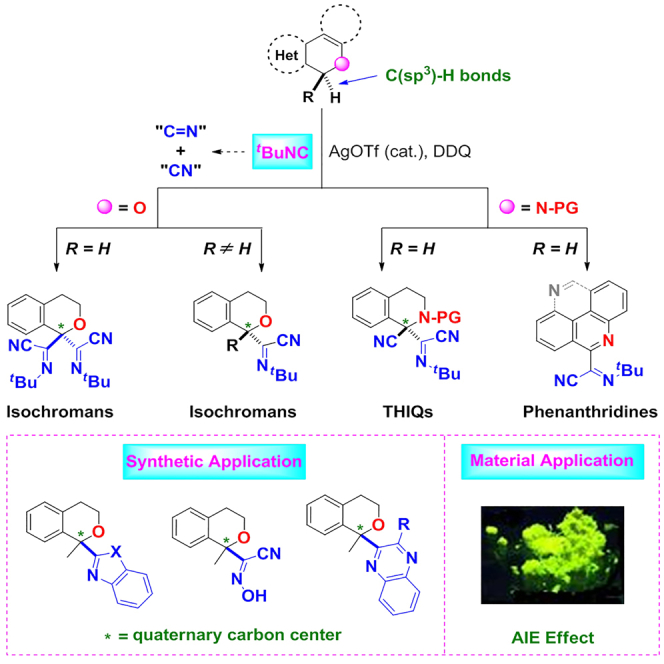

## Introduction

**Figure 1:**
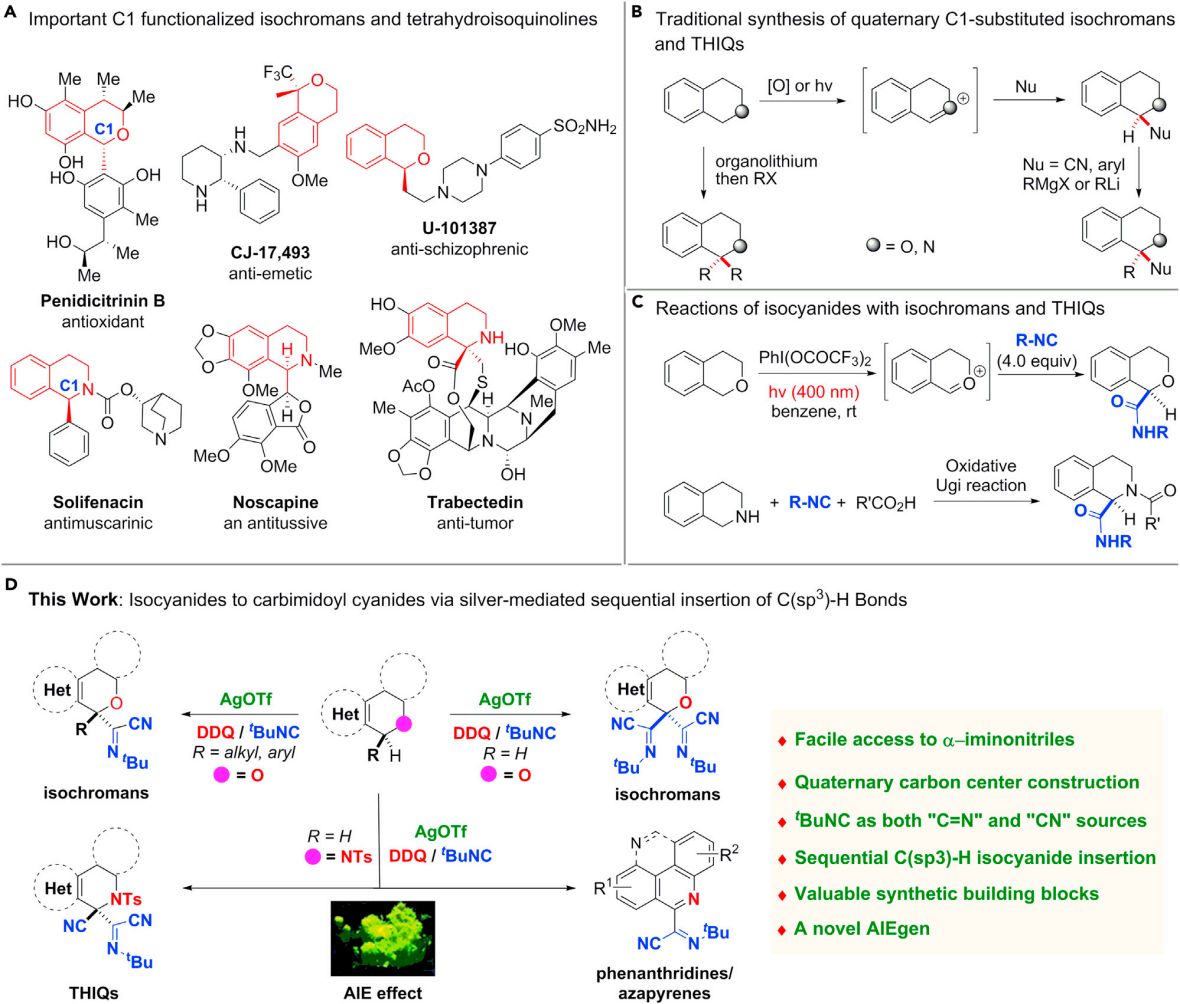
C1-Functionalization of Isochromans, THIQs, and Dihydrophenanthridines (A) Prevalence of C1 functionalized isochromans and THIQs motifs in marketing drugs and biologically active molecules. (B) Traditional methods for the construction of the quaternary C1 carbons are limited in scope and usually require multiple steps and active Grignard or organolithium reagents. (C) Reported reactions of isochromans and THIQs with isocyanides usually lead to C1 mono-functionalized amides. (D) Silver-mediated sequential isocyanide insertion of C(sp^3^)–H bond of isochromans, THIQs, and dihydrophenanthridines affords quaternary mono-/dual α-iminonitrile substituted products or phenanthridines, where the isocyanide acts as both “imine” and “CN” sources. The photograph was taken under ultraviolet (UV) lamp (365 nm) for an iminonitrile-decorated azapyrene with remarkable AIE effect.

Isochromans and tetrahydroisoquinolines (THIQs) are prevalent in many biologically active compounds including marketing drugs (Figure 1A) (Scott and Williams, 2002, Ennis et al., 1998). For example, penidicitrinin B is well known for its potent antioxidant activity (Clark et al., 2006, Lu et al., 2008). Solifenacin (VESIcare) is a muscarinic antagonist indicated for the treatment of overactive bladder with associated problems such as increased urination frequency and urge incontinence (Ohtake et al., 2004, Cardozo et al., 2004). In general, the functionalization of the C1 position of both scaffolds is important for their biologically activities. The site-selective C1 mono-functionalization of isochromans and THIQs has been extensively studied, which commonly involved the formation of oxonium/iminium ions or α-heteroatom carbon-centered radicals initiated by irradiation or treatment with an oxidant (Yoo et al., 2009, Zhou et al., 2017, Bartling et al., 2016, Lin et al., 2017, Muramatsu and Nakano, 2014, Muramatsu et al., 2013, Zhang et al., 2013, Meng et al., 2014). Although isochromans and THIQs with quaternary C1 carbons are of high potentials in drug discovery, represented by CJ-17493 (Shishido et al., 2008) and trabectedin (Germano et al., 2013, Demetri et al., 2009, Grosso et al., 2007), they still provide significant synthetic challenges to chemists. The C1 difunctionalization of isochromans and THIQs is limited in scope and commonly requires multiple steps using active Grignard or organolithium reagents (Figure 1B) (Guo et al., 2017, Li and Coldham, 2014).

Isocyanides have proven to be versatile C1 building blocks in organic synthesis and invoked ever-growing synthetic efforts, owing to their unique electronic configuration capable of reacting with electrophiles, nucleophiles, and radicals easily (Boyarskiy et al., 2015, Qiu et al., 2013, Song and Xu, 2017, Giustiniano et al., 2017). Although many challenges still remain due to the high energy barrier of activating the chemically inert C–H bonds regioselectively, the synergy from the combination of isocyanide insertion and C–H bond activation offers an efficient and powerful tool to establish complicated reactions and construct useful substances (Song and Xu, 2017). Numerous results have been reported on isocyanide insertions with C(sp^2^)–H or C(sp)–H bond. However, isocyanide insertion into C(sp^3^)–H bonds is challenging especially for the construction of quaternary carbon centers, since the pioneering intramolecular isocyanide insertion into benzylic C(sp^3^)–H bonds by Jones in the late 1980s (Jones and Kosar, 1986). Recently, a photolytic mono-amidation reaction of isochroman was achieved by Maruoka group through nucleophilic attack of excess amounts of isocyanide into the *in situ* generated oxocarbocation intermediate with phenyliodine bis(trifluoroacetate) (Figure 1C) (Sakamoto et al., 2015). In 2007, Zhu and co-workers reported an oxidative Ugi-type multicomponent reaction for the C1 monofunctionalization of THIQs (Figure 1C) (Ngouansavanh and Zhu, 2007). In these reports, no C1 disubstitution, leading to quaternary products could be observed from isochromans and THIQs.

α-Iminonitriles were generally prepared using highly toxic metal cyanides with multi-steps (Gualtierotti et al., 2012, You et al., 2014, Fontaine et al., 2008, Fontaine et al., 2009, Amos et al., 2003, De Corte et al., 1987, Surmont et al., 2009, Verhé et al., 1980, Maruoka et al., 1983), whereas improved synthetic method could be achieved by isocyanide insertion into C–O bond (Tobisu et al., 2007) or C–Halo bond (Chen et al., 2016). In view of the high bioactivities of isochromans and THIQs as well as our recent development of isocyanide chemistry (Huang et al., 2014, Fang et al., 2014, Hong et al., 2017), we herein report an unprecedented silver-mediated sequential isocyanide insertion of C(sp^3^)–H bonds to afford mono- or dual *α*-iminonitrile substituted isochromans and THIQs, as well as aromatized phenanthridines and azapyrene (Figure 1D). The significance of the given chemistry is as follows: (1) the formation of α-iminonitriles was first realized by the synergistically cascade isocyanide insertion via C–H bond activation, where the isocyanide was used as both the crucial “CN” and “imine” sources; (2) it is the first example to construct pharmacologically relevant α-iminonitrile substituted isochromans and THIQs with quaternary carbon centers through direct C(sp^3^)–H bond isocyanide insertion; (3) a remarkable aggregation-induced emission (AIE) effect was first observed for as-prepared α-iminonitrile substituted pyrene derivative, which may open a particular area for iminonitrile applications in materials science; (4) the α-iminonitrile substituted products are valuable synthetic building blocks for facile access of pharmacologically interesting heterocycles.

## Results and Discussion

### Reaction Optimization

We started our investigation by exploring the reaction of isochroman (**1a**) with *tert*-butyl isocyanide in chlorobenzene at 80°C in the presence of DDQ under a nitrogen atmosphere. To our surprise, a dual *α*-iminonitrile substituted isochroman **2a** was isolated in 47% yield, without observation of any direct cyanated products (Table 1, entry 1) (Xu et al., 2012, Hong et al., 2014, Peng et al., 2012). Various metal catalysts were next tested, including CuCl, FeCl_3_ and silver salts (entries 2–8), and the desired product **2a** was obtained in 61% yield when AgOTf was applied (entry 8). Screening of the other solvents indicated chlorobenzene to be the suitable choice (entries 8–15). An extensive screening of the amounts of AgOTf (entries 16 and 17), DDQ (entries 18 and 19) and *tert*-butyl isocyanide (entries 20–22), temperature (entries 23 and 24), and the atmosphere (entries 25 and 26) revealed that the use of 10 mol% of AgOTf and two equivalents of DDQ in chlorobenzene at 80°C under a nitrogen atmosphere provided the most suitable conditions.

**Table 1:** Optimization of Reaction Conditions^a^


Entry	Catalyst (mol%)	Isocyanide (equiv)	Solvent	Temp. (^o^C)	Yield (%)^b^
1	/	5.0	PhCl	80	47
2	CuCl (10)	5.0	PhCl	80	27
3	FeCl_3_ (10)	5.0	PhCl	80	36
4	Ag_2_CO_3_ (10)	5.0	PhCl	80	44
5	AgNO_3_ (10)	5.0	PhCl	80	38
6	AgTFA (10)	5.0	PhCl	80	39
7	AgOAc (10)	5.0	PhCl	80	42
8	AgOTf (10)	5.0	PhCl	80	61^c^
9	AgOTf (10)	5.0	DCE	80	35
10	AgOTf (10)	5.0	DMF	80	NP
11	AgOTf (10)	5.0	DMSO	80	NP
12	AgOTf (10)	5.0	CH_3_CN	80	NP
13	AgOTf (10)	5.0	dioxane	80	trace
14	AgOTf (10)	5.0	toluene	80	52
15	AgOTf (10)	5.0	CH_2_Cl_2_	20	22
16	AgOTf (5)	5.0	PhCl	80	51
17	AgOTf (20)	5.0	PhCl	80	50
18	AgOTf (10)	5.0	PhCl	80	54^d^
19	AgOTf (10)	5.0	PhCl	80	22^e^
20	AgOTf (10)	6.0	PhCl	80	56
21	AgOTf (10)	4.0	PhCl	80	54
22	AgOTf (10)	3.0	PhCl	80	36
23	AgOTf (10)	5.0	PhCl	100	44
24	AgOTf (10)	5.0	PhCl	60	39
25	AgOTf (10)	5.0	PhCl	80	52^f^
26	AgOTf (10)	5.0	PhCl	80	54^g^

a Reaction conditions: **1a** (0.3 mmol), catalyst (10 mol%), DDQ (2.0 equiv), solvent (3.0 mL), 3 h, under a nitrogen atmosphere. DDQ = 2,3-dichloro-5,6-dicyanobenzoquinone. NP = no product.

b Yields of isolated products are given.

c (*E*)-*N*-*tert*-butyl-1-cyanoisochroman-1-carbimidoyl cyanide (**2a′**) was also isolated in 17% yield.

d DDQ (3.0 equiv) was used.

e DDQ (1.0 equiv) was used.

f Under an oxygen atmosphere.

g Under an air atmosphere. H atoms of the X-ray structure were omitted for clarity.

### Substrate Scope of Isochromans

**Figure 2:**
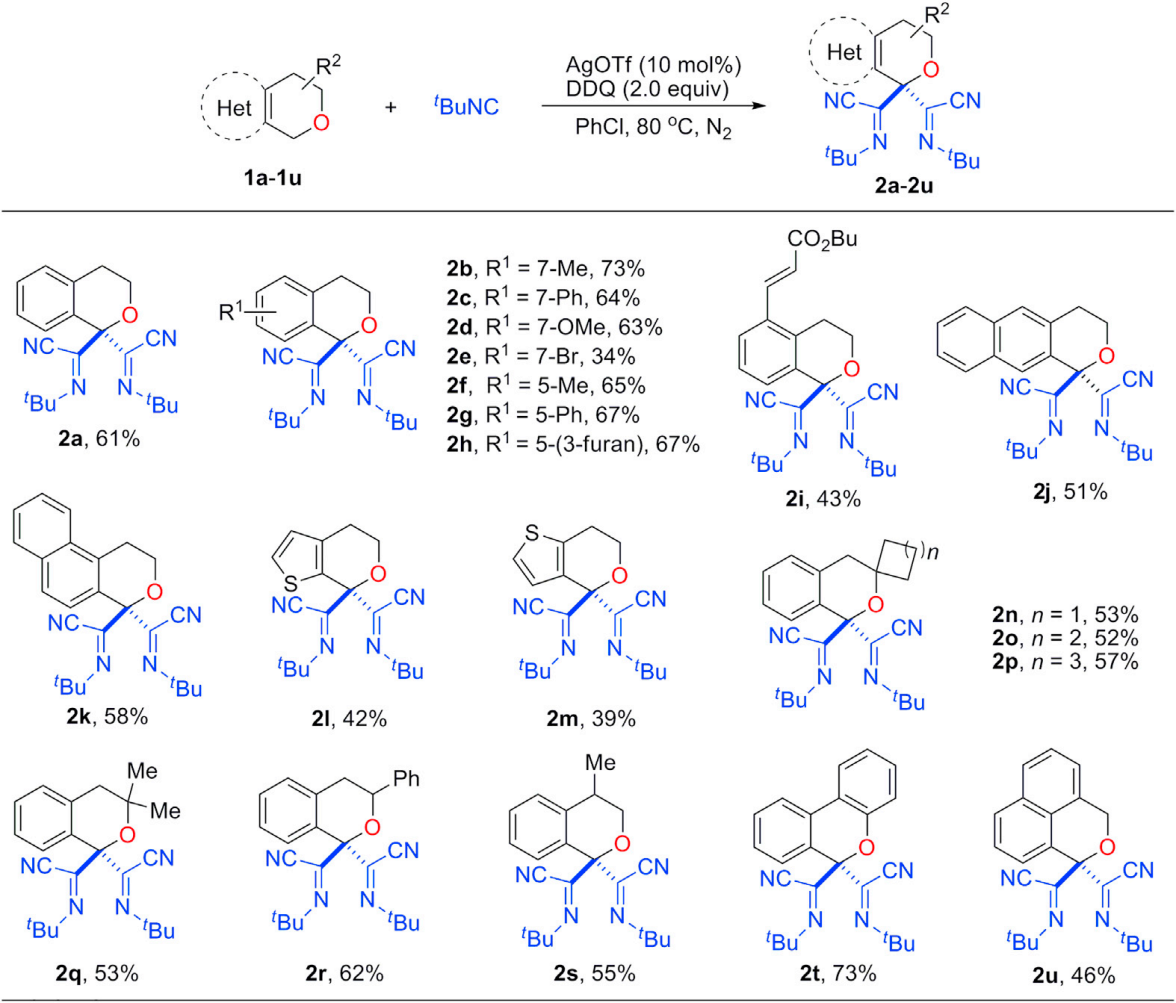
Substrate Scope of Isochroman Reaction Conditions: **1a**–**1u** (0.3 mmol), ^*t*^BuNC (5.0 equiv), AgOTf (10 mol%), DDQ (2.0 equiv), PhCl (3.0 mL), 3–6 h, under a nitrogen atmosphere, at 80°C. Yields of isolated products are given: 12 h for **2h**, **2i,** and **2u**; 10 h for **2l**; 7.5 h for **2m**.

With the optimized reaction conditions in hand, a variety of isochromans were examined as shown in Figure 2. Substrates bearing different functional groups on the aryl ring, regardless of their substitution patterns, were compatible with this reaction and provided the corresponding products in moderate to good yields (**2b**–**2i**). The reaction was not limited to simple isochromans, but naphthyl- or thienyl-fused substrates also gave the desired di-*α*-iminonitrile substituted products in moderate yields (**2j**–**2m**). Isochromans with 3- or 4-substituent could afford the spiro- (**2n**–**2p**); 3,3-dialkyl (**2q**); 3-aryl (**2r**); 4-alkyl (**2s**); and 3,4-fused (**2t**) products in moderate to good yields. Notably, when symmetrical 1*H*,3*H*-benzo[*de*]isochromene (**1u**) bearing two potential benzyl C(sp^3^)–H bond insertion positions was applied in this reaction, only one position was attacked and afforded the product **2u** predominately.

**Figure 3:**
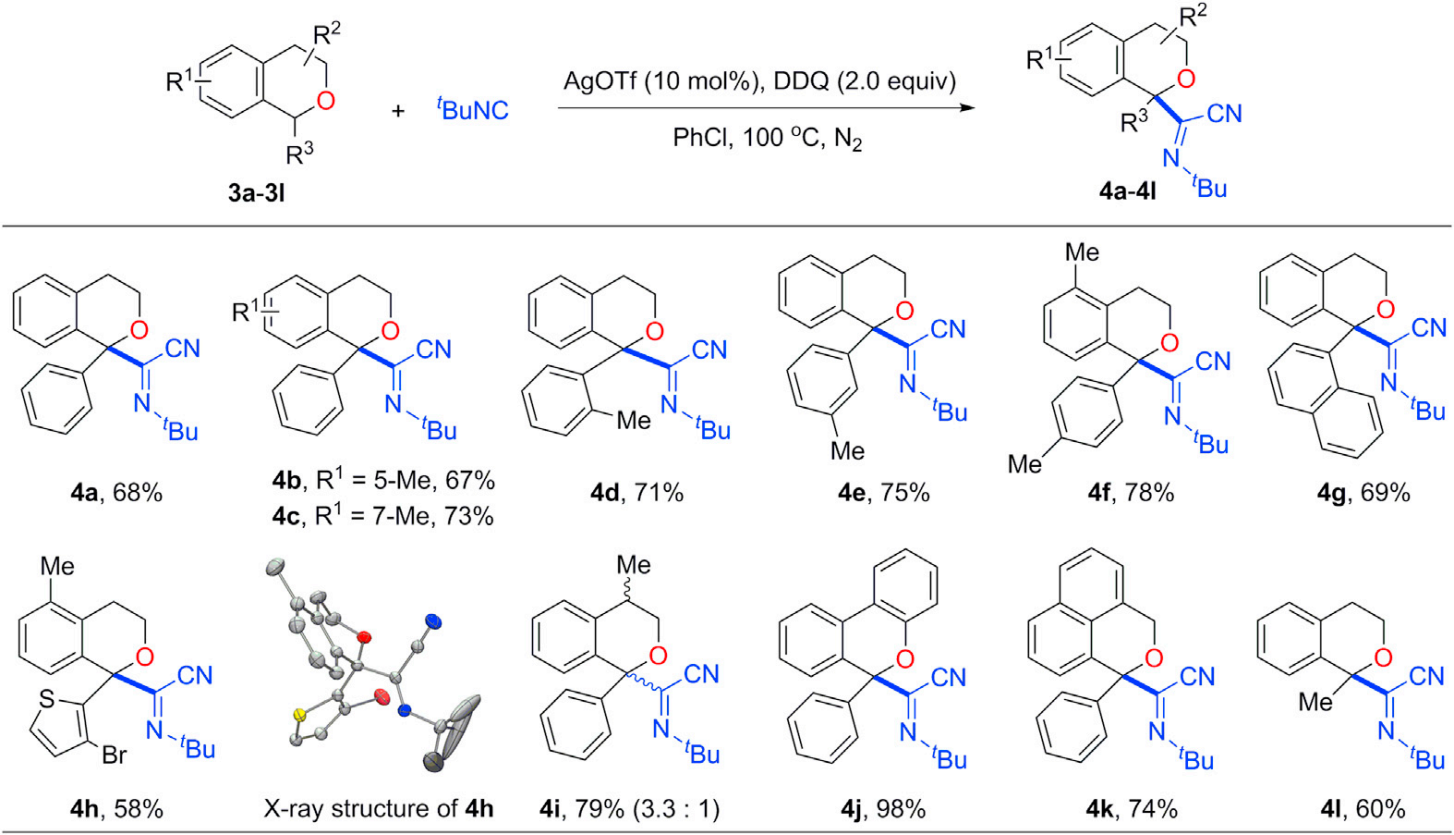
Substrate Scope of Isochroman Reaction conditions: **3a**–**3l** (0.3 mmol), ^*t*^BuNC (5.0 equiv), AgOTf (10 mol%), DDQ (2.0 equiv), PhCl (3.0 mL), 19–24 h, under a nitrogen atmosphere, at 100°C. Yields of isolated products are given. H atoms in the X-ray structure were omitted for clarity.

To further explore the scope and generality of this method, C1 mono-substituted isochromans were next explored for this insertion reaction with elevated temperature at 100°C. As illustrated in Figure 3, substrates with aryl groups, regardless of the substituent position on the aryl rings, provided the corresponding products in good yields (**4a**–**4f**). Similarly, 1-naphthyl or 1-thienyl isochromans afforded the desired products **4g** and **4h**, respectively. The identity of **4h** was determined by spectral analysis and further confirmed by X-ray crystallographic analysis. Moreover, 4-methyl-1-phenyl-isochroman (**3i**) could be employed in this transformation and afforded the product **4i** in 79% yield with a diastereomeric ratio of 3.3:1 as determined by proton NMR. Intriguingly, 6*H*-benzo[*c*]-chromene derivative **4j** could be isolated almost quantitatively, which may be attributed to the perfect stabilization of generated oxocarbenium ion (Meng et al., 2014, Jung and Floreancig, 2009) by the electron delocalization of conjugated system. Owing to the similar reason, isocyanide insertion will occur selectively on the more sterically hindered C1-position, instead of C3-position, to form isochroman **4k** in 74% yield. Furthermore, the less reactive 1-methyl-isochroman substrate also afforded the *α*-iminonitrile product **4l** in 60% yield at C1-position.

### Substrate Scope of THIQs

**Figure 4:**
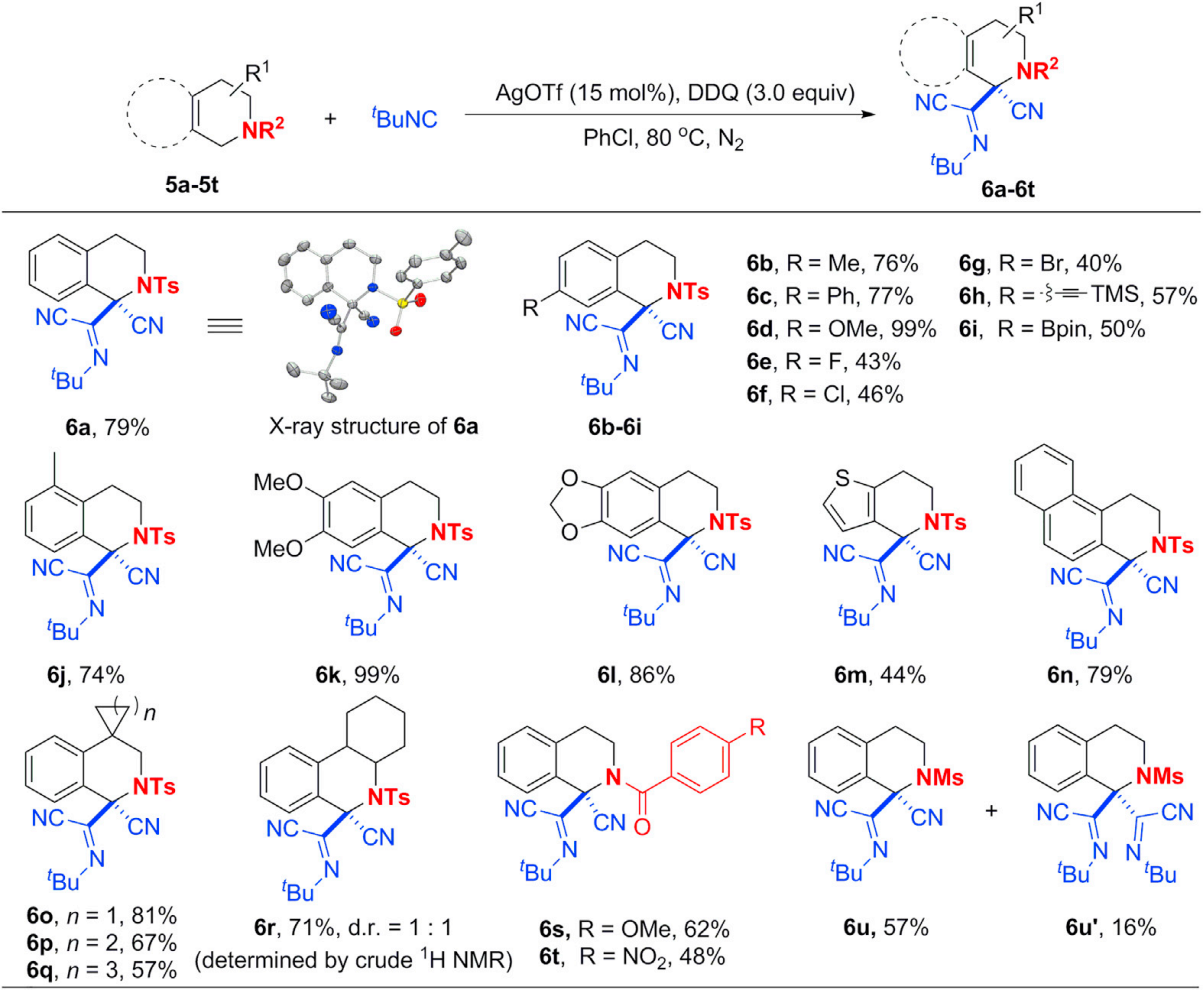
Substrate Scope of THIQs Reaction Conditions: **5a**–**5t** (0.3 mmol), ^*t*^BuNC (1.2 mmol), AgOTf (0.045 mmol), DDQ (0.9 mmol), PhCl (4.5 mL), 3–6 h, under nitrogen atmosphere, at 80°C. Yields of isolated products are given. H atoms in the X-ray structure were omitted for clarity.

The optimized conditions for isochromans could be further applicable to THIQs. Interestingly, in this case, only one α-iminonitrile group and a nitrile group were installed to the C1 position in comparison to the introduction of two α-iminonitriles for isochromans. As shown in Figure 4, THIQs bearing various substituents or functional groups on the aryl ring were smoothly converted into the corresponding products in moderate to excellent yields (**6a**–**6l**). Similarly, the expected products were obtained for THIQs analogues with fused heterocycle (**6m**) or extended π-systems (**6n**). THIQs with modified piperidine rings also afforded the desired spiro- or fused products (**6o**–**6r**). The replacement of the tosyl group by benzoyl groups gave similar results (**6s**–**6t**), whereas the use of acetyl group led to an unidentified mixture. However, when the tosyl group was replaced by methanesulfonyl group, a separable mixture of **6u** and **6u′** was obtained, which indicates that the existed more steric hindrance of tosyl group may prohibit the introduction of the second α-iminonitrile group. The different results of THIQs and isochromans may also attribute to the existence of the protecting group on THIQs, which sterically prohibits the introduction of the second α-iminonitrile group.

### Substrate Scope of Dihydrophenanthridines

**Figure 5:**
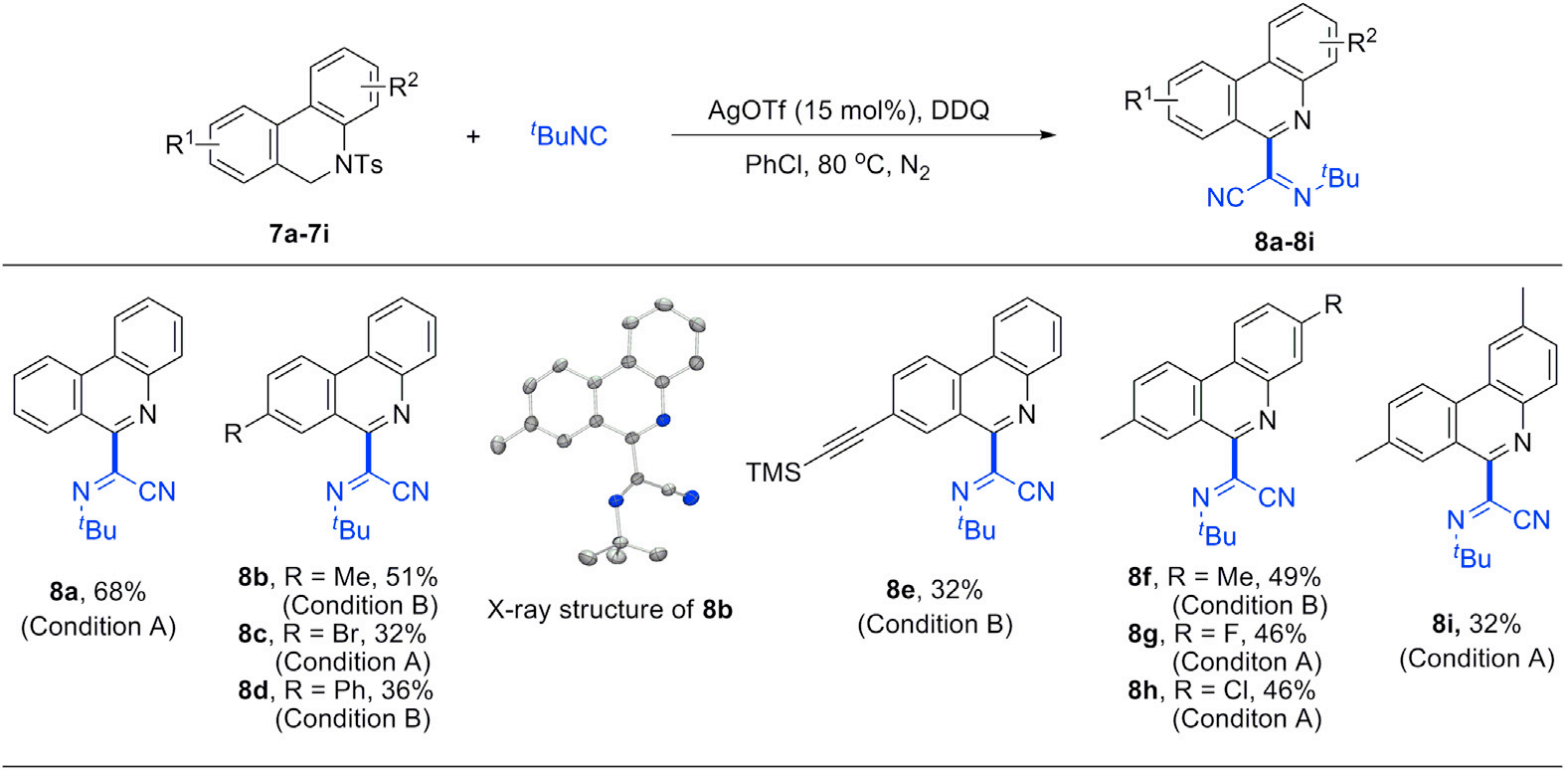
Substrate Scope of Dihydrophenanthridine Condition A: **7** (0.3 mmol), ^*t*^BuNC (1.2 mmol), AgOTf (0.045 mmol), DDQ (0.9 mmol), PhCl (4.5 mL), 3 h, under a nitrogen atmosphere, at 80°C. Condition B: **7** (0.3 mmol), ^*t*^BuNC (1.5 mmol), AgOTf (0.045 mmol), DDQ (1.2 mmol), PhCl (3.0 mL), 3 h, under a nitrogen atmosphere, at 80°C. Yields of isolated products are given. H atoms in the X-ray structure were omitted for clarity.

To our surprise, 5-tosyl-5,6-dihydro-phenanthridine (**7a**) under the same conditions gave aromatized phenanthridine **8a** with the elimination of the tosyl group. Functional groups such as methyl, halogen, phenyl, and alkynyl could be tolerated (**8b**–**8e**) (Figure 5). The structure of the product **8b** was confirmed by X-ray crystallographic analysis. Interestingly, the dihedral angle of the phenanthridine plane and the α-iminonitrile plane is 41°, which suggests an effective conjugation between the α-iminonitrile and the phenanthridine. Attributed to the strong tendency toward aromatization of dihydrophenathridine substrates, phenanthridines without substituents at the C6 position were observed in the reaction as a main byproduct, which lead to the formation of **8** in moderate yields. It should be noted that phenanthridines and their derivatives are of great interest in medicinal chemistry and materials science due to their potent biological activities and optoelectronic properties (Ishikawa, 2001, Dubost et al., 2012, Stevens et al., 2008).

### Synthetic Applications of the Products

**Figure 6:**
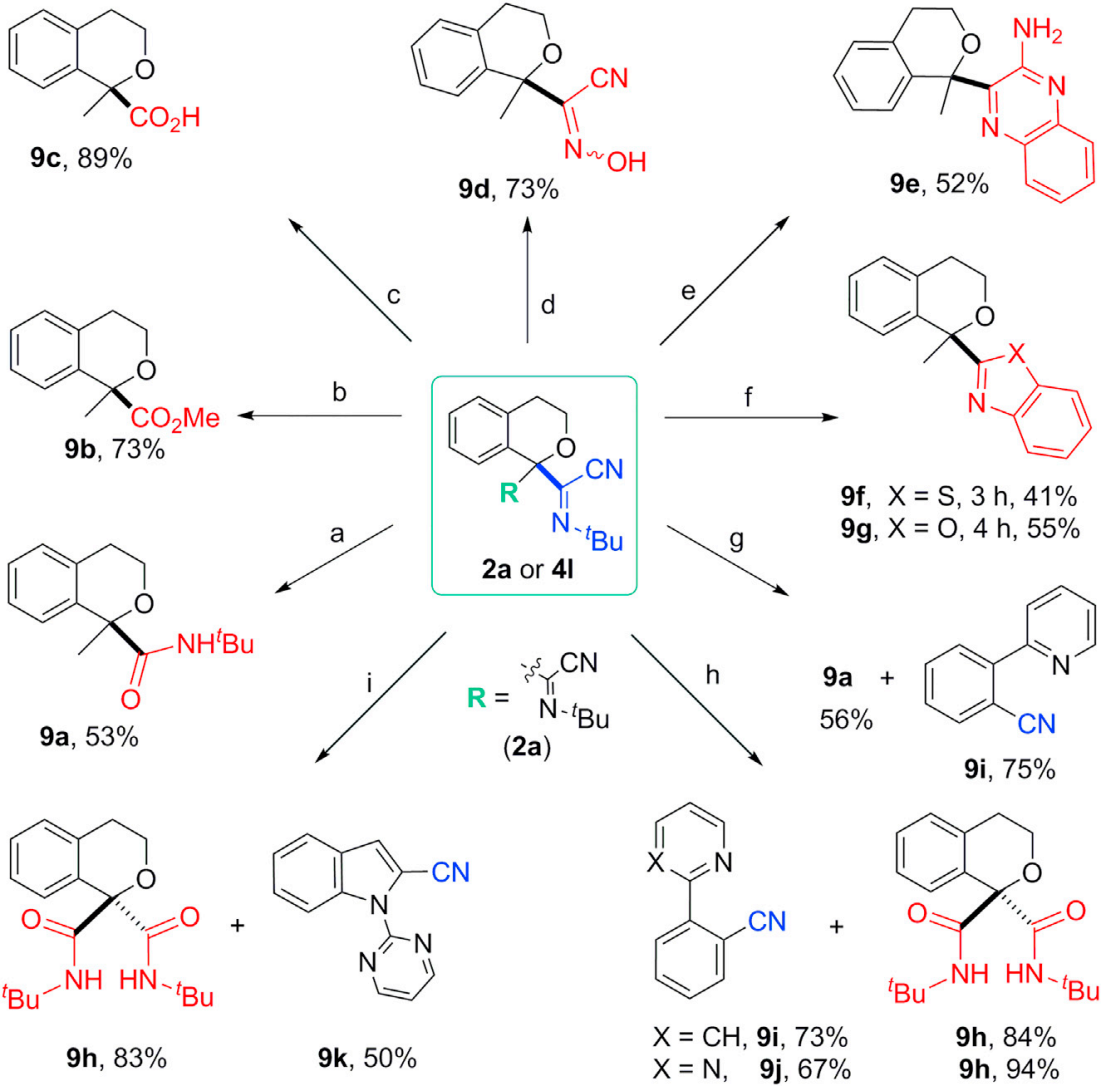
Synthetic applications Reaction conditions: (A) Al_2_O_3_, toluene, 150°C, 25 h; (B) HCl, MeOH, room temperature, 10 h; (C) HCl, CH_3_CN, room temperature, 2.5 h; (D) NH_2_OH· HCl, K_2_CO_3_, EtOH, reflux, 4 h; (E) *o*-Phenylenediamine, AcOH, 120°C, 7.5 h; (F) 2-Amino-benzenethiol or 2-aminophenol, AcOH, 120°C; (G) 2-Phenylpyridine, Pd(OAc)_2_, Cu(TFA)_2_, THF, 120°C, 23 h; (H) 2-Phenylpyridine or 2-phenylpyrimidine, Pd(OAc)_2_, Cu(TFA)_2_, THF, 120°C, 23 h; (I) 1-(Pyrimidin-2-yl)-1*H*-indole, Pd(OAc)_2_, Cu(TFA)_2_, THF, 120°C, 23 h

To demonstrate the synthetic utility of the given approach, we next turned our attention to the application of the current protocols, as depicted in Figure 6. Products (**2a** and **4l**) derived from isochromans were selected as examples. The corresponding isochroman carboxylate derivatives (**9a**–**9c**) could be easily obtained from *α*-iminonitrile **4l** in the presence of alumina or by treatment with hydrochloride solution, respectively. Exposure of **4l** to hydroxylamine in ethanol leads to the formation of *α*-cyanooxime **9d** in good yield. Notably, isochromans with aminoquinoxaline (**9e**), benzothiazole (**9f**), or benzoxazole (**9g**) substitutions at C1 position could be synthesized smoothly from *α*-iminonitrile **4l**, which provides a shortcut for pharmacologically interesting isochromanyl heterocycles. Iminonitrile substituted isochromans (**2a** and **4l**) are also proven to be excellent cyanating reagents, for example, direct C–H bond cyanation of 2-phenylpyridine or 2-phenylpyrimidine could be achieved to afford cyano products **9i** (Xu et al., 2012, Hong et al., 2014) or **9j** (Xu et al., 2012, Peng et al., 2012) efficiently, together with the formation of quaternary carbon centered amide (**9a**) or diamide (**9h**) in high yields, which is very difficult to obtain with general methods. Similarly, 1-(pyrimidin-2-yl)-1*H*-indole could be cyanated with **2a** to give the corresponding nitrile product **9k** in 50% yield (Xu et al., 2012).

### Application in Materials

**Figure 7:**
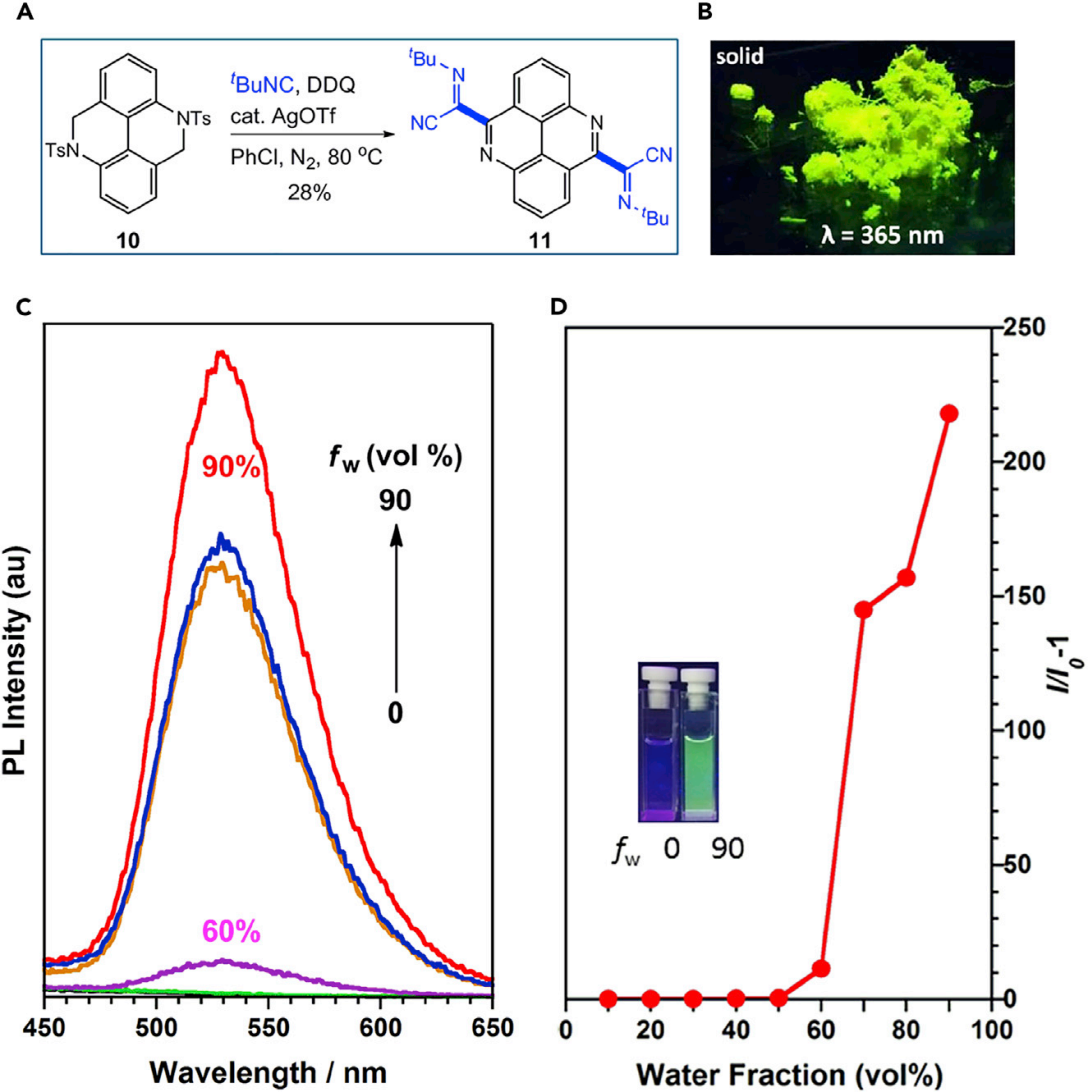
Aggregation-induced Emission (AIE) Behavior of Iminonitrile-decorated 4,9-diazapyrene (A) Synthesis through two-fold silver-mediated isocyanide insertion of C(sp^3^)–H Bond of **10**. (B) Photos of **11** in the solid state under UV lamp illumination. (C) PL spectra of **11** in THF/water mixtures with different fractions of water (*f*_w_). (D) Plot of *I*/*I*_0_ – 1 versus *f*_w_, where *I*_0_ is the PL intensity in pure THF solution ([**11**] = 20 μM). Inset: Photos of **11** in THF/water mixtures (*f*_w_ = 0, 90 vol%).

Luminescent materials are the basis of many high-tech innovations such as organic light-emitting diodes (OLEDs), biological probes, dyes, and chemical sensors. Pyrene, a flat aromatic molecule, exhibits excellent fluorescent properties and has found numerous applications in many fields (Duarte and Müllen, 2011). Therefore, we plan to prepare a α-iminonitrile-decorated pyrene derivative **11** by this newly developed method in order to investigate the effect of the introduced α-iminonitrile functional group on the optical properties. To our delight, compound **11** was successfully obtained through a two-fold isocyanide insertion to the C(sp^3^)–H bonds of **10** (Figure 7A). The optical properties of **11** were next investigated. It is well-known that most of pyrene derivatives are highly emissive in solution, whereas the emission is weak in the solid state due to the detrimental aggregation-caused quenching (ACQ). To our surprise, compound **11** was non-emissive when dissolved in organic solvents such as THF, but the solid showed bright green luminescence (λ_em_ = 528 nm, Figure 7B and Video S1). It underwent a further dramatic change from a non-emissive state in THF to highly emissive aggregated states in THF/water mixtures when the water content exceeded 60 vol% (Figures 7C, 7D, and S4); this phenomenon is a hallmark of the aggregation-induced emission (AIE) effect (Mei et al., 2015, Hong et al., 2011, Luo et al., 2001). In comparison, parent 4,9-diazapyrnene (Mosby, 1957), without α-iminonitrile substituent, is emissive in pure organic solvent (Figure S2), and no apparent AIE effect was observed. These results indicate that α-iminonitrile substituent might be an interesting AIEgen when appended to π-extended aromatic compounds. Furthermore, compound **11** showed a considerable bathochromic shift (63 nm) vs. parent 4,9-diazapyrnene both in the solid state (Figure S3), which disclosed that iminonitrile substituted isochromans would be an excellent chromophore for tuning the color of emissive materials.

Video S1. Aggregation-Induced Emission of Compound 11, Related to Figure 7

### Mechanistic Studies

**Figure 8:**
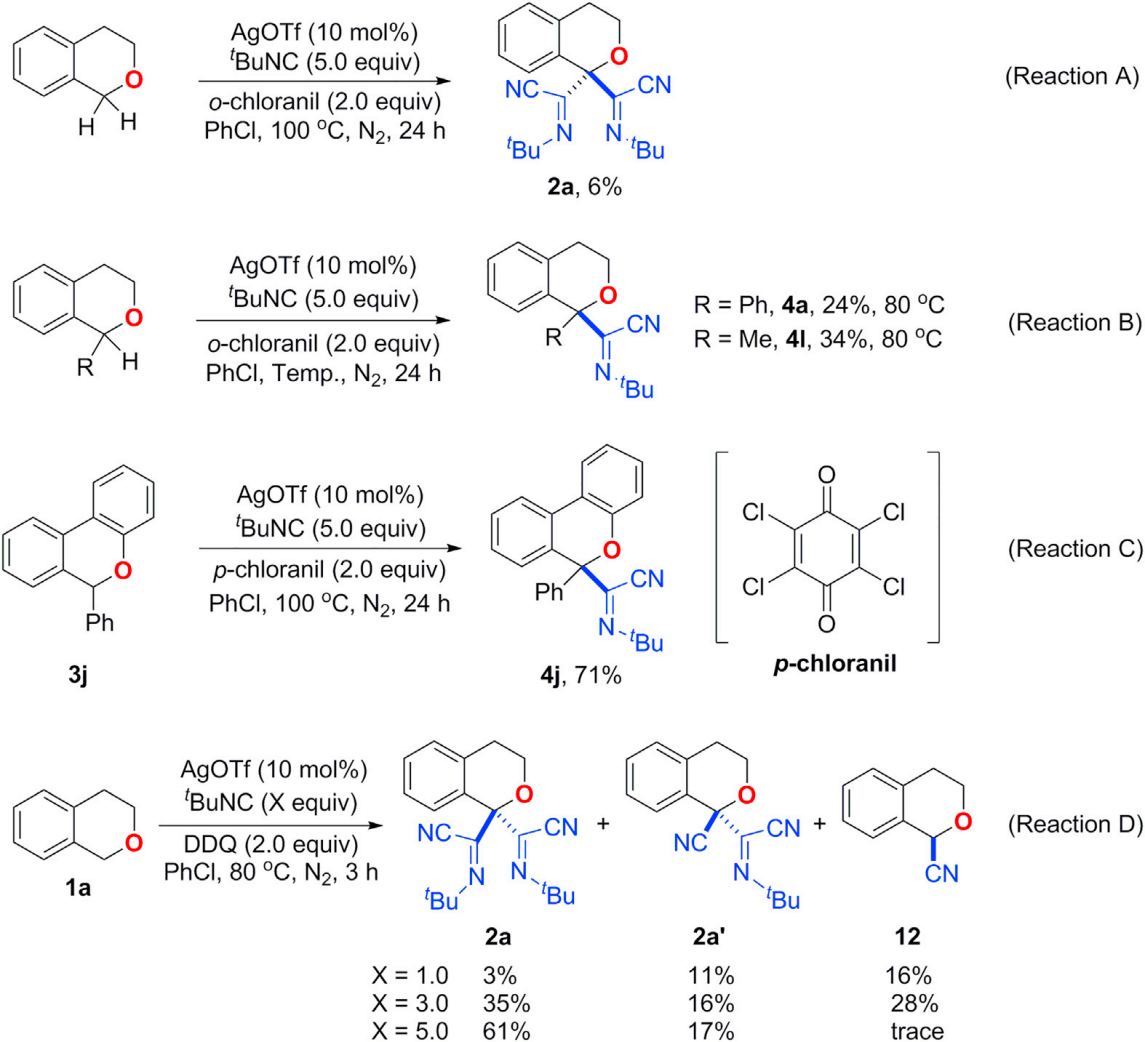
Preliminary Mechanistic Studies

To gain insight into the mechanism of this transformation, several control experiments were carried out as shown in Figure 8. Both isocyanide (Xu et al., 2012, Hong et al., 2014, Peng et al., 2012) and DDQ (Zhang et al., 2012) have been reported as effective cyanide sources in the literatures. To address the possible “CN” source in the reaction, the *o*- or *p*-chloranil, which has the similar character to DDQ except for the absence of cyanide groups, was used to replace DDQ under the optimized conditions. In the presence of *o*-chloranil, the desired products (**2a**, **4a** and **4l**) could also be afforded (Figure 8, Reactions A and B), albeit in relatively lower yields, which may be due to the different oxidative capacity between *o*-chloranil and DDQ. It was reported that DDQ has a higher reduction potential (0.6 V vs SCE) than *o*- and *p*-chloranil (0.14 and 0.02 V vs SCE, respectively) (Rathore and Kochi, 1998, Fukuzumi et al., 1993), which indicates that DDQ is a more powerful oxidant. When *p*-chloranil was used for the reaction of **3j**, iminonitrile **4j** could be afforded in 71% yield (Figure 8, Reaction C). When cyclohexyl- or 2,6-dimethylphenyl isocyanide was used instead, which are rarely used as “CN” source, no iminonitrile substituted isochromans could be isolated in the presence of DDQ. These results may rule out the possibility of DDQ as the main source of “CN.” Furthermore, the distribution of the cyanated products (**2a**, **2a′** and **12**) was sensitive to the amount of the isocyanide with the same amount of DDQ as an oxidant (Figure 8, Reaction D), which suggested the isocyanide as the “CN” source rather than DDQ. Interestingly, mono α-iminonitrile substituted isochroman was not obtained under these conditions.

**Figure 9:**
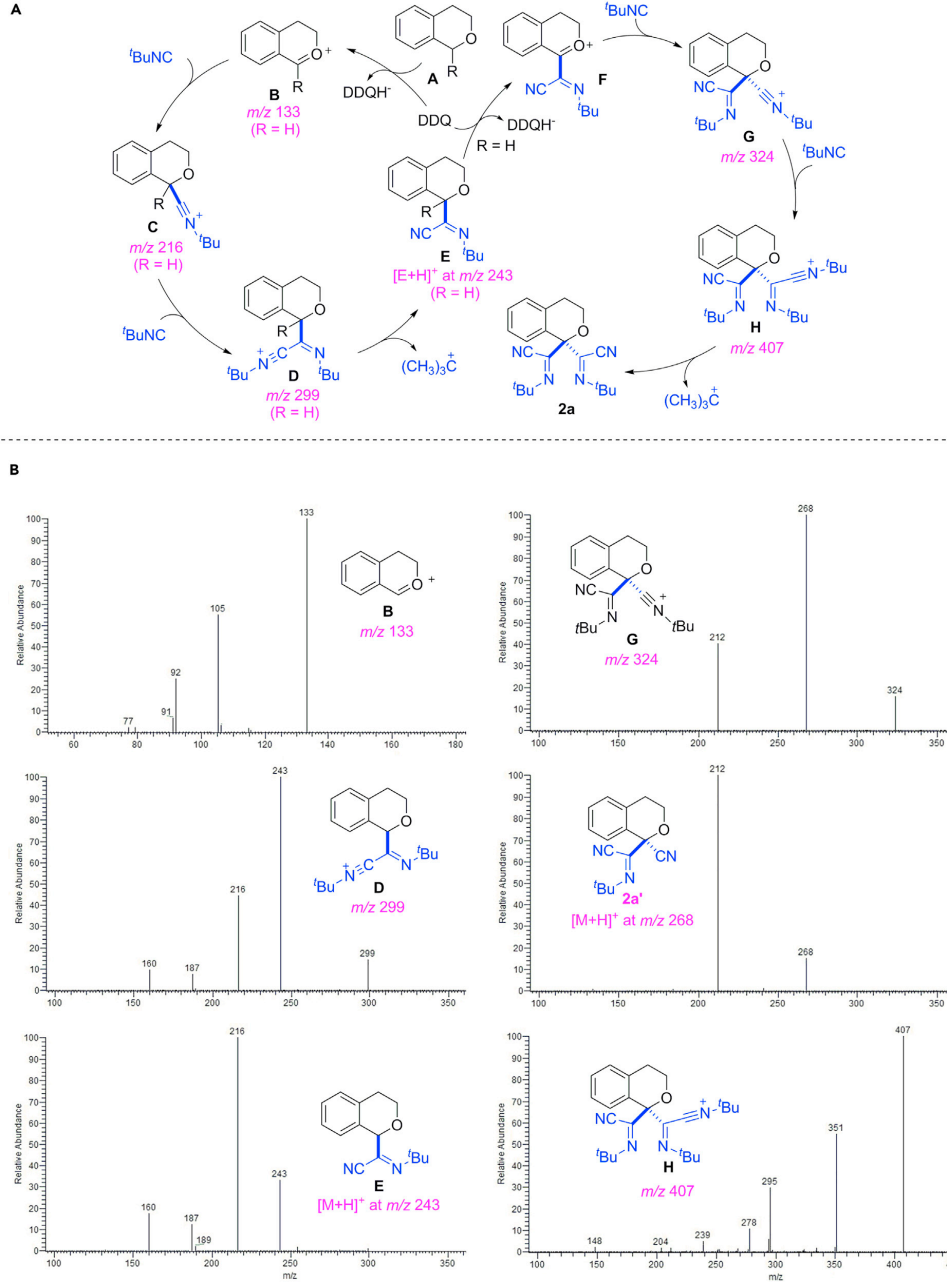
Plausible Mechanism and the Detection of the Key Intermediates by ESI-MS (A) Proposed mechanism for iminonitrile substituted isochromans. (B) The ESI-MS spectra of the intermediates in the reaction at the early stage of the reaction. Most of the proposed intermediates were detected.

The electrospray ionization mass spectroscopy (ESI-MS) has been used as an effective method for the characterization of reaction intermediates, which provides direct evidence for the reaction mechanism (Iacobucci et al., 2016, Guo et al., 2005, Hinderling et al., 1998). To further probe the progress of this cascade transformation, we monitored the reaction mixture of isochroman **1a**, ^*t*^BuNC, DDQ, and AgOTf in dichloromethane at room temperature by ESI-MS and electrospray ionization tandem mass spectrometry (ESI-MS/MS) techniques (for details, see Transparent Methods and Figures S9−S12). At the early stage of the reaction (30 min), the corresponding signal of some important ionic reactive species, such as intermediate **B** at *m/z* 133, **D** at *m/z* 299, [**E** + H]^+^ at *m/z* 243, **G** at *m/z* 324, and **H** at *m/z* 407, were observed in the positive ion ESI-MS spectrum of the reaction mixture (Figure 9B and S9–S12 and Schemes S1–S4). These results and the corresponding proposed dissociation pathways provide strong evidence for the reaction key intermediates.

Although a detailed reaction pathway remains to be clarified, a plausible mechanism for this reaction was proposed on the basis of above preliminary results (Figure 9A). A radical pathway might be ruled out as the reaction could not be inhibited by a typical radical scavenger 2,2,6,6-tetramethylpiperidine-1-oxyl (TEMPO). Initially, isochroman **A** was oxidized by DDQ in a reversible process to form the highly reactive benzoxy cation intermediate **B** (Jung and Floreancig, 2009), followed by the isocyanide addition to give the nitrilium ion intermediate **C**. The role of silver triflate may be accounted for the formation of coordinated silver-isocyanide complex to improve the nucleophilic reactivity of isocyanide (Gao et al., 2013, Liu et al., 2015, Álvarez-Corral et al., 2008). The attack by a second molecule of isocyanide on cation **C** afforded intermediate **D** (Tobisu et al., 2007, Saegusa et al., 1969), which would furnish the double isocyanide insertion product **E** via the leaving of *tert*-butyl cation by means of *β*-scission of the imidoyl cation (Saegusa et al., 1969, Xia and Ganem, 2002). The compound **E** (R = H) may generate the cation **F** rapidly as it has never been isolated during the reaction. Following the above procedure again, finally, the bis-iminonitrile product **2a** could be obtained smoothly from intermediate **H**.

### Conclusion

We have developed a direct synthesis of iminonitrile substituted isochromans and THIQs with quaternary carbon centers through silver-mediated sequential isocyanide insertion of C(sp^3^)–H bonds. The isocyanide is the typical precursor of *α*-iminonitrile and is conceived to play a two-fold role as both the crucial “CN” and “imine” sources. Mechanistic studies by ESI-MS and ESI-MS/MS techniques revealed that the reaction probably proceeded through nitrilium ion as the key intermediate. The given approach provided a convenient and practical method for the construction of synthetic meaningful *α*-iminonitrile skeleton in moderate to good yields with preferred substrate adaptability. The *α*-iminonitriles are not only valuable building blocks for the synthesis of pharmacologically interesting heterocycles but also potential chromophores for tuning the optical behavior of emissive materials, leading to an interesting AIEgen when appended to π-extended aromatics.

### Limitations of the Study

The substrates with strong electron-withdrawing groups such as CF_3_ and CN on the aryl rings are not suitable under standard conditions. Substrates with moderate electron-withdrawing halogens gave relatively lower yields. THIQs with free N–H bond or other protecting groups such as Boc and Ac gave trace amount of the desired products or complex mixtures. 1,3-Dihydroisobenzofuran and isoindoline also gave complicated mixture.

## Methods

All methods can be found in the accompanying Transparent Methods supplemental file.

## Data and Code Availability

The structures of **2a**, **4h**, **6a,** and **8b** reported in this article have been deposited in the Cambridge Crystallographic Data Center under accession numbers CCDC: 1533930, 1534967, 1829908, and 1829633.

## Supplemental Information

Document S1. Transparent Methods, Figures S1–S134, and Schemes S1–S4
