# Supplementary material for: From Isocyanides to Iminonitriles via Silver-mediated Sequential Insertion of C(sp3)–H Bond
Source: iScience. 2019 Nov 1;21:650–63. doi: 10.1016/j.isci.2019.10.057 (PMC6859232; doi:10.1016/j.isci.2019.10.057)
Supplement: Document S1. Transparent Methods, Figures S1–S134, and Schemes S1–S4 [file mmc1.pdf]

ISCI, Volume *21*

Supplemental Information

From Isocyanides to Iminonitriles via Silver-mediated Sequential Insertion of C(sp^3)–H Bond

Huiwen Chi, Hao Li, Bingxin Liu, Rongxuan Ye, Haoyang Wang, Yin-Long Guo, Qitao Tan, and Bin Xu

Transparent Methods

General Information

All reagents and metal catalysts were obtained from commercial sources without further purification, and commercially available solvents were purified before use. All new compounds were fully characterized. All melting points were taken on a WRS-1A or a WRS-1B Digital Melting Point Apparatus without correction. Infrared spectra were obtained using an AVATAR 370 FT-IR spectrometer. ^{1}H, ^{13}C, and ^{19}F NMR spectra were recorded with a Bruker AV-500 spectrometer operating at 500 MHz, 125 MHz and 470 MHz, respectively, with chemical shift values being reported in ppm relative to chloroform (δ = 7.26 ppm), dimethyl sulfoxide (δ = 2.50 ppm), acetone (δ = 2.09 ppm) or TMS (δ = 0.00 ppm) for ^{1}H NMR, with chloroform (δ = 77.16 ppm), dimethyl sulfoxide (δ = 39.52 ppm) or acetone (δ = 29.84 ppm) for ^{13}C NMR; and C_6F_6 (*δ* = -164.9 ppm) for ^{19}F NMR. Mass spectra and high resolution mass spectra (HRMS) were recorded with an Agilent 5975N using an Electron impact (EI) or Electrospray ionization (ESI) techniques. For mechanistic study, the electrospray ionization mass spectrometry (ESI-MS) and the subsequent tandem mass spectrometry (ESI-MS/MS) experiments were performed in Thermo TSQ Quantum Access^{TM} triple-quadrupole mass spectrometer. Ultraviolet spectra were measured on a PEGeneral spectrometer. Fluorescence spectra were recorded on a LS-55 spectrometer. Silica gel plate GF254 were used for thin layer chromatography (TLC) and silica gel H or 300–400 mesh were used for flash column chromatography. Yields refer to chromatographically and spectroscopically pure compounds, unless otherwise indicated.

Experimental Procedures

Synthesis and Characterization of Isochroman Substrates, Related to Figure 2 and Figure 3.

Isochroman **1a** is commercial available, and **1b**, **1d-1f**, **1j** and **1s-1u** were prepared according to the known methods (Zhou et al., 2013). Substrate **3a-3b**, **3d-3e**, **3i** and **3k-3l** were prepared according to the reported procedures (Muramatsu and Nakano, 2014). The spectra of the prepared substrates are consistent with the reported data. Other isochroman substrates are prepared as shown below.

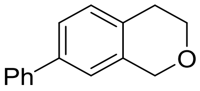

7-Phenylisochroman (1c): To a flask containing K_2CO_3 (138.2 mg, 1.0 mmol), $Pd(PPh_3)_4$ (14.4 mg, 1.25 mol%) and phenylboronic acid (67.1 mg, 0.55 mmol) in the aqueous solution of dioxane (5.0 mL) was added 7-bromoisochroman (**1e**) (106.0 mg, 0.5 mmol). The mixture was heated to 90 ^{o}C under N_2 for 8.5 h. Upon completion, the reaction mixture was cooled down to room temperature, diluted with ethyl acetate (10 mL) and washed with brine (2 × 30 mL). The combined organic phase was dried over Na_2SO_4 and purified by column chromatography on silica gel to give product **1c** as white solid (101.3 mg, 96%). M.p. 60-62 ^{o}C; IR (KBr, cm^{-1}):

3042, 2923, 2842, 1896, 1767, 1563, 1471, 1450, 1411, 1334, 1094, 988, 885, 816, 761, 699, 647; ^{1}H NMR ($CDCl_3$, 500 MHz): *δ* 7.59-7.57 (m, 2H), 7.46-7.41 (m, 3H), 7.37-7.33 (m, 1H), 7.23-7.20 (m, 2H), 4.86 (s, 2H), 4.03 (t, *J* = 5.7 Hz, 2H), 2.92 (t, *J* = 5.7 Hz, 2H); ^{13}C NMR ($CDCl_3$, 125 MHz): 140.9, 139.1, 135.3, 132.4, 129.3, 128.7, 127.2, 127.0, 125.2, 123.0, 68.1, 65.4, 28.1; EI-MS m/z (%): 210 (70) $[M]^+$, 180 (100), 181 (25), 165 (26); HRMS (EI) m/z calcd for $C_{15}H_{14}O$ $[M]^+$ 210.1045, found 210.1042.

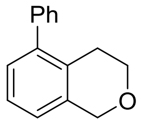

5-Phenylisochroman (1g): Following the general procedure as for **1c**, the reaction mixture of 5-bromoisochroman (254.4 mg, 1.2 mmol), phenylboronic acid (161.0 mg, 1.3 mmol), K_2CO_3 (331.7 mg, 2.4 mmol) and $Pd(PPh_3)_4$ (34.7 mg, 3.0 mol%) in the aqueous solution of dioxane (8.0 mL) was stirred at 90 ^{o}C for 14 h to afford product **1g** (204.0 mg, 81%) as white solid. M.p. 46-48 ^{o}C; IR (KBr, cm^{-1}): 2934, 2854, 1955, 1569, 1432, 1237, 1108, 1060, 999, 799, 757, 699; ^{1}H NMR ($CDCl_3$, 500 MHz): *δ* 7.41 (t, *J* = 7.5 Hz, 2H), 7.36-7.31 (m, 3H), 7.24 (t, *J* = 8.0 Hz, 1H), 7.13 (d, *J* = 7.5 Hz, 1H), 7.00 (d, *J* = 7.5 Hz, 1H), 4.86 (s, 2H), 3.89 (t, *J* = 5.7 Hz, 2H), 2.72 (t, *J* = 5.7 Hz, 2H); ^{13}C NMR ($CDCl_3$, 125 MHz): 142.0, 140.8, 135.1, 131.0, 129.1, 128.1, 127.7, 127.0, 125.8, 123.5, 68.1, 65.5, 27.5; EI-MS m/z (%): 210 (100) $[M]^+$, 181 (48), 180 (55), 166 (27), 165 (88); HRMS (EI) m/z calcd for $C_{15}H_{14}O$ $[M]^+$ 210.1045, found 210.1039.

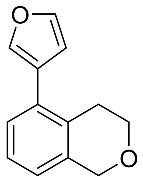

5-(Furan-3-yl)isochroman (1h): Following the general procedure as for **1c**, the reaction mixture of 5-bromoisochroman (196.1 mg, 0.9 mmol), furan-3-ylboronic acid (110.9 mg, 1.0 mmol), K_2CO_3 (229.4 mg, 1.8 mmol) and $Pd(PPh_3)_4$ (24.0 mg, 2.6 mol%) in the aqueous solution of dioxane was stirred at 90 ^{o}C for 8.5 h to afford product **1h** (86.5 mg, 54%) as white solid. M.p. 37-39 ^{o}C; IR (KBr, cm^{-1}): 2933, 2850, 1602, 1255, 1237, 1157, 1105, 1061, 1019, 873, 753, 725; ^{1}H NMR ($CDCl_3$, 500 MHz): *δ* 7.50 (d, *J* = 13.0 Hz, 2H), 7.23-7.19 (m, 2H), 6.96 (d, *J* = 6.5 Hz, 1H), 6.57 (s, 1H), 4.83 (s, 2H), 3.95 (t, *J* = 5.7 Hz, 2H), 2.85 (t, *J* = 5.5 Hz, 2H); ^{13}C NMR ($CDCl_3$, 125 MHz): 142.6, 140.0, 135.3, 132.3, 131.3, 127.3, 126.0, 124.5, 123.5, 111.4, 68.1, 65.4, 27.8; LC-MS (ESI) m/z 217 $[M+NH_4]^+$; HRMS (ESI) m/z calcd for $C_{13}H_{13}O_2$ $[M]^+$ 201.0910, found 201.0909.

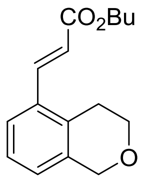

Butyl-3-(isochroman-5-yl)acrylate (1i): To a test tube containing 5-bromoisochroman (63.6 mg, 0.3 mmol), $Pd(OAc)_2$ (6.8 mg, 0.03 mmol) and Ph_3P (15.8 mg, 0.06 mmol) in DMF (3.0 mL), butyl acrylate (46.2 mg, 0.36 mmol) and TMEDA (69.7 mg, 0.6 mmol) were added. The mixture was heated to 125 ^{o}C under N_2 and stirred for 19 h. Upon completion, the reaction

mixture was cooled down to room temperature, diluted with ethyl acetate (10 mL) and washed with water (3 × 15 mL). The combined organic phase was dried over Na_2SO_4 and purified by column chromatography on silica gel to give product **1i** (86.8 mg, 100%) as colorless liquid. IR (KBr, cm^{-1}): 2959, 2864, 1712, 1634, 1459, 1308, 1263, 1222, 1172, 1114, 984, 786; ^{1}H NMR ($CDCl_3$, 500 MHz): δ 7.89 (d, J = 16.0 Hz, 1H), 7.44 (d, J = 7.5 Hz, 1H), 7.17 (t, J = 7.7 Hz, 1H), 6.99 (d, J = 7.5 Hz, 1H), 6.36 (d, J = 16.0 Hz, 1H), 4.76 (s, 2H), 4.20 (t, J = 6.7 Hz, 2H), 4.00 (t, J = 5.7 Hz, 2H), 2.91 (t, J = 5.5 Hz, 2H), 1.71-1.65 (m, 2H), 1.46-1.39 (m, 2H), 0.96 (t, J = 7.5 Hz, 3H); ^{13}C NMR ($CDCl_3$, 125 MHz): 167.0, 141.0, 135.6, 133.4, 132.7, 126.2, 126.1, 124.7, 120.0, 68.0, 65.1, 64.5, 30.7, 25.9, 19.2, 13.7; LC-MS (ESI) m/z 261 $[M+H]^+$; HRMS (ESI) m/z calcd for $C_{16}H_{21}O_3$ $[M^+H]$ 261.1485, found 261.1483.

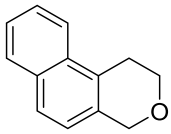

1,4-Dihydro-2*H*-benzo[*f*]isochromene (1k): To a flask containing lithium aluminum hydride (683.1 mg, 18.0 mmol) in dry THF (30 mL) was slowly added 2-(naphthalen-1-yl)acetic acid (2.79 g, 15.0 mmol) at 0 ^oC over a period of 10 min. Then the reaction mixture was warmed to room temperature and refluxed for 30 min. The excess lithium aluminum hydride was hydrolyzed by slow addition of 20% aqueous sodium hydroxide solution (20 mL). After filtration through a thin pad of celite, the filtrate was extracted with ethyl acetate (3 × 30 mL) and washed with brine (2 × 20 mL). The combined organic phase was dried over Na_2SO_4 and purified by column chromatography on silica gel to give 2-(naphthalen-1-yl)ethan-1-ol (2.25 g, 87%) as colorless liquid. A mixture of the 2-(naphthalen-1-yl)ethan-1-ol (1.37 g, 8 mmol), (chloromethoxy)ethane (1.13 g, 12 mmol) and N,N-diisopropylethylamine (2.07 g, 16 mmol) in dry dichloromethane (24 mL) was stirred for 6.5 h under N_2 at room temperature. The reaction mixture was then washed with brine (2 × 50 mL), dried over Na_2SO_4 and the solvent was removed in vacuo. The given crude acetal (0.69 g, 3.0 mmol) was dissolved in dry CH_3CN (9 mL) and trimethylsilyl trifluoromethanesulfonate (TMSOTf) (0.8 g, 3.6 mmol) was added at 0 ^oC. The reaction was carried out under N_2 for 14 h and quenched by the addition of $NaHCO_3$ (l.0 M, 10 mL). The organic phase was washed with brine (2 × 20 mL), dried with Na_2SO_4 and purified by column chromatography on silica gel to give product **1k** (421.8 mg, 66% yield for three steps) as white solid. M.p. 66-67 ^oC; IR (KBr, cm^{-1}): 3053, 2923, 2845, 2812, 1590, 1507, 1388, 1305, 1106, 1069, 990, 807, 737; ^{1}H NMR ($CDCl_3$, 500 MHz): δ 7.92 (d, J = 8.5 Hz, 1H), 7.83 (d, J = 8.0 Hz, 1H), 7.69 (d, J = 8.5 Hz, 1H), 7.56-7.53 (m, 1H), 7.50-7.47 (m, 1H), 7.11 (d, J = 8.5 Hz, 1H), 4.92 (s, 2H), 4.15 (t, J = 5.7 Hz, 2H), 3.17 (t, J = 5.5 Hz, 2H); ^{13}C NMR ($CDCl_3$, 125 MHz): 132.3, 132.1, 132.0, 128.6, 128.3, 126.3, 126.2, 125.3, 122.9, 122.5, 68.3, 65.2, 25.1; EI-MS m/z (%): 184 (100) $[M^+]$, 183 (20), 154 (55), 153 (35), 152 (32); HRMS (EI) m/z calcd for $C_{13}H_{12}O$ $[M]^+$ 184.0888, found 184.0884.

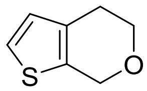

4,7-Dihydro-5*H*-thieno[2,3-*c*]pyran (1l) (Gonzalez-de-Castro et al., 2014)**:** The product **1l** was prepared following the general procedure as for **1k**. The reaction mixture of 2-(thiophen-3-yl)acetic acid (710.0 mg, 5.0 mmol) and lithium aluminum hydride (474.4 mg,

12.5 mmol) in dry THF (15 mL) was stirred at 0 °C for 8 h to afford 2-(thiophen-3-yl)ethan-1-ol as a crude. Then the mixture of the 2-(thiophen-3-yl)- ethan-1-ol (640.2 mg, 5.0 mmol), (chloromethoxy)ethane (695 μL, 7.5 mmol) and N,N-diiso- propylethylamine (1.6 mL, 10.0 mmol) in dry dichloromethane (15 mL) was stirred for 23 h under N_2 at room temperature to afford crude acetal product. Finally, the reaction mixture of acetal (258.1 mg, 1.5 mmol) and TMSOTf (50 μL, 0.26 mmol) in dry CH_3CN (9.0 mL) was stirred at 0 °C for 15 h to afford product **1l** (46.4 mg, 22% yield for three steps) as pale yellow liquid. IR (KBr, cm^{-1}): 2919, 2844, 1446, 1385, 1154, 1091, 1020, 974, 704; ^{1}H NMR ($CDCl_3$, 500 MHz): *δ* 7.15 (d, *J* = 5.0 Hz, 1H), 6.83 (d, *J* = 5.0 Hz, 1H), 4.83 (s, 2H), 3.95 (t, *J* = 5.7 Hz, 2H), 2.77-2.74 (m, 2H); ^{13}C NMR ($CDCl_3$, 125 MHz): 132.7, 127.0, 122.5, 65.6, 65.0, 26.1; EI-MS m/z (%): 140 (100) [M^+], 110 (72).

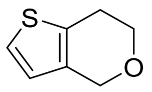

6,7-Dihydro-4*H*-thieno[3,2-*c*]pyran (1m): The product **1m** was prepared following the general procedure as for **1k**. The reaction mixture of 2-(thiophen-2-yl)acetic acid (1.1 g, 8.0 mmol) and lithium aluminum hydride (542.0 mg, 14.3 mmol) in dry Et_2O (30 mL) was stirred at 0 °C for 40 min, then refluxed for 6.5 h to afford 2-(thiophen-2-yl)ethan-1-ol as a crude. Then the mixture of the 2-(thiophen-2-yl)ethan-1-ol (947.4 mg, 7.4 mmol), (chloromethoxy)ethane (1.0 mL, 11.1 mmol) and N,N-diisopropylethylamine (2.5 mL, 14.8 mmol) in dry dichloromethane (30 mL) was stirred for 16 h under N_2 at room temperature to afford crude acetal product. Finally, the reaction mixture of acetal (279.1 mg, 1.5 mmol) and TMSOTf (130 μL, 0.68 mmol) in dry CH_3CN (9.0 mL) was stirred at 0 °C for 13.5 h to afford product **1m** (71.7 mg, 32% yield for three steps) as pale yellow liquid. IR (KBr, cm^{-1}): 3102, 2924, 2848, 1446, 1397, 1325, 1228, 1095, 1072, 966, 851, 705; ^{1}H NMR ($CDCl_3$, 500 MHz): *δ* 7.14 (d, *J* = 5.5 Hz, 1H), 6.76 (d, *J* = 5.0 Hz, 1H), 4.76 (t, *J* = 1.2 Hz, 2H), 4.00 (t, *J* = 5.5 Hz, 2H), 2.91 (t, *J* = 5.5 Hz, 2H); ^{13}C NMR ($CDCl_3$, 125 MHz): 133.7, 132.2, 123.6, 122.7, 66.5, 64.9, 25.5; EI-MS m/z (%): 140 (52) [M^+], 110 (100); HRMS (EI) m/z calcd for C_7H_8OS $[M]^+$ 140.0296, found 140.0294.

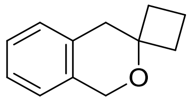

Spiro[cyclobutane-1,3'-isochroman] (1n): To a two-neck round bottom flask equipped with a reflux condenser under N_2 containing magnesium turnings (1.5 g, 60.0 mmol) in Et_2O (15 mL) and a particle of iodine was added dropwise (bromomethyl)benzene (5.1 g, 30.0 mmol) in Et_2O (15 mL) over 1 h. The mixture was stirred for 5 h under reflux. It was then allowed to cool to room temperature and transferred by syringe into a vial sealed with rubber stopper under a positive pressure of N_2. To a stirring solution of cyclobutanone (560.7 mg, 8.0 mmol) in dry Et_2O (24.0 mL), benzylmagnesium bromide (1.0 M in Et_2O, 12 mL) was added at 0 °C under N_2. Upon completion, water was added and the solution was extracted with ethyl acetate. The combined organic phase was washed with brine, dried over Na_2SO_4 and evaporated in vacuum to give the crude product 1-benzylcyclobutan-1-ol for further use. Following the general procedure as for **1k**, the mixture of crude 1-benzylcyclobutan-1-ol (1.13 g), (chloromethoxy)ethane (973 μL, 10.5 mmol) and *N*,*N*-diisopropylethylamine (2.3 mL, 14.0

mmol) in dry dichloromethane (24 mL) was stirred for 5 h under N_2 at room temperature. After reaction, the purified acetal (586.4 mg, 33% yield for two steps) was obtained by silica gel column chromatography. The reaction mixture of acetal (550.4 mg, 2.5 mmol) and TMSOTf (526 μL, 2.75 mmol) in dry CH_3CN (9.0 mL) was stirred at 0 ^oC, then warmed to room temperature and stirred for 10.5 h to afford product **1n** (284.4 mg, 61%) as colorless liquid. IR (KBr, cm^{-1}): 2976, 2933, 2835, 1692, 1550, 1532, 1505, 1266, 1091, 1047, 744; ^1H NMR ($CDCl_3$, 500 MHz): *δ* 7.17-7.11 (m, 3H), 6.98 (d, *J* = 8.5 Hz, 1H), 4.79 (s, 2H), 2.92 (s, 2H), 2.25-2.18 (m, 2H), 1.96-1.85 (m, 3H), 1.75-1.69 (m, 1H); ^{13}C NMR ($CDCl_3$, 125 MHz): 134.3, 132.7, 129.4, 126.3, 125.9, 123.9, 75.4, 63.3, 37.2, 32.4, 12.4; LC-MS (ESI) m/z 175 [M^+H]; HRMS (ESI) m/z calcd for $C_{12}H_{15}O$ $[M+H]^+$ 175.1117, found 175.1117.

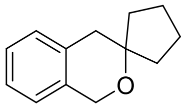

Spiro[cyclopentane-1,3'-isochroman] (1o): The product **1o** was prepared following the general procedure as for **1n**. The reaction mixture of cyclopentanone (672.0 mg, 8.0 mmol) and benzylmagnesium bromide (1.0 M in Et_2O, 12 mL) in dry Et_2O (24 mL) was stirred at 0 ^oC, then warmed to room temperature and stirred for 16 h to afford 1-benzylcyclopentan-1-ol as a crude. Then a mixture of the crude 1-benzylcyclopentan-1-ol (1.41 g), (chloromethoxy)ethane (1.1 mL, 12 mmol) and *N*,*N*-diisopropylethylamine (2.6 mL, 16 mmol) in dry dichloromethane (24 mL) was stirred for 5 h under N_2 at room temperature. After reaction, the purified acetal (580.2 mg, 29% yield for two steps) was obtained by silica gel column chromatography. Finally, the reaction mixture of acetal (749.3 mg, 3.2 mmol) and TMSOTf (0.68 mL, 3.52 mmol) in dry CH_3CN (11 mL) was stirred at 0 ^oC, then warmed to room temperature and stirred for 14 h to afford product **1o** (232.2 mg, 39%) as colorless liquid. IR (KBr, cm^{-1}): 2954, 1494, 1448, 1208, 1081, 744; ^1H NMR ($CDCl_3$, 500 MHz): *δ* 7.17-7.13 (m, 2H), 7.09-7.07 (m, 1H), 7.00-6.99 (m, 1H), 4.80 (s, 2H), 2.81 (s, 2H), 1.91-1.87 (m, 2H), 1.83-1.79 (m, 2H), 1.69-1.63 (m, 2H), 1.54-1.48 (m, 2H); ^{13}C NMR ($CDCl_3$, 125 MHz): 134.2, 133.6, 129.0, 126.1, 125.8, 123.9, 82.4, 63.3, 37.9, 36.4, 23.8; EI-MS m/z (%): 188 (12) [M^+], 104 (100); HRMS (EI) m/z calcd for $C_{13}H_{16}O$ $[M]^+$ 188.1201, found 188.1197.

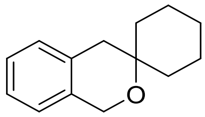

Spiro[cyclohexane-1,3'-isochroman] (1p): The product **1p** was prepared following the general procedure as for **1n**. The reaction mixture of cyclohexanone (783.0 mg, 8.0 mmol) and benzylmagnesium bromide (1.0 M in Et_2O, 12 mL) in dry Et_2O (24 mL) was stirred at 0 ^oC, then warmed to room temperature and stirred for 16 h to afford 1-benzylcyclohexan-1-ol as a crude. Then a mixture of the crude 1-benzylcyclohexan-1-ol (1.32 g), (chloromethoxy)ethane (973.0 μL, 10.5 mmol) and N,N-diisopropylethylamine (2.3 mL, 14.0 mmol) in dry dichloromethane (24 mL) was stirred for 5 h under N_2 at room temperature. After reaction, the purified acetal (761.6 mg, 40% yield for two steps) was obtained by silica gel column chromatography. Finally, the reaction mixture of acetal (570.8 mg, 2.3 mmol) and TMSOTf (444 μL, 2.53 mmol) in dry CH_3CN (9.0 mL) was stirred at 0 ^oC, then warmed to room temperature and stirred for 10.5 h to afford product **1p** (343.2 mg, 79%) as white solid. M.p. 50-52 ^oC; IR (KBr, cm^{-1}): 3032, 2850, 1444, 1075, 1026, 951, 748; ^1H NMR ($CDCl_3$, 500 MHz): *δ* 7.16-7.13 (m, 2H), 7.08-7.06 (m,

1H), 7.00-6.98 (m, 1H), 4.76 (s, 2H), 2.68 (s, 2H), 1.76-1.73 (m, 2H), 1.67-1.58 (m, 3H), 1.51-1.33 (m, 5H); ^{13}C NMR ($CDCl_3$, 125 MHz): 134.3, 132.7, 129.2, 126.2, 125.7, 123.9, 71.6, 62.1, 38.8, 34.8, 26.0, 21.9; LC-MS (ESI) m/z 203 $[M+H]^+$; HRMS (ESI) m/z calcd for $C_{14}H_{19}O$ $[M+H]^+$ 203.1430, found 203.1430.

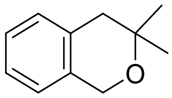

3,3-Dimethylisochroman (1q): The product **1q** was prepared following the general procedure as for **1n**. The reaction mixture of acetone (0.44 mL, 6.0 mmol) and benzylmagnesium bromide (1.0 M in Et_2O, 9 mL) in dry Et_2O (18 mL) was stirred at 0 ^oC, then warmed to room temperature and stirred for 5 h to afford product 2-methyl-1-phenylpropan-2-ol (636.0 mg, 71%). Then a mixture of the 2-methyl-1-phenylpropan-2-ol (630.4 mg, 4.2 mmol), (chloromethoxy)ethane (589 μL, 6.3 mmol) and N,N-diisopropylethylamine (1.4 mL, 8.4 mmol) in dry dichloromethane (12 mL) was stirred for 13 h under N_2 at room temperature to afford crude product acetal. Finally, the reaction mixture of acetal (624.4 mg, 3.0 mmol) and TMSOTf (638 μL, 3.3 mmol) in dry CH_3CN (9.0 mL) was stirred at 0 ^oC, then warmed to room temperature and stirred for 20 h to afford product **1q** (248.4 mg, 51% yield for two steps) as pale yellow oil. IR (KBr, cm^{-1}): 2968, 2925, 2847, 1549, 1500, 1456, 1368, 1212, 1080, 742; ^{1}H NMR ($CDCl_3$, 500 MHz): δ 7.18-7.14 (m, 2H), 7.09-7.07 (m, 1H), 7.03-6.99 (m, 1H), 4.80 (s, 2H), 2.72 (s, 2H), 1.29 (s, 6H); ^{13}C NMR ($CDCl_3$, 125 MHz): 133.9, 133.0, 129.1, 126.3, 125.8, 123.9, 70.8, 63.0, 39.6, 26.4; EI-MS m/z (%): 162 (5) $[M^+]$, 147 (5), 105 (12), 104 (100); HRMS (EI) m/z calcd for $C_{11}H_{14}O$ $[M]^+$ 162.1045, found 162.1048.

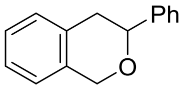

3-Phenylisochroman (1r): The product **1r** was prepared following the general procedure as for **1n**. The reaction mixture of benzaldehyde (530.6 mg, 5.0 mmol) and benzylmagnesium bromide (0.5 M in Et_2O, 15 mL) in dry Et_2O (10 mL) was stirred at 0 ^oC, then warmed to room temperature and stirred for 5.5 h to afford product 1,2-diphenylethan-1-ol (359.4 mg, 36%). Then a mixture of the 1,2-diphenylethan-1-ol (356.6 mg, 1.8 mmol), (chloromethoxy)ethane (0.25 mL, 2.7 mmol) and N,N-diisopropylethylamine (595 μL, 3.6 mmol) in dry dichloromethane (6 mL) was stirred for 19 h under N_2 at room temperature to afford acetal product (402.5 mg, 87%). Finally, the reaction mixture of acetal (402.2 mg, 1.57 mmol) and TMSOTf (334 μL, 1.7 mmol) in dry CH_3CN (6.0 mL) was stirred at 0 ^oC, then warmed to room temperature and stirred for 17 h to afford product **1r** (213.8 mg, 65%) as white solid. M.p. 75-76 ^oC; IR (KBr, cm^{-1}): 3024, 2910, 2851, 1487, 1444, 1367, 1084, 1027, 984, 735, 695; ^{1}H NMR ($CDCl_3$, 500 MHz): δ 7.49-7.47 (m, 2H), 7.42 (t, J = 7.5 Hz, 2H), 7.36-7.33 (m, 1H), 7.24-7.22 (m, 2H), 7.17-7.16 (m, 1H), 7.09-7.07 (m, 1H), 5.04 (s, 2H), 4.76 (dd, J = 11.0, 3.5 Hz, 1H), 3.14-3.08 (m, 1H), 3.00 (dd, J = 16.5, 3.0 Hz, 1H); ^{13}C NMR ($CDCl_3$, 125 MHz): 142.1, 134.5, 133.5, 128.8, 128.5, 127.7, 126.5, 126.2, 125.9, 124.2, 76.9, 68.7, 36.1; EI-MS m/z (%): 210 (5) $[M^+]$, 105 (12), 104 (100); HRMS (EI) m/z calcd for $C_{15}H_{14}O$ $[M]^+$ 210.1045, found 210.1044.

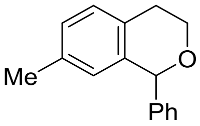

7-Methyl-1-phenylisochroman (3c): Following the procedure as for **3f** (see below), the reaction mixture of 7-methylisochroman (59.2 mg, 0.4 mmol), 2,3-dichloro-5,6-dicyano-benzoquinone (DDQ) (18.2 mg, 0.08 mmol), [bis(trifluoroacetoxy)iodo]benzene (PIFA) (172.0 mg, 0.4 mmol) in dry 1,2-dichloroethane (4.0 mL) was stirred at 80 ^{o}C under N_2 for 3.5 h, then phenyl- magnesium iodide was added at -15 ^{o}C and kept stirring for another 4.5 h to afford product **3c** (44.6 mg, 50%) as pale yellow oil. IR (KBr, cm^{-1}): 3027, 2961, 2919, 2852, 2723, 1606, 1499, 1453, 1274, 1090, 1020, 806, 748, 701; ^{1}H NMR ($CDCl_3$, 500 MHz): δ 7.46-7.41 (m, 5H), 7.16 (d, J = 8.0 Hz, 1H), 7.09 (d, J = 8.0 Hz, 1H), 6.68 (s, 1H), 5.80 (s, 1H), 4.30-4.26 (m, 1H), 4.02-3.97 (m, 1H), 3.20-3.18 (m, 1H), 2.88-2.84 (m, 1H), 2.29 (s, 3H); ^{13}C NMR ($CDCl_3$, 125 MHz): 142.4, 137.1, 135.5, 130.9, 129.0, 128.7, 128.5, 128.1, 127.6, 127.3, 79.7, 64.0, 28.6, 21.1; EI-MS m/z (%): 224 (100) [M^+], 223 (45), 209 (45), 178 (42), 147 (80), 119 (42); HRMS (EI) m/z calcd for $C_{16}H_{16}O$ $[M]^+$ 224.1201, found 224.1205.

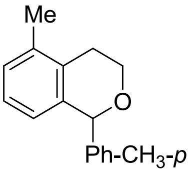

5-Methyl-1-(*p*-tolyl)isochroman (3f): To a two-neck round bottom flask equipped with a reflux condenser under N_2 containing magnesium turnings (466.6 mg, 19.2 mmol) in Et_2O (4.0 mL) was added 1-iodo-4-methylbenzene (3.5 g, 16.0 mmol) in Et_2O (4.0 mL) dropwise over 0.5 h. The mixture was stirred for 5.5 h under reflux. After cooling to room temperature, the produced *p*-tolylmagnesium iodide was then transferred by syringe into a vial sealed with rubber stopper under a positive pressure of N_2. The mixture of 5-methylisochroman (59.2 mg, 0.4 mmol), DDQ (18.2 mg, 0.08 mmol) and PIFA (172.0 mg, 0.4 mmol) in dry 1,2-dichloroethane (4.0 mL) in a test tube was stirred at 80 ^{o}C for 4 h under N_2, then *p*-tolylmagnesium iodide (2.0 M in Et_2O, 0.4 mL, 0.8 mmol) was added to the suspension at -15 ^{o}C. After stirring vigorously for 4 h at -15 ^{o}C, the reaction mixture was quenched with saturated aqueous $NaHCO_3$ and extracted with ethyl acetate. The organic layer was washed with brine, dried over Na_2SO_4 and purified by column chromatography on silica gel to give product **3f** (42.5 mg, 45%) as white solid. M.p. 70-71 ^{o}C; IR (KBr, cm^{-1}): 2929, 2872, 2812, 1909, 1462, 1266, 1106, 1061, 1011, 825, 796, 755; ^{1}H NMR ($CDCl_3$, 500 MHz): δ 7.18 (d, J = 8.0 Hz, 2H), 7.14 (d, J = 8.0 Hz, 2H), 7.05 (d, J = 7.0 Hz, 1H), 6.98 (t, J = 7.5 Hz, 1H), 6.61 (d, J = 8.0 Hz, 1H), 5.71 (s, 1H), 4.22-4.18 (m, 1H), 3.96-3.91 (m, 1H), 2.92-2.89 (m, 1H), 2.73-2.69 (m, 1H), 2.34 (s, 3H), 2.28 (s, 3H); ^{13}C NMR ($CDCl_3$, 125 MHz): 139.4, 137.7, 137.3, 136.1, 132.4, 129.0, 128.8, 127.8, 125.3, 124.6, 79.5, 63.4, 26.4, 21.2, 19.0; LC-MS (ESI) m/z 239 $[M+H]^+$; HRMS (ESI) m/z calcd for $C_{17}H_{19}O$ $[M+H]^+$ 239.1430, found 239.1429.

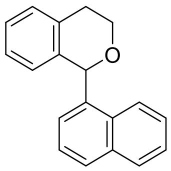

1-(Naphthalen-1-yl)isochroman (3g): Following the general procedure as for **3f**, the reaction mixture of isochroman (53.7 mg, 0.4 mmol), DDQ (18.2 mg, 0.08 mmol), PIFA (172.0 mg, 0.4 mmol) in dry 1,2-dichloroethane (4.0 mL) was stirred at 80 ^{o}C under N_2 for 4 h, then naphthalen- 1-ylmagnesium bromide was added at -15 ^{o}C and kept stirring for another 4 h to afford product **3g** (40.2 mg, 39%) as pale yellow solid. M.p. 122-123 ^{o}C; IR (KBr, cm^{-1}): 3019, 2972, 2923, 2874, 1589, 1495, 1450, 1363, 1263, 1080, 1040, 783, 740; ^{1}H NMR ($CDCl_3$, 500 MHz): δ 8.22-8.20 (m, 1H), 7.89-7.87 (m, 1H), 7.84 (d, *J* = 8.5 Hz, 1H), 7.49-7.47 (m, 2H), 7.43-7.40 (m, 1H), 7.31 (d, *J* = 7.0 Hz, 1H), 7.26-7.19 (m, 2H), 7.04 (t, *J* = 7.0 Hz, 1H), 6.76 (d, *J* = 7.5 Hz, 1H), 6.42 (s, 1H), 4.24-4.20 (m, 1H), 4.05-4.00 (m, 1H), 3.22-3.20 (m, 1H), 2.99-2.94 (m, 1H); ^{13}C NMR ($CDCl_3$, 125 MHz): 137.4, 137.2, 134.3, 133.8, 131.7, 129.0, 128.8, 128.6, 128.1, 126.7, 126.5, 126.2, 126.0, 125.6, 124.9 (2), 77.7, 63.7, 28.8; EI-MS m/z (%): 261 (20), 260 (100) [M^{+}], 259 (42), 133 (21); HRMS (EI) m/z calcd for $C_{19}H_{16}O$ $[M]^{+}$ 260.1201, found 260.1199.

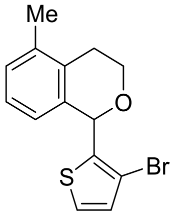

1-(3-Bromothiophen-2-yl)isochroman (3h): After stirring the mixture of 5-methylisochroman (59.2 mg, 0.4 mmol), DDQ (18.2 mg, 0.08 mmol) and PIFA (172.0 mg, 0.4 mmol) in dry 1,2-dichloroethane (4.0 mL) in a test tube at 80 ^{o}C for 4 h under N_2, 3-bromothiophene (78.3 mg, 0.48 mmol) was added to the suspension at room temperature. After stirring vigorously for 14 h at room temperature, the reaction mixture was purified by column chromatography on silica gel to give product **3h** (34.0 mg, 28%) as white solid. M.p. 86-87 ^{o}C; IR (KBr, cm^{-1}): 2967, 2921, 2851, 1731, 1458, 1262, 1094, 1016, 869, 804, 742, 701; ^{1}H NMR ($CDCl_3$, 500 MHz): δ 7.27 (d, *J* = 5.5 Hz, 1H), 7.09 (d, *J* = 7.0 Hz, 1H), 7.04 (t, *J* = 7.5 Hz, 1H), 7.00 (d, *J* = 5.0 Hz, 1H), 6.75 (d, *J* = 7.5 Hz, 1H), 6.18 (s, 1H), 4.31-4.27 (m, 1H), 4.02-3.97 (m, 1H), 2.96-2.90 (m, 1H), 2.71 (dt, *J* = 16.7 Hz, 3.8 Hz, 1H), 2.28 (s, 3H); ^{13}C NMR ($CDCl_3$, 125 MHz): 140.7, 136.2, 136.1, 132.0, 129.5, 128.5, 126.2, 125.7, 124.2, 110.8, 73.6, 63.9, 26.2, 19.0; EI-MS m/z (%): 310 (39) [M (^{81}Br)]$^{+}$, 308 (40) [M (^{79}Br)]$^{+}$, 229 (100), 201 (65), 184 (45); HRMS (EI) m/z calcd for $C_{14}H_{13}OSBr$ $[M]^{+}$ 307.9870, found 307.9871.

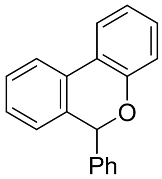

6-Phenyl-6*H*-benzo[*c*]chromene (3j): Following the general procedure as for **3f**, the reaction mixture of 6*H*-benzo[c]chromene (182.1 mg, 0.4 mmol), DDQ (18.2 mg, 0.08 mmol), PIFA

(172.0 mg, 0.4 mmol) in dry 1,2-dichloroethane (4.0 mL) was stirred at 80 °C under N_2 for 3 h, then phenylmagnesium iodide was added at -15 °C and kept stirring for another 9 h to afford product **3j** (79.0 mg, 77%) as white solid. M.p. 74-75 °C; IR (KBr, cm^{-1}): 3065, 3026, 2923, 1594, 1487, 1439, 1235, 1000, 743, 693, 607; ^1H NMR ($CDCl_3$, 500 MHz): *δ* 7.81-7.78 (m, 2H), 7.44-7.36 (m, 6H), 7.29-7.24 (m, 2H), 7.08 (td, *J* = 7.6, 1.0 Hz, 1H), 7.06-7.04 (m, 1H), 6.89 (d, *J* = 7.5 Hz, 1H), 6.20 (s, 1H); ^{13}C NMR ($CDCl_3$, 125 MHz): 153.7, 139.6, 134.0, 130.1, 129.6, 128.5 (2), 128.4, 128.1, 127.6, 126.3, 123.1, 122.8, 122.1, 117.9, 79.7; EI-MS m/z (%): 258 (55) [M^+], 257 (28), 181 (100); HRMS (EI) m/z calcd for $C_{19}H_{14}O$ $[M]^+$ 258.1045, found 258.1035.

C1 Functionalization of Isochromans, Related to Figure 2 and Figure 3.

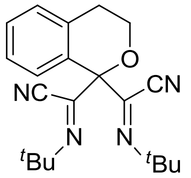

(1*E*,1*E*)-*N,N'*-Di-*tert*-butylisochroman-1,1-bis(carbimidoyl) cyanide (2a):
To a test tube, **1a** (39 μL, 0.3 mmol), ^{t}BuNC (170 μL, 1.5 mmol), AgOTf (7.8 mg, 0.03 mmol), DDQ (139.0 mg, 0.6 mmol), and dry PhCl (3.0 mL) were added in the glove box. The reaction mixture was stirred at 80 °C under N_2 for 3 h as monitored by TLC. Upon completion, the reaction mixture was cooled down to room temperature. After removed the solvent, the residue was purified by column chromatography on silica gel (petroleum ether/ethyl acetate = 100 : 1) to give product **2a** (64.1 mg, 61%) as white solid. M.p. 113-115 °C; IR (KBr, cm^{-1}): 2979, 2216, 1643, 1476, 1464, 1208, 914, 754; ^1H NMR ($CDCl_3$, 500 MHz): δ 7.29-7.27 (m, 1H), 7.19-7.15 (m, 2H), 6.90 (d, *J* = 7.5 Hz, 1H), 4.05 (t, *J* = 5.7 Hz, 2H), 2.97 (t, *J* = 5.5 Hz, 2H), 1.39 (s, 18H); ^{13}C NMR ($CDCl_3$, 125 MHz): 139.7, 134.8, 130.0, 129.2, 128.4, 127.8, 125.5, 111.2, 85.0, 62.0, 59.1, 29.1, 28.2; LC-MS (ESI) m/z 351 $[M+H]^+$; HRMS (ESI) m/z calcd for $C_{21}H_{27}ON_4$ $[M+H]^+$ 351.2179, found 351.2186.

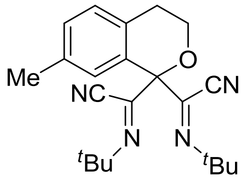

(1*E*,1*E*)-*N,N'*-Di-*tert*-butyl-7-methylisochroman-1,1-bis(carbimidoyl) cyanide (2b):
Following the general procedure as for **2a**, the reaction mixture of **1b** (44.5 mg, 0.3 mmol), ^{t}BuNC (170 μL, 1.5 mmol), AgOTf (7.8 mg, 0.03 mmol), and DDQ (139.0 mg, 0.6 mmol) in dry PhCl (3.0 mL) was stirred at 80 °C under N_2 for 4.5 h to afford product **2b** (79.3 mg, 73%) as white solid. M.p. 123-125 °C; IR (KBr, cm^{-1}): 2977, 2216, 1647, 1509, 1467, 1367, 1234, 1213, 1110, 1029, 948, 810; ^1H NMR ($CDCl_3$, 500 MHz): *δ* 7.07 (s, 2H), 6.69 (s, 1H), 4.02 (t, *J* = 5.5 Hz, 2H), 2.92 (t, *J* = 5.5 Hz, 2H), 2.28 (s, 3H), 1.39 (s, 18H); ^{13}C NMR ($CDCl_3$, 125 MHz): 139.6, 134.8, 131.6, 130.3, 129.2, 128.9, 127.4, 111.1, 84.9, 62.0, 58.9, 28.9, 27.7, 21.2; LC-MS (ESI) m/z 365 $[M+H]^+$; HRMS (ESI) m/z calcd for $C_{22}H_{29}ON_4$ $[M+H]^+$ 365.2336, found 365.2332.

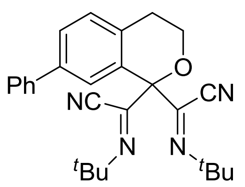

(1*E*,1*E*)-*N,N'*-Di-*tert*-butyl-7-phenylisochroman-1,1-bis(carbimidoyl) cyanide (2c): Following the general procedure as for **2a**, the reaction mixture of **1c** (63.0 mg, 0.3 mmol), ^{t}BuNC (170 μL, 1.5 mmol), AgOTf (7.8 mg, 0.03 mmol), and DDQ (139.0 mg, 0.6 mmol) in dry PhCl (3.0 mL) was stirred at 80 ^{o}C under N_2 for 4.5 h to afford product **2c** (81.7 mg, 64%) as white solid. M.p. 124-125 ^{o}C; IR (KBr, cm^{-1}): 2977, 2216, 1638, 1475, 1364, 1202, 1108, 762, 693; ^{1}H NMR ($CDCl_3$, 500 MHz): *δ* 7.53-7.51 (m, 3H), 7.45 (t, *J* = 7.5 Hz, 2H), 7.36 (t, *J* = 7.2 Hz, 1H), 7.28 (d, *J* = 8.0 Hz, 1H), 7.18 (s, 1H), 4.11 (t, *J* = 5.2 Hz, 2H), 3.03 (t, *J* = 5.2 Hz, 2H), 1.43 (s, 18H); ^{13}C NMR ($CDCl_3$, 125 MHz): 140.7, 139.6, 138.4, 133.8, 129.5, 128.8, 128.5, 128.1, 127.3, 126.8, 111.1, 85.1, 62.0, 59.1, 29.0, 27.8; LC-MS (ESI) m/z 427 [M+H]$^+$; HRMS (ESI) m/z calcd for $C_{27}H_{31}ON_4$ [M+H]$^+$ 427.2492, found 427.2489.

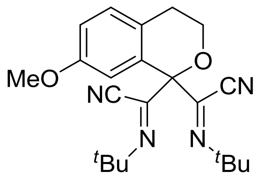

(1*E*,1*E*)-*N,N'*-Di-*tert*-butyl-7-methoxyisochroman-1,1-bis(carbimidoyl) cyanide (2d): Following the general procedure as for **2a**, the reaction mixture of **1d** (50.7 mg, 0.3 mmol), ^{t}BuNC (170 μL, 1.5 mmol), AgOTf (7.8 mg, 0.03 mmol), and DDQ (139.0 mg, 0.6 mmol) in dry PhCl (3.0 mL) was stirred at 80 ^{o}C under N_2 for 4.5 h to afford product **2d** (71.9 mg, 63%) as white solid. M.p. 124-125 ^{o}C; IR (KBr, cm^{-1}): 2977, 2216, 1646, 1508, 1467, 1322, 1240, 1211, 1104, 1029, 959, 820; ^{1}H NMR ($CDCl_3$, 500 MHz): *δ* 7.10 (d, *J* = 8.5 Hz, 1H), 6.85 (dd, *J* = 8.0, 2.5 Hz, 1H), 6.46 (d, *J* = 2.0 Hz, 1H) , 4.03 (t, *J* = 5.4 Hz, 2H), 3.72 (s, 3H), 2.90 (t, *J* = 5.4 Hz, 2H), 1.40 (s, 18H); ^{13}C NMR ($CDCl_3$, 125 MHz): 157.0, 139.6, 129.9, 128.6, 126.9, 115.3, 114.5, 111.0, 85.0, 62.1, 59.0, 55.2, 29.0, 27.2; LC-MS (ESI) m/z 381 [M+H]$^+$; HRMS (ESI) m/z calcd for $C_{22}H_{29}O_2N_4$ [M+H]$^+$ 381.2285, found 381.2293.

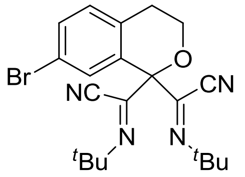

(1*E*,1*E*)-7-Bromo-*N,N'*-di-*tert*-butylisochroman-1,1-bis(carbimidoyl) cyanide (2e): Following the general procedure as for **2a**, the reaction mixture of **1e** (65.6 mg, 0.3 mmol), ^{t}BuNC (170 μL, 1.5 mmol), AgOTf (7.8 mg, 0.03 mmol), and DDQ (139.0 mg, 0.6 mmol) in dry PhCl (3.0 mL) was stirred at 80 ^{o}C under N_2 for 4.5 h to afford product **2e** (44.1 mg, 34%) as white solid. M.p. 108-110 ^{o}C; IR (KBr, cm^{-1}): 2975, 1727, 1645, 1483, 1454, 1366, 1212, 1089, 756, 697; ^{1}H NMR ($CDCl_3$, 500 MHz): *δ* 7.39 (dd, *J* = 8.5, 2.0 Hz, 1H), 7.07 (d, *J* = 8.0 Hz, 1H), 7.01 (d, *J* = 2.0 Hz, 1H) , 4.02 (t, *J* = 5.5 Hz, 2H), 2.92 (t, *J* = 5.5 Hz, 2H), 1.40 (s, 18H); ^{13}C NMR ($CDCl_3$, 125 MHz): 139.1, 133.6, 132.8, 131.4, 130.6, 129.8, 118.8, 110.8, 84.5, 61.7, 59.2, 28.9, 27.6; LC-MS (ESI) m/z (%): 431 (100) [M (^{81}Br)+H]$^+$, 429 (96) [M (^{79}Br)+H]$^+$; HRMS

(ESI) m/z calcd for $C_{21}H_{26}ON_4Br$ $[M+H]^+$ 429.1285, found 429.1281.

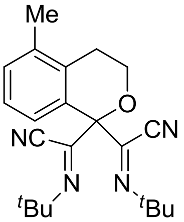

(1*E*,1*E*)-*N,N'*-Di-*tert*-butyl-5-methylisochroman-1,1-bis(carbimidoyl) cyanide (2f): Following the general procedure as for **2a**, the reaction mixture of **1f** (44.4 mg, 0.3 mmol), ^{t}BuNC (170 μL, 1.5 mmol), AgOTf (7.8 mg, 0.03 mmol), and DDQ (139.0 mg, 0.6 mmol) in dry PhCl (3.0 mL) was stirred at 80 ^{o}C under N_2 for 3.5 h to afford product **2f** (71.3 mg, 65%) as white solid. M.p. 103-105 ^{o}C; IR (KBr, cm^{-1}): 2976, 2220, 1643, 1467, 1367, 1211, 1096, 1028, 782; ^{1}H NMR ($CDCl_3$, 500 MHz): δ 7.14 (d, J = 7.0 Hz, 1H), 7.08 (t, J = 7.5 Hz, 1H), 6.75 (d, J = 8.0 Hz, 1H) , 4.06 (t, J = 5.5 Hz, 2H), 2.82 (t, J = 5.7 Hz, 2H), 2.27 (s, 3H), 1.39 (s, 18H); ^{13}C NMR ($CDCl_3$, 125 MHz): 139.8, 136.4, 133.3, 129.7, 127.7, 127.4, 124.8, 111.1, 85.1, 61.6, 58.9, 28.9, 25.6, 19.1; LC-MS (ESI) m/z 365 $[M+H]^+$; HRMS (ESI) m/z calcd for $C_{22}H_{29}ON_4$ $[M+H]^+$ 365.2336, found 365.2333.

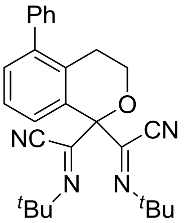

(1*E*,1*E*)-*N,N'*-Di-*tert*-butyl-5-phenylisochroman-1,1-bis(carbimidoyl) cyanide (2g): Following the general procedure as for **2a**, the reaction mixture of **1g** (63.0 mg, 0.3 mmol), ^{t}BuNC (170 μL, 1.5 mmol), AgOTf (7.8 mg, 0.03 mmol), and DDQ (139.0 mg, 0.6 mmol) in dry PhCl (3.0 mL) was stirred at 80 ^{o}C under N_2 for 5 h to afford product **2g** (85.1 mg, 67%) as white solid. M.p. 112-113 ^{o}C; IR (KBr, cm^{-1}): 2973, 2217, 1645, 1462, 1366, 1211, 1106, 1059, 755, 701; ^{1}H NMR ($CDCl_3$, 500 MHz): δ 7.44-7.41 (m, 2H), 7.38-7.34 (m, 3H), 7.26-7.23 (m, 2H), 6.95-6.93 (m, 1H), 3.94 (t, J = 5.5 Hz, 2H), 2.83 (t, J = 5.2 Hz, 2H), 1.42 (s, 18H); ^{13}C NMR ($CDCl_3$, 125 MHz): 141.9, 140.3, 139.7, 132.7, 129.7, 129.3, 129.2, 128.2, 127.7, 127.2, 125.0, 111.2, 85.2, 62.0, 59.0, 29.0, 27.3; LC-MS (ESI) m/z 427 $[M+H]^+$; HRMS (ESI) m/z calcd for $C_{27}H_{31}ON_4$ $[M+H]^+$ 427.2492, found 427.2485.

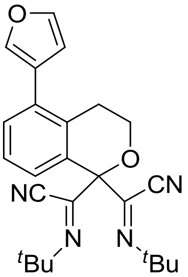

(1*E*,1*E*)-*N,N'*-Di-*tert*-butyl-5-(furan-3-yl)isochroman-1,1-bis(carbimidoyl) cyanide (2h): Following the general procedure as for **2a**, the reaction mixture of **1h** (60.1 mg, 0.3 mmol), ^{t}BuNC (170 μL, 1.5 mmol), AgOTf (7.8 mg, 0.03 mmol), and DDQ (139.0 mg, 0.6 mmol) in dry PhCl (3.0 mL) was stirred at 80 ^{o}C under N_2 for 12 h to afford product **2h** (83.2 mg, 67%) as

white solid. M.p. 114-115 °C; IR (KBr, cm^{-1}): 3130, 2977, 2216, 1641, 1506, 1466, 1364, 1234, 1210, 1108, 1055, 951, 791, 749; ^{1}H NMR ($CDCl_3$, 500 MHz): δ 7.53 (s, 1H), 7.49 (s, 1H), 7.31 (d, J = 7.5 Hz, 1H), 7.20 (t, J = 7.7 Hz, 1H), 6.88 (d, J = 7.5 Hz, 1H), 6.57 (s, 1H), 4.00 (t, J = 5.2 Hz, 2H), 2.96 (t, J = 5.2 Hz, 2H), 1.40 (s, 18H); ^{13}C NMR ($CDCl_3$, 125 MHz): 142.8, 140.2, 139.7, 132.9, 132.5, 129.4, 129.3, 127.9, 125.1, 124.2, 111.4, 111.1, 85.2, 61.9, 59.0, 29.0, 27.3; LC-MS (ESI) m/z 417 $[M+H]^+$; HRMS (ESI) m/z calcd for $C_{25}H_{29}O_2N_4$ $[M+H]^+$ 417.2285, found 417.2298.

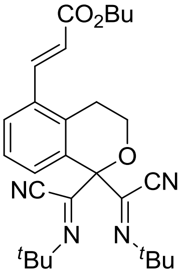

(*E*)-Butyl 3-(1,1-bis((*E*)-(*tert*-butylimino)(cyano)methyl)isochroman-5-yl)acrylate (2i): Following the general procedure as for **2a**, the reaction mixture of **1i** (78.1 mg, 0.3 mmol), ^{t}BuNC (170 μL, 1.5 mmol), AgOTf (7.7 mg, 0.03 mmol), and DDQ (139.0 mg, 0.6 mmol) in dry PhCl (3.0 mL) was stirred at 80 °C under N_2 for 12 h to afford product **2i** (62.0 mg, 43%) as white solid. M.p. 88-90 °C; IR (KBr, cm^{-1}): 2970, 1723, 1640, 1483, 1461, 1367, 1311, 1232, 1173, 1096, 1027, 977, 791; ^{1}H NMR ($CDCl_3$, 500 MHz): δ 7.90 (d, J = 16.0 Hz, 1H), 7.55 (d, J = 7.5 Hz, 1H), 7.21 (t, J = 7.7 Hz, 1H), 6.92 (d, J = 7.5 Hz, 1H), 6.38 (d, J = 15.5 Hz, 1H), 4.21 (t, J = 6.5 Hz, 2H), 4.06 (t, J = 5.7 Hz, 2H), 3.04 (t, J = 5.5 Hz, 2H), 1.71-1.66 (m, 2H), 1.45-1.42 (m, 2H), 1.39 (s, 18H), 0.96 (t, J = 7.2, 3H); ^{13}C NMR ($CDCl_3$, 125 MHz): 166.7, 140.6, 139.5, 134.1, 133.6, 131.7, 128.4, 126.7, 125.4, 120.8, 110.9, 85.1, 64.5, 61.3, 59.1, 30.7, 28.9, 25.5, 19.2, 13.7; LC-MS (ESI) m/z 477 $[M+H]^+$; HRMS (ESI) m/z calcd for $C_{28}H_{37}O_3N_4$ $[M+H]^+$ 477.2860, found 477.2854.

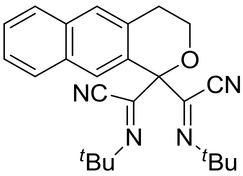

(1*E*,1*E*)-*N,N'*-Di-*tert*-butyl-3,4-dihydro-1*H*-benzo[*g*]isochromene-1,1-bis(carbimidoyl) cyanide (2j):

Following the general procedure as for **2a**, the reaction mixture of **1j** (55.2 mg, 0.3 mmol), ^{t}BuNC (170 μL, 1.5 mmol), AgOTf (7.8 mg, 0.03 mmol), and DDQ (139.0 mg, 0.6 mmol) in dry PhCl (3.0 mL) was stirred at 80 °C under N_2 for 4.5 h to afford product **2j** (60.8 mg, 51%) as white solid. M.p. 175-177 °C; IR (KBr, cm^{-1}): 2978, 2215, 1645, 1467, 1364, 1206, 1096, 1061, 914, 814, 751; ^{1}H NMR ($CDCl_3$, 500 MHz): δ 7.79 (d, J = 8.0 Hz, 1H), 7.76 (d, J = 8.0 Hz, 1H), 7.67 (d, J = 9.0 Hz, 1H), 7.36 (t, J = 7.2 Hz, 1H), 7.31-7.26 (m, 2H), 4.09 (t, J = 5.7 Hz, 2H), 3.17 (t, J = 5.5 Hz, 2H), 1.36 (s, 18H); ^{13}C NMR ($CDCl_3$, 125 MHz): 138.2, 134.7, 132.9, 131.0, 130.1, 128.5, 127.5 (2), 124.8 (2), 124.2, 111.1, 85.7, 60.6, 59.4, 29.4, 29.0; LC-MS (ESI) m/z 401 $[M+H]^+$; HRMS (ESI) m/z calcd for $C_{25}H_{29}ON_4$ $[M+H]^+$ 401.2336, found 401.2335.

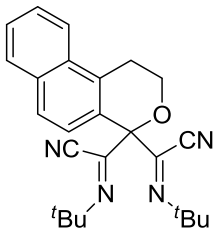

(1*E*,1*E*)-*N,N'*-Di-*tert*-butyl-1,2-dihydro-4*H*-benzo[*f*]isochromene-4,4-bis(carbimidoyl) cyanide (2k):

Following the general procedure as for **2a**, the reaction mixture of **1k** (55.2 mg, 0.3 mmol), ^{t}BuNC (170 μL, 1.5 mmol), AgOTf (7.7 mg, 0.03 mmol), and DDQ (139.0 mg, 0.6 mmol) in dry PhCl (3.0 mL) was stirred at 80 ^{o}C under N_2 for 4.5 h to afford product **2k** (69.5 mg, 58%) as white solid. M.p. 140-142 ^{o}C; IR (KBr, cm^{-1}): 2978, 2220, 1643, 1464, 1367, 1209, 1099, 1062, 810; ^{1}H NMR ($CDCl_3$, 500 MHz): *δ* 7.99 (d, *J* = 8.0 Hz, 1H), 7.85-7.83 (m, 1H), 7.64 (d, *J* = 9.0 Hz, 1H), 7.59-7.53 (m, 2H), 7.00 (d, *J* = 9.0 Hz, 1H), 4.20 (t, *J* = 5.5 Hz, 2H), 3.33 (t, *J* = 5.5 Hz, 2H), 1.40 (s, 18H); ^{13}C NMR ($CDCl_3$, 125 MHz): 139.7, 132.8, 131.6, 128.5, 126.6, 126.4, 126.3, 125.3 (2), 123.1, 111.0, 85.3, 61.4, 59.2, 29.0, 24.7; LC-MS (ESI) m/z 401 [M+H]$^+$; HRMS (ESI) m/z calcd for $C_{25}H_{29}ON_4$ [M+H]$^+$ 401.2336, found 401.2335.

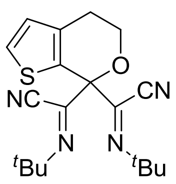

(1*E*,1*E*)-*N,N'*-Di-*tert*-butyl-4,5-dihydro-7*H*-thieno[2,3-*c*]pyran-7,7-bis(carbimidoyl) cyanide (2l):

Following the general procedure as for **2a**, the reaction mixture of **1l** (42.0 mg, 0.3 mmol), ^{t}BuNC (170 μL, 1.5 mmol), AgOTf (7.7 mg, 0.03 mmol), and DDQ (139.0 mg, 0.6 mmol) in dry PhCl (3.0 mL) was stirred at 80 ^{o}C under N_2 for 10 h to afford product **2l** (44.5 mg, 42%) as white solid. M.p. 110-112 ^{o}C; IR (KBr, cm^{-1}): 3101, 2976, 2215, 1642, 1468, 1367, 1236, 1208, 1064, 1020, 956, 891, 738; ^{1}H NMR ($CDCl_3$, 500 MHz): *δ* 7.39 (d, *J* = 5.0 Hz, 1H), 6.89 (d, *J* = 5.0 Hz, 1H), 4.07 (t, *J* = 5.5 Hz, 2H), 2.88 (t, *J* = 5.2 Hz, 2H), 1.40 (s, 18H); ^{13}C NMR ($CDCl_3$, 125 MHz): 139.4, 137.1, 128.6, 127.4, 126.4, 110.6, 84.7, 62.4, 59.1, 28.9, 25.7; LC-MS (ESI) m/z 357 [M+H]$^+$; HRMS (ESI) m/z calcd for $C_{19}H_{25}ON_4S$ [M+H]$^+$ 357.1744, found 357.1739.

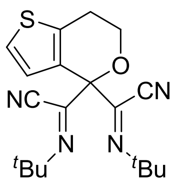

(1*E*,1*E*)-*N,N'*-Di-*tert*-butyl-6,7-dihydro-4*H*-thieno[3,2-*c*]pyran-4,4-bis(carbimidoyl) cyanide (2m):

Following the general procedure as for **2a**, the reaction mixture of **1m** (42.0 mg, 0.3 mmol), ^{t}BuNC (170 μL, 1.5 mmol), AgOTf (7.7 mg, 0.03 mmol), and DDQ (139.0 mg, 0.6 mmol) in dry PhCl (3.0 mL) was stirred at 80 ^{o}C under N_2 for 7.5 h to afford product **2m** (41.2 mg, 39%) as white solid. M.p. 103-105 ^{o}C; IR (KBr, cm^{-1}): 3116, 2972, 2212, 1642, 1466, 1366, 1236, 1209, 1087, 1017, 948, 867, 724; ^{1}H NMR ($CDCl_3$, 500 MHz): *δ* 7.09 (d, *J* = 5.0 Hz, 1H), 6.65 (d, *J* =

5.0 Hz, 1H), 4.07 (t, J = 5.5 Hz, 2H), 3.01 (t, J = 5.2 Hz, 2H), 1.39 (s, 18H); ^{13}C NMR ($CDCl_3$, 125 MHz): 139.1, 137.0, 127.7, 126.7, 122.2, 110.8, 84.6, 62.1, 59.1, 29.0, 24.9; LC-MS (ESI) m/z 357 $[M+H]^+$; HRMS (ESI) m/z calcd for $C_{19}H_{25}ON_4S$ $[M+H]^+$ 357.1744, found 357.1741.

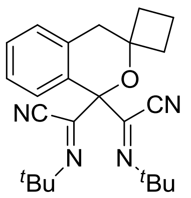

(1'*E*,1'*E*)-*N',N''*-Di-*tert*-butylspiro[cyclobutane-1,3'-isochroman]-1',1'-bis(carbimidoyl) cyanide (2n):

Following the general procedure as for **2a**, the reaction mixture of **1n** (52.3 mg, 0.3 mmol), ^{t}BuNC (170 μL, 1.5 mmol), AgOTf (7.7 mg, 0.03 mmol), and DDQ (139.0 mg, 0.6 mmol) in dry PhCl (3.0 mL) was stirred at 80 ^{o}C under N_2 for 5.5 h to afford product **2n** (62.0 mg, 53%) as white solid. M.p. 82-83 ^{o}C; IR (KBr, cm^{-1}): 2976, 2217, 1645, 1462, 1364, 1211, 1108, 1072, 754; ^{1}H NMR ($CDCl_3$, 500 MHz): δ 7.30-7.27 (m, 1H), 7.21-7.16 (m, 2H), 6.90 (d, J = 8.0 Hz, 1H), 3.06 (s, 2H), 2.32-2.25 (m, 2H), 1.92-1.84 (m, 3H), 1.66-1.60 (m, 1H), 1.37 (s, 18H); ^{13}C NMR ($CDCl_3$, 125 MHz): 140.2, 133.9, 130.2, 129.4, 128.4, 127.4, 125.3, 111.3, 84.4, 76.2, 58.6, 37.0, 34.9, 28.8, 13.4; LC-MS (ESI) m/z 391 $[M+H]^+$; HRMS (ESI) m/z calcd for $C_{24}H_{31}ON_4$ $[M+H]^+$ 391.2492, found 391.2491.

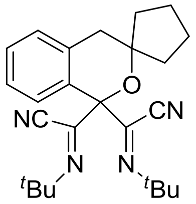

(1'*E*,1'*E*)-*N',N''*-Di-*tert*-butylspiro[cyclopentane-1,3'-isochroman]-1',1'-bis(carbimidoyl) cyanide (2o):

Following the general procedure as for **2a**, the reaction mixture of **1o** (56.4 mg, 0.3 mmol), ^{t}BuNC (170 μL, 1.5 mmol), AgOTf (7.7 mg, 0.03 mmol), and DDQ (139.0 mg, 0.6 mmol) in dry PhCl (3.0 mL) was stirred at 80 ^{o}C under N_2 for 4.5 h to afford product **2o** (62.5 mg, 52%) as white solid. M.p. 75-77 ^{o}C; IR (KBr, cm^{-1}): 2970, 2224, 1649, 1462, 1363, 1211, 1106, 1075, 921, 758; ^{1}H NMR ($CDCl_3$, 500 MHz): δ 7.28-7.26 (m, 1H), 7.19-7.16 (m, 2H), 6.99 (d, J = 8.0 Hz, 1H), 2.99 (s, 2H), 1.94-1.92 (m, 4H), 1.66-1.64 (m, 2H), 1.54-1.50 (m, 2H), 1.37 (s, 18H); ^{13}C NMR ($CDCl_3$, 125 MHz): 140.6, 135.2, 130.5, 128.9, 128.3, 127.2, 125.2, 111.5, 85.5, 84.2, 58.6, 38.0 (2), 28.8, 23.2; LC-MS (ESI) m/z 405 $[M+H]^+$; HRMS (ESI) m/z calcd for $C_{25}H_{33}ON_4$ $[M+H]^+$ 405.2649, found 405.2649.

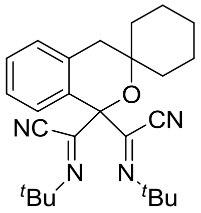

(1'*E*,1'*E*)-*N',N''*-Di-*tert*-butylspiro[cyclohexane-1,3'-isochroman]-1',1'-bis(carbimidoyl) cyanide (2p):

Following the general procedure as for **2a**, the reaction mixture of **1p** (60.7 mg, 0.3 mmol), ^{t}BuNC (170 μL, 1.5 mmol), AgOTf (7.7 mg, 0.03 mmol), and DDQ (139.0 mg, 0.6 mmol) in dry PhCl (3.0 mL) was stirred at 80 ^{o}C under N_2 for 5.5 h to afford product **2p** (72.5 mg, 57%) as white solid. M.p. 119-120 ^{o}C; IR (KBr, cm^{-1}): 2936, 2212, 1644, 1455, 1366, 1236, 1209, 1070, 752; ^{1}H NMR ($CDCl_3$, 500 MHz): *δ* 7.27 (t, *J* = 7.7 Hz, 1H), 7.19-7.14 (m, 2H), 6.96 (d, *J* = 8.0 Hz, 1H), 2.90 (s, 2H), 1.77-1.71 (m, 4H), 1.52-1.42 (m, 6H), 1.37 (s, 18H); ^{13}C NMR ($CDCl_3$, 125 MHz): 140.5, 134.0, 130.2, 129.1, 128.4, 127.3, 125.2, 111.7, 83.7, 75.8, 58.6, 38.6, 36.8, 28.8, 25.7, 22.4; LC-MS (ESI) m/z 419 [M+H]$^+$; HRMS (ESI) m/z calcd for $C_{26}H_{35}ON_4$ [M+H]$^+$ 419.2805, found 419.2799.

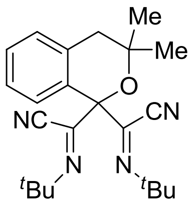

(1*E*,1*E*)-*N,N'*-Di-*tert*-butyl-3,3-dimethylisochroman-1,1-bis(carbimidoyl) cyanide (2q): Following the general procedure as for **2a**, the reaction mixture of **1q** (48.6 mg, 0.3 mmol), ^{t}BuNC (170 μL, 1.5 mmol), AgOTf (7.7 mg, 0.03 mmol), and DDQ (139.0 mg, 0.6 mmol) in dry PhCl (3.0 mL) was stirred at 80 ^{o}C under N_2 for 5 h to afford product **2q** (60.1 mg, 53%) as white solid. M.p. 101-103 ^{o}C; IR (KBr, cm^{-1}): 2979, 2212, 1646, 1465, 1371, 1208, 1076, 921, 758; ^{1}H NMR ($CDCl_3$, 500 MHz): *δ* 7.27-7.23 (m, 1H), 7.16 (t, *J* = 7.5 Hz, 1H), 7.12 (d, *J* = 7.5 Hz, 1H), 6.97 (d, *J* = 7.5 Hz, 1H), 2.86 (s, 2H), 1.34 (s, 18H) , 1.31 (s, 6H); ^{13}C NMR ($CDCl_3$, 125 MHz): 140.6, 134.2, 130.3, 129.1, 128.5, 126.8, 125.2, 111.4, 83.8, 74.3, 58.5, 40.1, 28.8, 28.3; LC-MS (ESI) m/z 379 [M+H]$^+$; HRMS (ESI) m/z calcd for $C_{23}H_{31}ON_4$ [M+H]$^+$ 379.2492, found 379.2491.

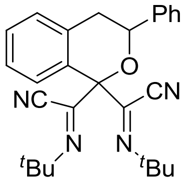

(1*E*,1*E*)-*N,N'*-Di-*tert*-butyl-3-phenylisochroman-1,1-bis(carbimidoyl) cyanide (2r): Following the general procedure as for **2a**, the reaction mixture of **1r** (63.0 mg, 0.3 mmol), ^{t}BuNC (170 μL, 1.5 mmol), AgOTf (7.7 mg, 0.03 mmol), and DDQ (139.0 mg, 0.6 mmol) in dry PhCl (3.0 mL) was stirred at 80 ^{o}C under N_2 for 5.5 h to afford product **2r** (79.9 mg, 62%) as white solid. M.p. 145-146 ^{o}C; IR (KBr, cm^{-1}): 2975, 2216, 1646, 1457, 1367, 1233, 1210, 1069, 916, 748, 692; ^{1}H NMR ($CDCl_3$, 500 MHz): *δ* 7.55 (d, *J* = 7.5 Hz, 2H), 7.42 (t, *J* = 7.5 Hz, 2H), 7.36-7.33 (m, 1H), 7.33-7.30 (m, 1H), 7.23 (t, *J* = 6.7 Hz, 2H), 6.99 (d, *J* = 7.5 Hz, 1H), 4.78 (dd, *J* = 11.5, 2.5 Hz, 1H), 3.34-3.28 (m, 1H), 3.03 (dd, *J* = 16.5, 2.5 Hz, 1H), 1.44 (s, 9H), 1.37 (s, 9H); ^{13}C NMR ($CDCl_3$, 125 MHz): 140.1, 139.7, 139.5, 135.1, 129.8, 129.1, 128.6, 128.5, 128.3, 128.2, 127.5, 126.3, 125.9, 125.7, 111.6, 110.6, 86.2, 73.5, 59.3, 58.8, 35.6, 29.0 (2); LC-MS (ESI) m/z 427 [M+H]$^+$; HRMS (ESI) m/z calcd for $C_{27}H_{31}ON_4$ [M+H]$^+$ 427.2492, found 427.2493.

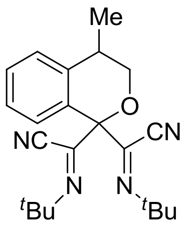

(1*E*,1*E*)-*N,N'*-Di-*tert*-butyl-4-methylisochroman-1,1-bis(carbimidoyl) cyanide (2s):
Following the general procedure as for **2a**, the reaction mixture of **1s** (44.4 mg, 0.3 mmol), ^{t}BuNC (170 μL, 1.5 mmol), AgOTf (7.7 mg, 0.03 mmol), and DDQ (139.0 mg, 0.6 mmol) in dry PhCl (3.0 mL) was stirred at 80 °C under N_2 for 5 h to afford product **2s** (59.7 mg, 55%) as white solid. M.p. 126-127 °C; IR (KBr, cm^{-1}): 2974, 2212, 1644, 1474, 1368, 1234, 1211, 1113, 981, 959, 755; ^{1}H NMR ($CDCl_3$, 500 MHz): *δ* 7.32-7.29 (m, 1H), 7.26 (d, *J* = 5.0 Hz, 1H), 7.18-7.15 (m, 1H), 6.88 (d, *J* = 7.5 Hz, 1H), 3.96 (dd, *J* = 11.0, 3.5 Hz, 1H), 3.87 (dd, *J* = 11.5, 4.0 Hz, 1H), 2.98-2.92 (m, 1H), 1.45 (d, *J* = 7.0 Hz, 3H), 1.42 (s, 9H), 1.36 (s, 9H); ^{13}C NMR ($CDCl_3$, 125 MHz): 140.1, 139.6, 139.5, 129.7, 128.5, 128.3, 127.0, 125.2, 111.4, 110.9, 85.4, 67.4, 59.1, 58.8, 32.0, 28.9, 20.1; LC-MS (ESI) m/z 365 [M+H]$^+$; HRMS (ESI) m/z calcd for $C_{22}H_{29}ON_4$ [M+H]$^+$ 365.2336, found 365.2332.

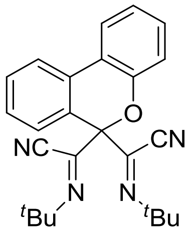

(6*E*,6*E*)-*N,N'*-Di-*tert*-butyl-6*H*-benzo[*c*]chromene-6,6-bis(carbimidoyl) cyanide (2t):
Following the general procedure as for **2a**, the reaction mixture of **1t** (54.7 mg, 0.3 mmol), ^{t}BuNC (170 μL, 1.5 mmol), AgOTf (7.7 mg, 0.03 mmol), and DDQ (139.0 mg, 0.6 mmol) in dry PhCl (3.0 mL) was stirred at 80 °C under N_2 for 5.5 h to afford product **2t** (87.1 mg, 73%) as white solid. M.p. 158-159 °C; IR (KBr, cm^{-1}): 2978, 2216, 1645, 1471, 1446, 1364, 1236, 1204, 1059, 1035, 752; ^{1}H NMR ($CDCl_3$, 500 MHz): *δ* 7.81 (d, *J* = 8.0 Hz, 1H), 7.70 (d, *J* = 8.0 Hz, 1H), 7.50 (t, *J* = 7.7 Hz, 1H), 7.36 (t, *J* = 7.5 Hz, 1H), 7.30-7.27 (m, 1H), 7.21 (d, *J* = 8.0 Hz, 1H), 7.10 (t, *J* = 7.5 Hz, 1H), 7.02 (d, *J* = 8.0 Hz, 1H), 1.33 (s, 18H); ^{13}C NMR ($CDCl_3$, 125 MHz): 150.3, 137.2, 130.0, 129.9, 129.8, 127.7, 127.2, 126.8, 123.3, 122.9, 122.7, 121.8, 118.7, 110.7, 86.6, 59.3, 28.9; LC-MS (ESI) m/z 399 [M+H]$^+$; HRMS (ESI) m/z calcd for $C_{25}H_{27}ON_4$ [M+H]$^+$ 399.2179, found 399.2178.

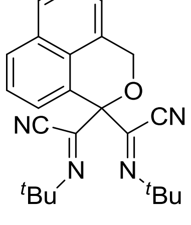

(1*E*,1*E*)-*N,N'*-Di-*tert*-butyl-1*H*,3*H*-benzo[*de*]isochromene-1,1-bis(carbimidoyl) cyanide (2u):
Following the general procedure as for **2a**, the reaction mixture of **1u** (51.1 mg, 0.3 mmol), ^{t}BuNC (170 μL, 1.5 mmol), AgOTf (7.7 mg, 0.03 mmol), and DDQ (139.0 mg, 0.6 mmol) in dry

PhCl (3.0 mL) was stirred at 80 °C under N_2 for 12 h to afford product **2u** (53.7 mg, 46%) as white solid. M.p. 146-148 °C; IR (KBr, cm^{-1}): 2975, 2216, 1641, 1464, 1365, 1207, 1068, 1037, 821, 766; ^1H NMR ($CDCl_3$, 500 MHz): *δ* 7.89 (d, *J* = 8.0 Hz, 1H), 7.82 (d, *J* = 8.0 Hz, 1H), 7.53-7.47 (m, 2H), 7.26-7.25 (m, 1H), 7.12 (d, *J* = 7.0 Hz, 1H), 5.20 (s, 2H), 1.42 (s, 18H); ^{13}C NMR ($CDCl_3$, 125 MHz): 139.2, 132.9, 129.6, 128.8, 127.2, 126.1, 125.7, 125.6, 125.4, 125.1, 120.8, 111.0, 85.7, 64.7, 59.3, 29.0; LC-MS (ESI) m/z 387 $[M+H]^+$; HRMS (ESI) m/z calcd for $C_{24}H_{27}ON_4$ $[M+H]^+$ 387.2179, found 387.2176.

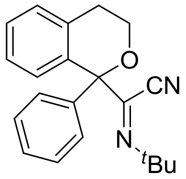

(*E*)-*N*-(*tert*-Butyl)-1-phenylisochroman-1-carbimidoyl cyanide (4a):

To a sealed tube, **3a** (63.1 mg, 0.3 mmol), ^{t}BuNC (170 μL, 1.5 mmol), AgOTf (7.7 mg, 0.03 mmol), and DDQ (139.0 mg, 0.6 mmol) in dry PhCl (3.0 mL) were added in the glove box. The mixture was stirred at 100 °C for 19 h under N_2. The reaction mixture was cooled down to room temperature and purified by column chromatography on silica gel to give product **4a** (64.5 mg, 68%) as white solid. M.p. 121-123 °C; IR (KBr, cm^{-1}): 2973, 2208, 1643, 1482, 1449, 1361, 1213, 1092, 1048, 919, 758, 695; ^1H NMR ($CDCl_3$, 500 MHz): *δ* 7.31-7.27 (m, 4H), 7.23-7.18 (m, 4H), 7.05 (d, *J* = 8.0 Hz, 1H), 4.04-4.00 (m, 1H), 3.90-3.85 (m, 1H), 3.18-3.12 (m, 1H), 2.88-2.84 (m, 1H), 1.40 (s, 9H); ^{13}C NMR ($CDCl_3$, 125 MHz): 143.1, 141.7, 134.7, 132.8, 129.3, 129.2, 128.9, 127.9, 127.7, 127.6, 125.5, 111.9, 84.9, 60.6, 58.5, 29.0, 28.3; LC-MS (ESI) m/z 319 $[M+H]^+$; HRMS (ESI) m/z calcd for $C_{21}H_{23}ON_2$ $[M+H]^+$ 319.1805, found 319.1802.

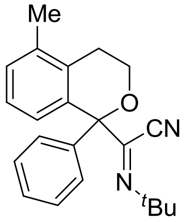

(*E*)-*N*-(*tert*-Butyl)-5-methyl-1-phenylisochroman-1-carbimidoyl cyanide (4b):

Following the general procedure as for **4a**, the reaction mixture of **3b** (67.3 mg, 0.3 mmol), ^{t}BuNC (170 μL, 1.5 mmol), AgOTf (7.7 mg, 0.03 mmol), and DDQ (139.0 mg, 0.6 mmol) in dry PhCl (3.0 mL) was stirred at 100 °C for 24 h to afford product **4b** (67.1 mg, 67%) as white solid. M.p. 127-128 °C; IR (KBr, cm^{-1}): 2977, 2216, 1636, 1482, 1461, 1365, 1233, 1208, 1093, 1053, 920, 784, 697; ^1H NMR ($CDCl_3$, 500 MHz): *δ* 7.31-7.28 (m, 3H), 7.23-7.21 (m, 2H), 7.16 (d, *J* = 7.0 Hz, 1H), 7.10 (t, *J* = 7.5 Hz, 1H), 6.90 (d, *J* = 8.0 Hz, 1H), 4.06-4.02 (m, 1H), 3.84-3.79 (m, 1H), 2.98-2.96 (m, 1H), 2.70-2.65 (m, 1H), 2.30 (s, 3H), 1.39 (s, 9H); ^{13}C NMR ($CDCl_3$, 125 MHz): 143.3, 141.7, 136.8, 133.4, 132.3, 129.3, 129.1, 127.9, 127.5, 126.8, 125.0, 112.0, 85.2, 60.2, 58.5, 29.0, 25.9, 19.2; LC-MS (ESI) m/z 333 $[M+H]^+$; HRMS (ESI) m/z calcd for $C_{22}H_{25}ON_2$ $[M+H]^+$ 333.1961, found 333.1961.

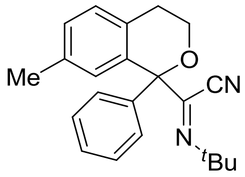

(*E*)-*N*-(*tert*-Butyl)-7-methyl-1-phenylisochroman-1-carbimidoyl cyanide (4c):
Following the general procedure as for **4a**, the reaction mixture of **3c** (68.7 mg, 0.3 mmol), ^{t}BuNC (170 μL, 1.5 mmol), AgOTf (7.7 mg, 0.03 mmol), and DDQ (139.0 mg, 0.6 mmol) in dry PhCl (3.0 mL) was stirred at 100 °C for 24 h to afford product **4c** (72.8 mg, 73%) as white solid. M.p. 153-154 °C; IR (KBr, cm^{-1}): 2983, 2203, 1636, 1497, 1452, 1365, 1209, 1083, 1048, 921, 756, 695; ^{1}H NMR ($CDCl_3$, 500 MHz): δ 7.30-7.28 (m, 3H), 7.19-7.17 (m, 2H), 7.11-7.06 (m, 2H), 6.82 (s, 1H), 3.99-3.95 (m, 1H), 3.81-3.76 (m, 1H), 3.10-3.08 (m, 1H), 2.74 (dt, *J* = 16.0, 3.7 Hz, 1H), 2.27 (s, 3H), 1.38 (s, 9H); ^{13}C NMR ($CDCl_3$, 125 MHz): 143.2, 141.7, 135.0, 132.4, 131.8, 129.4, 129.2 (2), 128.7, 127.9, 127.6, 112.0, 84.9, 60.6, 58.5, 29.0, 27.9, 21.2; LC-MS (ESI) m/z 333 $[M+H]^+$; HRMS (ESI) m/z calcd for $C_{22}H_{25}ON_2$ $[M+H]^+$ 333.1961, found 333.1967.

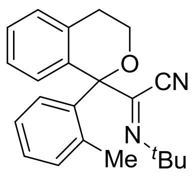

(*E*)-*N*-(*tert*-Butyl)-1-(*o*-tolyl)isochroman-1-carbimidoyl cyanide (4d):
Following the general procedure as for **4a**, the reaction mixture of **3d** (67.3 mg, 0.3 mmol), ^{t}BuNC (170 μL, 1.5 mmol), AgOTf (7.7 mg, 0.03 mmol), and DDQ (139.0 mg, 0.6 mmol) in dry PhCl (3.0 mL) was stirred at 100 °C for 24 h to afford product **4d** (70.7 mg, 71%) as white solid. M.p. 112-113 °C; IR (KBr, cm^{-1}): 2973, 2930, 2212, 1636, 1477, 1457, 1366, 1210, 1093, 1046, 919, 752; ^{1}H NMR ($CDCl_3$, 500 MHz): δ 7.28 (t, *J* = 7.5 Hz, 1H), 7.23-7.17 (m, 4H), 7.03-6.99 (m, 2H), 6.77 (d, *J* = 7.5 Hz, 1H), 4.03-3.99 (m, 1H), 3.76-3.71 (m, 1H), 3.21-3.17 (m, 1H), 2.77-2.74 (m, 1H), 2.32 (s, 3H), 1.39 (s, 9H); ^{13}C NMR ($CDCl_3$, 125 MHz): 143.2, 139.5, 138.1, 135.3, 132.8, 132.2, 130.6, 129.5 (2), 128.1, 127.7, 125.4, 124.3, 111.9, 86.1, 60.2, 58.4, 28.9, 28.1, 22.2; LC-MS (ESI) m/z 333 $[M+H]^+$; HRMS (ESI) m/z calcd for $C_{22}H_{25}ON_2$ $[M+H]^+$ 333.1961, found 333.1968.

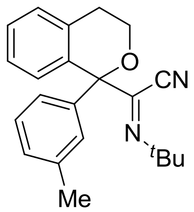

(*E*)-*N*-(*tert*-Butyl)-1-(*m*-tolyl)isochroman-1-carbimidoyl cyanide (4e):
Following the general procedure as for **4a**, the reaction mixture of **3e** (67.3 mg, 0.3 mmol), ^{t}BuNC (170 μL, 1.5 mmol), AgOTf (7.7 mg, 0.03 mmol), and DDQ (139.0 mg, 0.6 mmol) in dry PhCl (3.0 mL) was stirred at 100 °C for 21 h to afford product **4e** (74.7 mg, 75%) as white solid. M.p. 123-125 °C; IR (KBr, cm^{-1}): 2971, 2208, 1646, 1480, 1358, 1208, 1091, 1048, 922, 755, 699; ^{1}H NMR ($CDCl_3$, 500 MHz): δ 7.26-7.24 (m, 1H), 7.20-7.14 (m, 3H), 7.10 (d, *J* = 7.5 Hz, 1H), 7.05-7.01 (m, 2H), 7.00 (d, *J* = 8.0 Hz, 1H), 4.00-3.96 (m, 1H), 3.94-3.90 (m, 1H), 3.11-3.06 (m, 1H), 2.92-2.86 (m, 1H), 2.31 (s, 3H), 1.40 (s, 9H); ^{13}C NMR ($CDCl_3$, 125 MHz): 143.2, 141.6, 137.3, 134.5, 133.1, 129.5, 129.4, 129.2, 128.7, 127.6, 127.5, 125.9, 125.4, 111.9, 84.8, 60.7, 58.5, 29.1, 28.4, 21.6; LC-MS (ESI) m/z 333 $[M+H]^+$; HRMS (ESI) m/z calcd for $C_{22}H_{25}ON_2$ $[M+H]^+$ 333.1961, found 333.1957.

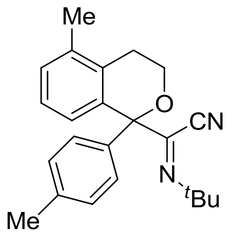

(*E*)-*N*-(*tert*-Butyl)-5-methyl-1-(*p*-tolyl)isochroman-1-carbimidoyl cyanide (4f):
Following the general procedure as for **4a**, the reaction mixture of **3f** (71.5 mg, 0.3 mmol), ^{t}BuNC (170 μL, 1.5 mmol), AgOTf (7.7 mg, 0.03 mmol), and DDQ (139.0 mg, 0.6 mmol) in dry PhCl (3.0 mL) was stirred at 100 ^{o}C for 22 h to afford product **4f** (81.1 mg, 78%) as white solid. M.p. 122-123 ^{o}C; IR (KBr, cm^{-1}): 2974, 2964, 2870, 2220, 1642, 1508, 1456, 1365, 1234, 1096, 1056, 918, 811, 773; ^{1}H NMR ($CDCl_3$, 500 MHz): δ 7.14 (d, J = 7.5 Hz, 1H), 7.09-7.06 (m, 5H), 6.87 (d, J = 8.0 Hz, 1H), 4.03-3.99 (m, 1H), 3.83-3.78 (m, 1H), 2.98-2.93 (m, 1H), 2.69-2.64 (m, 1H), 2.33 (s, 3H), 2.28 (s, 3H), 1.38 (s, 9H); ^{13}C NMR ($CDCl_3$, 125 MHz): 143.4, 138.8, 137.5, 136.6, 133.4, 132.6, 129.2, 129.0, 128.2, 126.8, 124.9, 112.0, 85.0, 60.1, 58.4, 29.0, 25.9, 21.1, 19.2; LC-MS (ESI) m/z 347 [M+H]$^+$; HRMS (ESI) m/z calcd for $C_{23}H_{27}ON_2$ [M+H]$^+$ 347.2118, found 347.2128.

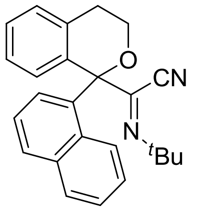

(*E*)-*N*-(*tert*-Butyl)-1-(naphthalen-1-yl)isochroman-1-carbimidoyl cyanide (4g):
Following the general procedure as for **4a**, the reaction mixture of **3g** (78.1 mg, 0.3 mmol), ^{t}BuNC (170 μL, 1.5 mmol), AgOTf (7.7 mg, 0.03 mmol), and DDQ (139.0 mg, 0.6 mmol) in dry PhCl (3.0 mL) was stirred at 100 ^{o}C for 23 h to afford product **4g** (76.5 mg, 69%) as white solid. M.p. 141-143 ^{o}C; IR (KBr, cm^{-1}): 2977, 2216, 1640, 1598, 1453, 1362, 1229, 1202, 1090, 1050, 910, 785, 745, 632; ^{1}H NMR ($CDCl_3$, 500 MHz): δ 8.17-8.15 (m, 1H), 7.84-7.82 (m, 1H), 7.78 (d, J = 8.0 Hz, 1H), 7.45-7.43 (m, 2H), 7.34-7.32 (m, 1H), 7.28 (d, J = 7.5 Hz, 1H), 7.24-7.21 (m, 2H), 7.11 (d, J = 7.5 Hz, 1H), 6.91 (d, J = 7.0 Hz, 1H), 4.08-4.04 (m, 1H), 3.73-3.68 (m, 1H), 3.34-3.28 (m, 1H), 2.72 (d, J = 16.0 Hz, 1H), 1.22 (s, 9H); ^{13}C NMR ($CDCl_3$, 125 MHz): 143.0, 137.7, 135.6, 134.6, 132.6, 131.0, 129.9, 129.8, 129.6, 129.2, 128.5 (2), 128.0, 125.6, 125.1, 125.0, 123.6, 111.9, 86.3, 60.2, 58.3, 28.7, 28.1; LC-MS (ESI) m/z 369 [M+H]$^+$; HRMS (ESI) m/z calcd for $C_{25}H_{25}ON_2$ [M+H]$^+$ 369.1961, found 369.1957.

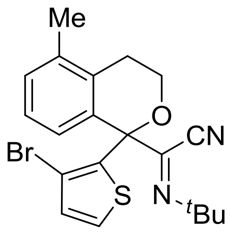

(*E*)-1-(3-Bromothiophen-2-yl)-*N*-(*tert*-butyl)-5-methylisochroman-1-carbimidoyl cyanide (4h):
Following the general procedure as for **4a**, the reaction mixture of **3h** (92.4 mg, 0.3 mmol),

^{t}BuNC (170 μL, 1.5 mmol), AgOTf (7.7 mg, 0.03 mmol), and DDQ (139.0 mg, 0.6 mmol) in dry PhCl (3.0 mL) was stirred at 100 °C for 24 h to afford product **4h** (72.3 mg, 58%) as white solid. M.p. 153-155 °C; IR (KBr, cm^{-1}): 3088, 2972, 2927, 1942, 1734, 1645, 1462, 1356, 1225, 1086, 1050, 868, 772, 740; ^{1}H NMR ($CDCl_3$, 500 MHz): δ 7.21-7.19 (m, 1H), 7.14-7.11 (m, 3H), 7.03 (d, J = 5.0 Hz, 1H), 4.20-4.16 (m, 1H), 3.85-3.79 (m, 1H), 3.08-3.01 (m, 1H), 2.63-2.59 (m, 1H), 2.28 (s, 3H), 1.41 (s, 9H); ^{13}C NMR ($CDCl_3$, 125 MHz): 140.3, 139.8, 137.0, 133.6, 132.4, 132.3, 130.1, 125.5, 125.4, 125.1, 111.5, 110.2, 82.3, 60.1, 58.5, 28.9, 25.5, 19.2; LC-MS (ESI) m/z (%): 419 (78) [M (^{81}Br)+H]$^+$, 417 (100) [M (^{79}Br)+H]$^+$; HRMS (ESI) m/z calcd for $C_{20}H_{22}ON_2BrS$ [M+H]$^+$ 417.0631, found 417.0630.

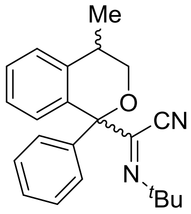

(*E*)-*N*-(*tert*-Butyl)-4-methyl-1-phenylisochroman-1-carbimidoyl cyanide (4i):
Following the general procedure as for **4a**, the reaction mixture of **3i** (44.5 mg, 0.3 mmol), ^{t}BuNC (170 μL, 1.5 mmol), AgOTf (7.7 mg, 0.03 mmol), and DDQ (139.0 mg, 0.6 mmol) in dry PhCl (3.0 mL) was stirred at 100 °C for 24 h to afford product **4i** (79.4 mg, 79%) as white solid. M.p. 113-114 °C; IR (KBr, cm^{-1}): 2972, 2207, 1640, 1483, 1450, 1365, 1230, 1211, 1109, 1045, 749, 699; ^{1}H NMR ($CDCl_3$, 500 MHz): δ 7.33-7.11 (m, 8H), 7.03 (d, J = 7.8 Hz, 0.23H), 6.97 (d, J = 7.8 Hz, 0.76H), 3.96 (dd, J = 11.5, 4.7 Hz, 0.78H), 3.80 (dd, J = 11.8, 3.8 Hz, 0.24H), 3.69 (dd, J = 11.5, 2.4 Hz, 0.23H), 3.64 (dd, J = 11.5, 6.5 Hz, 0.77H), 3.15-3.08 (m, 0.78H), 2.85-2.84 (m, 0.23H), 1.51 (d, J = 7.0 Hz, 0.79H), 1.39 (s, 6.96H), 1.37 (s, 2.13H), 1.34 (d, J = 7.0 Hz, 2.45H); ^{13}C NMR ($CDCl_3$, 125 MHz): 140.6, 139.6, 132.6, 131.5, 129.5, 129.4, 129.2, 128.5, 128.1, 128.0, 127.9, 127.7 (2), 127.4, 125.5, 125.2, 111.9, 85.5, 85.2, 66.7, 65.9, 58.6, 58.5, 32.4, 31.7, 29.0 (2), 21.8, 18.6; LC-MS (ESI) m/z 333 [M+H]$^+$; HRMS (ESI) m/z calcd for $C_{22}H_{25}ON_2$ [M+H]$^+$ 333.1961, found 333.1960.

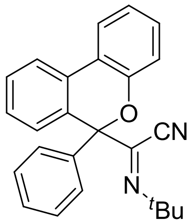

(*E*)-*N*-(*tert*-Butyl)-6-phenyl-6*H*-benzo[*c*]chromene-6-carbimidoyl cyanide (4j):
Following the general procedure as for **4a**, the reaction mixture of **3j** (77.5 mg, 0.3 mmol), ^{t}BuNC (170 μL, 1.5 mmol), AgOTf (7.7 mg, 0.03 mmol), and DDQ (139.0 mg, 0.6 mmol) in dry PhCl (3.0 mL) was stirred at 100 °C for 23 h to afford product **4j** (107.8 mg, 98%) as white solid. M.p. 142-144 °C; IR (KBr, cm^{-1}): 2969, 2224, 1646, 1593, 1484, 1440, 1231, 1019, 758, 694; ^{1}H NMR ($CDCl_3$, 500 MHz): δ 7.77 (d, J = 7.5 Hz, 1H), 7.71 (d, J = 7.5 Hz, 1H), 7.44-7.40 (m, 6H), 7.29-7.20 (m, 3H), 7.08 (t, J = 7.2 Hz, 1H), 6.84 (d, J = 7.5 Hz, 1H), 1.21 (s, 9H); ^{13}C NMR ($CDCl_3$, 125 MHz): 151.3, 140.7, 138.1, 133.1, 130.0, 129.6, 129.0, 128.6, 128.3, 128.0, 127.8, 127.3, 123.1, 122.9, 122.3, 119.0, 111.6, 86.8, 58.7, 28.8; LC-MS (ESI) m/z 367 [M+H]$^+$; HRMS (ESI) m/z calcd for $C_{25}H_{23}ON_2$ [M+H]$^+$ 367.1805, found 367.1804.

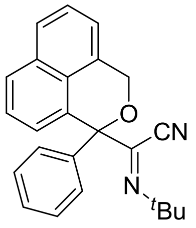

(*E*)-*N*-(*tert*-Butyl)-1-phenyl-1*H*,3*H*-benzo[*de*]isochromene-1-carbimidoyl cyanide (4k): Following the general procedure as for **4a**, the reaction mixture of **3k** (73.9 mg, 0.3 mmol), ^{t}BuNC (170 μL, 1.5 mmol), AgOTf (7.7 mg, 0.03 mmol), and DDQ (139.0 mg, 0.6 mmol) in dry PhCl (3.0 mL) was stirred at 100 °C for 23 h to afford product **4k** (78.4 mg, 74%) as white solid. M.p. 153-155 °C; IR (KBr, cm^{-1}): 2964, 2856, 2212, 1631, 1446, 1364, 1230, 1205, 1059, 822, 768, 690; ^{1}H NMR ($CDCl_3$, 500 MHz): δ 7.89 (d, *J* = 8.5 Hz, 1H), 7.81 (d, *J* = 8.5 Hz, 1H), 7.51 (t, *J* = 7.7 Hz, 1H), 7.46 (t, *J* = 7.7 Hz, 1H), 7.30-7.29 (m, 3H), 7.19 (t, *J* = 6.5 Hz, 2H), 7.16-7.15 (m, 2H), 5.11 (d, *J* = 15.0 Hz, 1H), 4.90 (d, *J* = 14.5 Hz, 1H), 1.41 (s, 9H); ^{13}C NMR ($CDCl_3$, 125 MHz): 142.5, 139.8, 133.1, 131.2, 130.4, 129.1, 128.2 (2), 127.9, 126.8, 126.7, 125.7, 125.1, 124.6, 120.6, 111.9, 85.8, 63.8, 58.7, 29.1; LC-MS (ESI) m/z 355 [M+H]$^{+}$; HRMS (ESI) m/z calcd for $C_{24}H_{23}ON_2$ [M+H]$^{+}$ 355.1805, found 355.1801.

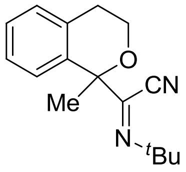

(*E*)-*N*-(*tert*-Butyl)-1-methylisochroman-1-carbimidoyl cyanide (4l): Following the general procedure as for **4a**, the reaction mixture of **3l** (44.5 mg, 0.3 mmol), ^{t}BuNC (170 μL, 1.5 mmol), AgOTf (7.7 mg, 0.03 mmol), and DDQ (139.0 mg, 0.6 mmol) in dry PhCl (3.0 mL) was stirred at 100 °C for 24 h to afford product **4l** (46.5 mg, 60%) as pale yellow oil. IR (KBr, cm^{-1}): 2975, 2212, 1645, 1460, 1368, 1232, 1109, 1031, 756; ^{1}H NMR ($CDCl_3$, 500 MHz): δ 7.23-7.14 (m, 3H), 6.99-6.97 (m, 1H), 4.17-4.13 (m, 1H), 4.03-3.98 (m, 1H), 3.15-3.09 (m, 1H), 2.75 (dt, *J* = 16.0, 3.5 Hz, 1H), 1.68 (s, 3H), 1.40 (s, 9H); ^{13}C NMR ($CDCl_3$, 125 MHz): 143.0, 135.6, 134.3, 129.1, 127.3, 126.5, 126.4, 111.7, 80.6, 60.8, 58.0, 29.1, 28.8, 25.3; LC-MS (ESI) m/z 257 [M+H]$^{+}$; HRMS (ESI) m/z calcd for $C_{16}H_{21}ON_2$ [M+H]$^{+}$ 257.1648, found 257.1645.

Synthesis and Characterization of 1,2,3,4-Tetrahydroisoquinolines, Related to Figure 4.

Compounds **5a** (Sullivan et al., 2014), **5k** (Pingaew et al., 2013), **5s** (Michael et al., 2010) and **5u** (Park et al., 2008) were prepared by known method. Othor tetrahydroisoquinolines were prepared as shown below.

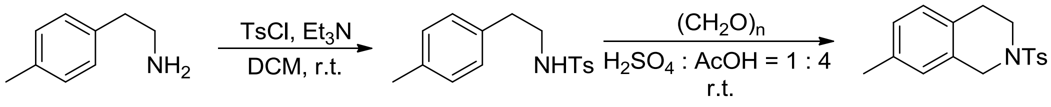

5b' **5b''** **5b**

7-Methyl-2-tosyl-1,2,3,4-tetrahydroisoquinoline (5b):

To a solution of **5b'** (135.2 mg, 1.0 mmol), Et_3N (0.28 mL, 2.0 mmol) and dichloromethane (3 mL) was added a solution of TsCl (228 mg, 1.2 mmol) in dichloromethane (3 mL). After stirred at room temperature for 6 h, the reaction was quenched with 2M HCl (15 mL) and extracted with dichloromethane (3 × 15 mL). The combined organic phase was washed with saturated Na_2CO_3 solution (15 mL) and brine (15 mL), dried over Na_2SO_4. The resulting solution was concentrated under reduced pressure. The residue was purified by flash column chromatography on silica gel (petroleum ether/ethyl acetate = 5 : 1) to give **5b"** (259.1 mg, 90%), which was directly used for the next step without further purification. To a mixture of **5b"** (259.1 mg, 0.9 mmol) and $(HCHO)_n$ (81 mg, 2.7 mmol) was added H_2SO_4/AcOH = 1 : 4 (5 mL). After stirred at room temperature for 12 h, the reaction was quenched with water (20 mL) and extracted with dichloromethane (3 × 15 mL). The combined organic phase was washed with saturated Na_2CO_3 solution (15 mL) and brine (15 mL), dried over Na_2SO_4. The resulting solution was concentrated under reduced pressure, and the residue was recrystallized to give pure product **5b** (225.0 mg, 83%) as a white solid. M.p. 164-165 ^{o}C; IR (KBr, cm^{-1}): 3022.4, 2863.4, 2829.4, 1935.1, 1588.2, 1502.1, 1455.0, 1338.9, 1161.9; ^{1}H NMR ($CDCl_3$, 500 MHz): δ 7.72 (d, J = 8.2 Hz, 2H), 7.32 (d, J = 8.0 Hz, 2H), 6.99-6.92 (m, 2 H), 6.84 (s, 1H), 4.21 (s, 2H), 3.33 (t, J = 5.9 Hz, 2H), 2.88 (t, J = 5.9 Hz, 2H), 2.42 (s, 3H), 2.27 (s, 3H); ^{13}C NMR ($CDCl_3$, 125 MHz): δ 143.73, 136.03, 133.44, 131.56, 130.11, 129.78, 128.75, 127.86, 127.70, 126.90, 47.62, 43.97, 28.60, 21.63, 21.08; EI-MS m/z (%): 146.1 (100), 301.1 (14) $[M]^+$; HRMS (EI) m/z calcd for $C_{17}H_{19}NO_2S$ $[M]^+$ 301.1136, found 301.1139.

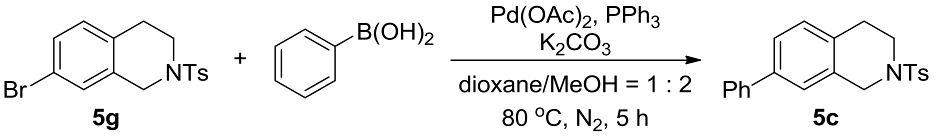

7-Phenyl-2-tosyl-1,2,3,4-tetrahydroisoquinoline (5c):

To a mixture of **5g** (219.8 mg, 0.6 mmol), phenylboronic acid (110 mg, 0.9 mmol), K_2CO_3 (231 mg, 1.8 mmol), $Pd(OAc)_2$ (6.7 mg, 0.03 mmol), PPh_3 (31.5 mg, 0.12 mmol) were added MeOH (1.2 mL) and dioxane (0.6 mL). After stirred at 80 ^{o}C for 5 h under N_2, the reaction mixture was filtered, and the filter residue was washed with ethyl acetate. The resulting solution was concentrated under reduced pressure. The residue was purified by flash column chromatography on silica gel (petroleum ether/ethyl acetate = 5 : 1) to give pure product **5c** (149.5 mg, 69%) as a white solid. M.p.118-119 ^{o}C; IR (KBr, cm^{-1}): 3051.5, 2927.7, 2847.0, 1962.2, 1902.0, 1594.6, 1487.1, 1454.7, 1339.4, 1157.0; ^{1}H NMR ($CDCl_3$, 500 MHz): δ 7.75 (d, J = 8.2 Hz, 2H), 7.52 (d, J = 7.6 Hz, 2H), 7.42 (t, J = 7.6 Hz, 2H), 7.38-7.30 (m, 4 H), 7.25 (s, 1H), 7.16 (d, J = 8.0 Hz, 1H), 4.31 (s, 2H), 3.39 (t, J = 5.9 Hz, 2H), 2.97 (t, J = 5.9 Hz, 2H), 2.42 (s, 3H); ^{13}C NMR ($CDCl_3$, 125 MHz): δ 143.85, 140.68, 139.59, 133.42, 132.34, 132.21, 129.86, 129.39, 128.93, 127.90, 127.47, 127.07, 125.73, 125.10, 47.83, 43.91, 28.76, 21.66; HRMS (EI) m/z calcd for $C_{22}H_{21}NO_2S$ $[M]^+$ 363.1293, found 363.1288.

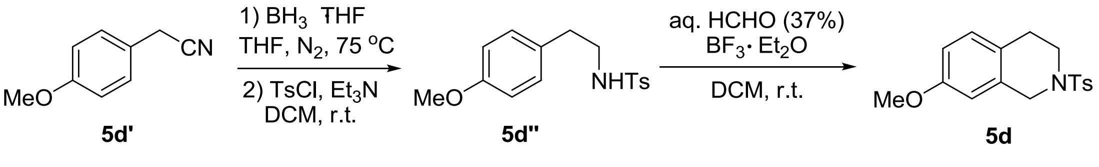

7-Methoxy-2-tosyl-1,2,3,4-tetrahydroisoquinoline (5d):

To a solution of **5d'** (736 mg, 5 mmol) and THF (1 mL) in three-necked bottle was added BH_3·THF (1M in THF, 15 mL) under N_2. After stirred at 75 °C overnight, the reaction was quenched with MeOH (5 mL) carefully, and concentrated under reduced pressure. After dichloromethane (8 mL), Et_3N (1.4 mL, 10 mmol) and pyridine (0.8 mL, 10 mmol) were added to this residue, a solution of TsCl (1.14 g, 6 mmol) in dichloromethane (8 mL) was added, and the mixture was stirred at room temperature overnight. The reaction was quenched with 2 M HCl (15 mL), extracted with dichloromethane (3 × 15 mL). The combined organic phase was washed with saturated Na_2CO_3 solution (15 mL) and brine (15 mL), dried over Na_2SO_4. The resulting solution was concentrated under reduced pressure. The residue was purified by flash column chromatography on silica gel (petroleum ether/ethyl acetate = 5 : 1) to give **5d''** (811.6 mg, 53% for two steps) without further purification. To a solution of **5d''** (305.4 mg, 1 mmol) in dichloromethane (2.4 mL) was added $BF_3·OEt_2$ (225 μL, 3 mmol) and aq. HCHO (37%) (93 μL). After stirred at room temperature for 2 h, the reaction was quenched with water (10 mL), and extracted with dichloromethane (3 × 10 mL). The combined organic phase washed with brine (15 mL), and dried over Na_2SO_4. The resulting solution was concentrated under reduced pressure. And the residue was purified by flash column chromatography on silica gel (petroleum ether/ethyl acetate = 5 : 1) to give product **5d** (114.8 mg, 36%) as a white solid. M.p. 115-117 °C; IR (KBr, cm^{-1}): 2925.4, 2849.8, 1609.2, 1503.6, 1457.5, 1337.4; ^{1}H NMR ($CDCl_3$, 500 MHz): δ 7.72 (d, J = 8.3 Hz, 2H), 7.32 (d, J = 8.2 Hz, 2H), 6.98 (d, J = 8.4 Hz, 1H), 6.71 (dd, J = 8.5, 2.6 Hz, 1H), 6.55 (d, J = 2.6 Hz, 1H), 4.21 (s, 2H), 3.75 (3H, s), 3.33 (t, J = 5.9 Hz, 2H), 2.85 (t. J = 5.9, 2H), 2.42 (s, 3H); ^{13}C NMR ($CDCl_3$, 125 MHz): δ 158.16, 143.78, 133.49, 132.78, 129.89, 129.83, 127.87, 125.28, 113.35, 111.05, 55.44, 47.82, 44.13, 28.19, 21.66; EI-MS m/z (%): 134.1 (100), 317.1 (32) [M]$^+$; HRMS (EI) m/z calcd for $C_{17}H_{19}NO_3S$ [M]$^+$ 317.1086, found 317.1087.

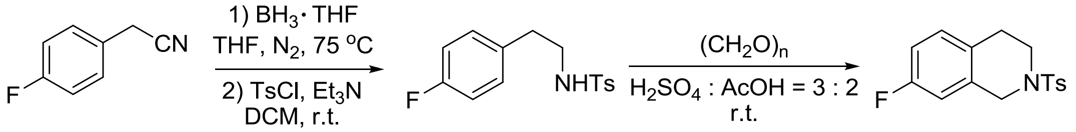

5e' **5e''** **5e**

7-Fluoro-2-tosyl-1,2,3,4-tetrahydroisoquinoline (5e):

To a solution of **5e'** (675 mg, 5 mmol) and THF (1 mL) in a three-necked bottle was added BH_3·THF (1M in THF, 15 mL) under N_2. After stirred at 75 °C overnight, the reaction was quenched with MeOH (5 mL) carefully, and concentrated under reduced pressure. After dichloromethane (8 mL), Et_3N (1.4 mL, 10 mmol) and pyridine (0.8 mL, 10 mmol) were added to this residue, a solution of TsCl (1.14 g, 6 mmol) in dichloromethane (8 mL) was dropped, and stirred at room temperature overnight. The reaction was quenched with 2 M HCl (15 mL), extracted with dichloromethane (3 × 15 mL). The combined organic phase was washed with saturated Na_2CO_3 solution (15 mL), brine (15 mL), and dried over Na_2SO_4. The resulting solution was concentrated under reduced pressure. The residue was purified by flash column chromatography on silica gel (petroleum ether/ethyl acetate = 5 : 1) to give **5e''** (670.9 mg, 46% for two steps). To a mixture of **5e''** (293.4 mg, 1 mmol) and $(HCHO)_n$ (90 mg, 3 mmol) was added H_2SO_4/AcOH = 3 : 2 (10 mL). After stirred at room temperature for 5 h, the reaction was quenched with water (20 mL), and extracted with dichloromethane (3 × 10 mL). The combined

organic phase was washed with saturated Na_2CO_3 solution (15 mL) and brine (15 mL), and then dried over Na_2SO_4. The resulting solution was concentrated under reduced pressure. The residue was recrystallized (dichloromethane/hexane) to give pure product **5e** (225.0 mg, 83%) as a white solid. M.p. 116-117 ^oC; IR (KBr, cm^{-1}): 2975.2, 2908.6, 1921.2, 1731.4, 1605.3, 1499.1, 1437.1; ^1H NMR ($CDCl_3$, 500 MHz): δ 7.72 (d, J = 8.3 Hz, 2H), 7.33 (d, J = 8.2 Hz, 2H), 7.03 (dd, J = 8.5, 5.7 Hz, 1H), 6.84 (td, J = 8.5, 2.6 Hz, 1H), 6.71 (dd, J = 9.2, 2.5 Hz, 1H), 4.21 (s, 2H), 3.34 (t, J = 5.9 Hz, 2H), 2.88 (t, J = 5.9 Hz, 2H), 2.42 (s, 3H); ^{19}F NMR ($CDCl_3$, 470 MHz): δ = -116.25 (m, Ar-F); ^{13}C NMR ($CDCl_3$, 125 MHz): δ 161.31 (d, $^1J_{C-F}$ = 244.7 Hz), 143.96, 133.58 (d, $^3J_{C-F}$ = 7.6 Hz), 133.34, 130.43 (d, $^3J_{C-F}$ = 7.8 Hz), 129.90, 128.82 (d, $^4J_{C-F}$ = 2.8 Hz), 127.86, 114.15 (d, $^2J_{C-F}$ = 21.2 Hz), 112.95 (d, $^2J_{C-F}$ = 22.0 Hz), 47.58 (d, $^4J_{C-F}$ = 2.3 Hz), 43.87, 28.33, 21.66; EI-MS m/z (%): 150.1 (100), 305.1 (16) $[M]^+$; HRMS (EI) m/z calcd for $C_{16}H_{16}FNO_2S$ $[M]^+$ 305.0886, found 305.0890.

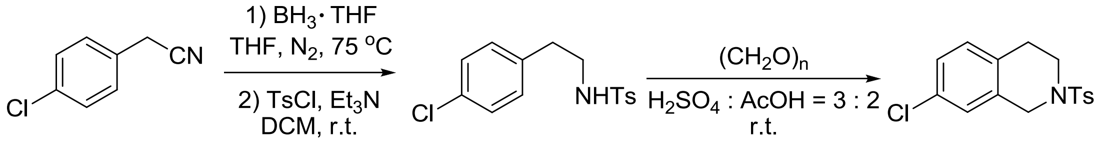

5f' **5f''** **5f**

7-Chloro-2-tosyl-1,2,3,4-tetrahydroisoquinoline (5f):

To a solution of **5f'** (758 mg, 5 mmol) and THF (1 mL) in a three-necked bottle was added BH_3·THF (1 M in THF, 15 mL) under N_2. After stirred at 75 ^oC overnight, the reaction was quenched with MeOH (5 mL) carefully, and concentrated under reduced pressure. After dichloromethane (8 mL), Et_3N (1.4 mL, 10 mmol) and pyridine (0.8 mL, 10 mmol) were added in this residue, asolution of TsCl (1.14 g, 6 mmol) in dichloromethane (8 mL) was added, and the mixture was stirred at room temperature overnight. The reaction was quenched with 2 M HCl (15 mL), extracted with dichloromethane (3 × 15 mL). The combined organic phase was washed with saturated Na_2CO_3 solution (15 mL), brine (15 mL), and dried over Na_2SO_4. The resulting solution was concentrated under reduced pressure. The residue was purified by flash column chromatography on silica gel (petroleum ether/ethyl acetate = 5 : 1) to give **5f''** (850.4 mg, 55% for two steps). To a mixture of **5f''** (309.8 mg, 1 mmol) and $(HCHO)_n$ (90 mg, 3 mmol) was added H_2SO_4/AcOH = 3 : 2 (10 mL). After stirred at room temperature for 5 h, the reaction was quenched with water (20 mL), and extracted with dichloromethane (3 × 10 mL). The combined organic phase was washed with saturated Na_2CO_3 solution (15 mL), brine (15 mL), and dried over Na_2SO_4. The resulting solution was concentrated under reduced pressure. The residue was recrystallized (dichloromethane/Hexane) to give pure product (266.6 mg, 83%) as a white solid. M.p. 156-158 ^oC; IR (KBr, cm^{-1}): 3032.1, 2930.9, 2843.7, 1925.6, 1741.2, 1598.4, 1483.4, 1419.8, 1336.9, 1161.4; ^1H NMR ($CDCl_3$, 500 MHz): δ 7.70 (d, J = 8.0 Hz, 2H), 7.32 (d, J = 7.9 Hz, 2H), 7.09 (d, J = 8.2 Hz, 1H), 7.05-6.95 (m, 2H), 4.18 (s, 2H), 3.32 (t, J = 5.8 Hz, 2H), 2.87 (t, J = 5.6 Hz, 2H), 2.41 (s, 3H); ^{13}C NMR ($CDCl_3$, 125 MHz): δ 143.95, 133.48, 133.20, 131.97, 131.66, 130.23, 129.86, 127.78, 127.03, 126.30, 47.32, 43.64, 28.39, 21.60; EI-MS m/z (%): 166.0 (100) $[M–Ts]^+$; HRMS (EI) m/z calcd for $C_{16}H_{16}ClNO_2S$ $[M]^+$ 321.0590, found 321.0601.

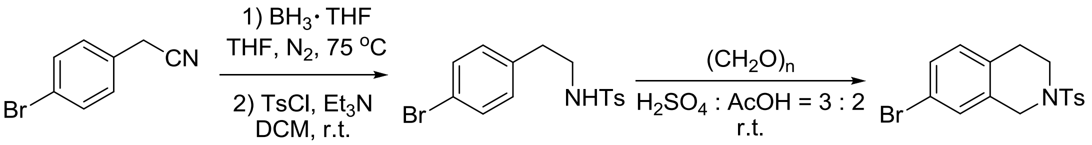

5g' **5g"** **5g**

7-Bromo-2-tosyl-1,2,3,4-tetrahydroisoquinoline (5g):

To a solution of **5g'** (980 mg, 5 mmol) and THF (1 mL) in a three-necked bottle was added BH_3·THF (1 M in THF, 15 mL) under N_2. After stirred at 75 ^oC overnight, the reaction was quenched with MeOH (5 mL) carefully, and concentrated under reduced pressure. After dichloromethane (8 mL) and Et_3N (1.4 mL, 10 mmol) were added to this residue, a solution of TsCl (1.14 g, 6 mmol) in dichloromethane (8 mL) was added, and the mixture was stirred at room temperature overnight. The reaction was quenched with 2 M HCl (15 mL), extracted with dichloromethane (3 × 15 mL). The combined organic phase was washed with saturated Na_2CO_3 solution (15 mL), brine (15 mL), and dried over Na_2SO_4. The resulting solution was concentrated under reduced pressure. The residue was purified by flash column chromatography on silica gel (petroleum ether/ethyl acetate = 5 : 1) to give **5g"** (884.2 mg, 50% for two steps). To a mixture of **5g"** (884.2 mg, 2.5 mmol) and $(HCHO)_n$ (225 mg, 7.5 mmol) was added H_2SO_4/AcOH = 3 : 2 (25 mL). After stirred at room temperature for 5 h, the reaction was quenched with water (40 mL). The mixture was filtered to collect residue solid. The residue was recrystallized (hexane/ethyl acetate) to give product **5g** (737.3 mg, 81%) as a white solid. M.p. 160-161 ^oC; IR (KBr, cm^{-1}): 3031.1, 2929.8, 2841.9, 1924.6, 1740.8, 1593.9, 1479.8, 1417.5 1337.9, 1161.3; ^1H NMR ($CDCl_3$, 500 MHz): δ 7.71 (d, J = 8.2 Hz, 2H), 7.32 (d, J = 8.1 Hz, 2H), 7.25 (d, J = 8.6 Hz, 1H), 7.18 (s, 1H), 6.95 (d, J = 8.2 Hz, 1H), 4.20 (s, 2H), 3.33 (t, J = 5.9 Hz, 2H), 2.86 (t, J = 5.8 Hz, 2H), 2.42 (s, 3H); ^{13}C NMR ($CDCl_3$, 125 MHz): δ 143.99, 133.95, 133.30, 132.24, 130.57, 130.00, 129.91, 129.32, 127.85, 119.96, 47.23, 43.62, 28.52, 21.66; EI-MS m/z (%): 210.0 (100), 364 (8) [M (^{79}Br)]$^+$, 366 (10) [M (^{81}Br)]$^+$; HRMS (EI) m/z calcd for $C_{16}H_{16}BrNO_2S$ $[M]^+$ 365.0085, found 365.0084.

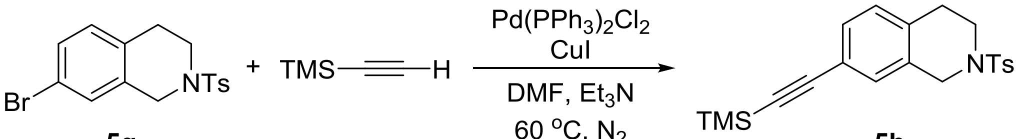

5g **5h**

2-Tosyl-7-((trimethylsilyl)ethynyl)-1,2,3,4-tetrahydroisoquinoline (5h):

To a mixture of **1g** (219.8 mg, 0.6 mmol), $Pd(PPh_3)_2Cl_2$ (42.1 mg, 0.06 mmol) and CuI (11.4 mg, 0.06 mmol) in DMF (3 mL) were added ethynyltrimethylsilane (169 μL, 1.2 mmol) and Et_3N (169 μL, 1.8 mmol). After stirred at 50 ^oC for 4 h, the reaction was quenched with saturated Na_2CO_3 solution, and extracted with EtOAc (10 mL) for three times. The combined organic phase were washed with water (3 × 10 mL), brine (10 mL), and dried over Na_2SO_4, The resulting solution was concentrated under reduced pressure. The residue was purified by flash column chromatography on silica gel (petroleum ether/ethyl acetate = 10 : 1) to give product **5h** (138.2 mg, 60%) as a white solid. M.p. 39-41 ^oC; IR (KBr, cm^{-1}): 3046.1, 2977.9, 2925.7, 2850.0, 1919.6, 1608.7, 1457.7, 1361.9, 1200.5, 1159.6; ^1H NMR ($CDCl_3$, 500 MHz): δ 7.70 (d, J = 8.0 Hz, 2H), 7.32 (d, J = 8.0 Hz, 2H), 7.22 (d, J = 7.8 Hz, 1H), 7.15 (s, 1H), 7.00 (d, J = 7.9 Hz, 1H), 4.19 (s, 2H), 3.34, (t, J = 5.7 Hz, 2H), 2.89 (t, J = 5.7 Hz, 2H), 2.41 (s, 3H), 0.23 (s, 9H); ^{13}C NMR ($CDCl_3$, 125 MHz): δ 143.91, 133.85, 133.37, 131.87, 130.31, 130.00, 129.87,

128.86, 127.85, 121.29, 104.66, 94.24, 47.37, 43.62, 28.90, 21.65, 0.08; EI-MS m/z (%): 228.1 (100), 383.1 (45) $[M]^+$; HRMS (EI) m/z calcd for $C_{21}H_{25}NO_2SSi$ $[M]^+$ 383.1375, found 383.1373.

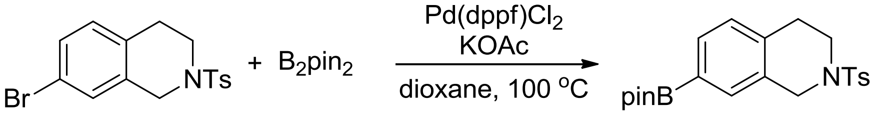

5g **5i**

7-Pinacolboryl-2-tosyl-1,2,3,4-tetrahydroisoquinoline (5i):

To a seal tube was added **1g** (256.4 mg, 0.7 mmol), $Pd(dppf)Cl_2$ (31 mg, 0.042 mmol), B_2pin_2 (200 mg, 0.78 mmol), KOAc (206 mg, 2.1 mmol) and dioxane (3 mL). After stirred at 100 °C for 2 h, the reaction was filtered by celite and washed with EtOAc. The solvent was removed under vacuum, and the residue was purified by flash column chromatography on silica gel to give pure product **5i** (212.4 mg, 73%) as a white solid. M.p. 133-134 °C; IR (KBr, cm^{-1}): 3046.1, 2977.9, 2925.7, 2850.0, 1919.6, 1608.7, 1457.7, 1361.9, 1200.5, 1159.6; ^1H NMR ($CDCl_3$, 500 MHz): δ 7.70 (d, J = 8.2 Hz, 2H), 7.56 (d, J = 7.6 Hz, 1H), 7.49 (s, 1H), 7.31 (d, J = 8.1 Hz, 2H), 7.08 (d, J = 7.6 Hz, 1H), 4.24 (s, 2H), 3.34 (t, J = 5.9 Hz, 2H), 2.93 (t, J = 5.7 Hz, 2H), 2.41 (s, 3H), 1.32 (s, 12H); ^{13}C NMR ($CDCl_3$, 125 MHz): δ 143.78, 136.54, 133.34, 133.05, 132.97, 131.20, 129.78, 128.35, 127.87, 83.96, 47.56, 43.64, 29.24, 24.96, 21.63; EI-MS m/z (%): 258.2 (100), 411.2 (4) $[M–H]^+$, 412.2 (14) $[M]^+$; HRMS (EI) m/z calcd for $C_{22}H_{27}BNO_4S$ $[M–H]^+$ 411.1790, found 411.1786.

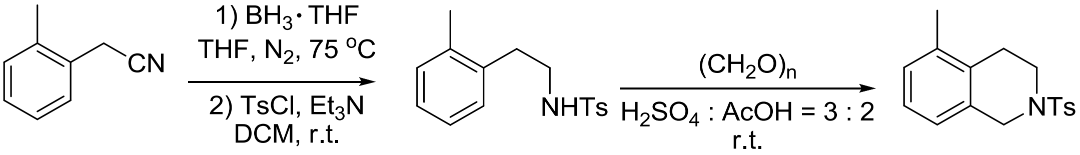

5j' **5j"** **5j**

5-Methyl-2-tosyl-1,2,3,4-tetrahydroisoquinoline (5j):

To a solution of **5j'** (656 mg, 5 mmol) and THF (1 mL) in a three-necked bottle was added BH_3·THF (1 M in THF, 15 mL) under N_2. After stirred at 75 °C overnight, the reaction was quenched with MeOH (5 mL) carefully, and concentrated under reduced pressure. To the residue were added dichloromethane (8 mL), Et_3N (1.4 mL, 10 mmol) and pyridine (0.8 mL, 10 mmol), and a solution of TsCl (1.14 g, 6 mmol) in dichloromethane (8 mL) was added dropwise. After stirred at room temperature overnight, the reaction was quenched with 2M HCl (15 mL), extracted with dichloromethane (3 x 15 mL). The combined organic phase was washed with saturated Na_2CO_3 solution (15 mL), brine (15 mL), and dried over Na_2SO_4. The resulting solution was concentrated under reduced pressure. The residue was purified by flash column chromatography on silica gel (petroleum ether/ethyl acetate = 5 : 1) to give **5j"** (714.0 mg, 49% for two steps). To a mixture of **5j"** (289.4 mg, 1 mmol) and $(HCHO)_n$ (90 mg, 3 mmol) was added H_2SO_4/AcOH = 1 : 4 (10 mL). After stirred at room temperature for 5 h, the reaction was quenched with water (40 mL). The mixture was filtered to collect the solid. The residue was recrystallized (hexane/ethyl acetate) to give product **5j** (273.4 mg, 91%) as a white solid. M.p. 160-162 °C; IR (KBr, cm^{-1}): 3029.4, 2925.7, 1922.7, 17.6.6, 1659.4, 1493.5, 1465.3, 1335.4, 1160.5; ^1H NMR ($CDCl_3$, 500 MHz): δ 7.73 (d, J = 8.3 Hz, 2H), 7.33 (d, J = 8.2 Hz, 2H), 7.06 (t, J = 7.5 Hz, 1H), 7.01 (d, J = 7.2 Hz, 1H), 6.88 (d, J = 7.6 Hz, 1H), 4.22 (s, 2H), 3.36 (t, J = 6.0 Hz, 2H), 2.79 (t, J = 6.0 Hz, 2H), 2.42 (s, 3H), 2.19 (s, 3H); ^{13}C NMR ($CDCl_3$, 125 MHz): δ

143.78, 136.57, 133.27, 131.74, 131.71, 129.78, 128.23, 127.94, 126.15, 124.20, 48.11, 43.94, 26.58, 21.64, 19.28; EI-MS m/z (%): 146.1 (100), 301.1 (10) $[M]^+$; HRMS (EI) m/z calcd for $C_{17}H_{19}NO_2S$ $[M]^+$ 301.1136, found 301.1129.

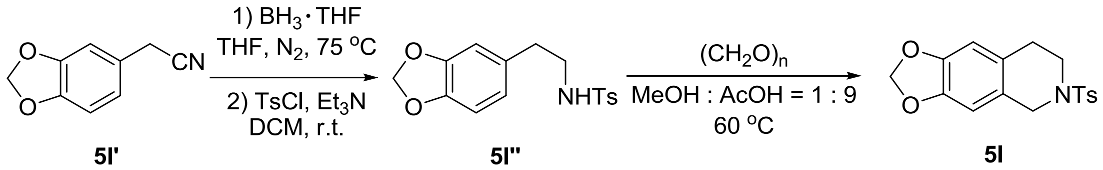

6-Tosyl-5,6,7,8-tetrahydro-[1,3]dioxolo[4,5-g]isoquinoline(5l):

To a solution of **5l'** (806 mg, 5 mmol) and THF (1 mL) in three-necked bottle was added BH_3·THF (1 M in THF, 15 mL) under N_2. After stirred at 75 °C overnight, the reaction was quenched with MeOH (5 mL) carefully, and concentrated under reduced pressure. After dichloromethane (8 mL) and Et_3N (1.4 mL, 10 mmol) were added in this residue, a solution of TsCl (1.14 g, 6 mmol) in dichloromethane (8 mL) was added, and the mixture was stirred at room temperature overnight. The reaction was quenched with 2 M HCl (15 mL), extracted with dichloromethane (3 × 15 mL). The combined organic phase was washed with saturated Na_2CO_3 solution (15 mL), brine (15 mL), and dried over Na_2SO_4. The resulting solution was concentrated under reduced pressure. The residue was purified by flash column chromatography on silica gel (petroleum ether/ethyl acetate = 3 : 1) to give **5l''** (850.4 mg, 55% for two steps). To a mixture of **5l''** (302.1 mg, 0.94 mmol) and $(HCHO)_n$ (84.6 mg, 2.82 mmol) was added MeOH (1 mL) and AcOH (9 mL). After stirred at 60 °C for 12 h, the reaction was quenched with water (20 mL), and extracted with dichloromethane (3 × 10 mL). The combined organic phase was washed with saturated Na_2CO_3 solution (15 mL), brine (15 mL), and dried over Na_2SO_4. The resulting solution was concentrated under reduced pressure. The residue was recrystallized (dichloromethane/hexane) to give product **5l** (304.8 mg, 92%) as a white solid. M.p. 150-151 °C; IR (KBr, cm^{-1}): 3046.4, 2893.3, 2837.2, 1917.7, 1710.3, 1594.4, 1495.5, 1391.1, 1344.3, 1160.6; ^1H NMR ($CDCl_3$, 500 MHz): δ 7.70 (d, J = 8.3 Hz, 2H), 7.31 (d, J = 8.2 Hz, 2H), 6.51 (s, 1H), 6.46 (s, 1H), 5.88 (s, 2H), 4.12 (s, 2H), 3.30 (t, J = 5.9 Hz, 2H), 2.80 (t, J = 5.9 Hz, 2H), 2.41 (s, 3H); ^{13}C NMR ($CDCl_3$, 125 MHz): δ 146.59, 146.36, 143.77, 133.39, 129.79, 127.81, 126.25, 124.55, 108.52, 106.20, 100.99, 47.64, 43.79, 28.91, 21.61; EI-MS m/z (%): 175.1 (100), 331.1 (14) $[M]^+$; HRMS (EI) m/z calcd for $C_{17}H_{17}NO_4S$ $[M]^+$ 331.0878, found 331.0877.

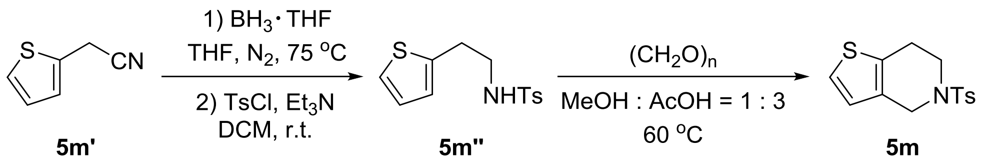

5-Tosyl-4,5,6,7-tetrahydrothieno[3,2-c]pyridine (5m):

To a solution of **5m'** (616 mg, 5 mmol) and THF (1 mL) in three-necked bottle was added BH_3·THF (1 M in THF, 15 mL) under N_2. After stirred at 65 °C overnight, the reaction was quenched with MeOH (5 mL) carefully, and concentrated under reduced pressure. After dichloromethane (10 mL) and Et_3N (1.4 mL, 10 mmol) were added to this residue, a solution of TsCl (950 mg, 5 mmol) in dichloromethane (10 mL) was added dropwise, and stirred at room temperature for 6 h. The reaction was quenched with 2 M HCl (15 mL), extracted with

dichloromethane (3 × 15 mL). The combined organic phase was washed with saturated Na_2CO_3 solution (15 mL), brine (15 mL), and dried over Na_2SO_4. The resulting solution was concentrated under reduced pressure. The residue was purified by flash column chromatography on silica gel (petroleum ether/ethyl acetate = 3 : 1) to give **5m”** (874.6 mg, 62% for two steps). To a mixture of **5m”** (517.1 mg, 1.8 mmol) and $(HCHO)_n$ (165.4 mg, 5.4 mmol) were added MeOH (4.5 mL) and AcOH (13.5 mL). After stirred at 60 ^oC for 12 h, the reaction was quenched with water (20 mL), and extracted with dichloromethane (3 × 10 mL). The combined organic phase was washed with saturated Na_2CO_3 solution (15 mL) and brine (15 mL), and then dried over Na_2SO_4. The resulting solution was concentrated under reduced pressure. The residue was purified by flash column chromatography on silica gel (petroleum ether/ethyl acetate = 5 : 1) to give product **5m** (116.6 mg, 22%) as a white solid. M.p. 159-160 ^oC; IR (KBr, cm^{-1}): 3083.5, 3026.2, 2918.2, 2859.4, 1920.8, 1593.9, 1455.6, 1344.6; ^1H NMR ($CDCl_3$, 500 MHz): δ 7.71 (d, J = 8.3 Hz, 2H), 7.31 (d, J = 8.1 Hz, 2H), 7.09 (d, J = 5.3 Hz, 1H), 6.71 (d, J = 5.1 Hz, 1H), 4.19 (s, 2H), 3.41 (t, J = 5.7 Hz, 2H), 2.90 (t, J = 5.7 Hz, 2H), 2.42 (s, 3H); ^{13}C NMR ($CDCl_3$, 125 MHz): δ 143.80, 133.77, 132.65, 130.69, 129.81, 127.70, 124.79, 123.70, 45.94, 43.99, 25.29, 21.62; EI-MS m/z (%): 110.0 (100), 293.1 (7) $[M]^+$; HRMS (EI) m/z calcd for $C_{14}H_{15}NO_2S_2$ $[M]^+$ 293.0544, found 293.0547.

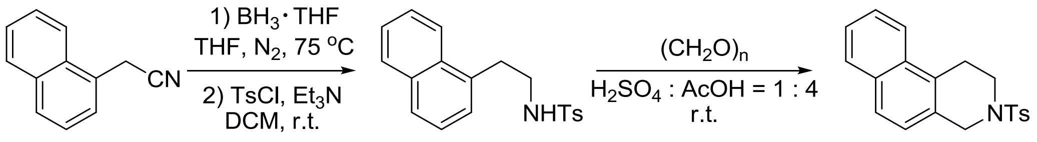

5n' **5n''** **5n**

3-Tosyl-1,2,3,4-tetrahydrobenzo[*f*]isoquinoline (5n):

To a solution of **5n’** (836 mg, 5 mmol) and THF (1 mL) in a three-necked bottle was added BH_3·THF (1 M in THF, 15 mL) under N_2. After stirred at 75 ^oC overnight, the reaction was quenched with MeOH (5 mL) carefully, and concentrated under reduced pressure. After dichloromethane (8 mL), Et_3N (1.4 mL, 10 mmol) was added in this residue, the solution of TsCl (1.14 g, 6 mmol) in dichloromethane (8 mL) was added, and stirred at room temperature for 10 h. The reaction was quenched with 2 M HCl (15 mL), extracted with dichloromethane (3 × 15 mL). The combined organic phase was washed with saturated Na_2CO_3 solution (15 mL), brine (15 mL), and dried over Na_2SO_4. The resulting solution was concentrated under reduced pressure. The residue was purified by flash column chromatography on silica gel (petroleum ether/ethyl acetate = 5 : 1) to give **5n”** (787.2 mg, 48% for two steps). To a mixture of **5n”** (684.1 mg, 2.1 mmol) and $(HCHO)_n$ (189 mg, 6.3 mmol) was added H_2SO_4/AcOH = 1 : 4 (10 mL). After stirred at room temperature for 5 h, the reaction was quenched with water (40 mL), and extracted with dichloromethane (3 × 15 mL). The combined organic phase was washed with saturated Na_2CO_3 solution (20 mL), brine (20 mL), and dried over Na_2SO_4. The resulting solution was concentrated under reduced pressure. The residue was purified by flash column chromatography on silica gel (petroleum ether/ethyl acetate = 5 : 1) to give product **5n** (383.1 mg, 54%) as a white solid. M.p. 239-241 ^oC; IR (KBr, cm^{-1}): 3058.0, 2970.7, 2923.4, 2857.3, 2822.8, 1925.1, 1591.1, 1500.5, 1340.4, 1158.7; ^1H NMR ($CDCl_3$, 500 MHz): δ 7.86 (d, J = 8.3 Hz, 1H), 7.83-7.72 (m, 3H), 7.66 (d, J = 8.4 Hz, 1H), 7.52 (t, J = 7.2 Hz, 1H), 7.49 (t, J = 7.2 Hz, 1H), 7.34 (d, J = 8.0 Hz, 2H), 7.12 (d, J = 8.5 Hz, 1H), 4.36 (s, 2H), 3.49 (t, J = 5.9 Hz, 2H), 3.27 (t, J = 5.5 Hz, 2H), 2.42 (s, 3H); ^{13}C NMR ($CDCl_3$, 125 MHz): δ 143.90, 133.27, 132.43,

131.89, 129.88, 129.06, 128.69, 128.44, 127.98, 127.00, 126.63, 125.76, 124.59, 122.81, 48.22, 43.68, 25.77, 21.66; EI-MS m/z: 337.1 $[M]^+$; HRMS (EI) m/z calcd for $C_{20}H_{19}NO_2S$ $[M]^+$ 337.1136, found 337.1133.

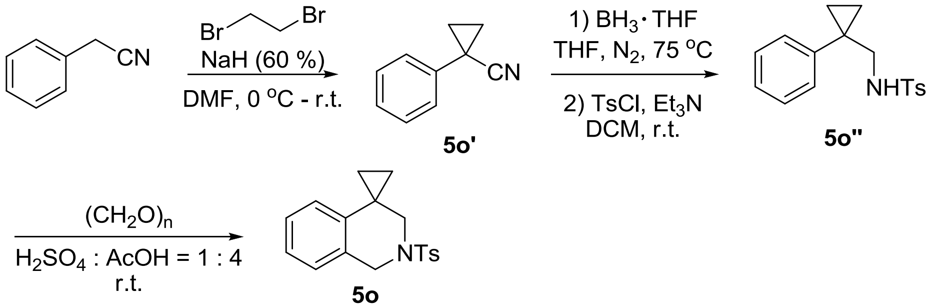

2'-Tosyl-2',3'-dihydro-1'H-spiro[cyclopropane-1,4'-isoquinoline] (5o):

To a solution of NaH (60%) (640 mg, 16 mmol) and DMF (5.6 mL) was added dropwisely a solution of 2-phenylacetonitrile (937.1 mg, 8 mmol) in DMF (9.4 mL) at 0 °C under N_2 atmosphere. After stirred for 40 min, 1,2-dibromoethane (1.8 g, 9.6 mmol) was added dropwise, and kept stirred for 5 h. The reaction was quenched with water, and extracted with ethyl acetate (3 × 15 mL). The combined organic phase was washed with brine (15 mL), and dried over Na_2SO_4. The resulting solution was concentrated under reduced pressure. The residue was purified by flash column chromatography on silica gel (petroleum ether/ethyl acetate = 50 : 1) to give **5o'** (541.8 mg, 47%) as a colorless liquid. To a solution of **5o'** (716 mg, 5 mmol) and THF (1 mL) in three-necked bottle was added BH_3·THF(1 M in THF, 15 mL) under N_2. After stirred at 75 °C overnight, the reaction was quenched with MeOH (5 mL) carefully, and concentrated under reduced pressure. After dichloromethane (8 mL), Et_3N (1.4 mL, 10 mmol) was added in this residue, a solution of TsCl (1.14 g, 6 mmol) in dichloromethane (8 mL) was added dropwise, and stirred at room temperature for 5 h. The reaction was quenched with 2 M HCl (15 mL), extracted with dichloromethane (3 × 15 mL). The combined organic phase was washed with saturated Na_2CO_3 solution (15 mL), brine (15 mL), and dried over Na_2SO_4. The resulting solution was concentrated under reduced pressure. The residue was purified by flash column chromatography on silica gel (petroleum ether/ethyl acetate = 5 : 1) to give **5o''** (903.5 mg, 60% for two steps). To a mixture of **5o''** (304.1 mg, 1 mmol) and $(HCHO)_n$ (90 mg, 3 mmol) was added H_2SO_4/AcOH = 1 : 4 (10 mL). After stirred at room temperature for 4 h, the reaction was quenched with water (40 mL). The mixture was filtered to collect the solid. The residue was recrystallized (hexane/ethyl acetate) to give product **5o** (260.1 mg, 83%) as a white solid. M.p. 155-157 °C; IR (KBr, cm^{-1}): 3074.1, 2995.3, 2921.2, 2839.6, 1933.6, 1598.0, 1491.4, 1452.7, 1338.1; ^1H NMR ($CDCl_3$, 500 MHz): δ 7.70 (d, J = 8.1 Hz, 2H), 7.31 (d, J = 8.1 Hz, 2H), 7.17-7.05 (m, 2H), 7.02 (d, J = 7.3 Hz, 1H), 6.67 (d, J = 7.4 Hz, 1H), 4.35 (s, 2H), 3.14 (s, 2H), 2.42 (s, 3H), 1.05-0.91 (m, 4H); ^{13}C NMR ($CDCl_3$, 125 MHz): δ 143.74, 138.35, 133.61, 132.02, 129.77, 127.91, 127.39, 126.18, 125.66, 121.71, 53.08, 48.83, 21.65, 19.54, 16.88; EI-MS m/z (%): 130.1 (100), 313.1 (9) $[M]^+$; HRMS (EI) m/z calcd for $C_{18}H_{19}NO_2S$ $[M]^+$ 313.1136, found 313.1135.

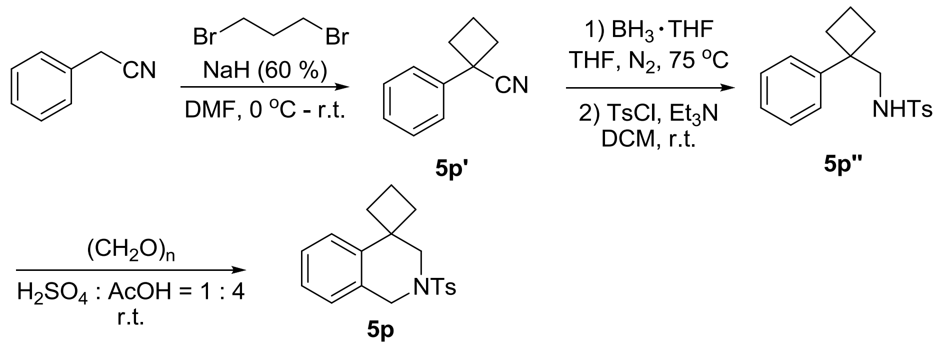

2'-Tosyl-2',3'-dihydro-1'H-spiro[cyclobutane-1,4'-isoquinoline] (5p):

To a solution of NaH (60%) (960 mg, 24 mmol) and DMF (7 mL) was added a solution of 2-phenylacetonitrile (1.17 g, 10 mmol) in DMF (11 mL) dropwise at 0 °C under N_2 atmosphere. After stirred for 40 min, 1,3-dibromopropane (2.42 g, 12 mmol) was added dropwise, and kept stirred for 5 h. The reaction was quenched with water, extracted with ethyl acetate (3 × 15 mL). The combined organic phase was washed with brine (15 mL), and dried over Na_2SO_4. The resulting solution was concentrated under reduced pressure. The residue was purified by flash column chromatography on silica gel (petroleum ether/ethyl acetate = 50 : 1) to give **5p'** (1.02 g, 65%) as colorless liquid. To a solution of **5p'** (786 mg, 5 mmol) and THF (1 mL) in three-necked bottle was added BH_3·THF (1 M in THF, 15 mL) under N_2. After stirred at 75 °C overnight, the reaction was quenched with MeOH (5 mL) carefully, and concentrated under reduced pressure. After dichloromethane (8 mL) and Et_3N (1.4 mL, 10 mmol) were added in to the residue, a solution of TsCl (1.14 g, 6 mmol) in dichloromethane (8 mL) was added dropwise, and stirred at room temperature for 5 h. The reaction was quenched with 2 M HCl (15 mL), extracted with dichloromethane (3 × 5 mL). The combined organic phase was washed with saturated Na_2CO_3 solution (15 mL) and brine (15 mL), and dried over Na_2SO_4. The resulting solution was concentrated under reduced pressure. The residue was purified by flash column chromatography on silica gel (petroleum ether/ethyl acetate = 5 : 1) to give **5p''** (815.8 mg, 52% for two steps). To a mixture of **5p''** (304.1 mg, 1 mmol) and $(HCHO)_n$ (90 mg, 3 mmol) was added H_2SO_4/AcOH = 1 : 4 (10 mL), and stirred at room temperature for 4 h. After quenched with water (40 mL), the mixture was filtered to collect residue solid. The residue was recrystallized (hexane/ethyl acetate) to give the product **5p** (301.2 mg, 92%) as a white solid. M.p. 175-176 °C; IR (KBr, cm^{-1}): 3063.1, 2980.5, 2937.1, 2843.0, 1925.2, 1593.1, 1489.6, 1337.3, 1162.9; ^1H NMR ($CDCl_3$, 500 MHz): δ 7.75 (d, J = 8.2 Hz, 2H), 7.56, (d, J = 7.8 Hz, 1H), 7.36 (d, J = 8.0 Hz, 2H), 7.26 (t, J = 7.5 Hz, 1H), 7.14 (t, J = 7.4 Hz, 1H), 6.98 (d, J = 7.6 Hz, 1H), 4.19 (s, 2H), 3.31 (s, 2H), 2.44 (s, 3H), 2.40-2.30 (m, 2H), 2.20-2.00 (m, 4H); ^{13}C NMR ($CDCl_3$, 125 MHz): δ 143.78, 141.23, 133.29, 130.80, 129.84, 127.88, 127.41, 126.32, 126.07, 53.68, 48.49, 41.57, 32.88, 21.63, 15.17; EI-MS m/z (%): 143.1 (100), 327.1 (15) $[M]^+$; HRMS (EI) m/z calcd for $C_{19}H_{21}NO_2S$ $[M]^+$ 327.1293, found 327.1287.

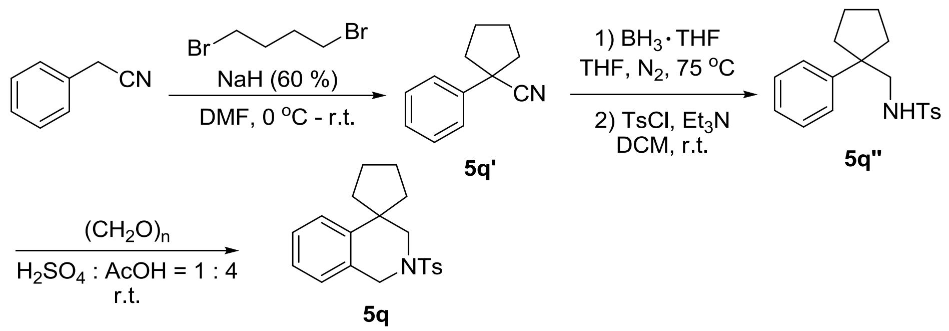

2'-Tosyl-2',3'-dihydro-1'H-spiro[cyclopentane-1,4'-isoquinoline] (5q):

To a solution of NaH (60%) (960 mg, 24 mmol) in DMF (7 mL) was added a solution of 2-phenylacetonitrile (1.17 g, 10 mmol) in DMF (11 mL) dropwise at 0 °C under N_2 atmosphere. After stirred for 40 min, 1,4-dibromobutane (2.59 g, 12 mmol) was added dropwise, and kept stirred for 5 h. The reaction was quenched by water, extracted with ethyl acetate (15 mL × 3). The combined organic phase was washed with brine (15 mL), and dried over Na_2SO_4. The resulting solution was concentrated under reduced pressure. The residue was purified by flash column chromatography on silica gel (petroleum ether/ethyl acetate = 50 : 1) to give **5q'** (1.76 g, 99%) as colorless liquid. To a solution of **5q'** (786 mg, 5 mmol) and THF (1 mL) in three-necked bottle was added BH_3·THF (1 M in THF, 15 mL) under N_2. After stirred at 75 °C overnight, the reaction was quenched with MeOH (5 mL) carefully, and concentrated under reduced pressure. After dichloromethane (8 mL) and Et_3N (1.4 mL, 10 mmol) were added in this residue, a solution of TsCl (1.14 g, 6 mmol) in dichloromethane (8 mL) was added dropwise, and stirred at room temperature for 5 h. The reaction was quenched with 2 M HCl (15 mL), extracted with dichloromethane (3 × 15 mL). The combined organic phase was washed with saturated Na_2CO_3 solution (15 mL), brine (15 mL), and dried over Na_2SO_4. The resulting solution was concentrated under reduced pressure. The residue was purified by flash column chromatography on silica gel (petroleum ether/ethyl acetate = 5 : 1) to give **5q"** (632.8 mg, 38% for two steps). To a mixture of **5q"** (240 mg, 0.73 mmol) and $(HCHO)_n$ (66 mg, 2.2 mmol) was added H_2SO_4/AcOH = 1 : 4 (7.3 mL), and stirred at room temperature for 12 h. After quenched with water (40 mL), the mixture was filtered to collect residue solid. The residue was recrystallized (hexane/ethyl acetate) to give pure product to give product **5q** (224.1 mg, 98%) as a white solid. M.p. 161-163 °C; IR (KBr, cm^{-1}): 3035.1, 2953.7, 2861.7, 1923.6, 1593.2, 1468.7, 1450.3, 1340.7, 1219.0, 1162.3; ^1H NMR ($CDCl_3$, 500 MHz): δ 7.74 (d, J = 8.3 Hz, 2H), 7.35 (d, J = 8.0 Hz, 2H), 7.27 (d, J = 8.0 Hz, 1H), 7.19 (t, J = 7.3 Hz, 1H), 7.11 (dt, J = 7.6, 1.1 Hz, 1H), 6.98 (d, J = 7.6 Hz, 1H), 4.20 (s, 2H), 3.00 (s, 2H), 2.43 (s, 3H), 2.0-1.74 (m, 8H); ^{13}C NMR ($CDCl_3$, 125 MHz): δ 143.75, 143.29, 133.34, 131.09, 129.85, 127.90, 127.38, 126.25, 126.13, 125.94, 53.63, 48.43, 46.86, 40.23, 26.10, 21.66; EI-MS m/z (%): 158.1 (100), 341.1 (24) $[M]^+$; HRMS (EI) m/z calcd for $C_{20}H_{23}NO_2S$ $[M]^+$ 341.1449, found 341.1453.

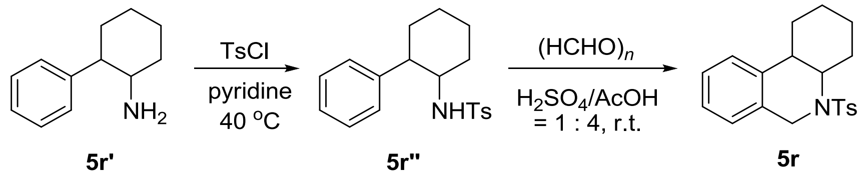

5-Tosyl-1,2,3,4,4a,5,6,10b-octahydrophenanthridine (5r):

To the solution of **5r'** (Cheng et al., 2016) (360 mg, 2 mmol) in pyridine (5 mL) was added TsCl

(570 mg, 3 mmol). After stirred at 40 °C overnight, the reaction was cooled to room temperature, then quenched with water (30 mL) and extracted with dichloromethane (3 × 15 mL). The combined organic phase was washed with water (2 × 20 mL), 2 M HCl (20 mL), saturated Na_2CO_3 solution (20 mL) and brine (20 mL), then dried over Na_2SO_4. The solvent was removed under reduced pressure, and the residue was purified by flash column chromatography on silica gel (petroleum ether/ethyl acetate = 5 : 1) to give pure **5r"** as a white solid (552.9 mg, 84%). To a mixture **5r"** (306.3 mg, 0.93 mmol) and $(HCHO)_n$ was added H_2SO_4/AcOH = 1 : 4 (10 mL) and stirred at room temperature for 12 h. The reaction was quenched with water (20 mL), then stirred for 20 min and filtered to collect the residue solid. The residue solid was washed with water and recrystallized (hexane/ethyl acetate) to give product **5r** (272.4 mg, 86%) as a white solid. M.p. 100-102 °C; IR (KBr, cm^{-1}): 3058.5, 2925.6, 2857.4, 1597.1, 1493.0, 1454.2, 1386.8, 1336.3; ^{1}H NMR ($CDCl_3$, 500 MHz): δ 7.74 (d, J = 8.0 Hz, 2H), 7.34-7.24 (m, 3H), 7.22 (t, J = 7.5 Hz, 1H), 7.17 (t, J = 7.2 Hz, 1H), 7.07 (d, J = 7.4 Hz, 1H), 4.63 (d, J = 15.9 Hz, 1H), 4.32 (d, J = 15.8 Hz, 1H), 4.21-4.08 (m, 1H), 3.07 (s, 1H), 2.45 (d, J = 14.3 Hz, 1H), 2.40 (s, 3H), 1.75-1.63 (m, 1H), 1.62-1.20 (m, 5H), 1.15-1.00 (m, 1H); ^{13}C NMR ($CDCl_3$, 125 MHz): δ 143.29, 137.30, 135.14, 132.34, 129.81, 127.22, 127.19, 126.35, 126.34, 126.06, 54.51, 44.01, 37.31, 27.86, 25.89, 25.41, 21.61, 19.87; ESI-MS m/z: 342.2 $[M+H]^+$; HRMS (ESI) m/z calcd for $C_{20}H_{24}NO_2S$ $[M+H]^+$ 342.1522, found 342.1521.

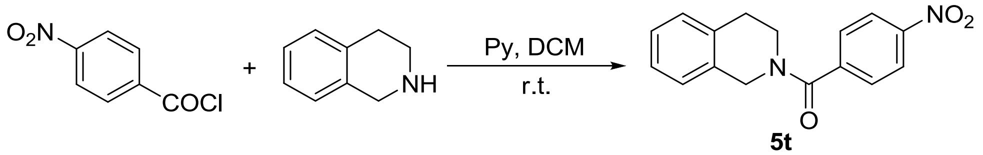

5t

(3,4-Dihydroisoquinolin-2(1H)-yl)(4-nitrophenyl)methanone (5t):

To a solution of 1,2,3,4-tetrahydroisoquinoline (133.2 mg, 1 mmol), pyridine (0.24 mL, 3 mmol) and dichloromethane (5 mL) was added a solution of 4-nitrobenzoyl chloride (278.3mg, 1.5 mmol) in dichloromethane (5 mL) dropwise. After stirred at room temperature overnight, the reaction was quenched with HCl (2 M, 10 mL), and then extracted with dichloromethane (15 mL) for three times. The combined organic phase was washed with saturated Na_2CO_3 solution (15 mL) and brine (15 mL), and then dried over Na_2SO_4. The resulting solution was concentrated under reduced pressure. The residue was purified by flash column chromatography on neutral alumina (petroleum ether/ethyl acetate/dichloromethane = 3 : 1 : 1) to give product **5t** (201.6 mg, 71%) as a white solid. M.p. 148-149 °C; IR (KBr, cm^{-1}): 2974.2, 2892.9, 1930.6, 1628.8, 1593.2, 1520.3, 1438.1; ^{1}H NMR ($CDCl_3$, 500 MHz) (rotational isomers): δ 8.30 (s, 2H), 7.62 (s, 2H), 7.35-6.80 (m, 4H), 4.90 (s, 1H), 4.51 (s, 1H), 4.01 (s, 1H), 3.59 (s, 1H), 3.01 (s, 1H), 2.88 (s, 1H); ^{13}C NMR ($CDCl_3$, 125 MHz) (rotational isomers): δ 168.70, 168.27, 148.60, 142.39, 142.27, 134.61, 133.50, 132.52, 132.20, 129.28, 128.81, 128.31, 128.04, 127.42, 127.00, 126.90, 126.68, 125.95, 124.07, 49.78, 45.35, 44.94, 40.80, 29.62, 28.25; EI-MS m/z (%): 282.1 (100) $[M]^+$; HRMS (EI) m/z calcd for $C_{16}H_{14}N_2O_3$ $[M]^+$ 282.1004, found 282.1012.

C1 Functionalization of 1,2,3,4-Tetrahydroisoquinolines, Related to Figure 4.

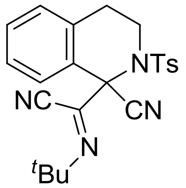

(*Z*)-*N*-(*tert*-Butyl)-1-cyano-2-tosyl-1,2,3,4-tetrahydroisoquinoline-1-carbimidoyl cyanide (6a):

To a mixture of **5a** (86.2 mg, 0.3 mmol), DDQ (204.3 mg, 0.9 mmol) and AgOTf (11.6 mg, 0.045 mmol, 15 mol%) was added PhCl (4.5 mL) and ^{t}BuNC (134 μL, 1.2 mmol) in a glovebox. The reaction was stirred at 80 ^{o}C for 3 h under N_2 atmosphere. Upon completion, the reaction mixture was cooled down to room temperature and the solvent was removed under reduced pressure. Then, purification of the residue by column chromatography on silica gel (petroleum ether/ethyl acetate = 10 : 1) to give the desired product **6a** (99.8 mg, 79%) as a white solid. M.p. 172-174 ^{o}C; IR (KBr, cm^{-1}): 2977.3, 2931.3, 2872.0, 2271.8, 1931.4, 1646.3, 1596.5, 1331.4, 1162.9; ^{1}H NMR ($CDCl_3$, 500 MHz): δ 7.88 (d, J = 8.4 Hz, 2H), 7.38 (d, J = 8.4, 2H), 7.36-7.28 (m, 3H), 7.22 (d, J = 7.2, 1H), 4.23-4.15 (m, 1H), 3.35-3.25 (m, 1H), 3.10-3.01 (m, 1H), 2.87-2.79 (m, 1H), 2.45 (s, 3H), 1.52 (s, 9H); ^{13}C NMR ($CDCl_3$, 125 MHz): δ 145.35, 137.47, 134.74, 133.45, 130.01, 129.92, 129.88, 129.02, 128.53, 128.00, 127.76, 114.27, 110.00, 66.74, 59.31, 43.01, 29.04, 28.95, 21.80; ESI-MS m/z: 421.2 [M+H]$^+$; HRMS (DART Positive) m/z calcd for $C_{23}H_{25}N_4O_2S$ [M+H]$^+$ 421.1693, found 421.1690.

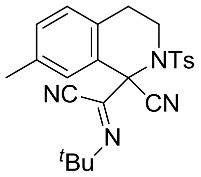

(*Z*)-*N*-(*tert*-Butyl)-1-cyano-7-methyl-2-tosyl-1,2,3,4-tetrahydroisoquinoline-1-carbimidoyl cyanide (6b):

Following the general procedure for **6a**, the reaction of **5b** (90.4 mg, 0.3 mmol), DDQ (204.3 mg, 0.9 mmol), AgOTf (11.6 mg, 15 mol%) and ^{t}BuNC (134 μL, 1.2 mmol) in PhCl (4.5 mL) at 80 ^{o}C for 3 h afforded the desired product **6b** as a white solid (98.6 mg, 76%). M.p. 181-183 ^{o}C; IR (KBr, cm^{-1}): 2974.7, 2925.4, 2868.0, 2226.8, 1914.4, 1646.3, 1599, 1362, 1164; ^{1}H NMR ($CDCl_3$, 500 MHz): δ 7.88 (d, J = 8.4 Hz, 2H), 7.38 (d, J = 8.4 Hz, 2H), 7.16-7.06 (m, 3H), 4.19-4.12 (m, 1H), 3.28-3.17 (m, 1H), 3.08-3.00 (m, 1H), 2.81-2.75 (m, 1H), 2.45 (s, 3H), 2.31 (s, 3H), 1.53 (s, 9H); ^{13}C NMR ($CDCl_3$, 125 MHz): δ 145.33, 137.89, 137.55, 133.58, 131.67, 130.93, 129.91, 129.83, 129.07, 128.27, 128.09, 114.40, 110.07, 66.70, 59.31, 43.18, 29.00, 28.65, 21.85, 21.26; ESI-MS m/z: 435.2 [M+H]$^+$; HRMS (DART Positive) m/z calcd for $C_{23}H_{25}N_4O_2S$ [M+H]$^+$ 435.1849, found 435.1849.

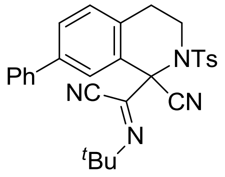

(*Z*)-*N*-(*tert*-Butyl)-1-cyano-7-phenyl-2-tosyl-1,2,3,4-tetrahydroisoquinoline-1-carbimidoyl cyanide (6c):

Following the general procedure for **6a**, the reaction of **5c** (109.5 mg, 0.3 mmol), DDQ (204.3 mg, 0.9 mmol), AgOTf (11.6 mg, 15 mol%) and ^{t}BuNC (134 μL, 1.2 mmol) in PhCl (4.5 mL) at 80 ^{o}C for 3 h afforded the desired product **6c** as a white solid (115.3 mg, 77%). M.p. 193-195 ^{o}C; IR (KBr, cm^{-1}): 2212, 1645, 1593, 1477, 1337, 1160; ^{1}H NMR ($CDCl_3$, 500 MHz): *δ* 7.91 (d, *J* = 8.2 Hz, 2H), 7.57 (d, *J* = 7.9 Hz, 1H), 7.52-7.30 (m, 9H), 4.25-4.16 (m, 1H), 3.39-3.26 (m, 1H), 3.15-3.05 (m, 1H), 2.88 (d, *J* = 16.0 Hz, 1H), 2.46 (s, 3H), 1.55 (s, 9H); ^{13}C NMR ($CDCl_3$, 125 MHz): *δ* 145.41, 141.24, 139.56, 137.50, 133.54, 133.46, 130.47, 129.93, 129.12, 129.06, 128.99, 128.78, 128.08, 126.95, 126.27, 114.28, 110.08, 66.89, 59.43, 43.10, 29.07, 28.77, 21.83; ESI-MS m/z: 497.2 [M+H]$^+$; HRMS (DART Positive) m/z calcd for $C_{29}H_{29}N_4O_2S$ [M+H]$^+$ 497.2006, found 497.2005.

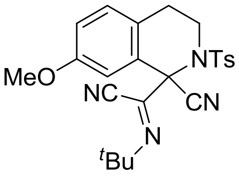

(*Z*)-*N*-(*tert*-butyl)-1-cyano-7-methoxy-2-tosyl-1,2,3,4-tetrahydroisoquinoline-1-carbimidoyl cyanide (6d):

Following the general procedure for **6a**, the reaction of **5d** (95.1 mg, 0.3 mmol), DDQ (204.3 mg, 0.9 mmol), AgOTf (11.6 mg, 15 mol%) and ^{t}BuNC (134 μL, 1.2 mmol) in PhCl (4.5 mL) at 80 ^{o}C for 3 h afforded the desired product **6d** as a white solid (135.3 mg, 99%). M.p. 177-178 ^{o}C; IR (KBr, cm^{-1}): 2979.7, 1936.6, 2249.7, 2219.1, 1644.6, 1607.7, 1503.1, 1338.5, 1279.0, 1203.3; ^{1}H NMR ($CDCl_3$, 500 MHz): *δ* 7.88 (d, *J* = 8.4 Hz, 2H), 7.37 (d, *J* = 8.2 Hz, 2H), 7.13 (d, *J* = 8.5 Hz, 1H), 6.90 (dd, *J* = 8.5, 2.6 Hz, 1H), 6.77 (d, *J* = 2.6 Hz, 1H), 4.18-4.11 (m, 1H), 3.76 (s, 3H), 3.24-3.14 (m, 1H), 3.07-2.99 (m, 1H), 2.80-2.72 (m, 1H), 2.45 (s, 3H), 1.54 (s, 9H); ^{13}C NMR ($CDCl_3$, 125 MHz): *δ* 159.02, 145.37, 137.54, 133.52, 131.03, 129.91, 129.23, 129.07, 126.67, 117.09, 114.22, 111.85, 110.02, 66.79, 59.39, 55.53, 43.33, 29.06, 28.22, 21.84; ESI-MS m/z: 451.2 [M+H]$^+$; HRMS (DART Positive) m/z calcd for $C_{24}H_{27}N_4O_3S$ [M+H]$^+$ 451.1798, found 451.1798.

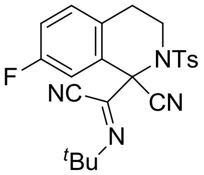

(*Z*)-*N*-(*tert*-Butyl)-1-cyano-7-fluoro-2-tosyl-1,2,3,4-tetrahydroisoquinoline-1-carbimidoyl cyanide (6e):

Following the general procedure for **6a**, the reaction of **5e** (91.6 mg, 0.3 mmol), DDQ (204.3 mg, 0.9 mmol), AgOTf (11.6 mg, 15 mol%) and ^{t}BuNC (134 μL, 1.2 mmol) in PhCl (4.5 mL) at 80 ^{o}C for 3 h afforded the desired product **6e** as a white solid (55.9 mg, 43%). M.p. 125-128 ^{o}C; IR (KBr, cm^{-1}): 2982.3, 2925.8, 2865.2, 2219.7, 1918.0, 1645.6, 1501.2, 1350.4, 1276.6, 1200.8; ^{1}H NMR ($CDCl_3$, 500 MHz): *δ* 7.87 (d, *J* = 8.4 Hz, 2H), 7.38 (d, *J* = 8.2 Hz, 2H), 7.21 (dd, *J* = 8.5, 5.6 Hz, 1H), 7.07 (dt, *J* = 5.2, 2.6 Hz, 2H), 7.02 (dd, *J* = 9.3, 2.6 Hz, 1H), 4.23-4.15 (m, 1H), 3.30-3.18 (m, 1H), 3.09-2.99 (m, 1H), 2.86-2.78, (m, 1H), 2.45 (s, 3H), 1.53 (s, 9H);

^{19}F NMR ($CDCl_3$, 470 MHz): δ -112.0 (m, Ar-F); ^{13}C NMR ($CDCl_3$, 125 MHz): δ 161.73 (d, $^1J_{C\text{-}F}$ = 248.2 Hz), 145.56, 137.27, 133.30, 131.72 (d, $^3J_{C\text{-}F}$ = 7.7 Hz), 130.56 (d, $^4J_{C\text{-}F}$ = 2.8 Hz), 130.26 (d, $^3J_{C\text{-}F}$ = 7.4 Hz), 129.97, 129.08, 117.81 (d, $^2J_{C\text{-}F}$ = 21.6 Hz), 114.49 (d, $^2J_{C\text{-}F}$ = 24.6 Hz), 113.83, 109.87, 66.63, 59.65, 43.13, 28.89, 28.49, 21.85; ESI-MS m/z: 439.2 $[M+H]^+$; HRMS (DART Positive) m/z calcd for $C_{23}H_{24}FN_4O_2S$ $[M+H]^+$ 439.1599, found 439.1599.

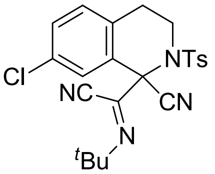

(*Z*)-*N*-(*tert*-Butyl)-7-chloro-1-cyano-2-tosyl-1,2,3,4-tetrahydroisoquinoline-1-carbimidoyl cyanide (6f):

Following the general procedure for **6a**, the reaction of **5f** (96.5 mg, 0.3 mmol), DDQ (204.3 mg, 0.9 mmol), AgOTf (11.6 mg, 15 mol%) and ^{t}BuNC (134 μL, 1.2 mmol) in PhCl (4.5 mL) at 80 ^oC for 3 h afforded the desired product **6f** as a white solid (62.4 mg, 46%). M.p. 137-139 ^oC; IR (KBr, cm^{-1}): 2982.5, 2927.9, 2218.4, 1922.3, 1643.8, 1485.1, 1348.8, 1164.7; ^1H NMR ($CDCl_3$, 500 MHz): δ 7.87 (d, J = 8.2 Hz, 2H), 7.39 (d, J = 8.1 Hz, 2H), 7.32 (dd, J = 8.1, 1.9 Hz, 1H), 7.29 (d, 1.9 Hz, 1H), 7.18 (d, J = 8.2 Hz, 1H), 4.22-4.13 (m, 1H), 3.29-3.17 (m, 1H), 3.07-2.97 (m, 1H), 2.86-2.76 (m, 1H), 2.45 (s, 3H), 1.54 (s, 9H); ^{13}C NMR ($CDCl_3$, 125 MHz): δ 145.60, 137.21, 133.80, 133.28, 133.22, 131.32, 130.39, 130.32, 129.99, 129.10, 127.84, 113.82, 109.87, 66.47, 59.68, 42.93, 28.98, 28.63, 21.86; ESI-MS m/z (%): 455.1 [M (^{35}Cl)+H]$^+$ (100), 457.1 [M (^{37}Cl)+H]$^+$ (36); HRMS (DART Positive) m/z calcd for $C_{23}H_{24}ClN_4O_2S$ $[M+H]^+$ 455.1303, found 455.1301.

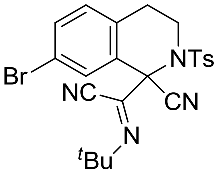

(*Z*)-7-bromo-*N*-(*tert*-butyl)-1-cyano-2-tosyl-1,2,3,4-tetrahydroisoquinoline-1-carbimidoyl cyanide (6g):

Following the general procedure for **6a**, the reaction of **5g** (109.9 mg, 0.3 mmol), DDQ (204.3 mg, 0.9 mmol), AgOTf (11.6 mg, 15 mol%) and ^{t}BuNC (134 μL, 1.2 mmol) in PhCl (4.5 mL) at 80 ^oC for 3 h afforded the desired product **6g** as a white solid (60.2 mg, 40%). M.p. 158-160 ^oC; IR (KBr, cm^{-1}): 2977.0, 2932.9, 2245.6, 2220.7, 1925.7, 1645.9, 1593.0, 1483.3, 1338.5, 1209.5; ^1H NMR ($CDCl_3$, 500 MHz): δ 7.87 (d, J = 8.3 Hz, 2H), 7.49-7.42 (m, 2H), 7.38 (d, J = 8.2 Hz, 2H), 7.11 (d, J = 8.2 Hz, 1H), 4.20-4.13 (m, 1H), 3.25-3.15 (m, 1H), 3.07-2.99 (m, 1H), 2.84-2.76 (m, 1H), 2.45 (s, 3H), 1.54 (s, 9H); ^{13}C NMR ($CDCl_3$, 125 MHz): δ 145.60, 137.22, 133.70, 133.23, 133.21, 131.54, 130.80, 130.57, 129.98, 129.08, 121.36, 113.82, 109.86, 66.26, 59.66, 42.84, 28.96, 28.65, 21.85; ESI-MS m/z (%): 499.1 [M (^{79}Br)+H]$^+$ (88), 501.1 [M (^{81}Br)+H]$^+$ (100); HRMS (DART Positive) m/z calcd for $C_{23}H_{24}BrN_4O_2S$ $[M+H]^+$ 499.0798, found 499.0798.

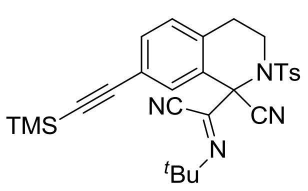

(*Z*)-*N*-(*tert*-Butyl)-1-cyano-2-tosyl-7-((trimethylsilyl)ethynyl)-1,2,3,4-tetrahydroisoquinoline-1-carbimidoyl cyanide (6h):

Following the general procedure for **6a**, the reaction of **5h** (115.1 mg, 0.3 mmol), DDQ (204.3 mg, 0.9 mmol), AgOTf (11.6 mg, 15 mol%) and ^{t}BuNC (134 μL, 1.2 mmol) in PhCl (4.5 mL) at 80 ^{o}C for 4 h afforded the desired product **6h** as a white solid (86.2 mg, 57%). M.p. 185-187 ^{o}C; IR (KBr, cm^{-1}): 2969, 2878, 2156, 1648, 1598, 1494, 1358, 1169, 852; ^{1}H NMR ($CDCl_3$, 500 MHz): δ 7.87 (d, J = 8.4 Hz, 2H), 7.42-7.35 (m, 4H), 7.16 (d, J = 8.0 Hz, 1H), 4.20-4.12 (m, 1H), 3.30-3.20 (m, 1H,), 3.09-3.00 (m, 1H), 2.86-2.78 (m, 1H), 2.45 (s, 3H), 1.54 (s, 9H), 0.24 (s, 9H); ^{13}C NMR ($CDCl_3$, 125 MHz): δ 145.50, 137.15, 134.88, 133.34, 132.99, 131.52, 129.97, 129.96, 129.07, 128.86, 123.32, 114.01, 109.92, 103.23, 96.19, 66.50, 59.60, 42.86, 28.98, 28.95, 21.84, -0.07; ESI-MS m/z: 517.2 [M+H]$^+$; HRMS (DART Positive) m/z calcd for $C_{28}H_{33}N_4O_2SSi$ [M+H]$^+$ 517.2086, found 517.2086.

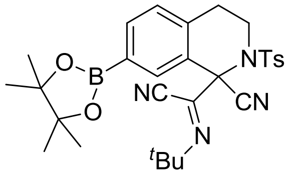

(*Z*)-*N*-(*tert*-Butyl)-1-cyano-7-(4,4,5,5-tetramethyl-1,3,2-dioxaborolan-2-yl)-2-tosyl-1,2,3,4-tetrahydroisoquinoline-1-carbimidoyl cyanide (6i):

Following the general procedure for **6a**, the reaction of **5i** (124.0 mg, 0.3 mmol), DDQ (204.3 mg, 0.9 mmol), AgOTf (11.6 mg, 15 mol%) and ^{t}BuNC (134 μL, 1.2 mmol) in PhCl (4.5 mL) at 80 ^{o}C for 3 h afforded the desired product **6i** as a white solid (81.9 mg, 50%). M.p. 240-242 ^{o}C; IR (KBr, cm^{-1}): 2983.4, 2934.5, 2873.8, 2224.2, 1915.5, 1736.4, 1645.3, 1606.0, 1334.9, 1212.4; ^{1}H NMR ($CDCl_3$, 500 MHz): δ 7.89 (d, J = 8.2 Hz, 2H), 7.74 (s, 1H), 7.72 (dd, J = 7.6, 0.8 Hz, 1H), 7.38 (d, J = 8.1 Hz, 2H), 7.21 (d, J = 7.6 Hz, 1H), 4.16-4.09 (m, 1H), 3.31-3.21 (m, 1H), 3.14-3.06 (m, 1H), 2.88-2.80 (m, 1H), 2.45 (s, 3H), 1.54 (s, 9H), 1.33 (s, 6H), 1.29 (s, 6H); ^{13}C NMR ($CDCl_3$, 125 MHz): δ 145.40, 137.38, 137.14, 135.69, 134.90, 133.46, 129.95, 129.33, 129.12, 128.01, 114.28, 110.09, 84.23, 66.76, 59.42, 42.89, 29.11, 28.90, 25.26, 24.76, 21.85; ESI-MS m/z: 547.3 [M+H]$^+$; HRMS (DART Positive) m/z calcd for $C_{29}H_{36}BN_4O_4S$ [M+H]$^+$ 546.2584, found 546.2579.

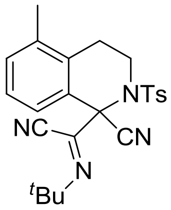

(*Z*)-*N*-(*tert*-Butyl)-1-cyano-5-methyl-2-tosyl-1,2,3,4-tetrahydroisoquinoline-1-carbimidoyl cyanide (6j):

Following the general procedure for **6a**, the reaction of **5j** (90.4 mg, 0.3 mmol), DDQ (204.3 mg,

0.9 mmol), AgOTf (11.6 mg, 15 mol%) and ^{t}BuNC (134 μL, 1.2 mmol) in PhCl (4.5 mL) at 80 ^{o}C for 3 h afforded the desired product **6j** as a white solid (97.1 mg, 74%). M.p. 197-199 ^{o}C; IR (KBr, cm^{-1}): 2973.7, 2862.5, 2407.4, 2226.6, 1916.3, 1649.8, 1595.4, 1466.4, 1411.3; ^{1}H NMR ($CDCl_3$, 500 MHz): *δ* 7.87 (d, *J* = 8.4 Hz, 2H), 7.38 (d, *J* = 8.3 Hz, 2H), 7.24-7.18 (m, 2H), 7.16-7.10 (m, 1H), 4.27-4.16 (m, 1H), 3.10-3.00 (m, 2H), 2.86-2.76 (m, 1H), 2.45 (s, 3H), 2.27 (s, 3H), 1.53 (s, 9H); ^{13}C NMR ($CDCl_3$, 125 MHz): *δ* 145.38, 137.69, 137.45, 133.38, 131.25, 129.90, 129.10, 128.58. 127.50, 125.50, 114.18, 110.01, 67.05, 59.31, 42.81, 28.97, 26.43, 21.84, 19.39; ESI-MS m/z: 435.2 $[M+H]^+$; HRMS (DART Positive) m/z calcd for $C_{24}H_{27}N_4O_2S$ $[M+H]^+$ 435.1849, found 435.1848.

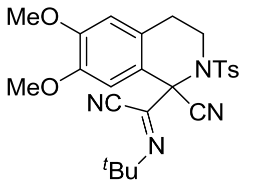

(*Z*)-*N*-(*tert*-Butyl)-1-cyano-6,7-dimethoxy-2-tosyl-1,2,3,4-tetrahydroisoquinoline-1-carbimidoyl cyanide (6k):

Following the general procedure for **6a**, the reaction of **5k** (104.2 mg, 0.3 mmol), DDQ (204.3 mg, 0.9 mmol), AgOTf (11.6 mg, 15 mol%) and ^{t}BuNC (134 μL, 1.2 mmol) in PhCl (4.5 mL) at 80 ^{o}C for 3 h afforded the desired product **6k** as a white solid (142.6 mg, 99%). M.p. 194-196 ^{o}C; IR (KBr, cm^{-1}): 2977.3, 2936.6, 2250.9, 1658.9, 1453.6, 1269.3; ^{1}H NMR ($CDCl_3$, 500 MHz): *δ* 7.88 (d, *J* = 8.4 Hz, 2H), 7.38 (d, *J* = 8.2 Hz, 2H), 6.68 (s, 1H), 6.63 (s, 1H), 4.17-4.10 (m, 1H), 3.88 (s, 3H), 3.81 (s, 3H), 3.26-3.16 (m, 1H), 3.08-3.00 (m, 1H), 2.76-2.68 (m, 1H), 2.45 (s, 3H), 1.52 (s, 9H); ^{13}C NMR ($CDCl_3$, 125 MHz): *δ* 150.42, 148.86, 145.37, 137.76, 133.52, 129.91, 129.08, 127.79, 119.41, 114.39, 111.60, 110.07, 109.44, 66.44, 59.27, 56.13, 56.09, 43.08, 29.12, 28.64, 21.84; ESI-MS m/z: 481.2 $[M+H]^+$; HRMS (DART Positive) m/z calcd for $C_{25}H_{29}N_4O_4S$ $[M+H]^+$ 481.1904, found 481.1901.

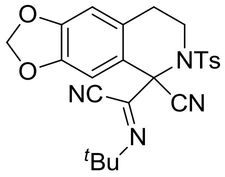

(*Z*)-*N*-(*tert*-Butyl)-5-cyano-6-tosyl-5,6,7,8-tetrahydro-[1,3]dioxolo[4,5-*g*]isoquinoline-5-carbimidoyl cyanide (6l):

Following the general procedure for **6a**, the reaction of **5l** (99.4 mg, 0.3 mmol), DDQ (204.3 mg, 0.9 mmol), AgOTf (11.6 mg, 15 mol%) and ^{t}BuNC (134 μL, 1.2 mmol) in PhCl (4.5 mL) at 80 ^{o}C for 3 h afforded the desired product **6l** as a white solid (120.0 mg, 86%). M.p. 189-191 ^{o}C; IR (KBr, cm^{-1}): 2979.2, 2915.4, 2874.6, 2249.8, 2221.3, 1646.6, 1483.2, 1341.1, 1288.7; ^{1}H NMR ($CDCl_3$, 500 MHz): *δ* 7.86 (d, *J* = 8.4 Hz, 2H), 7.37 (d, *J* = 8.2 Hz, 2H), 6.68 (s, 1H), 6.61 (s, 1H), 5.99 (dd, *J* = 5.9, 1.2 Hz, 2H), 4.17-4.09 (m, 1H), 4.24-4.13 (m, 1H), 3.05-2.96 (m, 1H), 2.74-2.66 (m, 1H), 2.44 (s, 3H), 1.52, (s, 9H); ^{13}C NMR ($CDCl_3$, 125 MHz): *δ* 149.25, 147.82, 145.38, 137.50, 133.44, 129.89, 129.34, 129.05, 120.85, 114.20, 110.05, 109.09, 106.83, 102.05, 66.79, 59.38, 43.06, 29.10, 29.02, 21.83; ESI-MS m/z: 465.2 $[M+H]^+$; HRMS (DART Positive) m/z calcd for $C_{24}H_{25}N_4O_4S$ $[M+H]^+$ 465.1591, found 465.1590.

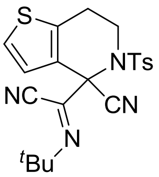

(*Z*)-*N*-(*tert*-Butyl)-4-cyano-5-tosyl-4,5,6,7-tetrahydrothieno[3,2-*c*]pyridine-4-carbimidoyl cyanide (6m):

Following the general procedure for **6a**, the reaction of **5m** (88.2 mg, 0.3 mmol), DDQ (204.3 mg, 0.9 mmol), AgOTf (11.6 mg, 15 mol%) and ^{t}BuNC (134 μL, 1.2 mmol) in PhCl (4.5 mL) at 80 °C for 3 h afforded the desired product **6m** as a white solid (56.3 mg, 44%). M.p. 156-158 °C; IR (KBr, cm^{-1}): 2978.6, 2930.4, 2875.7, 2223.4, 1921.7, 1645.6, 1595.3, 1398.7, 1337.5, 1162.0; ^{1}H NMR ($CDCl_3$, 500 MHz): δ 7.88 (d, *J* = 8.0 Hz, 2H), 7.38 (d, *J* = 8.0 Hz, 2H), 7.24 (d, *J* = 5.3 Hz, 1H), 6.77 (d, *J* = 5.4 Hz, 1H), 4.26-4.17 (m, 1H), 3.25-3.16 (m, 1H), 3.15-3.07 (m, 1H), 2.93 (d, *J* = 15.8 Hz, 1H), 2.45 (s, 3H), 1.52 (s, 9H); ^{13}C NMR ($CDCl_3$, 125 MHz): δ 145.50, 138.04, 136.66, 133.34, 129.95, 129.06, 126.81, 126.07, 124.28, 113.29, 109.79, 65.75, 59.41, 43.81, 29.03, 25.06, 21.83; ESI-MS m/z: 427.1 [M+H]$^+$; HRMS (DART Positive) m/z calcd for $C_{21}H_{23}N_4O_2S_2$ [M+H]$^+$ 427.1257, found 427.1256.

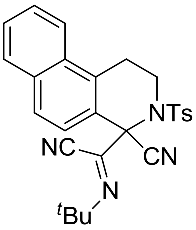

(*Z*)-*N*-(*tert*-Butyl)-4-cyano-3-tosyl-1,2,3,4-tetrahydrobenzo[*f*]isoquinoline-4-carbimidoyl cyanide (6n):

Following the general procedure for **6a**, the reaction of **5n** (101.2 mg, 0.3 mmol), DDQ (204.3 mg, 0.9 mmol), AgOTf (11.6 mg, 15 mol%) and ^{t}BuNC (134 μL, 1.2 mmol) in PhCl (4.5 mL) at 80 °C for 3 h afforded the desired product **6n** as a white solid (111.3 mg, 79%). M.p. 212-214 °C; IR (KBr, cm^{-1}): 2977.3, 2932.9, 2226.7, 1915.8, 1643.0, 1353.3, 1200.9, 1164.5; ^{1}H NMR ($CDCl_3$, 500 MHz): δ 7.97 (d, *J* = 7.7 Hz, 1H), 7.92 (d, *J* = 8.2 Hz, 2H), 7.86 (d, *J* = 8.4 Hz, 1H), 7.80 (d, *J* = 8.9 Hz, 1H), 7.65-7.56 (m, 2H), 7.40 (d, *J* = 8.2 Hz, 2H), 7.34 (d, *J* = 8.9 Hz, 1H), 4.41-4.32 (m, 1H), 3.55-3.35 (m, 2H), 3.21-3.10 (m, 1H), 2.46 (s, 3H), 1.54 (s, 9H); ^{13}C NMR ($CDCl_3$, 125 MHz): δ 145.48, 137.33, 133.34, 133.17, 132.20, 131.60, 129.96, 129.16, 128.81, 128.70, 127.93, 127.67, 125.17, 123.64, 123.24, 114.07, 109.91, 67.20, 59.53, 42.66, 29.03, 25.70, 21.86; ESI-MS m/z: 471.2 [M+H]$^+$; HRMS (DART Positive) m/z calcd for $C_{27}H_{27}N_4O_2S$ [M+H]$^+$ 471.1849, found 471.1848.

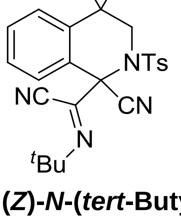

(*Z*)-*N*-(*tert*-Butyl)-1'-cyano-2'-tosyl-2',3'-dihydro-1'H-spiro[cyclopropane-1,4'-isoquinolin e]-1'-carbimidoyl cyanide (2o):

Following the general procedure for **6a**, the reaction of **5o** (94.0 mg, 0.3 mmol), DDQ (204.3

mg, 0.9 mmol), AgOTf (11.6 mg, 15 mol%) and ^{t}BuNC (134 μL, 1.2 mmol) in PhCl (4.5 mL) at 80 ^{o}C for 3 h afforded the desired product **6o** as a white solid (108.3 mg, 81%). M.p. 192-195 ^{o}C; IR (KBr, cm^{-1}): 2980.4, 2228.0, 1921.0, 1645.3, 1489.5, 1334.7, 1164.6; ^{1}H NMR ($CDCl_3$, 500 MHz): *δ* 7.85 (d, *J* = 8.3 Hz, 2H), 7.37 (d, *J* = 8.2 Hz, 2H), 7.36-7.30 (m, 1H), 7.30-7.23 (m, 2H), 6.87 (d, *J* = 7.7 Hz, 1H), 3.49 (dd, *J* = 12.4, 0.8 Hz, 1H), 3.26 (d, *J* = 12.4 Hz, 1H), 2.45 (s, 3H), 1.53 (s, 9H), 1.47-1.38 (m, 1H), 1.18-1.04 (m, 1H), 1.00-0.90 (m, 2H); ^{13}C NMR ($CDCl_3$, 125 MHz): *δ* 145.35, 139.77, 137.59, 133.50, 130.32, 129.88, 129.07, 129.02, 127.77, 127.06, 122.91, 114.14, 109.93, 67.74, 59.31, 51.54, 28.98, 21.84, 20.17, 19.78, 11.92; ESI-MS m/z: 447.2 [M+H]$^+$; HRMS (DART Positive) m/z calcd for $C_{25}H_{27}N_4O_2S$ [M+H]$^+$ 447.1849, found 447.1848.

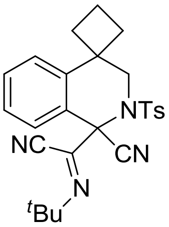

(*Z*)-*N*-(*tert*-Butyl)-1'-cyano-2'-tosyl-2',3'-dihydro-1'H-spiro[cyclobutane-1,4'-isoquinoline]-1'-carbimidoyl cyanide (6p):

Following the general procedure for **6a**, the reaction of **5p** (98.0 mg, 0.3 mmol), DDQ (204.3 mg, 0.9 mmol), AgOTf (11.6 mg, 15 mol%) and ^{t}BuNC (134 μL, 1.2 mmol) in PhCl (4.5 mL) at 80 ^{o}C for 3 h afforded the desired product **6p** as a white solid (92.6 mg, 67%). M.p. 158-161 ^{o}C; IR (KBr, cm^{-1}): 2979.4, 2936.5, 2863.9, 2220.7, 1692.2, 1648.5, 1482.7, 1356.2, 1165.5; ^{1}H NMR ($CDCl_3$, 500 MHz): *δ* 7.89 (d, *J* = 8.2 Hz, 2H), 7.66 (d, *J* = 7.9 Hz, 1H), 7.45 (t, *J* = 7.2 Hz, 1H), 7.40 (d, *J* = 8.2 Hz, 2H), 7.29 (t, *J* = 7.4 Hz, 1H), 7.22 (d, *J* = 7.9 Hz, 1H), 4.17 (d, *J* = 12.1 Hz, 1H), 3.03 (d, *J* = 12.1 Hz, 1H), 2.70-2.60 (m, 1H), 2.50-2.39 (m, 4H), 2.25-2.03 (m, 3H), 1.95-1.85 (m ,1H), 1.53 (s, 9H); ^{13}C NMR ($CDCl_3$, 125 MHz): *δ* 145.44, 142.52, 137.54, 133.34, 130.51, 129.89, 129.23, 127.67, 127.52, 127.48, 126.78, 114.01, 109.89, 67.58, 59.31, 51.82, 41.09, 34.82, 28.98, 28.85, 21.87, 15.17; ESI-MS m/z: 461.2 [M+H]$^+$; HRMS (DART Positive) m/z calcd for $C_{26}H_{29}N_4O_2S$ [M+H]$^+$ 461.2006, found 461.2006.

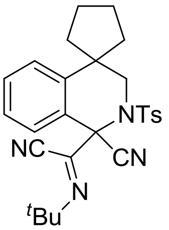

(*Z*)-*N*-(*tert*-Butyl)-1'-cyano-2'-tosyl-2',3'-dihydro-1'*H*-spiro[cyclopentane-1,4'-isoquinoline]-1'-carbimidoyl cyanide (6q):

Following the general procedure for **6a**, the reaction of **5q** (102.5 mg, 0.3 mmol), DDQ (204.3 mg, 0.9 mmol), AgOTf (11.6 mg, 15 mol%) and ^{t}BuNC (134 μL, 1.2 mmol) in PhCl (4.5 mL) at 80 ^{o}C for 3 h afforded the desired product **6q** as a white solid (77.5 mg, 54%). M.p. 234-236 ^{o}C; IR (KBr, cm^{-1}): 2972.2, 2869.4, 2226.5, 1934.9, 1647.5, 1592.7, 1484.9, 1453.4, 1339.0, 1169.6; ^{1}H NMR (acetone-d_6, 500 MHz): *δ* 7.94 (d, *J* = 8.4 Hz, 2H), 7.58 (d, *J* = 7.9 Hz, 1H), 7.52 (d, *J* = 8.2 Hz, 2H), 7.49 (t, *J* = 7.4 Hz, 1H), 7.38 (t, *J* = 7.3 Hz, 1H), 7.25 (d, *J* = 8.0 Hz, 1H), 3.92 (d, *J* = 12.6 Hz, 1H), 2.95 (d, *J* = 12.6 Hz, 1H), 2.47 (s, 3H), 2.31-2.24 (m, 1H),

2.24-2.14 (m, 1H), 2.00-1.78 (m, 3H), 1.75-1.60 (m, 3H), 1.55 (s, 9H); ^{13}C NMR (acetone-d_6, 125 MHz): δ 146.59, 145.77, 138.75, 134.35, 131.56, 130.87, 130.22, 128.95, 128.42, 128.15, 114.55, 111.17, 68.45, 59.80, 52.34, 47.37, 41.77, 38.21, 29.12, 26.79, 25.99, 21.72; ESI-MS m/z: 475.2 $[M+H]^+$; HRMS (DART Positive) m/z calcd for $C_{27}H_{31}N_4O_2S$ $[M+H]^+$ 475.2162, found 475.2158.

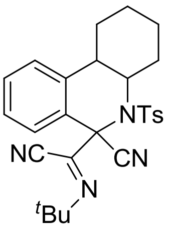

(*Z*)-*N*-(*tert*-Butyl)-6-cyano-5-tosyl-1,2,3,4,4a,5,6,10b-octahydrophenanthridine-6-carbimidoyl cyanide (6r):

Following the general procedure for **6a**, the reaction of **5r** (102.5 mg, 0.3 mmol), DDQ (204.3 mg, 0.9 mmol), AgOTf (11.6 mg, 15 mol%) and ^{t}BuNC (134 μL, 1.2 mmol) in PhCl (4.5 mL) at 80 ^{o}C for 3 h afforded the desired product **6r** as a white solid (101.1 mg, 71%, d.r. = 1:1 (determined by crude ^{1}H NMR)). One of the isomers can be obtained through recrystallization in ethyl acetate and hexane. One isomer: M.p. 235-237 ^{o}C; IR (KBr, cm^{-1}): 2933.3, 2863.6, 2216.7, 1940.8, 1645.8, 1598.1, 1454.4; ^{1}H NMR ($CDCl_3$, 500 MHz): δ 8.02 (d, J = 8.3 Hz, 2H), 7.47 (t, J = 7.4 Hz, 1H), 7.41 (d, J = 7.6 Hz, 1H), 7.37 (d, J = 8.2 Hz, 2H), 7.34 (t, J = 7.6 Hz, 1H), 7.21 (d, J = 7.9 Hz, 1H), 4.05 (dt, J = 12.2, 4.2 Hz, 1H), 3.12 (s, 1H), 2.51 (d, J =14.6 Hz, 1H), 2.45 (s, 3H), 1.96 (d, J = 13.1 Hz, 1H), 1.79-1.66 (m, 2H), 1.64 (s, 9H), 1.52-1.47 (m, 1H), 1.43-1.31 (m, 2H), 1.25-1.12 (m ,1H); ^{13}C NMR ($CDCl_3$, 125 MHz): δ 144.97, 136.87, 136.30, 135.38, 130.56, 129.85, 129.59, 128.78, 127.92, 127.82, 126.26, 115.16, 110.51, 64.43, 59.78, 57.80, 36.53, 30.68, 29.15, 27.06, 25.95, 21.81, 19.32; ESI-MS m/z: 475.2 $[M+H]^+$; HRMS (DART Positive) m/z calcd for $C_{20}H_{19}N_3$ $[M+H]^+$ 475.2162, found 475.2157.

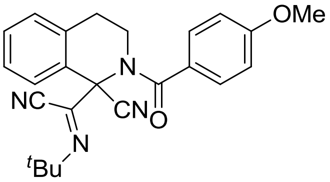

(*Z*)-*N*-(*tert*-Butyl)-1-cyano-2-(4-methoxybenzoyl)-1,2,3,4-tetrahydroisoquinoline-1-carbimidoyl cyanide (6s):

Following the general procedure for **6a**, the reaction of **5s** (80.0 mg, 0.3 mmol), DDQ (204.3 mg, 0.9 mmol), AgOTf (11.6 mg, 15 mol%) and ^{t}BuNC (134 μL, 1.2 mmol) in PhCl (4.5 mL) at 80 ^{o}C for 3 h afforded the desired product **6s** as a white solid (75.0 mg, 62%). M.p. 158-160 ^{o}C; IR (KBr, cm^{-1}): 2979.6, 2937.5, 2220.7, 1646.4, 1605.2, 1508.6, 1422.9, 1369.9, 1250.1, 1174.6; ^{1}H NMR ($CDCl_3$, 500 MHz): δ 7.66-7.58 (m, 3H), 7.43-7.34 (m, 2H), 7.31-7.26 (m, 1H), 6.99 (d, J = 8.7 Hz, 2H), 4.31-4.24 (m, 1H), 3.87 (s, 3H), 3.53-3.31 (m, 2H), 2.90-2.80 (m, 1H), 1.44 (s, 9H); ^{13}C NMR ($CDCl_3$, 125 MHz): δ 172.94, 162.23, 136.99, 135.57, 129.80, 129.76, 129.73, 128.60, 128.24, 128.02, 126.16, 116.76, 114.35, 109.94, 65.27, 59.34, 55.61, 45.20, 29.30, 29.06; ESI-MS m/z: 401.2 $[M+H]^+$; HRMS (DART Positive) m/z calcd for $C_{24}H_{25}N_4O_2$ $[M+H]^+$ 401.1972, found 401.1971.

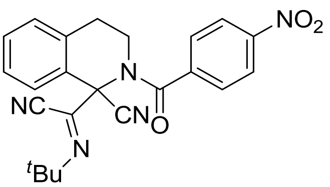

(*Z*)-*N*-(*tert*-Butyl)-1-cyano-2-(4-nitrobenzoyl)-1,2,3,4-tetrahydroisoquinoline-1-carbimidoyl cyanide (6t):

Following the general procedure for **6a**, the reaction of **5t** (85.0 mg, 0.3 mmol), DDQ (204.3 mg, 0.9 mmol), AgOTf (11.6 mg, 15 mol%) and ^{t}BuNC (134 µL, 1.2 mmol) in PhCl (4.5 mL) at 80 ^{o}C for 3 h afforded the desired product **6t** as a white solid (59.3 mg, 48%). M.p. 198-200 ^{o}C; IR (KBr, cm^{-1}): 2975.0, 2930.6, 2245.6, 2214.1, 1765.4, 1658.2, 1523.5, 1347.9; ^{1}H NMR ($CDCl_3$, 500 MHz): δ 8.37 (d, J = 8.6 Hz, 2H), 7.81 (d, J = 8.7 Hz, 2H), 7.66-7.60 (m, 1H), 7.44-7.37 (m, 2H), 7.32-7.27 (m, 1H), 4.07-4.00 (m, 1H), 3.55-3.46 (m, 1H), 3.39-3.28 (m, 1H), 2.91-2.82 (m, 1H), 1.44 (s, 9H); ^{13}C NMR ($CDCl_3$, 125 MHz): δ 170.70, 149.41, 140.13, 136.36, 134.91, 130.14, 129.78, 128.50, 128.42, 127.59, 124.45, 116.21, 109.98, 65.03, 59.67, 44.93, 29.05, 29.00; ESI-MS m/z: 416.2 [M+H]$^{+}$; HRMS (DART Positive) m/z calcd for $C_{23}H_{22}N_5O_3$ [M+H]$^{+}$ 416.1717, found 416.1717.

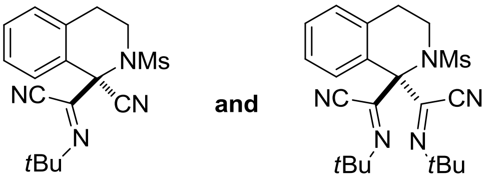

6u **6u'**

(*Z*)-*N*-(*tert*-butyl)-1-cyano-2-(methylsulfonyl)-1,2,3,4-tetrahydroisoquinoline-1-carbimidoyl cyanide (6u) and (1*Z*,1*Z*)-*N*,*N*'-di-*tert*-butyl-2-(methylsulfonyl)-3,4-dihydroisoquinoline-1,1(2*H*)-bis(carbimidoyl) dicyanide (6u'):

To a test tube, **5u** (63.4 mg, 0.3 mmol), ^{t}BuNC (136.0 µL, 1.2 mmol), AgOTf (11.6 mg, 0.045 mmol), DDQ (204.3 mg, 0.9 mmol) and dry chlorobenzene (3.0 mL) were added in a glove box. The mixture was stirred at 80 ^{o}C for 3 h under a nitrogen atmosphere as monitored by TLC. Upon completion of the reaction, the solution was cooled down to room temperature. After removal of the solvent, the residue was purified by column chromatography on silica gel (petroleum ether/ethyl acetate = 6 : 1 to 3 : 1) to give the product **6u** (59.1 mg, 57 %) and **6u'** (20.5 mg, 16 %), respectively, as a yellow solid.

6u: M.p. 161-162 ^{o}C; IR (KBr, cm^{-1}): 3432.8, 2979.4, 2870.9, 2220.7, 1644.7, 1351.0, 1165.1, 964.0, 767.7, 495.3; ^{1}H NMR ($CDCl_3$, 500 MHz): δ 7.40-7.34 (m, 3H), 7.28 (d, J =7.3 Hz, 1H), 4.22-4.18 (m, 1H), 3.40-3.33 (m, 1H), 3.27-3.22 (m, 1H), 3.20 (s, 3H), 2.95-2.91 (m, 1H), 1.44 (s, 9H); ^{13}C NMR ($CDCl_3$, 125 MHz): δ 137.56, 134.79, 130.13, 130.07, 128.19, 127.95, 127.72, 115.73, 109.64, 66.96, 59.32, 42.75, 37.56, 29.21, 28.87; ESI-MS m/z: 345.14 [M^{+}H]; HRMS (DART) m/z calcd for $C_{17}H_{21}N_4O_2S$ [M^{+}H] 345.1380, found 345.1380.

6u': M.p. 147-148 ^{o}C; IR (KBr, cm^{-1}): 3433.7, 2975.3, 2216.6, 1643.5, 1340.1, 1152.9, 1075.5, 777.0; ^{1}H NMR ($CDCl_3$, 500 MHz): δ 7.44-7.42 (m, 1H), 7.38-7.33 (m, 2H), 7.26-7.24 (m, 1H), 3.61 (t, J = 6.0 Hz, 2H), 3.14 (s, 3H), 3.02 (t, J =6.0 Hz, 2H), 1.46 (s, 18H); ^{13}C NMR ($CDCl_3$, 125 MHz): δ 138.23, 135.59, 129.97, 129.52, 129.25, 129.13, 127.00, 111.52, 76.45, 59.44,

42.86, 42.38, 29.33, 28.92; ESI-MS m/z: 428.21 $[M+H]^+$; HRMS (DART) m/z calcd for $C_{22}H_{30}N_5O_2S$ $[M+H]^+$ 428.2115, found 428.2116.

Synthesis and Characterization of 5,6-Dihydrophenanthridines, Related to Figure 5

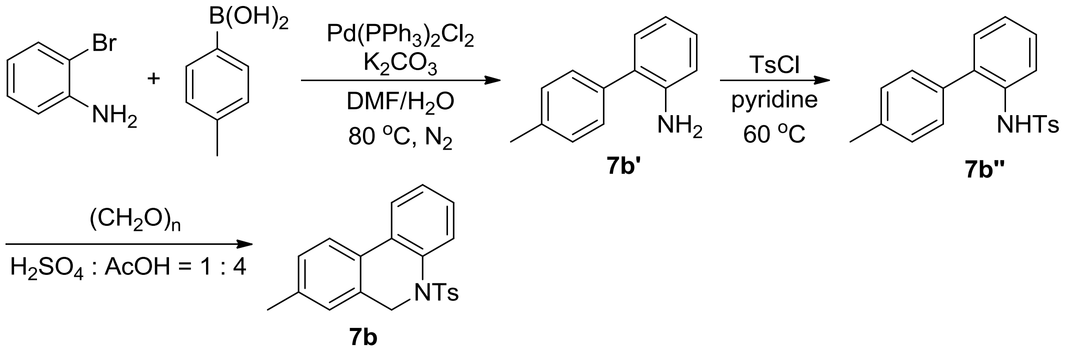

8-Methyl-5-tosyl-5,6-dihydrophenanthridine (7b):

To a mixture of 2-bromoaniline (1.72 g, 10 mmol), *p*-tolylboronic acid (1.43 g, 10.5 mmol), $Pd(PPh_3)_2Cl_2$ (351 mg, 0.5 mmol), K_2CO_3 (5.53 g, 40 mmol) was added water (10 mL) and DMF (40 mL), then reduced pressure and backfilled with N_2. After stirred at 80 ^{o}C for 2 h, the mixture was cooled down to room temperature and filtered on diatomite. The filtrate was washed with water (2 × 15 mL) and brine (20 mL), and dried over Na_2SO_4. The resulting solution was concentrated under reduced pressure. The residue was purified by flash column chromatography on silica gel (petroleum ether/ethyl acetate = 50 : 1) to give pure **7b'** (1.53 g, 83%). To a solution of **7b'** (1.53 g, 8.34 mmol) in pyridine (15 mL) was added TsCl (1.75 g, 9.17 mmol) portionwise. After stirred at 60 ^{o}C for 12 h, pyridine was removed under vacuum. To this residue was added dichloromethane (20 mL) and 2 M HCl (20 mL). After stirred for 10 min, the mixture was extracted by dichloromethane (2 × 15 mL). The combined organic phase was washed with saturated Na_2CO_3 solution (15 mL) and brine (15 mL), and then dried over Na_2SO_4. The resulting solution was concentrated under reduced pressure to give crude **7b"** (2.39 g, 85%) without further purification.

To a mixture of **7b"** (1.687 g, 5 mmol) and $(HCHO)_n$ (450 mg, 15 mmol) was added H_2SO_4/AcOH = 1 : 4 (25 mL). After stirred at room temperature for 5 h, the reaction was quenched with water (50 mL), then filtered and washed with water for several times. The residue was collected and purified by flash column chromatography on basic alumina (petroleum ether/ethyl acetate/dichloromethane = 20 : 1 : 2) to give **7b** (1.42 g, 81%) as a white solid. M.p. 150-152 ^{o}C; IR (KBr, cm^{-1}): 3038.3, 2989.5, 2908.9, 1915.7, 1591.6, 1476.7, 1341.7, 1200.2, 1158.1; ^{1}H NMR ($CDCl_3$, 500 MHz): δ 7.77 (dd, J = 7.9, 1.2 Hz, 1H), 7.54 (dd, J = 7.5, 1.4 Hz, 1H), 7.36-7.29 (m, 2H), 7.11 (d, J = 7.8 Hz, 2H), 6.96 (d, J = 8.3 Hz, 2H), 6.90 (d, J = 7.9 Hz, 1H), 6.87 (s, 1H), 6.69 (d, J = 8.1 Hz, 2H), 4.79 (s, 2H), 2.30 (s, 3H), 2.15 (s, 3H); ^{13}C NMR ($CDCl_3$, 125 MHz): δ 142.95, 137.92, 135.86, 134.88, 131.37, 130.85, 128.42, 128.37, 128.31, 128.18, 127.99, 127.47, 127.23, 126.80, 123.58, 123.02, 49.96, 21.38, 21.15; EI-MS m/z (%): 194.1 (100), 349.1 (17) $[M]^+$; HRMS (EI) m/z calcd for $C_{21}H_{19}NO_2S$ $[M]^+$ 349.1136, found 349.1140.

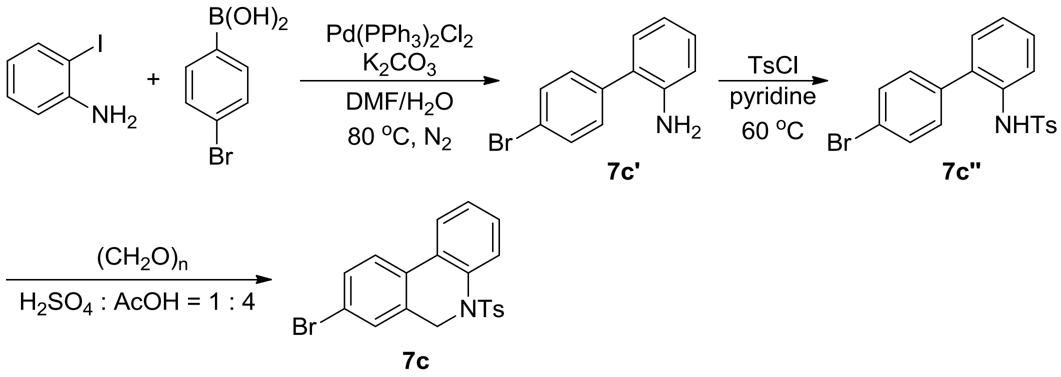

8-Bromo-5-tosyl-5,6-dihydrophenanthridine (7c):

To a mixture of 2-iodoaniline (2.2 g, 10 mmol), (4-bromophenyl)boronic acid (2.04 g, 10.2mmol), $Pd(PPh_3)_2Cl_2$ (140.4 mg, 0.2 mmol), K_2CO_3 (5.52 g, 40 mmol) was added water (10 mL) and DMF (40 mL), then reduced pressure and backfilled with N_2. After stirred at 80 ^oC for 2 h, the mixture was cooled down to room temperature and filtered on diatomite. The filtrate was washed with water (2 × 15 mL) and brine (20 mL), and dried over Na_2SO_4. The resulting solution was concentrated under reduced pressure. The residue was purified by flash column chromatography on silica gel (petroleum ether/ethyl acetate = 50 : 1) to give pure **7c'** (2.16 g, 87%). To a solution of **7c'** (2.16 g, 8.7 mmol) in pyridine (15 mL) was added TsCl (2.00 g, 10.44 mmol) portionwise. After stirred at 60 ^oC for 9 h, pyridine was removed under vacuum. To this residue was added dichloromethane (20 mL) and 2M HCl (20 mL). After stirred for 10 min, the mixture was extracted by dichloromethane (2 × 15 mL). The combined organic phase was washed with saturated Na_2CO_3 solution (15 mL) and brine (15 mL), and then dried over Na_2SO_4. The resulting solution was concentrated under reduced pressure to give crude **7c"** (2.78 g, 80%) without further purification.

To a mixture of **7c"** (371.7 mg, 1 mmol) and $(HCHO)_n$ (90 mg, 3 mmol) was added H_2SO_4/AcOH = 1 : 4 (10 mL). After stirred at room temperature for 24 h, the reaction was quenched with water (30 mL), and the mixture was extracted by dichloromethane (2 × 15 mL). The combined organic phase was washed with saturated Na_2CO_3 solution (15 mL) and brine (15 mL), and dried over Na_2SO_4. The solvent was removed under vacuum, and the residue was purified by flash column chromatography on neutral alumina (petroleum ether/ethyl acetate/dichloromethane = 20 : 1 : 2) to give pure product **7c** (159.3 mg, 38%) as a white solid. M.p. 153-155 ^oC; IR (KBr, cm^{-1}): 3054.7, 2917.9, 1909.3, 1590.0, 1474.1, 1439.1, 1403.6, 1341.1; ^1H NMR ($CDCl_3$, 500 MHz): δ 7.78 (d, J = 7.9 Hz, 1H), 7.54 (d, J = 7.6 Hz, 1H), 7.41 (t, J = 7.6 Hz, 1H), 7.34 (t, J = 7.5 Hz, 1H), 7.21 (d, J = 8.2 Hz, 1H), 7.18 (s, 1H), 7.08 (d, J = 8.2 Hz, 1H), 6.98 (d, J = 8.1 Hz, 2H), 6.76 (d, J = 8.0 Hz, 2H), 4.77 (s, 2H), 2.19 (s, 3H); ^{13}C NMR ($CDCl_3$, 125 MHz): δ 143.48, 136.05, 134.65, 133.28, 130.63, 130.13, 129.76, 129.21, 128.95, 128.64, 128.49, 127.73, 127.23, 124.66, 123.70, 121.93, 49.35, 21.46; EI-MS m/z (%): 258.0 (100), 413.0 (30) [M (^{79}Br)]$^+$, 415.0 (22) [M (^{81}Br)]$^+$; HRMS (EI) m/z calcd for $C_{20}H_{16}BrNO_2S$ $[M]^+$ 413.0085, found 413.0080.

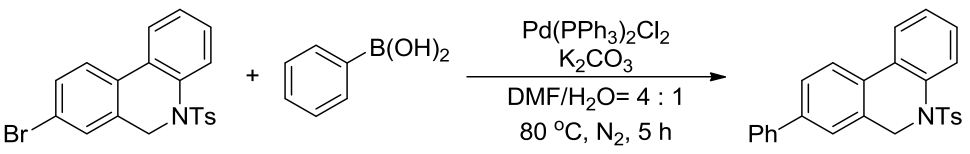

7c **7d**

8-Phenyl-5-tosyl-5,6-dihydrophenanthridine (7d):

To a mixture of **7c** (248.6 mg, 0.6 mmol), phenylboronic acid (88 mg, 0.72 mmol), K_2CO_3 (332 mg, 2.4 mmol), $Pd(PPh_3)_2Cl_2$ (21 mg, 0.03 mmol) were added water (0.6 mL) and DMF (2.4 mL). After stirred at 80 ^oC for 5 h under N_2, the reaction mixture was filtered, and the filter residue was washed with ethyl acetate. The resulting solution was concentrated under reduced pressure. The residue was purified by flash column chromatography on basic Al_2O_3 (petroleum ether/ethyl acetate = 10 : 1) to give product **7d** (153.2 mg, 62%) as a white solid. M.p.144-146 ^oC; IR (KBr, cm^{-1}): 3030.8, 2918.0, 1918.0, 1593.1, 1470.8, 1342.2, 1157.9, 1077.8; ^1H NMR ($CDCl_3$, 500 MHz): δ 7.81 (d, J = 7.5 Hz, 1H), 7.60 (d, J = 7.2 Hz, 1H), 7.56-7.42 (m, 4H), 7.42-7.16 (m, 6H), 6.98 (d, J = 7.6 Hz, 2H), 6.66 (d, J = 7.5 Hz, 2H), 4.86 (s, 2H), 2.09 (s, 3H); ^{13}C NMR ($CDCl_3$, 125 MHz): δ 143.09, 140.82, 140.31, 136.13, 134.73, 131.76, 130.45, 130.07, 129.05, 128.45, 128.43, 128.37, 127.77, 127.60, 127.22, 126.89, 126.27, 124.84, 123.78, 123.56, 50.05, 21.37; EI-MS m/z (%): 256.1 (100) $[M–Ts]^+$, 301.1 (22) $[M]^+$; HRMS (EI) m/z calcd for $C_{26}H_{21}NO_2S$ $[M]^+$ 411.1293, found 411.1289.

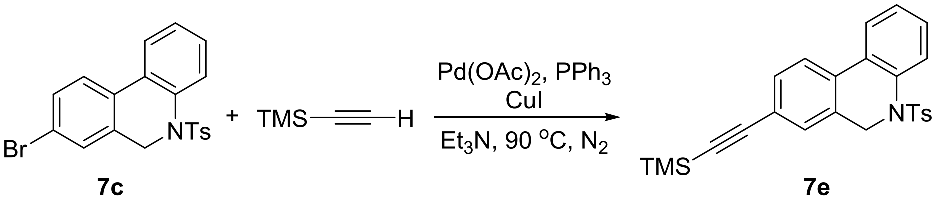

7c **7e**

5-Tosyl-8-((trimethylsilyl)ethynyl)-5,6-dihydrophenanthridine (7e):

To a mixture of **7c** (331.4 mg, 0.8 mmol), $Pd(OAc)_2$ (9.0 mg, 0.04 mmol), CuI (7.6 mg, 0.04 mmol) in a schlenk tube was added ethynyltrimethylsilane (340 μL, 2.4 mmol) and Et_3N (2.5 mL) under N_2. After stirred at 90 ^oC for 24 h, the reaction was concentrated under reduced pressure. The residue was purified by flash column chromatography on silica gel (petroleum ether/ethyl acetate = 10 : 1) to give **7e** (196.4 mg, 57%) as a white solid. M.p. 162-164 ^oC; IR (KBr, cm^{-1}): 2958.2, 2142.1, 1597.0, 1466.2, 1434.4, 1240.9, 1160.0; ^1H NMR ($CDCl_3$, 500 MHz): δ 7.78 (d, J = 7.9 Hz, 1H), 7.54 (d, J = 7.5 Hz, 1H), 7.38 (t, J = 7.2 Hz, 1H), 7.32 (t, J = 7.5 Hz, 1H), 7.22-7.10 (m, 3H), 6.97 (d, J = 8.2 Hz, 2H), 6.74 (d, J = 8.0 Hz, 2H), 4.78 (s, 2H), 2.17 (s, 3H), 0.28 (s, 9H); ^{13}C NMR ($CDCl_3$, 125 MHz): δ 143.32, 136.26, 134.69, 131.38, 131.12, 131.08, 130.02, 129.53, 128.90, 128.59, 128.31, 127.58, 127.23, 124.00, 122.84, 122.69, 104.50, 95.78, 49.56, 21.43, 0.10; EI-MS m/z (%): 276.1 (100) $[M–Ts]^+$; 431.1 (40) $[M]^+$; HRMS (EI) m/z calcd for $C_{25}H_{25}NO_2SSi$ $[M]^+$ 431.1375, found 431.1372.

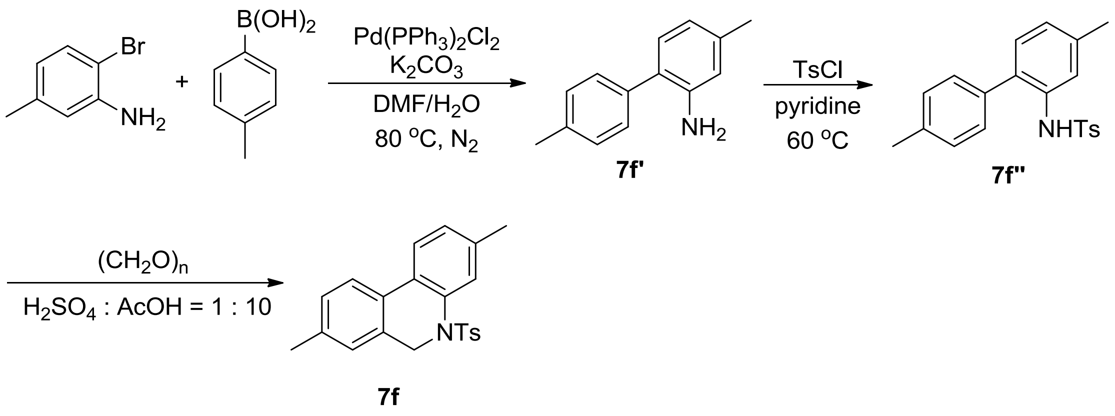

3,8-Dimethyl-5-tosyl-5,6-dihydrophenanthridine (7f):

To a mixture of 2-bromo-5-methylaniline (930 mg, 5 mmol), *p*-tolylboronic acid (748 mg, 5.5 mmol), $Pd(PPh_3)_2Cl_2$ (70.1 mg, 0.1 mmol), K_2CO_3 (2.76 g, 20 mmol) was added water (10 mL) and DMF (40 mL), then reduced pressure and backfilled with N_2. After stirred at 80 °C for 2 h, the mixture was cooled down to room temperature and filtered on diatomite. The filtrate was washed with water (2 × 15 mL) and brine (20 mL), and dried over Na_2SO_4. The resulting solution was concentrated under reduced pressure. The residue was purified by flash column chromatography on silica gel (petroleum ether/ethyl acetate = 50 : 1) to give **7f'** (835.7 mg, 85%) . To a solution of **7f'** (835.7 mg, 4.24 mmol) in pyridine (10 mL) was added TsCl (969 mg, 5.1 mmol) portionwise. After stirred at 60 °C for 12 h, pyridine was removed under vacuum. To this residue was added dichloromethane (20 mL) and 2 M HCl (20 mL). After stirred for 10 min, the mixture was extracted by dichloromethane (2 × 15 mL). The combined organic phase was washed with saturated Na_2CO_3 solution (15 mL) and brine (15 mL), and dried over Na_2SO_4. The resulting solution was concentrated under reduced pressure to give crude **7f''** (1.32 g, 89%) without further purification.

To a mixture of **7f''** (351.5 mg, 1 mmol) and $(HCHO)_n$ (90 mg, 3 mmol) was added H_2SO_4/AcOH = 1 : 10 (10 mL). After stirred at room temperature for 5 h, the reaction was quenched with water (20 mL), then filtered and washed with water for several times. The residue was collected and purified by flash column chromatography on neutral alumina (petroleum ether/ethyl acetate/dichloromethane = 20 : 1 : 2) to give pure product **7f** (210.4 mg, 58%) as a white solid. M.p. 176-178 °C; IR (KBr, cm^{-1}): 3026.8, 2917.9, 2858.8, 1906.7, 1603.6, 1518.3, 1479.8, 1342.4, 1285.5, 1160.9; ^1H NMR ($CDCl_3$, 500 MHz): δ 7.59 (d, J = 0.5 Hz, 1H), 7.42 (d, J = 7.9 Hz, 1H), 7.12 (dd, J = 7.9, 1.1 Hz, 1H), 7.07 (d, J = 7.9 Hz, 1H), 6.97 (d, J = 8.3 Hz, 2H), 6.88 (d, J = 8.0 Hz, 1H), 6.84 (s, 1H), 6.70 (d, J = 7.9 Hz, 2H), 4.76 (d, 2H), 2.43 (s, 3H), 2.28 (s, 3H), 2.15 (s, 3H); ^{13}C NMR ($CDCl_3$, 125 MHz): δ 142.89, 138.13, 137.41, 135.76, 134.92, 131.00, 128.58, 128.56, 128.41, 128.33, 128.22, 128.12, 127.26, 126.75, 123.39, 122.71, 50.09, 21.47, 21.38, 21.12; EI-MS m/z (%): 208.1 (100), 363.1 (26) $[M]^+$; HRMS (EI) m/z calcd for $C_{22}H_{21}NO_2S$ $[M]^+$ 363.1293, found 363.1292.

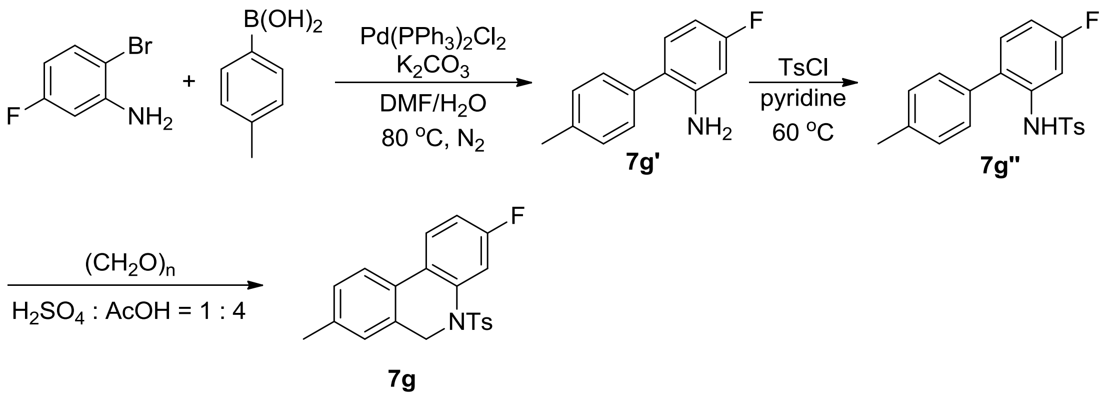

3-Fluoro-8-methyl-5-tosyl-5,6-dihydrophenanthridine (7g):

To a mixture of 2-bromo-5-fluoroaniline (950 mg, 5 mmol), *p*-tolylboronic acid (1.02 g, 7.5 mmol), $Pd(PPh_3)_2Cl_2$ (175.5 mg, 0.25 mmol), K_2CO_3 (2.76 g, 20 mmol) was added water (5 mL) and DMF (20 mL), then reduced pressure and backfilled with N_2. After stirred at 80 ^oC for 2 h, the mixture was cooled down to room temperature and filtered on diatomite. The filtrate was washed with water (2 × 15 mL) and brine (20 mL), and dried over Na_2SO_4. The resulting solution was concentrated under reduced pressure. The residue was purified by flash column chromatography on silica gel (petroleum ether/ethyl acetate = 50 : 1) to give **7g’** (977.3 mg, 97%) . To a solution of **7g’** (977.3 mg, 4.86 mmol) in pyridine (10 mL) was added TsCl (1.38 g, 7.29 mmol) portionwise. After stirred at 60 ^oC for 12 h, pyridine was removed under vacuum. To this residue was added dichloromethane (20 mL) and 2 M HCl (20 mL). After stirred for 10 min, the mixture was extracted by dichloromethane (2 × 15 mL). The combined organic phase was washed with saturated Na_2CO_3 solution (15 mL) and brine (15 mL), and dried over Na_2SO_4. The resulting solution was concentrated under reduced pressure to give crude **7g”** (1.43 g, 83%) without further purification.

To a mixture of **7g”** (351.5 mg, 1 mmol) and $(HCHO)_n$ (90 mg, 3 mmol) was added H_2SO_4/AcOH = 1 : 4 (10 mL). After stirred at room temperature for 12 h, the reaction was quenched with water (20 mL), then filtered and washed with water for several times. The residue was collected and purified by flash column chromatography on neutral alumina (petroleum ether/ethyl acetate/dichloromethane = 20 : 1 : 2) to give product **7g** (286.7 mg, 78%) as a white solid. M.p. 152-153 ^oC; IR (KBr, cm^{-1}): 3034.3, 2916.6, 1918.5, 1603.4, 1481.5, 1334.2, 1279.1, 1160.0; ^1H NMR ($CDCl_3$, 500 MHz): δ 7.58-7.45 (m, 2H), 7.07 (d, J = 7.9 Hz, 1H), 7.05-6.97 (m, 3H), 6.91 (d, J = 8.0 Hz, 1H), 6.88 (s, 1H), 6.73 (d, J = 8.1 Hz, 2H), 4.79 (s, 2H), 2.30 (s, 3H), 2.18 (s, 3H); ^{19}F NMR ($CDCl_3$, 470 MHz): δ -112.53 (m, Ar-F); ^{13}C NMR ($CDCl_3$, 125 MHz): δ 161.90 (d, $^1J_{C\text{-}F}$ = 247.1 Hz), 143.27, 137.90, 137.27 (d, $^3J_{C\text{-}F}$ = 11.0 Hz), 134.85, 130.81, 128.55, 128.51, 127.84, 127.24, 127.09 (d, $^4J_{C\text{-}F}$ =3.6 Hz), 126.83, 124.87 (d, $^3J_{C\text{-}F}$ = 9.0 Hz), 122.81, 115.18 (d, $^2J_{C\text{-}F}$ = 24.0 Hz), 114.76 (d, $^2J_{C\text{-}F}$ = 21.6 Hz), 49.95, 21.44, 21.16; EI-MS m/z (%): 212.1 (100), 367.1 (25) $[M]^+$; HRMS (EI) m/z calcd for $C_{21}H_{18}FNO_2S$ $[M]^+$ 367.1042, found 367.1034.

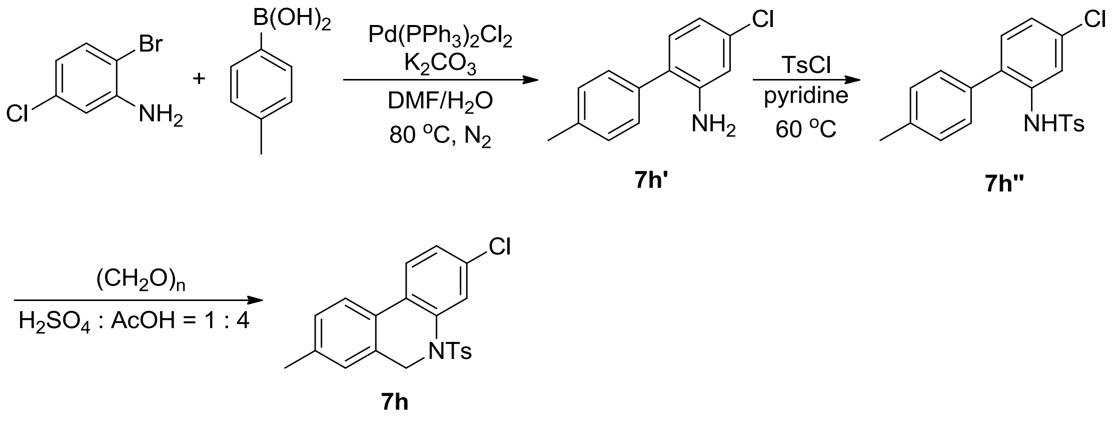

3-Chloro-8-methyl-5-tosyl-5,6-dihydrophenanthridine (7h):

To a mixture of 2-bromo-5-chloroaniline (1.03 g, 5 mmol), *p*-tolylboronic acid (1.02 g, 7.5 mmol), $Pd(PPh_3)_2Cl_2$ (175.5 mg, 0.25 mmol), K_2CO_3 (2.76 g, 20 mmol) was added water (5 mL) and DMF (20 mL), then reduced pressure and backfilled with N_2. After stirred at 80 °C for 2 h, the mixture was cooled down to room temperature and filtered on diatomite. The filtrate was washed with water (2 × 15 mL) and brine (20 mL), and dried over Na_2SO_4. The resulting solution was concentrated under reduced pressure. The residue was purified by flash column chromatography on silica gel (petroleum ether/ethyl acetate = 50 : 1) to give pure **7h'** (760.1 mg, 70%). To a solution of **7h'** (760.1 mg, 3.5 mmol) in pyridine (7 mL) was added TsCl (995 mg, 5.24 mmol) portionwise. After stirred at 60 °C for 12 h, pyridine was removed under vacuum. To this residue was added dichloromethane (20 mL) and 2 M HCl (20 mL). After stirred for 10 min, the mixture was extracted by dichloromethane (2 × 15 mL). The combined organic phase was washed with saturated Na_2CO_3 solution (15 mL) and brine (15 mL), and dried over Na_2SO_4. The resulting solution was concentrated under reduced pressure to give crude **7h"** (1.43g, 83%) without further purification.

To a mixture of **7h"** (371.7 mg, 1 mmol) and $(HCHO)_n$ (90 mg, 3 mmol) was added H_2SO_4/AcOH = 1 : 4 (10 mL). After stirred at room temperature for 12 h, the reaction was quenched with water (20 mL), then filtered and washed with water for several times. The residue was collected and purified by flash column chromatography on neutral alumina (petroleum ether/ethyl acetate/dichloromethane = 20 : 1 : 2) to give product **7h** (340.7 mg, 89%) as a white solid. M.p. 152-153 °C; IR (KBr, cm^{-1}): 3030.2, 2915.9, 2855.3, 1906.9, 1591.2, 1465.0, 1337.5, 1158.4; ^1H NMR ($CDCl_3$, 500 MHz): δ 7.79 (d, J = 2.2 Hz, 1H), 7.46 (d, J = 8.5 Hz, 1H), 7.27 (dd, J = 8.5, 2.2 Hz, 1H), 7.08 (d, J = 7.9 Hz, 1H), 7.00 (d, J = 8.3 Hz, 2H), 6.91 (d, J = 7.9 Hz, 1H), 6.86 (s, 1H), 6.72 (d, J = 8.0 Hz, 2H), 4.76 (s, 2H), 2.29 (s, 3H), 2.16 (s, 3H); ^{13}C NMR ($CDCl_3$, 125 MHz): δ 143.28, 138.34, 136.82, 134.70, 133.15, 131.08, 129.33, 128.51, 128.49, 128.01, 127.67, 127.55, 127.19, 126.83, 124.60, 122.91, 49.82, 21.38, 21.15; EI-MS m/z (%): 227.0 (100), 385.1 (28) [M (^{37}Cl)]$^+$, 383.1 (83) [M (^{35}Cl)]$^+$; HRMS (EI) m/z calcd for $C_{21}H_{18}ClNO_2S$ [M]$^+$ 383.0747, found 383.0740.

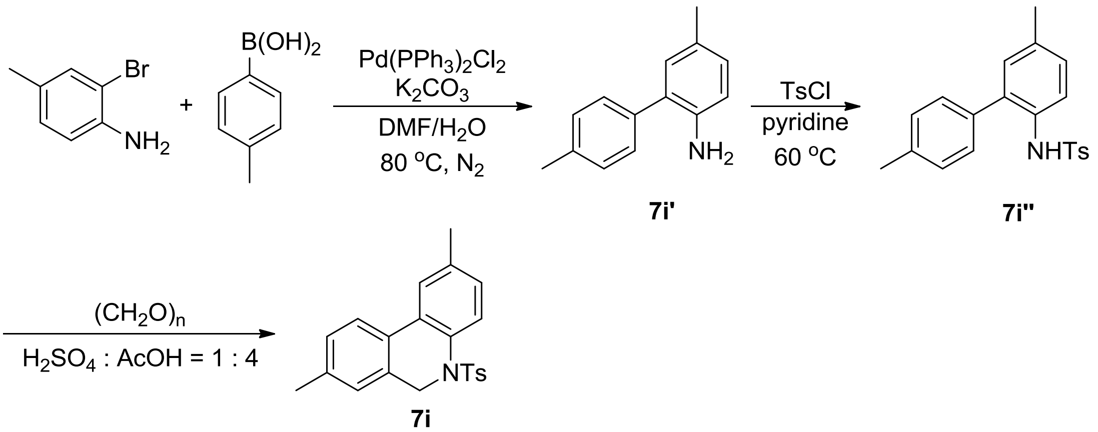

2,8-Dimethyl-5-tosyl-5,6-dihydrophenanthridine (7i):

To a mixture of 2-bromo-4-methylaniline (930 mg, 5 mmol), *p*-tolylboronic acid (748 mg, 5.5 mmol), $Pd(PPh_3)_2Cl_2$ (70.1 mg, 0.1 mmol), K_2CO_3 (2.76 g, 20 mmol) was added water (10 mL) and DMF (40 mL), then reduced pressure and backfilled with N_2. After stirred at 80 °C for 2 h, the mixture was cooled down to room temperature and filtered on diatomite. The filtrate was washed with water (2 × 15 mL) and brine (20 mL), and dried over Na_2SO_4. The resulting solution was concentrated under reduced pressure. The residue was purified by flash column chromatography on silica gel (petroleum ether/ethyl acetate = 50 : 1) to give **7i'** (838.8 mg, 85%). To a solution of **7i'** (838.8 mg, 4.25 mmol) in pyridine (10 mL) was added TsCl (969 mg, 5.1 mmol) portionwise. After stirred at 60 °C for 12 h, pyridine was removed under vacuum. To this residue was added dichloromethane (20 mL) and 2 M HCl (20 mL). After stirred for 10 min, the mixture was extracted by dichloromethane (2 × 15 mL). The combined organic phase was washed with saturated Na_2CO_3 solution (15 mL) and brine (15 mL), and then dried over Na_2SO_4. The resulting solution was concentrated under reduced pressure to give crude **7i"** (1.36 g, 91%) without further purification.

To a mixture of **7i"** (351.5 mg, 1 mmol) and $(HCHO)_n$ (90 mg, 3 mmol) was added H_2SO_4/AcOH = 1 : 10 (10 mL). After stirred at room temperature for 5 h, the reaction was quenched with water (20 mL), then filtered and washed with water for several times. The residue was collected and purified by flash column chromatography on basic alumina (petroleum ether/ethyl acetate/dichloromethane = 20 : 1 : 2) to give product **7i** (312.7 mg, 86%) as a white solid. M.p. 183-185 °C; IR (KBr, cm^{-1}): 3033.5, 2918.5, 2859.4, 1909.5, 1645.3, 1483.0, 1343.6, 1281.9, 1159.0; ^1H NMR ($CDCl_3$, 500 MHz): δ 7.65 (d, J = 8.2 Hz, 1H), 7.34 (d, J = 1.2 Hz, 1H), 7.15 (dd, J = 8.1, 1.4 Hz, 1H), 7.10 (d, J = 8.0 Hz, 1H), 6.97 (d, J = 8.3 Hz, 2H), 6.89 (d, J = 7.9 Hz, 1H), 6.84 (s, 1H), 6.70 (d, J = 8.0 Hz, 2H), 4.76 (s, 2H), 2.40 (s, 3H), 2.29 (s, 3H), 2.15 (s, 3H); ^{13}C NMR ($CDCl_3$, 125 MHz): δ 142.84, 137.72, 137.19, 134.88, 133.32, 131.33, 130.51, 128.80, 128.51, 128.33, 128.20, 127.93, 127.25, 126.78, 124.05, 122.94, 50.04, 21.47, 21.37, 21.12; EI-MS m/z (%): 208.1 (100), 363.1 (22) $[M]^+$; HRMS (EI) m/z calcd for $C_{22}H_{21}NO_2S$ $[M]^+$ 363.1293, found 363.1295.

C1 Functionalization of 5,6-Dihydrophenanthridines, Related to Figure 5.

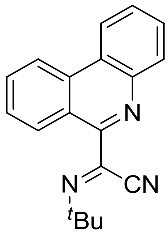

(*Z*)-*N*-(*tert*-Butyl)phenanthridine-6-carbimidoyl cyanide (8a):

Following the general procedure for **6a**, the reaction of **7a** (100.6 mg, 0.3 mmol), DDQ (204.3 mg, 0.9 mmol), AgOTf (11.6 mg, 15 mol%) and ^{t}BuNC (134 μL, 1.2 mmol) in PhCl (4.5 mL) at 80 °C for 3 h afforded the desired product **8a** as a white solid (58.6 mg, 68%). M.p. 104-105 °C; IR (KBr, cm^{-1}): 3075, 2975, 2217, 1612, 1450, 1362, 1207, 937; ^{1}H NMR (CDCl$_3$, 500 MHz): *δ* 9.10 (d, *J* = 8.4 Hz, 1H), 8.69 (d, *J* = 8.3 Hz, 1H), 8.60 (d, *J* = 7.8 Hz, 1H), 8.31 (d, *J* = 7.8 Hz, 1H), 7.89 (t, *J* = 7.4 Hz, 1H), 7.83-7.74 (m, 2H), 7.72 (t, *J* = 7.4 Hz, 1H,), 1.70 (s, 9H); ^{13}C NMR (CDCl$_3$, 125 MHz): *δ* 151.44, 142.74, 139.05, 134.01, 131.09, 131.05, 129.22, 128.97, 128.05, 127.73, 124.81, 123.82, 122.38, 122.12, 112.44, 59.94, 29.41; ESI-MS m/z: 288.1 [M+H]$^+$; HRMS (DART Positive) m/z calcd for $C_{19}H_{18}N_3$ [M+H]$^+$ 288.1495, found 288.1492.

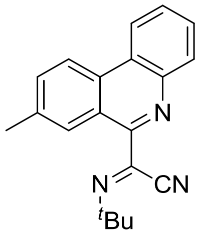

(*Z*)-*N*-(*tert*-Butyl)-8-methylphenanthridine-6-carbimidoyl cyanide (8b):

To a mixture of **7b** (104.7 mg, 0.3 mmol), DDQ (272.4 mg, 1.2 mmol) and AgOTf (11.6 mg, 15 mol%) was added PhCl (3.0 mL) and ^{t}BuNC (168 μL, 1.5 mmol) in glovebox. The reaction was stirred at 80 °C for 3 h under N_2 atmosphere. Upon completion, the reaction mixture was cooled down to room temperature and removed solvent under reduced pressure. Then, purified by column chromatography on basic Al_2O_3 (petroleum ether/ethyl acetate = 30 : 1) to give the desired product **8b** (45.9 mg, 51%) as a white solid. M.p. 159-161 °C; IR (KBr, cm^{-1}): 2965.9, 2208.3, 1954.4, 1741.1, 1621.8, 1568.0, 1459.3, 1366.4, 1234.9; ^{1}H NMR (CDCl$_3$, 500 MHz): *δ* 8.90 (s, 1H), 8.59-8.50 (m, 2H), 8.31-8.25 (m, 1H), 7.80-7.68 (m, 3H), 2.59 (s, 3H), 1.71 (s, 9H); ^{13}C NMR (CDCl$_3$, 125 MHz): *δ* 151.10, 142.48, 139.03 138.06, 132.76, 131.95, 131.02, 128.88, 128.76, 127.18, 124.93, 123.97, 122.28, 121.95, 112.45, 59.94, 29.40, 22.18; EI-MS m/z (%): 245.1 (100), 301.2 (18) [M]$^+$; HRMS (EI) m/z calcd for $C_{20}H_{19}N_3$ [M]$^+$ 301.1579, found 301.1584.

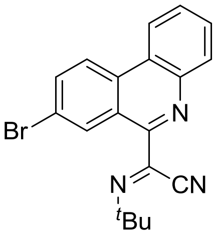

(*Z*)-8-Bromo-*N*-(*tert*-butyl)phenanthridine-6-carbimidoyl cyanide (8c):

Following the general procedure for **6a**, the reaction of **7c** (124.3 mg, 0.3 mmol), DDQ (204.3

mg, 0.9 mmol), AgOTf (11.6 mg, 15 mol%) and ^{t}BuNC (134 μL, 1.2 mmol) in PhCl (4.5 mL) at 80 ^{o}C for 3 h afforded the desired product **8c** as a white solid (34.5 mg, 31%). M.p. 172-175 ^{o}C; IR (KBr, cm^{-1}): 2967.0, 2931.7, 2220.7, 1614.3, 1692.6, 1616.6, 1566.5; ^{1}H NMR (CDCl$_3$, 500 MHz): *δ* 9.47 (d, *J* = 2.0 Hz, 1H), 8.56-8.47 (m, 2H), 8.30 (dd, *J* = 8.2, 1.2 Hz, 1H), 7.95 (dd, *J* = 8.8, 2.0 Hz, 1H), 7.84-7.74 (m, 2H), 1.71 (s, 9H); ^{13}C NMR (CDCl$_3$, 125 MHz): *δ* 149.67, 142.66, 139.10, 134.17, 132.64, 131.33, 130.68, 129.65, 129.52, 124.96, 124.32, 124.08, 122.43, 121.97, 112.08, 60.13, 29.34; ESI-MS m/z (%): 366.1 [M (^{79}Br)+H]$^+$ (81), 368.1 [M (^{81}Br)+H]$^+$ (100); HRMS (DART Positive) m/z calcd for $C_{19}H_{17}N_3Br$ [M+H]$^+$ 366.0600, found 366.0601.

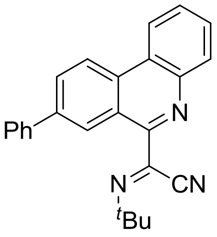

(*Z*)-*N*-(*tert*-Butyl)-3,8-dimethylphenanthridine-6-carbimidoyl cyanide (8d):
Following the general procedure for **8b**, the reaction of **7d** (123.45 mg, 0.3 mmol), DDQ (272.4 mg, 1.2 mmol), AgOTf (11.6 mg, 15 mol%) and ^{t}BuNC (168 μL, 1.5 mmol) in PhCl (3 mL) at 80 ^{o}C for 3 h afforded the desired product **8d** as a white solid (38.5 mg, 36%). M.p. 112-115 ^{o}C; IR (KBr, cm^{-1}): 2965.7, 2859.4, 2216.6, 1608.8, 1463.4, 1395.9; ^{1}H NMR (CDCl$_3$, 500 MHz): *δ* 9.55 (d, *J* = 1.3 Hz, 1H), 8.70 (d, *J* = 8.7 Hz, 1H), 8.58 (d, *J* = 7.7 Hz, 1H), 8.31 (d, *J* = 7.5 Hz, 1H), 8.13 (dd, *J* = 8.6, 1.5 Hz, 1H), 7.82-7.72 (m, 4H), 7.54 (t, *J* = 7.7 Hz, 2H), 7.44 (t, *J* = 7.4 Hz, 1H), 1.73 (s, 9H); ^{13}C NMR (CDCl$_3$, 125 MHz): *δ* 151.15, 142.71, 140.56, 140.41, 139.40, 132.97, 131.16, 129.97, 129.26, 129.18, 129.14, 128.07, 127.38, 126.05, 124.67, 124.27, 122.94, 122.14, 112.32, 59.90, 29.43; EI-MS m/z (%): 307.1 (100), 363.2 (33) [M]$^+$; HRMS (EI) m/z calcd for $C_{25}H_{21}N_3$ [M]$^+$ 363.1735, found 363.1736.

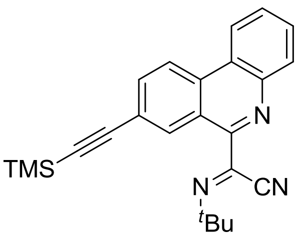

(*Z*)-*N*-(*tert*-Butyl)-8-((trimethylsilyl)ethynyl)phenanthridine-6-carbimidoyl cyanide (8e):
Following the general procedure for **8b**, the reaction of **7e** (123.45 mg, 0.3 mmol), DDQ (272.4 mg, 1.2 mmol), AgOTf (11.6 mg, 15 mol%) and ^{t}BuNC (168 μL, 1.5 mmol) in PhCl (3 mL) at 80 ^{o}C for 3 h afforded the desired product **8e** as a white solid (36.8 mg, 32%). M.p. 135-139 ^{o}C; IR (KBr, cm^{-1}): 2966.6, 2150.3, 1690.5, 1647.2, 1619.5, 1465.2, 1363.4; ^{1}H NMR (CDCl$_3$, 500 MHz): *δ* 9.36 (d, *J* = 1.4 Hz, 1H), 8.59 (d, *J* = 8.6 Hz, 1H), 8.55 (d, *J* = 7.9 Hz, 1H), 8.30 (dd, *J* = 8.2, 1.3 Hz, 1H), 7.89 (dd, *J* = 8.6, 1.7 Hz, 1H), 7.84-7.72 (m, 2H), 1.71 (s, 9H), 0.30 (s, 9H); ^{13}C NMR (CDCl$_3$, 125 MHz): *δ* 150.40, 142.88, 138.78, 133.38, 133.35, 132.13, 131.10, 129.51, 129.15, 124.33, 123.43, 122.86, 122.25, 122.21, 112.05, 104.63, 96.30, 59.96, 29.17, -0.11; EI-MS m/z (%): 327.1 (100), 383.2 (30) [M]$^+$; HRMS (EI) m/z calcd for $C_{24}H_{25}N_3Si$ [M]$^+$ 383.1818, found 383.1815.

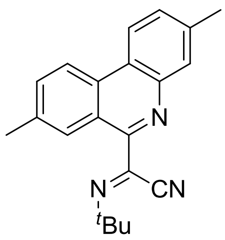

(*Z*)-*N*-(*tert*-Butyl)-3,8-dimethylphenanthridine-6-carbimidoyl cyanide (8f):
Following the general procedure for **8b**, the reaction of **7f** (108.9 mg, 0.3 mmol), DDQ (272.4 mg, 1.2 mmol), AgOTf (11.6 mg, 15 mol%) and ^{t}BuNC (168 μL, 1.5 mmol) in PhCl (3 mL) at 80 ^{o}C for 3 h afforded the desired product **8f** as a white solid (46.2 mg, 49%). M.p. 159-161 ^{o}C; IR (KBr, cm^{-1}): 2970.1, 2909.2, 2212.4, 1619.9, 1565.2, 1470.6; ^{1}H NMR ($CDCl_3$, 500 MHz): *δ* 8.89 (s, 1H), 8.52 (d, *J* = 8.5 Hz, 1H), 8.43 (d, *J* = 8.4 Hz, 1H), 8.09 (s, 1H), 7.68 (dd, *J* = 8.5, 1.6 Hz, 1H), 7.56 (dd, *J* = 8.4, 1.6 Hz, 1H), 2.60 (s, 3H), 2.57 (s, 3H), 1.70 (s, 9H); ^{13}C NMR ($CDCl_3$, 125 MHz): *δ* 151.06, 142.64, 139.17, 138.98, 137.53, 132.69, 132.04, 130.72, 130.46, 127.11, 123.70, 122.64, 122.10, 121.73, 112.50, 59.87, 29.41, 22.16, 21.55; EI-MS m/z (%): 259.1 (100), 315.2 (36) [M]$^+$; HRMS (EI) m/z calcd for $C_{21}H_{21}N_3$ [M]$^+$ 315.1735, found 315.1738.

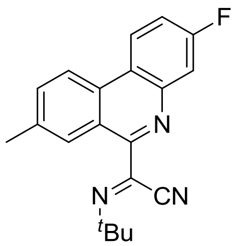

(*Z*)-*N*-(*tert*-Butyl)-3-fluoro-8-methylphenanthridine-6-carbimidoyl cyanide (8g):
Following the general procedure for **6a**, the reaction of **7g** (110.2 mg, 0.3 mmol), DDQ (204.3 mg, 0.9 mmol), AgOTf (11.6 mg, 15 mol%) and ^{t}BuNC (134 μL, 1.2 mmol) in PhCl (4.5 mL) at 80 ^{o}C for 3 h afforded the desired product **8g** as a white solid (44.3 mg, 46%). M.p. 162-164 ^{o}C; IR (KBr, cm^{-1}): 2966.8, 2927.6, 2220.2, 1620.7, 1575.9, 1472.7; ^{1}H NMR ($CDCl_3$, 500 MHz): *δ* 8.82 (s, 1H), 8.48 (dd, *J* = 9.1, 5.7 Hz, 1H), 8.44 (d, *J* = 8.4 Hz, 1H), 7.90 (dd, *J* = 9.5, 2.5 Hz, 1H), 7.68 (d, *J* = 8.3 Hz, 1H), 7.46 (td, *J* = 8.2, 1.8 Hz, 1H), 2.57 (s, 3H), 1.71 (s, 9H); ^{19}F NMR ($CDCl_3$, 470 MHz): *δ* -111.95 (m, Ar-F); ^{13}C NMR ($CDCl_3$, 125 MHz): *δ* 162.59 (d, $^1J_{C-F}$ = 249.0 Hz), 152.24, 143.55 (d, $^3J_{C-F}$ = 12.0 Hz), 138.67, 137.89, 133.14, 131.78, 127.22, 123.89 (d, $^3J_{C-F}$ = 9.3 Hz), 123.48, 122.03, 121.60 (d, $^4J_{C-F}$ = 2.2 Hz), 117.98 (d, $^2J_{C-F}$ = 24.0 Hz), 115.17 (d, $^2J_{C-F}$ = 20.7 Hz), 112.30, 60.09, 29.38, 22.12; EI-MS m/z (%): 263.1 (100), 319.2 (21) [M]$^+$; HRMS (EI) m/z calcd for $C_{20}H_{18}N_3F$ [M]$^+$ 319.1485, found 319.1484.

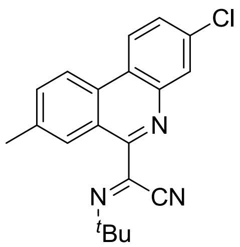

(*Z*)-*N*-(*tert*-Butyl)-3-chloro-8-methylphenanthridine-6-carbimidoyl cyanide (8h):
Following the general procedure for **6a**, the reaction of **7h** (115.2 mg, 0.3 mmol), DDQ (204.3 mg, 0.9 mmol), AgOTf (11.6 mg, 15 mol%) and ^{t}BuNC (134 μL, 1.2 mmol) in PhCl (4.5 mL) at

80 °C for 3 h afforded the desired product **8h** as a white solid (46.6 mg, 46%). M.p. 207-209 °C; IR (KBr, cm^{-1}): 2966.4, 2923.7, 2218.4, 1614.3, 1466.8, 1365.1; ^{1}H NMR ($CDCl_3$, 500 MHz): δ 8.82 (s, 1H), 8.40 (d, *J* = 8.5 Hz, 1H), 8.36 (d, *J* = 8.8 Hz, 1H), 8.20 (d, *J* =2.0 Hz, 1H), 7.67 (dd, *J* = 8.4, 1.0 Hz, 1H), 7.61 (dd, *J* = 8.8, 2.1 Hz, 1H), 2.57 (s, 3H), 1.71 (s, 9H); ^{13}C NMR ($CDCl_3$, 125 MHz): δ 152.00, 142.87, 138.68, 138.39, 134.35, 133.10, 131.44, 129.87, 129.24, 127.26, 123.75, 123.25, 123.24, 122.09, 112.25, 60.08, 29.36, 22.17; EI-MS m/z (%): 279.1 (100), 337.1 (7) [M (^{37}Cl)]$^+$, 335.1 (21) [M (^{35}Cl)]$^+$; HRMS (EI) m/z calcd for $C_{20}H_{18}N_3Cl$ [M]$^+$ 335.1189, found 335.1183.

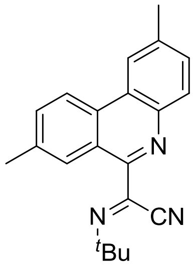

(*Z*)-*N*-(*tert*-Butyl)-2,8-dimethylphenanthridine-6-carbimidoyl cyanide (8i):
Following the general procedure for **8b**, the reaction of **7h** (108.9 mg, 0.3 mmol), DDQ (272.4 mg, 1.2 mmol), AgOTf (11.6 mg, 15 mol%) and ^{t}BuNC (168 μL, 1.5 mmol) in PhCl (3 mL) at 80 °C for 3 h afforded the desired product **8h** as a white solid (29.1 mg, 31%). M.p. 139-140 °C; IR (KBr, cm^{-1}): 2970.6, 2915.2, 2212.4, 1899.3, 1743.5, 1609.2, 1567.8, 1462.8, 1233.3, 1200.3; ^{1}H NMR ($CDCl_3$, 500 MHz): δ 8.91 (s, 1H), 8.52 (d, *J* = 8.5 Hz, 1H), 8.30 (s, 1H), 8.16 (d, *J* = 8.3 Hz, 1H), 7.67 (d, *J* = 8.4 Hz, 1H), 7.57 (d, *J* = 8.3 Hz, 1H), 2.64 (s, 3H), 2.57 (s, 3H), 1.71 (s, 9H); ^{13}C NMR ($CDCl_3$, 125 MHz): δ 150.13, 140.85, 139.22, 139.14, 137.87, 132.48, 131.60, 130.75, 130.55, 127.10, 124.77, 124.06, 122.22, 121.55, 112.51, 59.80, 29.40, 22.35, 22.17; EI-MS m/z (%): 259.1 (100), 315.2 (38) [M]$^+$; HRMS (EI) m/z calcd for $C_{21}H_{21}N_3$ [M]$^+$ 315.1735, found 315.1728.

Synthetic applications of the α-Iminonitrile-decorated Isochromans, Related to Figure 6.

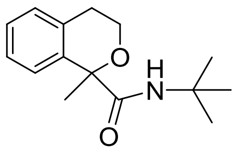

***N*-*tert*-Butyl-1-methylisochroman-1-carboxamide (9a):**
To a sealed tube containing Al_2O_3 (1.0 g) was added (*E*)-*N*-(*tert*-butyl)-1-methyl-isochroman-1-carbimidoyl cyanide **4l** (51.3 mg, 0.2 mmol) in toluene (2.0 mL) and the mixture was stirred at 150 °C for 25 h. Upon completion, the reaction mixture was cooled down to room temperature and diluted with ethyl acetate. After filtration through a thin pad of celite, the solid was repeatedly rinsed with ethyl acetate (3 × 10 mL). Then the combined organic phase was evaporated in vacuum to give the crude product which was purified by column chromatography on silica gel to give product **9a** (26.0 mg, 53%) as white solid. M.p. 52-54 °C; IR (KBr, cm^{-1}): 3364, 2977, 2931, 1665, 1510, 1450, 1363, 1286, 1235, 1109, 1041, 976, 745, 653; ^{1}H NMR ($CDCl_3$, 500 MHz): δ 7.67 (d, *J* = 9.0 Hz, 1H), 7.22-7.15 (m, 2H), 7.06 (d, *J* = 7.0 Hz, 1H), 6.72 (s, 1H), 3.98 (t, *J* = 5.7 Hz, 1H), 2.92-2.86 (m, 1H), 2.84-2.79 (m, 1H), 1.68 (s,

3H), 1.31 (s, 9H); ^{13}C NMR ($CDCl_3$, 125 MHz): 172.8, 136.8, 132.2, 128.3, 127.6, 126.8, 126.2, 79.1, 61.2, 50.6, 29.1, 28.6, 27.0; LC-MS (ESI) m/z 248 $[M+H]^+$; HRMS (ESI) m/z calcd for $C_{15}H_{22}O_2N$ 248.1645 $[M+H]^+$, found 248.1644.

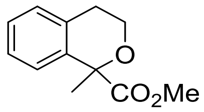

Methyl 1-methylisochroman-1-carboxylate (9b):

To a test tube containing (*E*)-*N*-(*tert*-butyl)- 1-methylisochroman-1-carbimidoyl cyanide **4l** (51.3 mg, 0.2 mmol) in MeOH (3.0 mL) was added 1M HCl (0.6 mL) and the mixture was stirred at room temperature for 10 h. Then water (30 mL) was added and the solution was extracted with ethyl acetate (3 × 10 mL). The combined organic phase was washed with brine and dried over Na_2SO_4. After that, the filtrate was evaporated in vacuum to give the crude product which was purified by column chromatography on silica gel to give **9b** (30.1 mg, 73%) as pale yellow oil. IR (KBr, cm^{-1}): 2949, 1738, 1446, 1250, 1117, 977, 742; ^1H NMR ($CDCl_3$, 500 MHz): δ 7.43-7.41 (m, 1H), 7.22-7.20 (m, 2H), 7.12-7.10 (m, 1H), 4.15-4.07 (m, 2H), 3.74 (s, 3H), 3.04-2.97 (m, 1H), 2.74-2.69 (m, 1H), 1.74 (s, 3H); ^{13}C NMR ($CDCl_3$, 125 MHz): 174.1, 136.2, 133.5, 128.7, 127.1, 126.8, 126.3, 78.3, 62.1, 52.5, 28.7, 27.9; LC-MS (ESI) m/z 224 $[M+NH_4]^+$; HRMS (ESI) m/z calcd for $C_{12}H_{15}O_3$ $[M+H]^+$ 207.1016, found 207.1015.

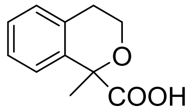

Methylisochroman-1-carboxylic acid (9c):

(*E*)-*N*-(*tert*-butyl)-1-methylisochroman-1- carbimidoyl cyanide **4l** (51.3 mg, 0.2 mmol) was subjected to hydrolysis in aqueous CH_3CN (80% v/v, 50 mL) containing 0.1 N HCl at room temperature for 2.5 h. Upon completion, water (50 mL) was added and the solution was extracted with dichloromethane (3 × 10 mL). The combined organic phase was washed with brine and dried over Na_2SO_4. After that, the filtrate was evaporated in vacuum to give the crude product which was purified by column chromatography on silica gel to give **9c** (34.1 mg, 89%) as pale yellow oil. IR (KBr, cm^{-1}): 2933, 2631, 1711, 1449, 1373, 1286, 1217, 1117, 740, 652; ^1H NMR ($CDCl_3$, 500 MHz): δ 7.55-7.54 (m, 1H), 7.23-7.22 (m, 2H), 7.11-7.09 (m, 1H), 4.17-4.13 (m, 1H), 4.08-4.03 (m, 1H), 2.93-2.83 (m, 2H), 1.78 (s, 3H); ^{13}C NMR ($CDCl_3$, 125 MHz): 176.7, 135.0, 133.1, 128.7, 127.5, 127.0, 126.6, 78.3, 61.9, 28.7, 27.1; LC-MS (ESI) m/z 210 $[M+NH_4]^+$; HRMS (ESI) m/z calcd for $C_{11}H_{16}O_3N$ $[M+NH_4]^+$ 210.1125, found 210.1124.

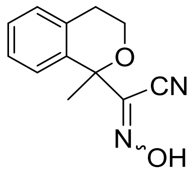

***N*-Hydroxy-1-methylisochroman-1-carbimidoyl cyanide (9d):**

To a sealed tube containing $NH_2OH{\cdot}HCl$ (16.7 mg, 0.24 mmol) and K_2CO_3 (41.5 mg, 0.3 mmol) was added (*E*)-*N*-(*tert*- butyl)-1-methylisochroman-1-carbimidoyl cyanide **4l** (51.3 mg, 0.2 mmol) in EtOH (3.0 mL). The mixture was stirred at 100 ^oC for 4 h. Upon completion, the reaction mixture was cooled down to room temperature and diluted with ethyl acetate. After

filtration through a thin pad of celite, the solid was repeatedly rinsed with ethyl acetate (3 × 10 mL). Then the combined organic phase was evaporated in vacuum to give the crude product which was purified by column chromatography on silica gel to give product **9d** (31.6 mg, 73%) as colorless oil. IR (KBr, cm^{-1}): 3133, 2988, 2865, 1619, 1482, 1453, 1375, 1286, 1091, 994, 754, 665; ^{1}H NMR (d$_6$-DMSO, 500 MHz): *δ* 13.41 (s, 1H), 7.23-7.17 (m, 3H), 7.13-7.11 (m, 1H), 4.00-3.96 (m, 1H), 3.81-3.77 (m, 1H), 2.88-2.83 (m, 1H), 2.76 (dt, *J* = 16.5, 9.5 Hz, 1H), 1.72 (s, 3H); ^{13}C NMR (d$_6$-DMSO, 125 MHz): 137.1, 135.9, 134.0, 129.4, 127.8, 127.3, 126.6, 110.4, 76.7, 60.3, 28.4, 26.4; LC-MS (ESI) m/z 234 [M+NH$_4$]$^+$; HRMS (ESI) m/z calcd for $C_{12}H_{16}O_2N_3$ [M+NH$_4$]$^+$ 234.1237, found 234.1235.

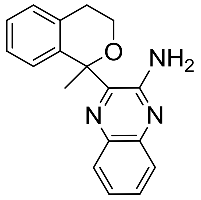

3-(1-Methylisochroman-1-yl)quinoxalin-2-amine (9e):

To a test tube containing benzene-1,2-diamine (26.0 mg, 0.24 mmol) and NaOAc (19.7 mg, 0.24 mmol), (*E*)-*N*-(*tert*-butyl)-1-methylisochroman-1-carbimidoyl cyanide **4l** (51.3 mg, 0.2 mmol) in AcOH (2.0 mL) was added. The mixture was stirred at 120 ^{o}C for 7.5 h. Upon completion, the reaction mixture was poured into water (50 mL) and extracted with dichloromethane (3 × 10 mL). The combined organic phase was dried over anhydrous Na_2SO_4 and evaporated in vacuum to give the crude product which was purified by column chromatography on silica gel to give product **9e** (30.3 mg, 52%) as yellow solid. M.p. 155-157 ^{o}C; IR (KBr, cm^{-1}): 3454, 2929, 1727, 1626, 1423, 1365, 1274, 1101, 1038, 756; ^{1}H NMR (CDCl$_3$, 500 MHz): *δ* 7.88 (d, *J* = 8.1 Hz, 1H), 7.60-7.53 (m, 2H), 7.40-7.37 (m, 1H), 7.21-7.15 (m, 2H), 7.11 (t, *J* = 7.4 Hz, 1H), 7.01 (d, *J* = 7.8 Hz, 1H), 5.89 (s, 2H), 4.31-4.25 (m, 1H), 4.14-4.10 (m, 1H), 3.14-3.08 (m, 1H), 2.96 (dt, *J* = 16.7, 4.3 Hz, 1H), 2.08 (s, 3H); ^{13}C NMR (CDCl$_3$, 125 MHz): 150.7, 147.9, 140.9, 138.0, 136.4, 132.1, 129.9, 129.2, 128.8, 127.5, 126.9, 126.1, 124.9, 124.5, 81.6, 60.6, 28.5, 26.2; EI-MS m/z (%): 291 (30) [M$^+$], 263 (27), 147 (100), 129 (20); HRMS (EI) m/z calcd for $C_{18}H_{17}N_3O$ [M]$^+$ 291.1372, found 291.1370.

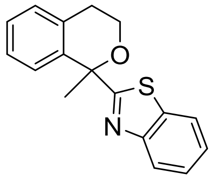

2-(1-Methylisochroman-1-yl)benzo[*d*]thiazole (9f):

Following the above procedure as for **5e**, the reaction mixture of (*E*)-*N*-(*tert*-butyl)-1-methylisochroman-1-carbimidoyl cyanide **4l** (51.3 mg, 0.2 mmol), 2-aminobenzenethiol (30.0 mg, 0.24 mmol), and NaOAc (19.7 mg, 0.24 mmol) in AcOH (2.0 mL) was stirred at 120 ^{o}C for 3 h to afford product **9f** (22.9 mg, 41%) as white solid. M.p. 96-98 ^{o}C; IR (KBr, cm^{-1}): 2978, 2919, 1935, 1735, 1513, 1481, 1440, 1365, 1274, 1202, 1110, 1012, 762, 723; ^{1}H NMR (CDCl$_3$, 500 MHz): *δ* 8.03 (d, *J* = 8.0 Hz, 1H), 7.82 (d, *J* = 8.0 Hz, 1H), 7.64 (d, *J* = 7.0 Hz, 1H), 7.44 (t, *J* = 7.5 Hz, 1H), 7.33 (t, *J* = 7.5 Hz, 1H), 7.23-7.19 (m, 2H), 7.13 (d, *J* = 7.0 Hz, 1H), 4.13 (t, *J* = 5.5 Hz, 2H), 2.96 (t, *J* = 5.5 Hz, 2H), 2.08 (s, 3H); ^{13}C NMR (CDCl$_3$, 125 MHz): 178.3, 153.4,

138.1, 135.5, 132.9, 128.7, 127.6, 127.1, 126.3, 125.7, 124.8, 123.3, 121.5, 78.9, 61.3, 29.6, 29.1; LC-MS (ESI) m/z 282 [M+H]$^+$; HRMS (ESI) m/z calcd for $C_{17}H_{16}ONS$ [M+H]$^+$ 282.0947, found 282.0946.

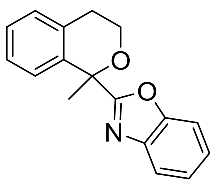

2-(1-Methylisochroman-1-yl)benzo[*d*]oxazole (9g):

Following the above procedure as for **5e**, the reaction mixture of (*E*)-*N*-(*tert*-butyl)-1-methyl-isochroman-1-carbimidoyl cyanide **4l** (51.3 mg, 0.2 mmol), 2-aminophenol (26.2 mg, 0.24 mmol), and NaOAc (19.7 mg, 0.24 mmol) in AcOH (4.0 mL) was stirred at 120 °C for 4 h to afford product **9g** (29.4 mg, 55%) as pale yellow oil. IR (KBr, cm^{-1}): 2987, 2934, 1738, 1556, 1451, 1370, 1282, 1242, 1104, 936, 841, 744; ^{1}H NMR ($CDCl_3$, 500 MHz): δ 7.75-7.74 (m, 1H), 7.52-7.50 (m, 1H), 7.33-7.30 (m, 3H), 7.25-7.19 (m, 3H), 4.18-4.15 (m, 2H), 3.10-3.08 (m, 1H), 2.88-2.84 (m, 1H), 2.07 (s, 3H); ^{13}C NMR ($CDCl_3$, 125 MHz): 168.0, 150.9, 140.7, 136.8, 133.4, 129.1, 127.4, 126.8, 126.4, 125.2, 124.3, 120.4, 110.9, 75.3, 61.8, 28.8, 28.4; LC-MS (ESI) m/z 266 [M+H]$^+$; HRMS (ESI) m/z calcd for $C_{17}H_{16}O_2N$ [M+H]$^+$ 266.1176, found 266.1174.

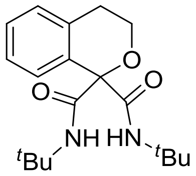

***N,N'*-di-*tert*-butylisochroman-1,1-dicarboxamide (9h):**

To a sealed tube containing $Pd(OAc)_2$ (2.3 mg, 0.01 mmol), $Cu(TFA)_2$ (202.7 mg, 0.7 mmol) and (1*E*,1*E*)-*N,N'*-di-*tert*-butyl- isochroman-1,1-bis(carbimidoyl) cyanide **2a** (84.1 mg, 0.24 mmol), 2-phenylpyridine (31.0 mg, 0.2 mmol) in THF (2.0 mL) was added. The mixture was stirred at 120 °C for 24 h. Upon completion, the reaction mixture was cooled down to room temperature and was purified by column chromatography on silica gel to give product **9h** (67.1 mg, 84%) as white solid, together with **5i** (26.3 mg, 73%) as pale yellow solid. M.p. 139-141 °C; IR (KBr, cm^{-1}): 3352, 2971, 1693, 1517, 1452, 1362, 1223, 1116, 1036, 746, 646; ^{1}H NMR ($CDCl_3$, 500 MHz): δ 7.90-7.88 (m, 1H), 7.23-7.21 (m, 2H), 7.09-7.08 (m, 1H), 7.05 (s, 2H), 4.24 (t, J = 5.5 Hz, 2H), 2.87 (t, J = 5.5 Hz, 2H), 1.31 (s, 18H); ^{13}C NMR ($CDCl_3$, 125 MHz): 168.5, 133.0, 131.3, 128.6, 127.6, 127.5, 126.3, 81.4, 63.2, 51.2, 28.6, 28.5; LC-MS (DART) m/z 333 [M+H]$^+$; HRMS (DART) m/z calcd for $C_{19}H_{29}O_3N_2$ [M+H]$^+$ 333.2173, found 333.2171.

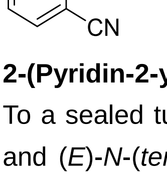

2-(Pyridin-2-yl)benzonitrile (9i) (Xu et al., 2012):

To a sealed tube containing $Pd(OAc)_2$ (2.3 mg, 0.01 mmol), $Cu(TFA)_2$ (115.8 mg, 0.4 mmol) and (*E*)-*N*-(*tert*-butyl)-1-methyl- isochroman-1-carbimidoyl cyanide **4l** (61.5 mg, 0.24 mmol), 2-phenylpyridine (31.1 mg, 0.2 mmol) in THF (1.0 mL) was added. The mixture was stirred at

120 ^{o}C for 23 h. Upon completion, the reaction mixture was cooled down to room temperature and was purified by column chromatography on silica gel to give product **9i** (27.1 mg, 75%) as pale yellow solid, together with **5a** (33.2 mg, 56%) as white solid. M.p. 42-43 ^{o}C; IR (KBr, cm^{-1}): 3062, 2923, 2856, 2224, 1956, 1579, 1460, 1432, 1300, 1155, 1100, 760; ^{1}H NMR ($CDCl_3$, 500 MHz): δ 8.75 (d, J = 4.5 Hz, 1H), 7.82-7.74 (m, 4H), 7.66 (t, J = 7.5 Hz, 1H), 7.47 (t, J = 7.5 Hz, 1H), 7.33-7.31 (m, 1H); ^{13}C NMR ($CDCl_3$, 125 MHz): 155.2, 149.9, 143.4, 136.8, 134.1, 132.8, 129.9, 128.7, 123.3, 123.2, 118.7, 111.0; LC-MS (ESI) m/z 181 [M+H]$^{+}$.

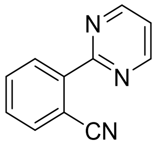

2-(Pyrimidin-2-yl)benzonitrile (9j) (Xu et al., 2012):
Following the above procedure as for **5i** and **5h**, the reaction mixture of $Pd(OAc)_2$ (2.3 mg, 0.01 mmol), $Cu(TFA)_2$ (202.7 mg, 0.7 mmol), (1*E*,1*E*)-*N,N'*-di-*tert*-butylisochroman-1,1-bis-(carbimidoyl) cyanide **2a** (84.1 mg, 0.24 mmol) and 2-phenyl-pyrimidine (31.2 mg, 0.2 mmol) in THF (2.0 mL) was stirred at 120 ^{o}C for 22 h to afford product **9j** (26.7 mg, 67%) as white solid, together with **9h** (75.4 mg, 94%) as white solid. M.p. 140-141 ^{o}C; IR (KBr, cm^{-1}): 3422, 3039, 2922, 2220, 1644, 1555, 1412, 1365, 757; ^{1}H NMR ($CDCl_3$, 500 MHz): δ 8.91 (d, J = 4.5 Hz, 2H), 8.35 (dd, J = 8.0, 0.5 Hz, 1H), 7.84 (dd, J = 7.5, 1.0 Hz, 1H), 7.70 (td, J = 7.5, 1.0 Hz, 1H), 7.56 (td, J = 8.0, 1.5 Hz, 1H), 7.32 (t, J = 5.0 Hz, 1H); ^{13}C NMR ($CDCl_3$, 125 MHz): 162.8, 157.3, 140.3, 135.0, 132.5, 130.4, 130.2, 120.1, 118.9, 111.8; EI-MS m/z (%): 181 (100) [M]$^{+}$, 128 (96).

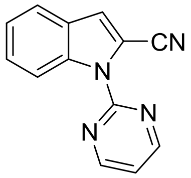

1-(Pyrimidin-2-yl)-1*H*-indole-2-carbonitrile (9k) (Xu et al., 2012):
Following the above procedure as for **5i** and **5h**, the reaction mixture of $Pd(OAc)_2$ (2.3 mg, 0.01 mmol), $Cu(TFA)_2$ (202.7 mg, 0.7 mmol), (1*E*,1*E*)-*N,N'*-di-*tert*-butylisochroman-1,1-bis-(carbimidoyl) cyanide **2a** (84.1 mg, 0.24 mmol) and 1-(pyrimidin-2-yl)-1*H*-indole (39.0 mg, 0.2 mmol) in THF (2.0 mL) was stirred at 120 ^{o}C for 23 h to afford product **9k** (22.1 mg, 50%) as white solid, together with **9h** (66.4 mg, 83%) as white solid. M.p. 124-125 ^{o}C; IR (KBr, cm^{-1}): 3436, 3104, 3036, 2360, 1571, 1439, 1338, 1254, 813, 735; ^{1}H NMR ($CDCl_3$, 500 MHz): δ 8.83 (d, J = 4.5 Hz, 2H), 8.69 (dd, J = 8.5, 0.5 Hz, 1H), 7.68 (d, J = 8.0 Hz, 1H), 7.52-7.48 (m, 1H), 7.47 (d, J = 0.5 Hz, 1H), 7.34-7.31 (m, 1H), 7.23 (t, J = 4.7 Hz, 1H); ^{13}C NMR ($CDCl_3$, 125 MHz): 158.3, 156.5, 136.6, 127.7, 127.5, 123.5, 122.0, 120.9, 117.9, 116.1, 114.2, 108.9; EI-MS m/z (%): 220 (100) [M]$^{+}$.

Application for the Synthesis of Pyrene-based Materials, Related to Figure 7.

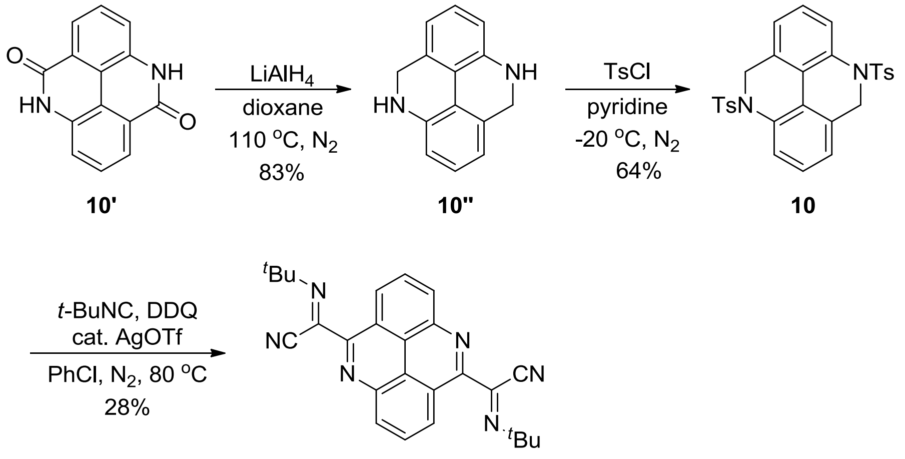

11

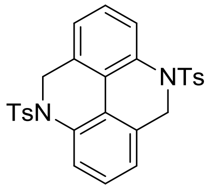

4,9-Ditosyl-4,5,9,10-tetrahydropyrido[2,3,4,5-*lmn*]phenanthridine (10)

To a mixture of $LiAlH_4$ (760 mg, 20 mmol) and anhydrous 1,4-dioxane (20 mL) was added pyrido[2,3,4,5-*imn*]phenanthridine-5,10(4*H*,9*H*)-dione **10'** (472 mg, 2 mmol) (Gawlak adn Robbins, 1964) at 0 °C under a nitrogen atmosphere. The mixture was stirred at 110 °C for 24 h. The reaction was quenched with saturated Na_2SO_4 solution after cooling to room temperature. The mixture was filtered and the residue was washed with dichloromethane (5 × 5 mL). Evaporation of the solvent gave the product **10"** (344.9 mg, 83%) as a yellow solid, which was directly used for the next step without further purification. To a mixture of TsCl (912 mg, 4.8 mmol) and pyridine (8 mL) was added **10"** at 0 °C under a nitrogen atmosphere. After stirred for 5 min, the reaction was transferred to a refrigerator at -20 °C overnight. Pyridine was removed on rotary evaporator, and the residue was dissolved in dichloromethane (15 mL), washed with 2 M HCl (15 mL). The aqueous phase was extracted by dichloromethane (3 × 15 mL). The combined organic phase was washed with saturated Na_2CO_3 (15 mL) solution and brine (15 mL) and dried over Na_2SO_4. The solvent was removed on a rotary evaporator and the residue was recrystallized by dichloromethane/Hexane (below 5 °C) to give the product **10** (527.3 mg, 64%) as a white solid. M.p. 213-215 °C; IR (KBr, cm^{-1}): 2922, 1914, 1599, 1447, 1344, 1294, 1161; ^1H NMR ($CDCl_3$, 500 MHz): δ 7.48 (d, J = 8.0 Hz, 2H), 7.34 (d, J = 8.0 Hz, 4H), 7.19 (t, J = 7.5 Hz, 2H), 7.02 (d, J = 8.0 Hz, 4H), 6.92 (d, J = 7.5 Hz, 2H), 4.74 (s, 4H), 2.30 (s, 6H); ^{13}C NMR ($CDCl_3$, 125 MHz): δ 144.23, 136.06, 134.28, 130.05, 129.46, 128.54, 127.00, 124.01, 122.85, 122.22, 48.89, 21.58; ESI-MS m/z: 534.2 $[M+NH_4]^+$; HRMS (DART Positive) m/z calcd for $C_{28}H_{25}N_2O_4S_2$ $[M+H]^+$ 517.1250, found 517.1250.

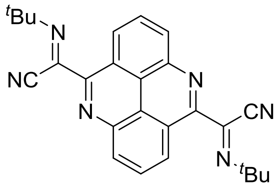

(5*Z*,10*Z*)-*N'*[5],*N'*[10]-di-*tert*-butylpyrido[2,3,4,5-*lmn*]phenanthridine-5,10-bis(carbimidoyl cyanide) (11)

To a mixture of **10** (51.7 mg, 0.1 mmol), DDQ (113.0 mg, 0.5 mmol), AgOTf (3.9 mg, 30 mol%,) and PhCl (1.5 mL) was added ^{t}BuNC (90 uL, 0.8 mmol). The mixture was sealed and stirred at 80 ^{o}C under nitrogen atmosphere; the reaction was cooled to room temperature and the solvent was removed under reduced pressure. The residue was purified by column chromatography on basic Al_2O_3 (petroleum ether/dichloromethane = 2 : 1) to give the product **11** as a yellow solid (11.9 mg, 28%). M.p. >300 ^{o}C; IR (KBr, cm^{-1}): 2965.2, 2926.1, 2858.9, 2235.6, 1831.8, 1696.4, 1636.5, 1463.9; ^{1}H NMR ($CDCl_3$, 500 MHz): δ 9.64 (d, J = 8.0 Hz, 2H), 8.85 (d, J = 7.5 Hz, 2H), 8.34 (t, J = 7.5 Hz, 2H), 1.76 (s, 18H); ^{13}C NMR ($CDCl_3$, 125 MHz): δ 151.78, 140.48, 139.65, 131.76, 129.81, 128.14, 122.33, 122.11, 112.08, 60.28, 29.43; ESI-MS m/z: 421.2 $[M+H]^+$; HRMS (ESI) m/z calcd for $C_{26}H_{25}N_6$ $[M+H]^+$ 421.2135, found 421.2132.

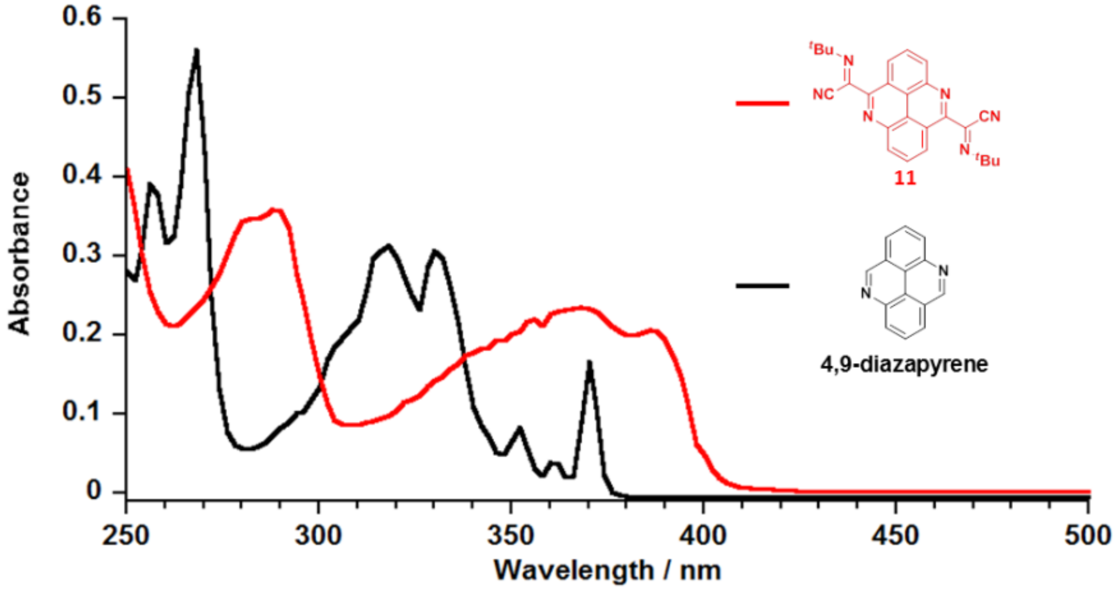

Figure S1. UV-Vis absorption of compound **11** (red curve) and 4,9-diazapyrene (Black curve). c = 5×10^{-5} M in THF. Related to **Figure 7**.

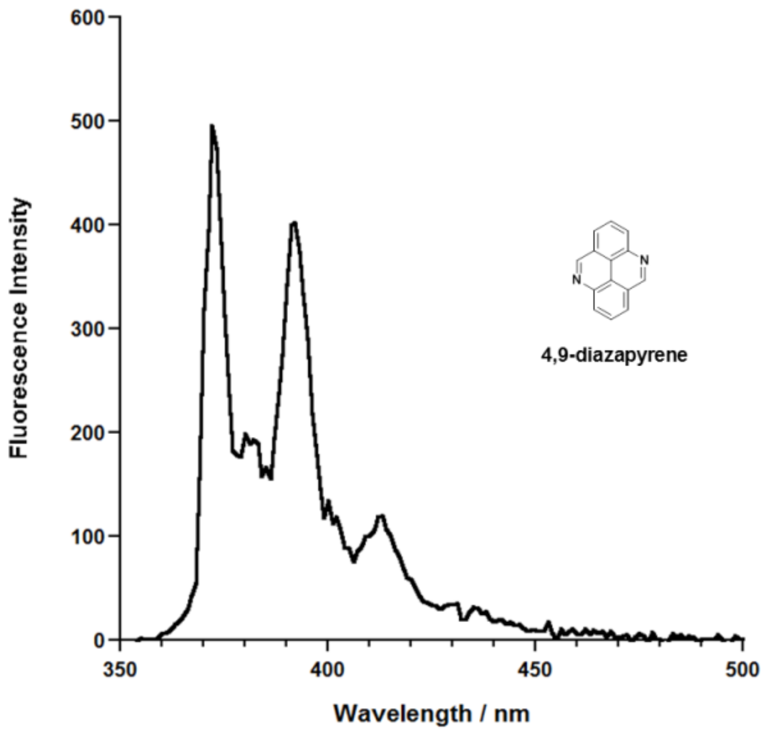

Figure S2. Emission spectrum of 4,9-diazapyrene. c = 2×10^{-5} M in THF, excited at 330 nm. Related to **Figure 7**.

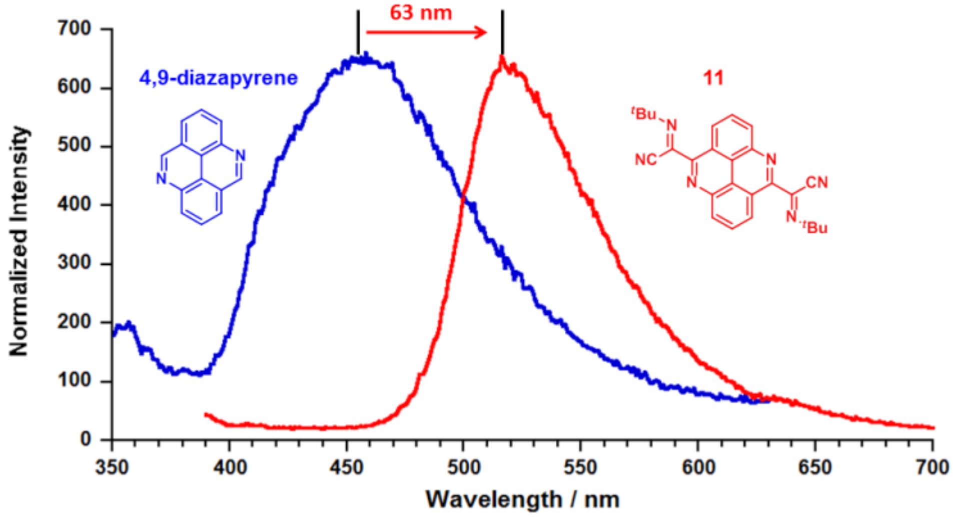

Figure S3. Emission spectra of 4,9-diazapyrene (blue curve) and compound **11** (red curve) in the solid state. Related to **Figure 7**.

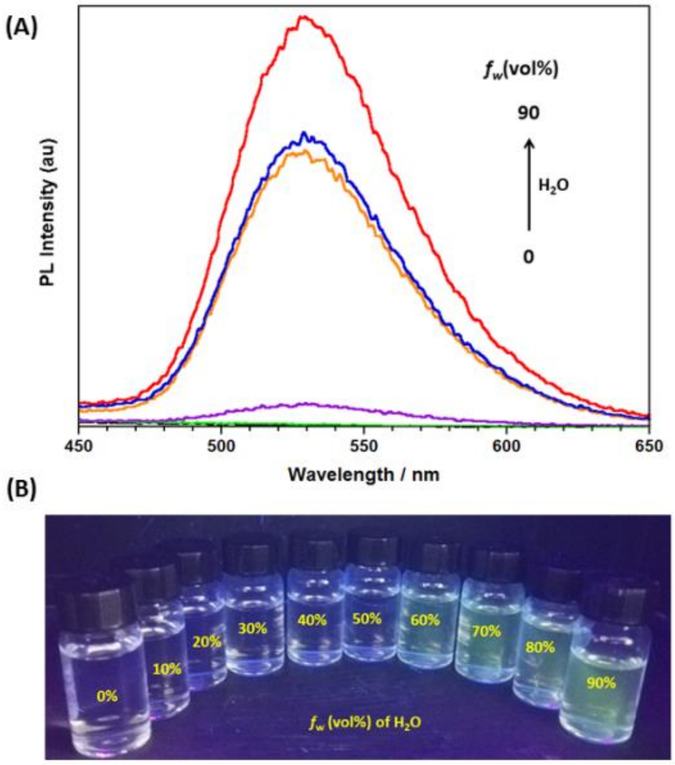

Figure S4. Aggregation-induced Emssion (AIE) of Compound **11**. **(A)** PL spectra of **11** in THF/water mixtures with different fractions of water (f_w). Observation of the aggregation-induced emission (AIE): Stock solutions of **11** with a concentration of 200 μM in THF were first prepared; 1 mL aliquots of the stock solutions were transferred into 10 mL volumetric flasks; Appropriate amounts of THF were then added, after which water was added dropwise under vigorous stirring to furnish 20 μM solutions with defined fractions of water (0% to 90%). (**B**) Photographs taken under illumination of a UV lamp (365 nm). Related to **Figure 7**.

X-ray Crystallographic Analysis for 2a, 4h, 6a and 8b

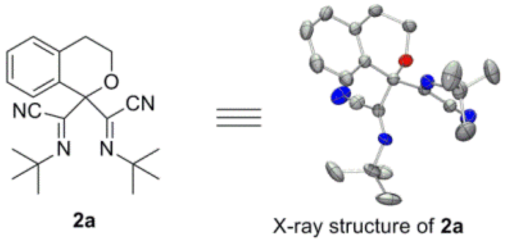

Figure S5. Crystallographic data for **2a.** 25% probability ellipsoids. $C_{21}H_{26}N_4O$, M = 350.46, Monoclinic, P 21/c (No. 14), a = 13.460 (11) Å, b = 9.739 (8) Å, c = 16.398 (13) Å, β = 100.331 (10)^o, V = 2115 (3) Å^3, Z = 4, Crystal size: 0.24 × 0.22 × 0.18 mm, T = 293 K, R_1 = 0.0713 (I>4σ(I)), wR_2 = 0.2813 (all data), GOF = 1.048, reflections collected/unique: 11496 / 4758 (Rint = 0.0660), Data: 2565, restraints: 0, parameters: 236. CCDC 1533930 contains the supplemental crystallographic data for this paper. The data can be obtained free of charge from The Cambridge Crystallographic Data Centre via www.ccdc.cam.ac.uk/data_request/cif. Related to **Table 1**.

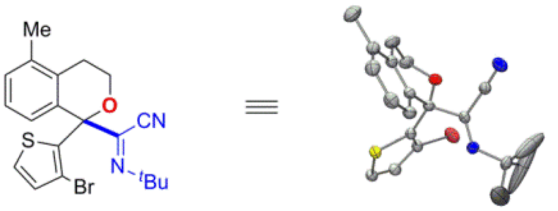

4h X-ray structure of **4h**

Figure S6. Crystallographic data for **4h**. 25% probability ellipsoids; $C_{20}H_{21}BrN_2OS$, M = 417.36, monoclinic, P21/c (No. 14), a = 9.581 (5) Å, b = 13.015 (6) Å, c = 16.536 (8) Å, β = 106.114 (6)°, V = 1981 (2) Å^3, Z = 4, Crystal size: 0.26 × 0.18 × 0.14 mm, T = 293 K, R_1 = 0.0351 (I>4σ(I)), wR_2 = 0.0905 (all data), GOF = 1.055, reflections collected/unique: 9979 / 3503 (Rint = 0.0246), Data: 2600, restraints: 0, parameters: 254. CCDC 1534967 contains the supplemental crystallographic data for this paper. The data can be obtained free of charge from The Cambridge Crystallographic Data Centre via www.ccdc.cam.ac.uk/data_request/cif. Related to **Figure 3**.

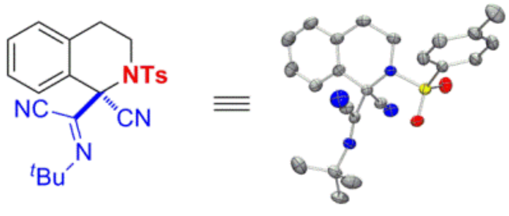

6a X-ray structure of **6a**

Figure S7. Crystallographic data for **6a.** 25% probability ellipsoids; Chemical Formula: $C_{23}H_{24}N_4O_2S$, M = 420.52, monoclinic, P21/n, a = 9.745 (8) Å, b = 11.135 (9) Å, c = 20.716 (16) Å, β = 93.037 (11)°, V = 2245 (3) Å^3, Z = 4, Crystal size: 0.24 × 0.15 × 0.12 mm, T = 293 K, R_1 = 0.0541 (I>4σ(I)), wR_2 = 0.1748 (all data), GOF = 1.050, reflections collected/unique: 9937/3942 (Rint = 0.0700), Data: 2417, restraints: 0, parameters: 271. CCDC 1829908 contains the supplemental crystallographic data for this paper. The data can be obtained free of charge from The Cambridge Crystallographic Data Centre via www.ccdc.cam.ac.uk/data_request/cif. Related to **Figure 4**.

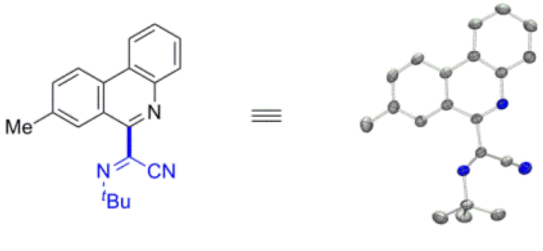

8b X-ray structure of **8b**

Figure S8. Crystallographic data for **8b**. 25% probability ellipsoids; Chemical Formula: $C_{20}H_{19}N_3$, M = 301.38, triclinic, P -1, a = 7.219 (9) Å, b = 9.096 (11) Å, c = 13.168 (16) Å, α = 79.250 (14)°, β = 83.431 (14)°, γ = 89.505(15)°, V = 844 (2) Å^3, Z = 2, Crystal size: 0.21 × 0.18 × 0.14 mm, T = 293 K, R_1 = 0.0531 (I>4σ(I)), wR_2 = 0.1663 (all data), GOF = 1.058, reflections collected/unique: 5215/3694 (Rint = 0.0238), Data: 2421, restraints: 0, parameters: 209. CCDC 1829633 contains the supplemental crystallographic data for this paper. The data can be obtained free of charge from The Cambridge Crystallographic Data Centre via www.ccdc.cam.ac.uk/data_request/cif. Related to **Figure 5**.

Mechanistic Studies, Related to Figure 8 and Figure 9.

(A) Control Experiments, Related to Figure 8.

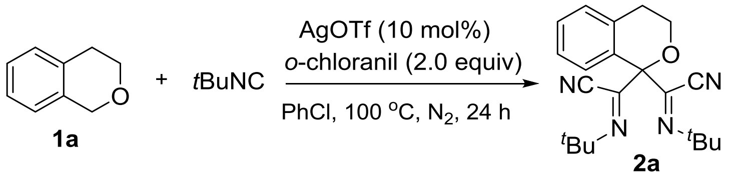

1a **2a**

To a sealed tube were added **1a** (40.2 mg, 0.3 mmol), ^{t}BuNC (170 μL, 1.5 mmol), AgOTf (7.8 mg, 0.03 mmol), *o*-chloranil (147.5 mg, 0.6 mmol) in dry PhCl (3.0 mL) in the glove box. The mixture was stirred at 100 °C under N_2 for 24 h. Upon completation, the reaction mixture was cooled down to room temperature and purified by silica gel plate to give product **2a** as white solid (6.8 mg, 6%).

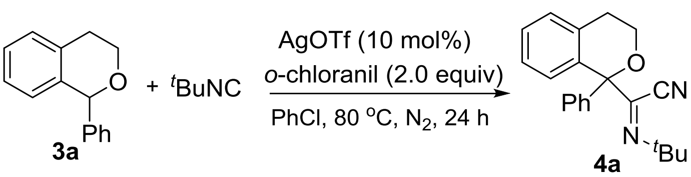

3a **4a**

To a test tube were added **3a** (63.1 mg, 0.3 mmol), ^{t}BuNC (170 μL, 1.5 mmol), AgOTf (7.8 mg, 0.03 mmol), *o*-chloranil (147.5 mg, 0.6 mmol) in dry PhCl (3.0 mL) in the glove box. The mixture was stirred at 80 °C under N_2 for 24 h. Upon completation, the reaction mixture was cooled down to room temperature and purified by column chromatography on silica gel to give product **4a** as white solid (22.9 mg, 24%).

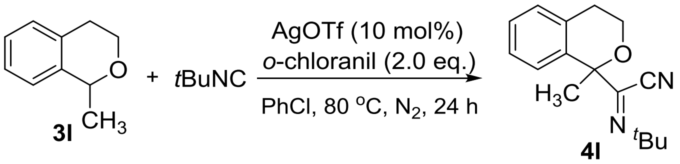

3l **4l**

To a test tube were added **3l** (44.4 mg, 0.3 mmol), ^{t}BuNC (170 μL, 1.5 mmol), AgOTf (7.8 mg, 0.03 mmol), *o*-chloranil (147.5 mg, 0.6 mmol) in dry PhCl (3.0 mL) in the glove box. The mixture was stirred at 80 °C under N_2 for 24 h. Upon completation, the reaction mixture was cooled down to room temperature and was purified by column chromatography on silica gel to give product **4l** as pale yellow oil (26.5 mg, 34%).

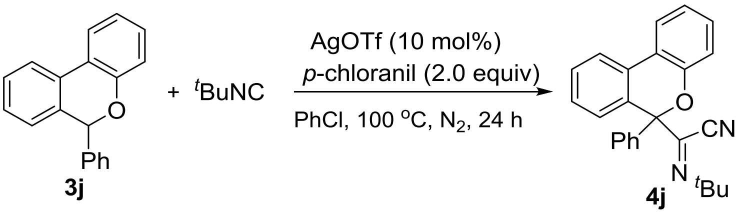

3j **4j**

To a sealed tube were added **3j** (77.5 mg, 0.3 mmol), ^{t}BuNC (170 μL, 1.5 mmol), AgOTf (7.8 mg, 0.03 mmol), *p*-chloranil (147.5 mg, 0.6 mmol) in dry PhCl (3.0 mL) in the glove box. The mixture was stirred at 100 °C under N_2 for 24 h. Upon completation, the reaction mixture was cooled down to room temperature and purified by column chromatography on silica gel to give product **4j** as pale yellow solid (78.3 mg, 71%).

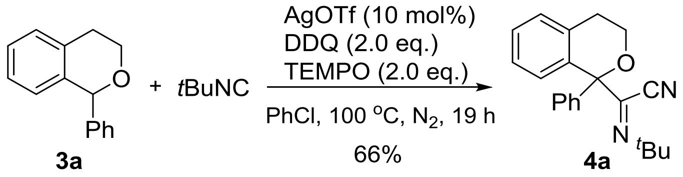

To a test tube were added **3a** (63.1 mg, 0.3 mmol), ^{t}BuNC (169 μL, 1.5 mmol), AgOTf (7.8 mg, 0.03 mmol), DDQ (139.0 mg, 0.6 mmol), and TEMPO (93.8 mg, 0.6 mmol) in dry PhCl (3.0 mL) in the glove box. The mixture was stirred at 100 °C under N_2 for 19 h. Upon completion, the reaction mixture was cooled down to room temperature and was purified by column chromatography on silica gel to give product **4a** as pale yellow oil (63.3 mg, 66%). In the absence of the radical scavenger TEMPO, the yield was 68%. These results indicate that the radical pathway can probably been ruled out.

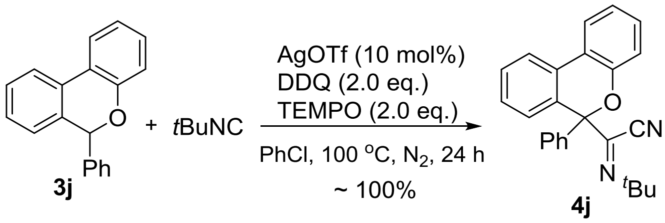

To a test tube were added **3j** (77.4 mg, 0.3 mmol), ^{t}BuNC (169 μL, 1.5 mmol), AgOTf (7.9 mg, 0.03 mmol), DDQ (139.0 mg, 0.6 mmol), and TEMPO (93.8 mg, 0.6 mmol) in dry PhCl (3.0 mL) in the glove box. The mixture was stirred at 100 °C under N_2 for 24 h. Upon completion, the reaction mixture was cooled down to room temperature and was purified by column chromatography on silica gel to give product **4j** as pale yellow oil (82.0 mg, ~100%). In the absence of the radical scavenger TEMPO, the yield was 98%. These results again indicate that the radical pathway can probably been ruled out.

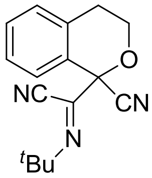

(*E*)-*N*-(*tert*-Butyl)-1-cyanoisochroman-1-carbimidoyl cyanide (2a'):

To a sealed tube was added **1a** (40.2 mg, 0.3 mmol), ^{t}BuNC (3.0 equiv), AgOTf (7.8 mg, 0.03 mmol), DDQ (139.0 mg, 0.6 mmol) in dry PhCl (3.0 mL) in the glove box. The mixture was stirred at 80 °C under N_2 for 3 h. Upon completation, the reaction mixture was cooled down to room temperature and purified by silica gel plate to give products **2a** (36.8 mg, 35%)**, 2a**' (12.8 mg, 16%) and **6a** (13.4 mg, 28%), respectively. colorless oil; IR (KBr, cm^{-1}): 2978, 2220, 1647, 1453, 1367, 1285, 1195, 1101, 1061, 762, 746; ^{1}H NMR ($CDCl_3$, 500 MHz): *δ* 7.37 (td, *J* = 7.5, 1.1 Hz, 1H), 7.31 (t, *J* = 7.5 Hz, 1H), 7.26 (d, *J* = 7.5 Hz, 1H), 7.18-7.17 (m, 1H), 4.43-4.39 (m, 1H), 4.13 (td, *J* = 11.8, 2.7 Hz, 1H), 3.32-3.25 (m, 1H), 2.75 (dd, *J* = 16.5, 2.0 Hz, 1H), 1.48 (s, 9H); ^{13}C NMR ($CDCl_3$, 125 MHz): 136.3, 134.3, 130.0, 129.9, 127.8, 127.7, 126.1, 116.3, 109.4, 80.2, 63.4, 59.7, 29.0, 27.3; LC-MS (ESI) m/z 268 [M+H]$^+$; HRMS (EI) m/z calcd for $C_{16}H_{18}ON_3$ [M+H]$^+$ 268.1444, found 268.1446.

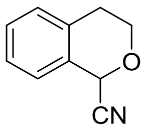

Isochroman-1-carbonitrile (12) (Yan et al., 2014): white solid. M.p. 43-44 ^{o}C; IR (KBr, cm^{-1}): 3071, 3030, 2973, 2933, 2866, 2734, 2232, 2093, 1929, 1821, 1603, 1489, 1434, 1289, 1262, 1197, 1099, 992, 956, 892, 751; ^{1}H NMR ($CDCl_3$, 500 MHz): δ 7.31-7.26 (m, 2H), 7.22-7.17 (m, 2H), 5.65 (s, 1H), 4.19-4.10 (m, 2H), 3.05-3.01 (m, 1H), 2.77 (dt, J = 17.0, 3.2 Hz, 1H); ^{13}C NMR ($CDCl_3$, 125 MHz): 132.9, 129.4, 129.1, 128.7, 127.0, 125.4, 118.1, 65.3, 63.3, 27.2; EI-MS m/z (%): 159 (88) $[M]^{+}$, 131 (42), 129 (100), 102 (35), 77 (23).

(B) Mass Spectrometry, Related to Figure 9.

Experimental conditions

Tandem Mass spectrometry instrument:

The electrospray ionization mass spectrometry (ESI-MS) and the subsequent tandem mass spectrometry (ESI-MS/MS) experiments were performed in Thermo TSQ Quantum Access^{TM} triple-quadrupole mass spectrometer (Thermo-Fisher Scientific, Waltham, MA, USA). The basic ESI-MS conditions were: spray voltage, 3000 V; capillary temperature, 275 ^{o}C; sheath gas pressure, 2 arb. units; aux gas pressure, 2 arb. units; the collision energy ranged from 5 to 30 eV depending on the dissociation capability of the precursor ions in MS/MS. Data acquisition and analysis were carried out with the Xcalibur software package (Version 2.0, Thermo Fisher Scientific).

General MS experimental conditions:

The concentration of the reaction solution was too high for direct ESI-MS analysis. Therefore, the concentrated reaction solutions in solvent CH_2Cl_2 were first filtered by 0.5 µm membrane and then were diluted 200 times with CH_2Cl_2 before ESI-MS analysis. The diluted CH_2Cl_2 solution was injected by a 500 µL air-tight syringe with speed of the diluted solution was set to 8 µL/min to ESI-MS. We carefully monitored the diluted reaction solution by ESI-MS and found some signals of the reactive intermediates. The electrospray ionization tandem mass spectrometry (ESI-MS/MS) method was performed to assign the possible structures of the reactive intermediates observed by ESI-MS.

Mass spectrometric experiment results

The *Reaction Solution 1* was prepared by mixing **1a** (39.0 µL, 0.3 mmol), ^{t}BuNC (170.0 µL, 1.5 mmol), AgOTf (7.8 mg, 0.03 mmol), DDQ (139.0 mg, 0.6 mmol) in dry CH_2Cl_2 (3.0 mL). In order to get better and stable signal in ESI-MS analysis, the solvent PhCl at 80 ^{o}C (Eq. 1 in Scheme S1) was displaced by CH_2Cl_2 (Eq. 2 in Scheme S1) at room temperature. The synthetic experiments showed that the reaction could also work at such condition. The mixture was stirred at room temperature and ready for measurement in different reaction time.

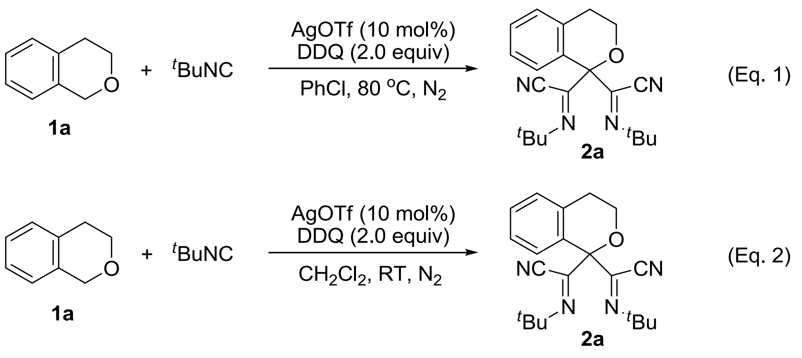

Scheme S1. The typical reaction condition and the reaction condition for ESI-MS studying by using CH_2Cl_2 as solvent. Related to **Figure 9**

The corresponding signal of some important ionic reactive species in the early stage of the reaction, such as **B** at *m/z* 133, **D** at *m/z* 299, [**E**+H]$^+$ at *m/z* 243 were observed in the positive ion ESI-MS spectrum of *Reaction Solution 1* (Figure S1a). The possible structures of these intermediates were supposed in Scheme S2 and the ESI-MS/MS experiments for these species were performed and shown in Figure S2. Their proposed dissociation pathways supported their proposed structures (Scheme S3).

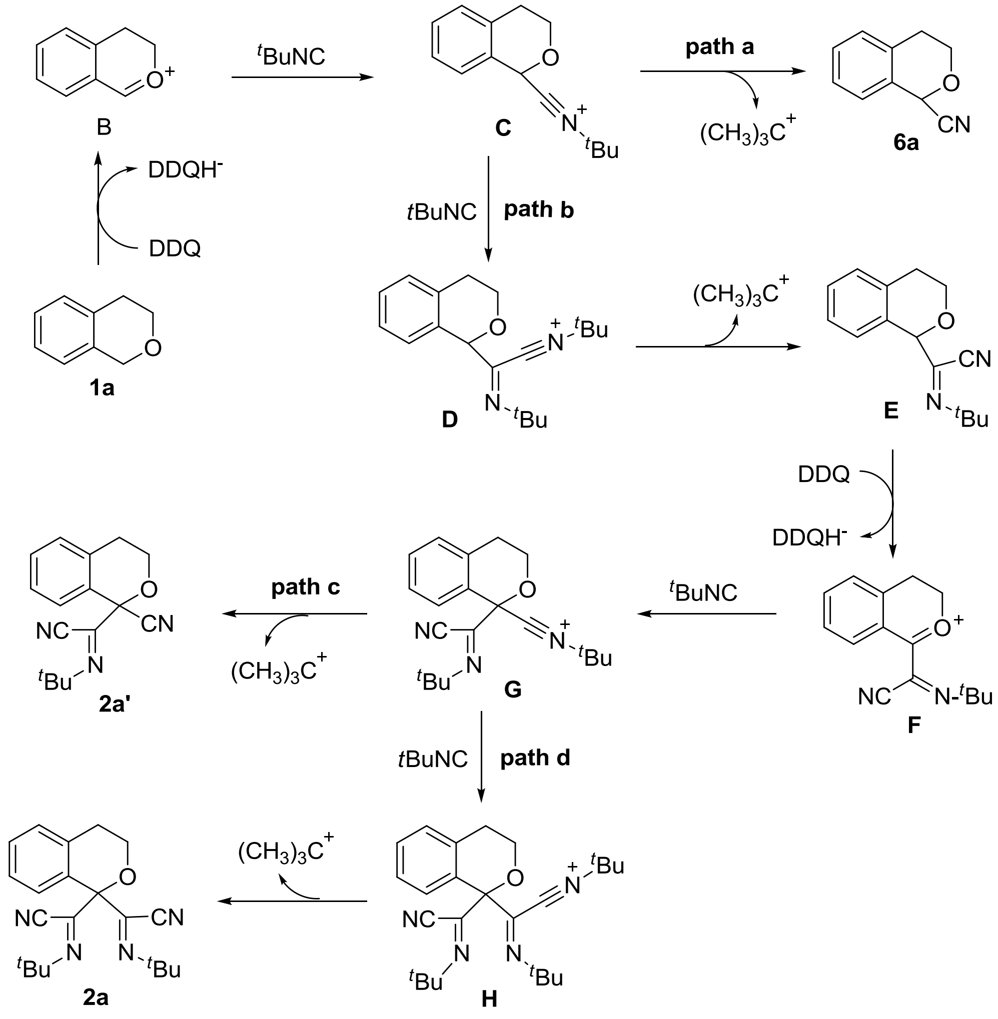

Scheme S2. The possible process of the cascade insertion reaction. Related to **Figure 9**.

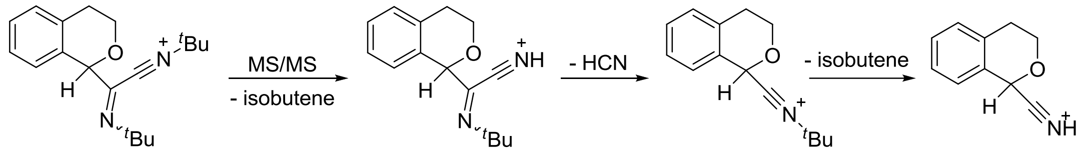

D at *m/z* 299 [**E**+H]$^+$ at *m/z* 243 **C** at *m/z* 216 [**6a**+H]$^+$ *m/z* 160

Scheme S3. The proposed fragmentation patterns of the important ionic reactive intermediate **D** at *m/z* 299, which could give rise to [**E**+H]$^+$ at *m/z* 243 by loss of isobutene. These results supported such structure assignments. Related to **Figure 9**.

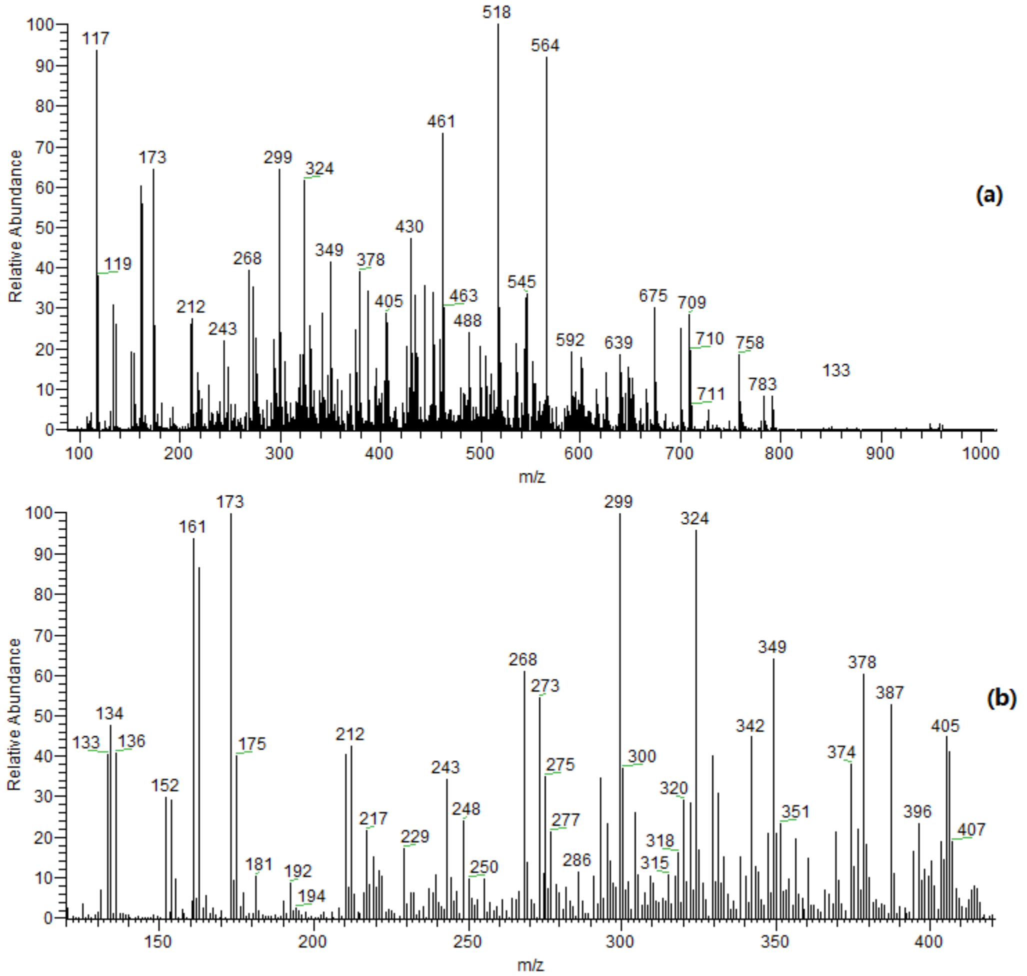

Figure S9. (a) The ESI-MS spectrum in positive ion mode of the diluted *Reaction Solution 1* at reaction time of 30 min; (b) the expanded ESI-MS spectrum in positive ion mode of *Reaction Solution 1* at reaction time of 30 min. Related to **Figure 9**.

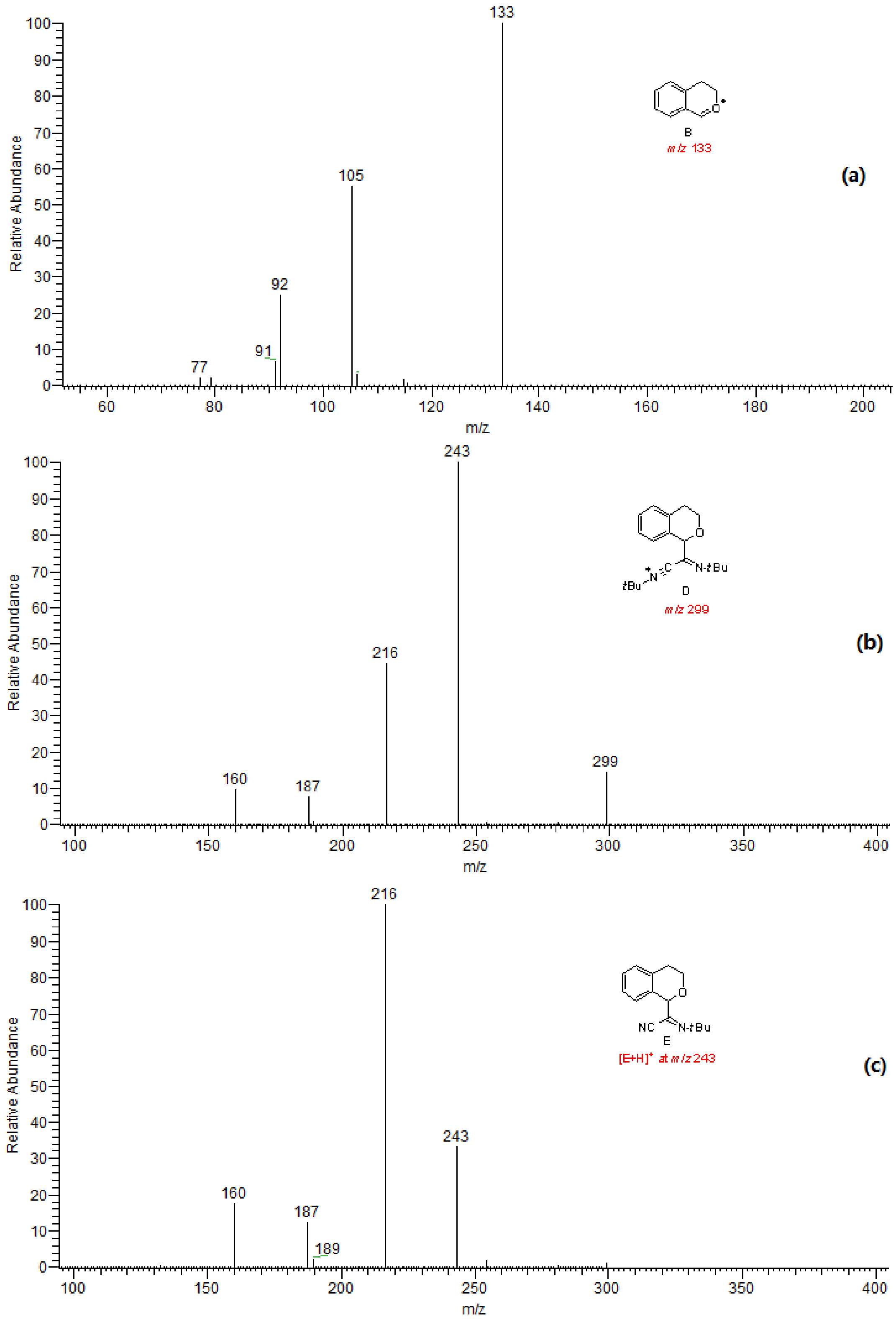

Figure S10. The ESI-MS/MS spectra in positive ion mode of ionic species from *Reaction Solution 1*: (a) at *m/z* 133; (b) at *m/z* 299; (c) at *m/z* 243. Related to **Figure 9**.

The corresponding signal of some important ionic reactive species in the early stage of the reaction, such as **G** at *m/z* 324, [**2a’**+H]$^+$ at *m/z* 268, and **H** at *m/z* 407 were observed in the positive ion ESI-MS spectrum of *Reaction Solution 1* (Figure S1). The possible structures of these intermediates were supposed in Scheme S2 and the ESI-MS/MS experiments for these species were performed and shown in Figure S3. Their proposed dissociation pathways supported their proposed structures (Scheme S4).

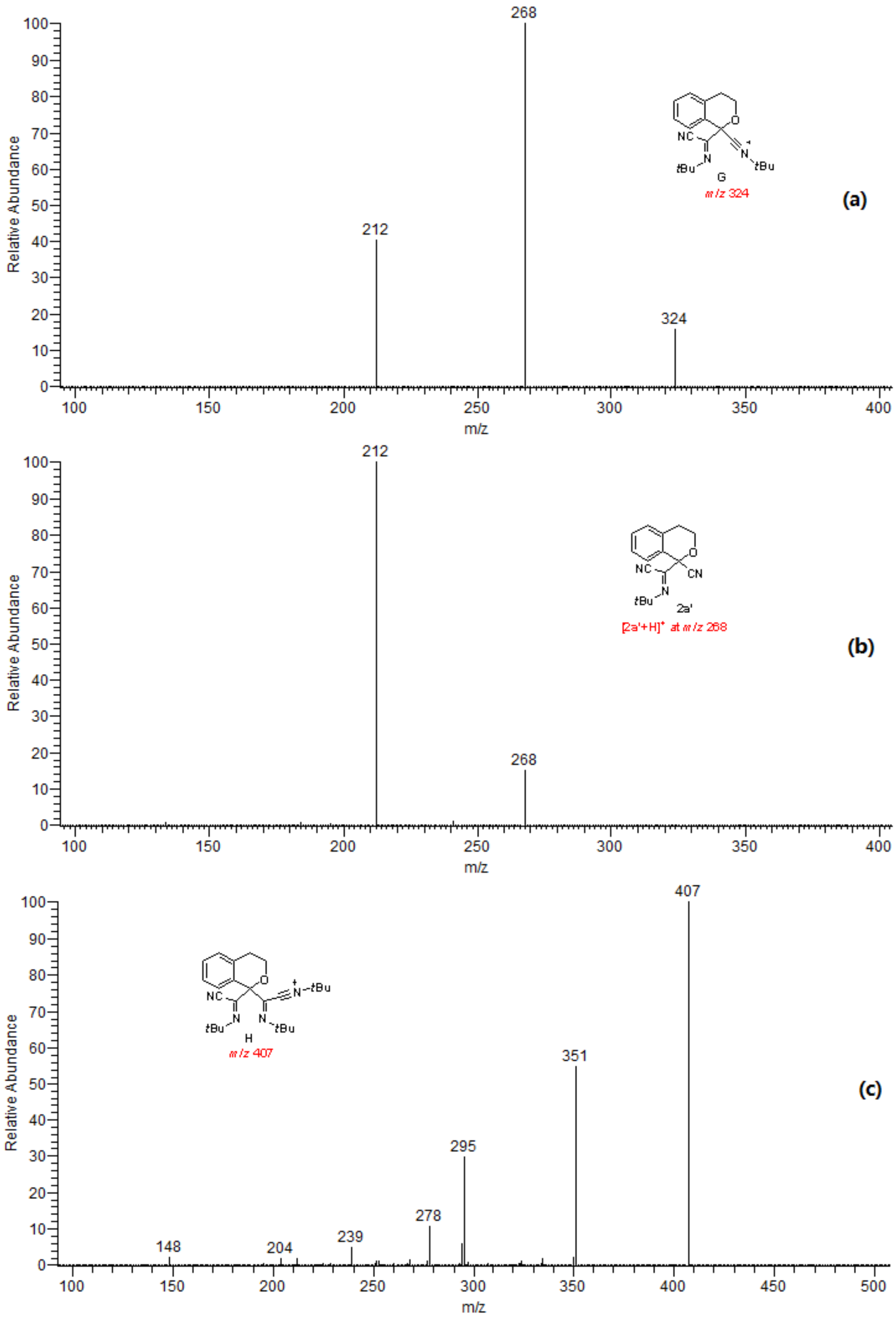

Figure S11. The ESI-MS/MS spectra in positive ion mode of ionic species from *Reaction Solution 1*: (a) at *m/z* 324; (b) at *m/z* 268; (c) at *m/z* 407. Related to **Figure 9**.

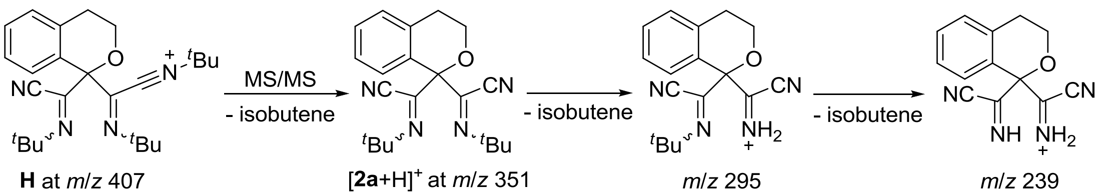

Scheme S4. The proposed fragmentation patterns of the important ionic reactive intermediate **H** at *m/z* 407, which could give rise to signal of the product [**2a**+H]$^+$ at *m/z* 351 by loss of isobutene. These results supported such structure assignments. Related to **Figure 9**.

The corresponding signal of some negative ionic species in the reaction, such as $CF_3SO_3^-$ at *m/z* 149, negative radical anion of DDQH$^-$ at *m/z* 226 were observed in the negative ion ESI-MS spectrum of *Reaction Solution 1* (Figure S4a). The ESI-MS/MS experiments of the negative radical anion of DDQH$^-$ at *m/z* 226 was performed and shown in Figure S4b, which is proposed structure (Figure S4b).

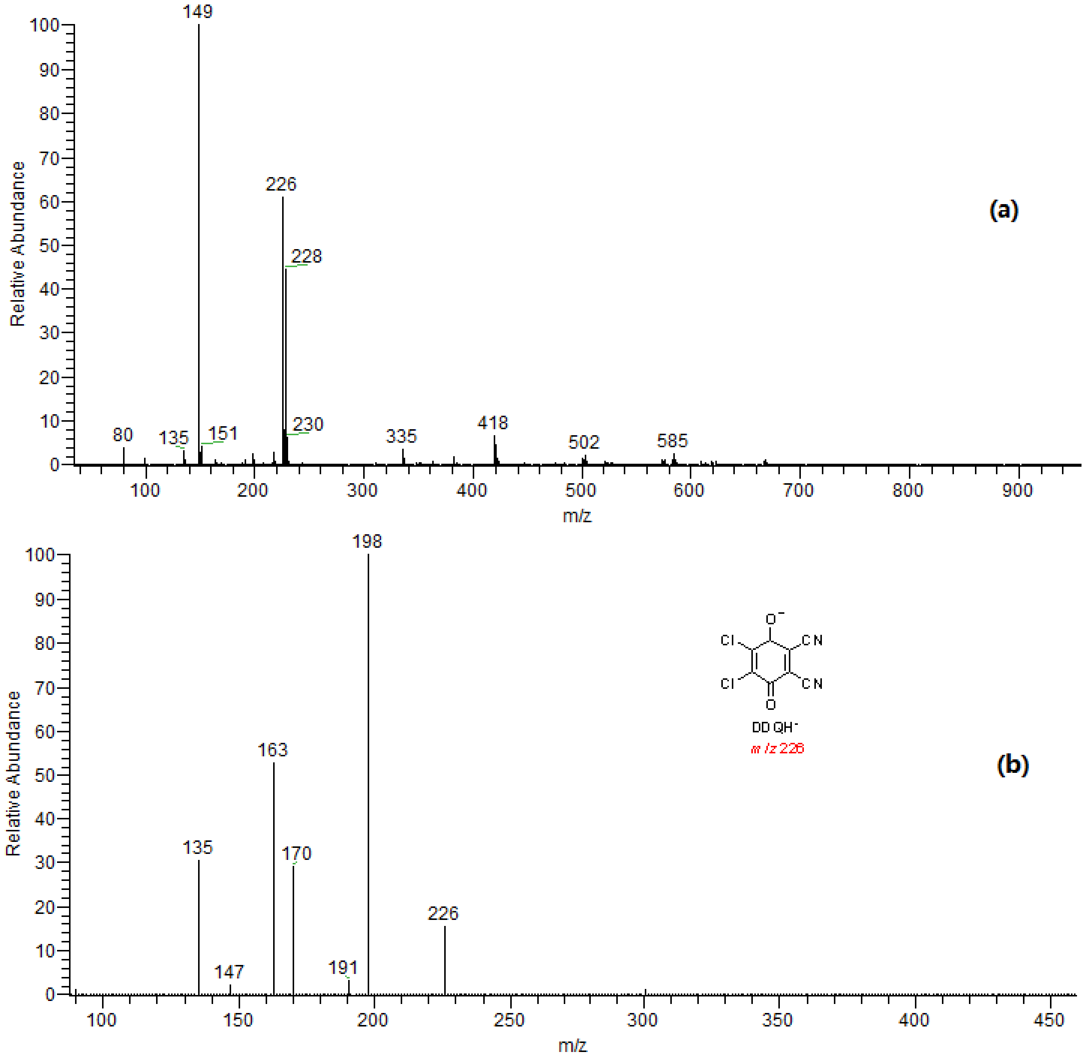

Figure S12. (a) The ESI-MS spectrum in negative ion mode of the diluted *Reaction Solution 1*; (b) the ESI-MS/MS spectrum of the negative ion at *m/z* 226. Related to **Figure 9**.

Supplemental Figures: ^{1}H, ^{13}C and ^{19}F NMR Spectra

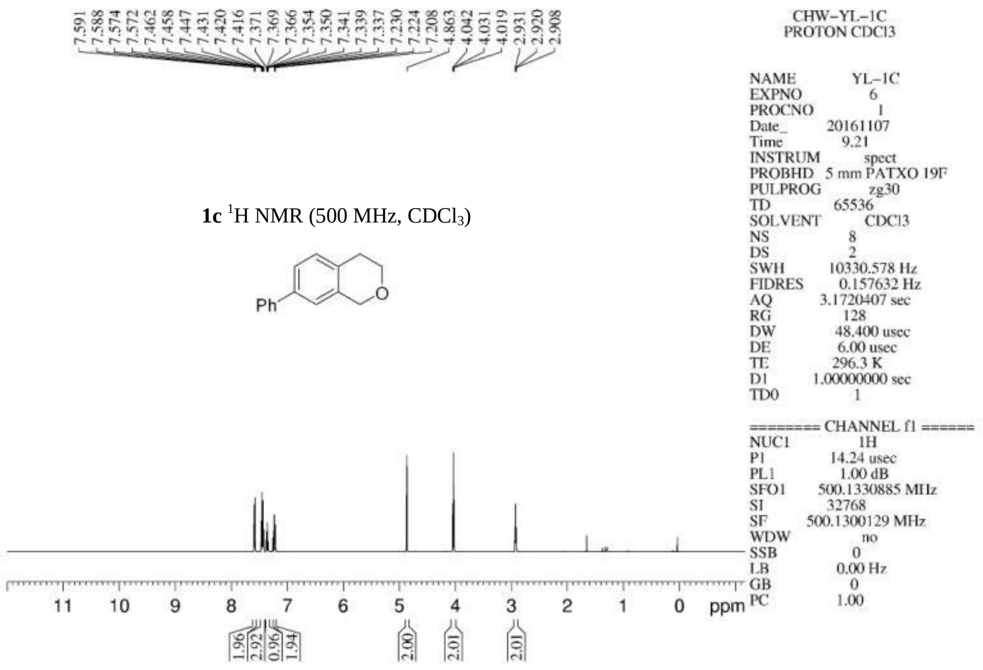

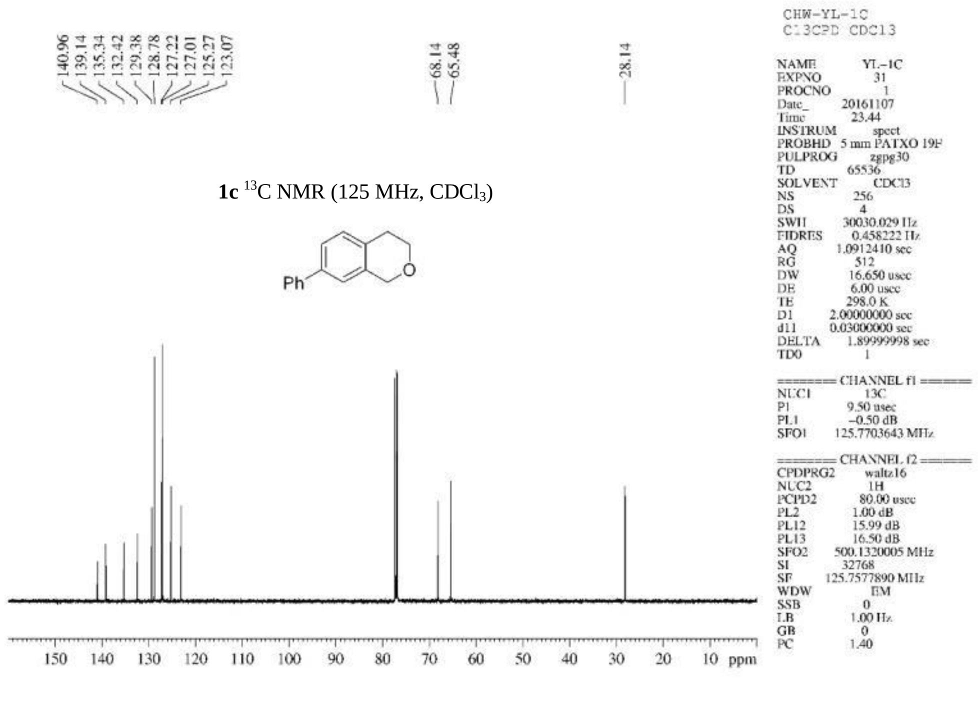

Figure S13. ^{1}H and ^{13}C NMR spectra of **1c.** Related to **Figure 2.**

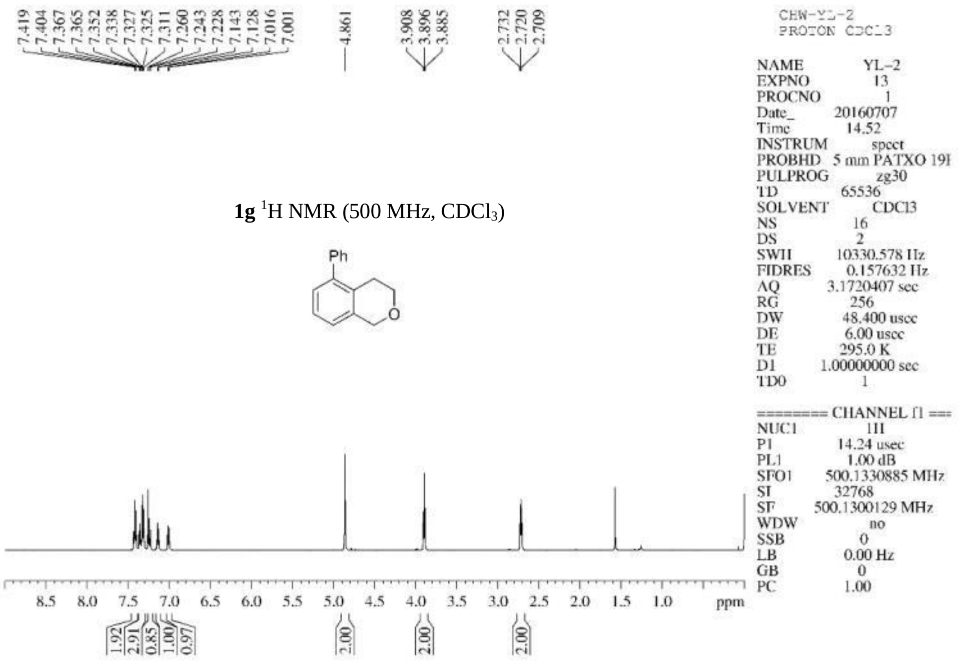

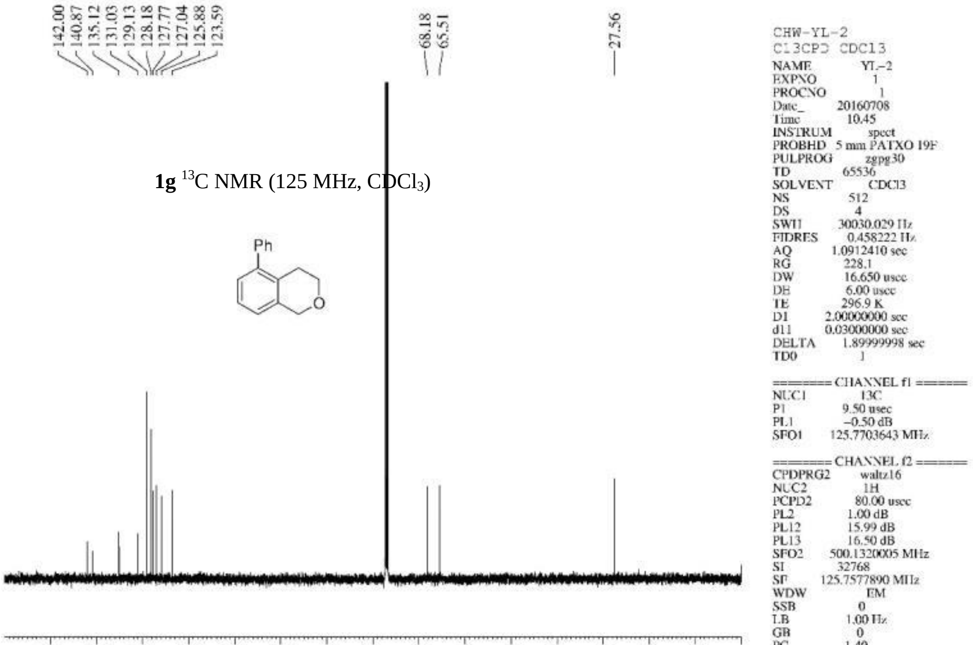

Figure S14. ^{1}H and ^{13}C NMR spectra of **1g**. Related to **Figure 2**.

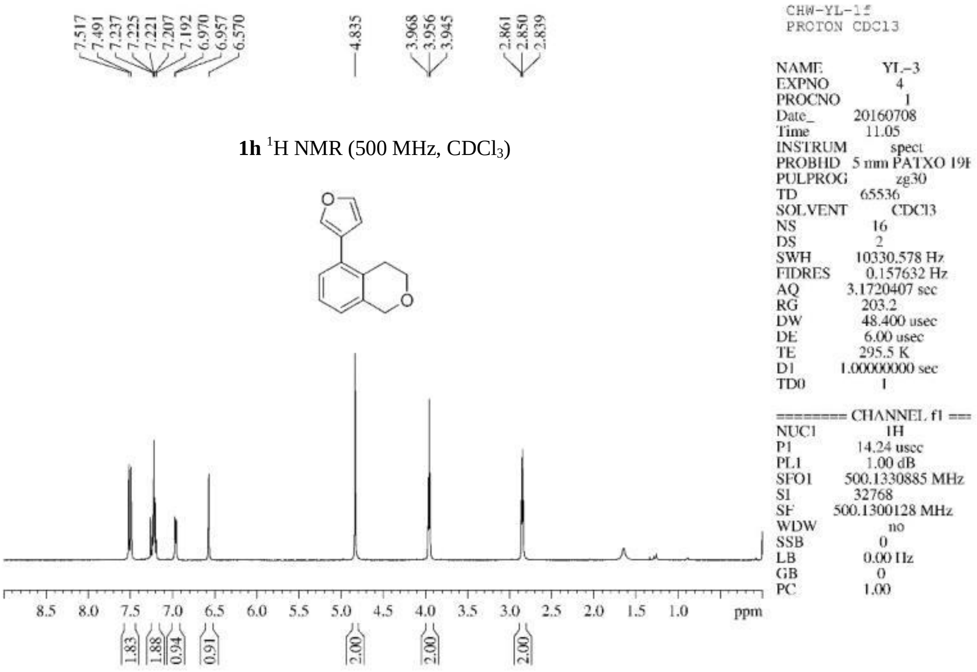

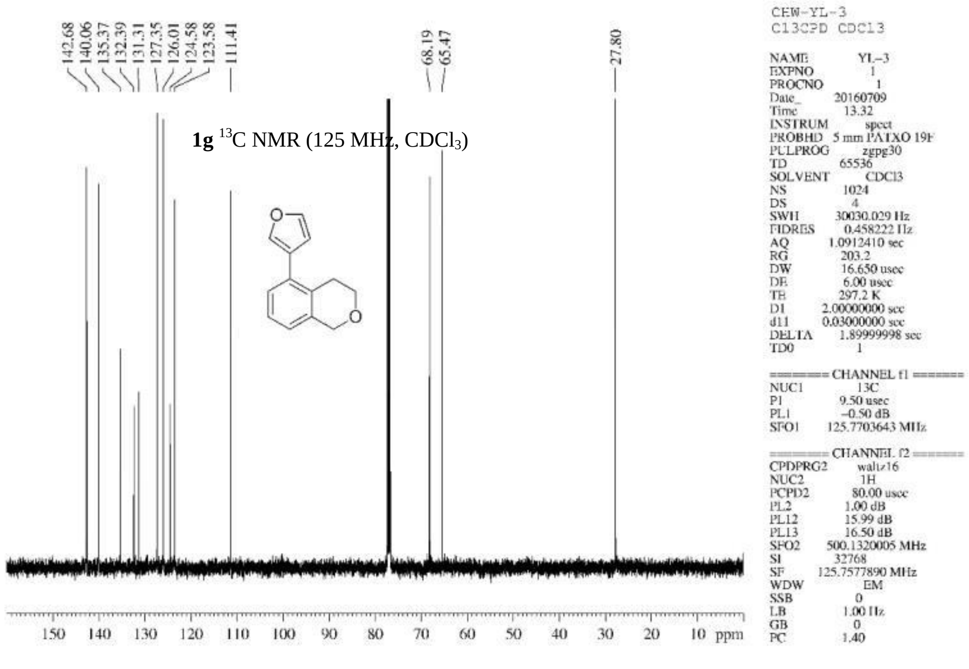

Figure S15. ^{1}H and ^{13}C NMR spectra of **1h.** Related to **Figure 2**.

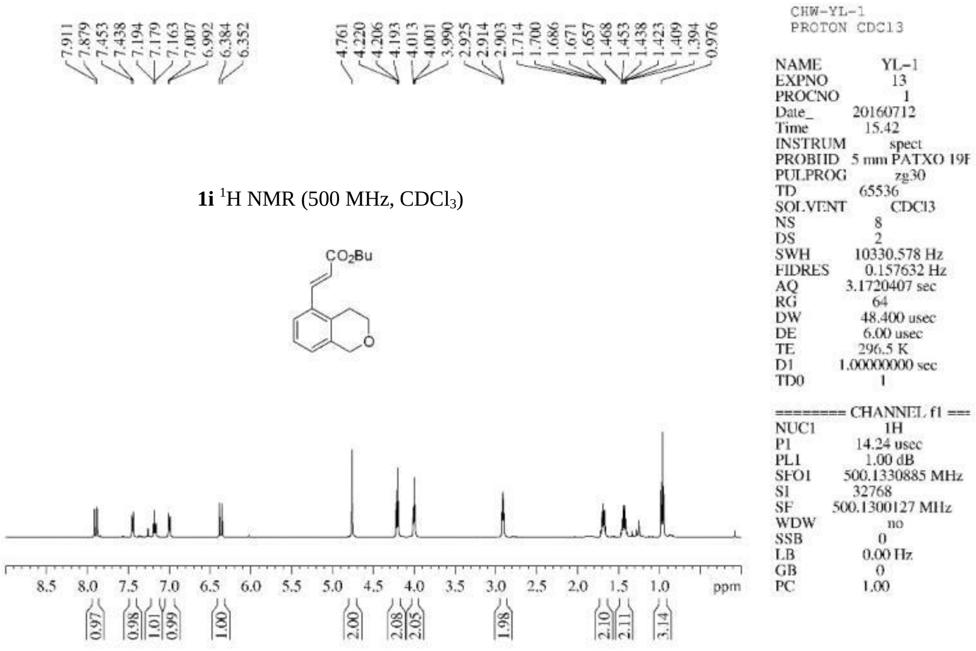

1i ^{1}H NMR (500 MHz, $CDCl_3$)

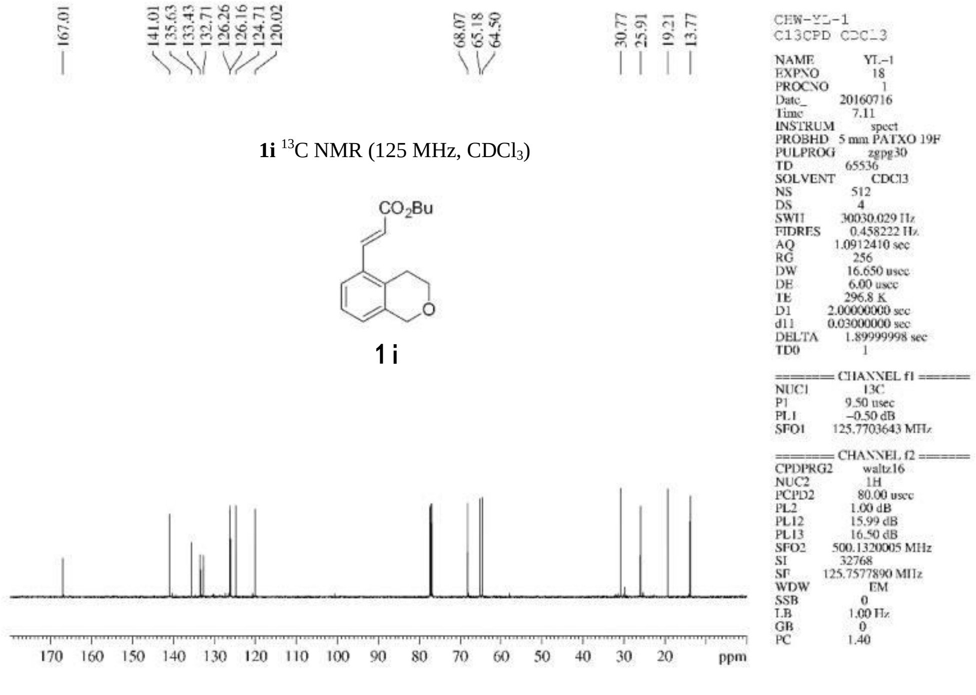

1i ^{13}C NMR (125 MHz, $CDCl_3$)

Figure S16. ^{1}H and ^{13}C NMR spectra of **1i**. Related to **Figure 2**.

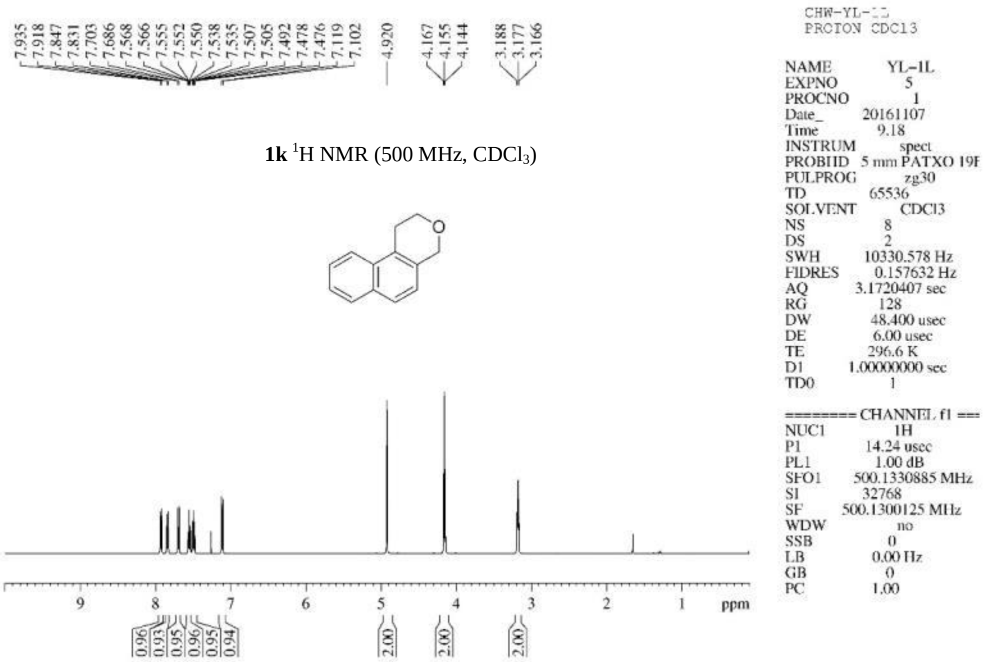

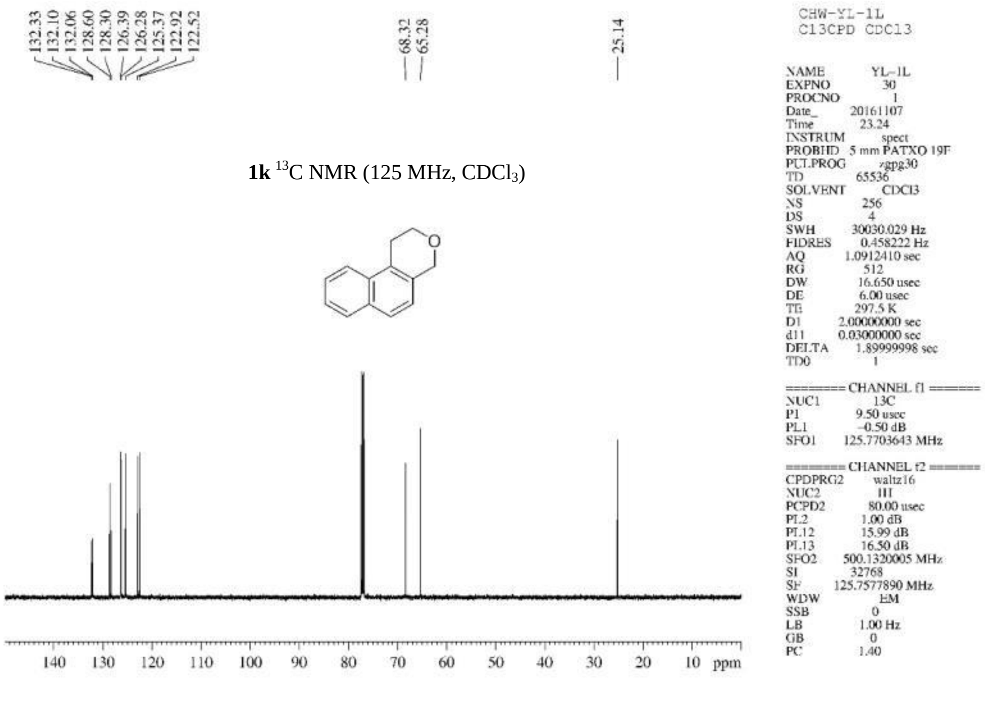

Figure S17. ^{1}H and ^{13}C NMR spectra of **1k**. Related to **Figure 2**.

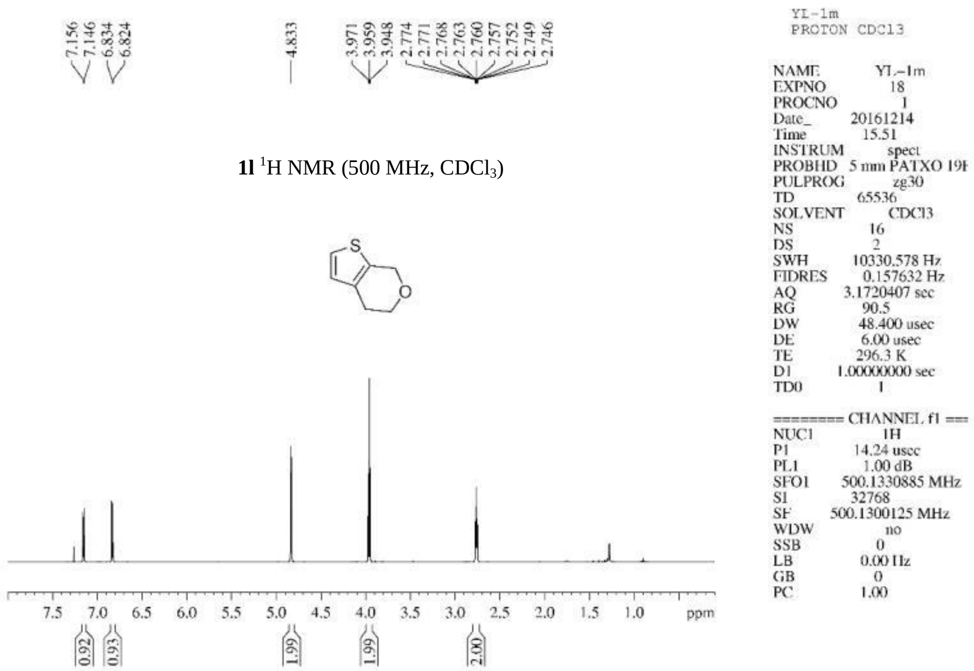

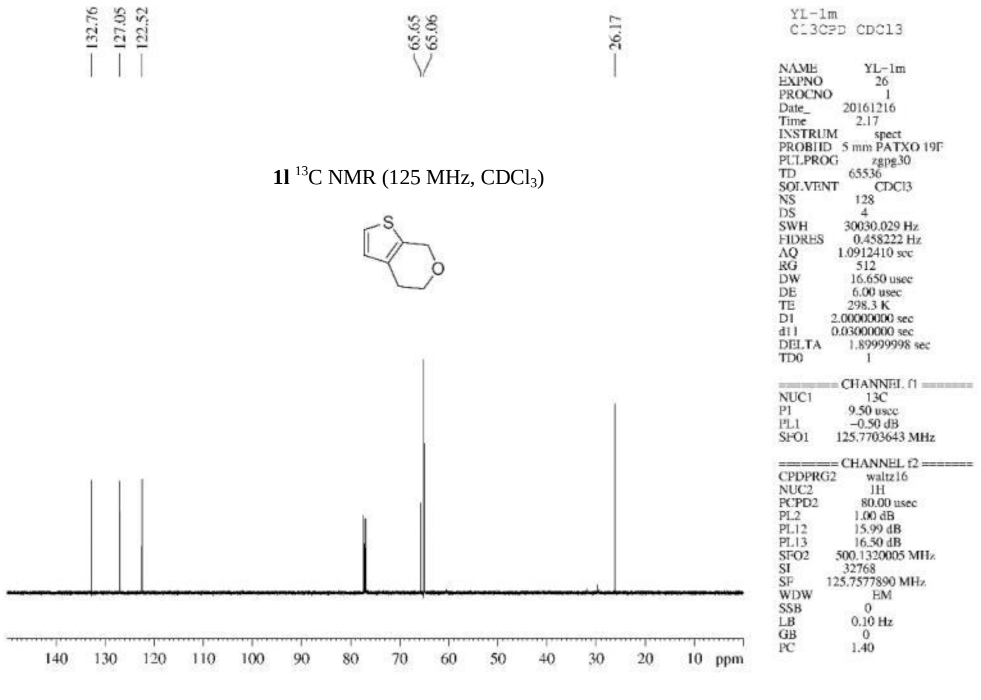

Figure S18. ^{1}H and ^{13}C NMR spectra of **1l**. Related to **Figure 2**.

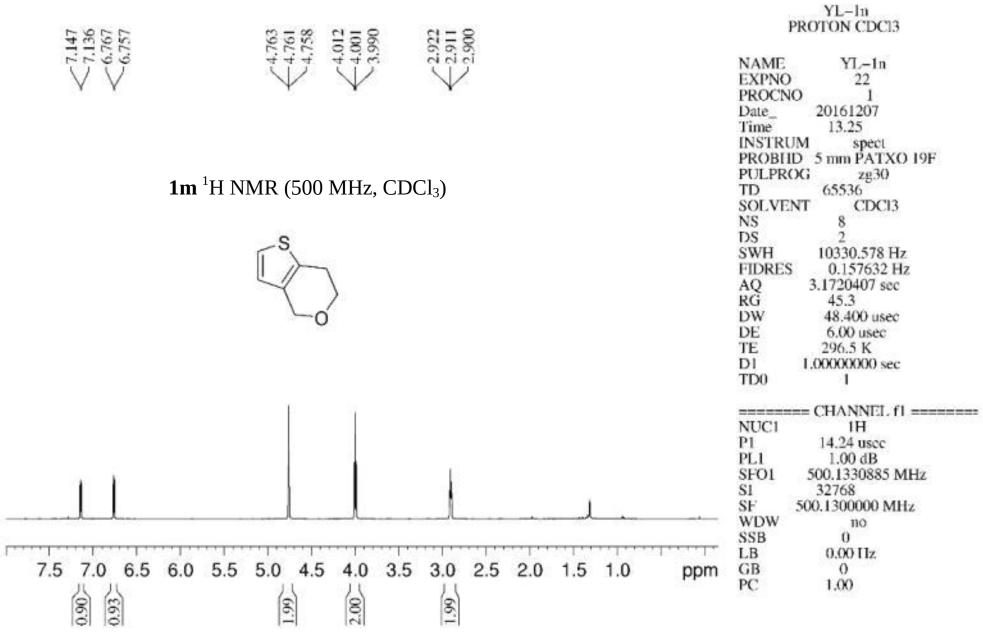

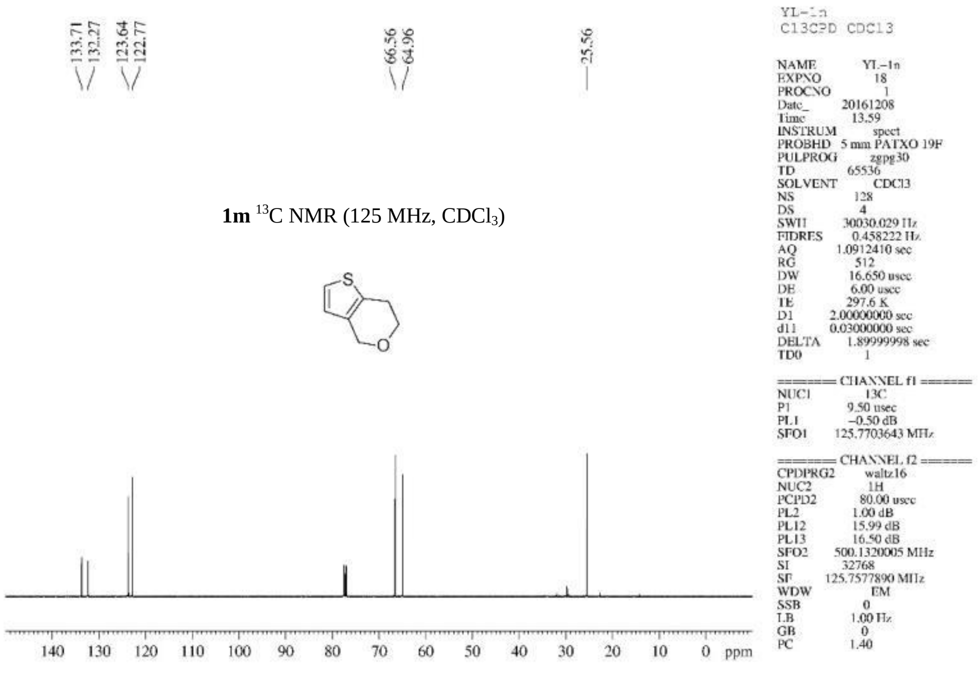

Figure S19. ^{1}H and ^{13}C NMR spectra of **1m**. Related to **Figure 2**.

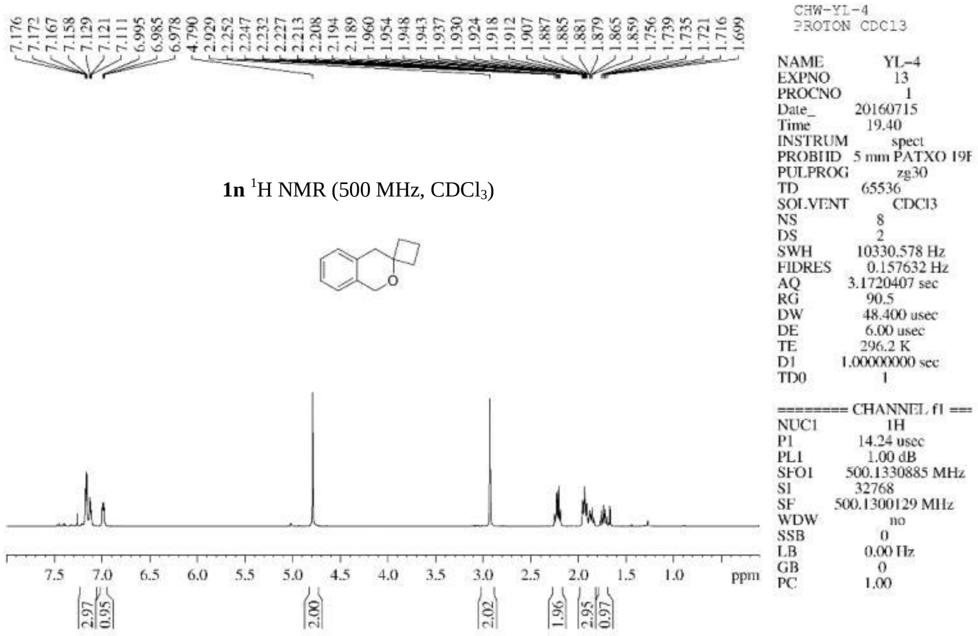

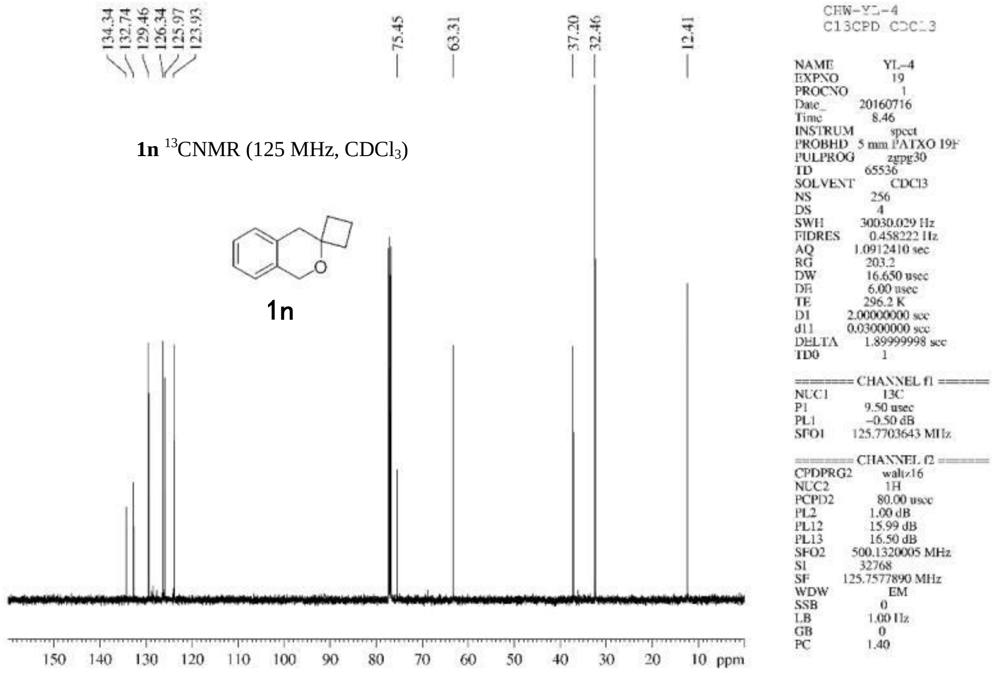

Figure S20. ^{1}H and ^{13}C NMR spectra of **1n.** Related to **Figure 2**.

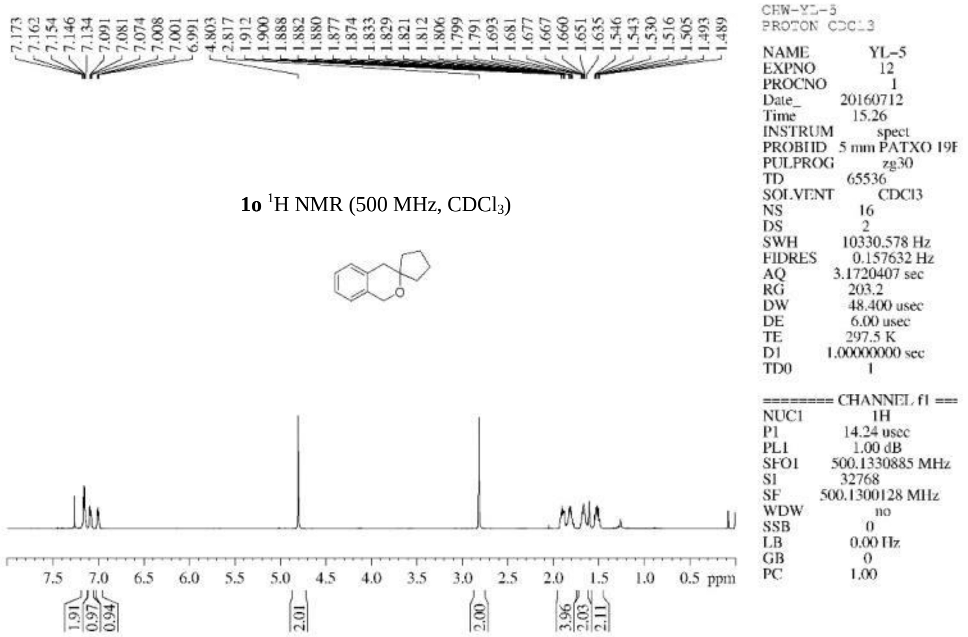

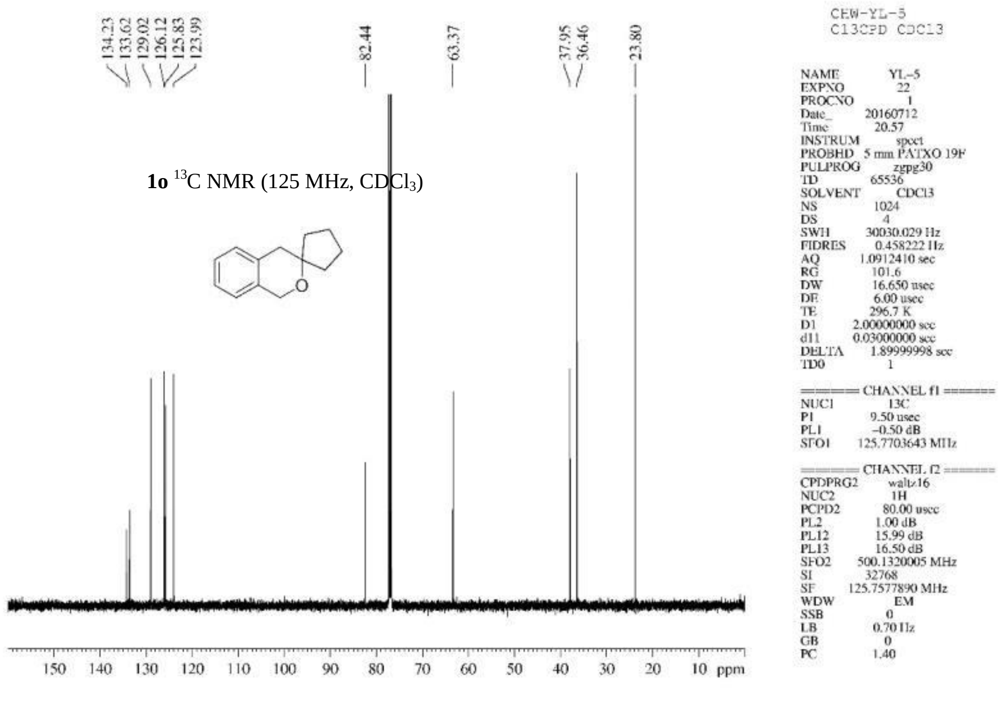

Figure S21. ^{1}H and ^{13}C NMR spectra of **1o**. Related to **Figure 2**.

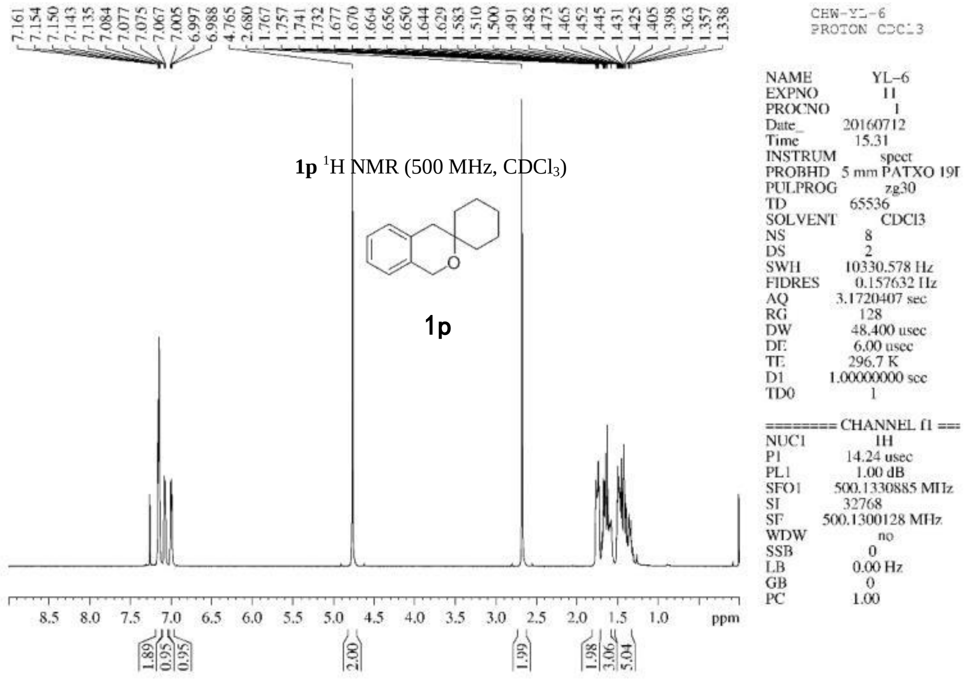

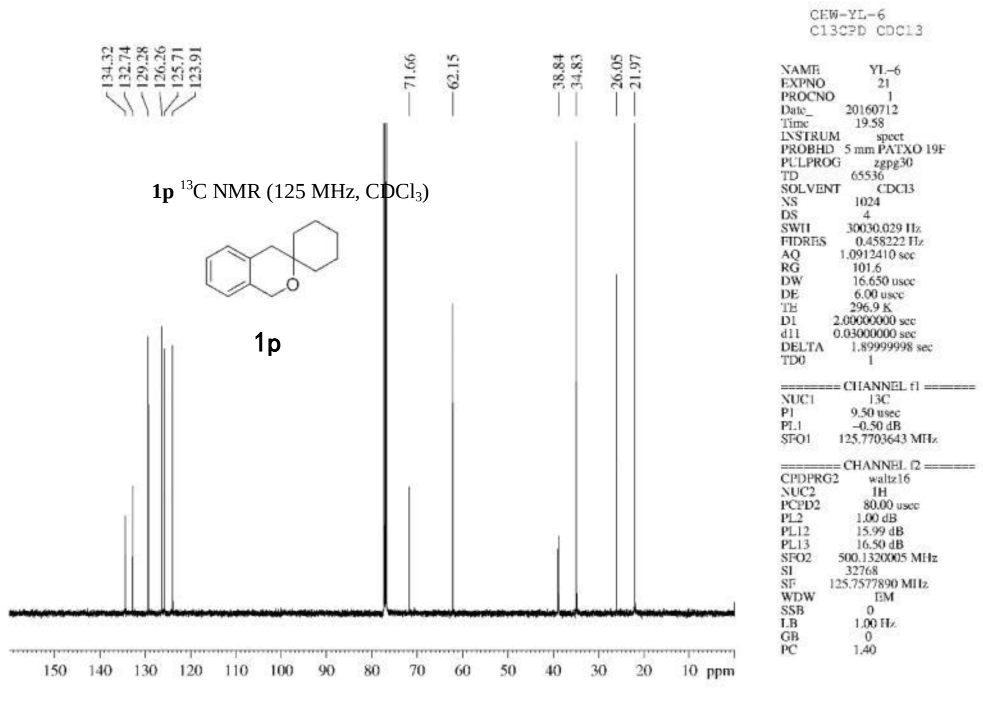

Figure S22. ^{1}H and ^{13}C NMR spectra of **1p**. Related to **Figure 2**.

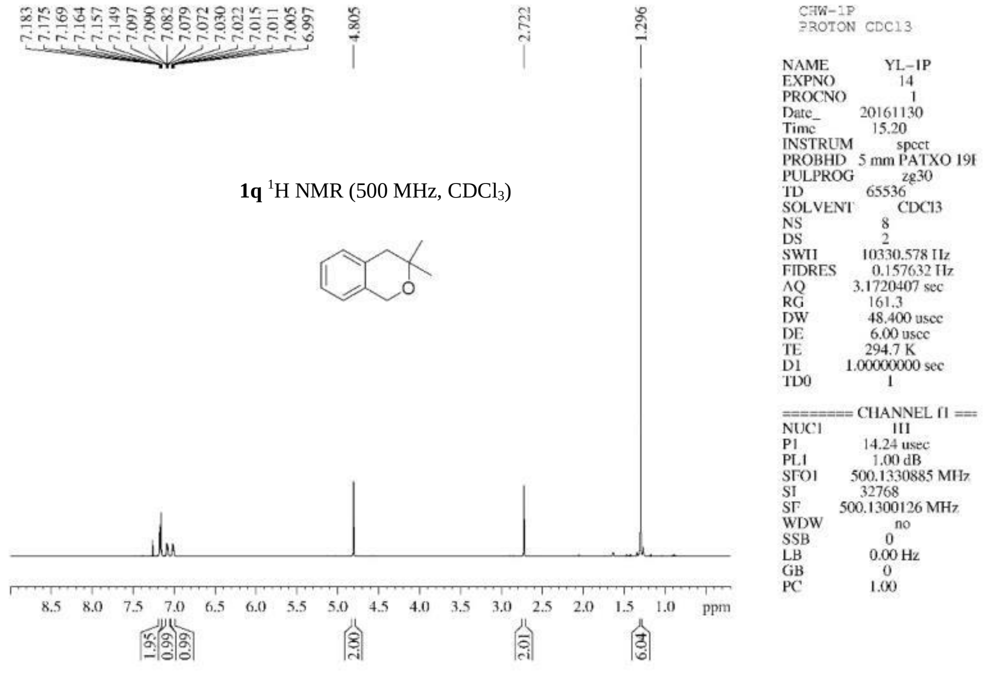

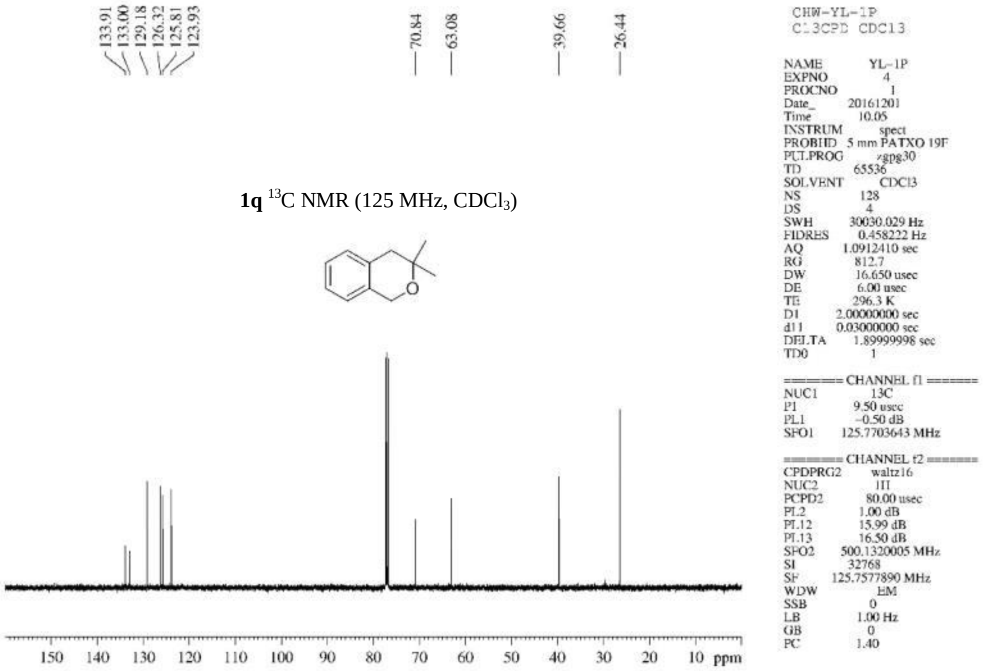

Figure S23. ^{1}H and ^{13}C NMR spectra of **1q**. Related to **Figure 2**.

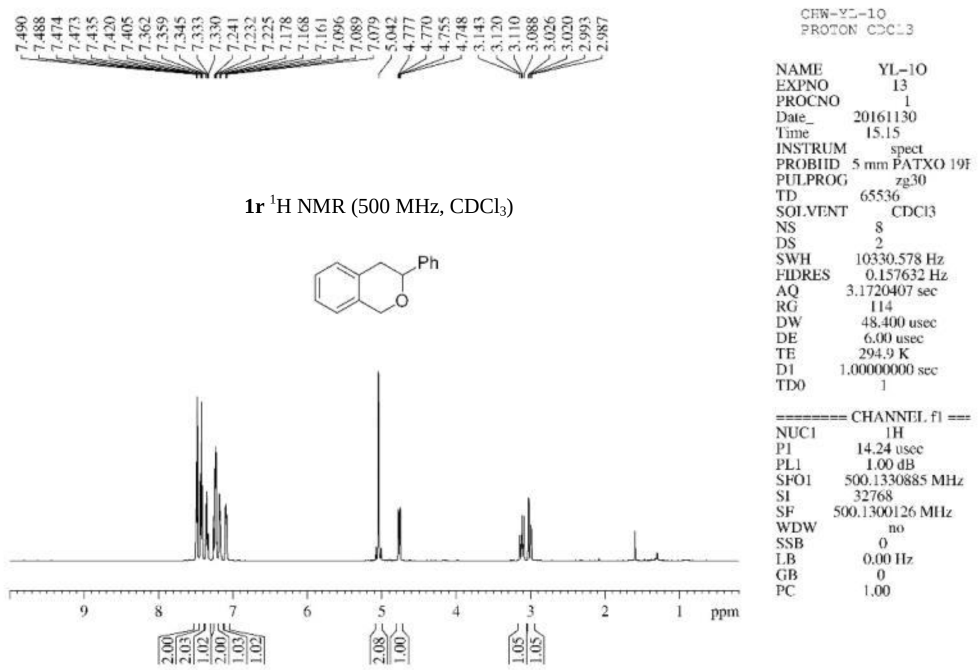

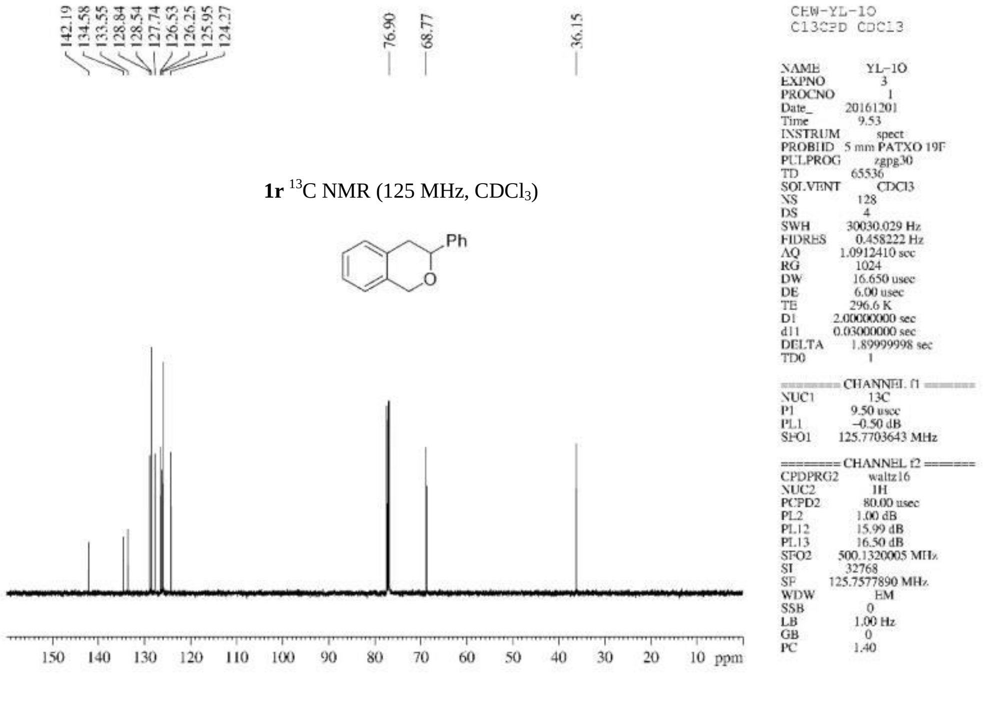

Figure S24. ^{1}H and ^{13}C NMR spectra of **1r**. Related to **Figure 2**.

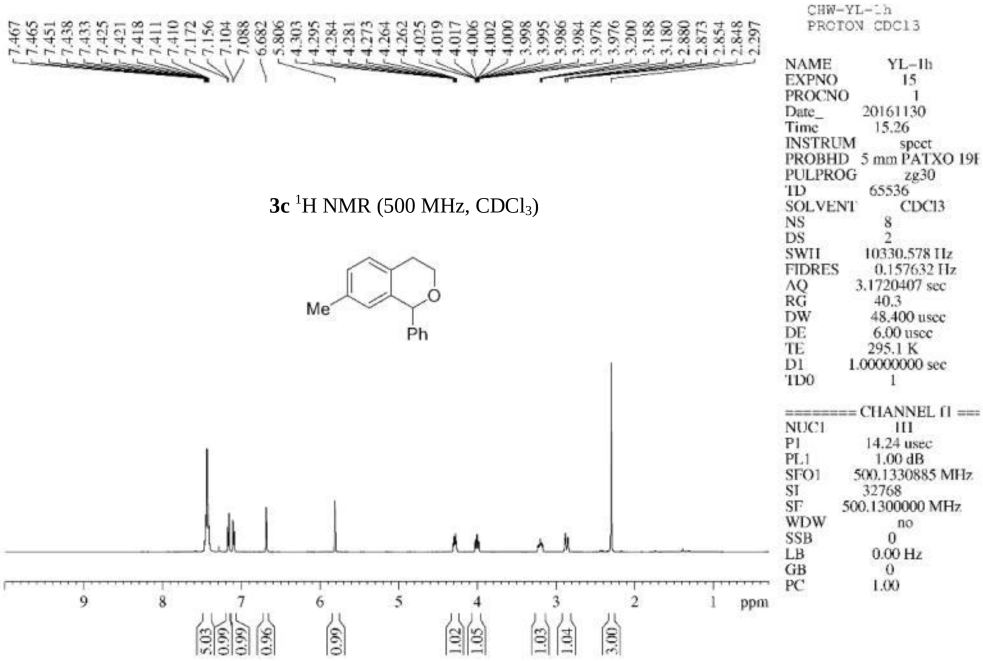

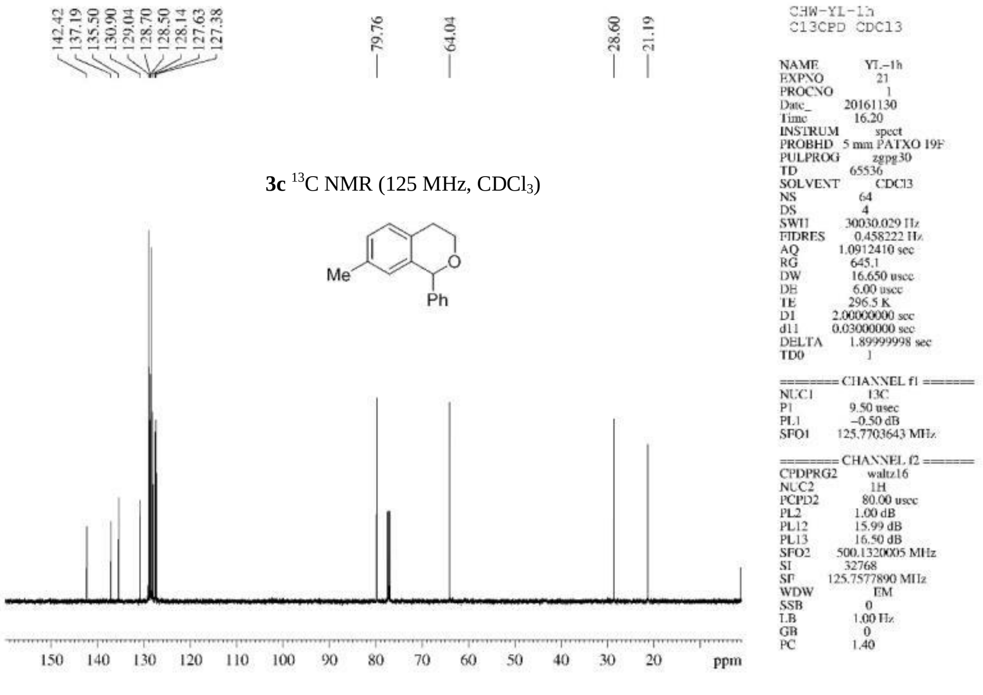

Figure S25. ^{1}H and ^{13}C NMR spectra of **3c**. Related to **Figure 3**.

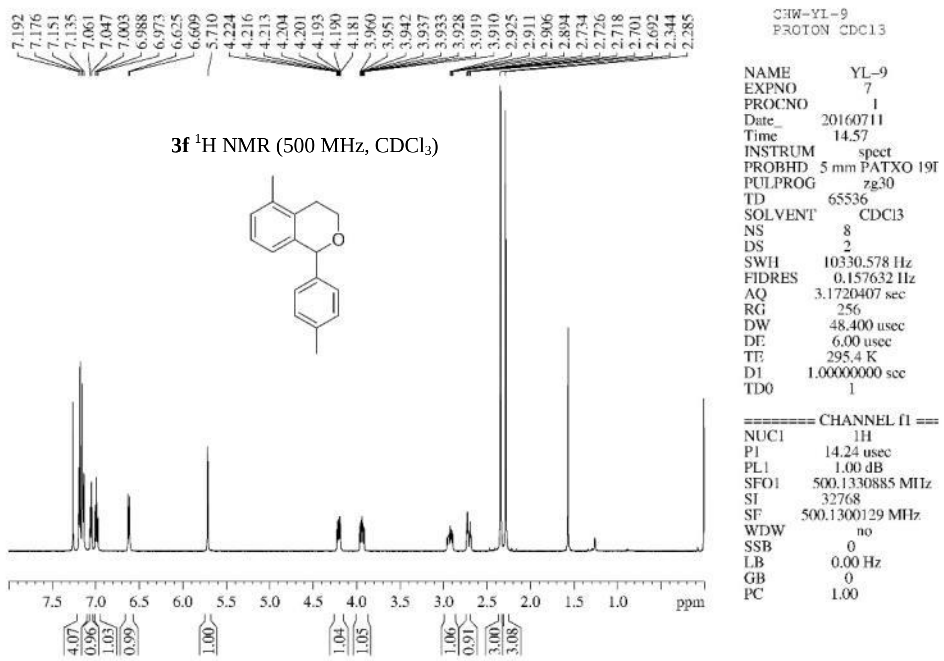

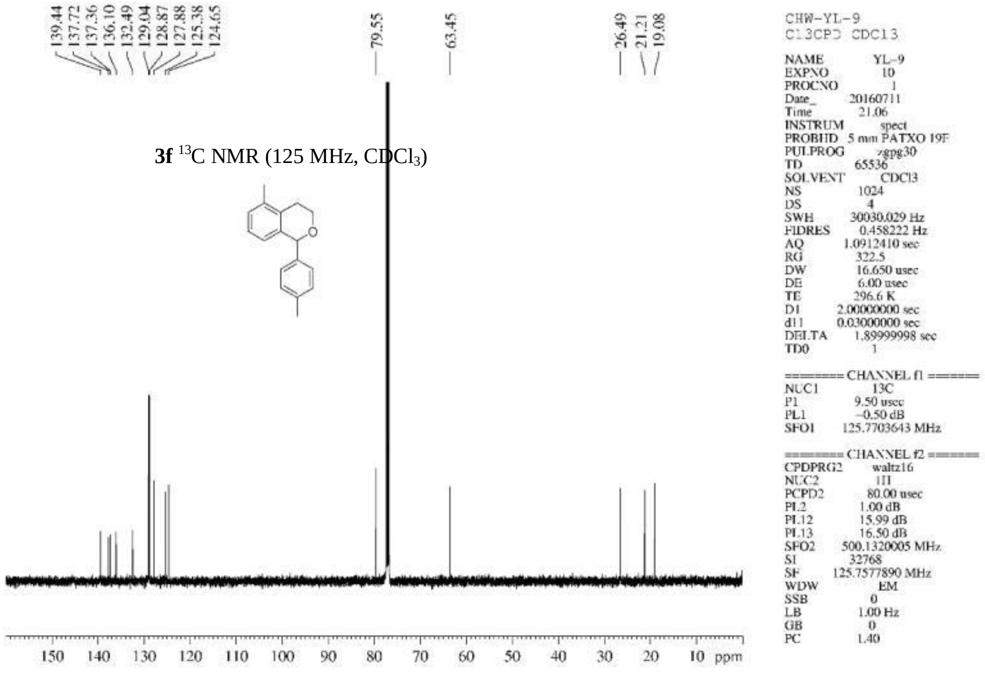

Figure S26. ^{1}H and ^{13}C NMR spectra of **3f**. Related to **Figure 3**.

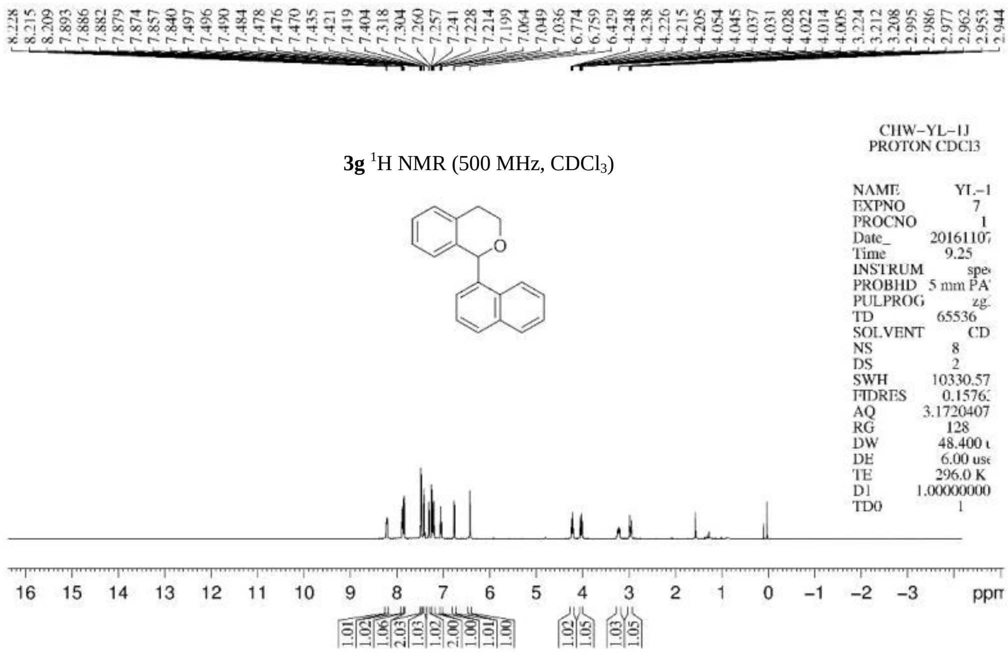

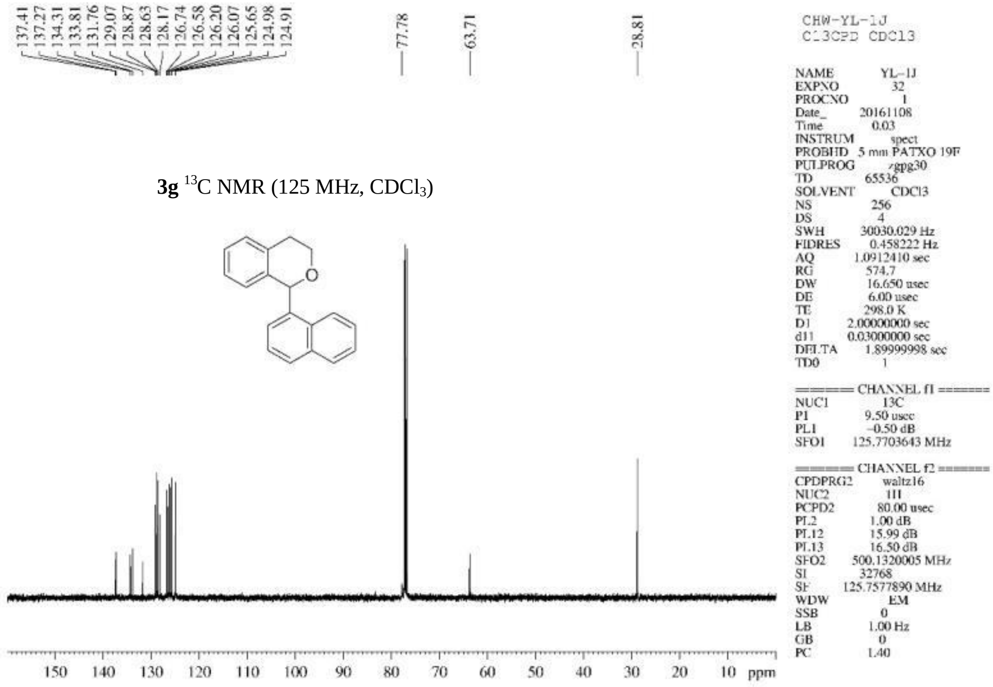

Figure S27. ^{1}H and ^{13}C NMR spectra of **3g**. Related to **Figure 3**.

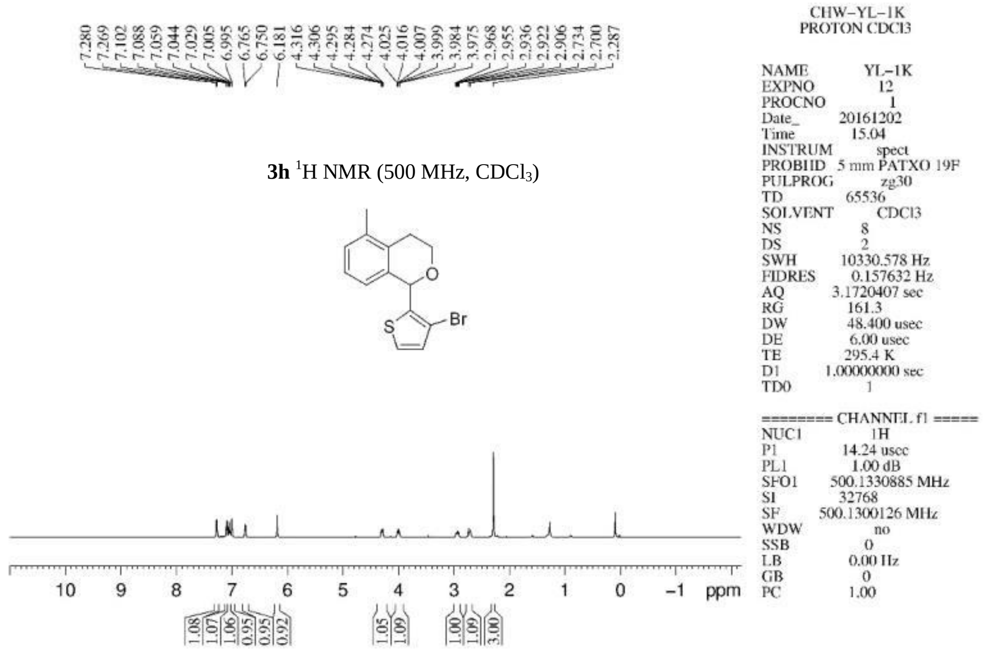

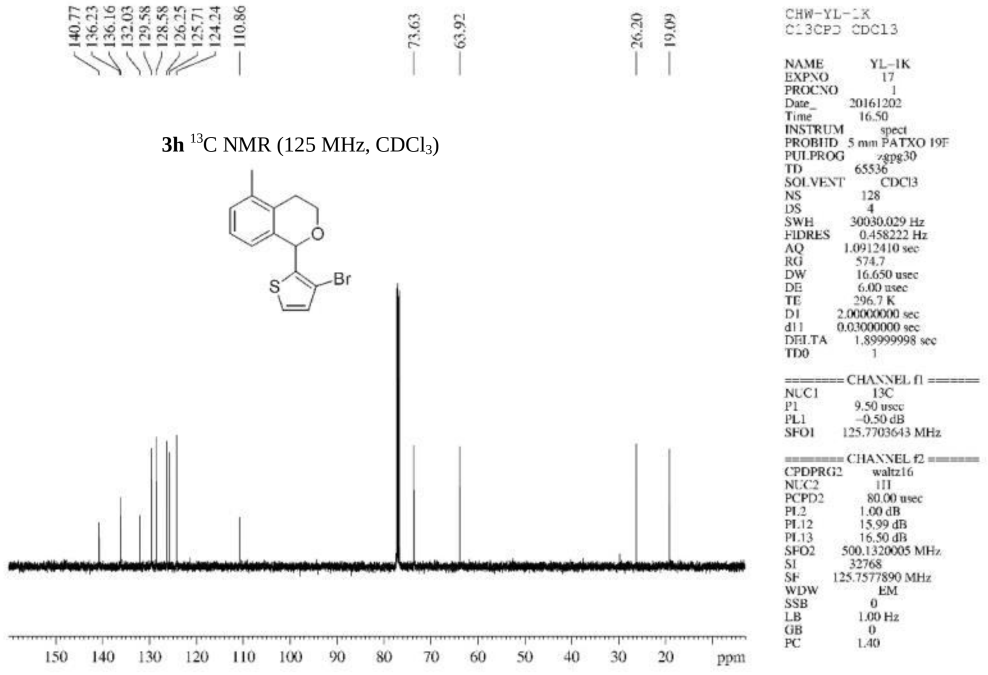

Figure S28. ^{1}H and ^{13}C NMR spectra of **3h**. Related to **Figure 3**.

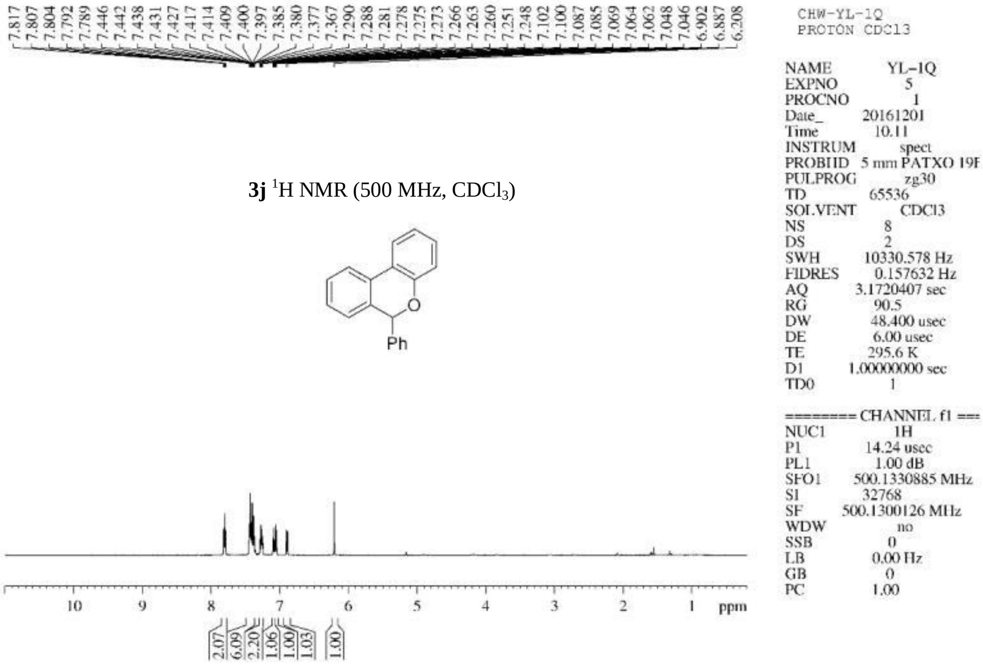

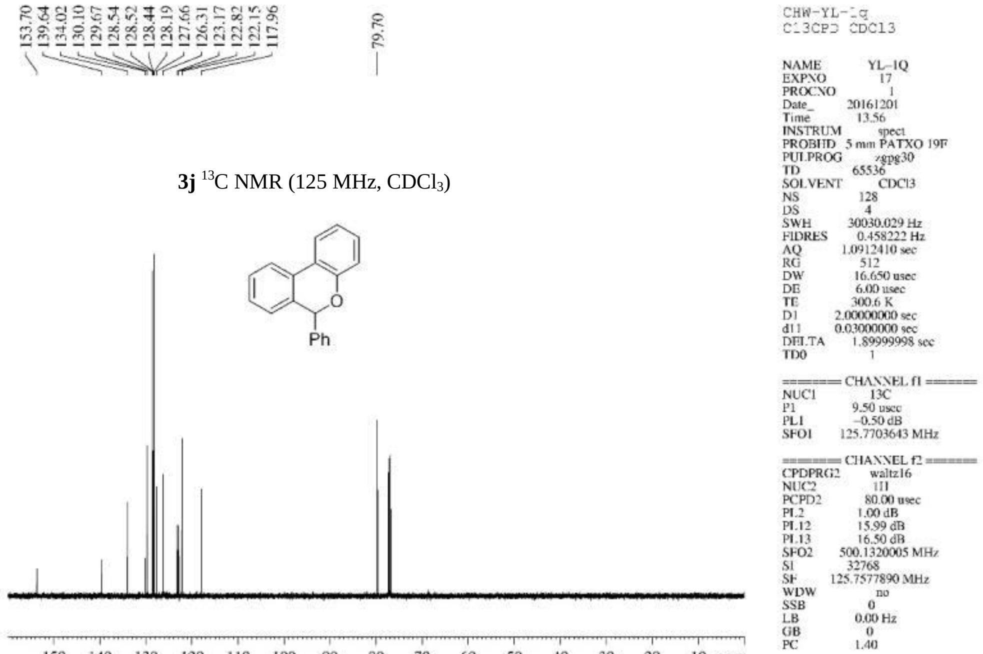

Figure S29. ^{1}H and ^{13}C NMR spectra of **3j**. Related to **Figure 3**.

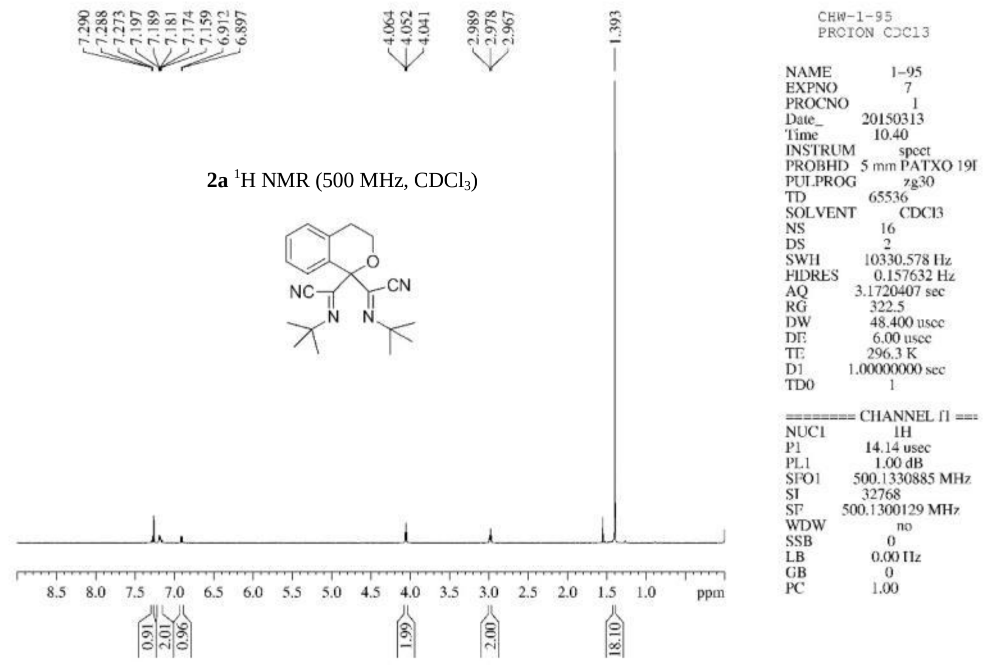

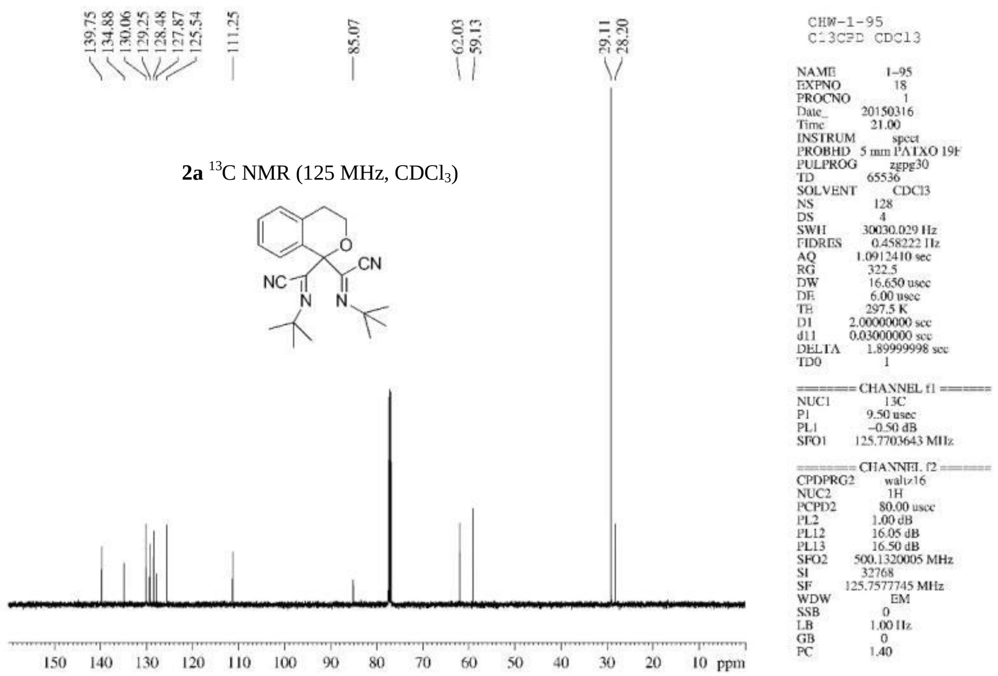

Figure S30. ^{1}H and ^{13}C NMR spectra of **2a.** Related to **Table 1**.

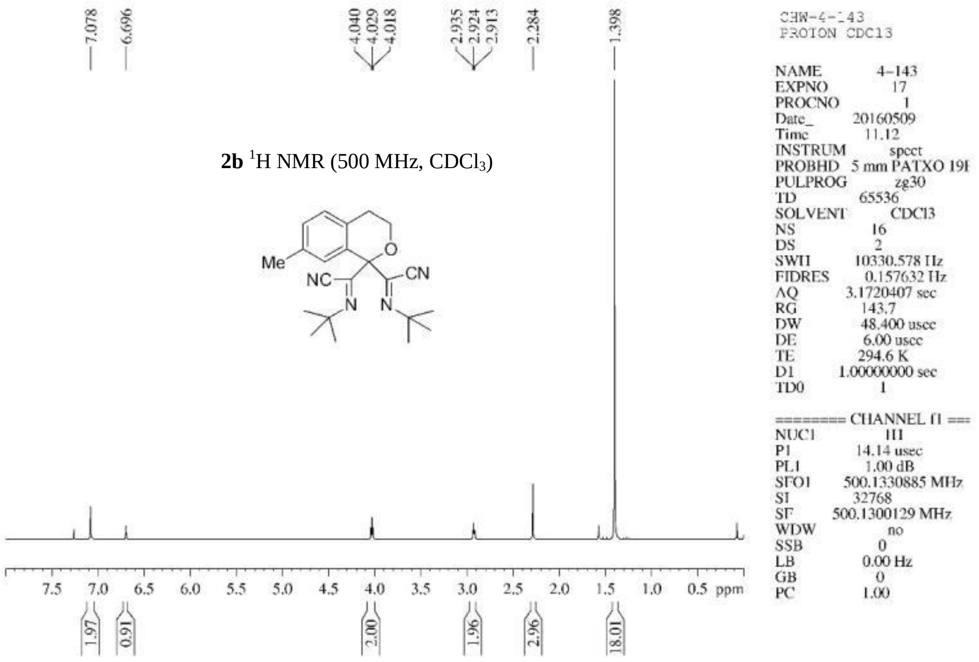

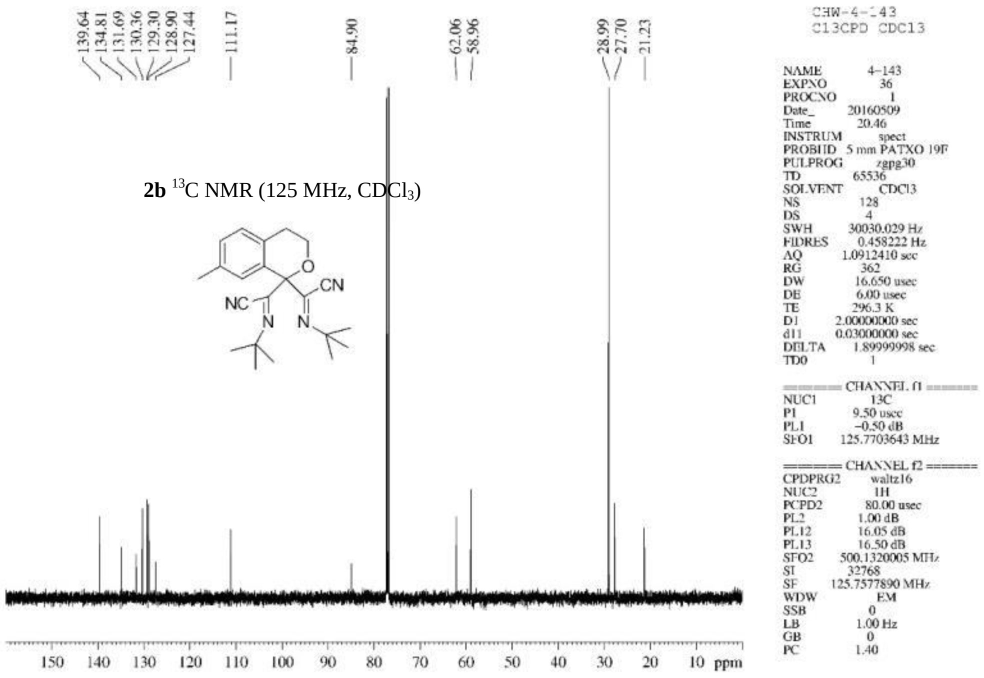

Figure S31. ^{1}H and ^{13}C NMR spectra of **2b**. Related to **Figure 2**.

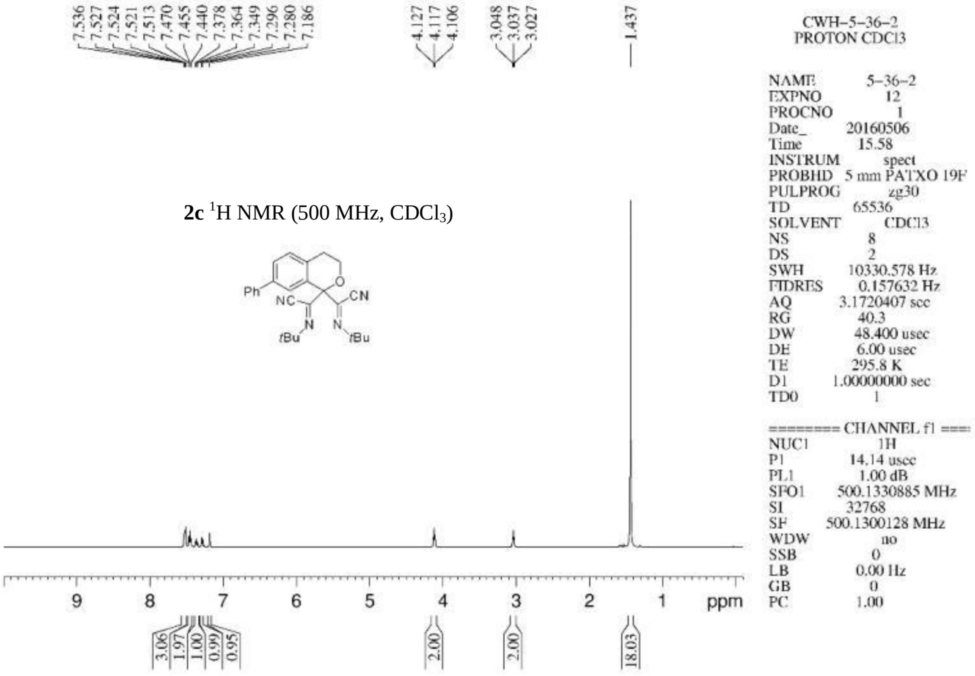

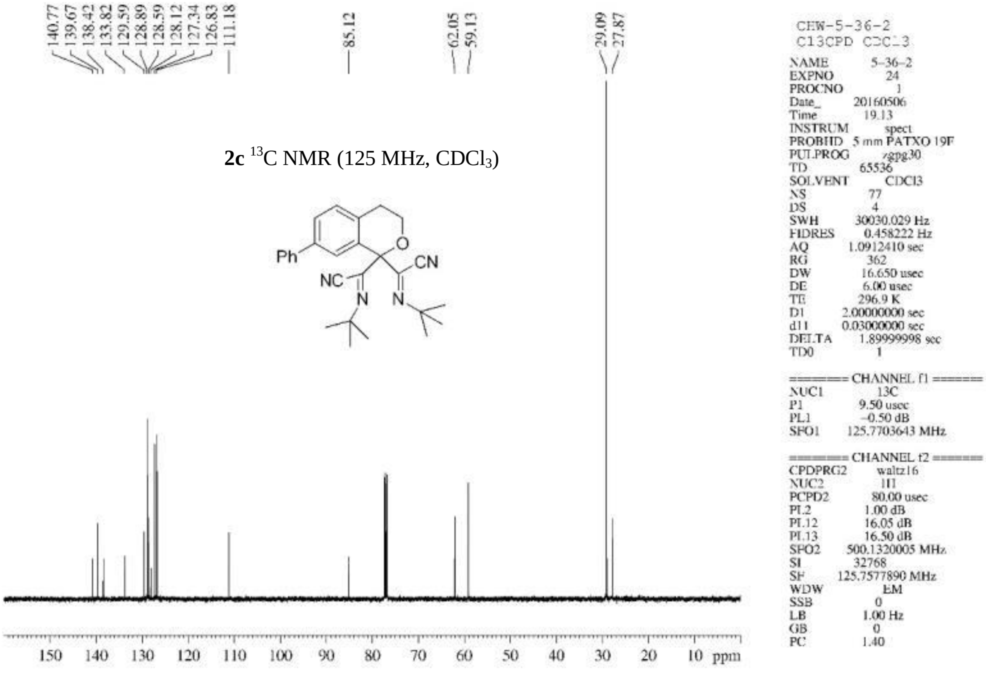

Figure S32. ^{1}H and ^{13}C NMR spectra of **2c**. Related to **Figure 2**.

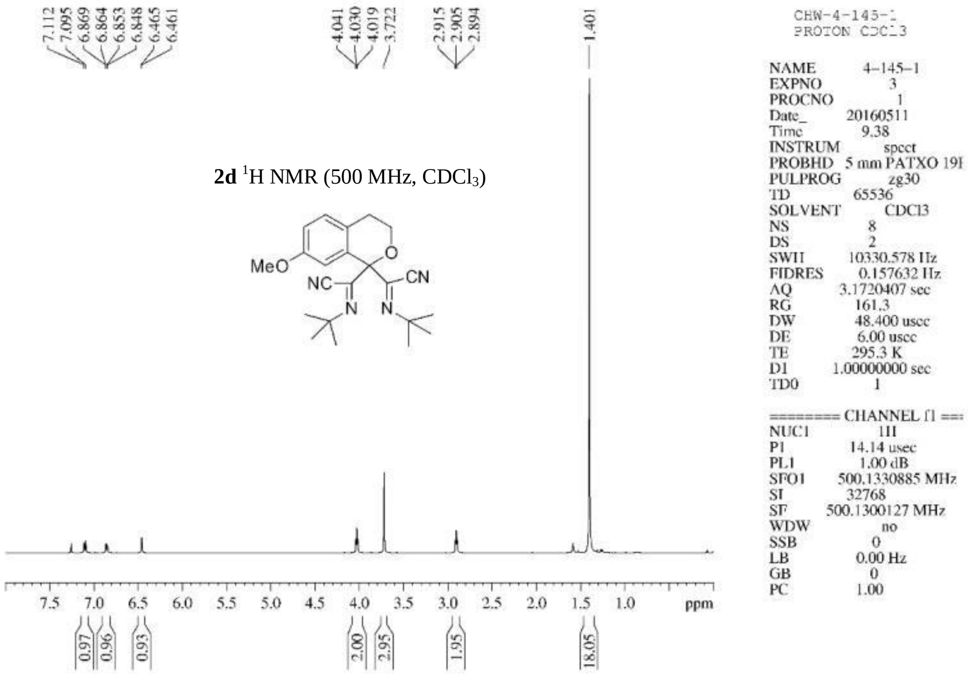

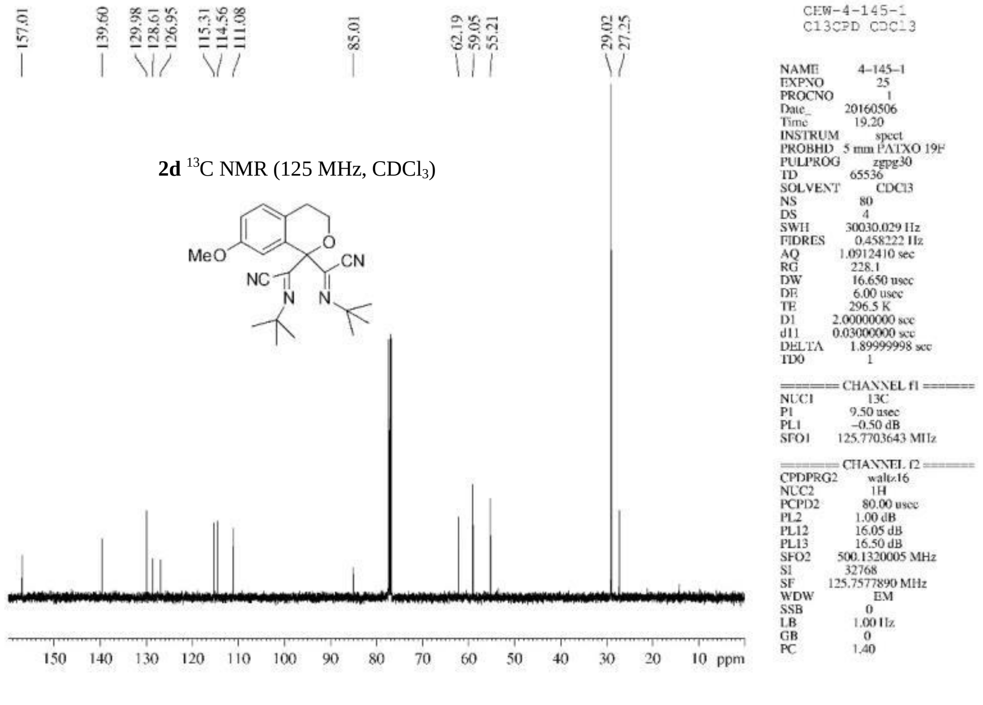

Figure S33. ^{1}H and ^{13}C NMR spectra of **2d**. Related to **Figure 2**.

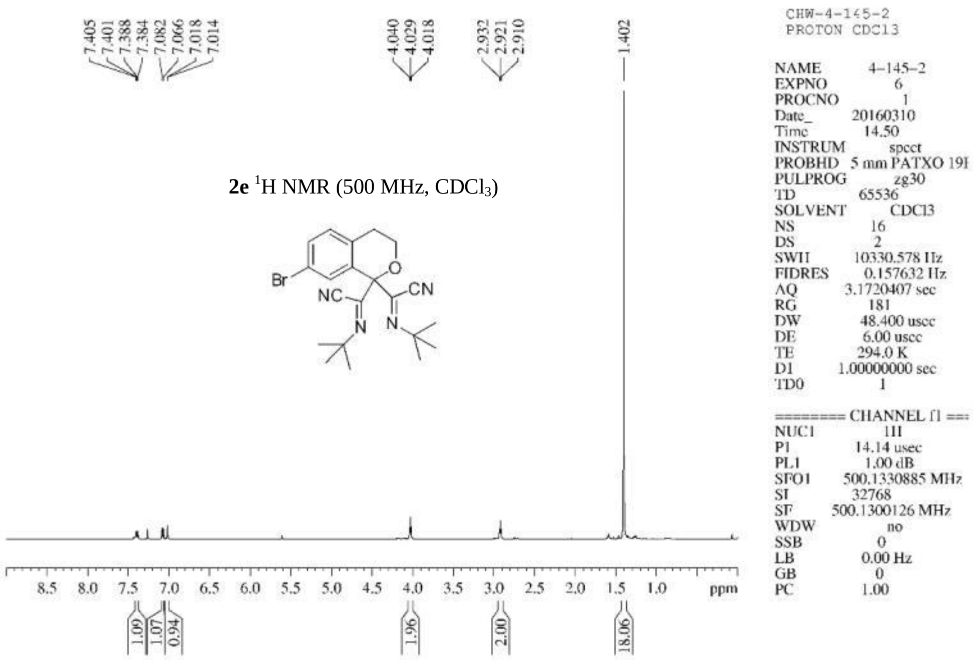

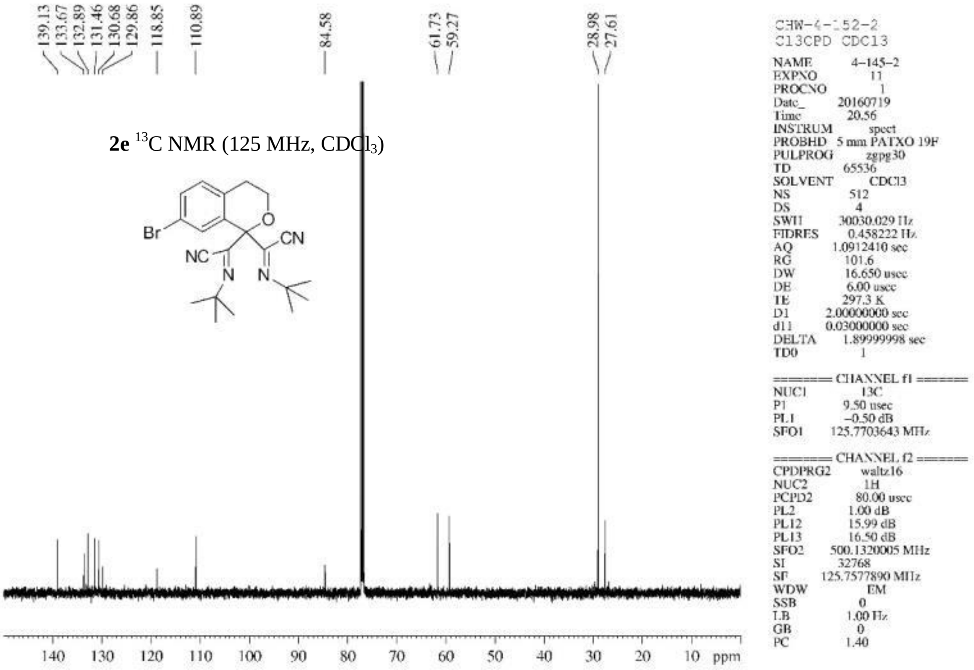

Figure S34. ^{1}H and ^{13}C NMR spectra of **2e**. Related to **Figure 2**.

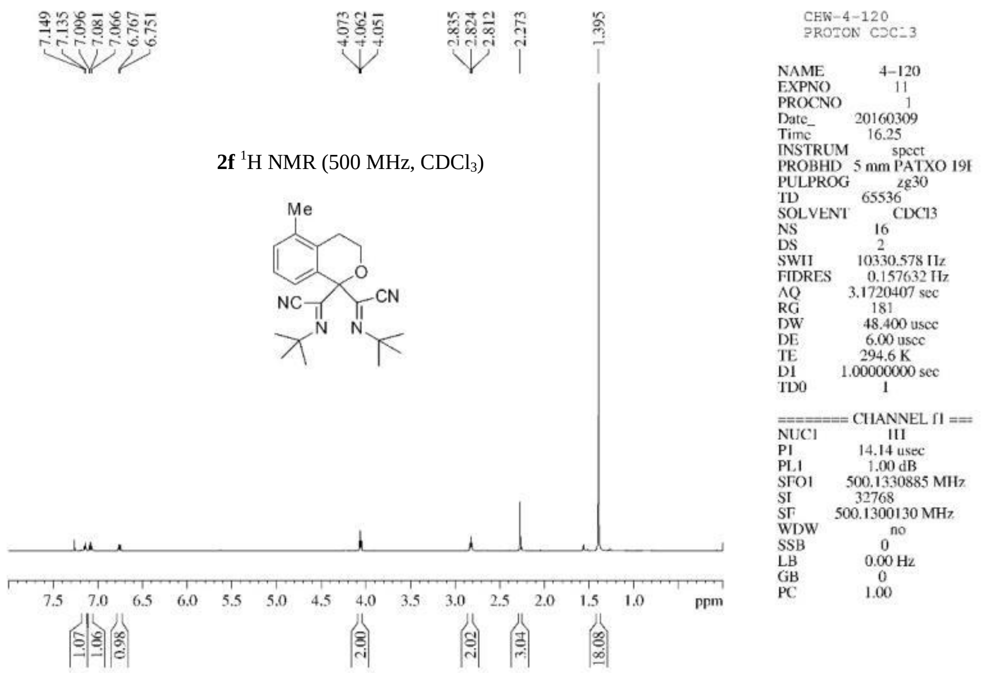

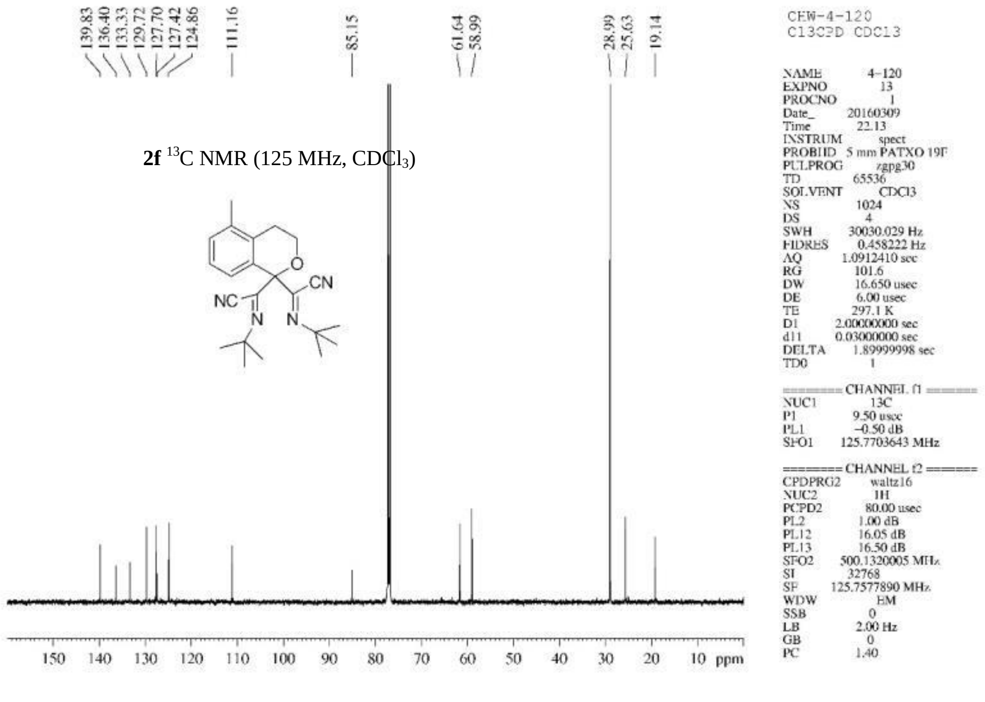

Figure S35. ^{1}H and ^{13}C NMR spectra of **2f**. Related to **Figure 2**.

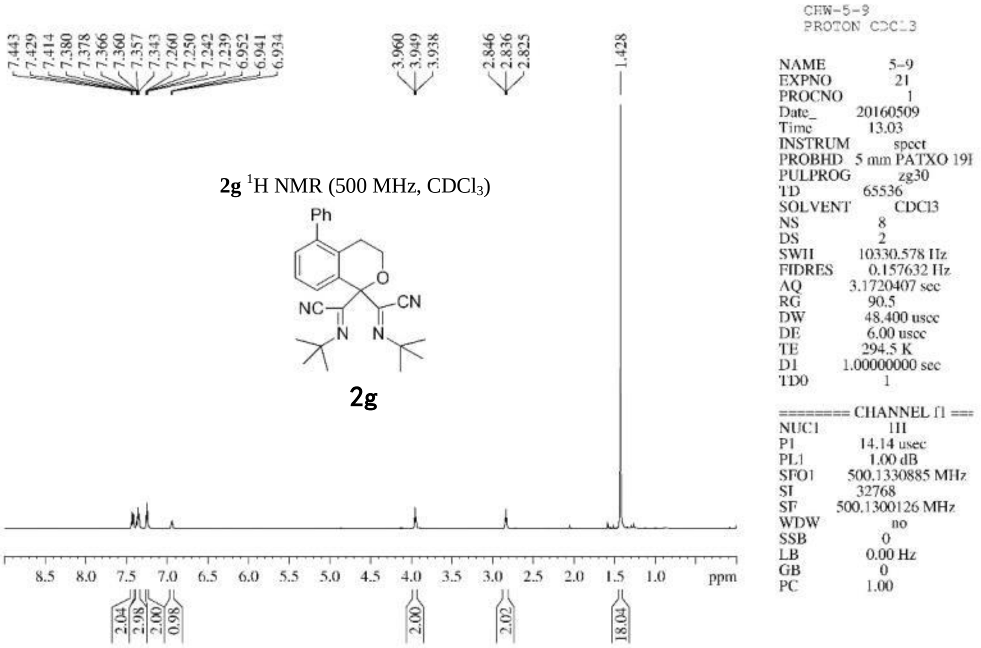

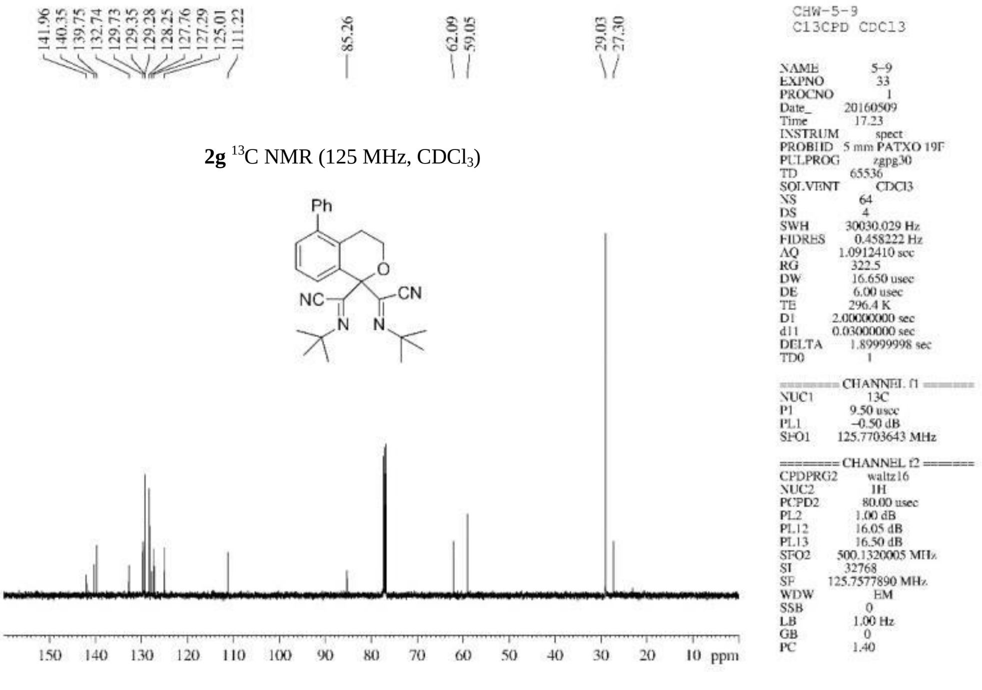

Figure S36. ^{1}H and ^{13}C NMR spectra of **2g**. Related to **Figure 2**.

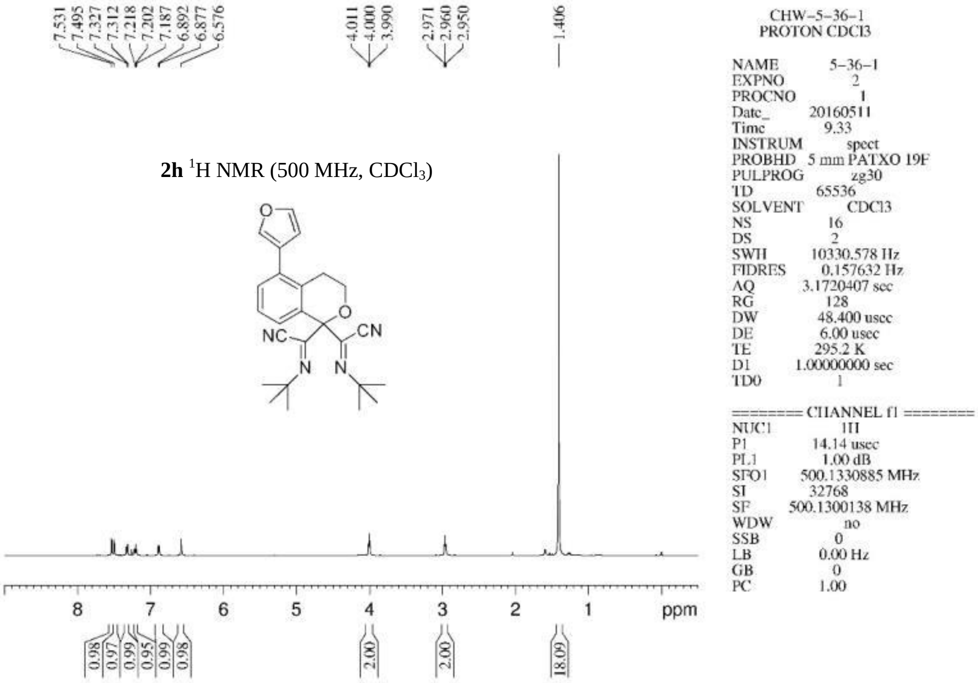

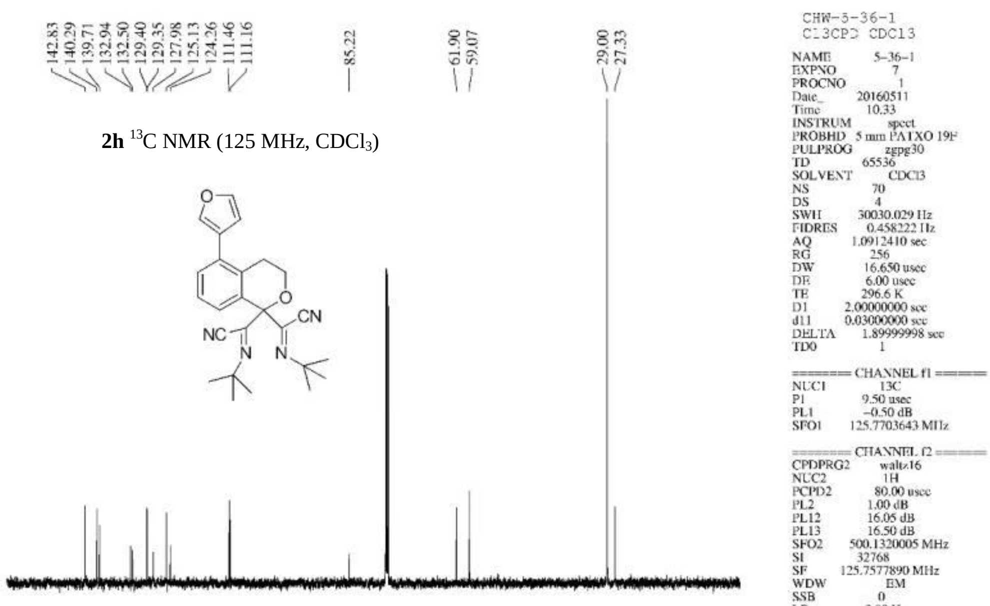

Figure S37. ^{1}H and ^{13}C NMR spectra of **2h**. Related to **Figure 2**.

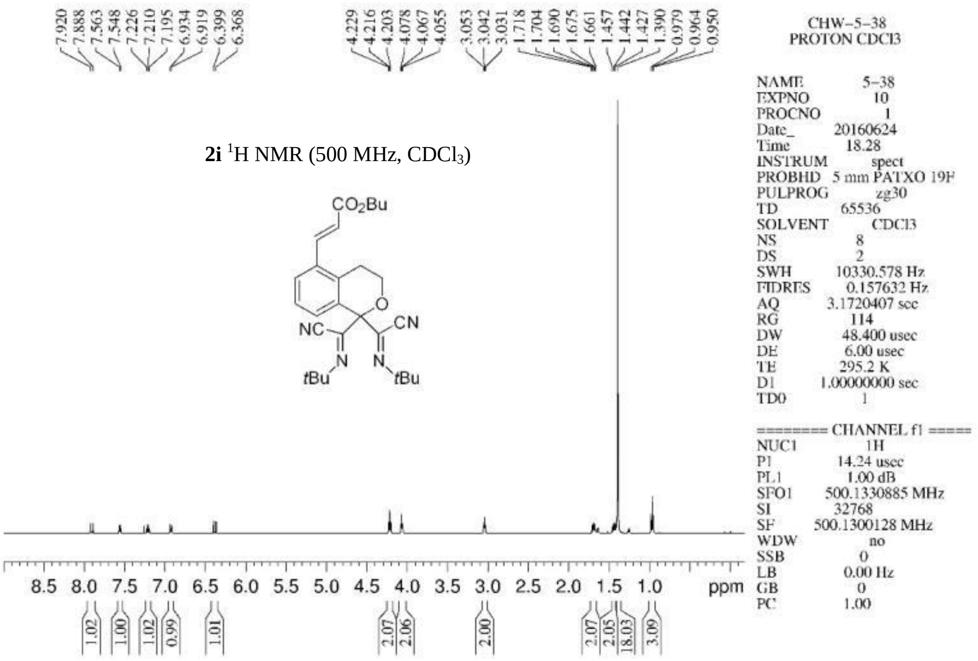

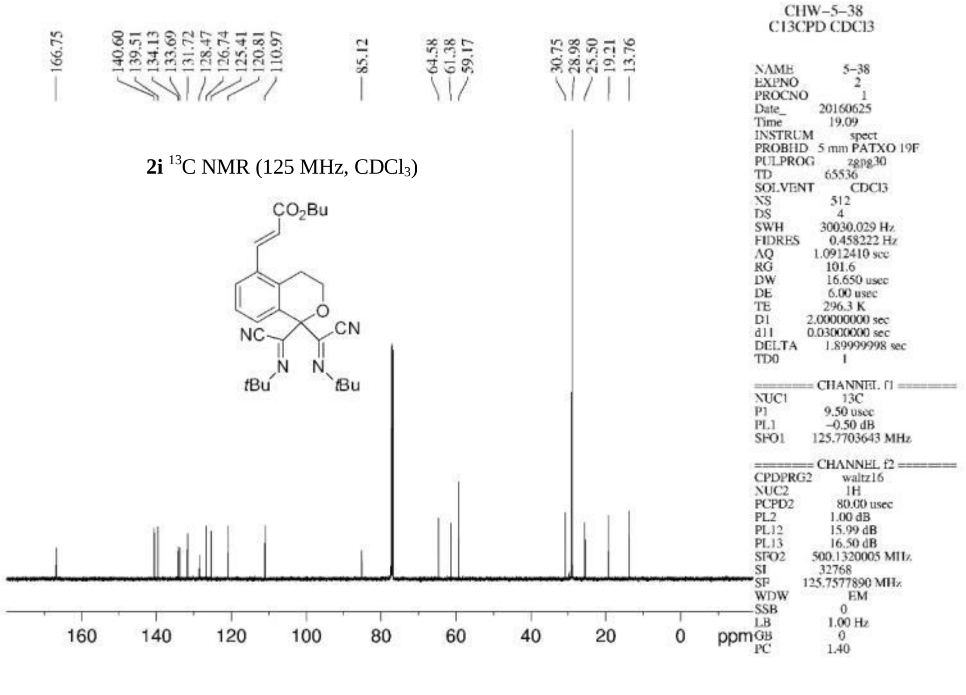

Figure S38. ^{1}H and ^{13}C NMR spectra of **2i.** Related to **Figure 2**.

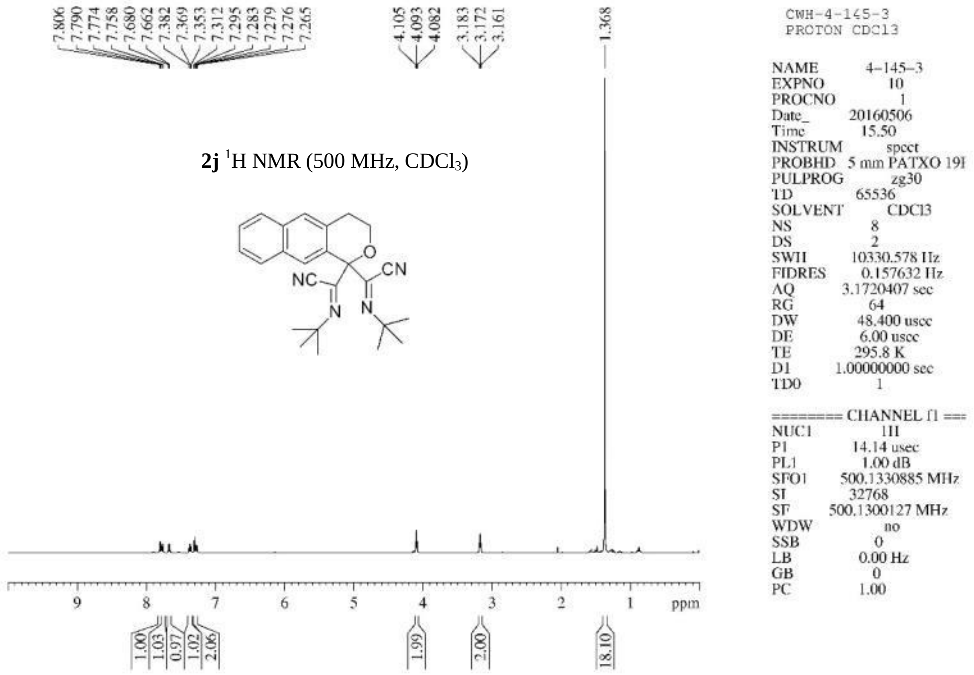

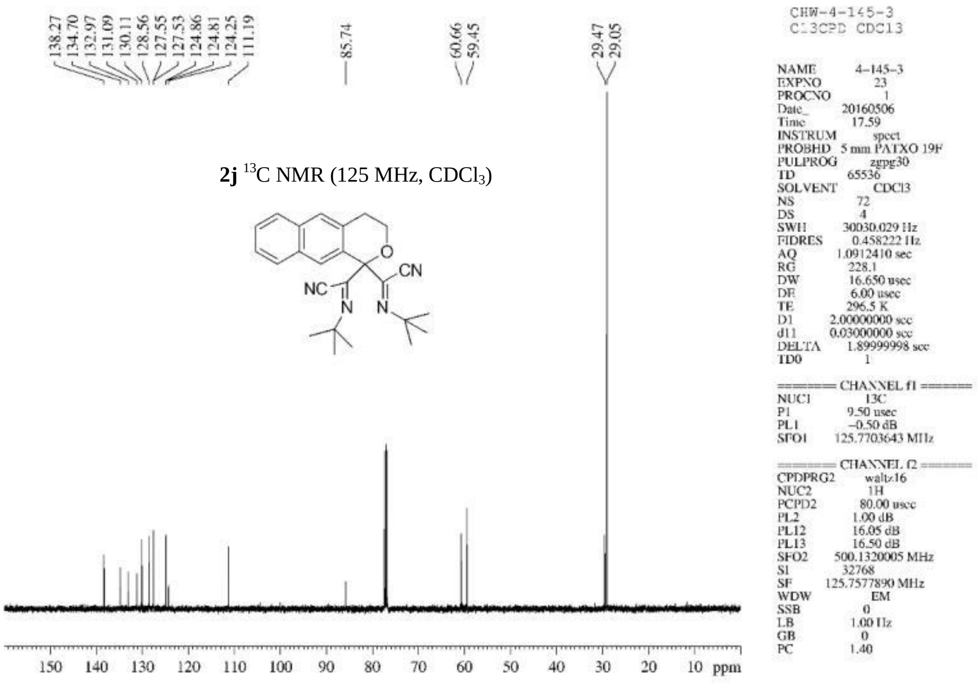

Figure S39. ^{1}H and ^{13}C NMR spectra of **2j**. Related to **Figure 2**.

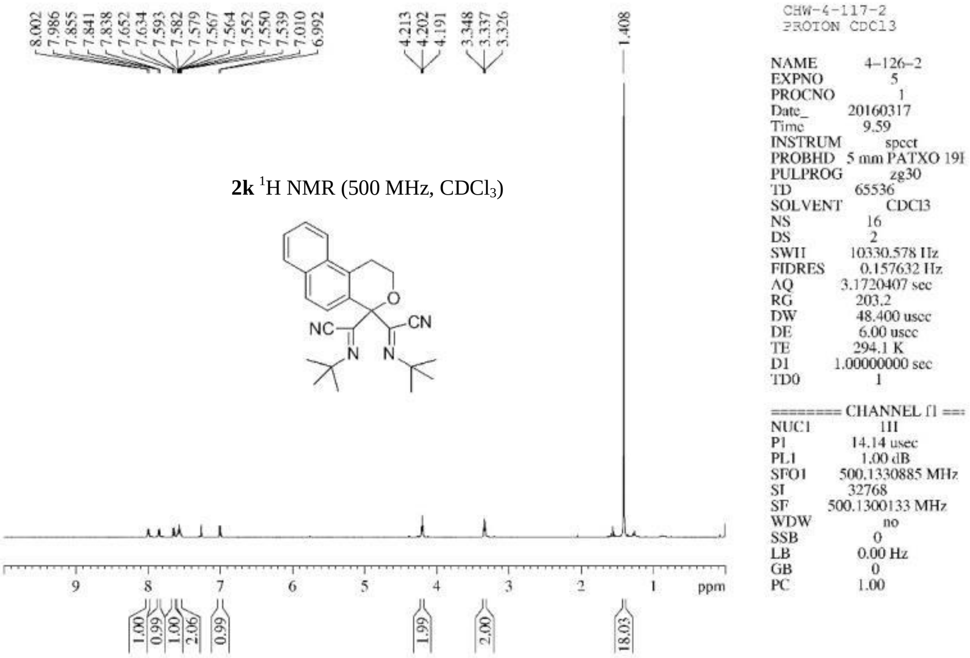

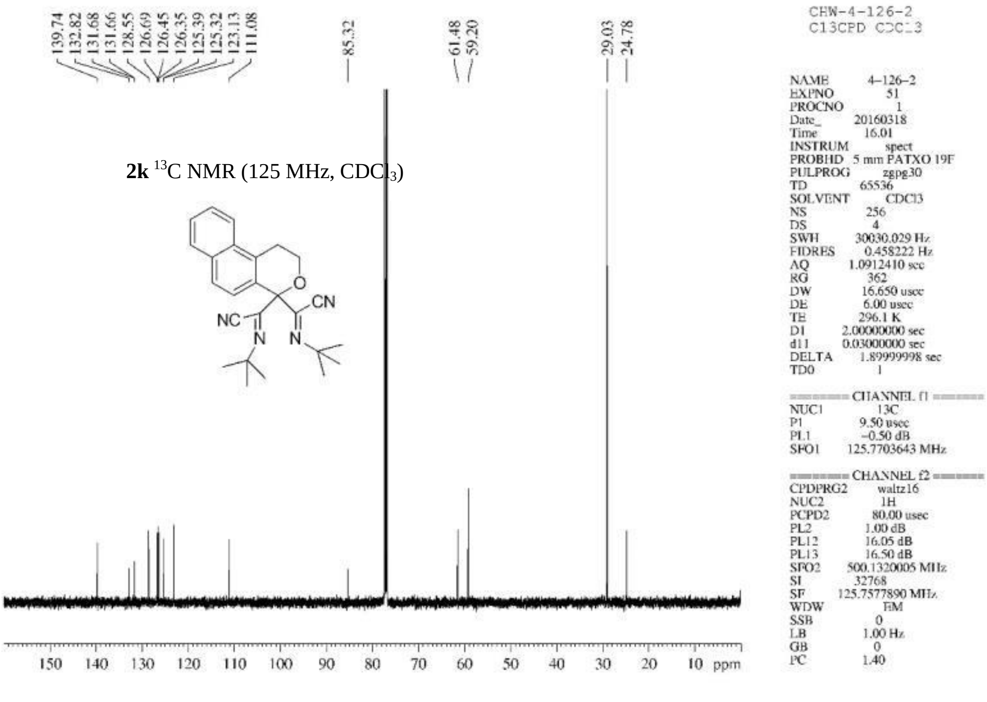

Figure S40. ^{1}H and ^{13}C NMR spectra of **2k**. Related to **Figure 2**.

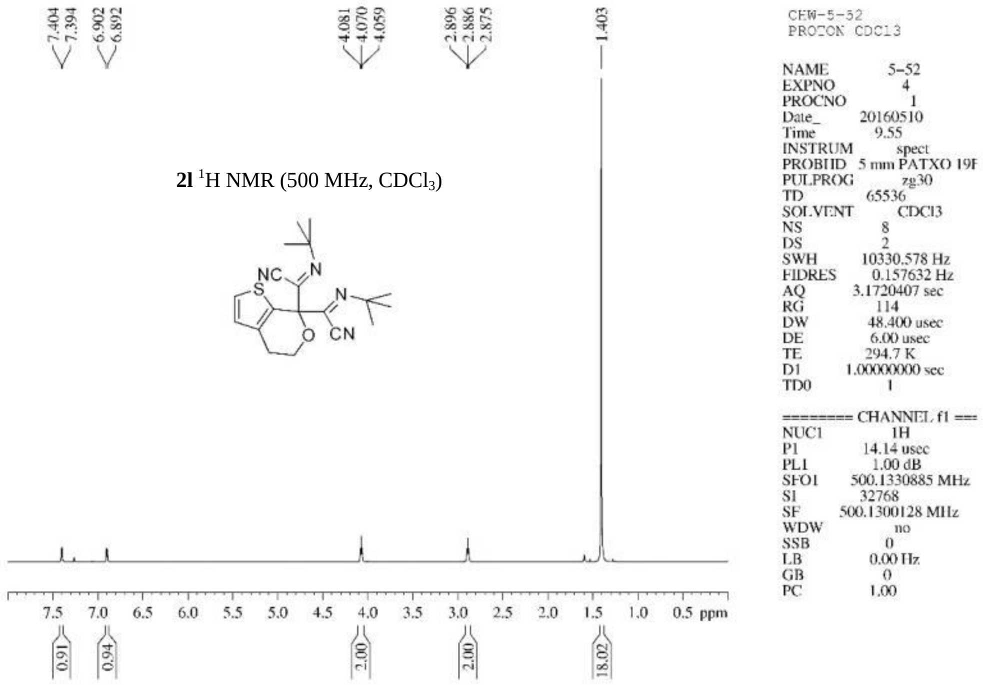

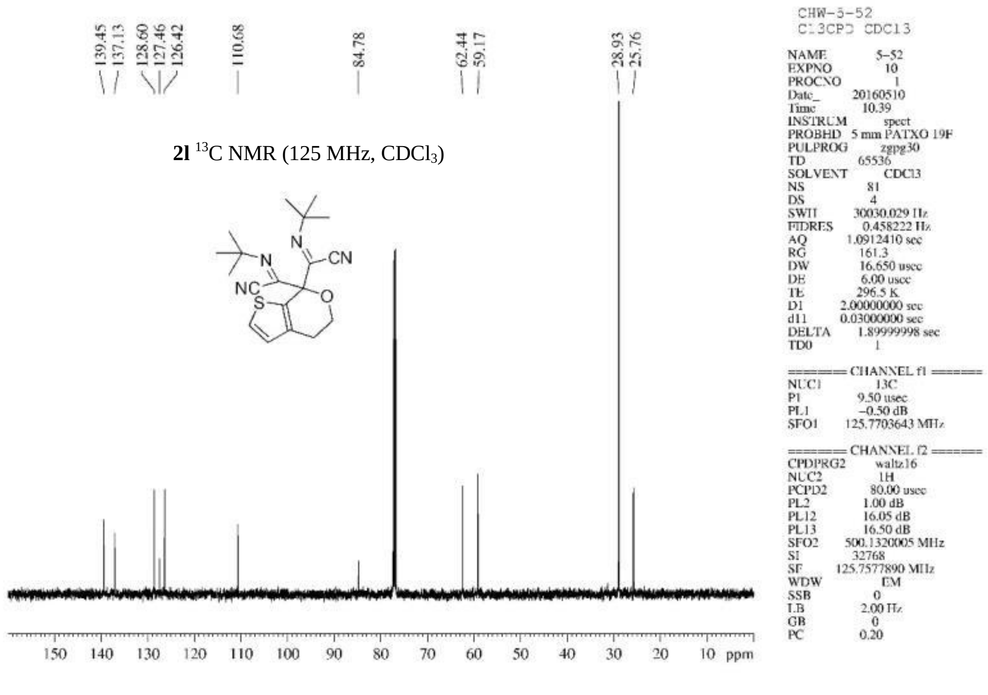

Figure S41. ^{1}H and ^{13}C NMR spectra of **2l**. Related to **Figure 2**.

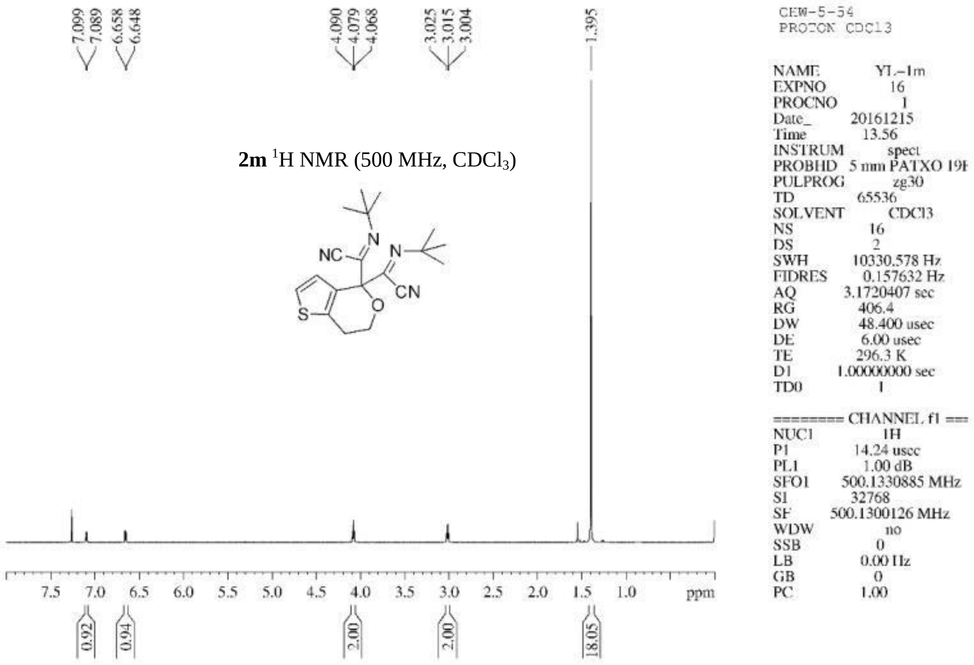

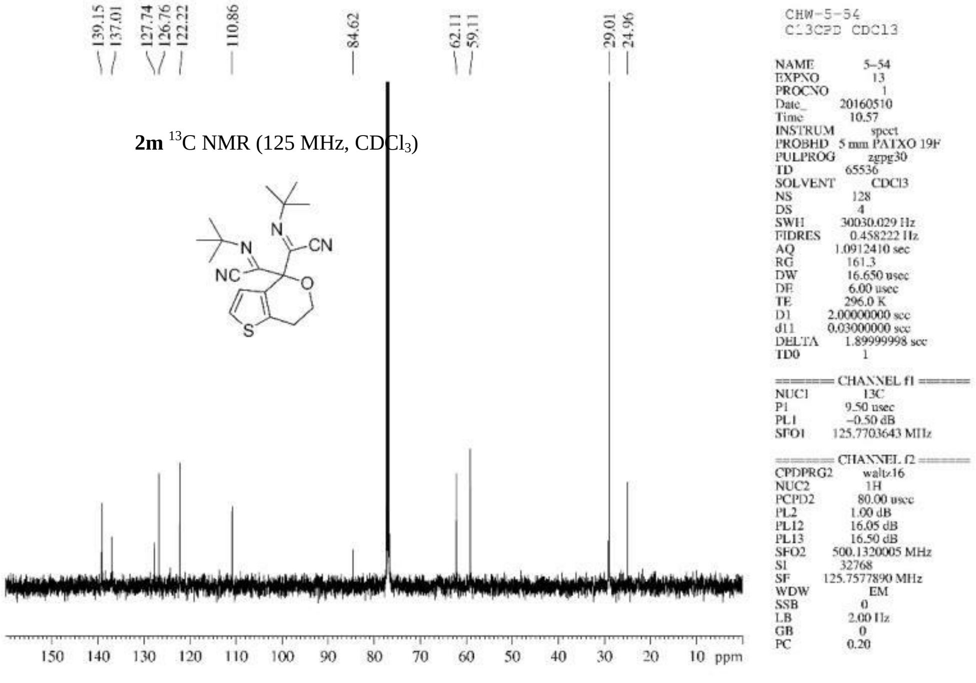

Figure S42. ^{1}H and ^{13}C NMR spectra of **2m**. Related to **Figure 2**.

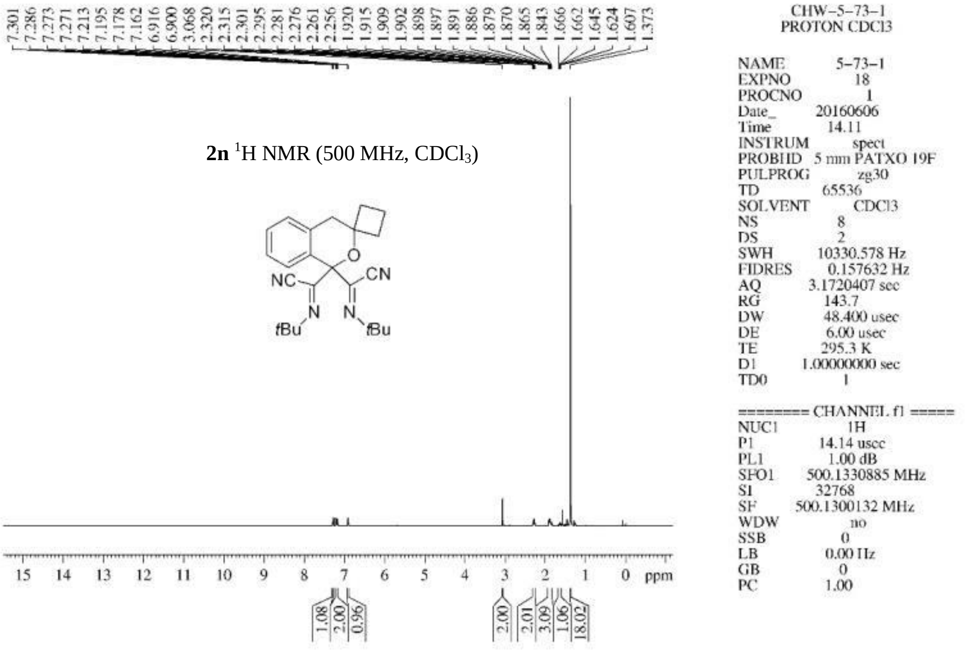

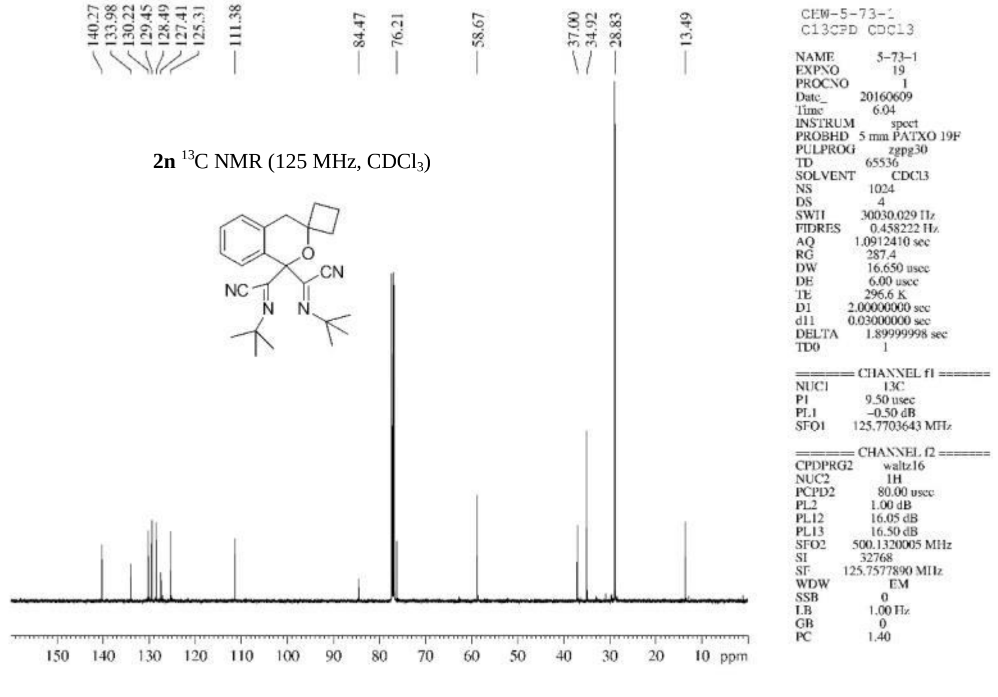

Figure S43. ^{1}H and ^{13}C NMR spectra of **2n**. Related to **Figure 2**.

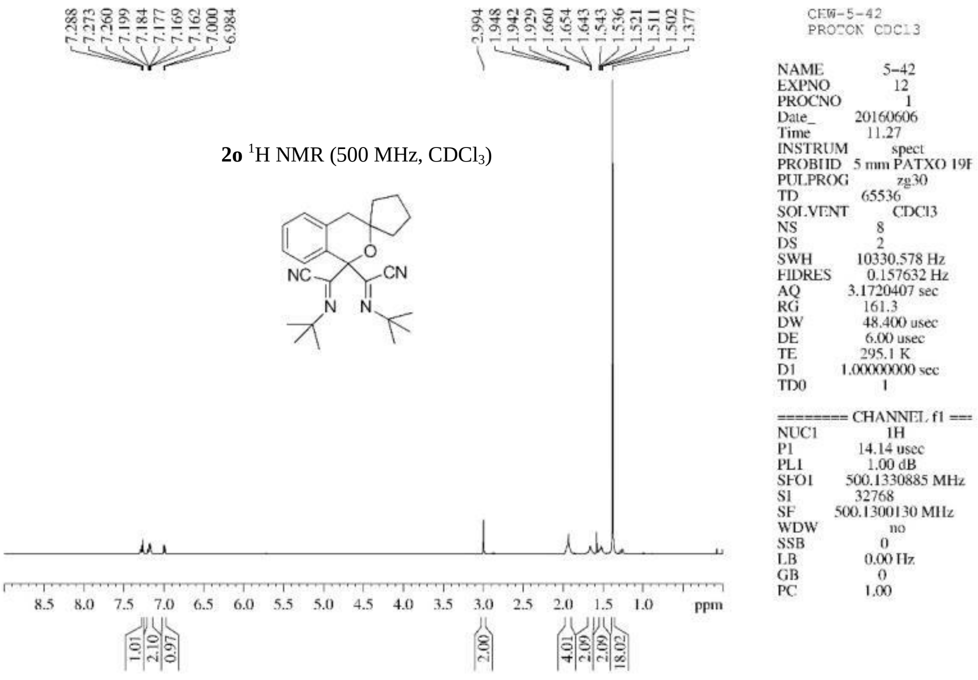

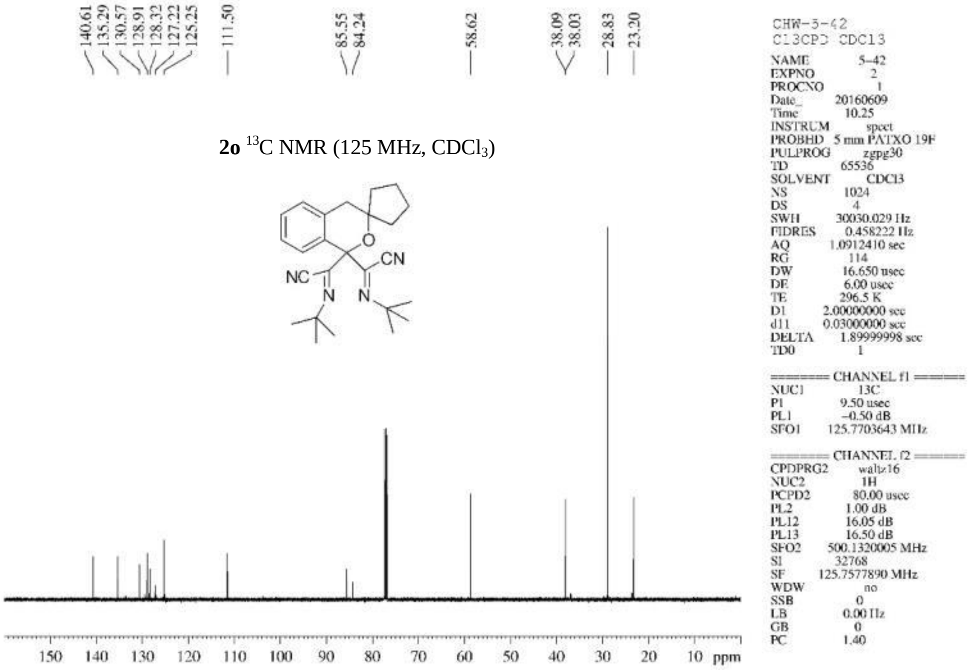

Figure S44. ${}^{1}H$ and ${}^{13}C$ NMR spectra of **2o**. Related to **Figure 2**.

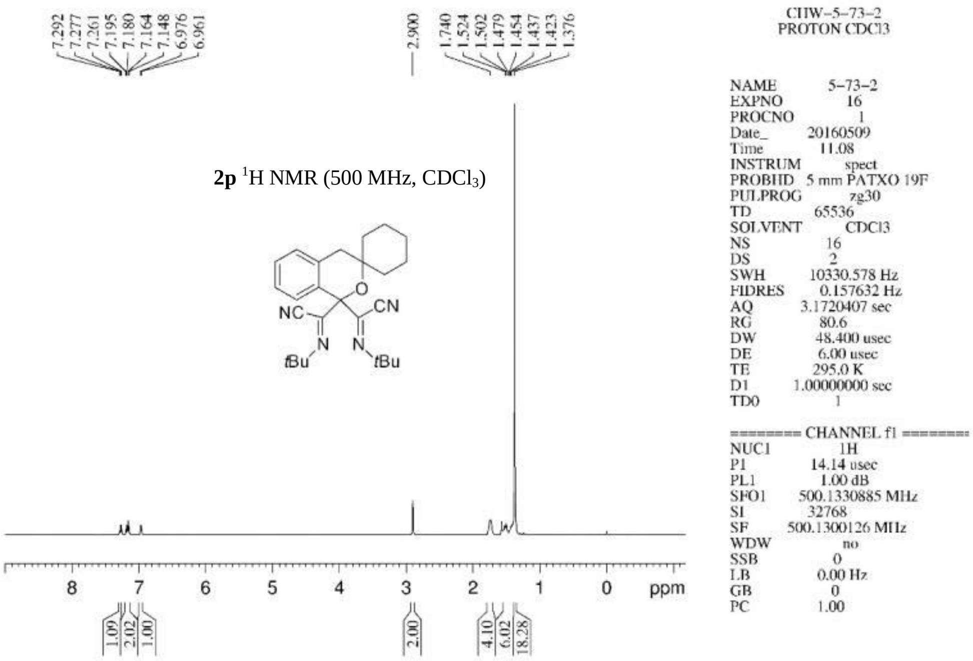

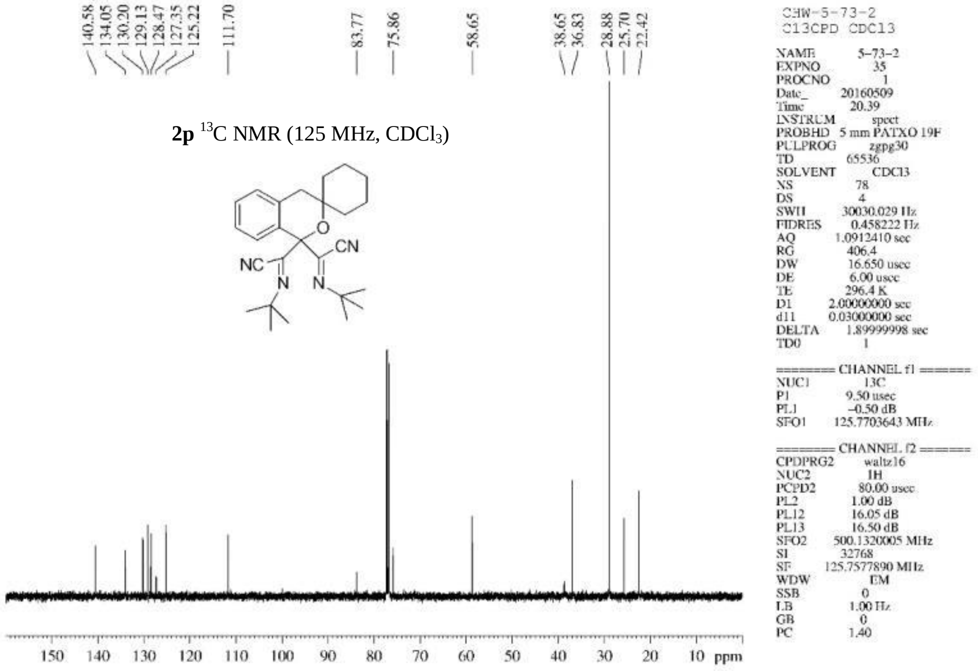

Figure S45. ^{1}H and ^{13}C NMR spectra of **2p**. Related to **Figure 2**.

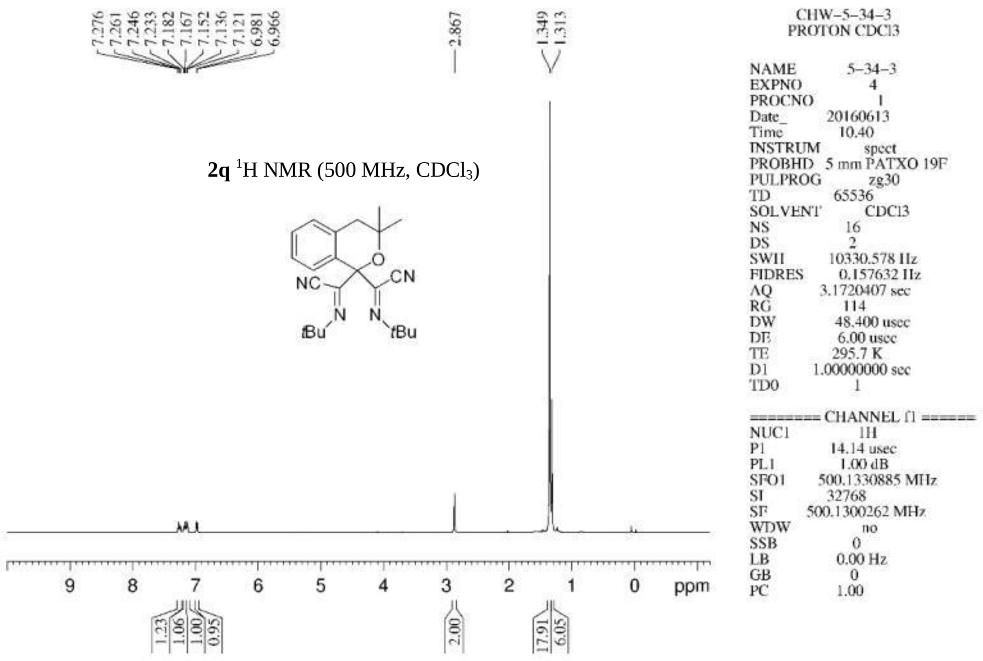

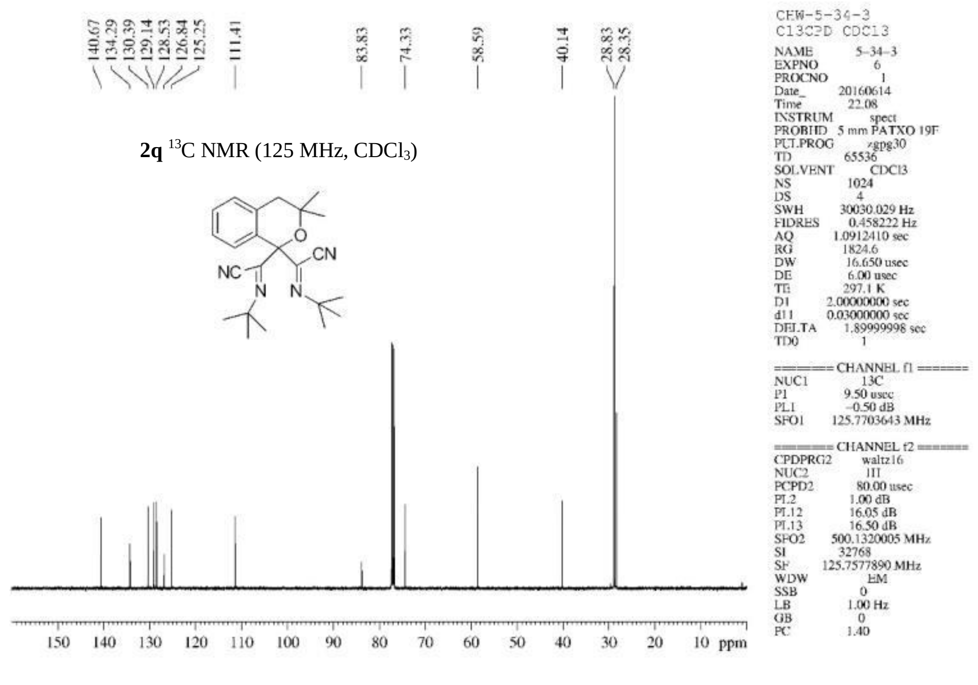

Figure S46. ^{1}H and ^{13}C NMR spectra of **2q**. Related to **Figure 2**.

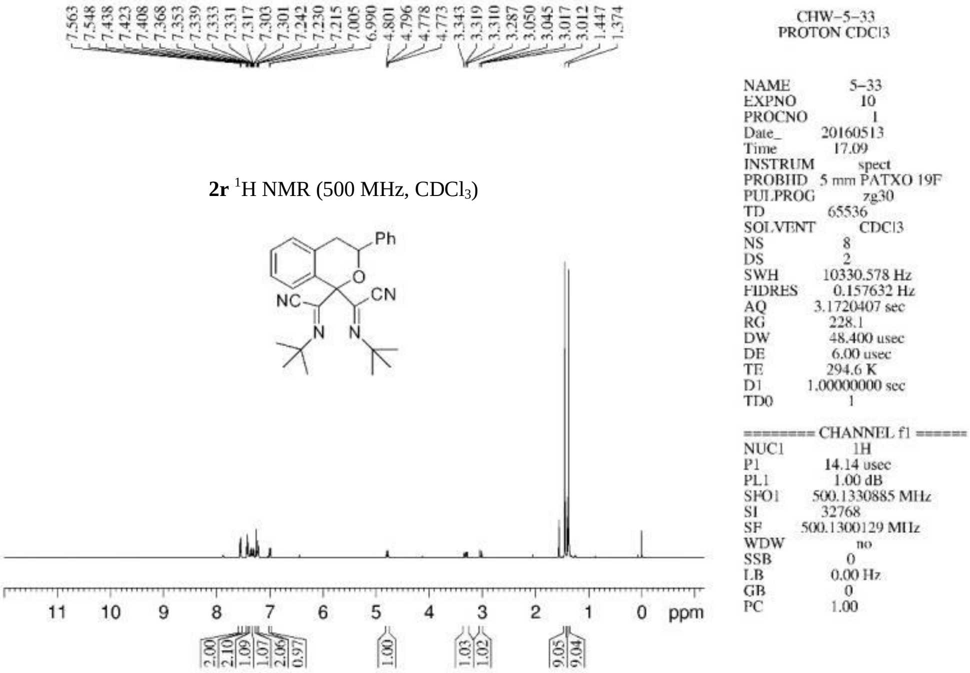

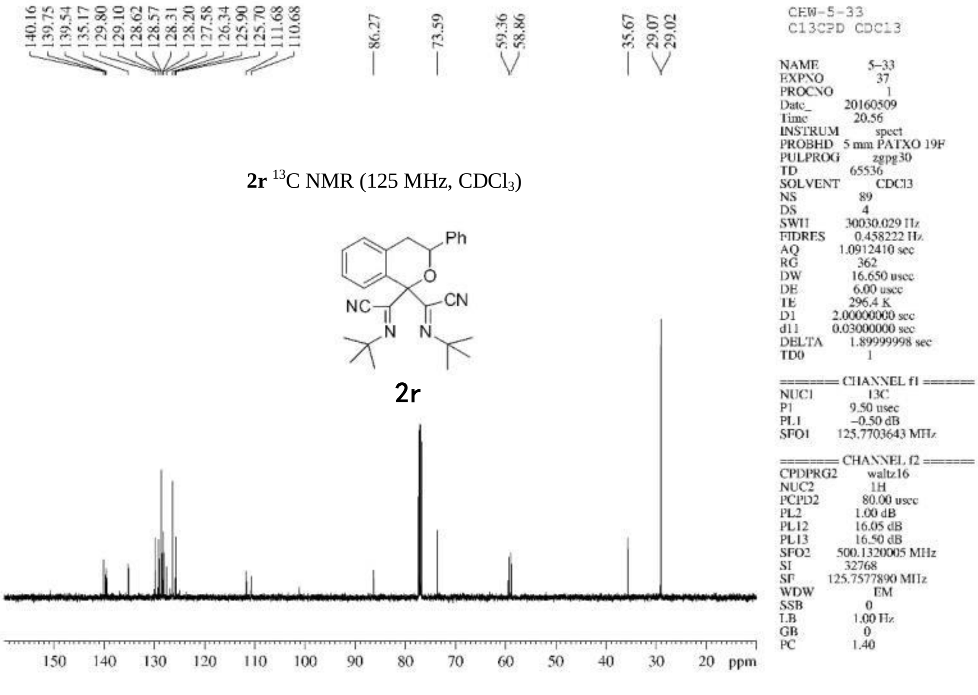

Figure S47. ^{1}H and ^{13}C NMR spectra of **2r**. Related to **Figure 2**.

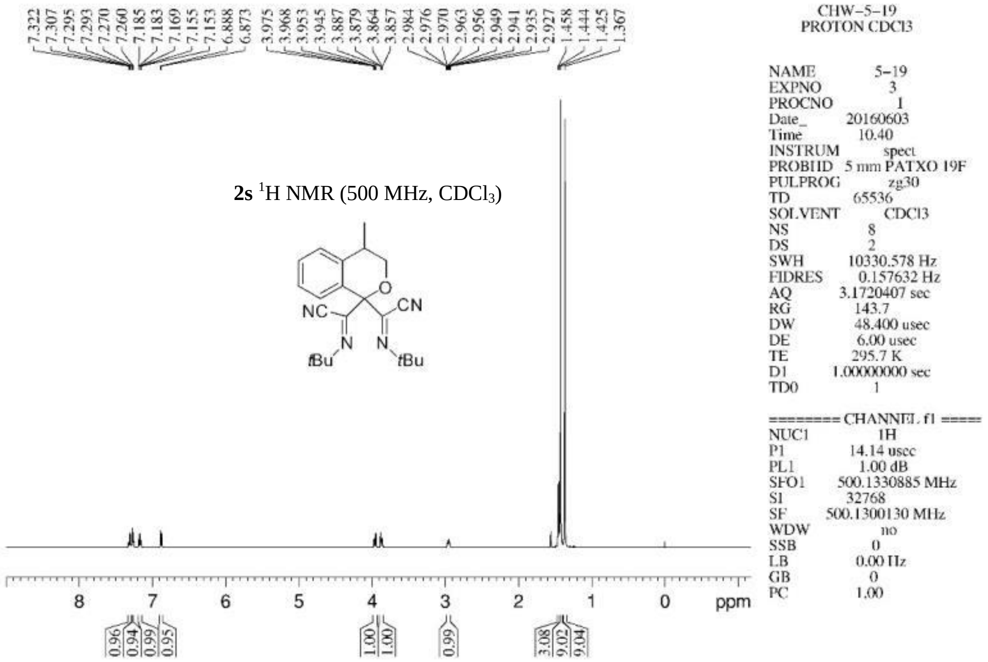

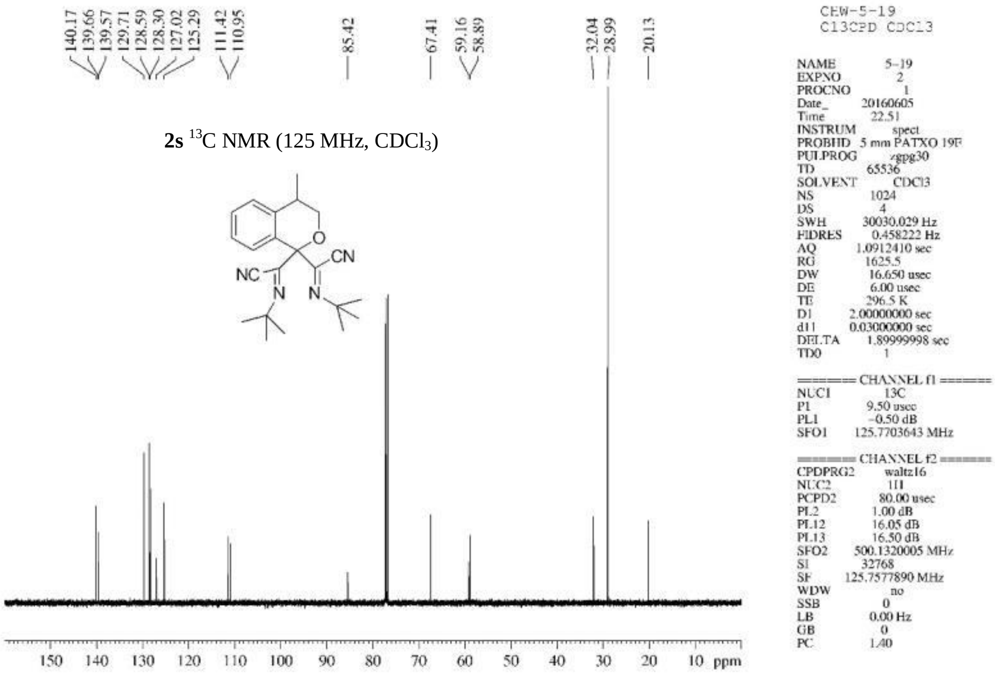

Figure S48. ^{1}H and ^{13}C NMR spectra of **2s**. Related to **Figure 2**.

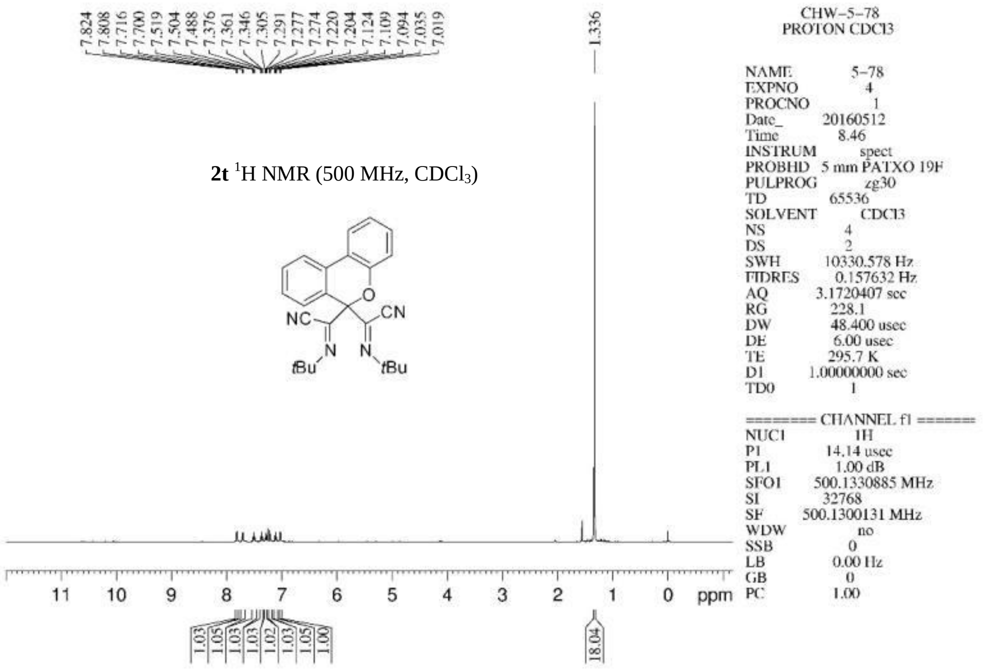

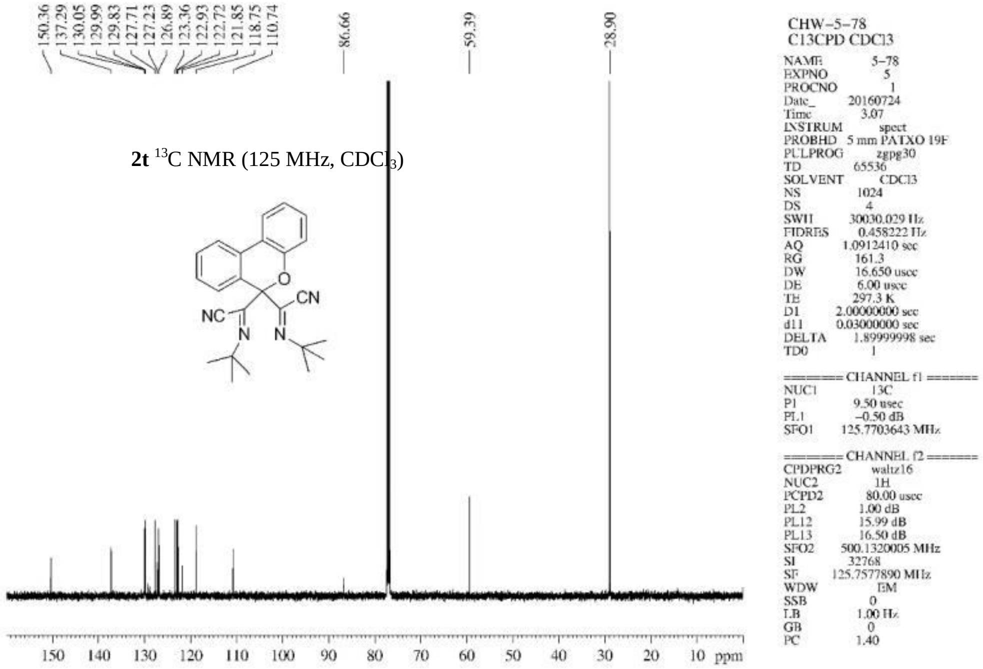

Figure S49. ^{1}H and ^{13}C NMR spectra of **2t**. Related to **Figure 2**.

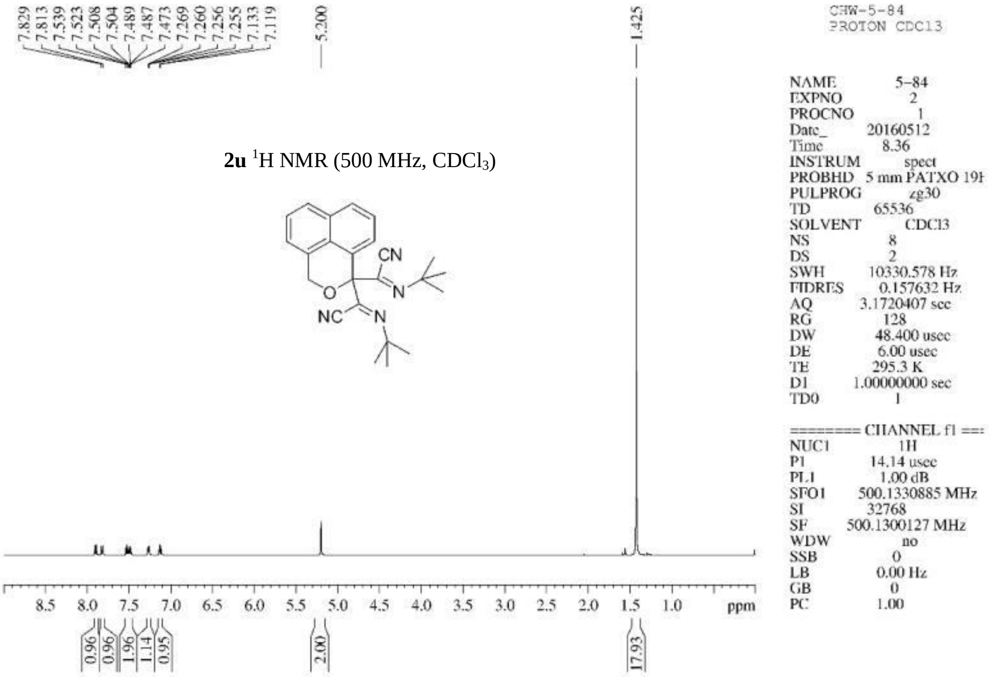

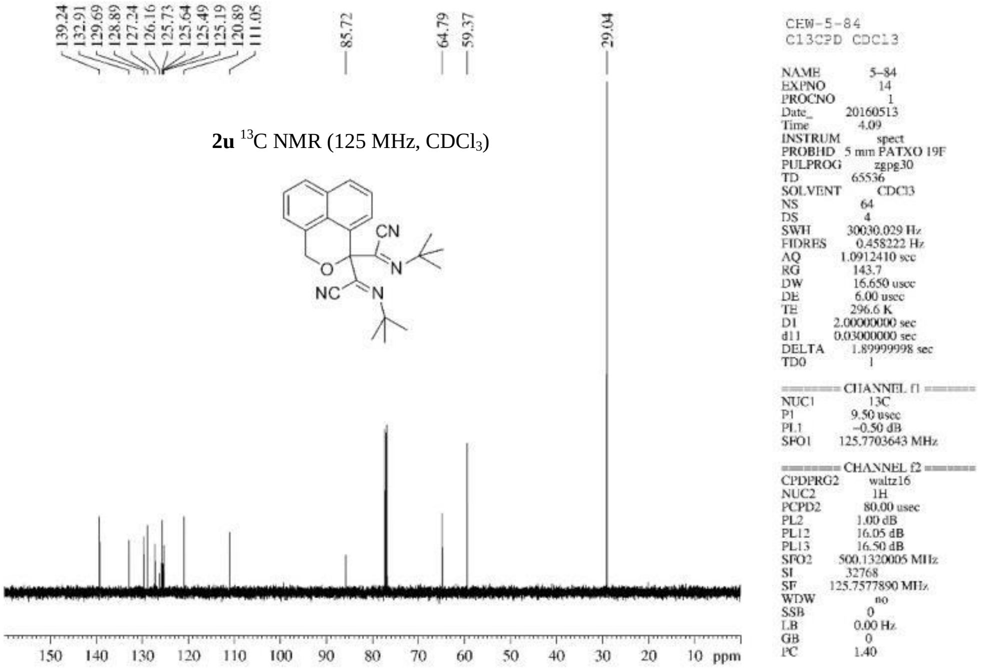

Figure S50. ^{1}H and ^{13}C NMR spectra of **2u**. Related to **Figure 2**.

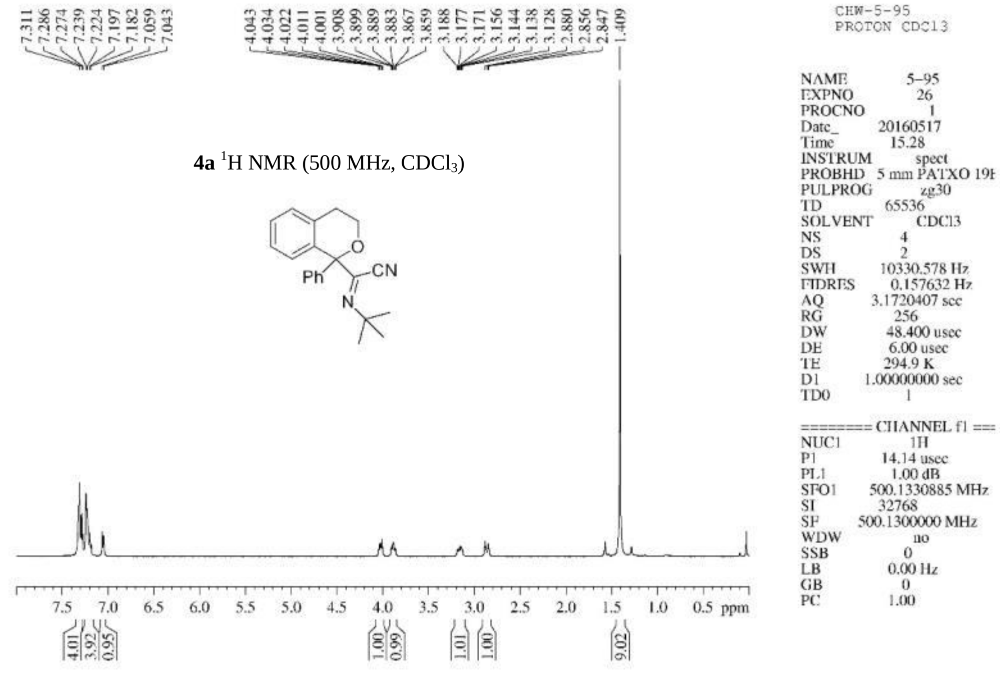

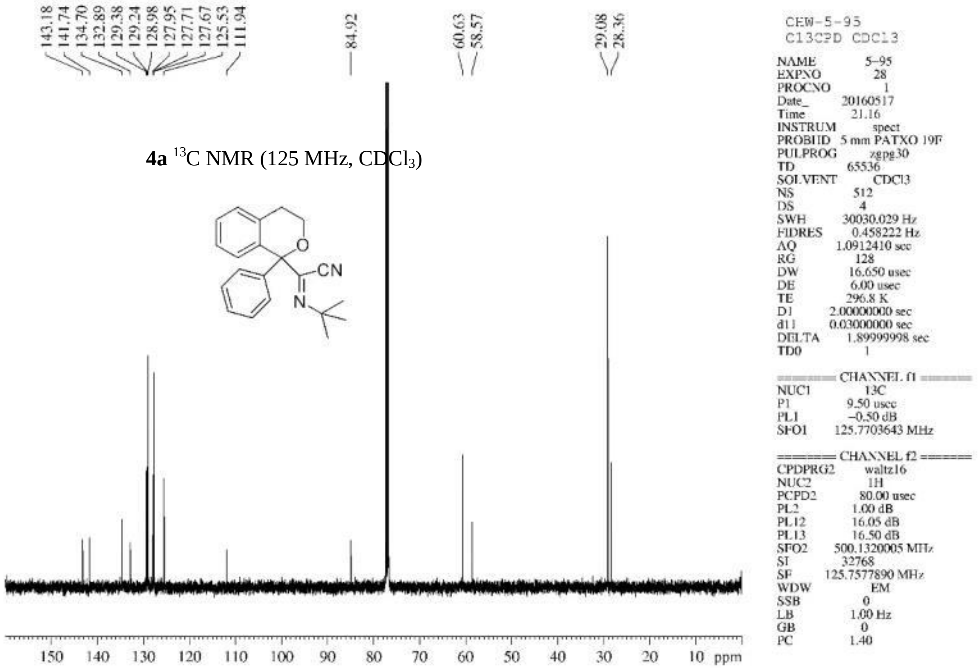

Figure S51. ^{1}H and ^{13}C NMR spectra of **4a**. Related to **Figure 3**.

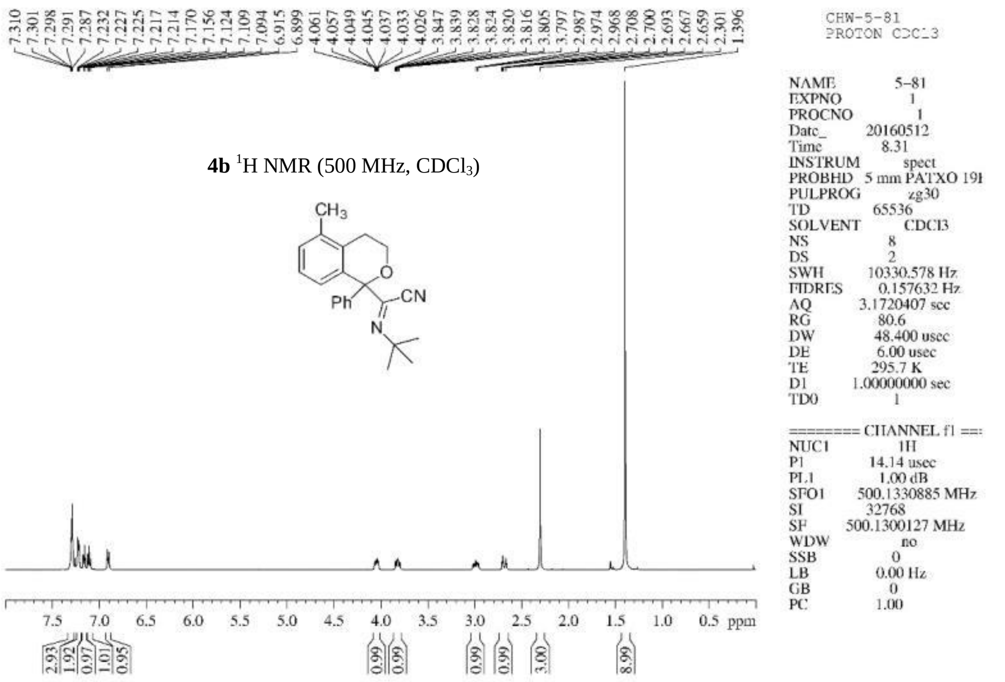

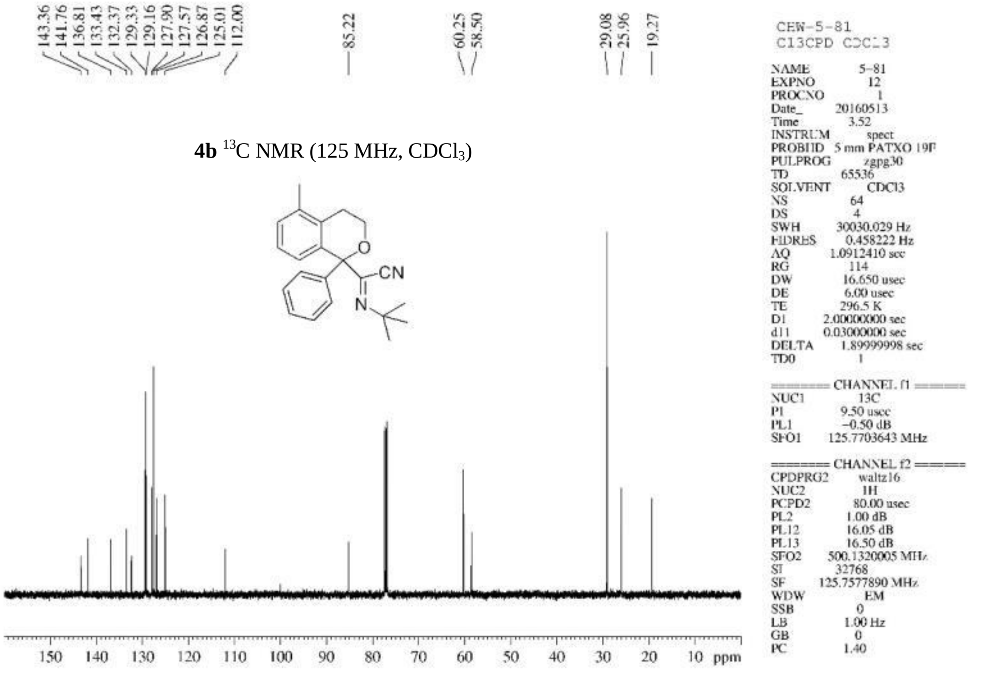

Figure S52. ^{1}H and ^{13}C NMR spectra of **4b**. Related to **Figure 3**.

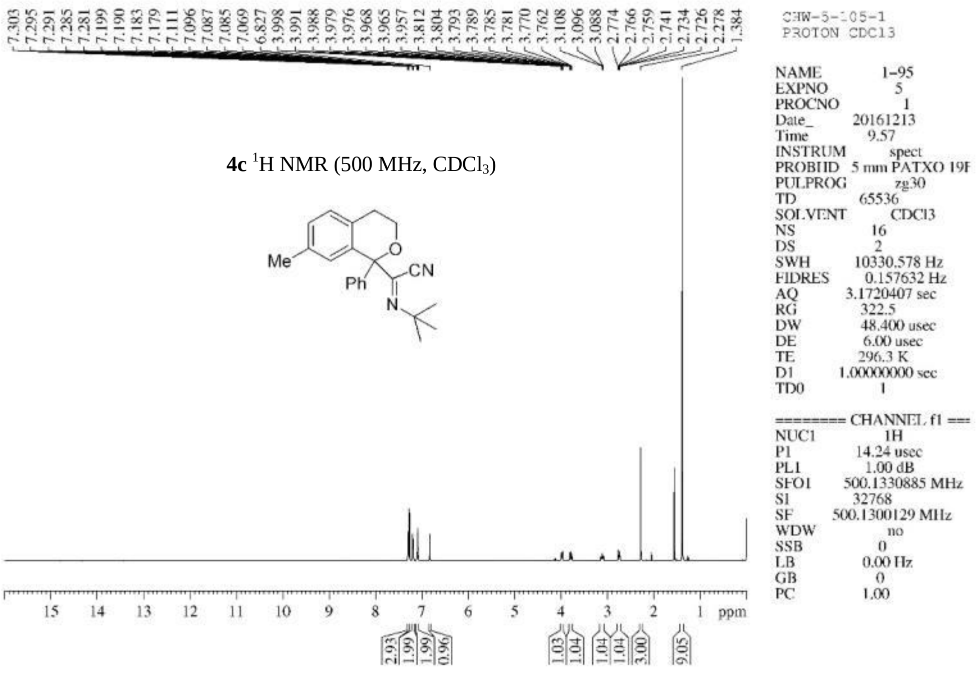

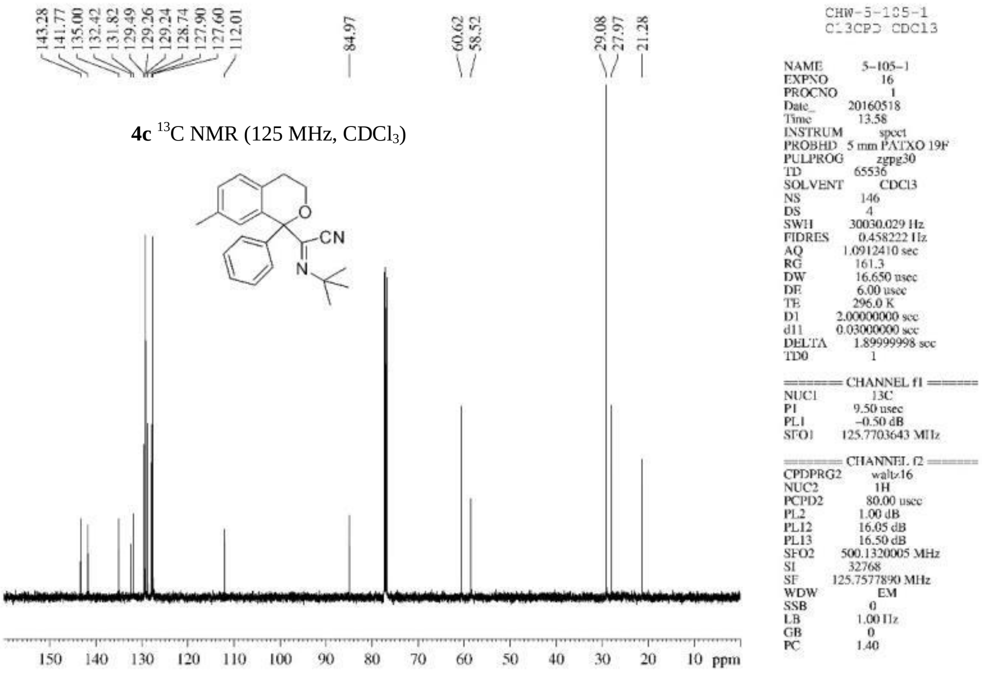

Figure S53. ^{1}H and ^{13}C NMR spectra of **4c**. Related to **Figure 3**.

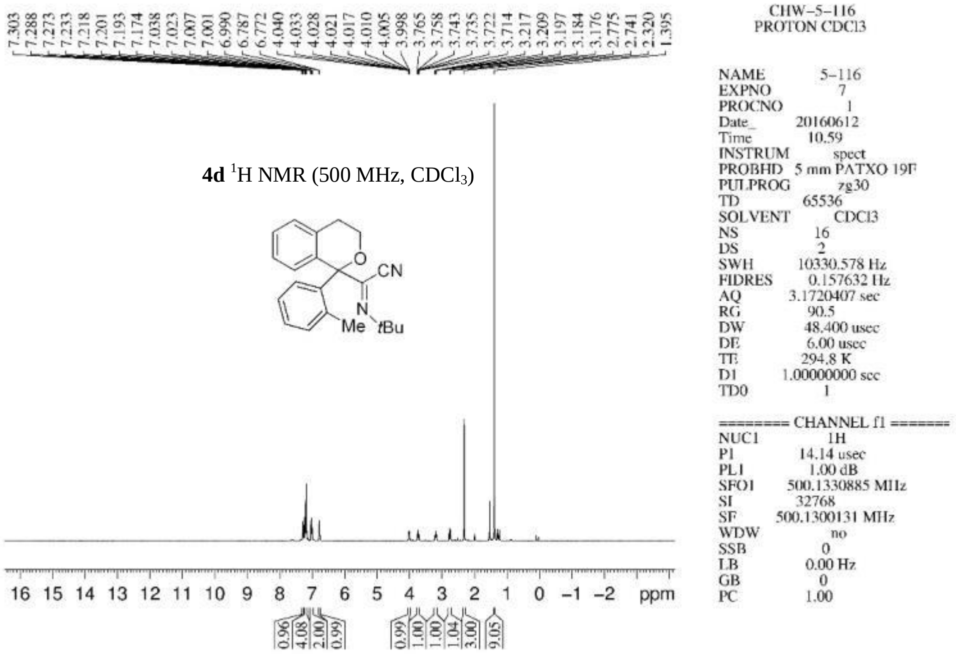

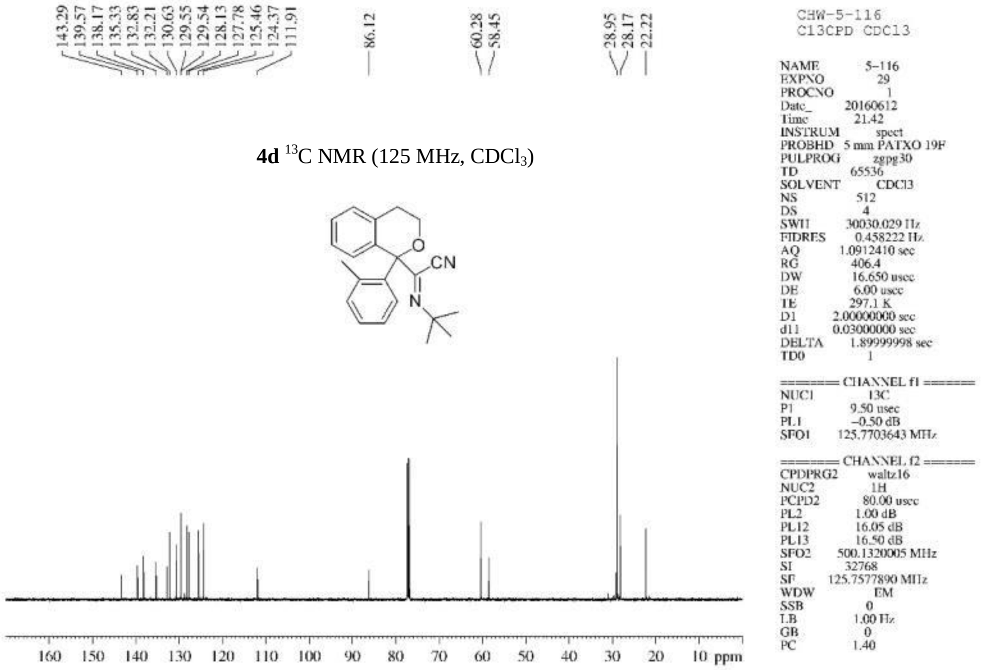

Figure S54. ^{1}H and ^{13}C NMR spectra of **4d**. Related to **Figure 3**.

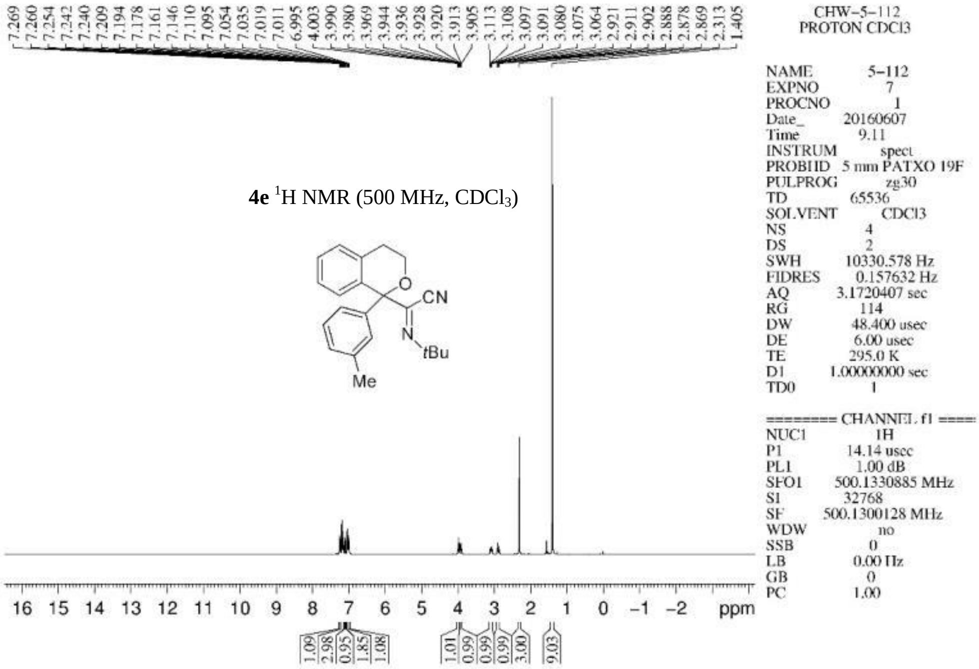

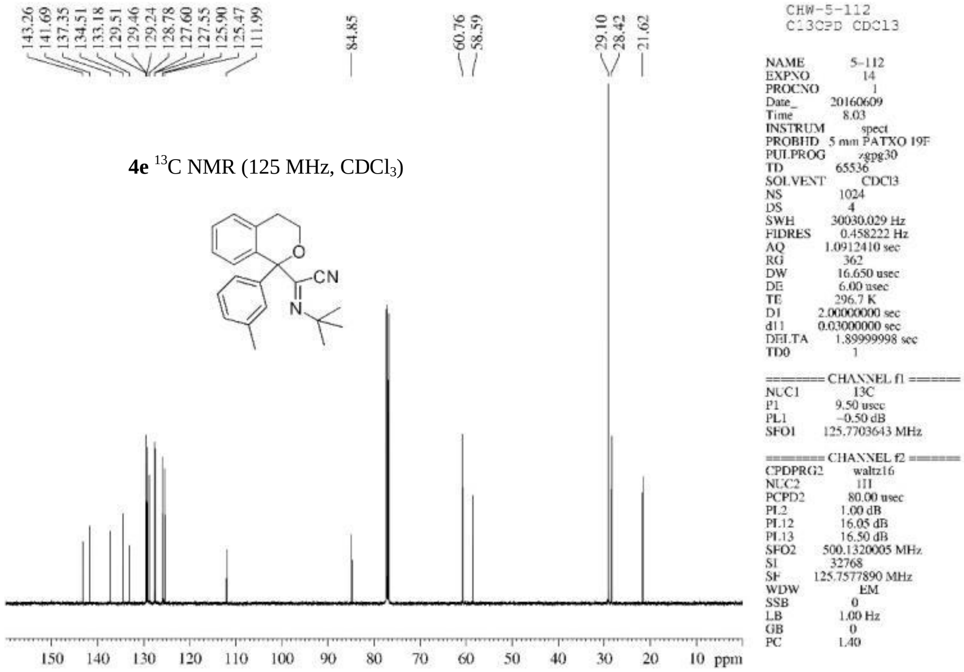

Figure S55. ^{1}H and ^{13}C NMR spectra of **4e**. Related to **Figure 3**.

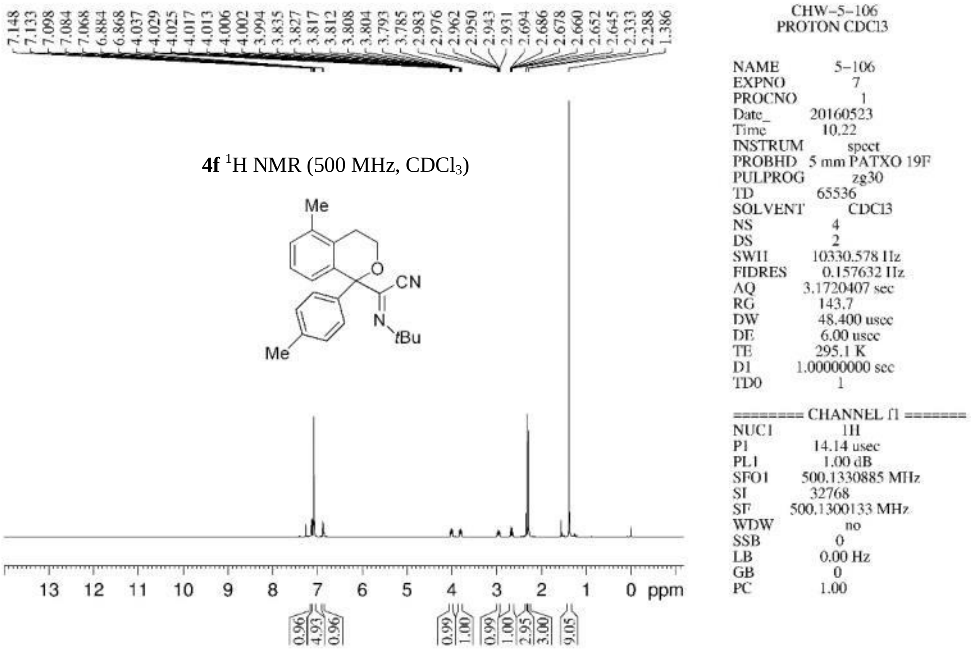

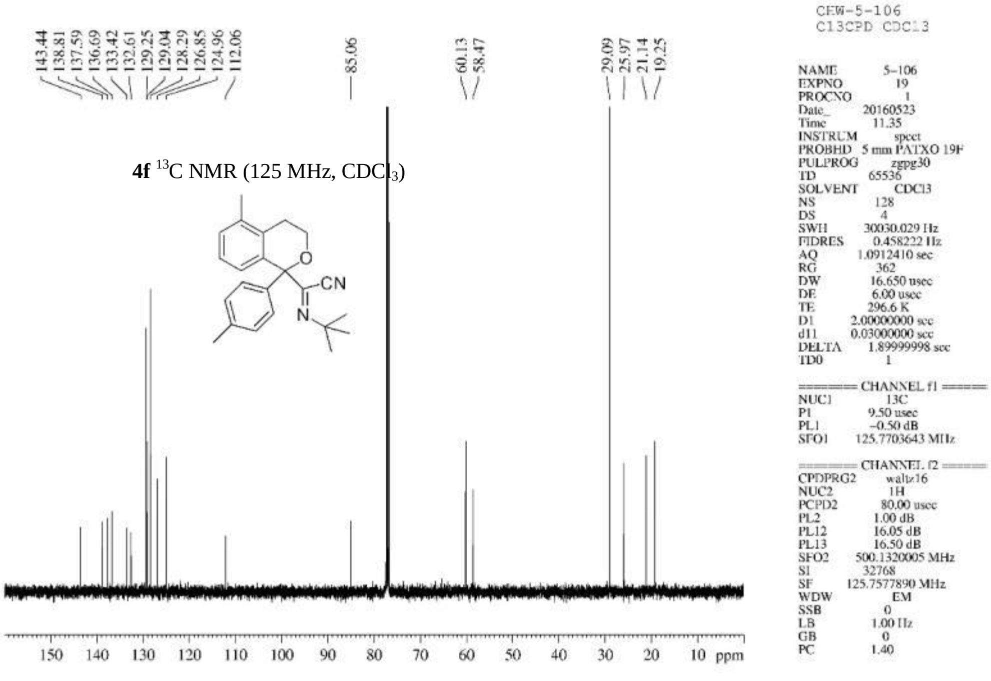

Figure S56. ^{1}H and ^{13}C NMR spectra of **4f**. Related to **Figure 3**.

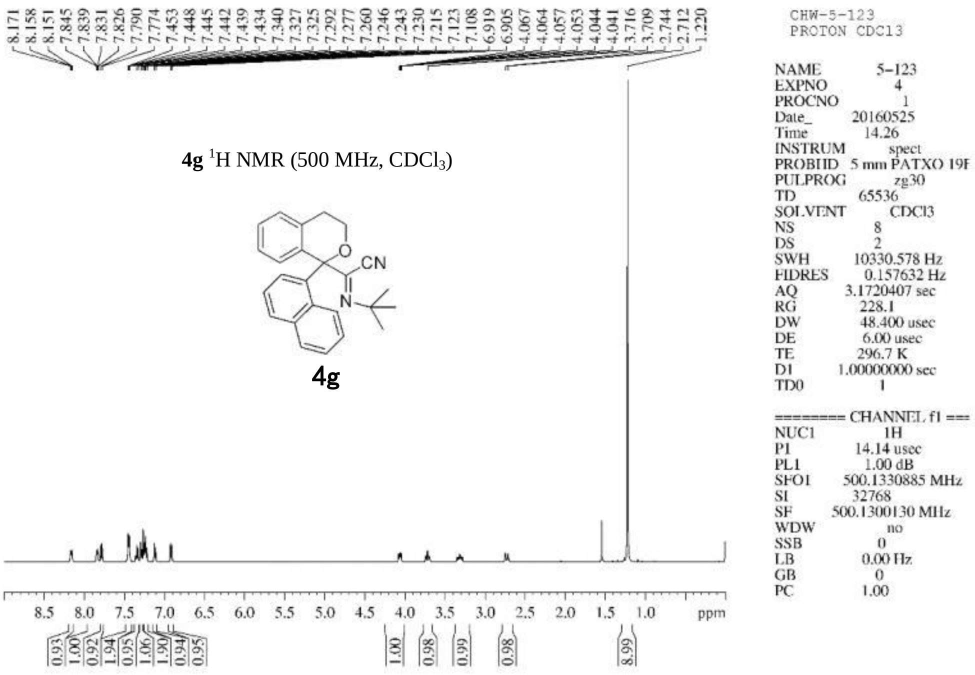

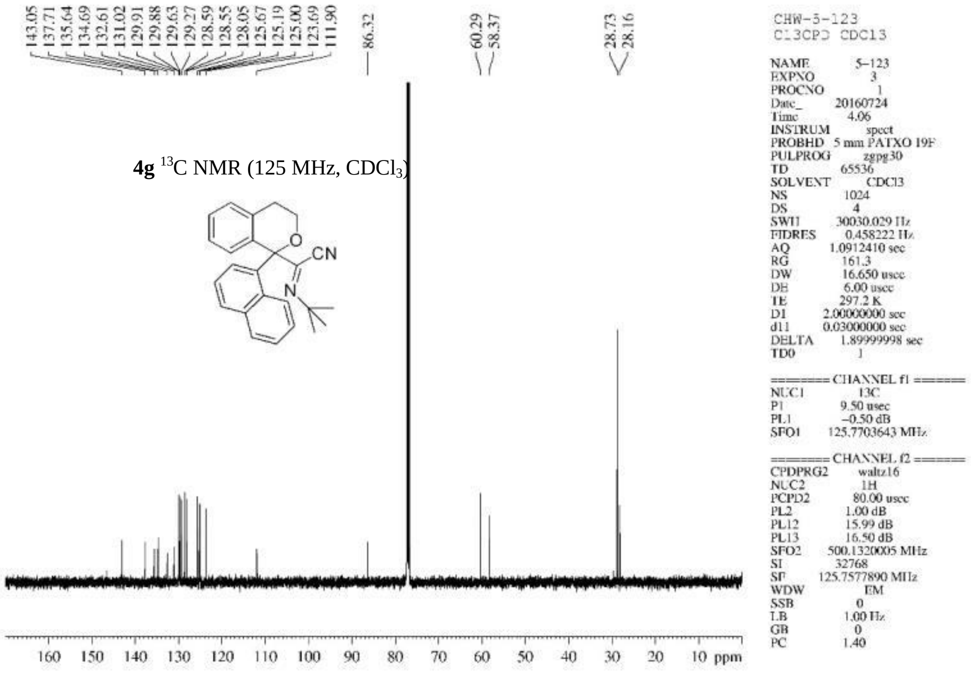

Figure S57. ^{1}H and ^{13}C NMR spectra of **4g**. Related to **Figure 3**.

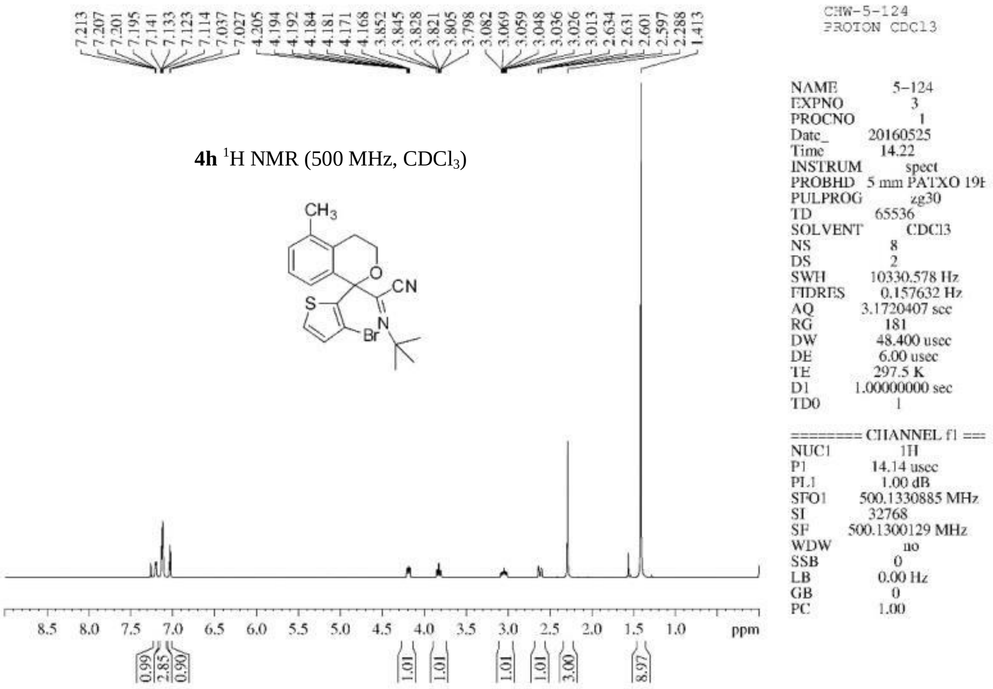

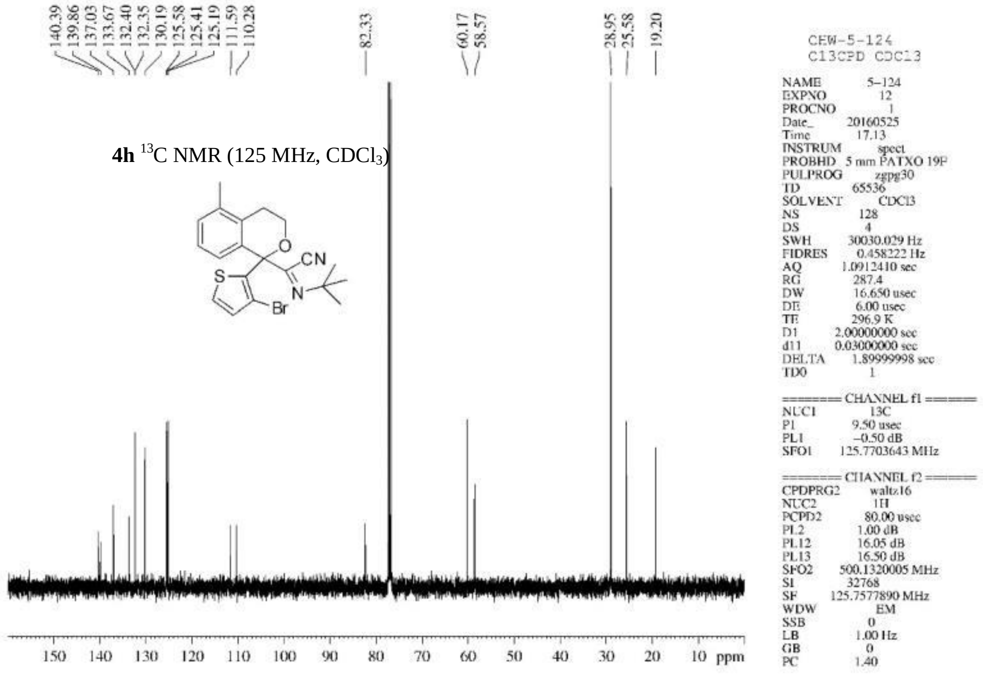

Figure S58. ^{1}H and ^{13}C NMR spectra of **4h**. Related to **Figure 3**.

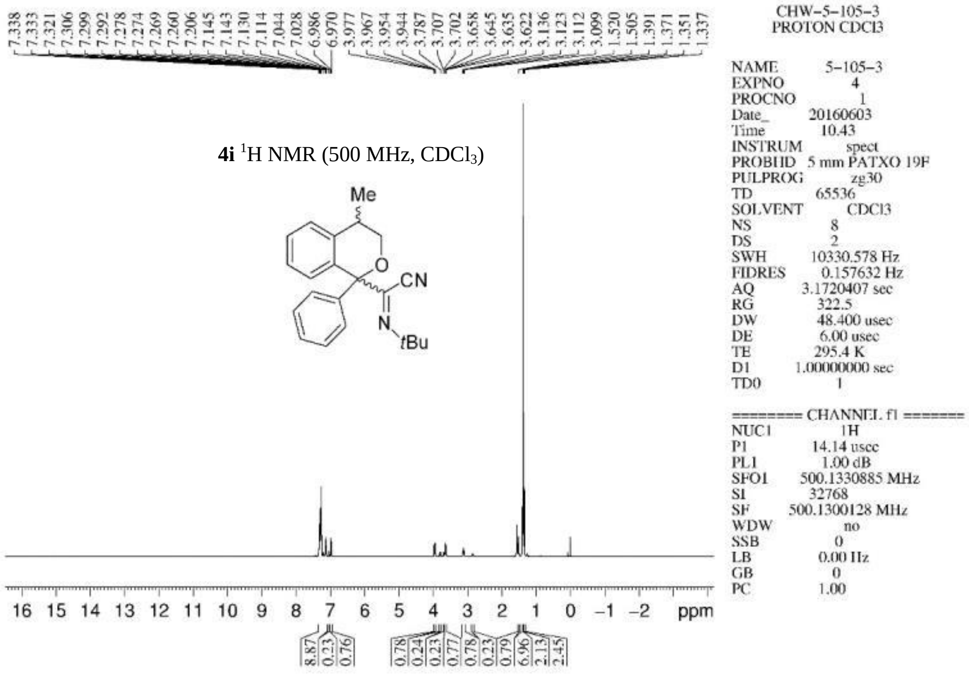

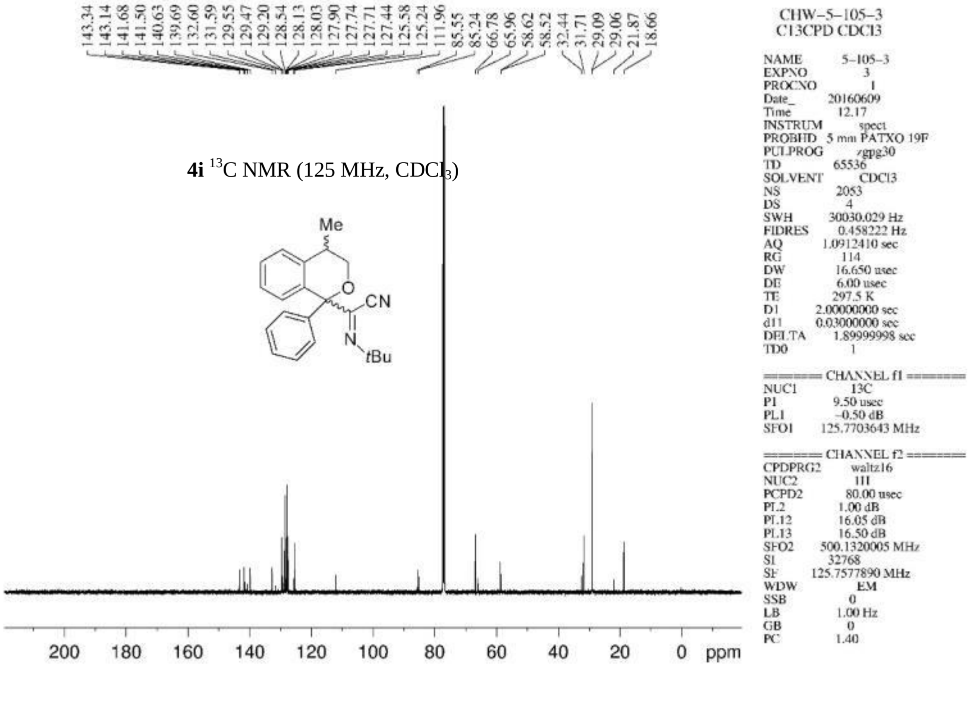

Figure S59. ^{1}H and ^{13}C NMR spectra of **4i**. Related to **Figure 3**.

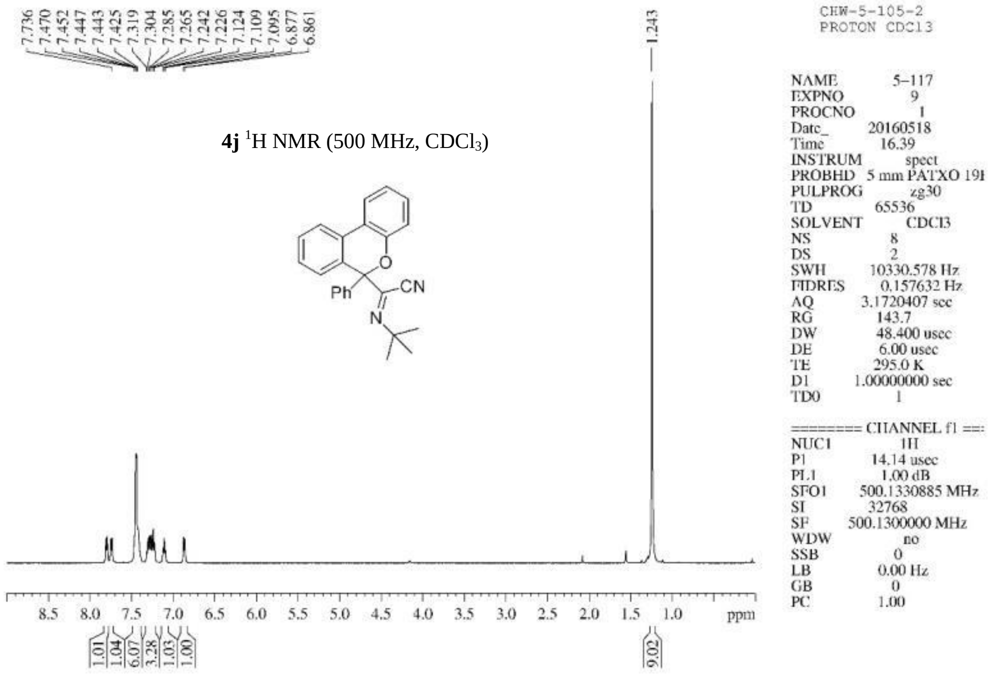

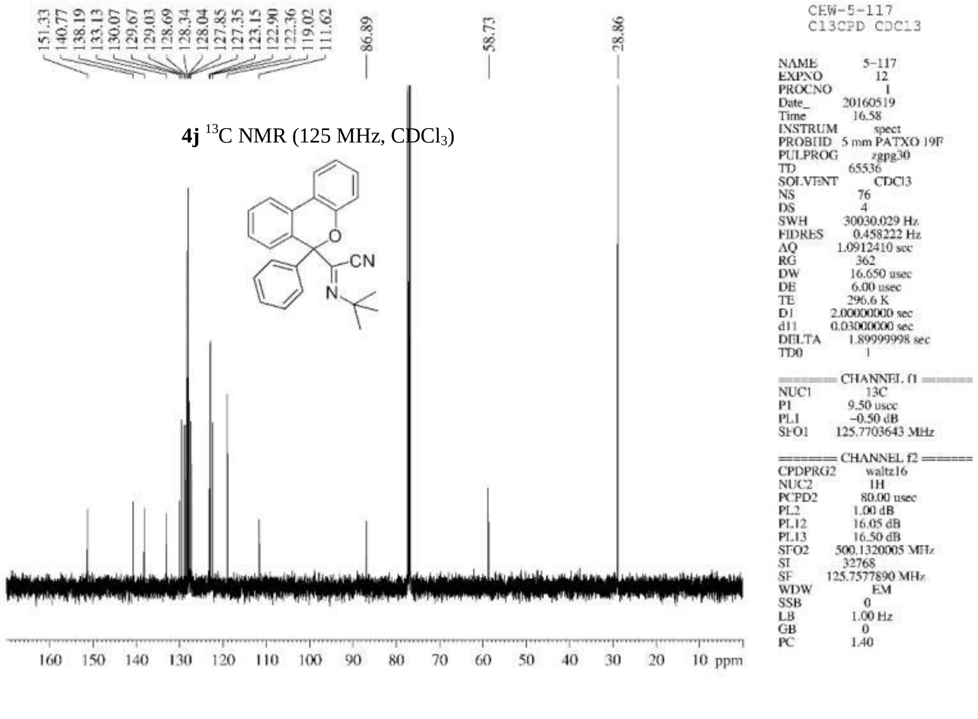

Figure S60. ^{1}H and ^{13}C NMR spectra of **4j**. Related to **Figure 3**.

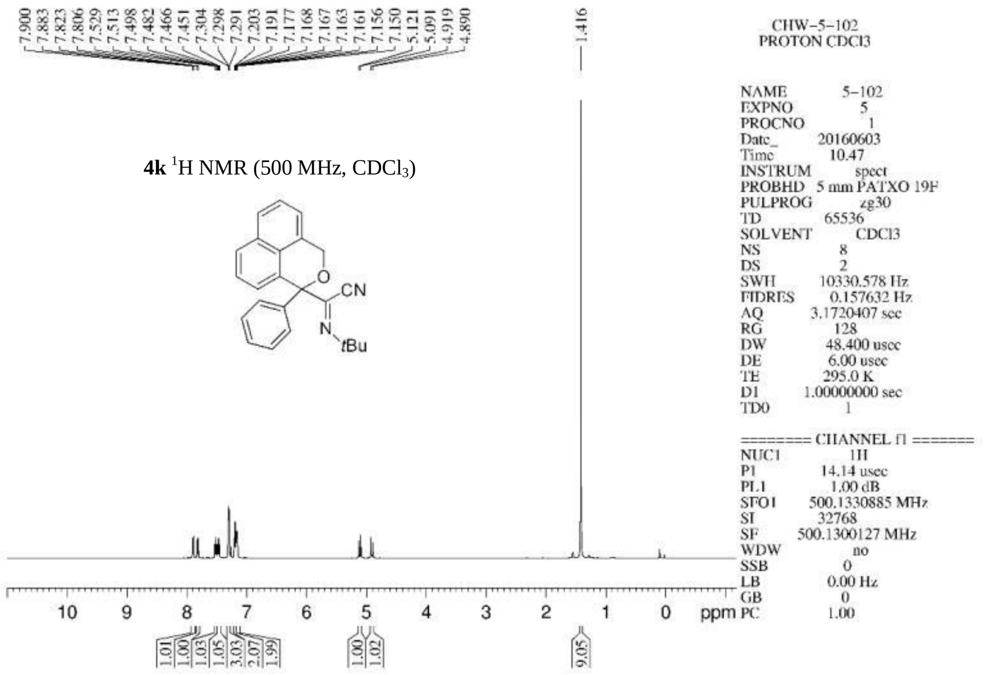

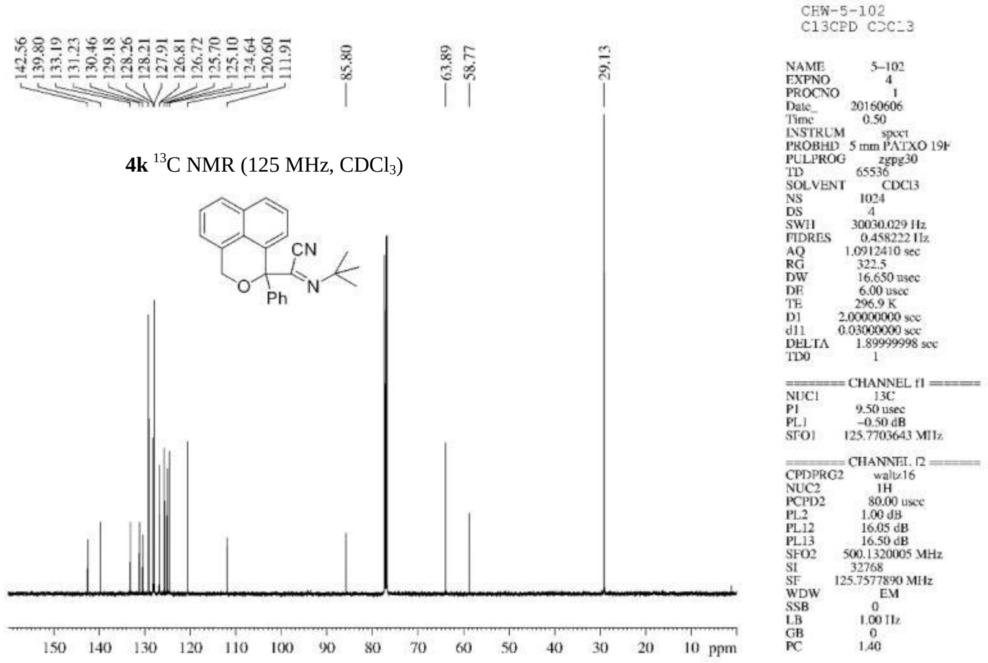

Figure S61. ^{1}H and ^{13}C NMR spectra of **4k**. Related to **Figure 3**.

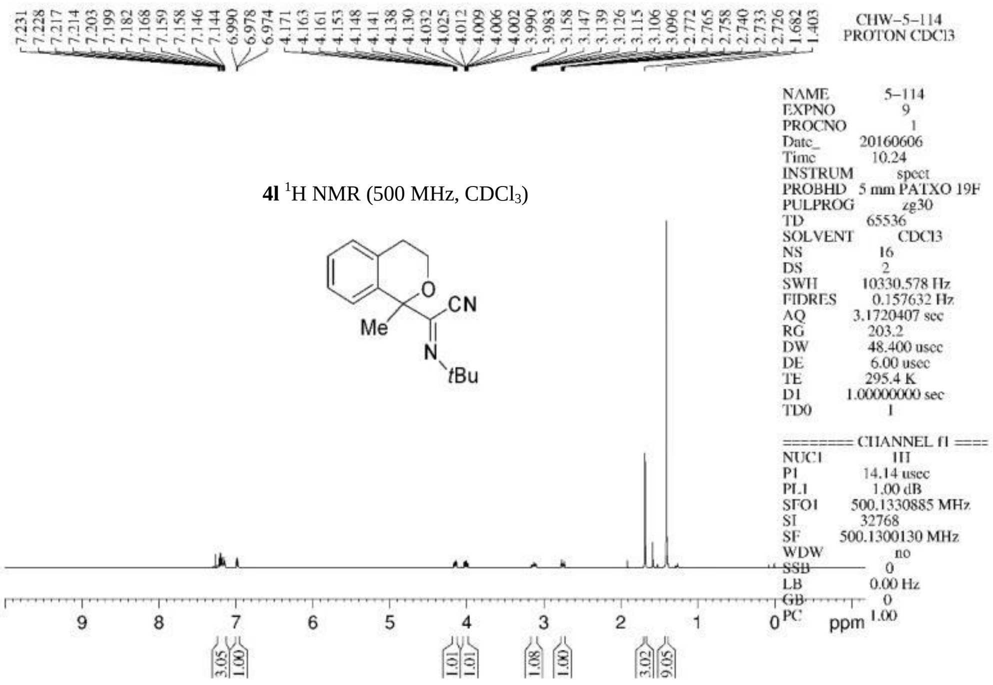

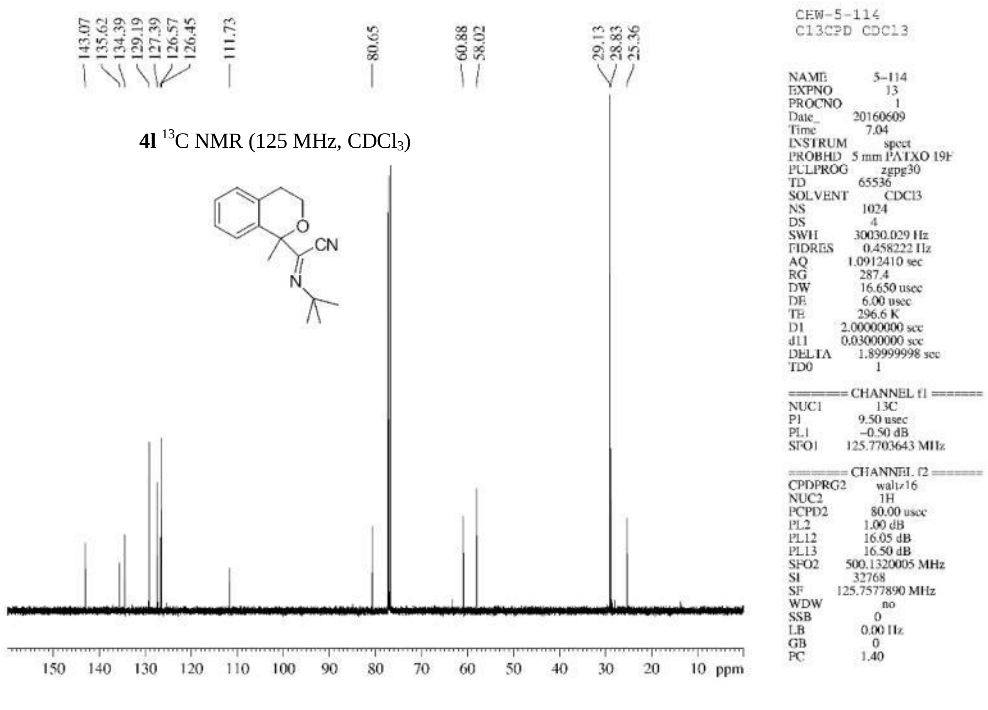

Figure S62. ^{1}H and ^{13}C NMR spectra of **4l**. Related to **Figure 3**.

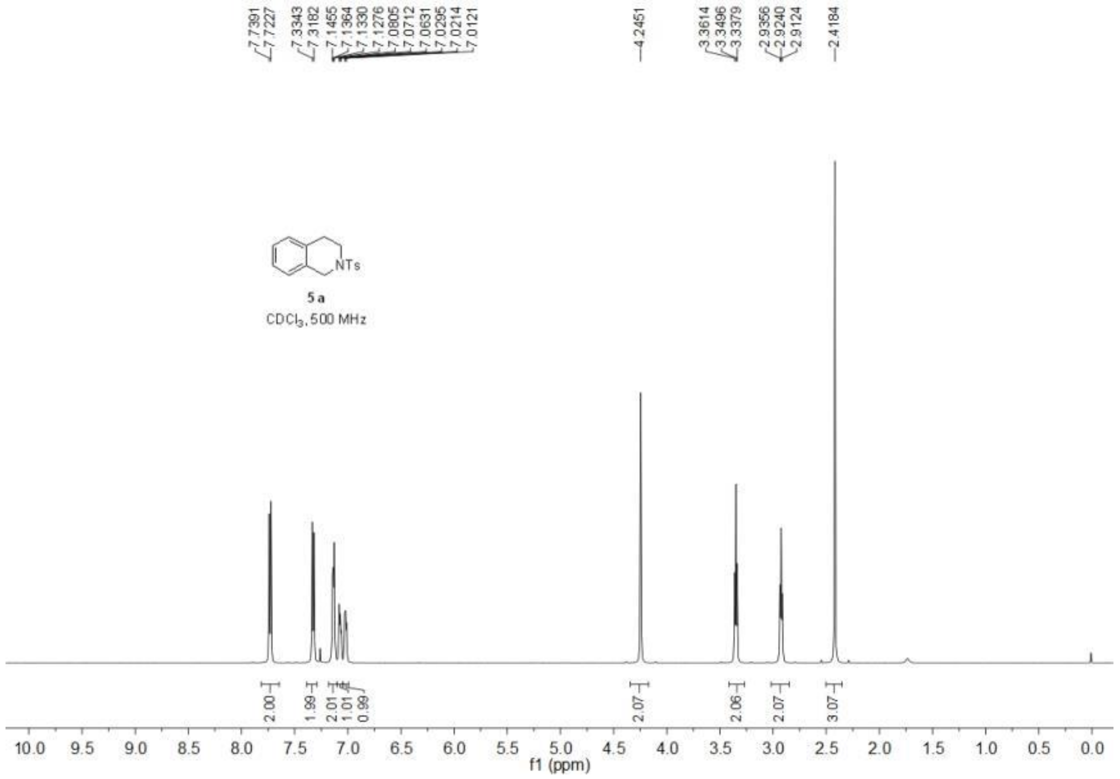

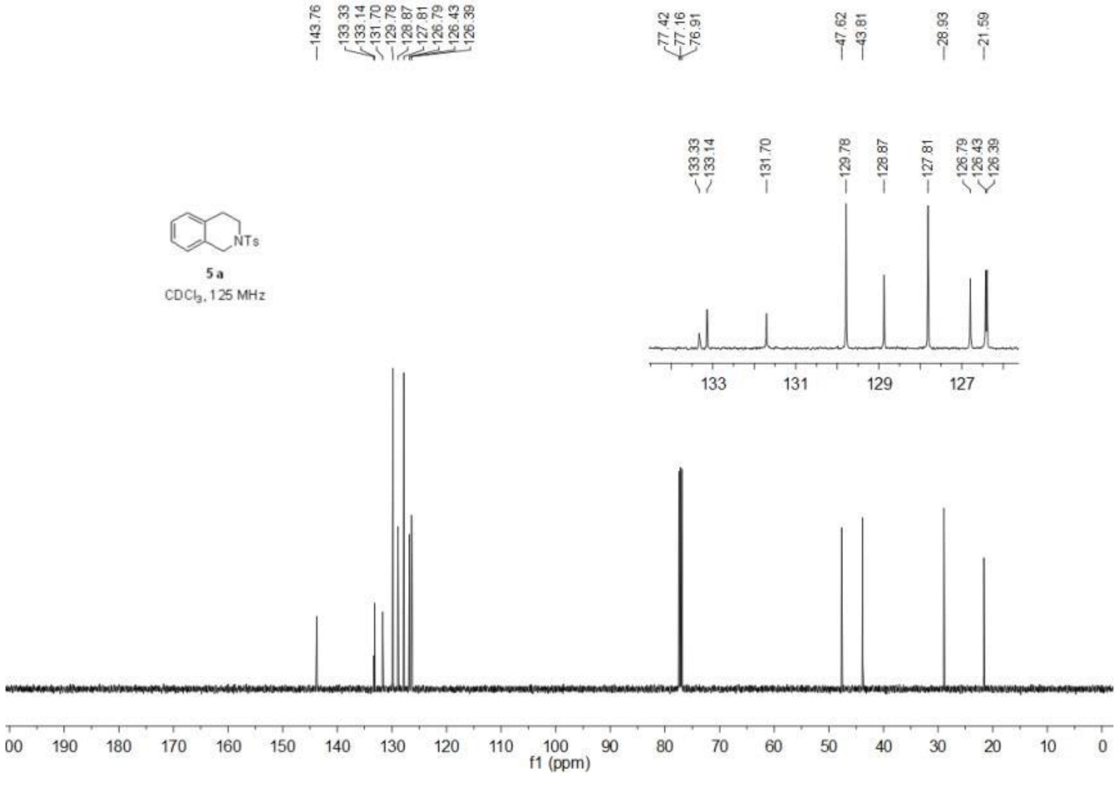

Figure S63. ^{1}H and ^{13}C NMR spectra of **5a**. Related to **Figure 4**.

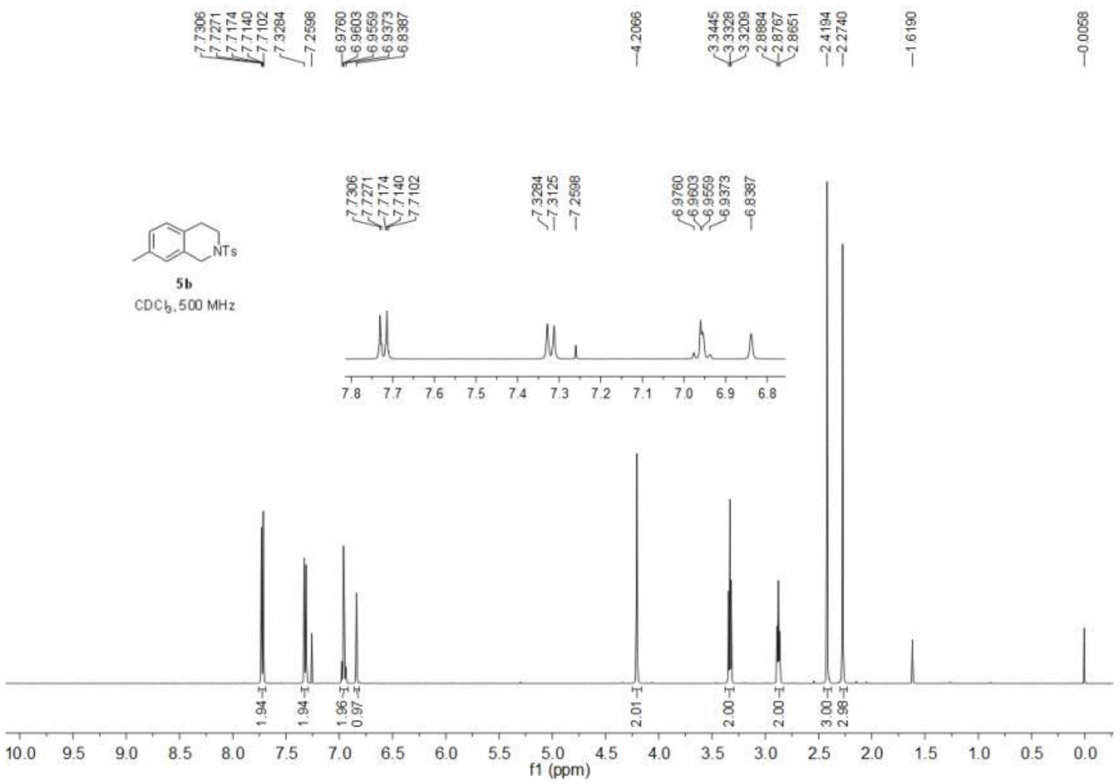

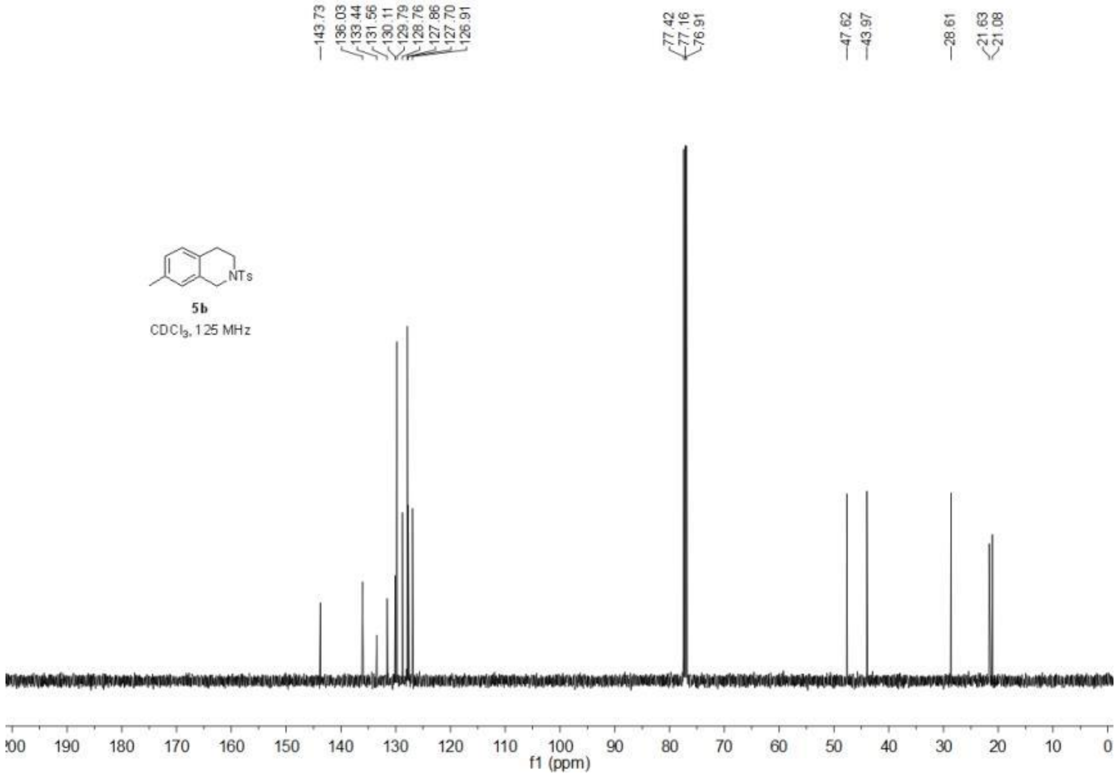

Figure S64. ^{1}H and ^{13}C NMR spectra of **5b**. Related to **Figure 4**.

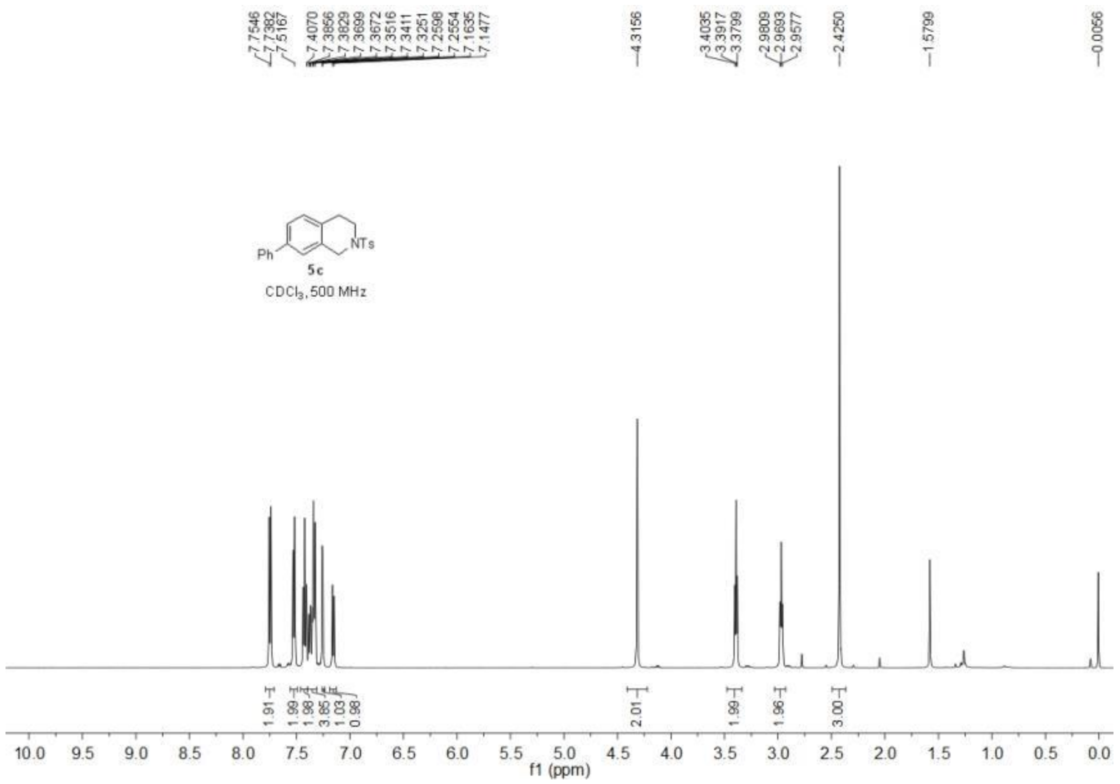

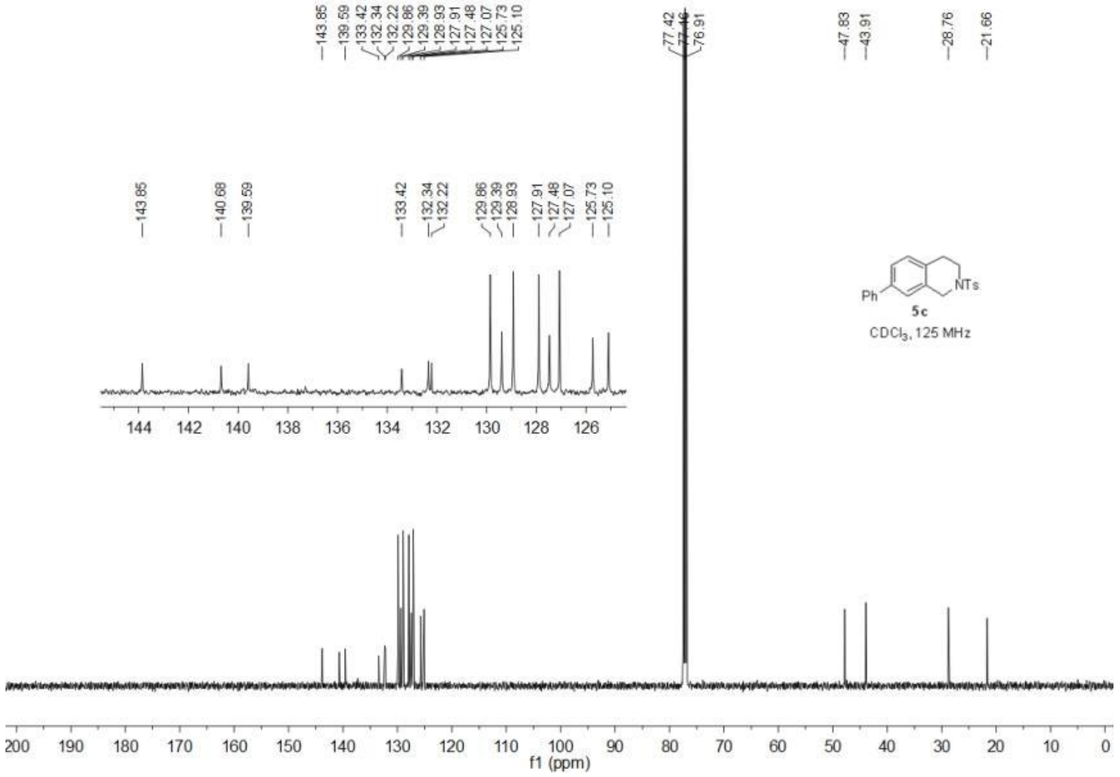

Figure S65. ^{1}H and ^{13}C NMR spectra of **5c**. Related to **Figure 4**.

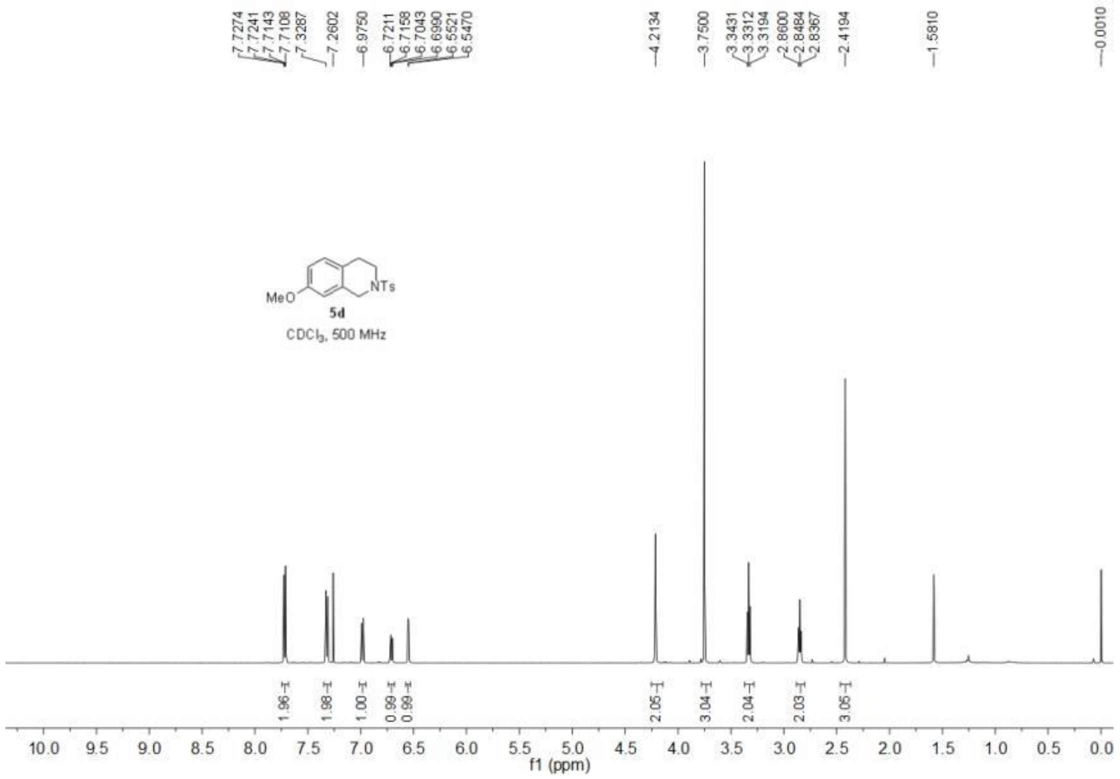

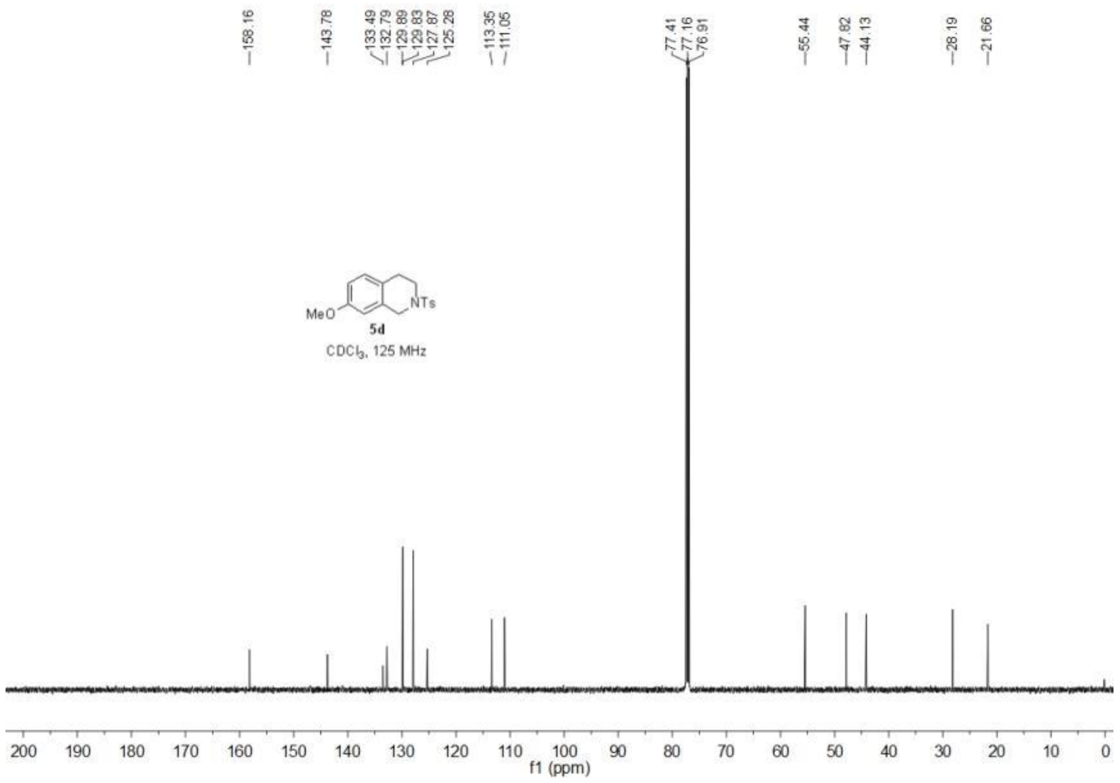

Figure S66. ^{1}H and ^{13}C NMR spectra of **5d.** Related to **Figure 4**.

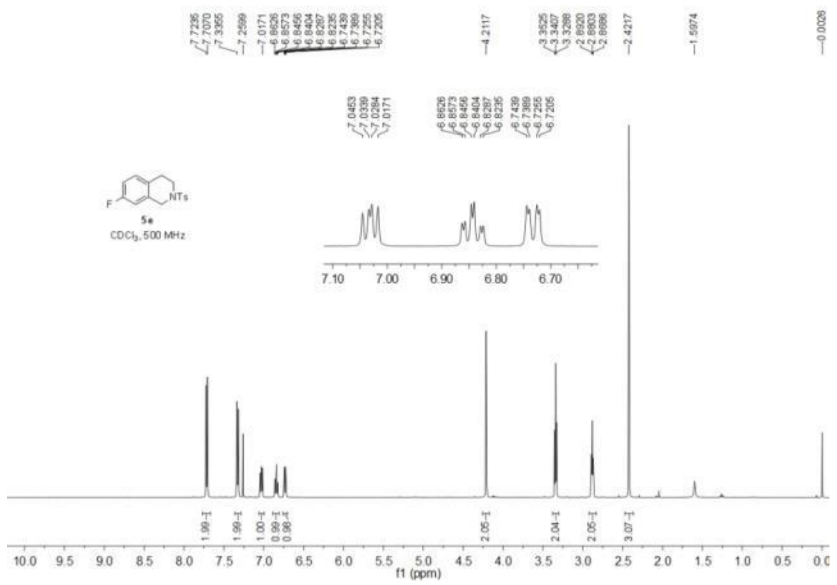

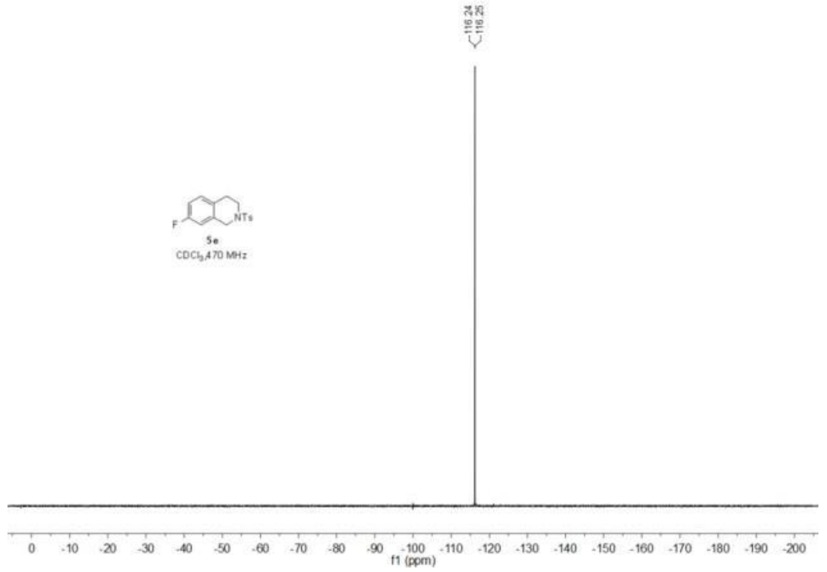

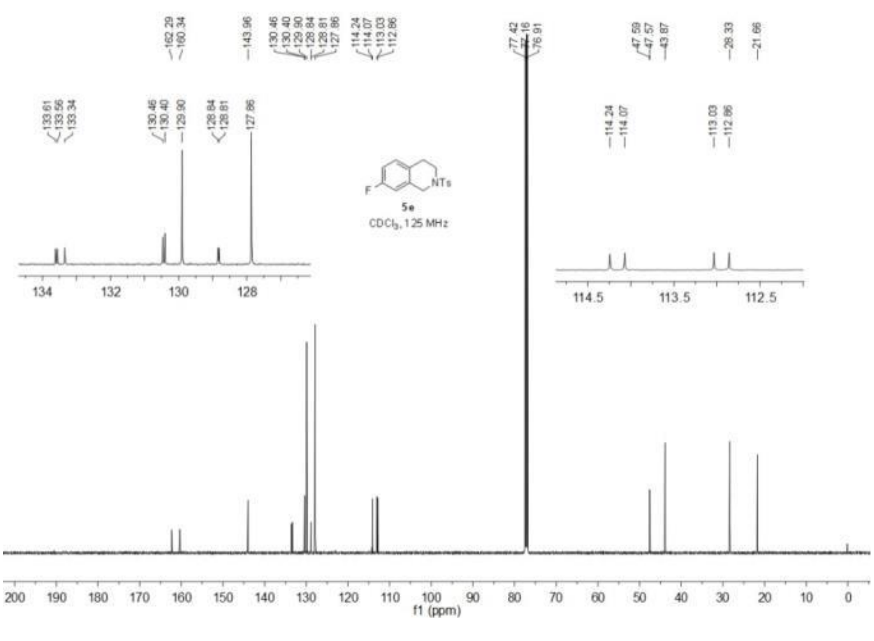

Figure S67. ^{1}H, ^{19}F and ^{13}C NMR spectra of **5e**. Related to **Figure 4**.

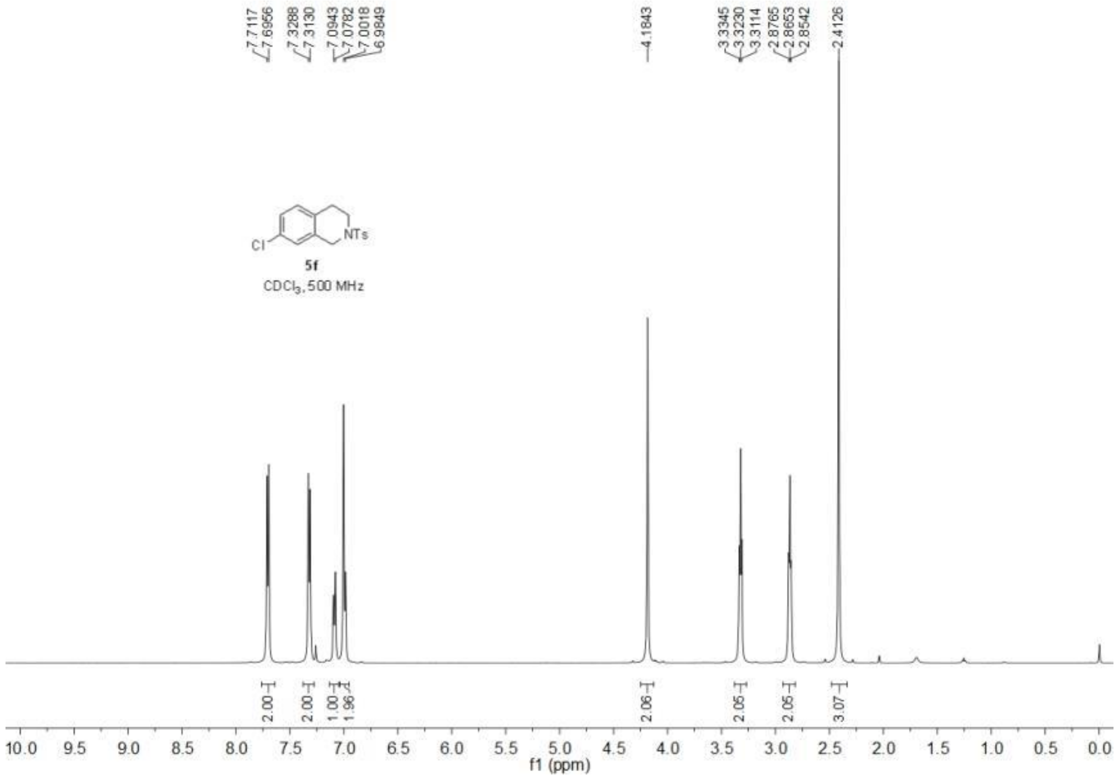

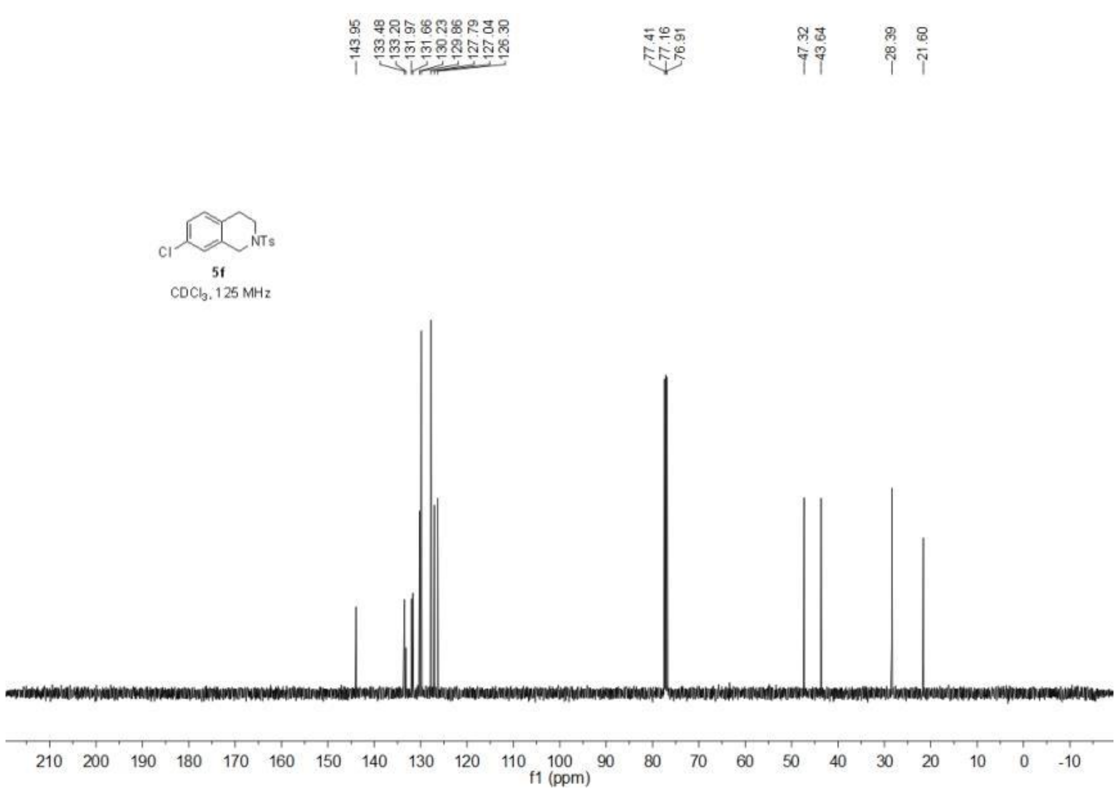

Figure S68. ^{1}H and ^{13}C NMR spectra of **5f**. Related to **Figure 4**.

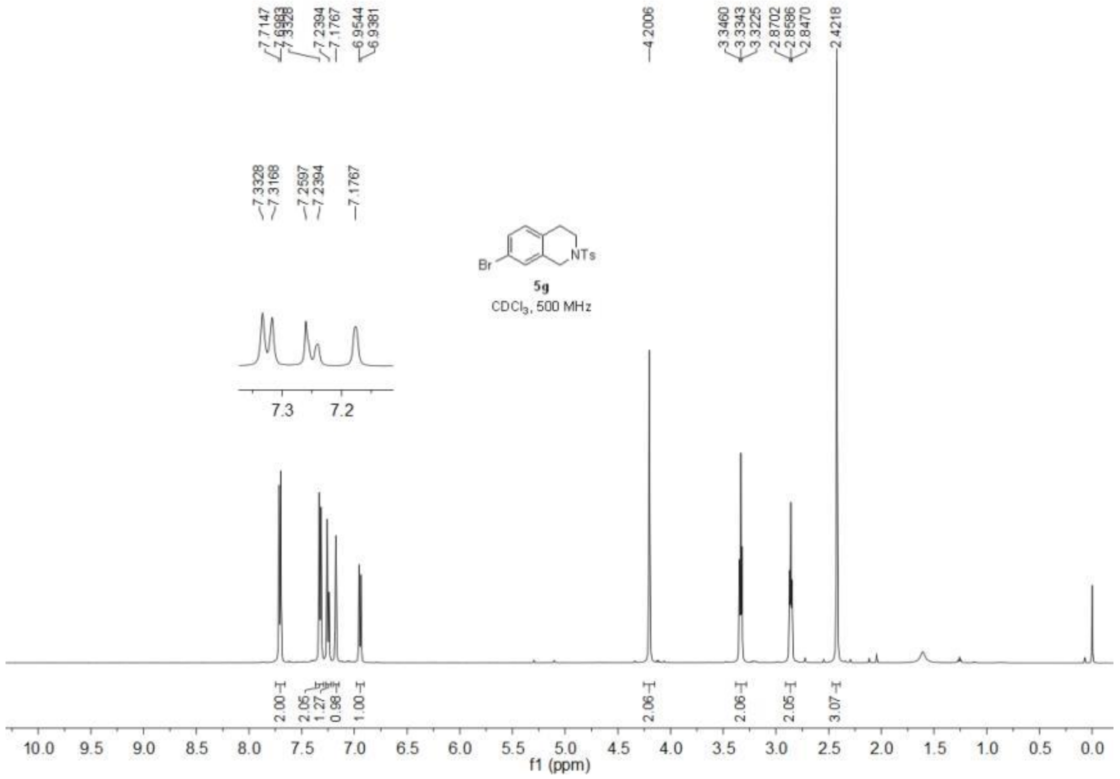

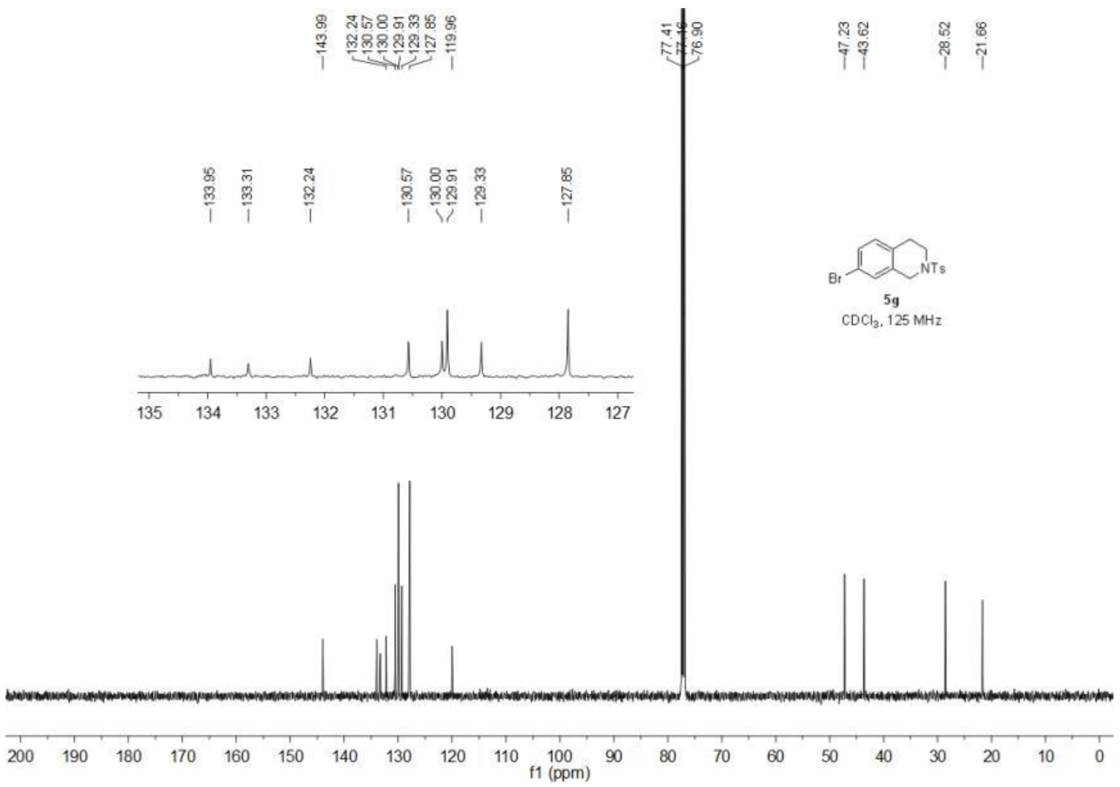

Figure S69. ^{1}H and ^{13}C NMR spectra of **5g**. Related to **Figure 4**.

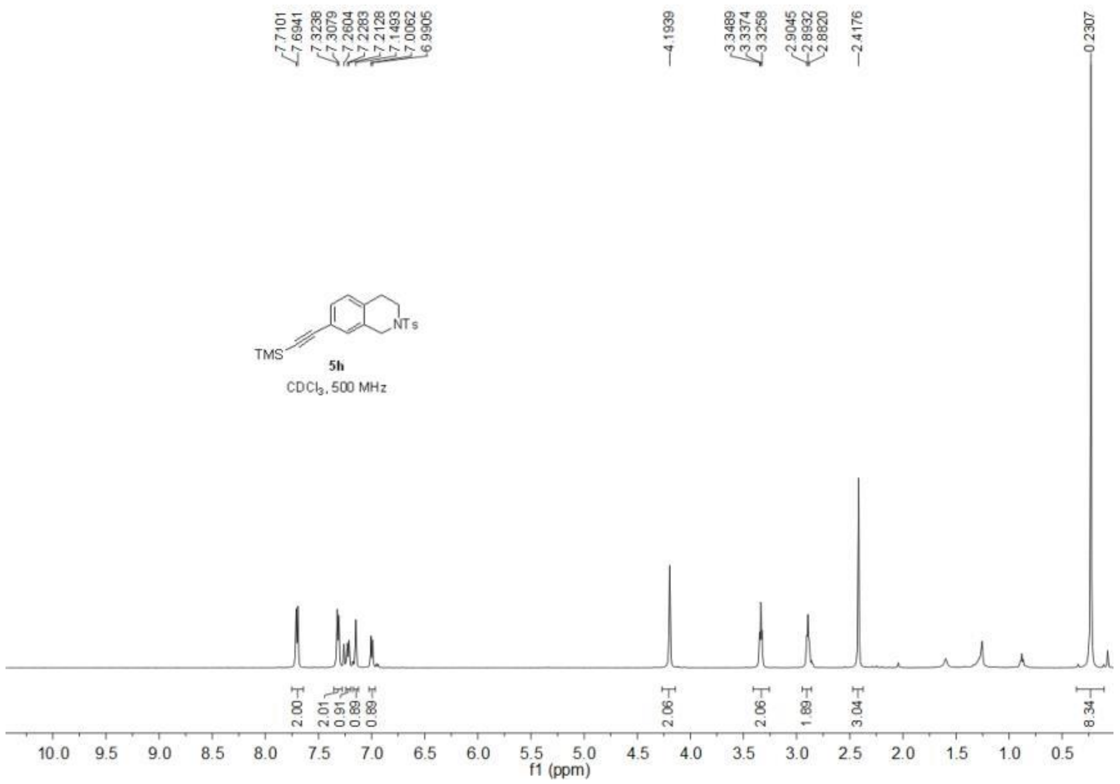

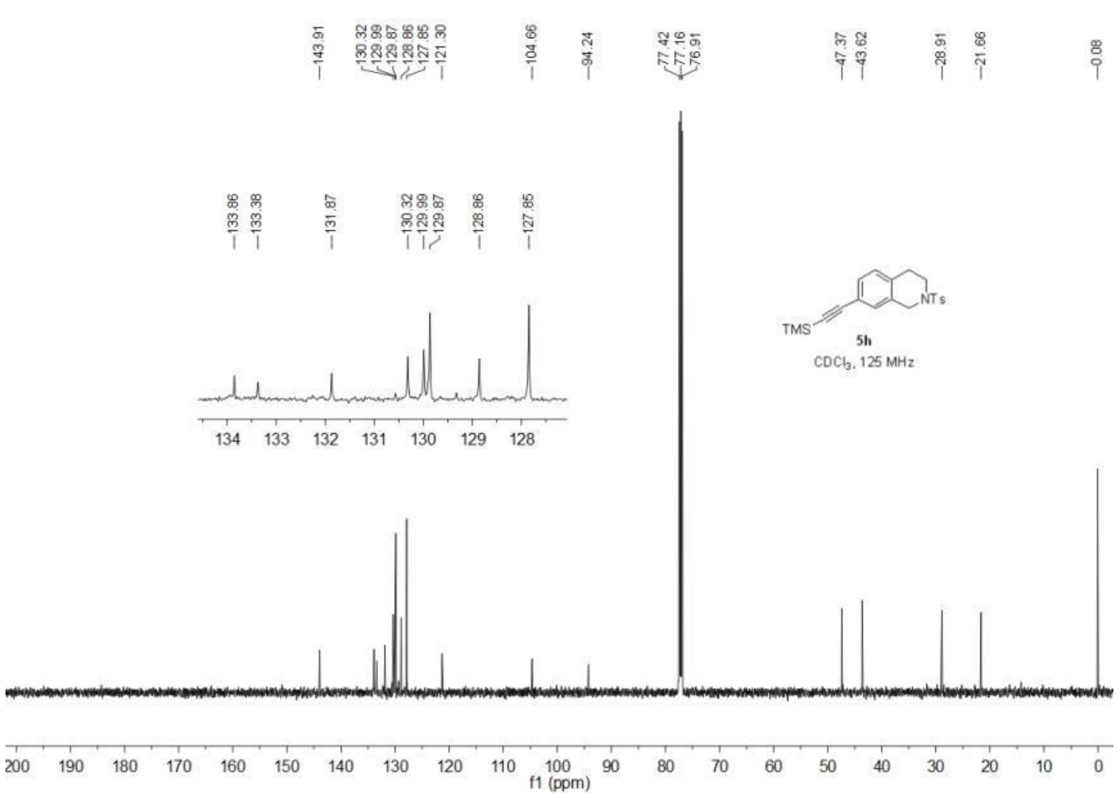

Figure S70. ^{1}H and ^{13}C NMR spectra of **5h**. Related to **Figure 4**.

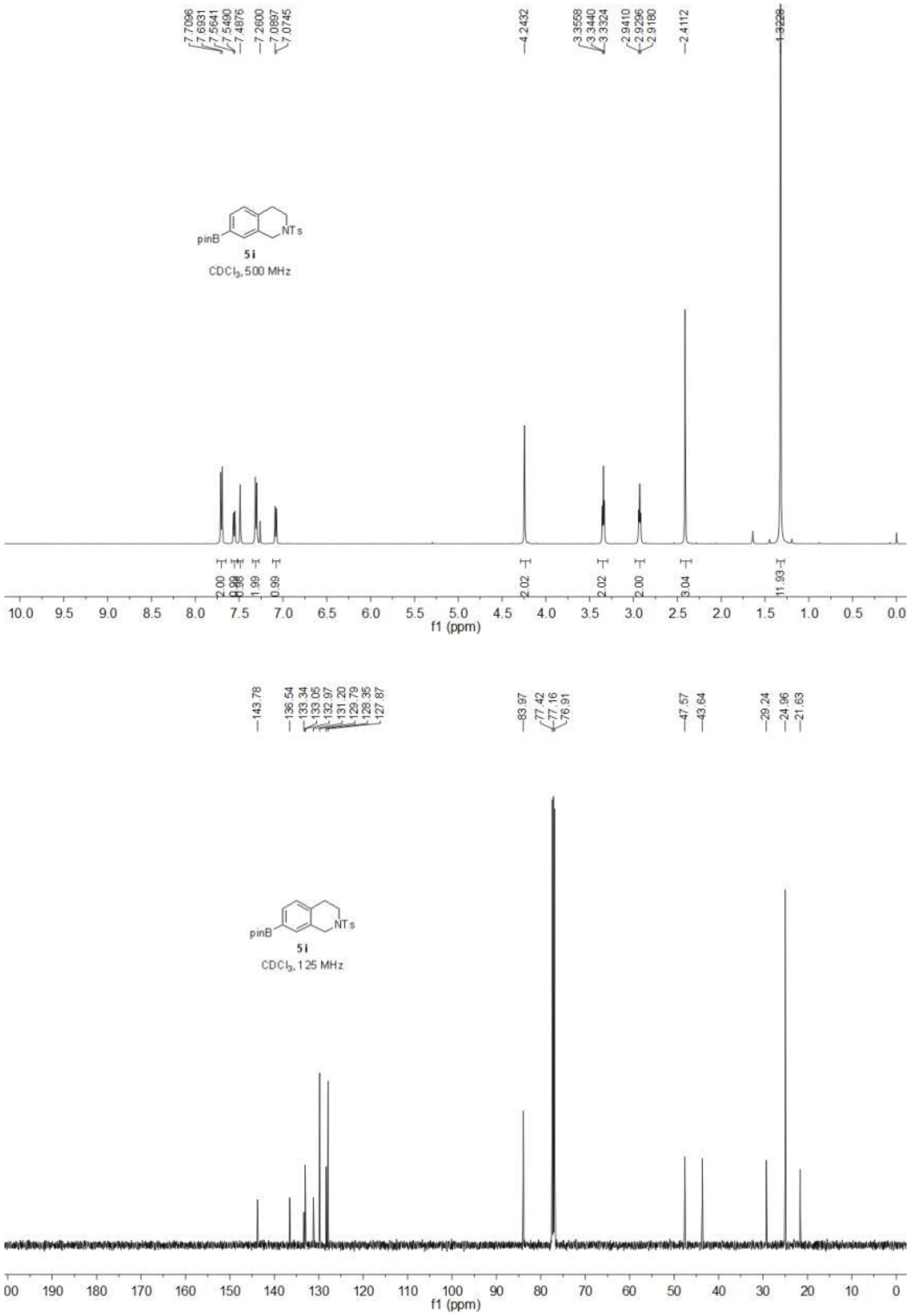

Figure S71. ^{1}H and ^{13}C NMR spectra of **5i**. Related to **Figure 4**.

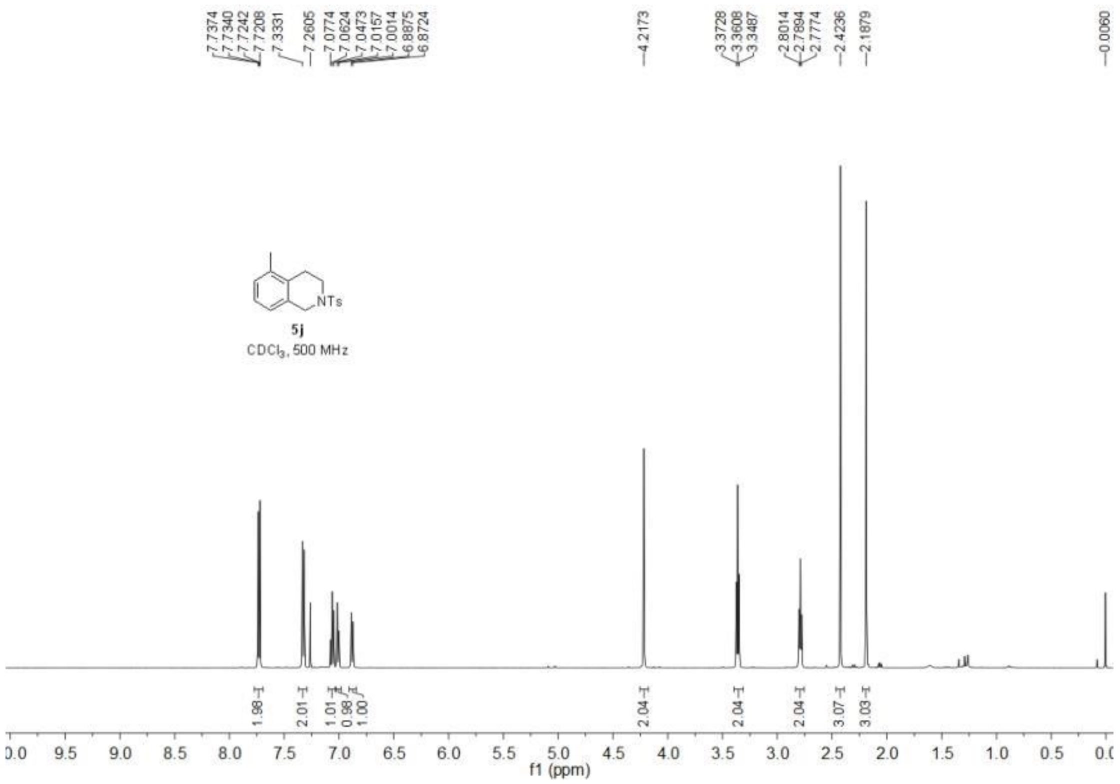

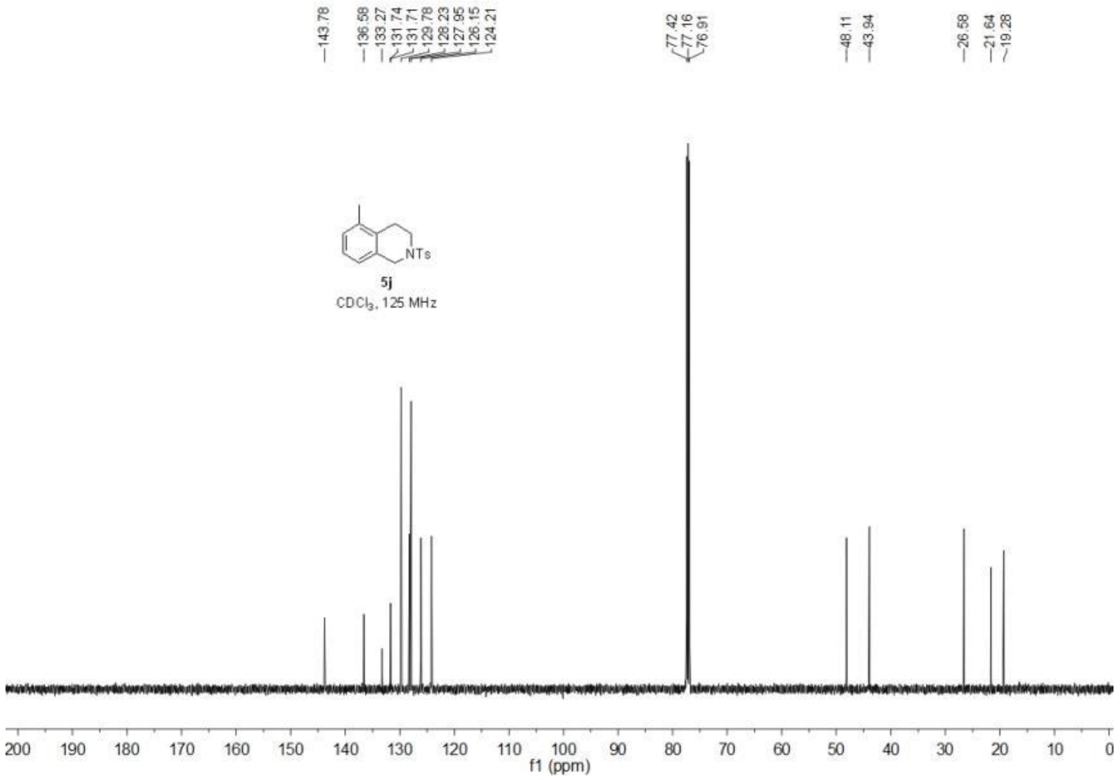

Figure S72. ^1H and ^{13}C NMR spectra of **5j**. Related to **Figure 4**.

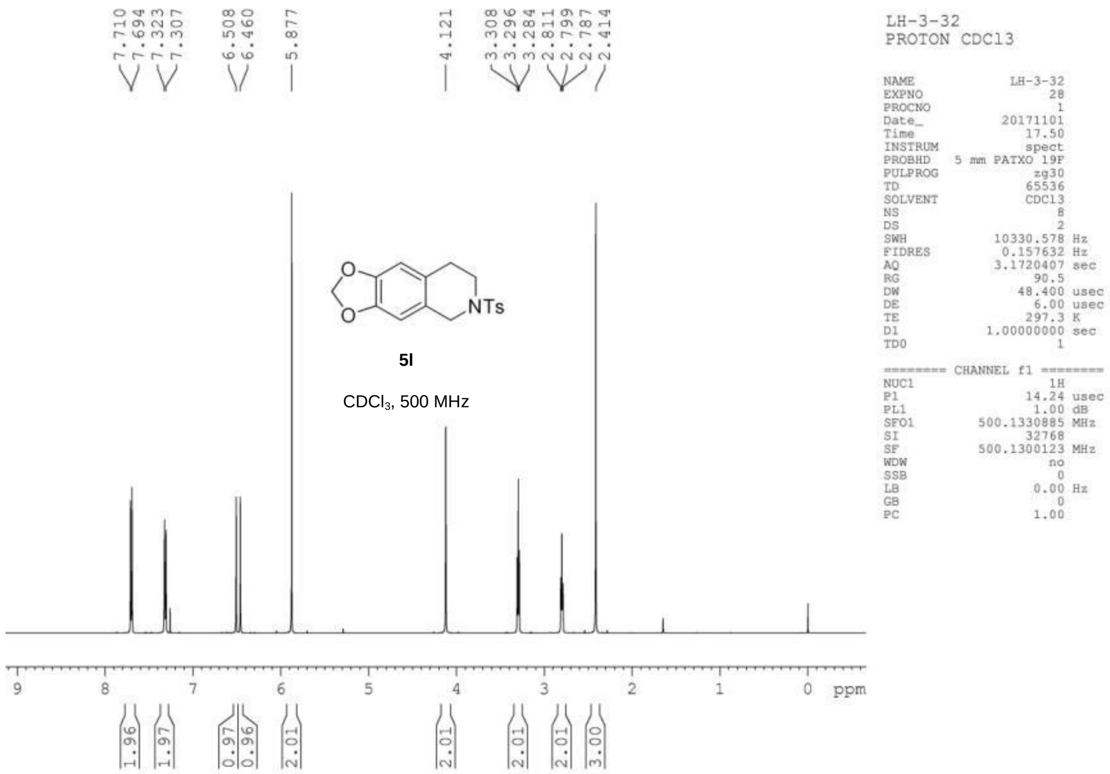

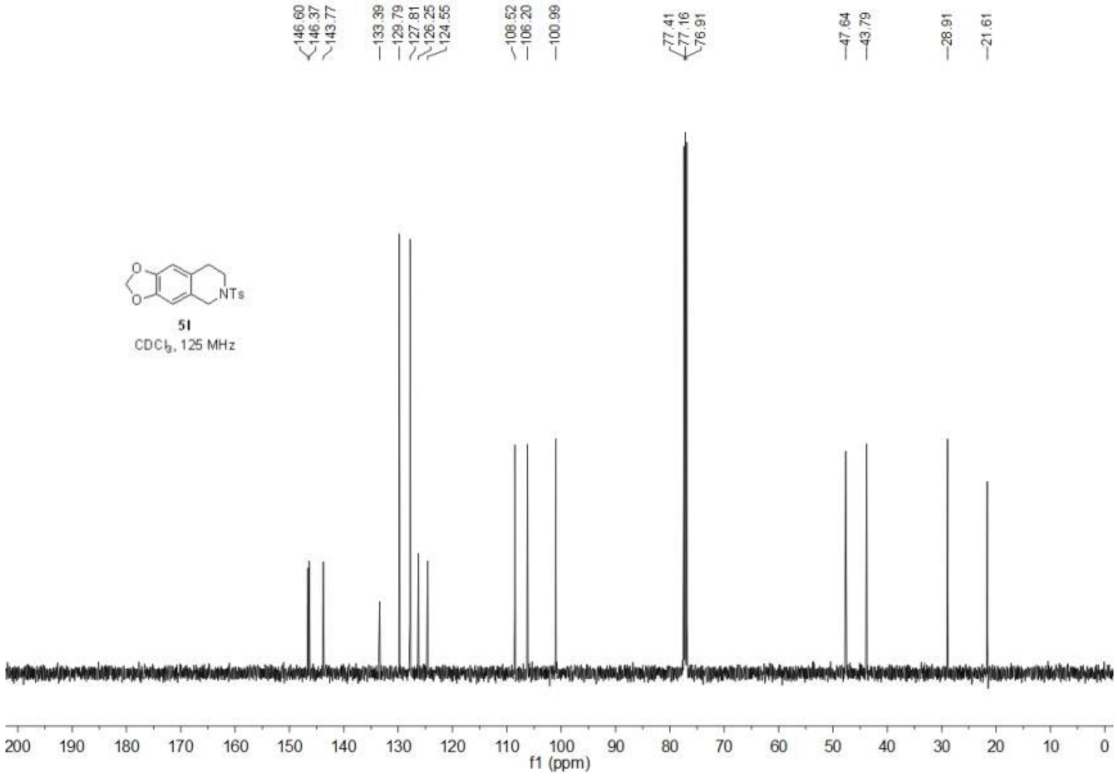

Figure S73. ^{1}H and ^{13}C NMR spectra of **5l**. Related to **Figure 4**.

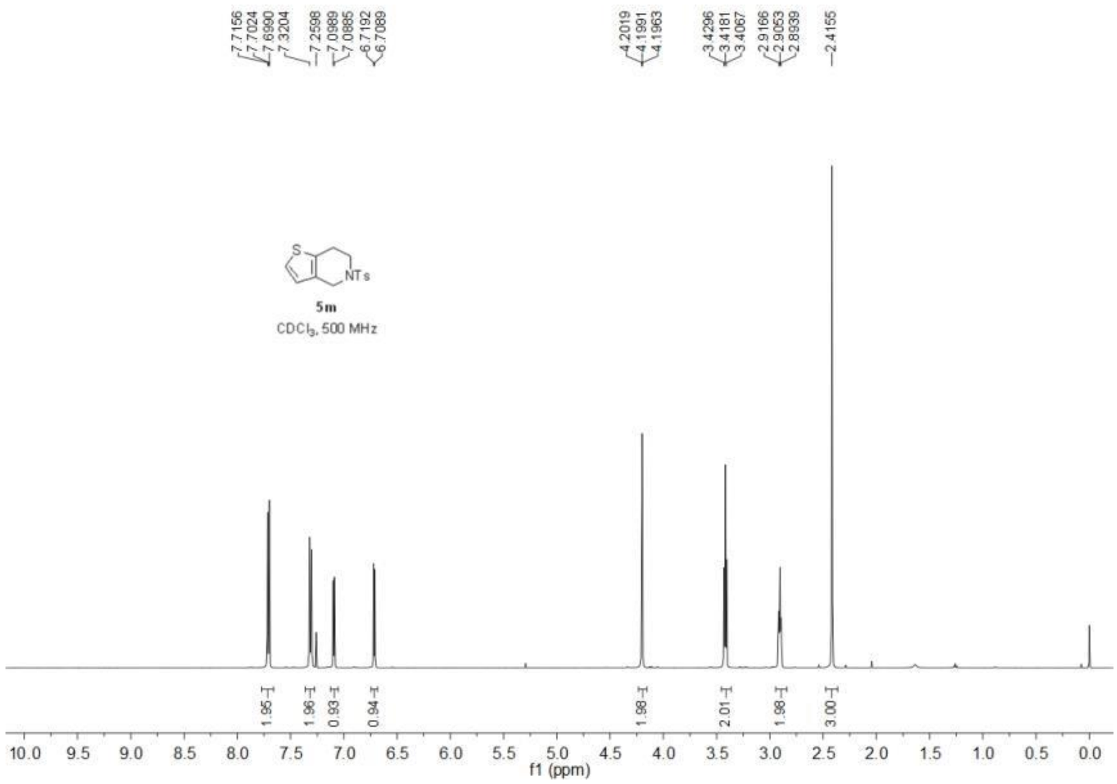

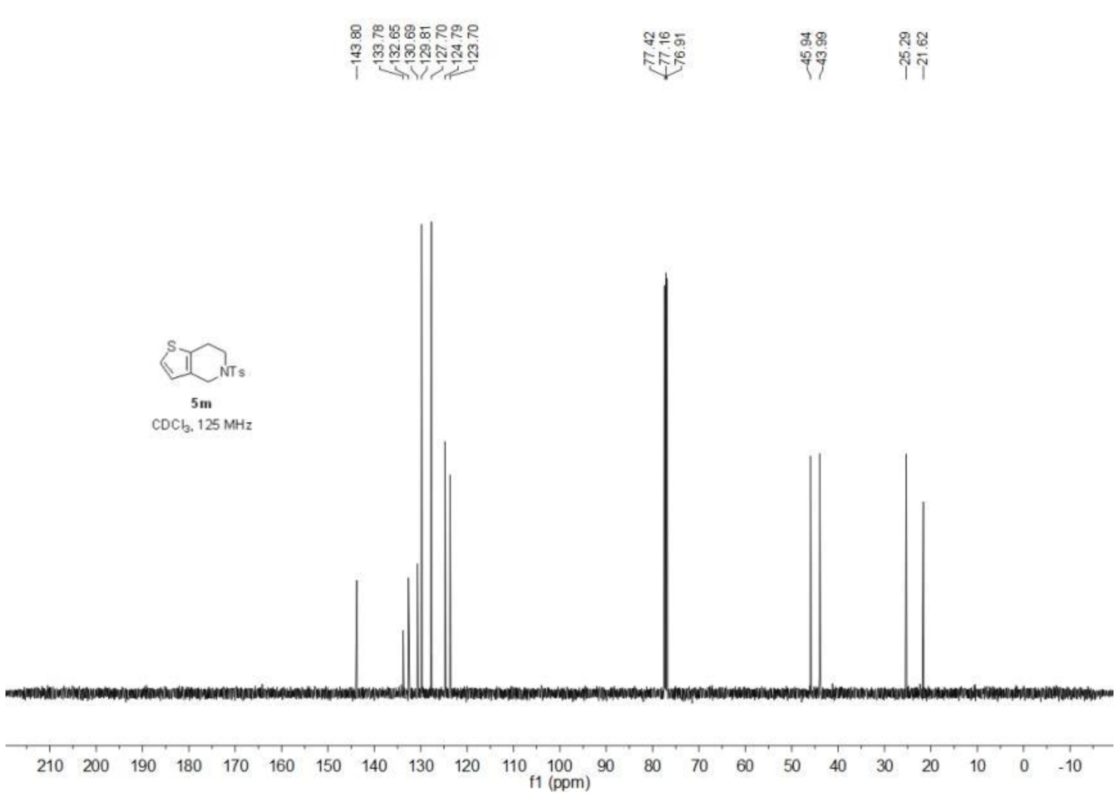

Figure S74. ^{1}H and ^{13}C NMR spectra of **5m.** Related to **Figure 4**.

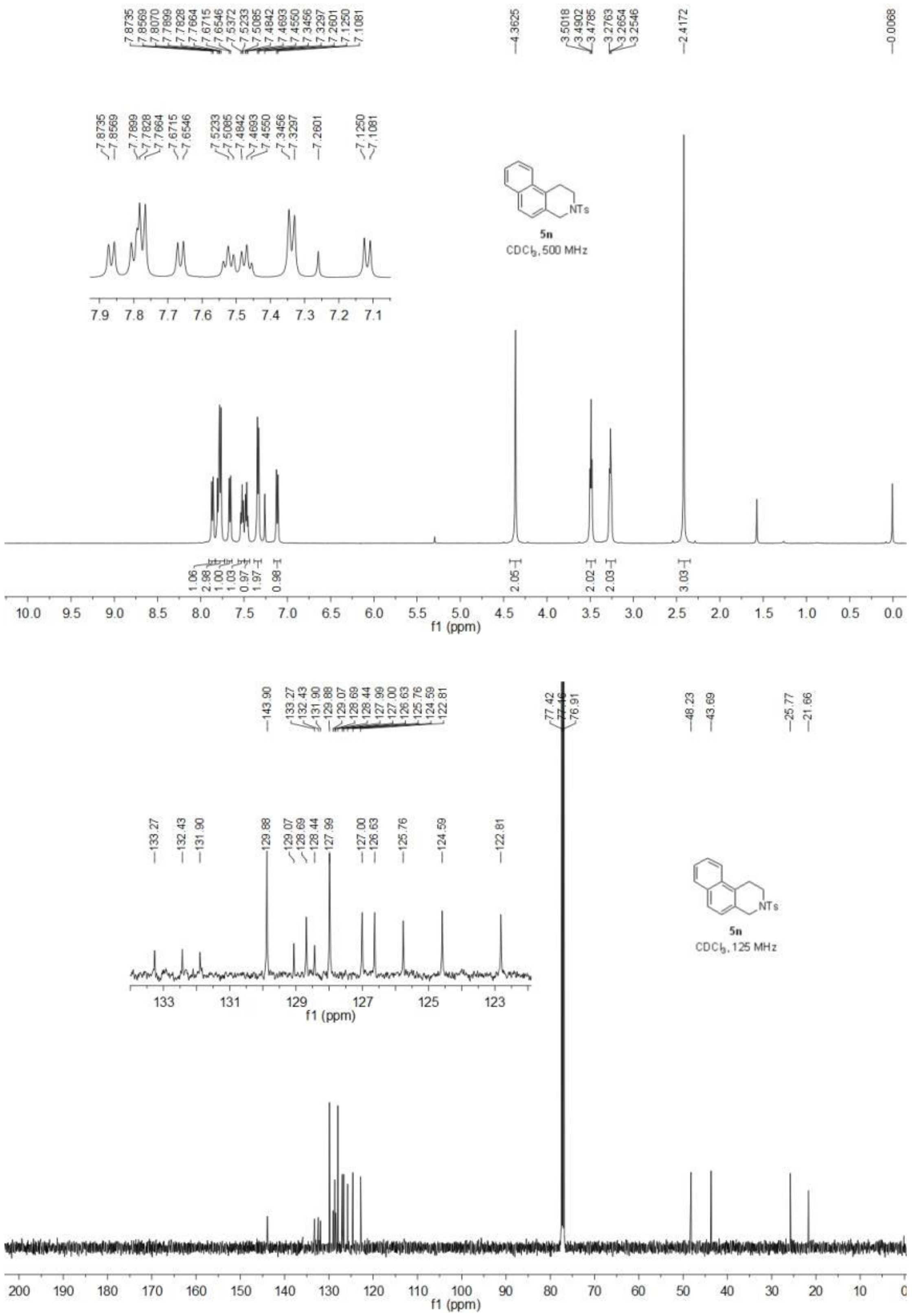

Figure S75. ^{1}H and ^{13}C NMR spectra of **5n**. Related to **Figure 4**.

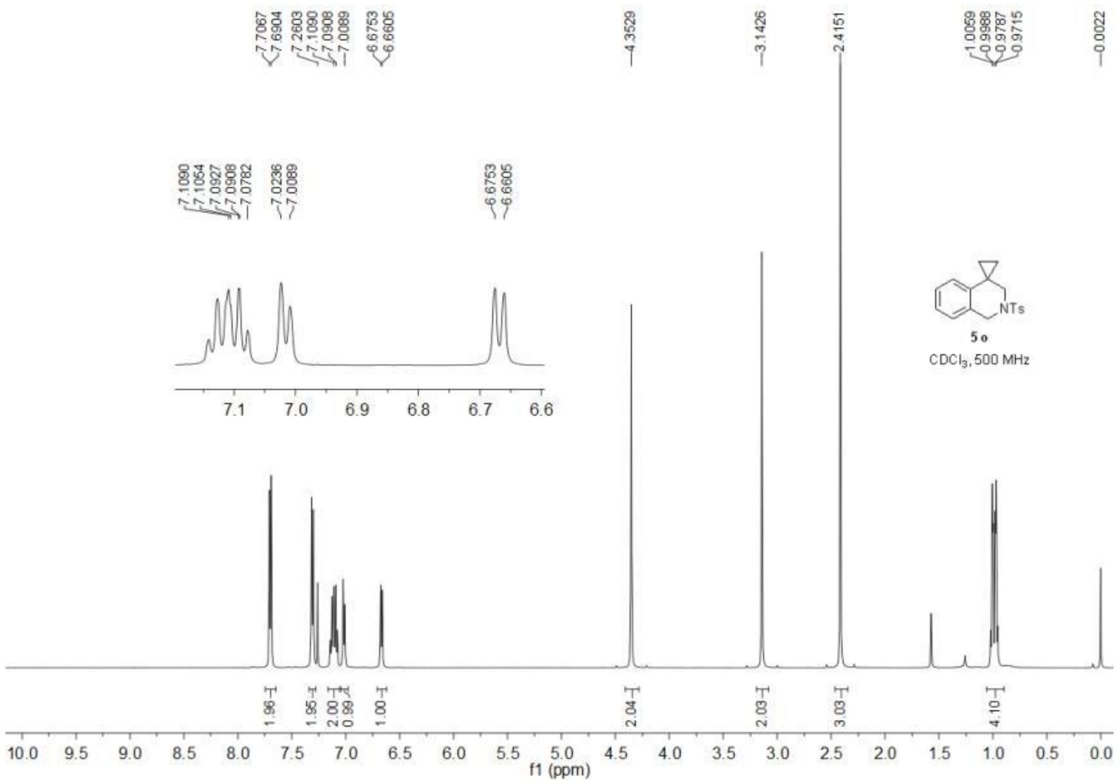

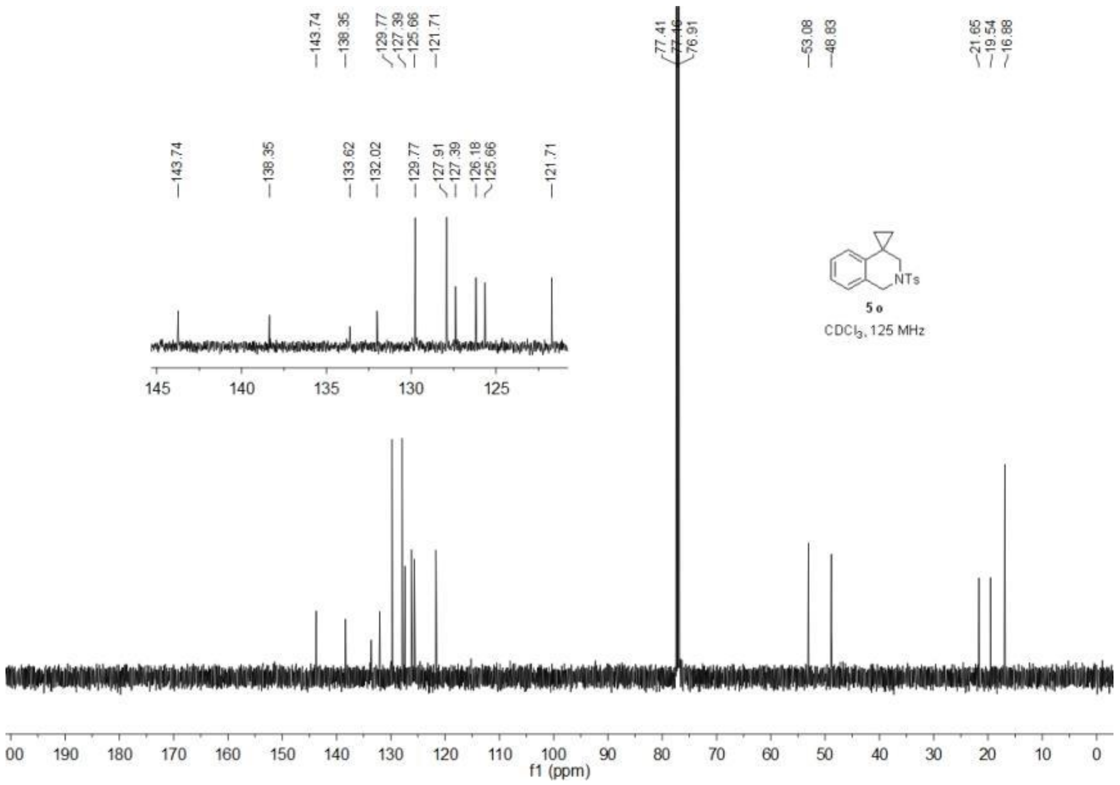

Figure S76. ^{1}H and ^{13}C NMR spectra of **5o**. Related to **Figure 4**.

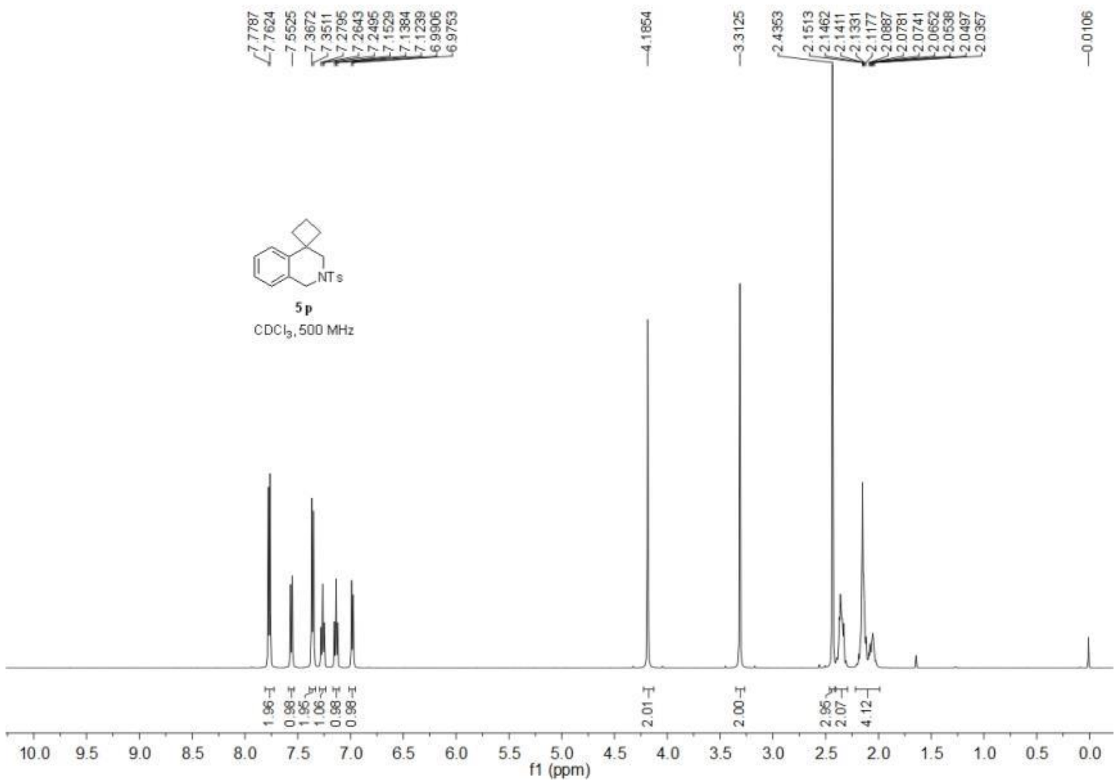

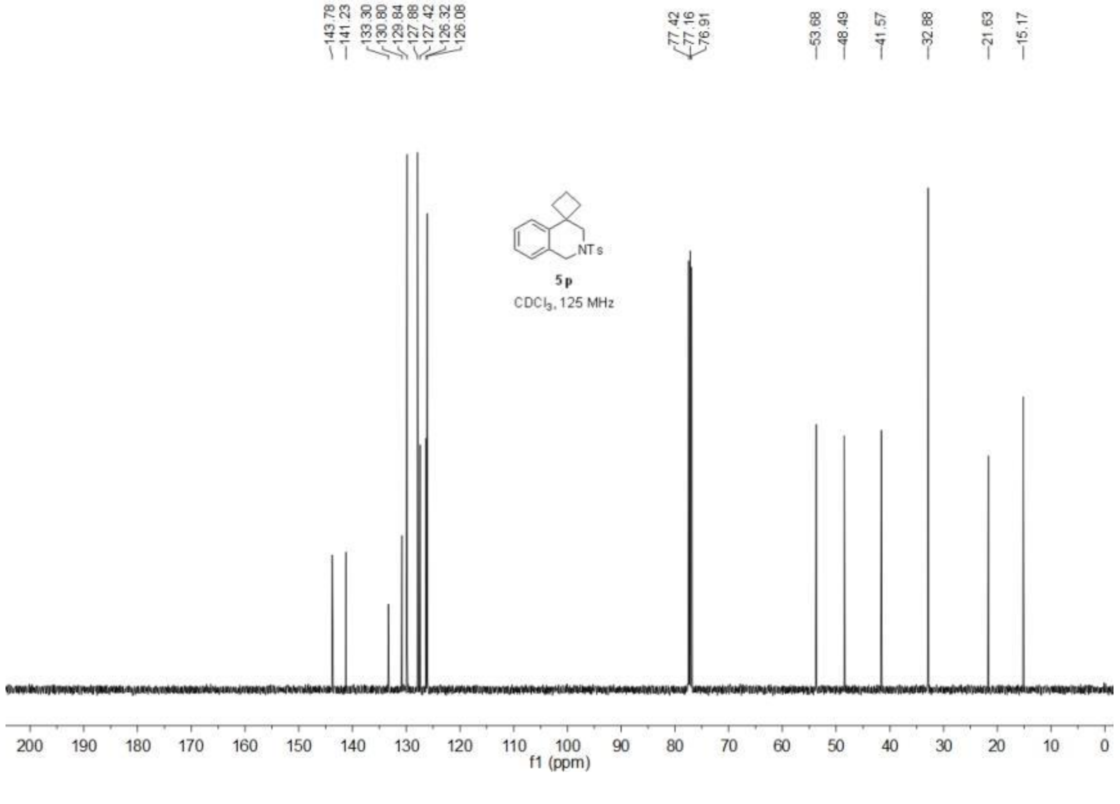

Figure S77. ^{1}H and ^{13}C NMR spectra of **5p**. Related to **Figure 4**.

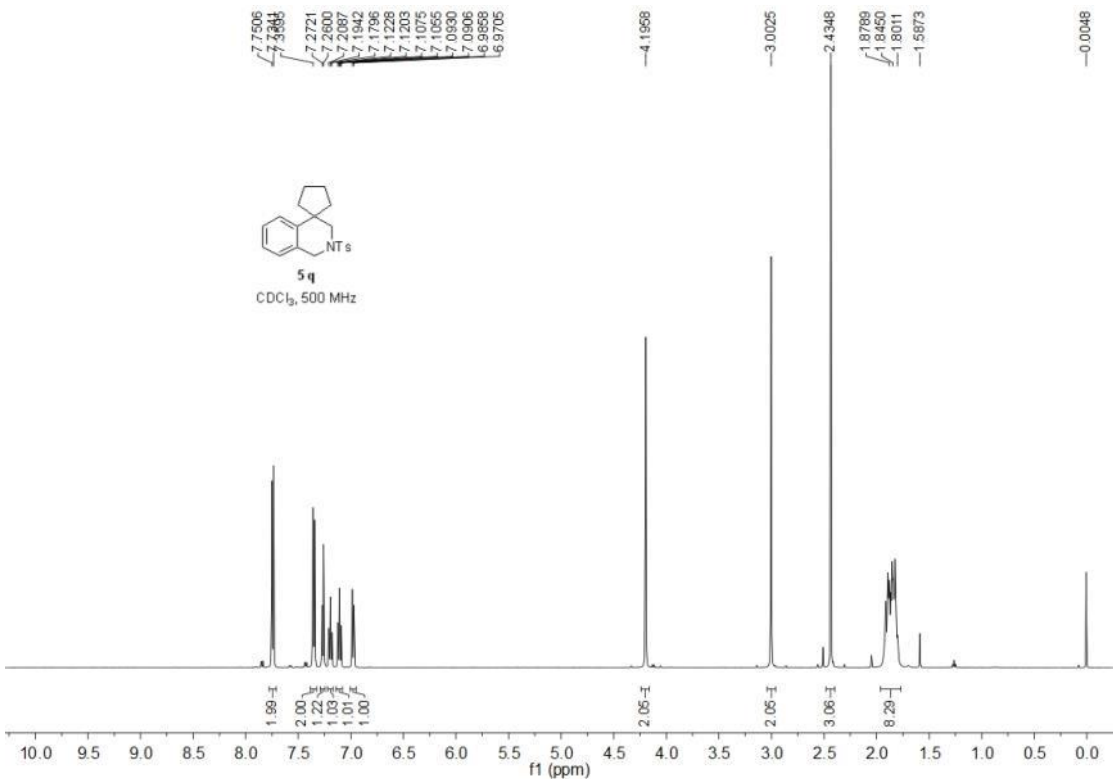

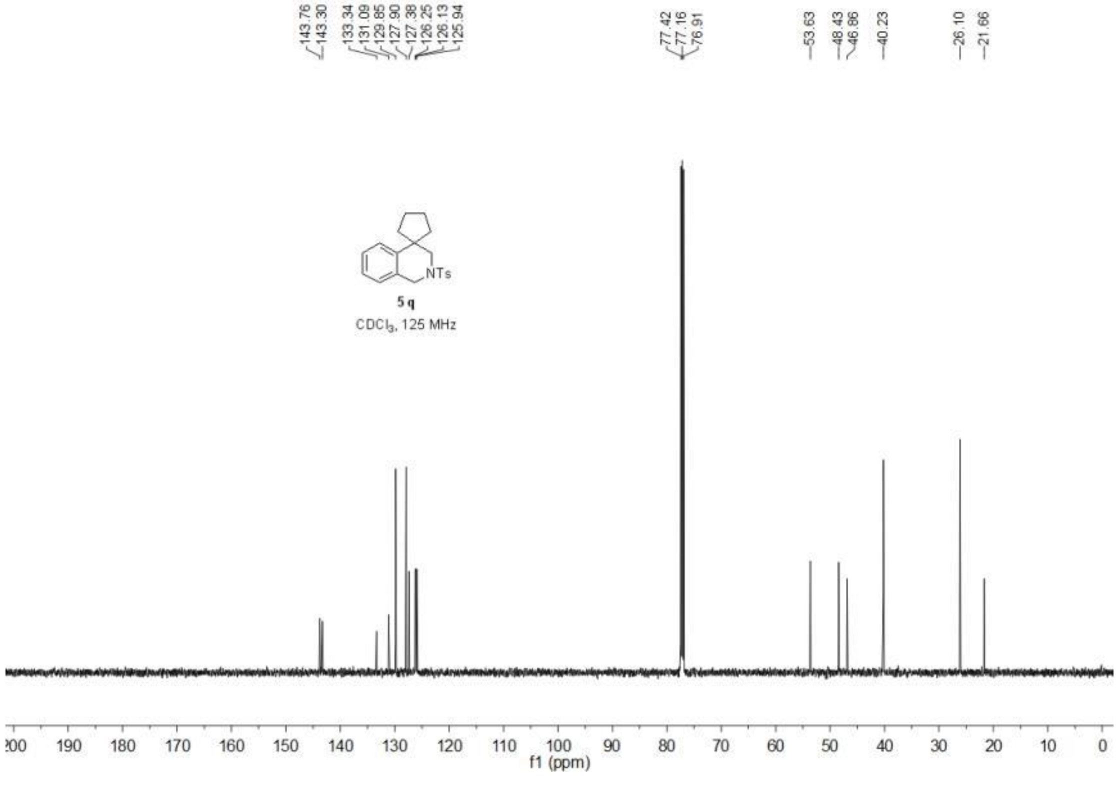

Figure S78. ^{1}H and ^{13}C NMR spectra of **5q.** Related to **Figure 4**.

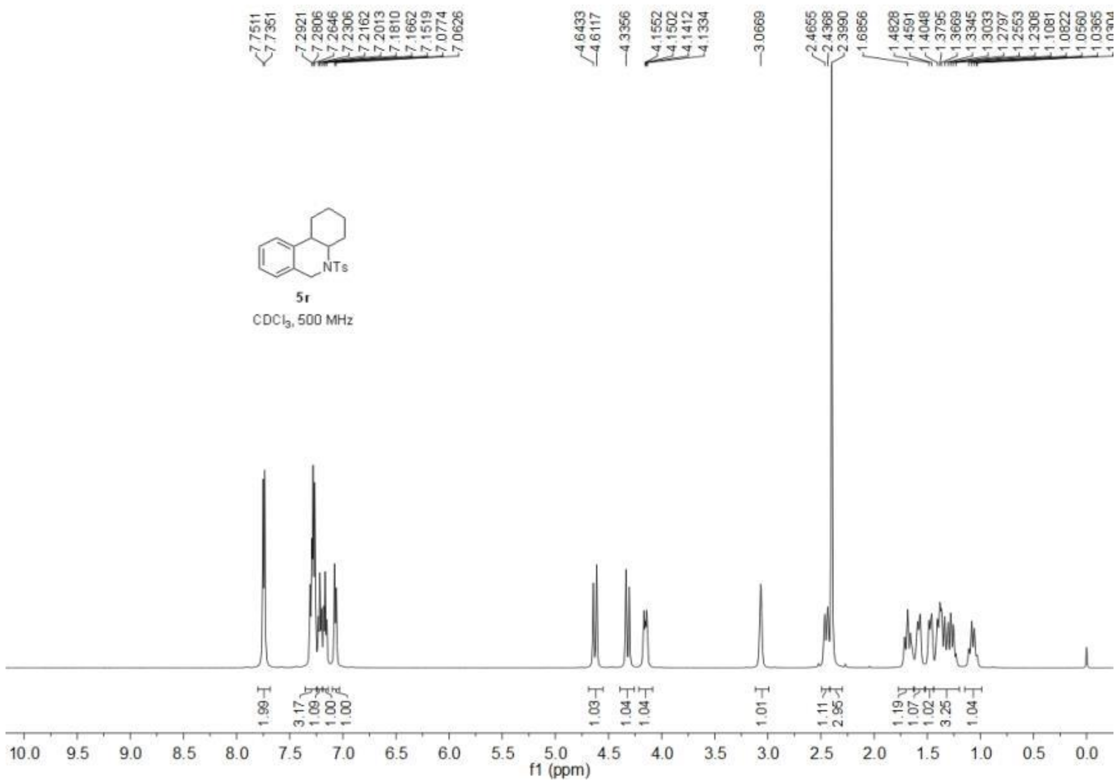

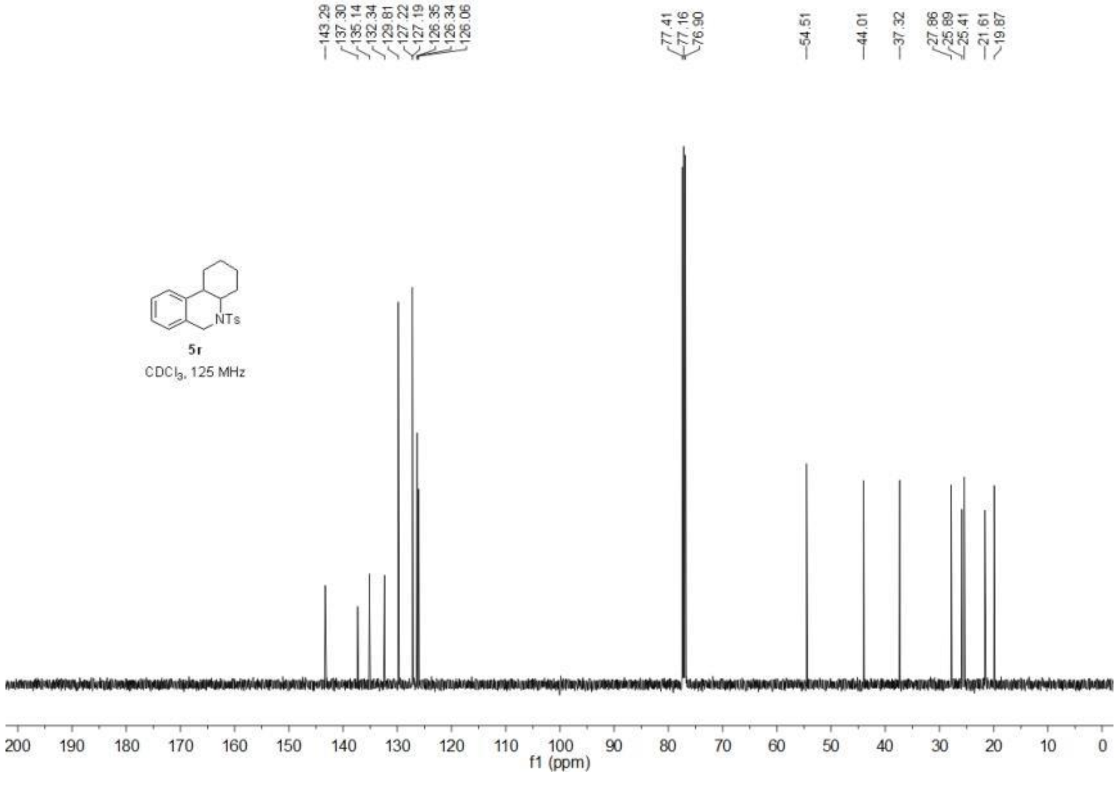

Figure S79. ^{1}H and ^{13}C NMR spectra of **5r**. Related to **Figure 4**.

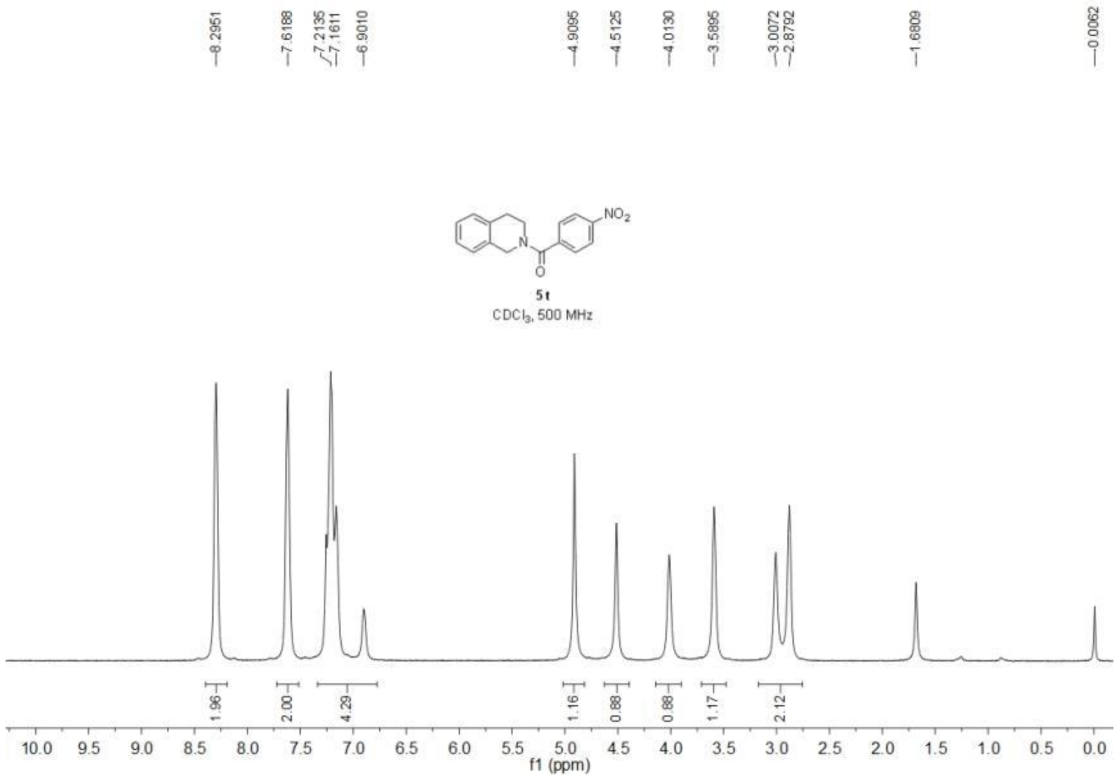

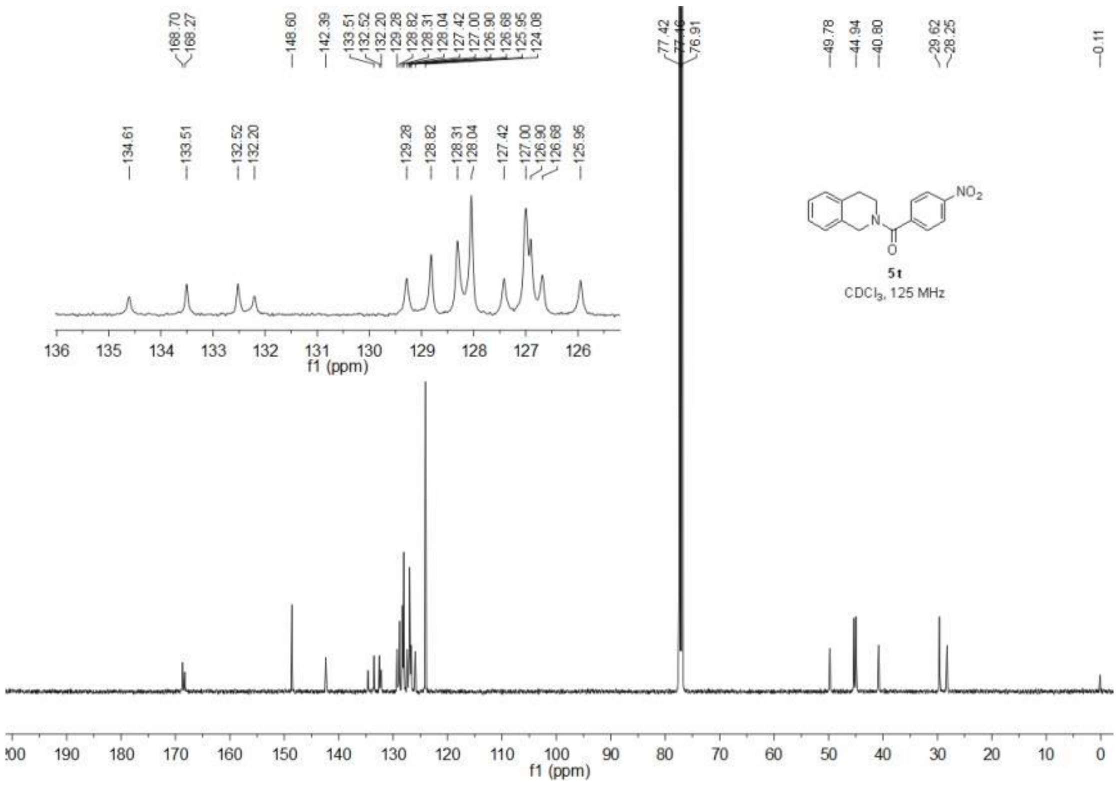

Figure S80. ^{1}H and ^{13}C NMR spectra of **5t**. Related to **Figure 4**.

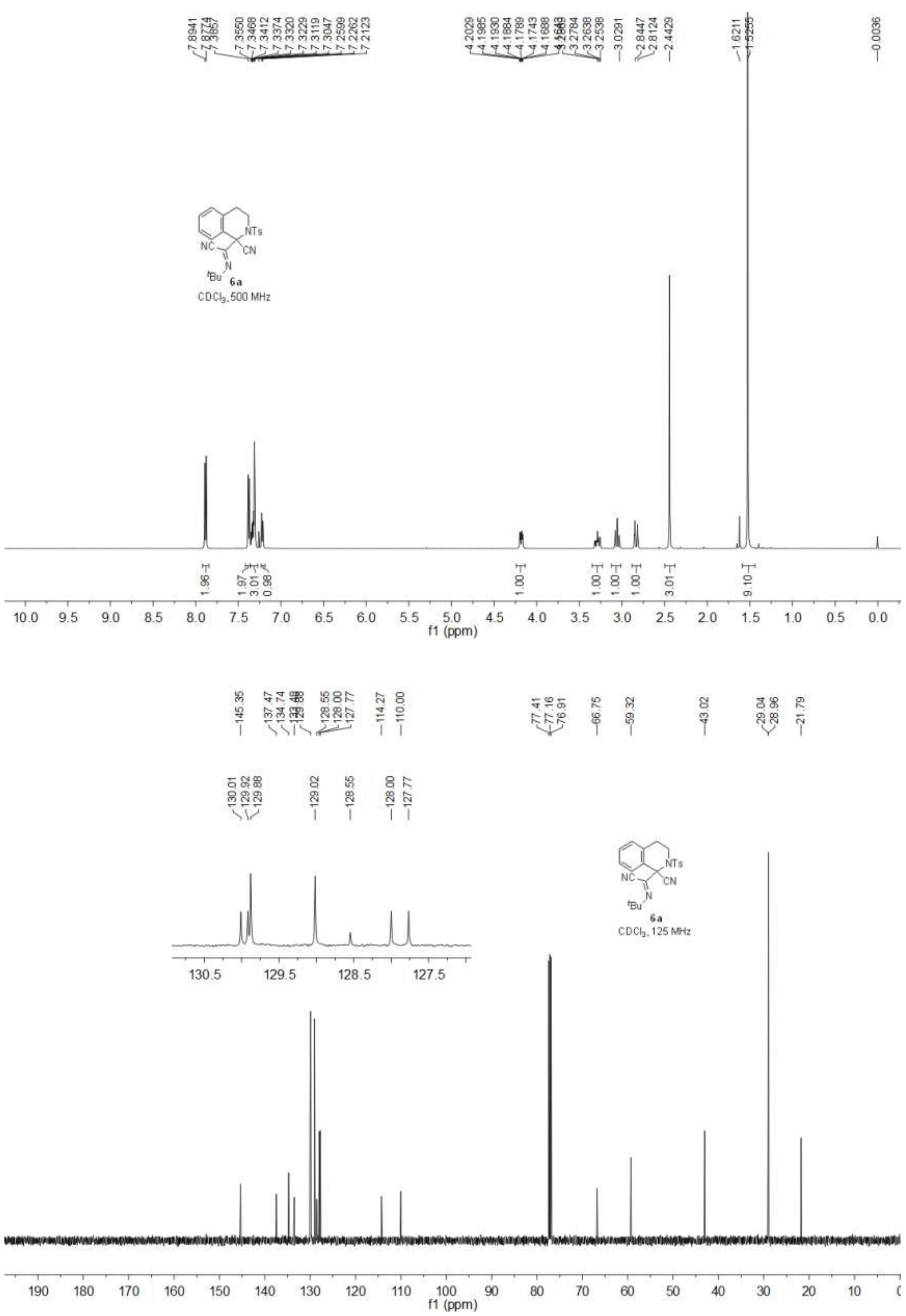

Figure S81. ^{1}H and ^{13}C NMR spectra of **6a**. Related to **Figure 4**.

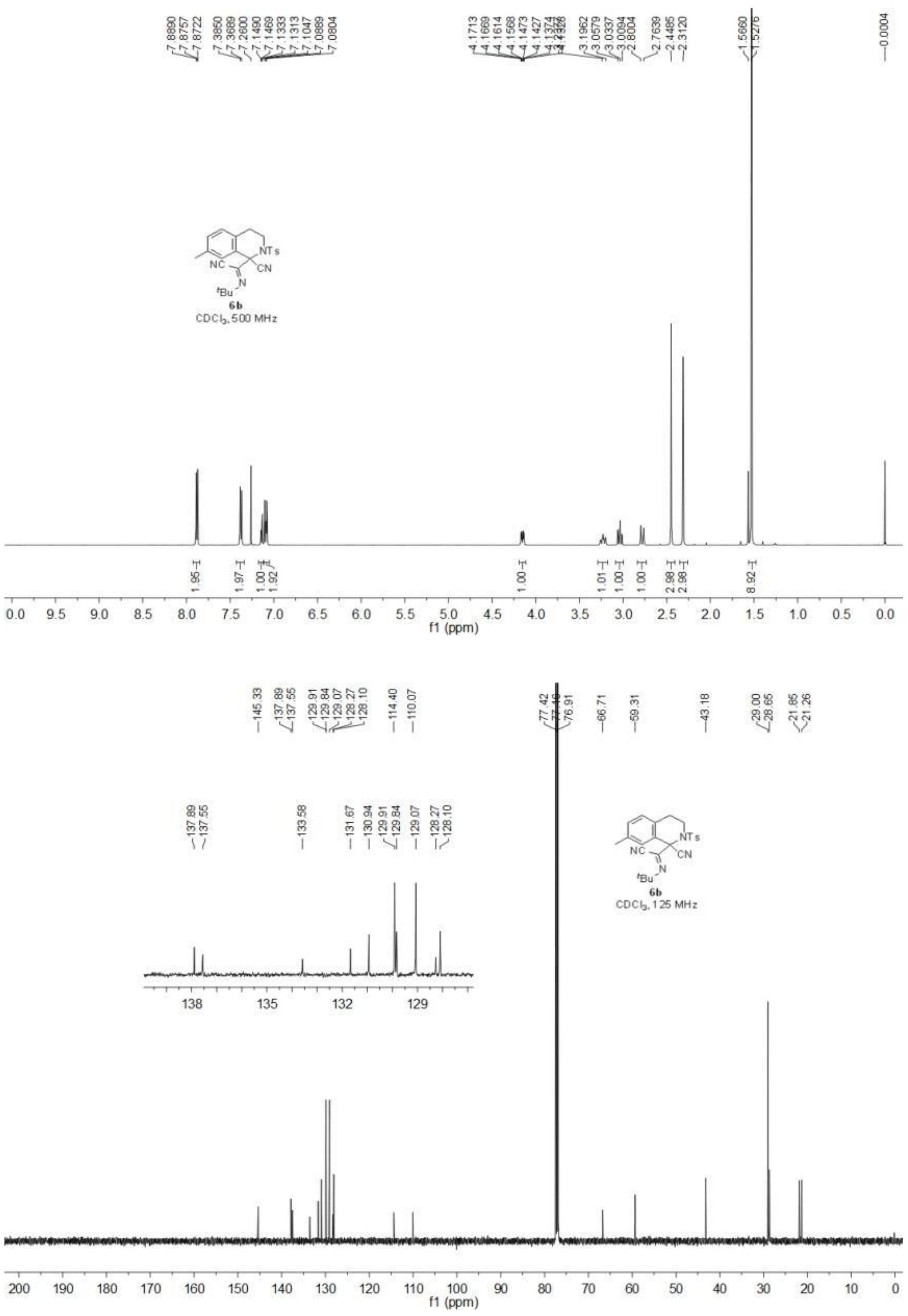

Figure S82. ^{1}H and ^{13}C NMR spectra of **6b**. Related to **Figure 4**.

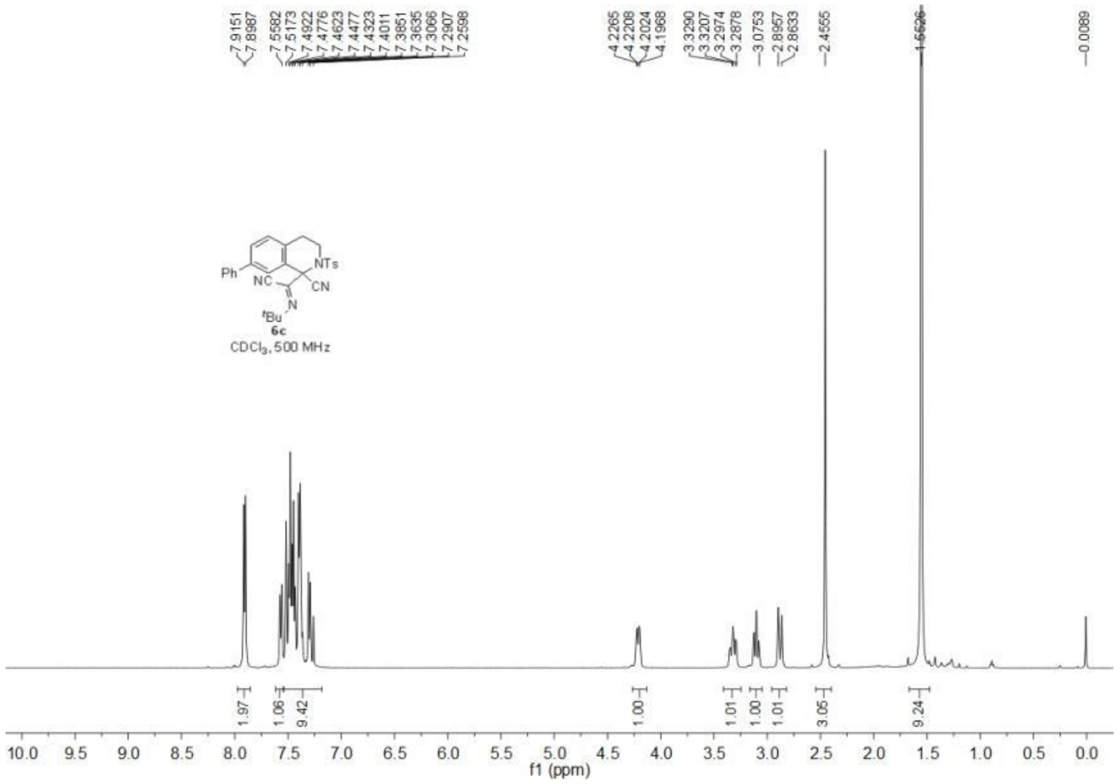

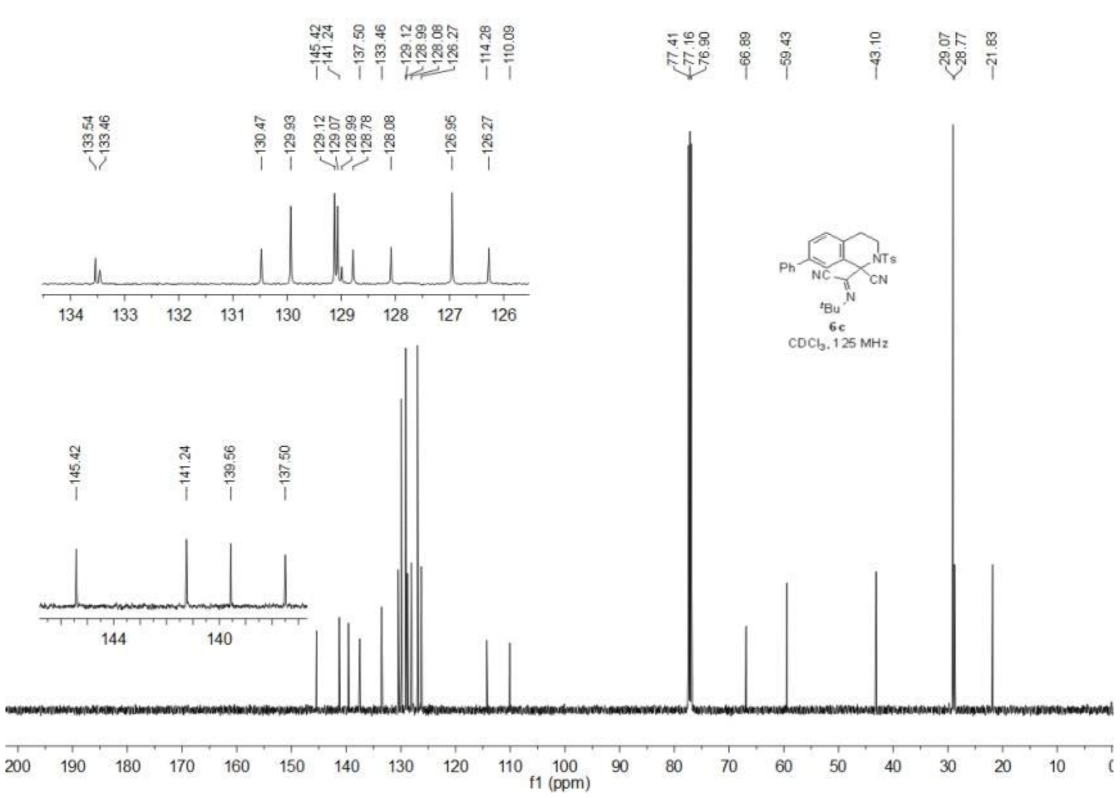

Figure S83. ^{1}H and ^{13}C NMR spectra of **6c**. Related to **Figure 4**.

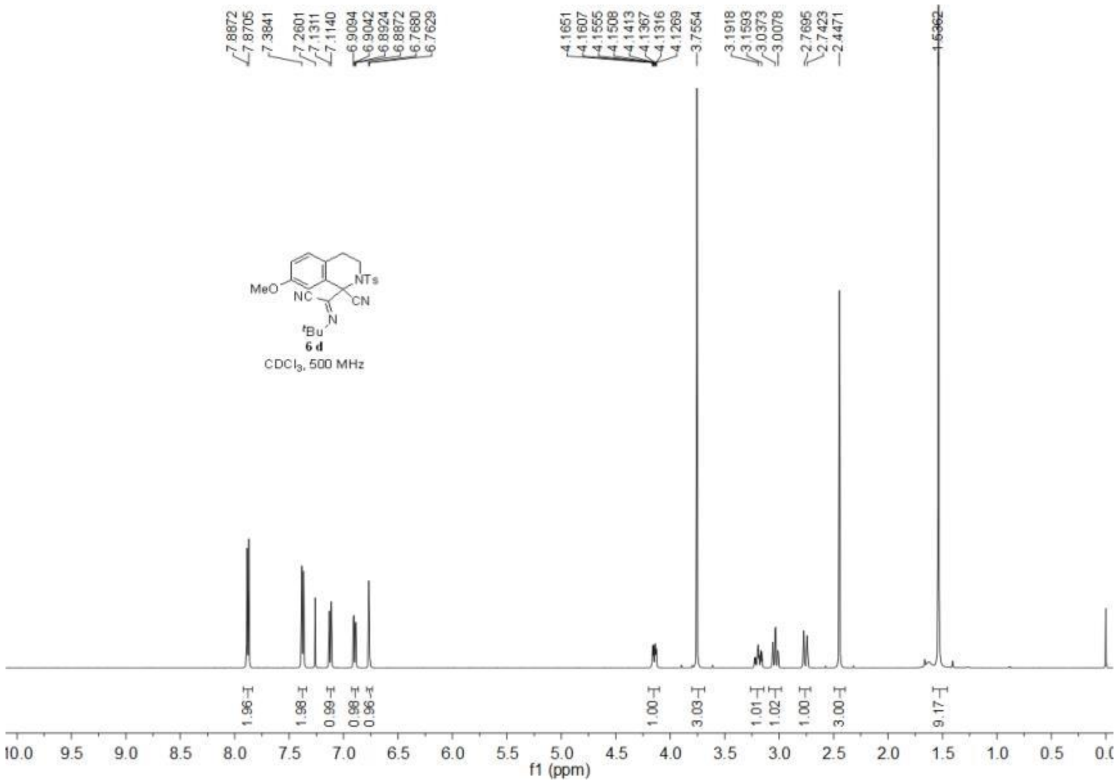

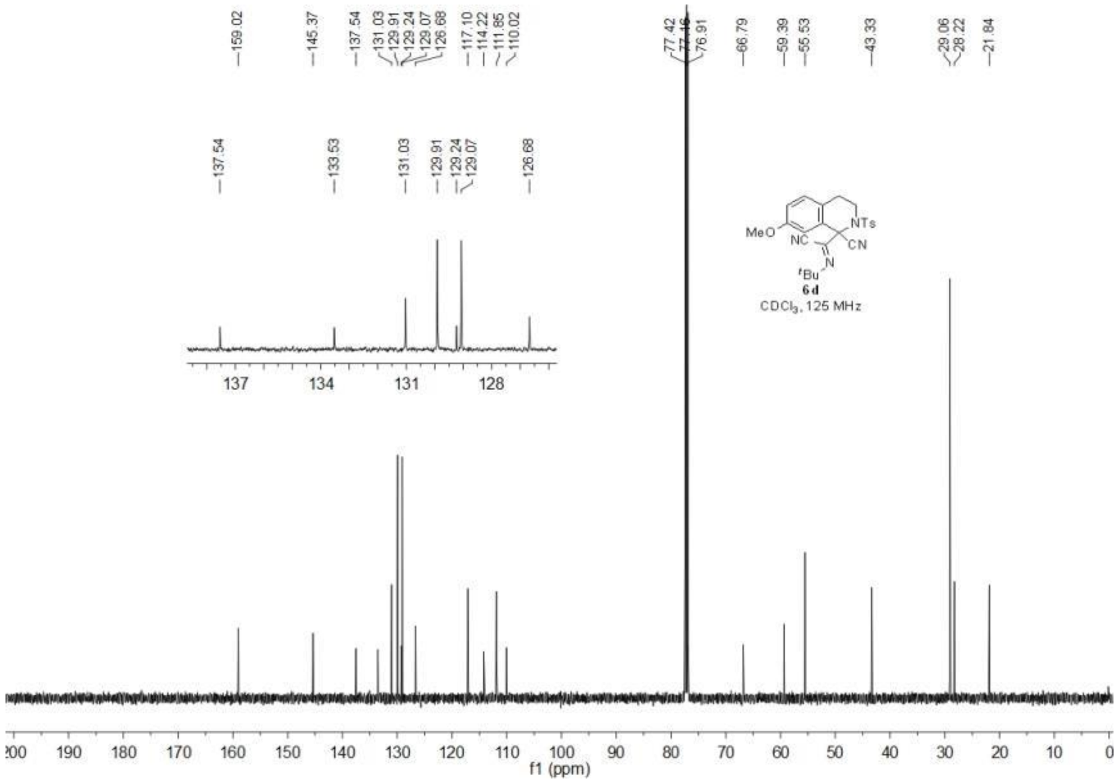

Figure S84. ^{1}H and ^{13}C NMR spectra of **6d**. Related to **Figure 4**.

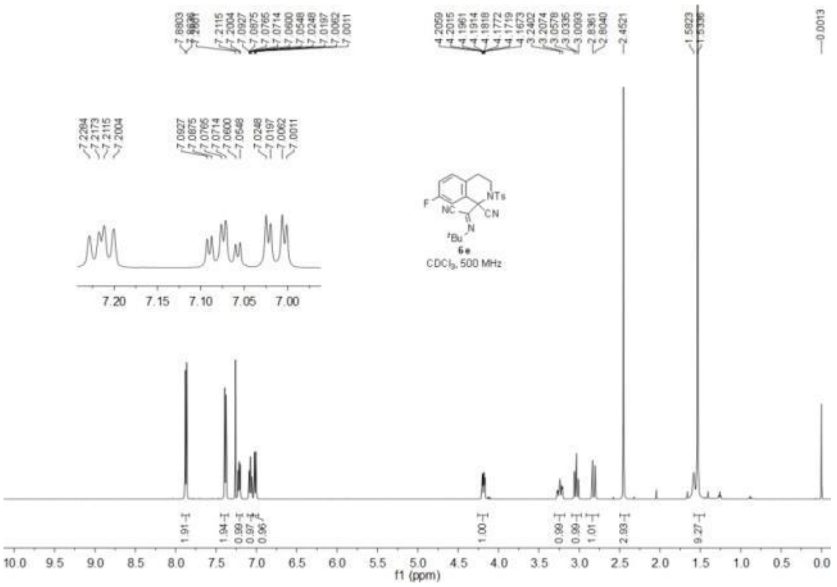

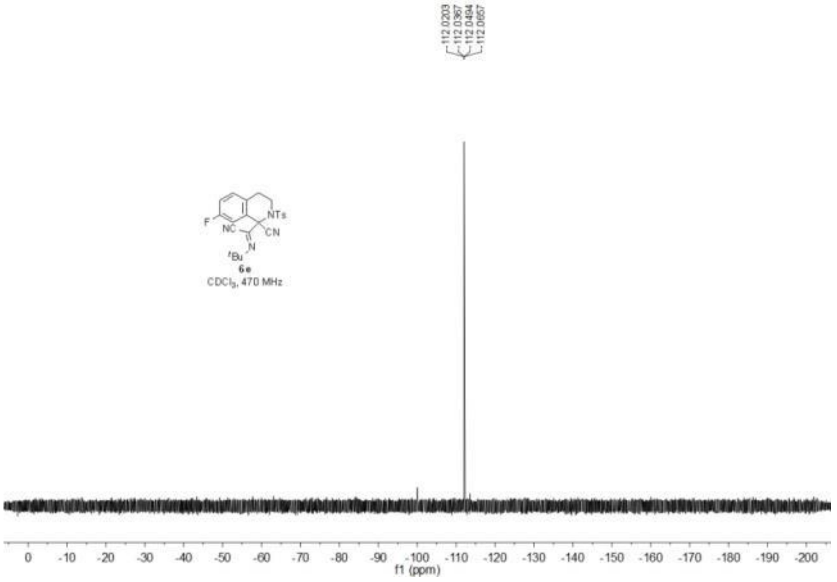

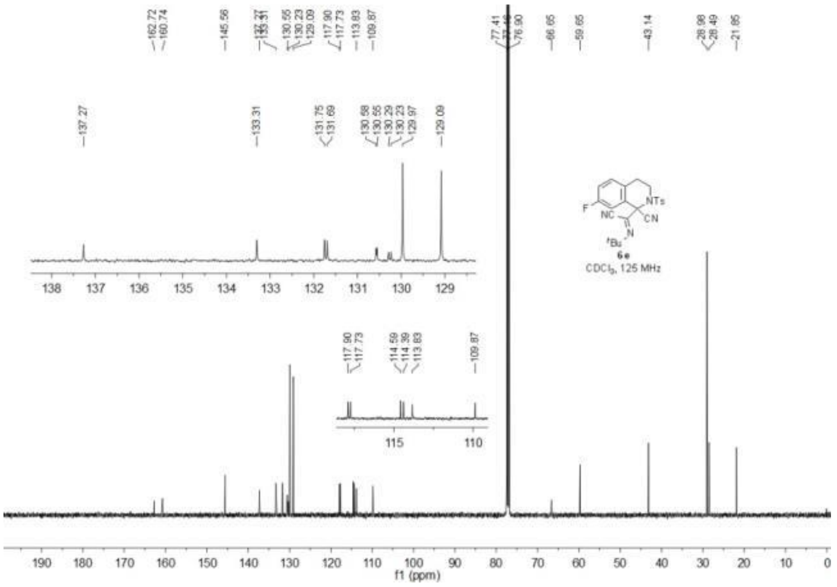

Figure S85. ^{1}H, ^{19}F and ^{13}C NMR spectra of **6e**. Related to **Figure 4**.

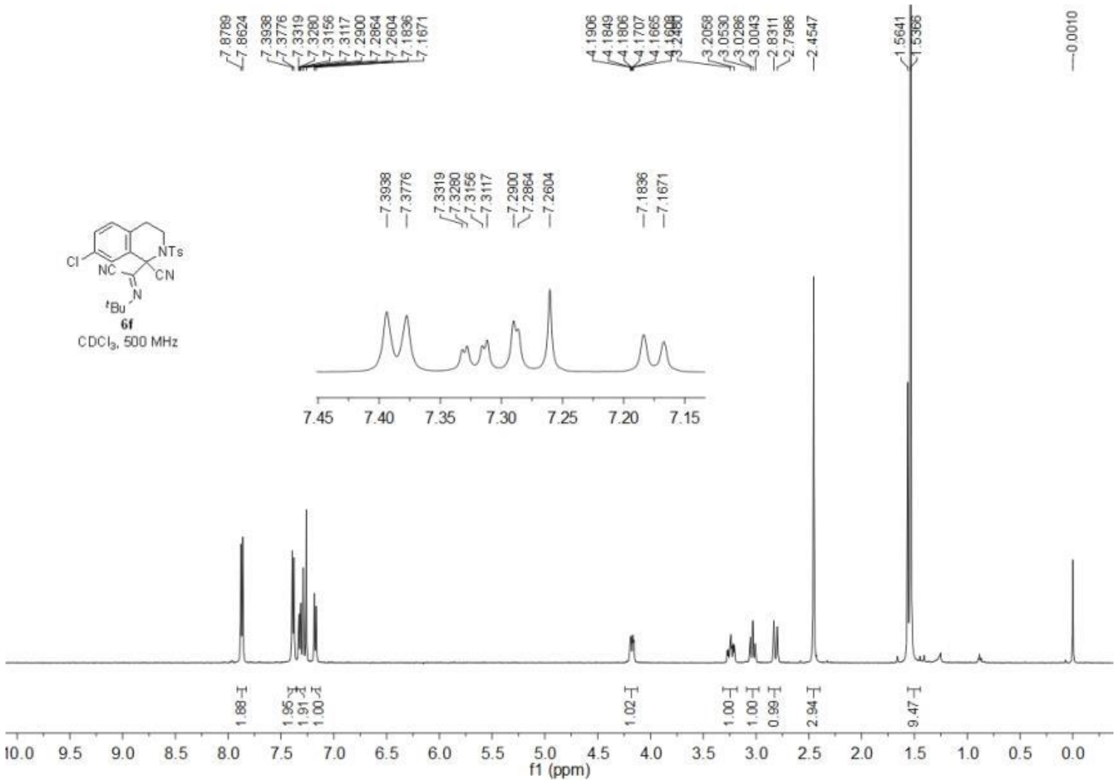

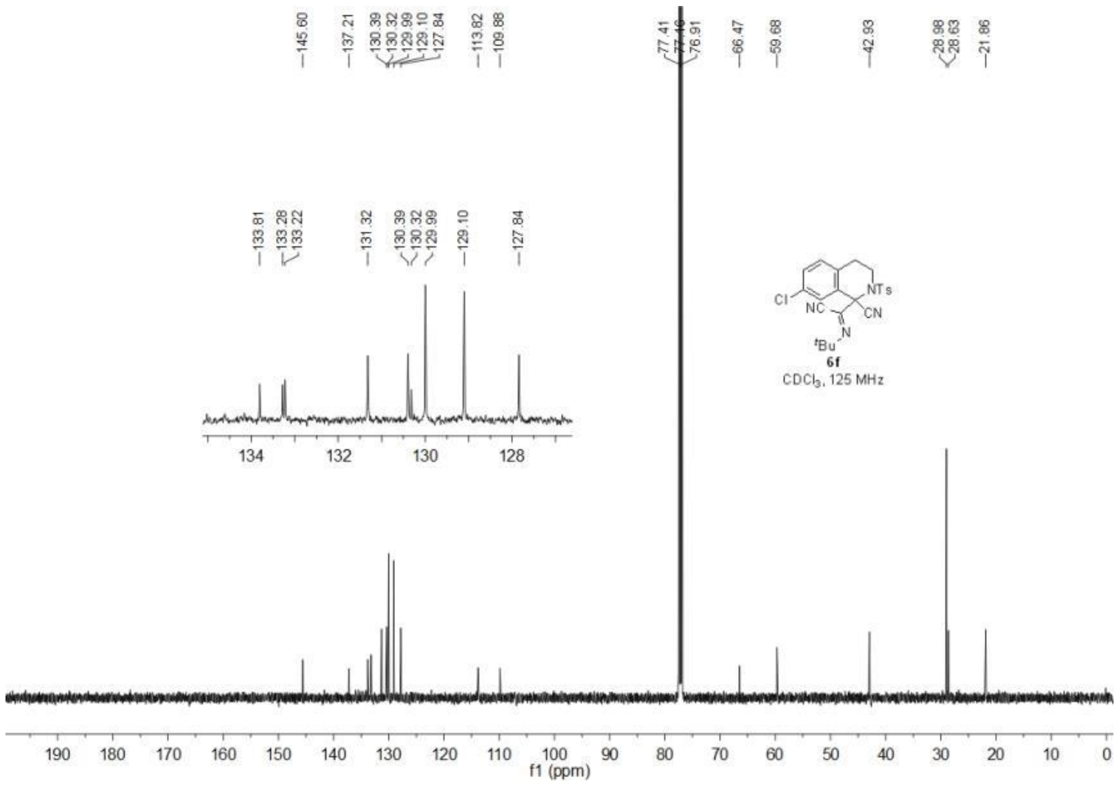

Figure S86. ^{1}H and ^{13}C NMR spectra of **6f**. Related to **Figure 4**.

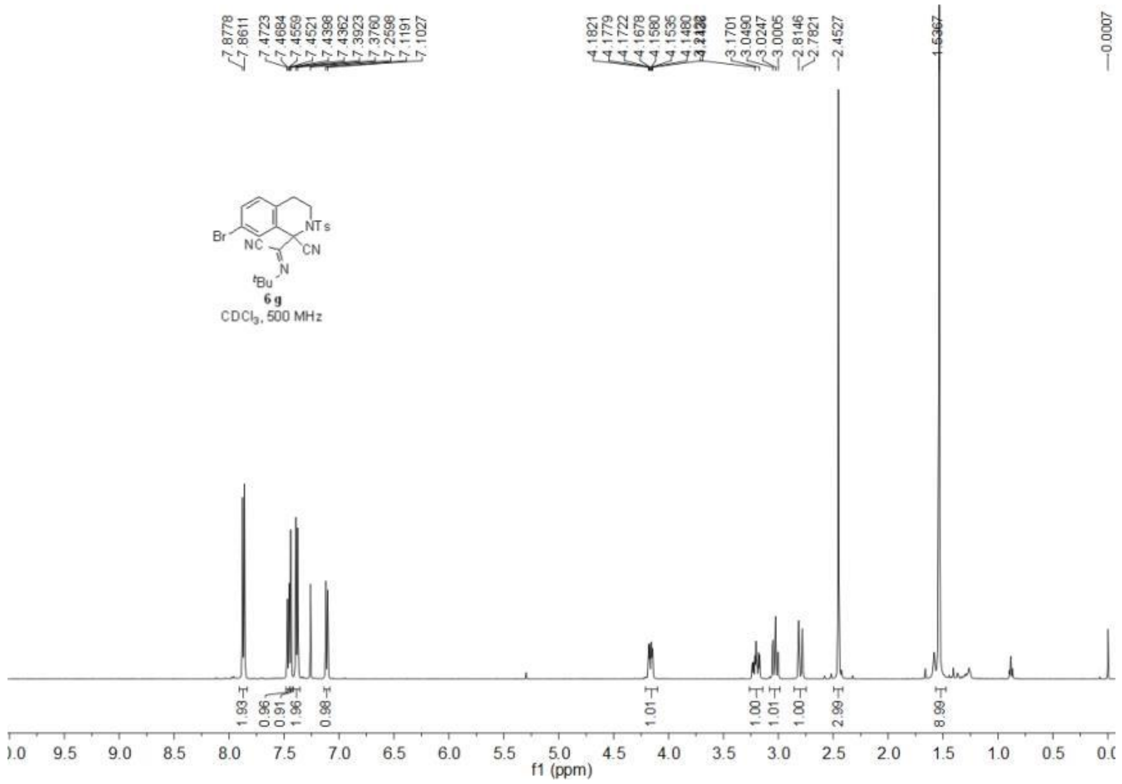

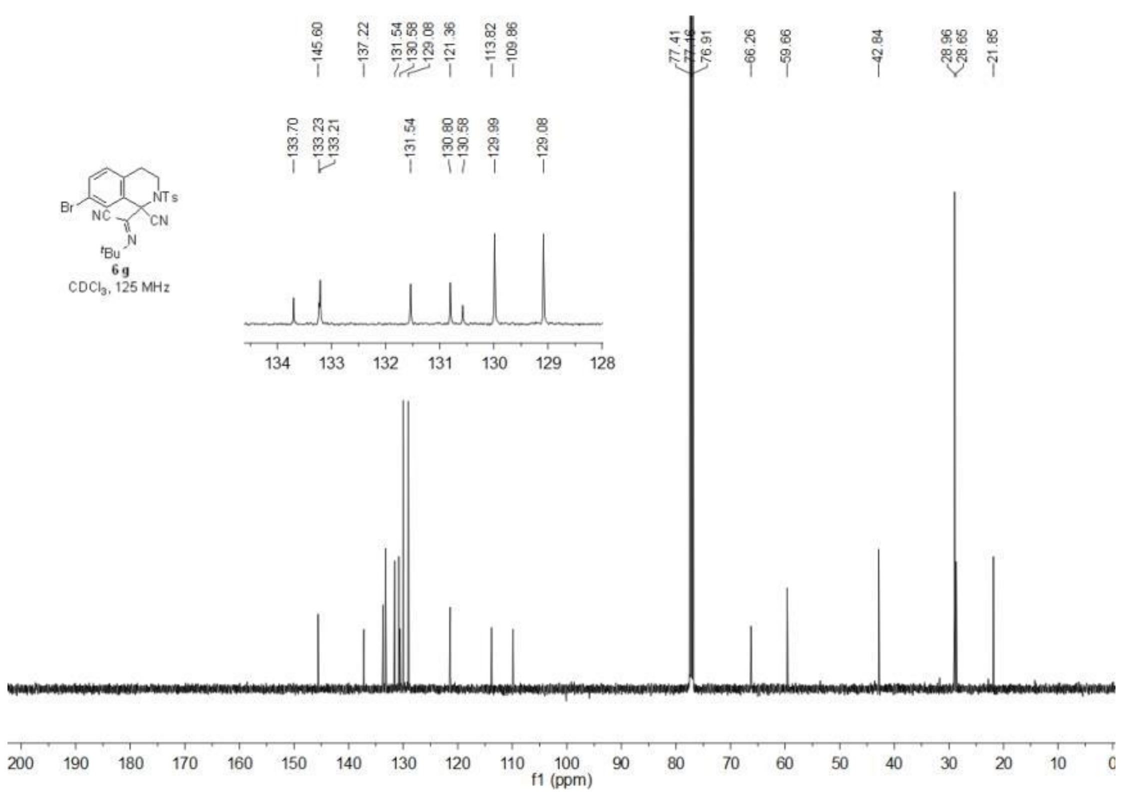

Figure S87. ^{1}H and ^{13}C NMR spectra of **6g.** Related to **Figure 4**.

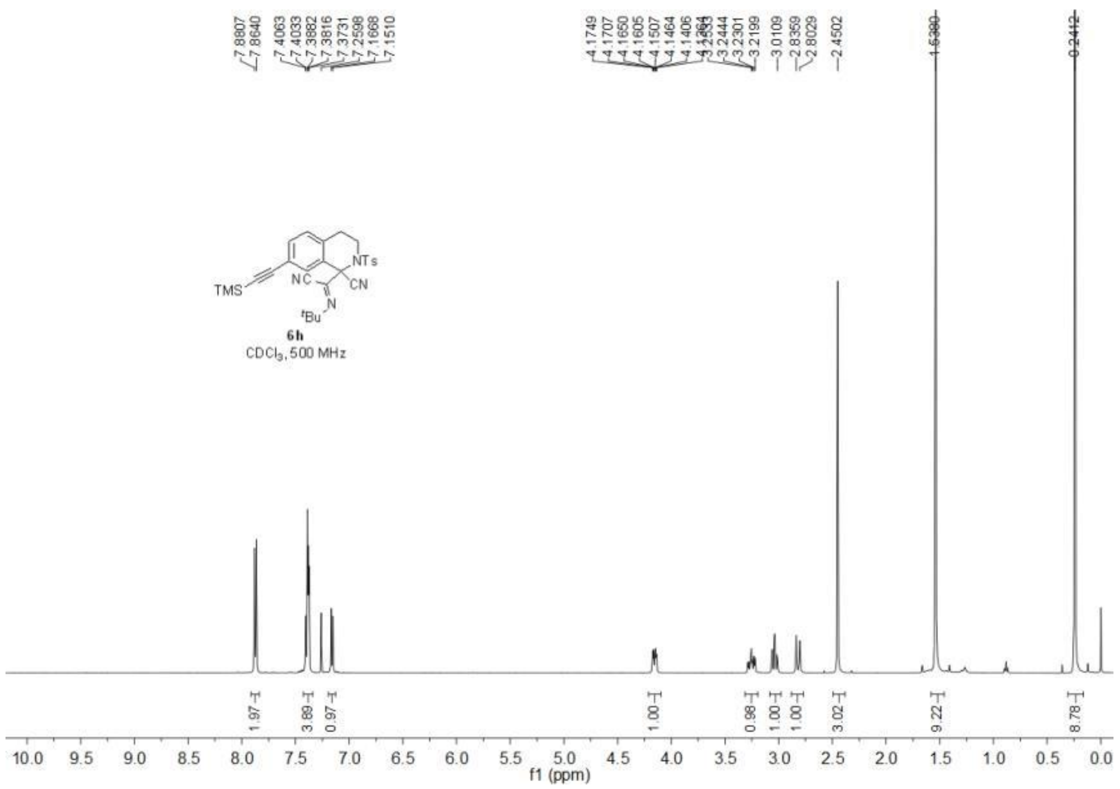

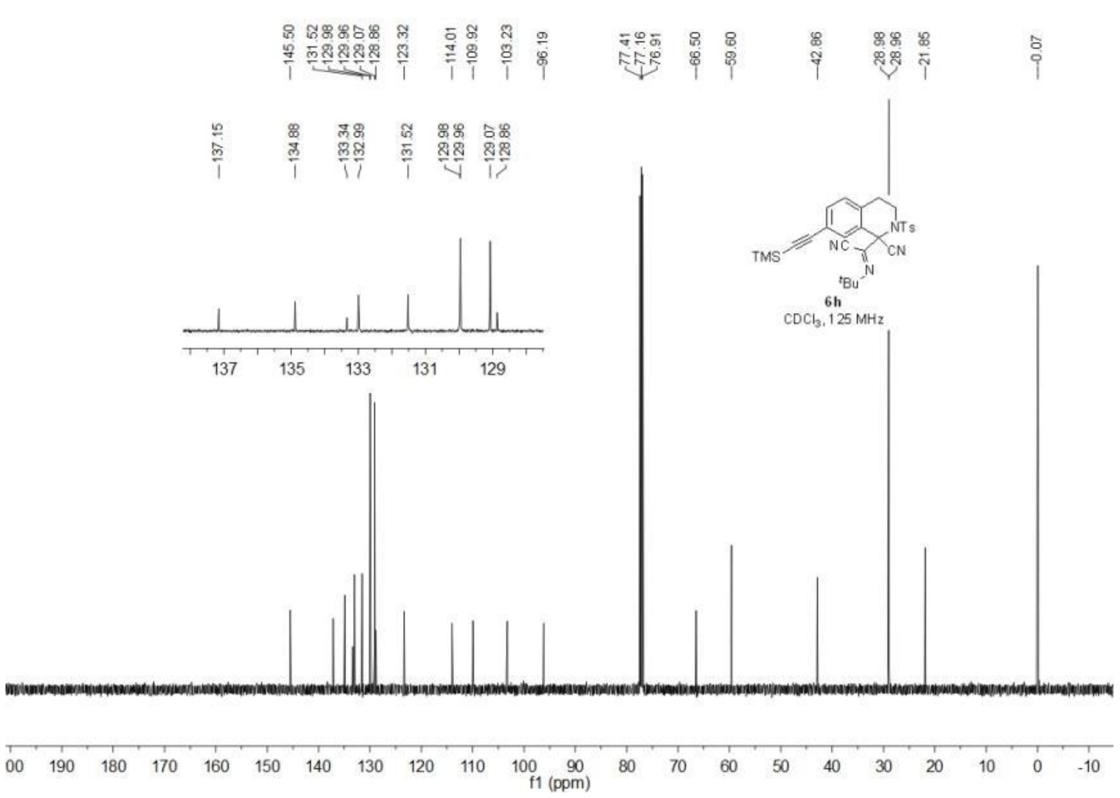

Figure S88. ^{1}H and ^{13}C NMR spectra of **6h**. Related to **Figure 4**.

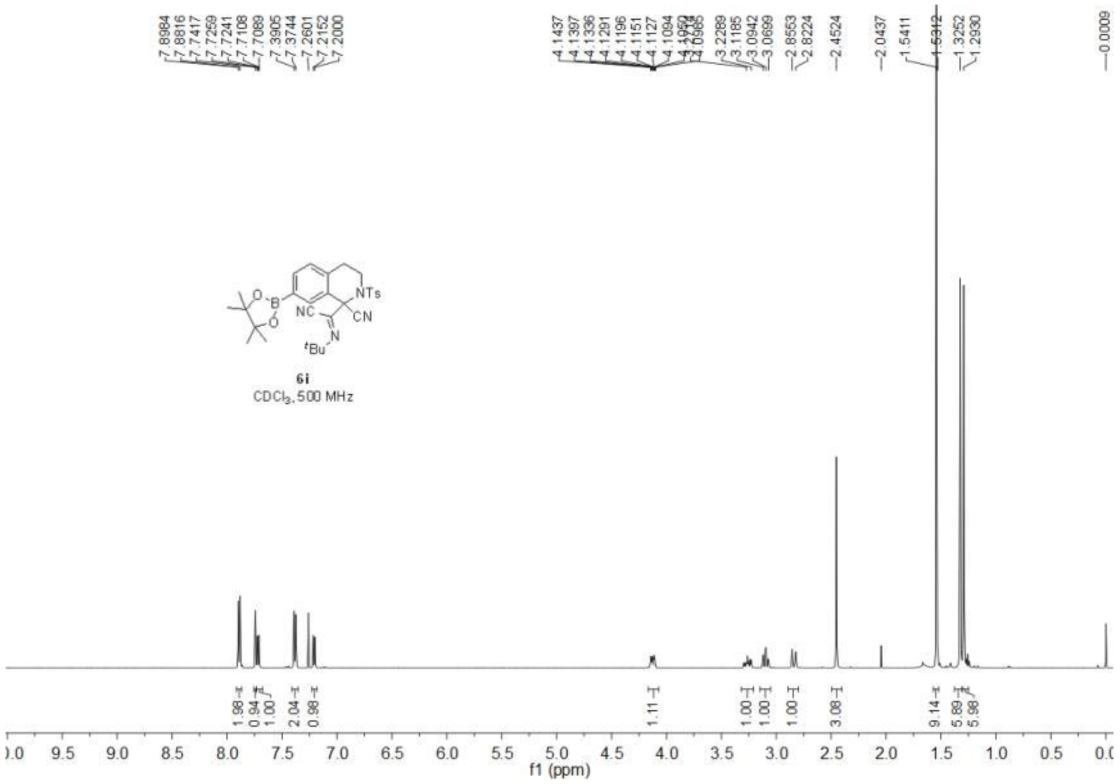

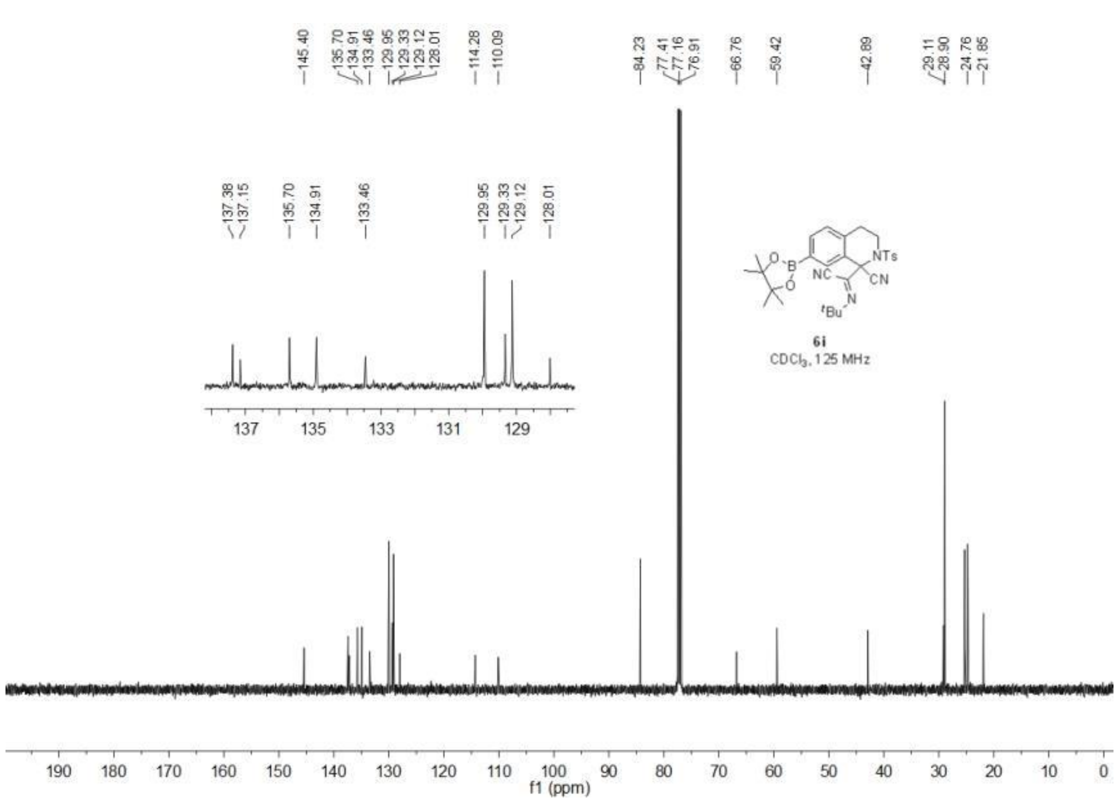

Figure S89. ^{1}H and ^{13}C NMR spectra of **6i.** Related to **Figure 4**.

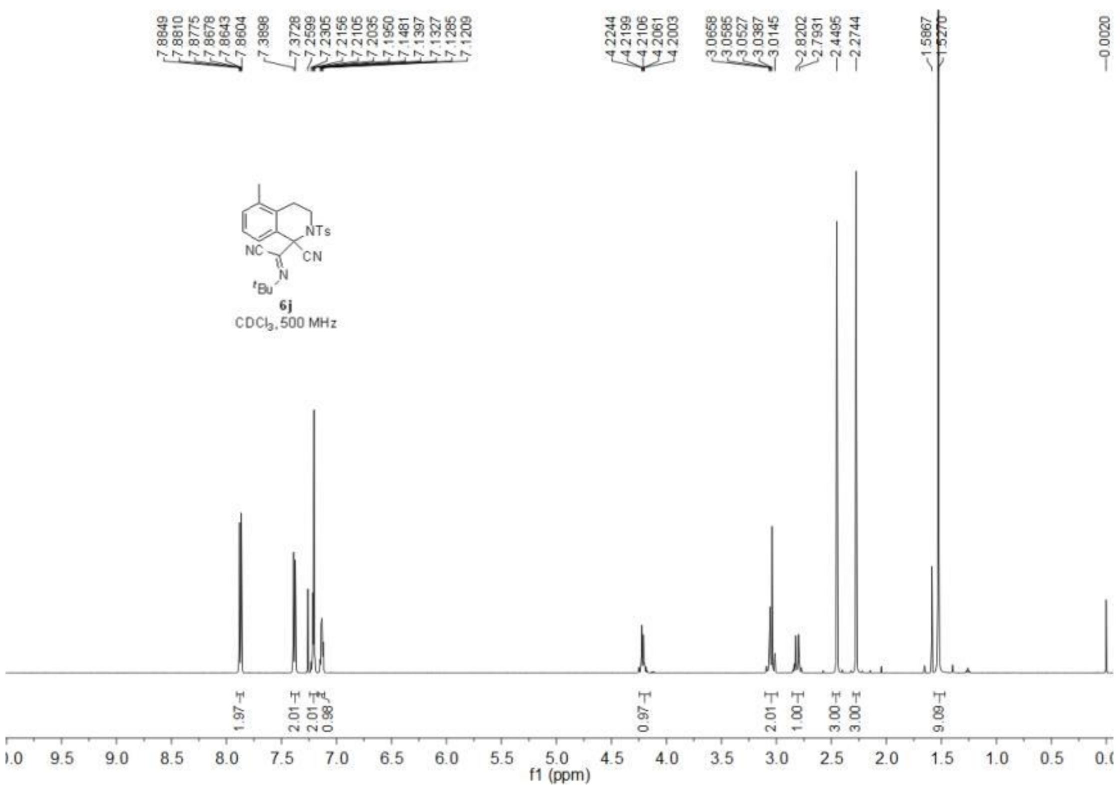

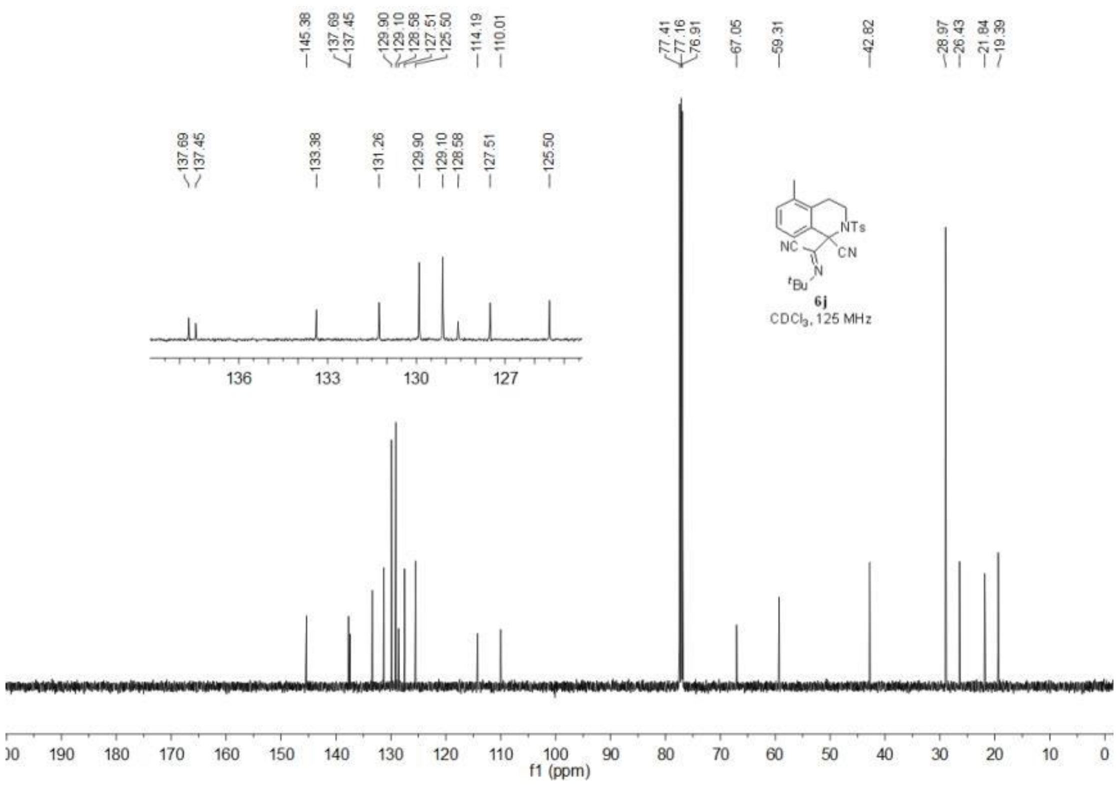

Figure S90. ^{1}H and ^{13}C NMR spectra of **6j**. Related to **Figure 4**.

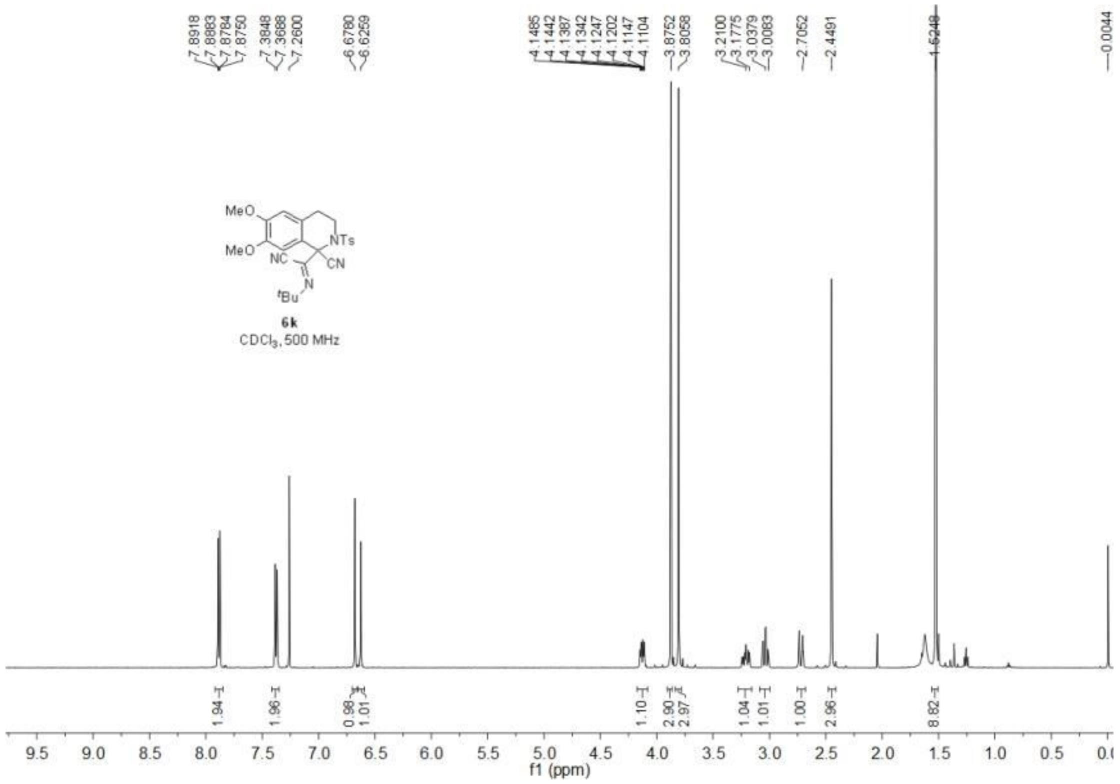

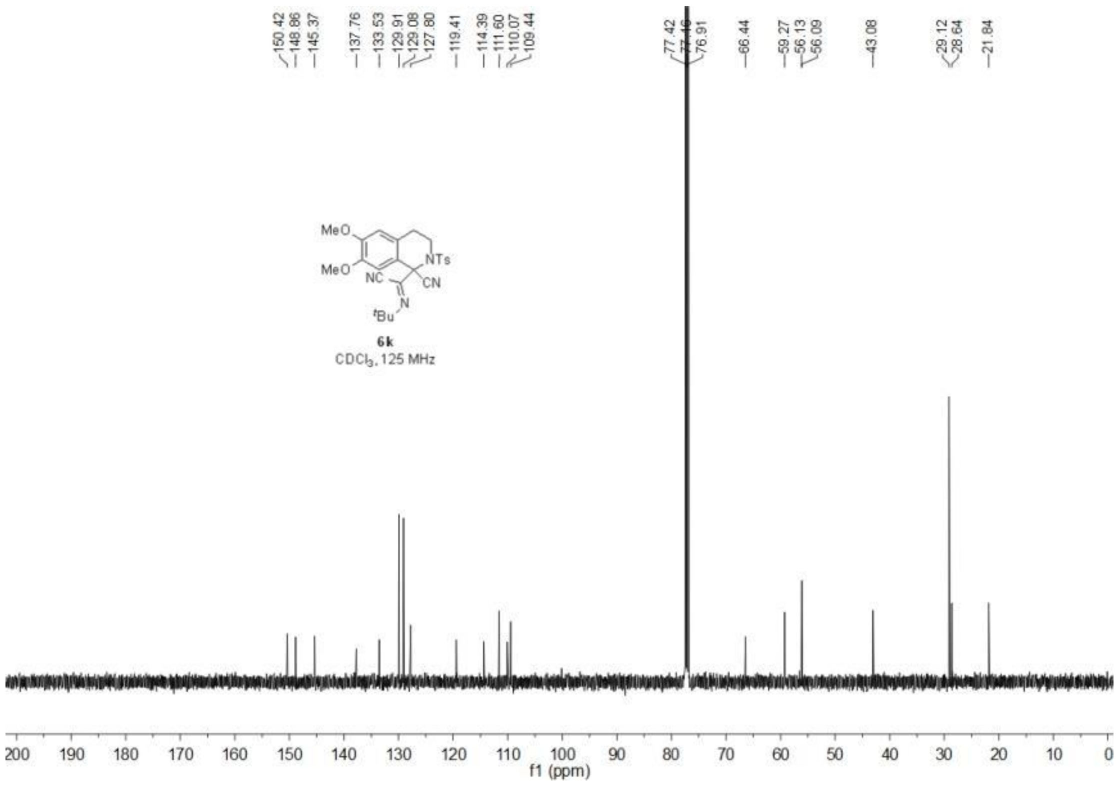

Figure S91. ^{1}H and ^{13}C NMR spectra of **6k**. Related to **Figure 4**.

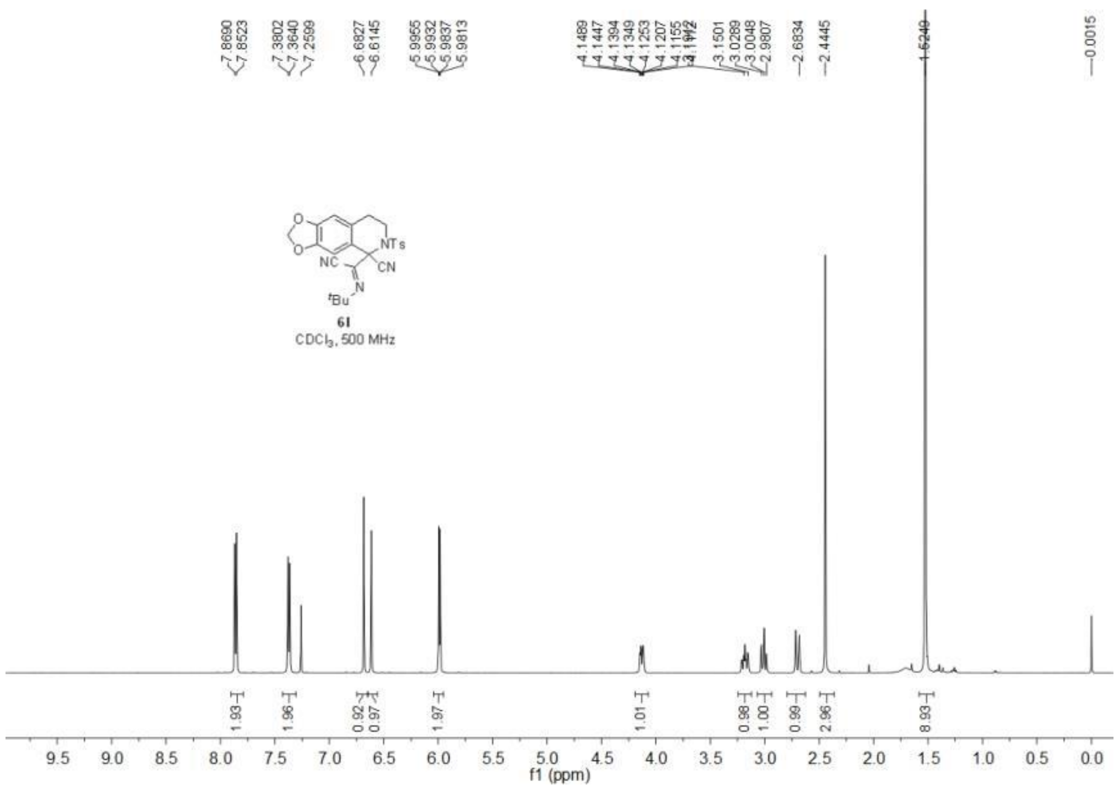

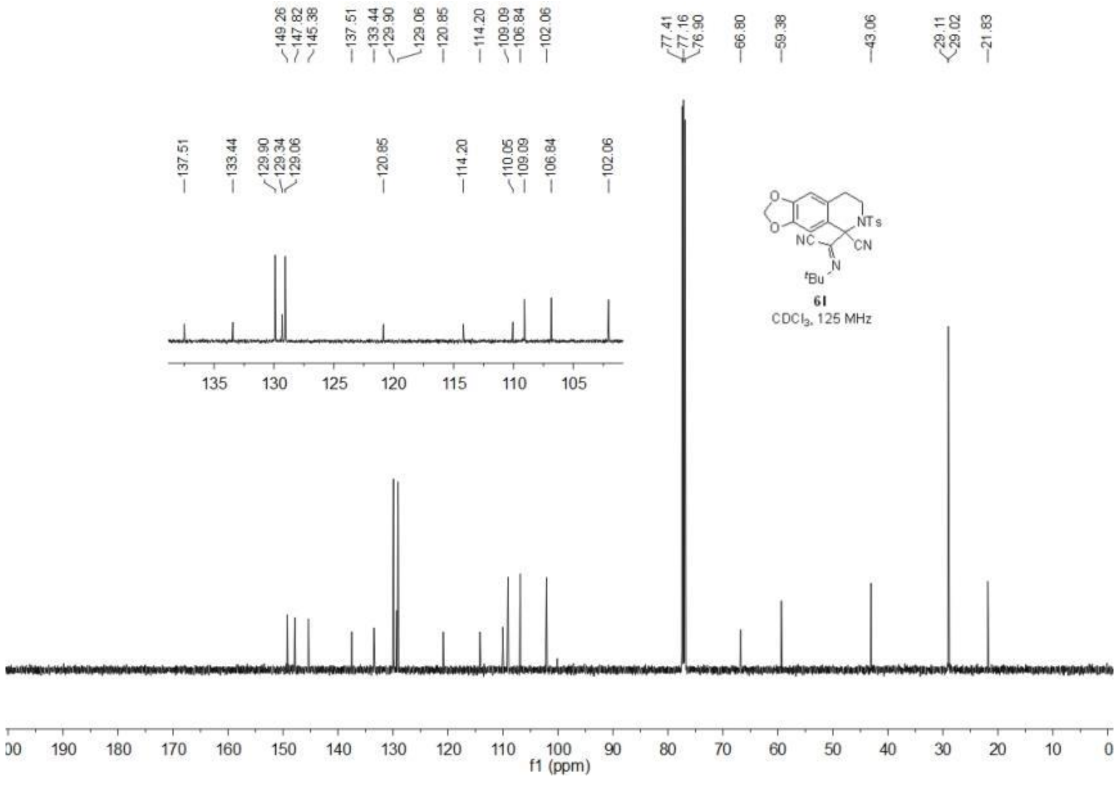

Figure S92. ^{1}H and ^{13}C NMR spectra of **6l.** Related to **Figure 4**.

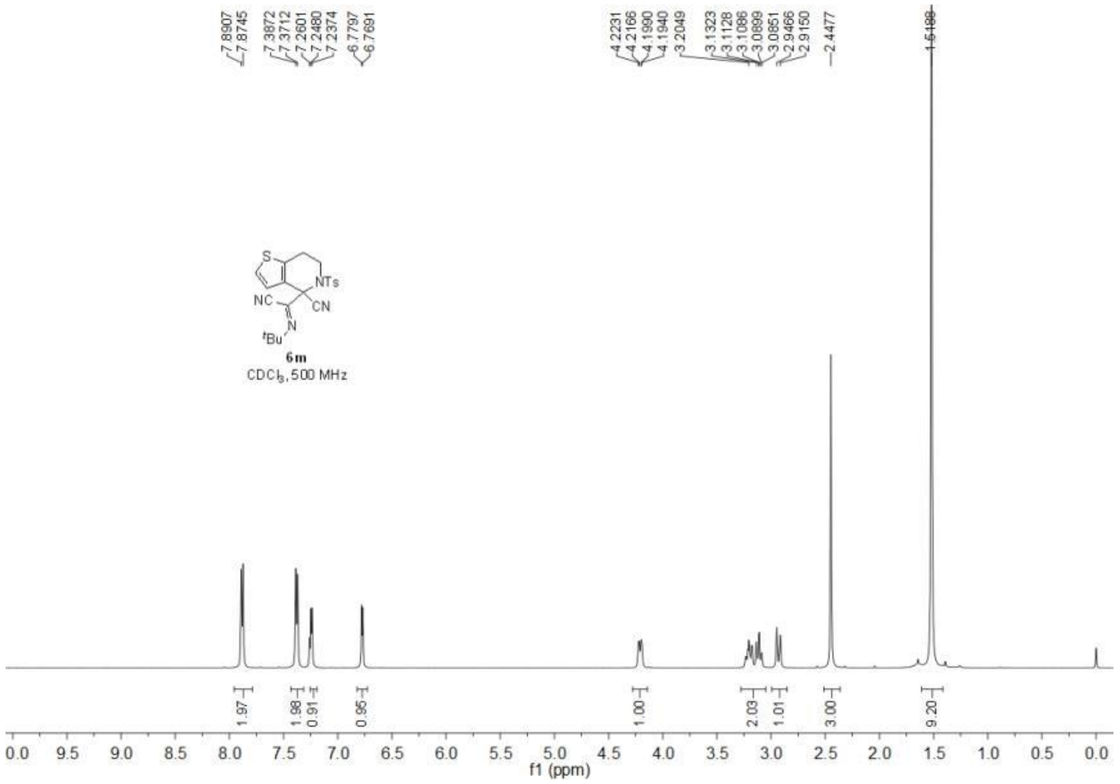

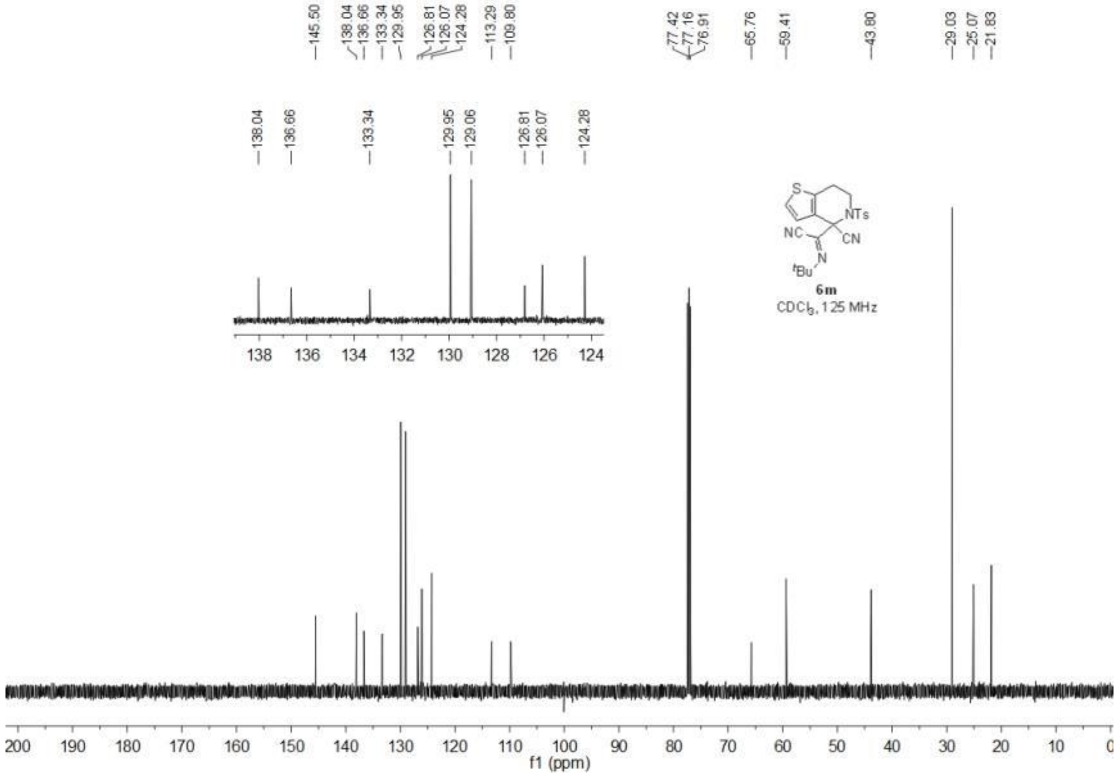

Figure S93. ^{1}H and ^{13}C NMR spectra of **6m**. Related to **Figure 4**.

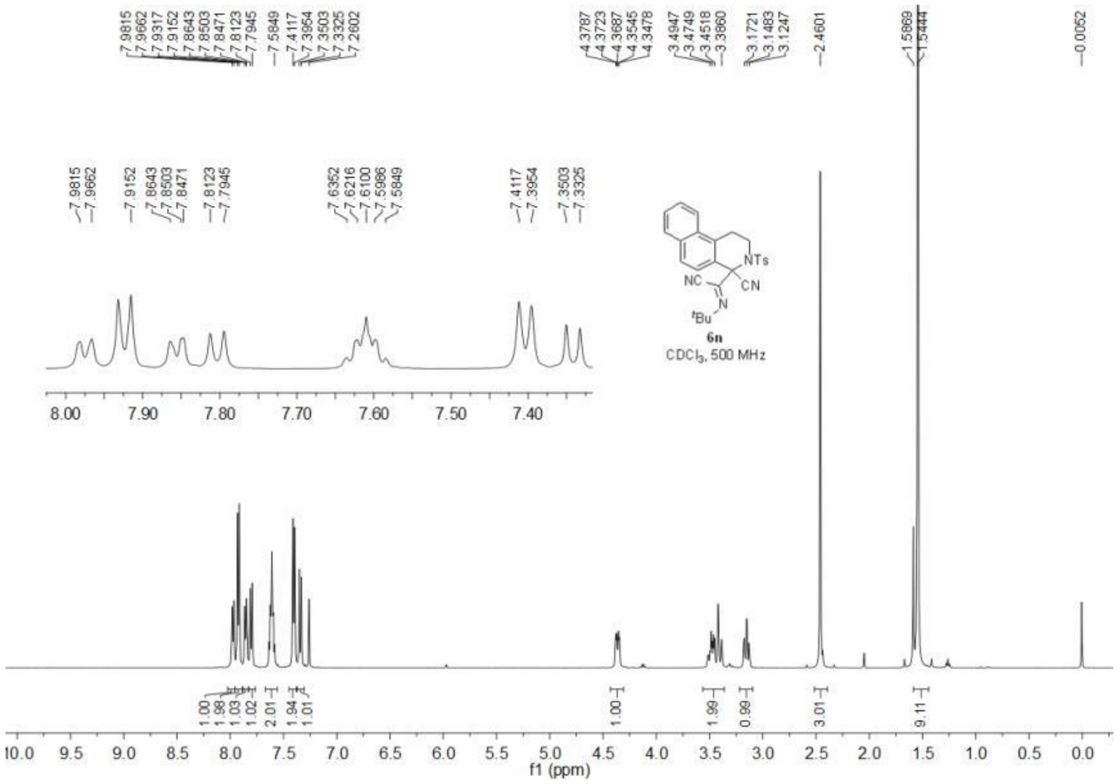

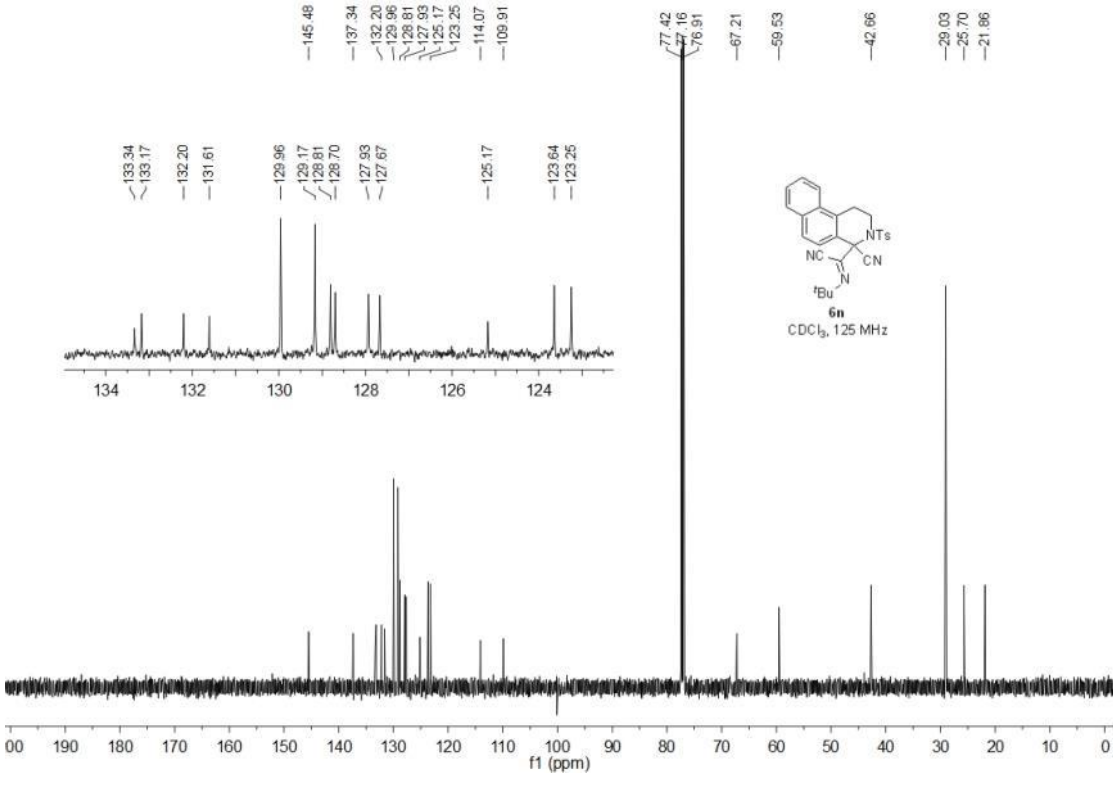

Figure S94. ^{1}H and ^{13}C NMR spectra of **6n**. Related to **Figure 4**.

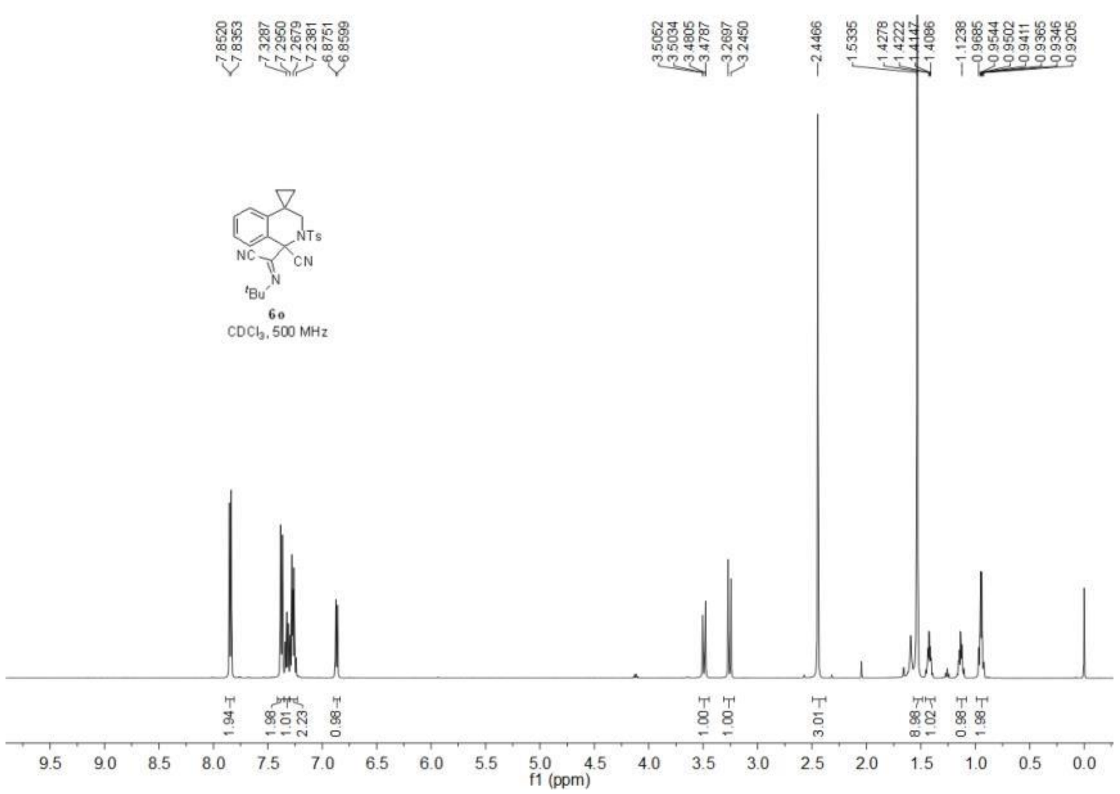

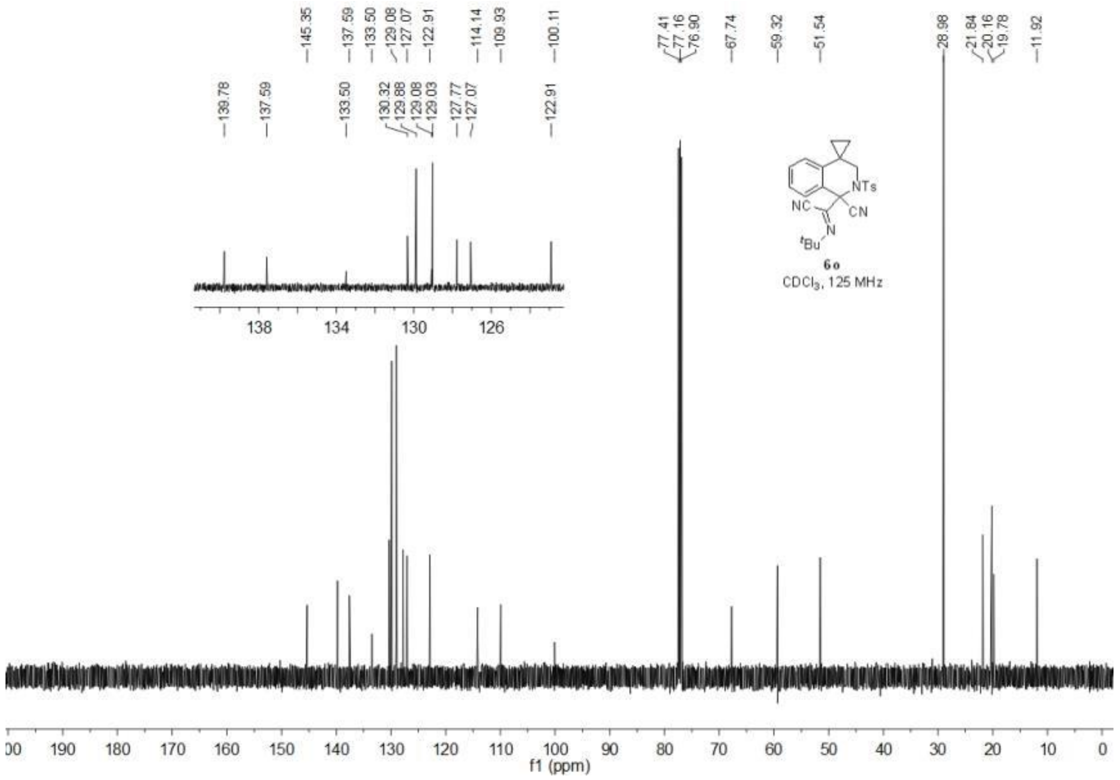

Figure S95. ^{1}H and ^{13}C NMR spectra of **6o**. Related to **Figure 4**.

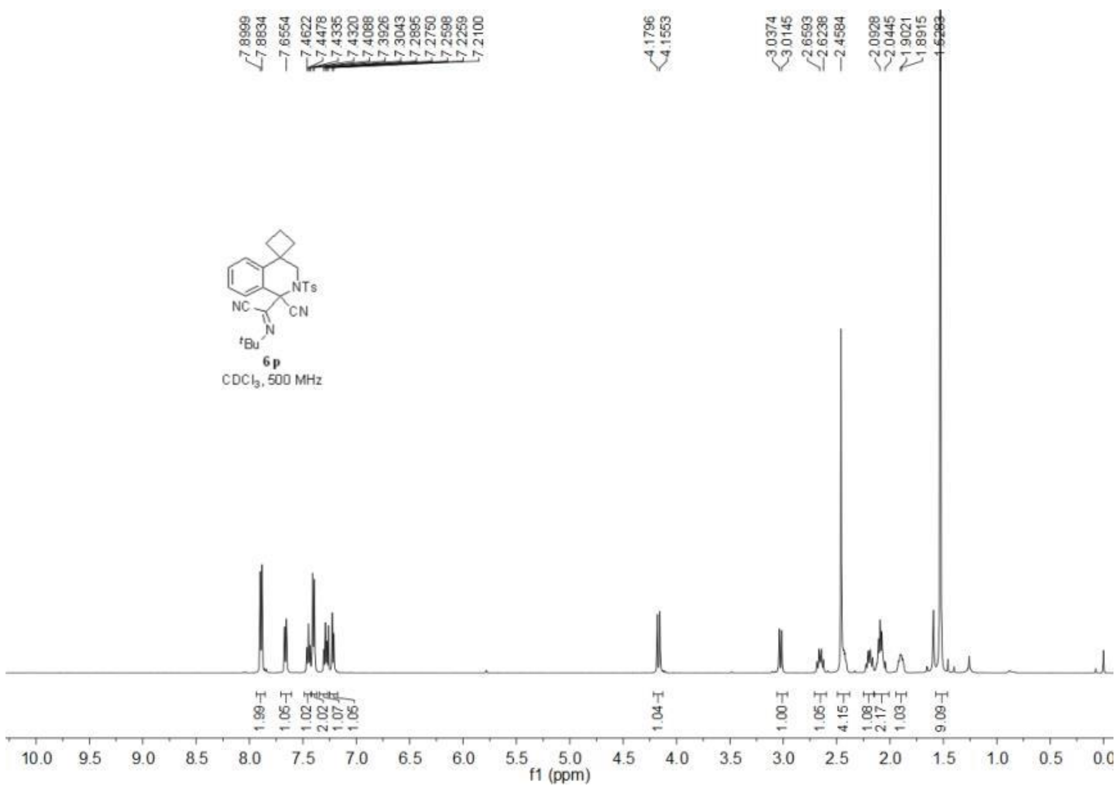

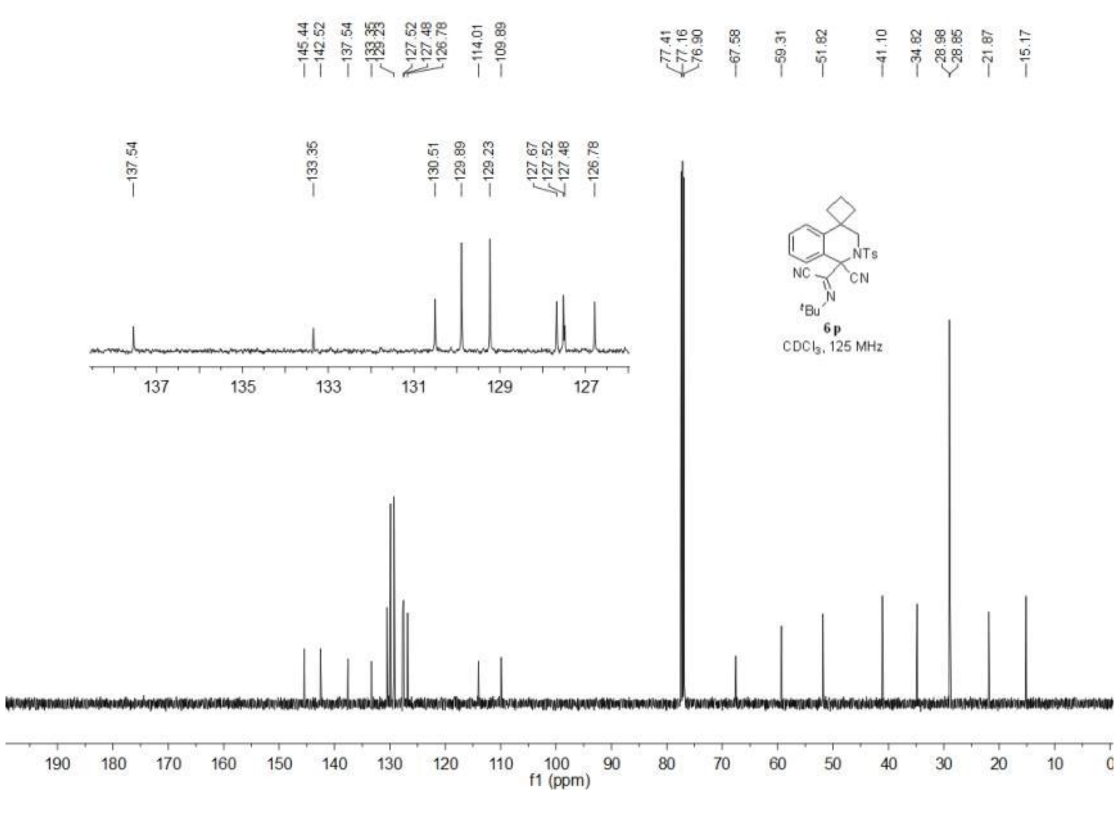

Figure S96. ^{1}H and ^{13}C NMR spectra of **6p.** Related to **Figure 4**.

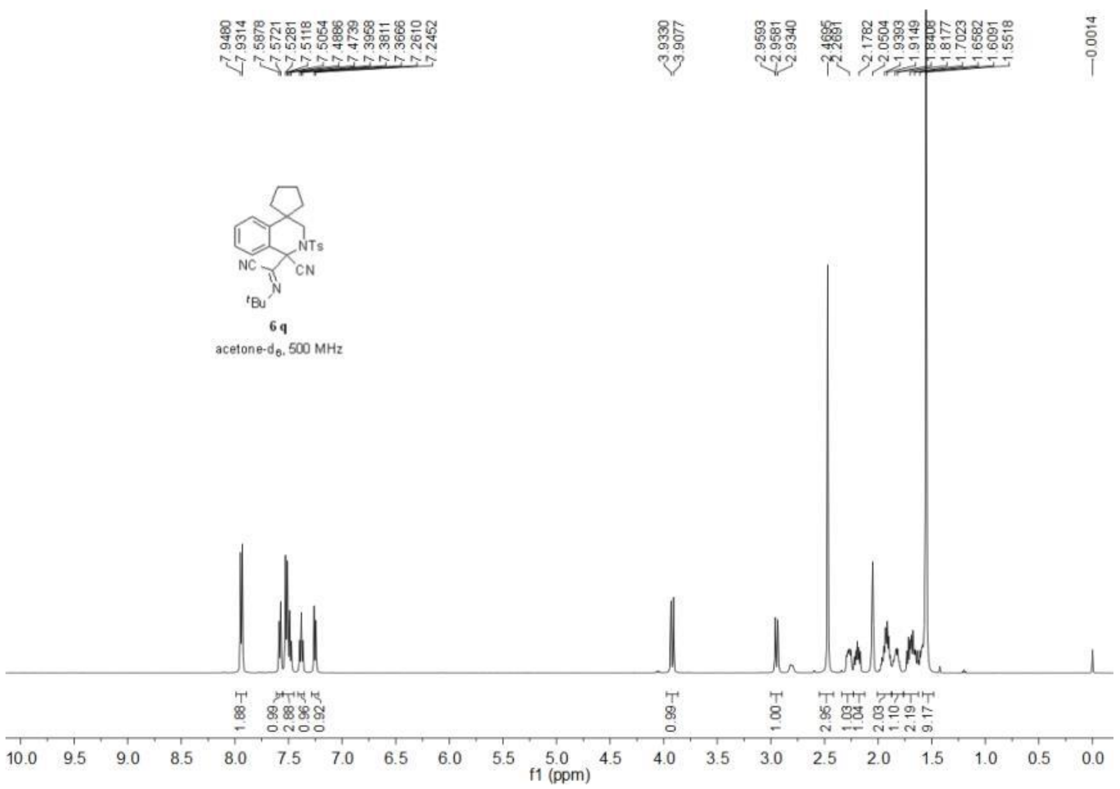

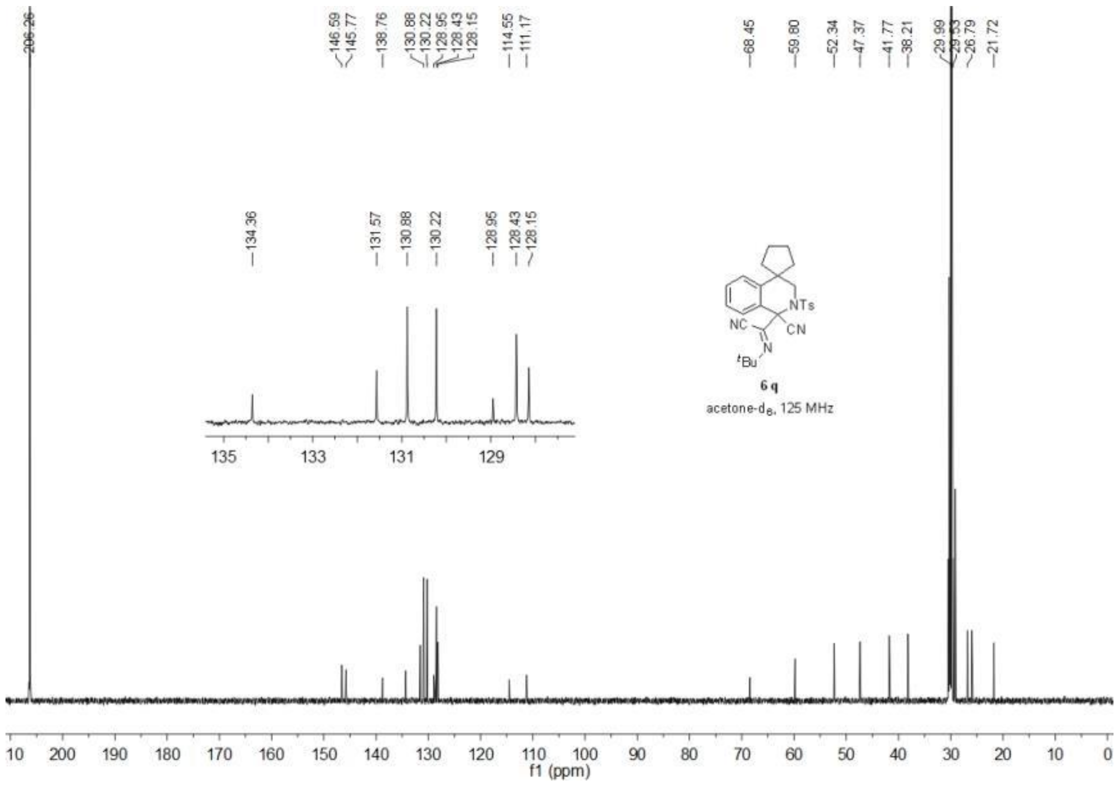

Figure S97. ^{1}H and ^{13}C NMR spectra of **6q**. Related to **Figure 4**.

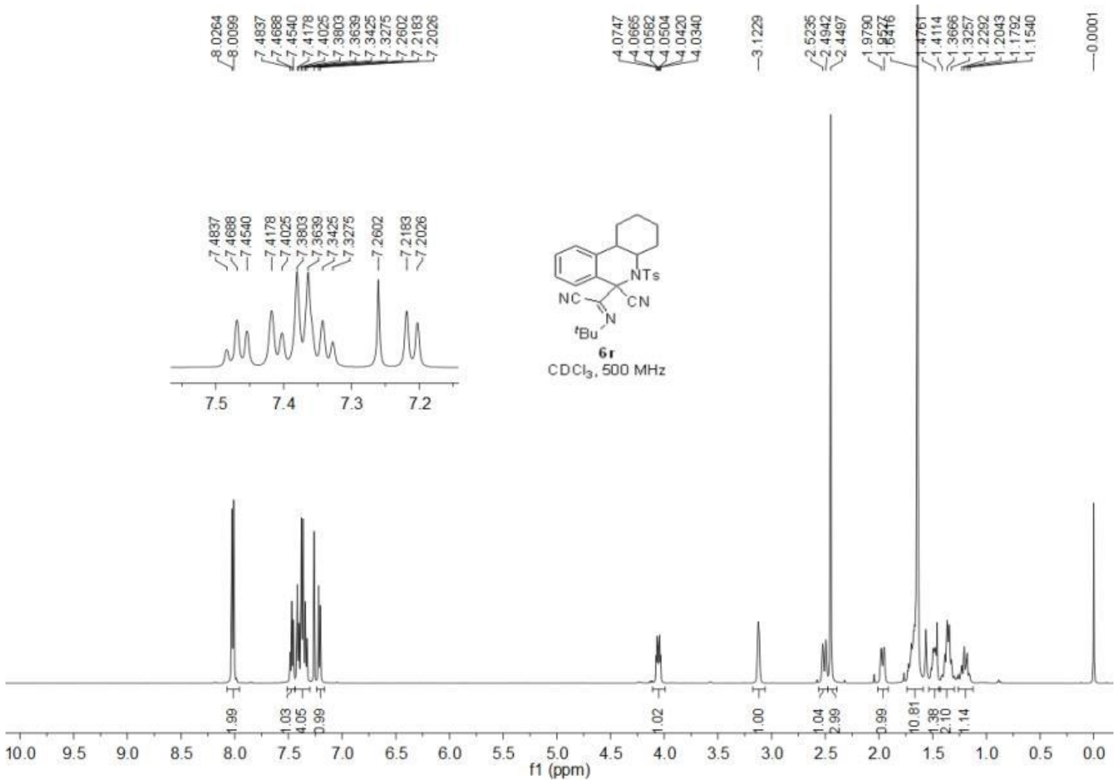

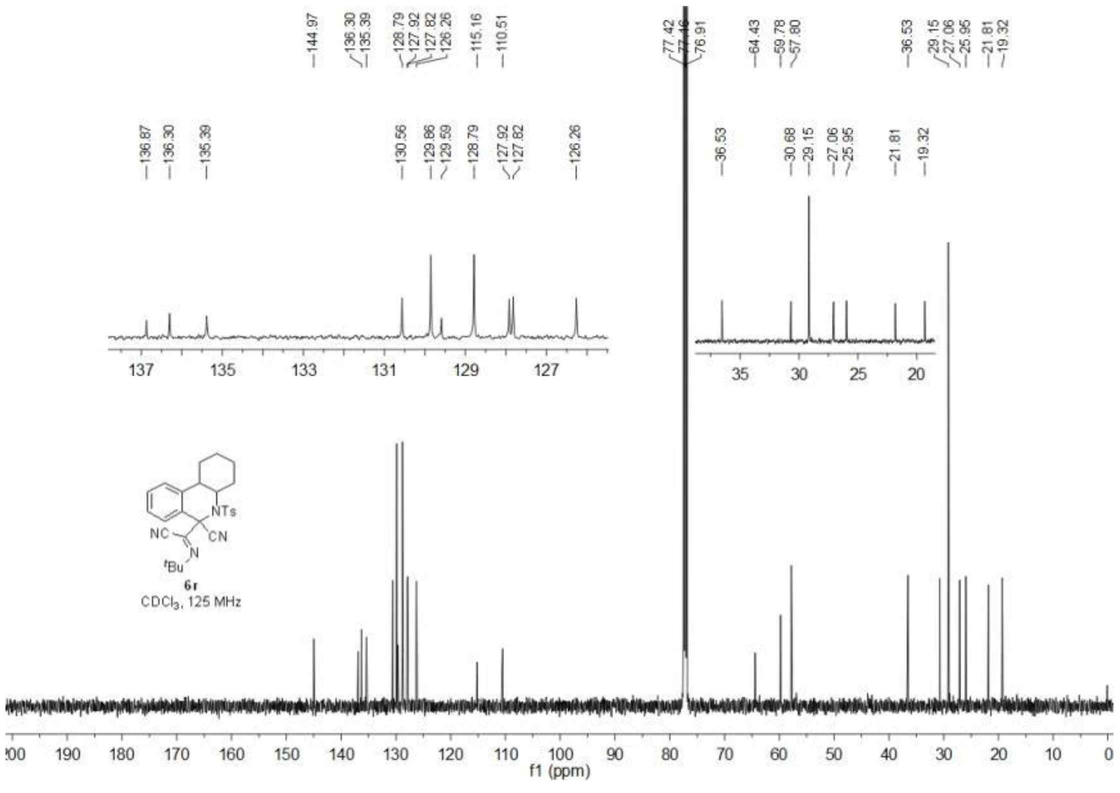

Figure S98. ^{1}H and ^{13}C NMR spectra of **6r**. Related to **Figure 4**.

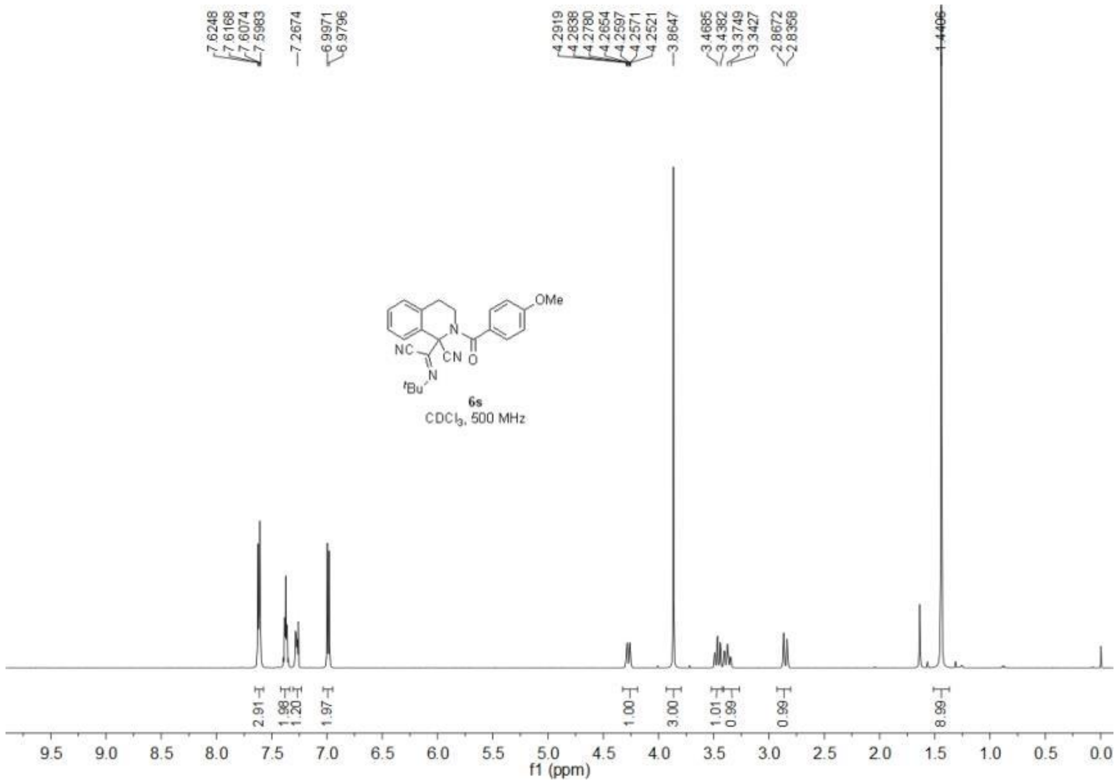

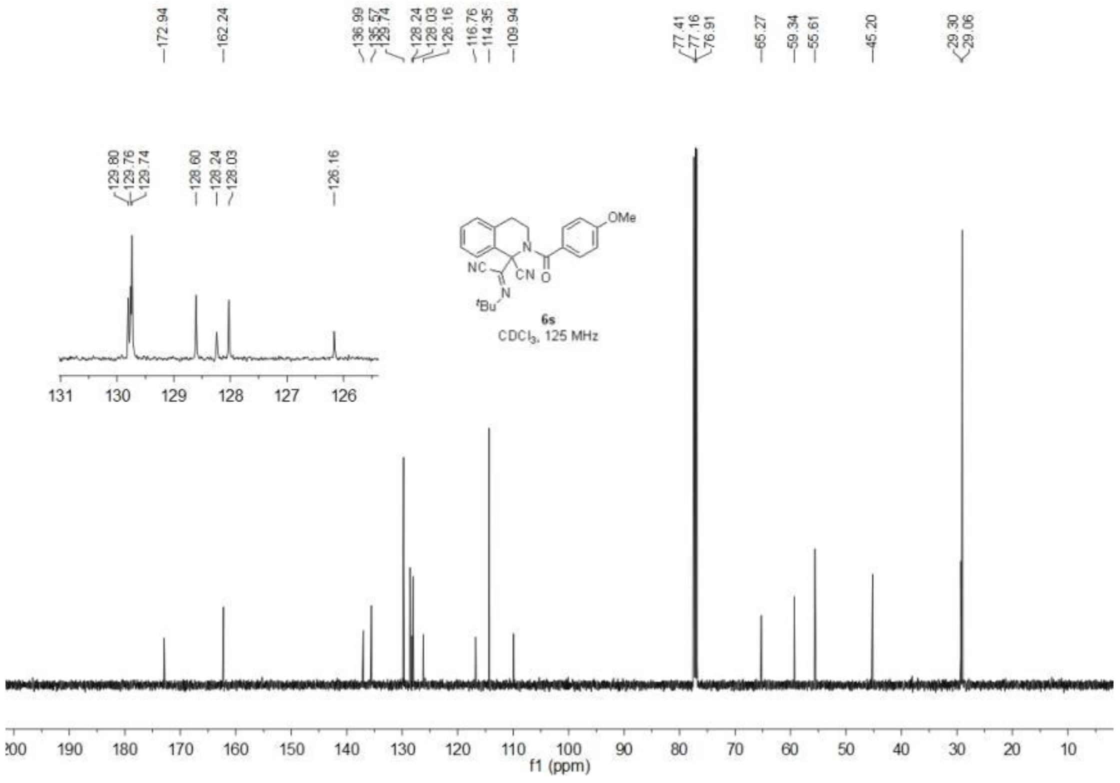

Figure S99. ^{1}H and ^{13}C NMR spectra of **6s**. Related to **Figure 4**.

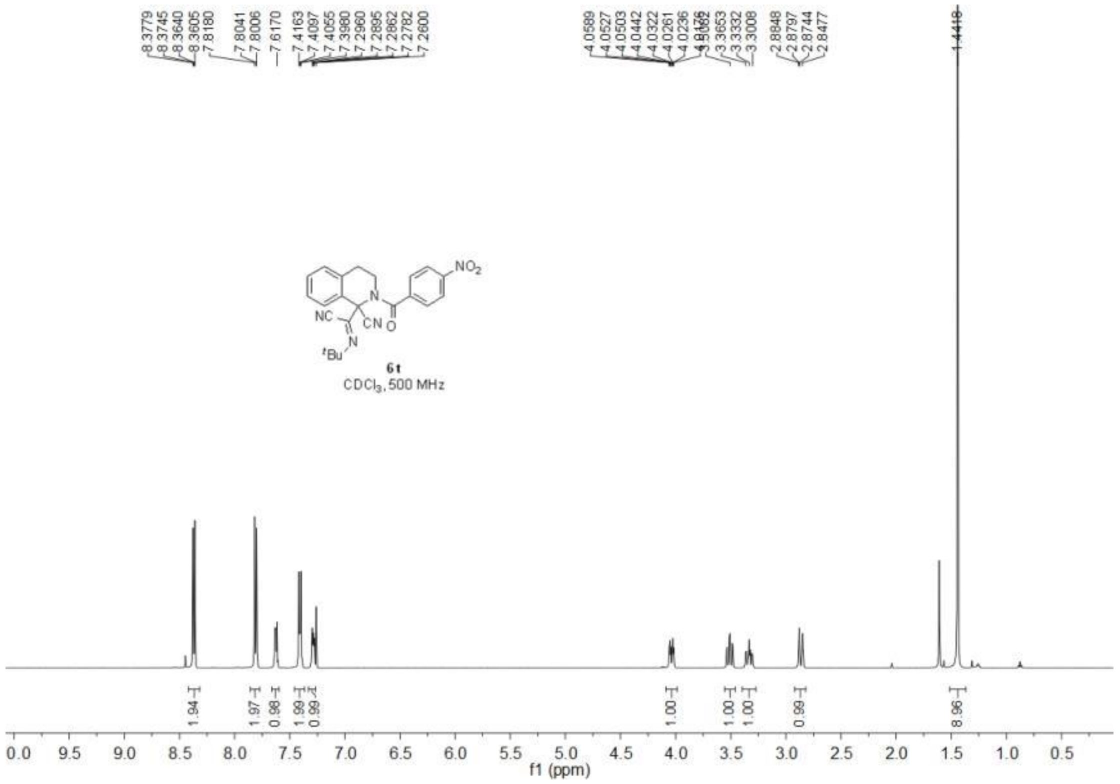

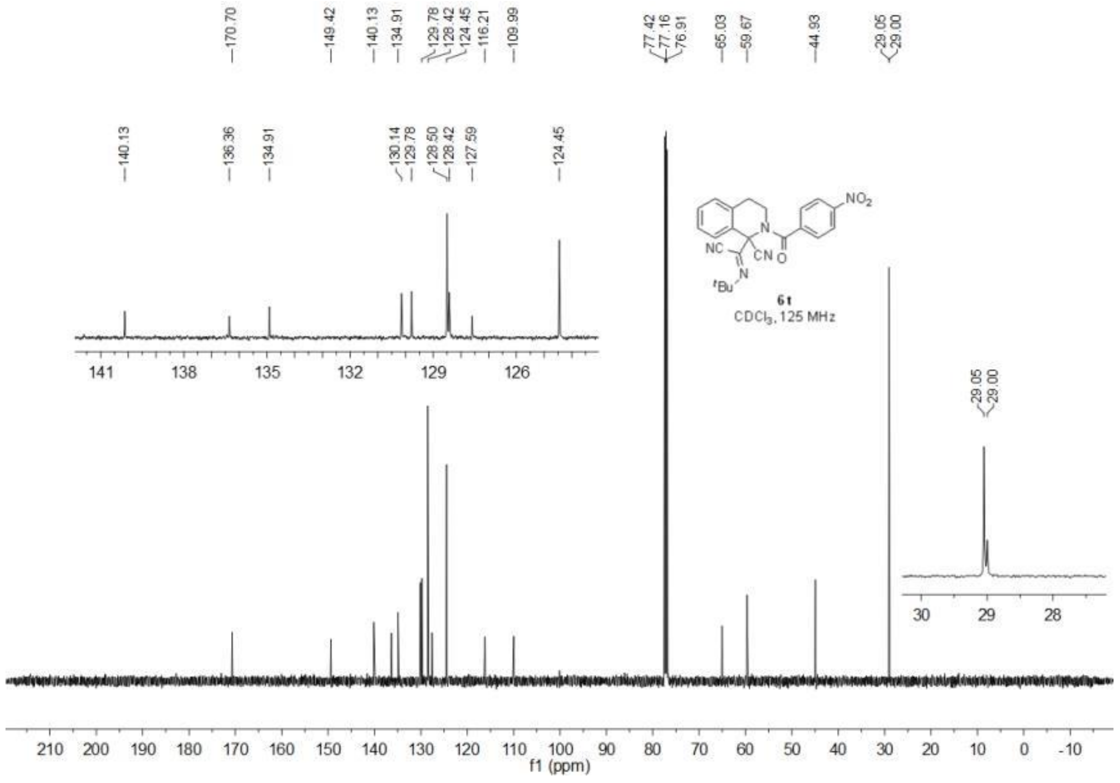

Figure S100. ¹H and ¹³C NMR spectra of **6t.** Related to **Figure 4**.

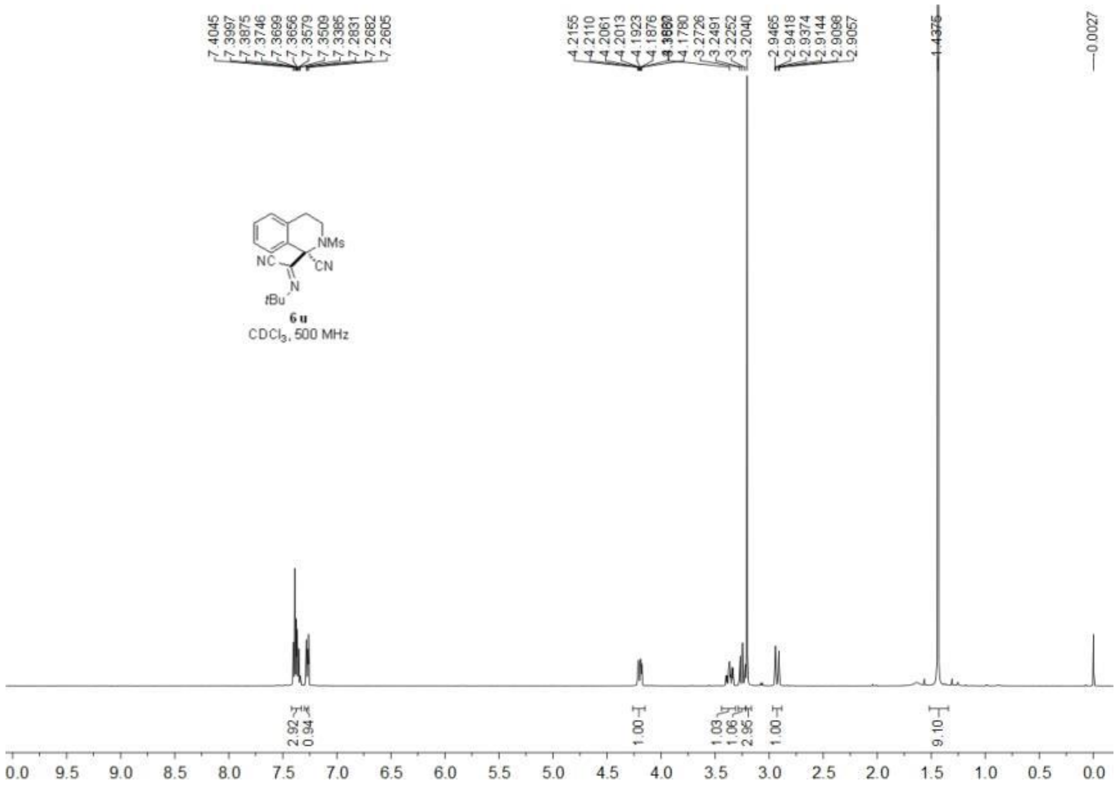

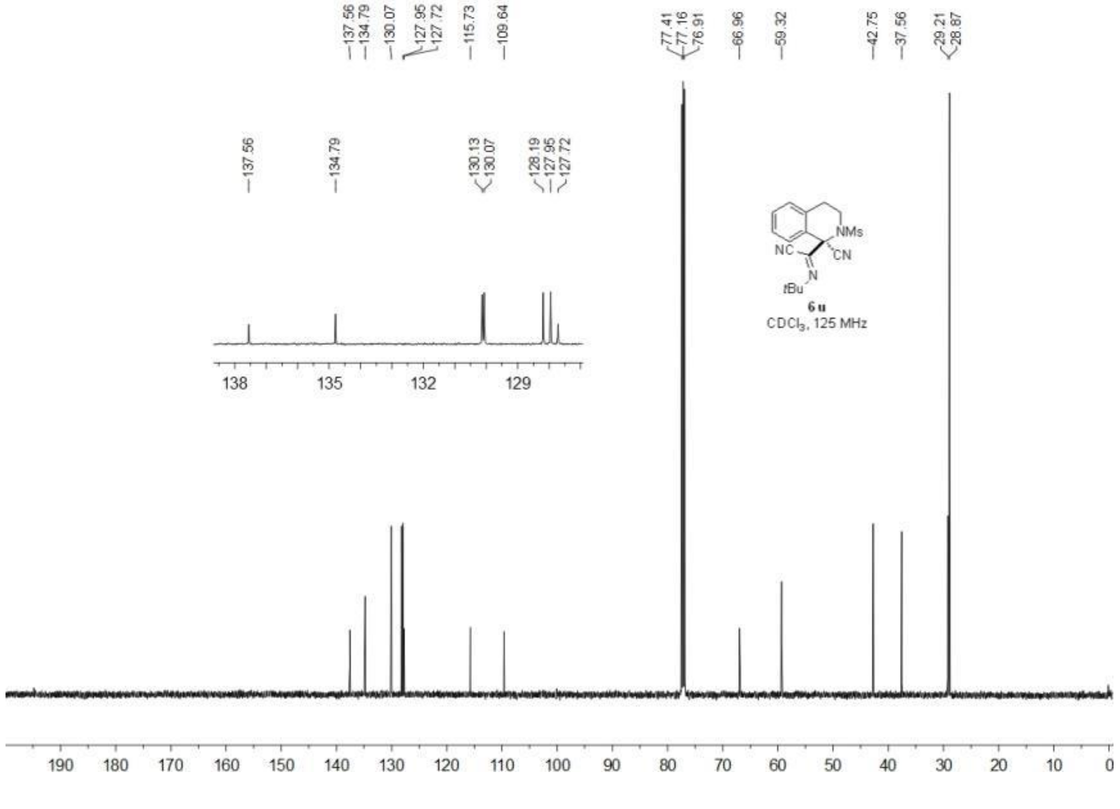

Figure S101. ^{1}H and ^{13}C NMR spectra of **6u**. Related to **Figure 4**.

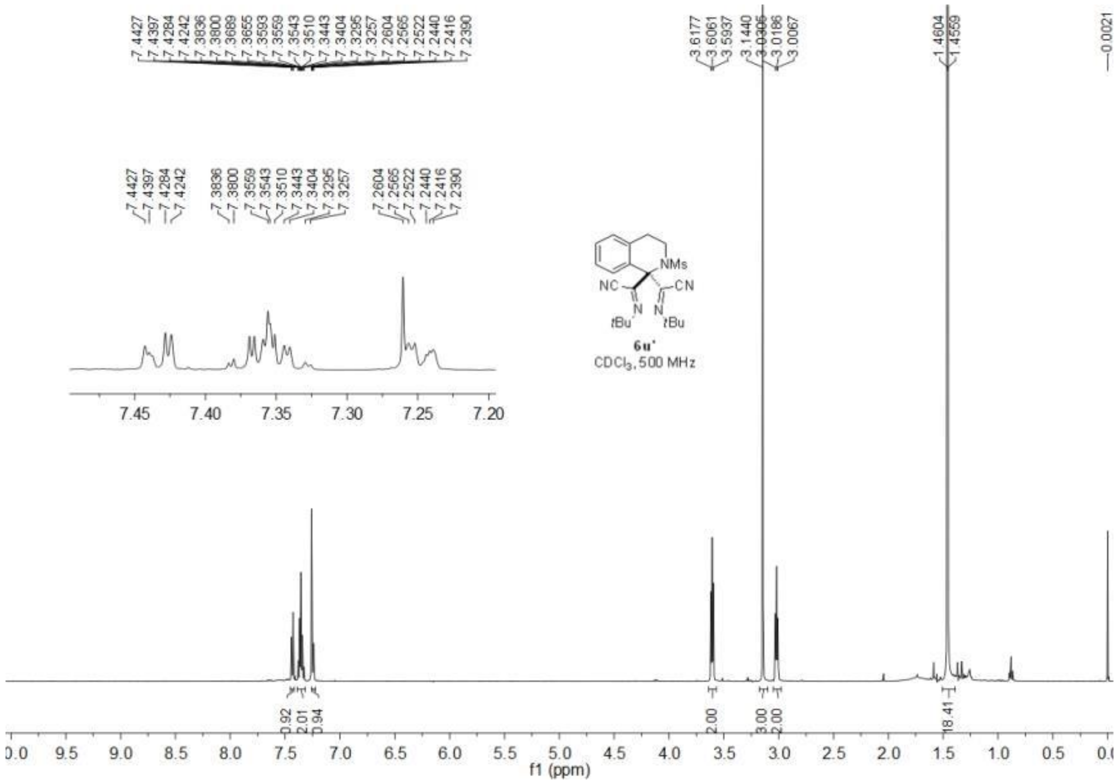

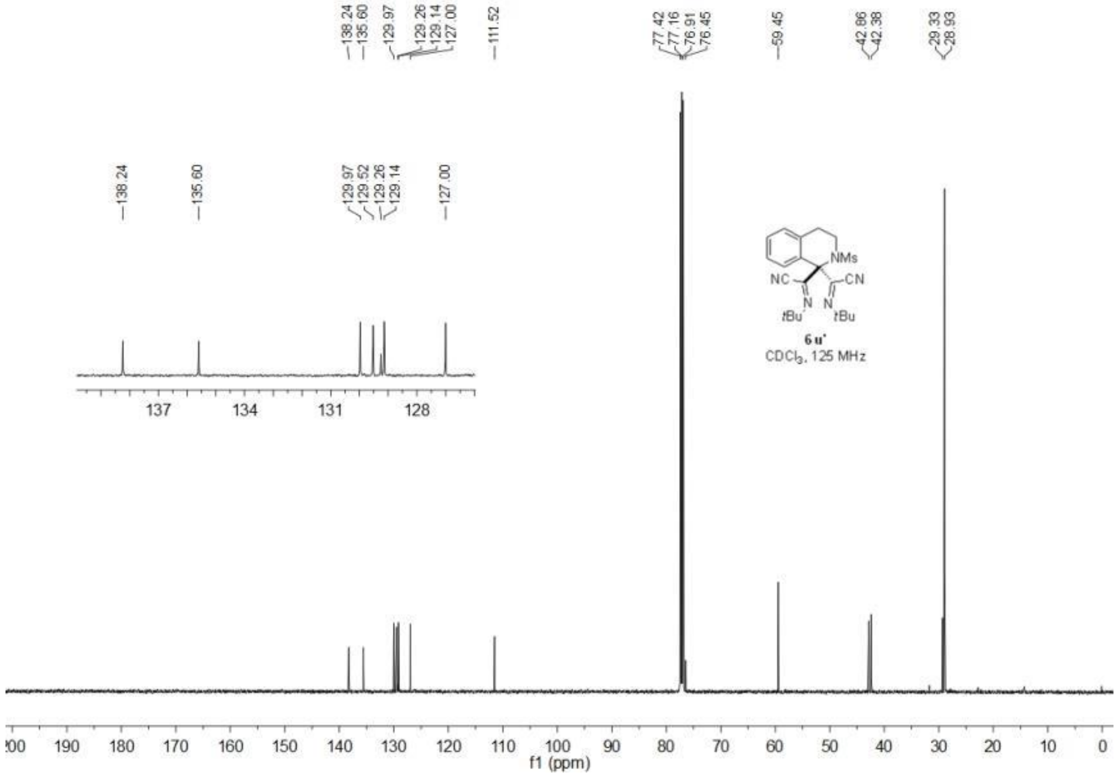

Figure S102. ^{1}H and ^{13}C NMR spectra of **6u'.** Related to **Figure 4**.

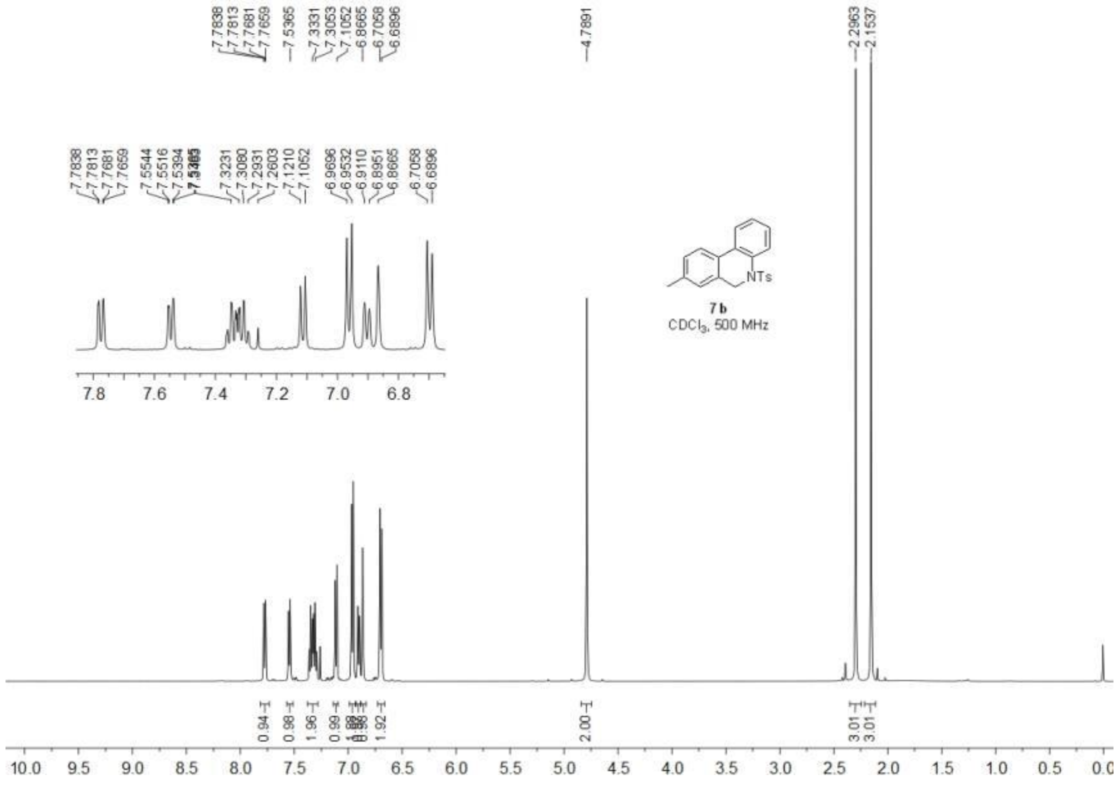

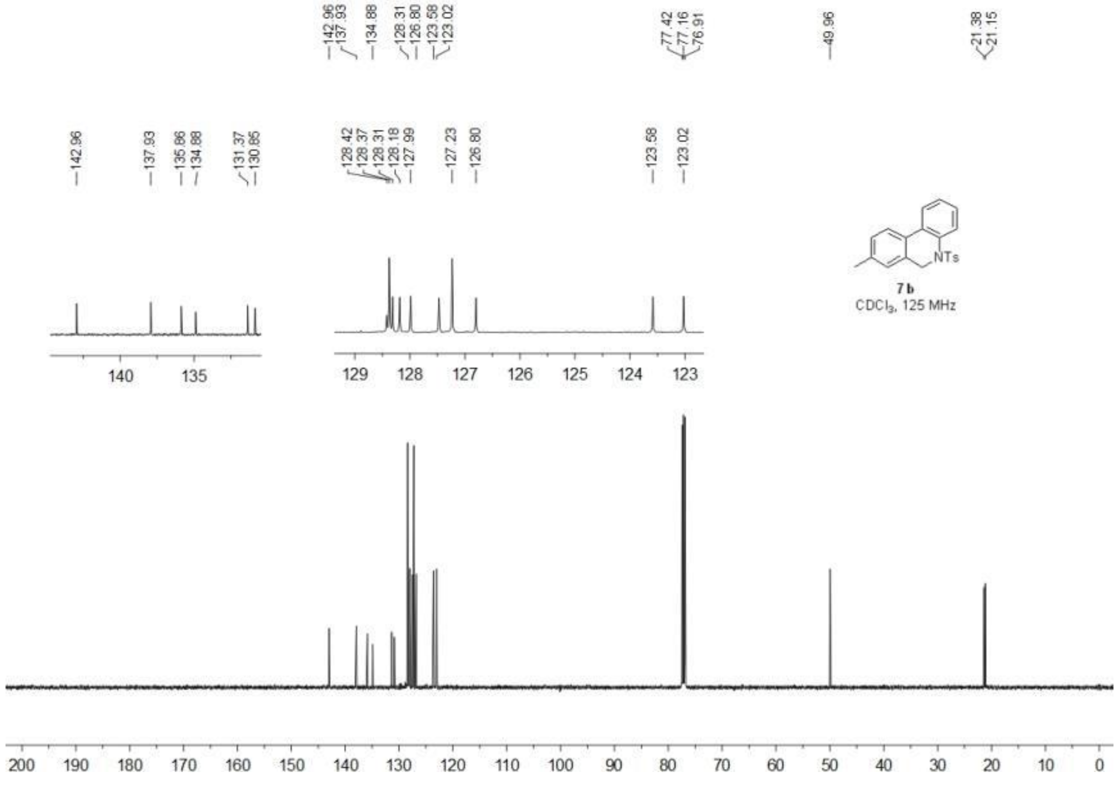

Figure S103. ^{1}H and ^{13}C NMR spectra of **7b**. Related to **Figure 5**.

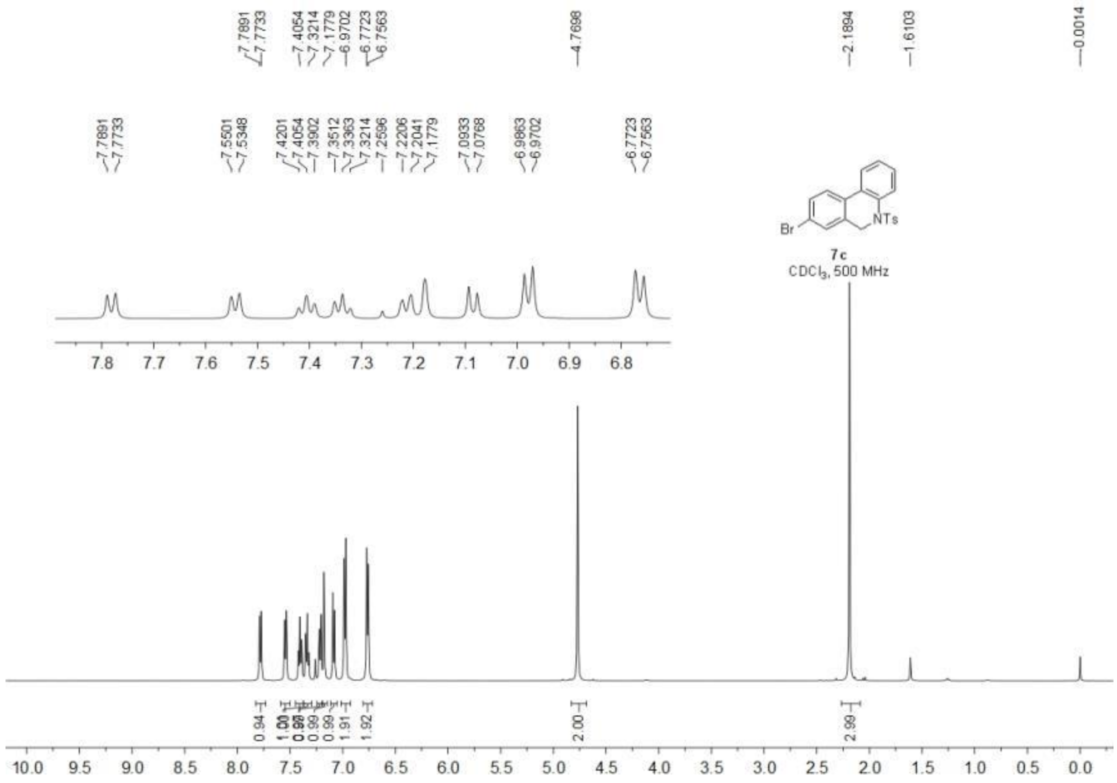

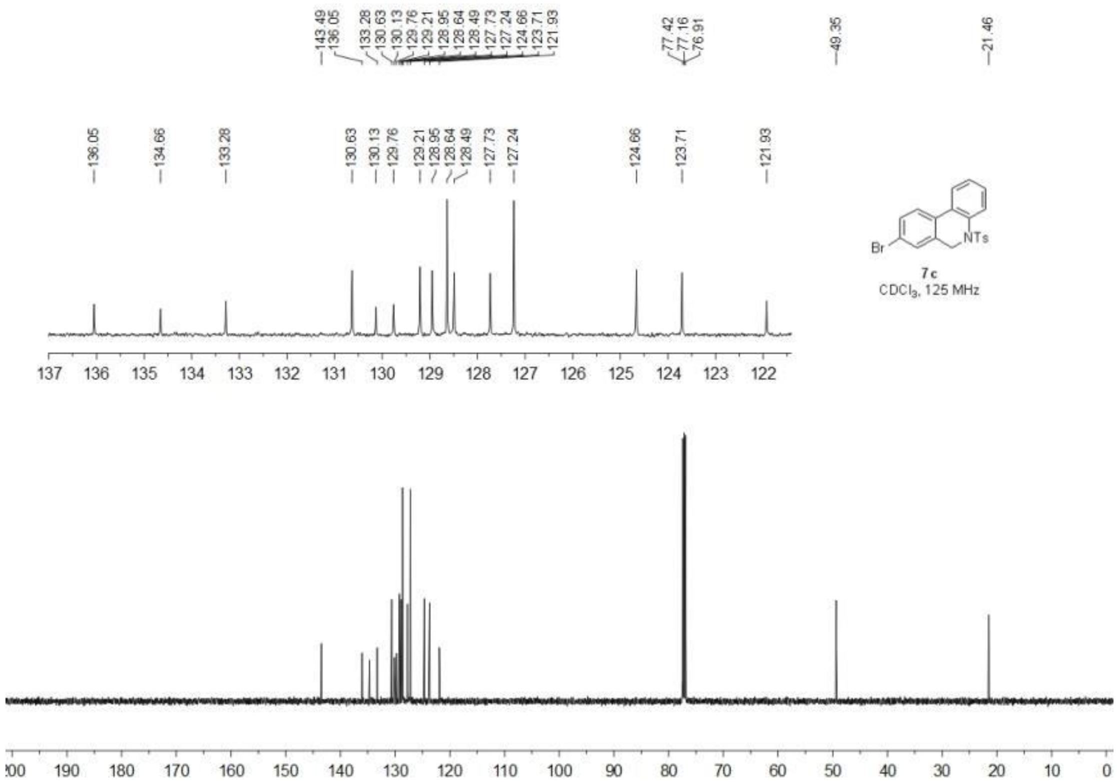

Figure S104. ^{1}H and ^{13}C NMR spectra of **7c**. Related to **Figure 5**.

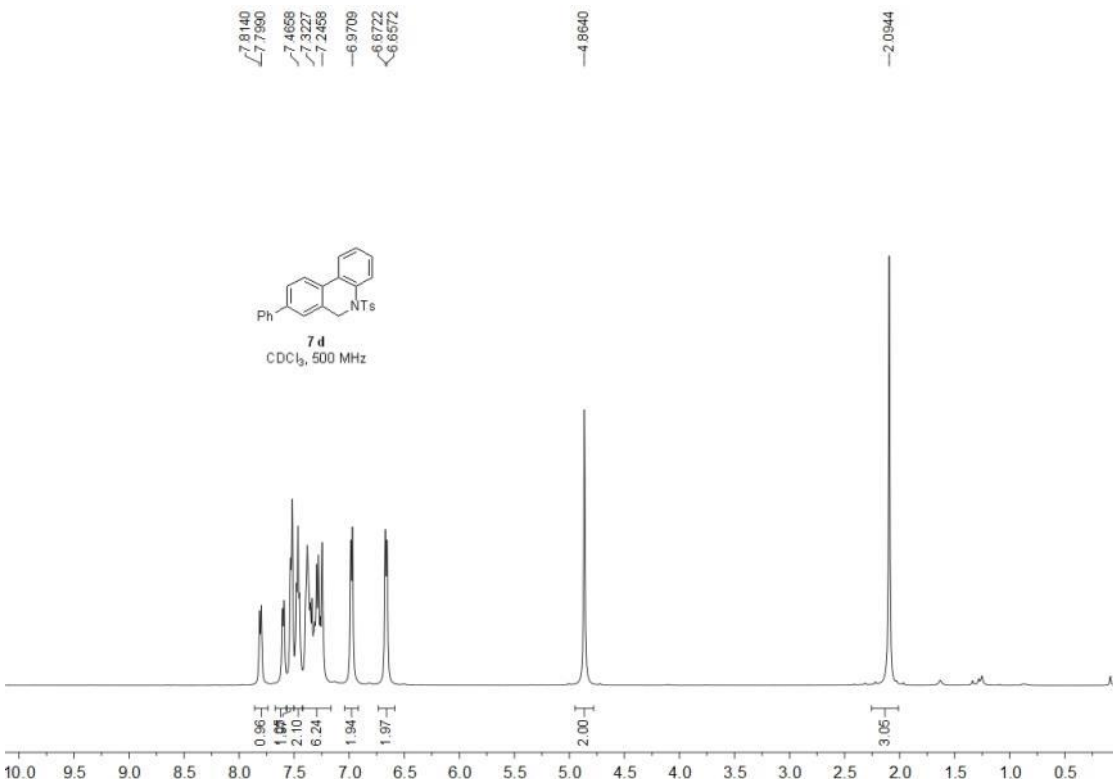

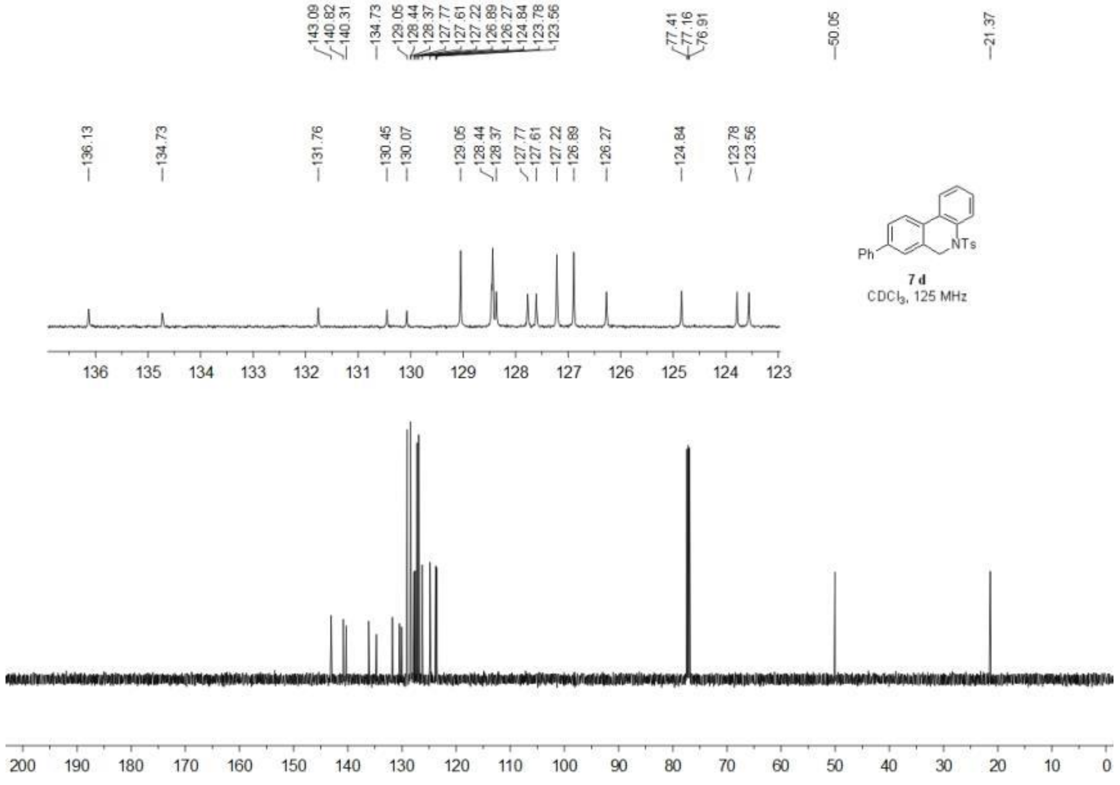

Figure S105. ^{1}H and ^{13}C NMR spectra of **7d**. Related to **Figure 5**.

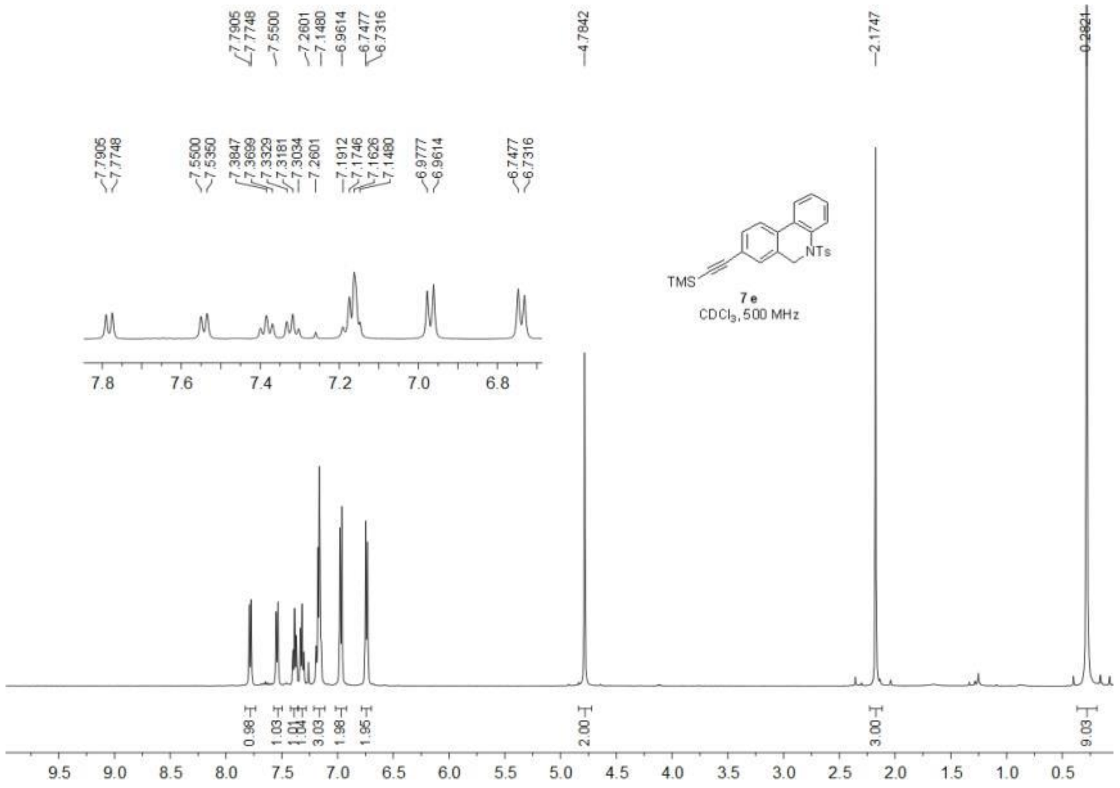

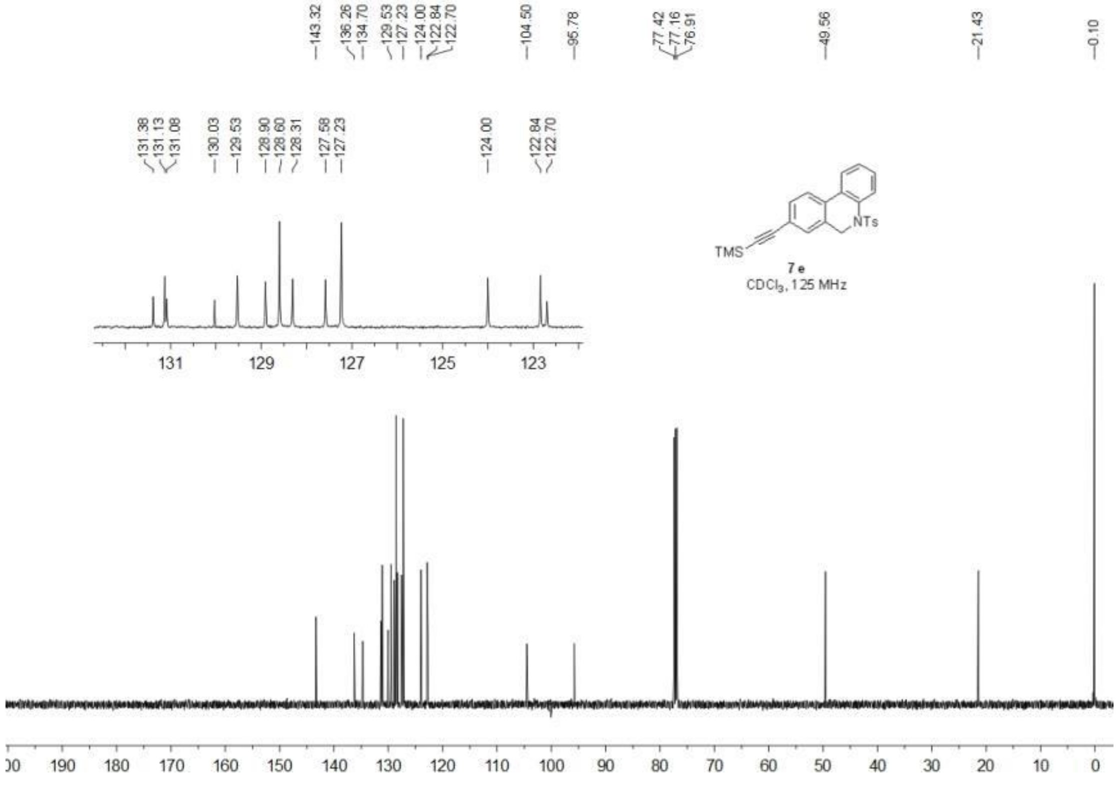

Figure S106. ^{1}H and ^{13}C NMR spectra of **7e**. Related to **Figure 5**.

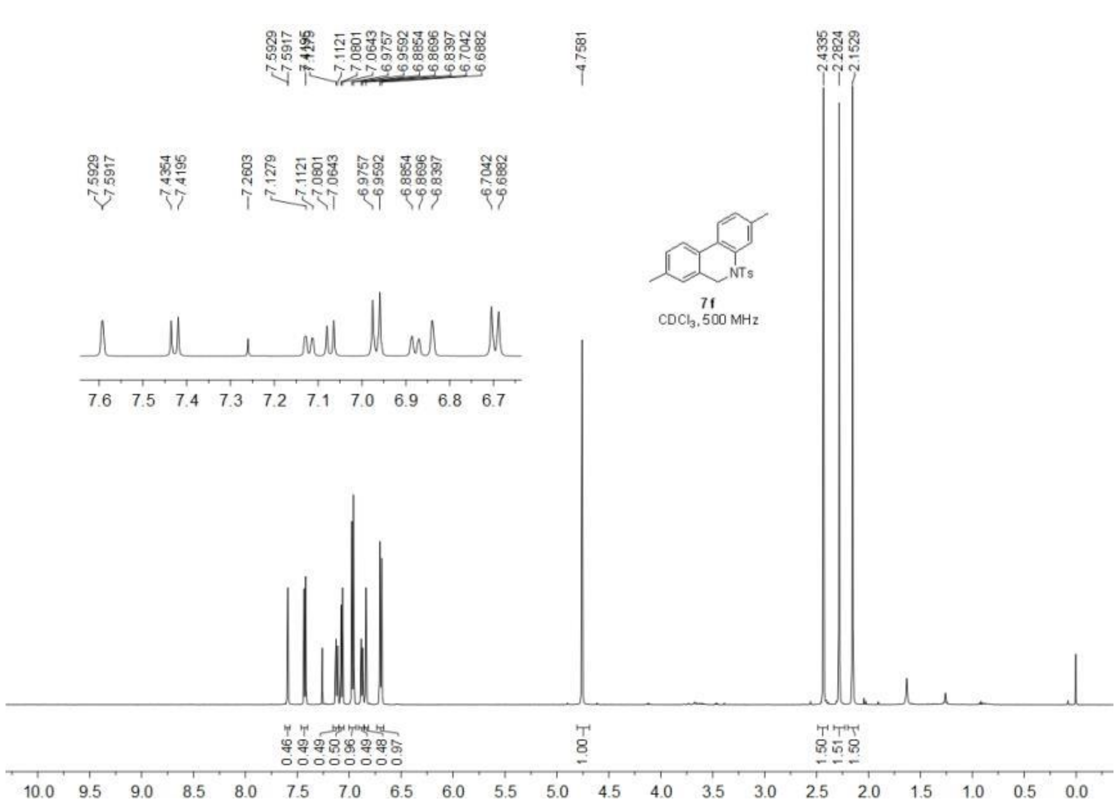

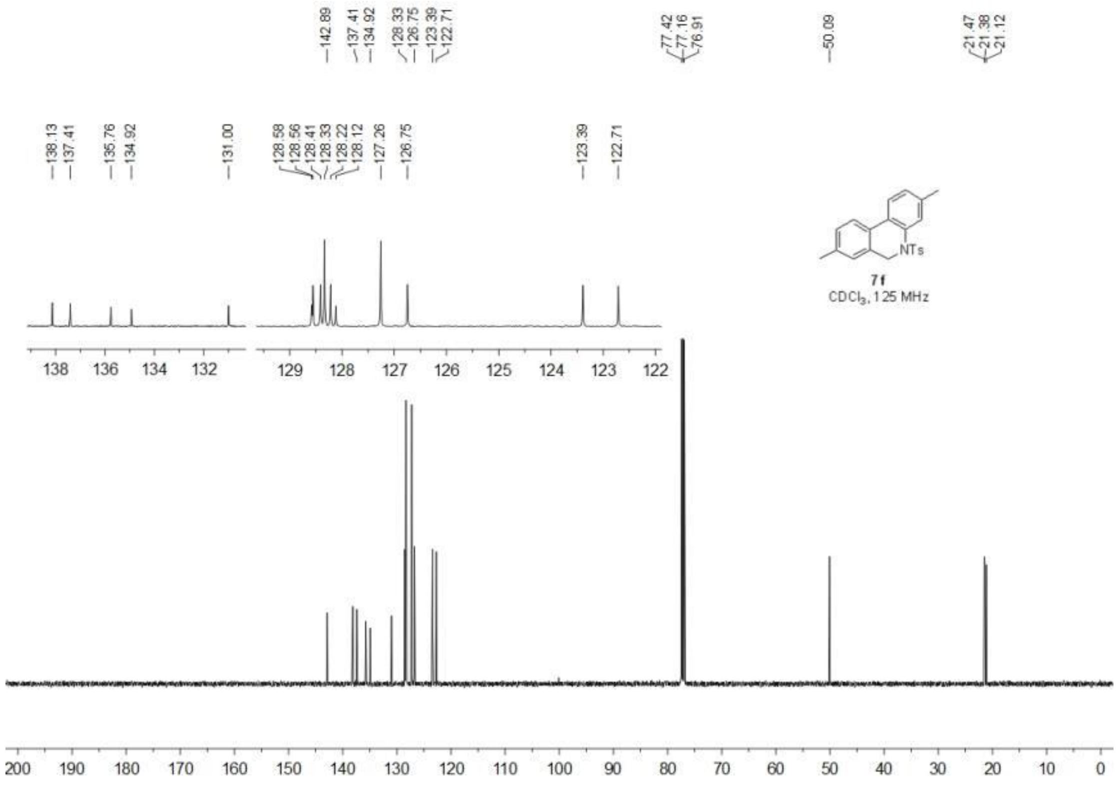

Figure S107. ^{1}H and ^{13}C NMR spectra of **7f.** Related to **Figure 5**.

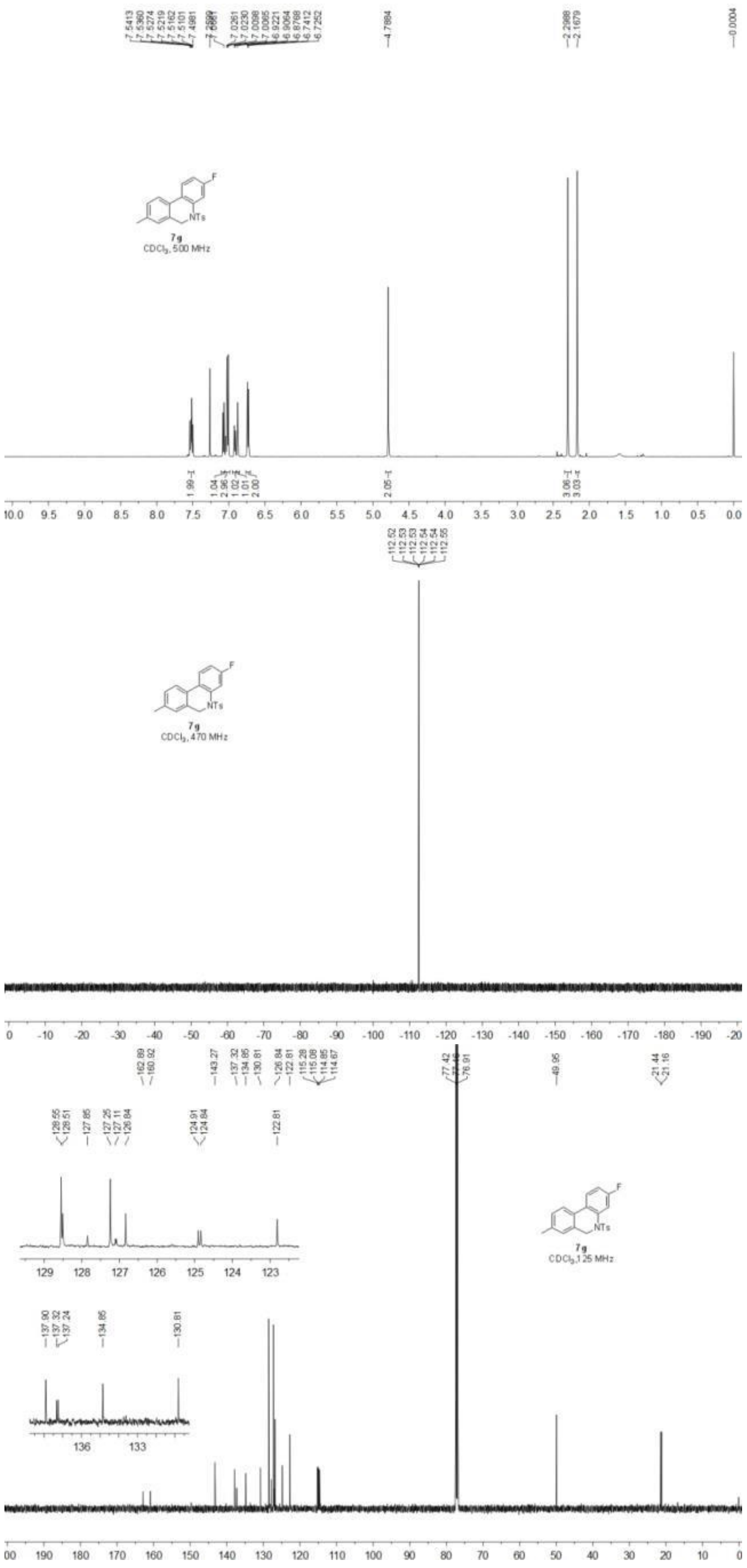

Figure S108. ^{1}H, ^{19}F and ^{13}C NMR spectra of **7g**. Related to **Figure 5**.

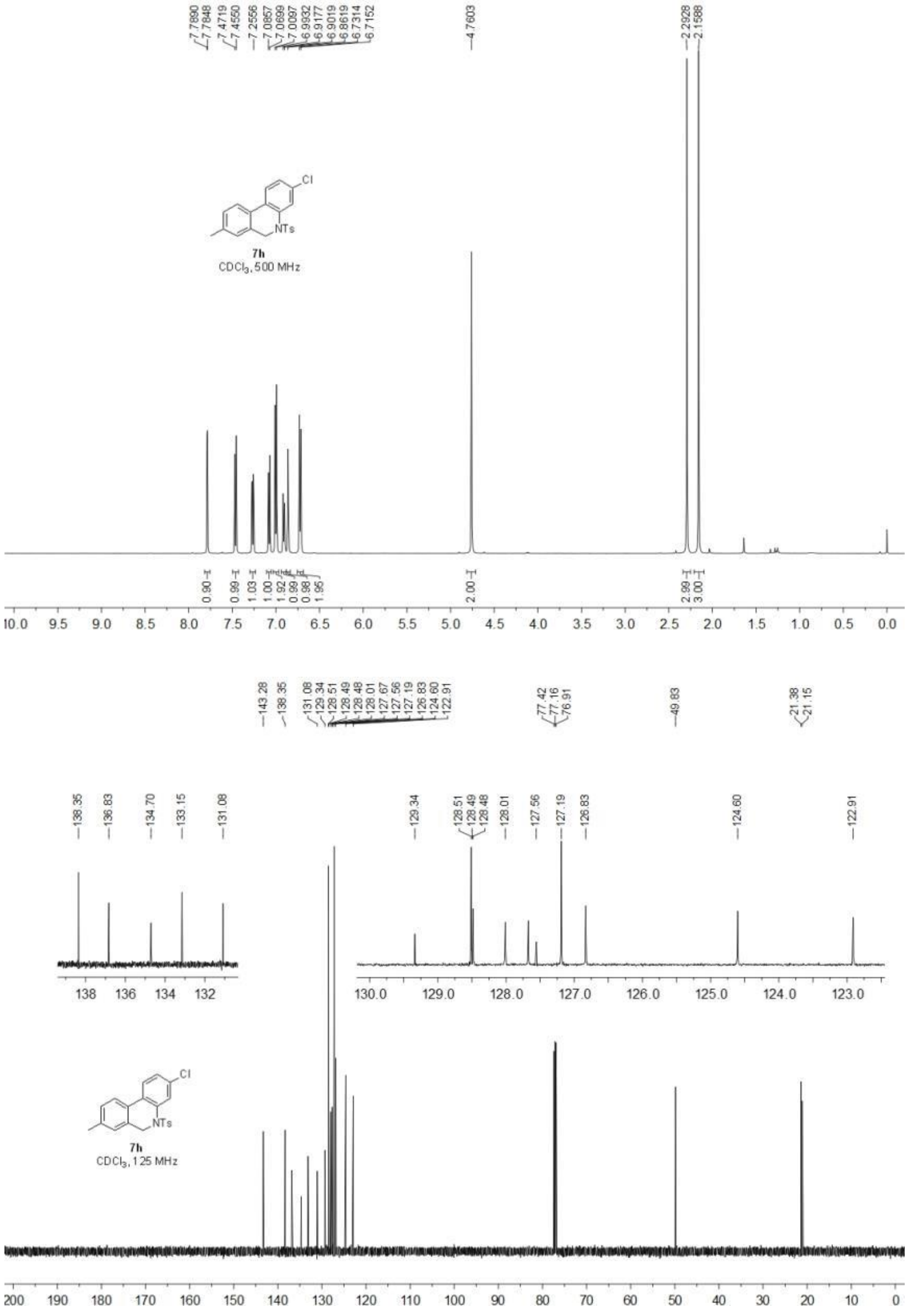

Figure S109. ^{1}H and ^{13}C NMR spectra of **7h**. Related to **Figure 5**.

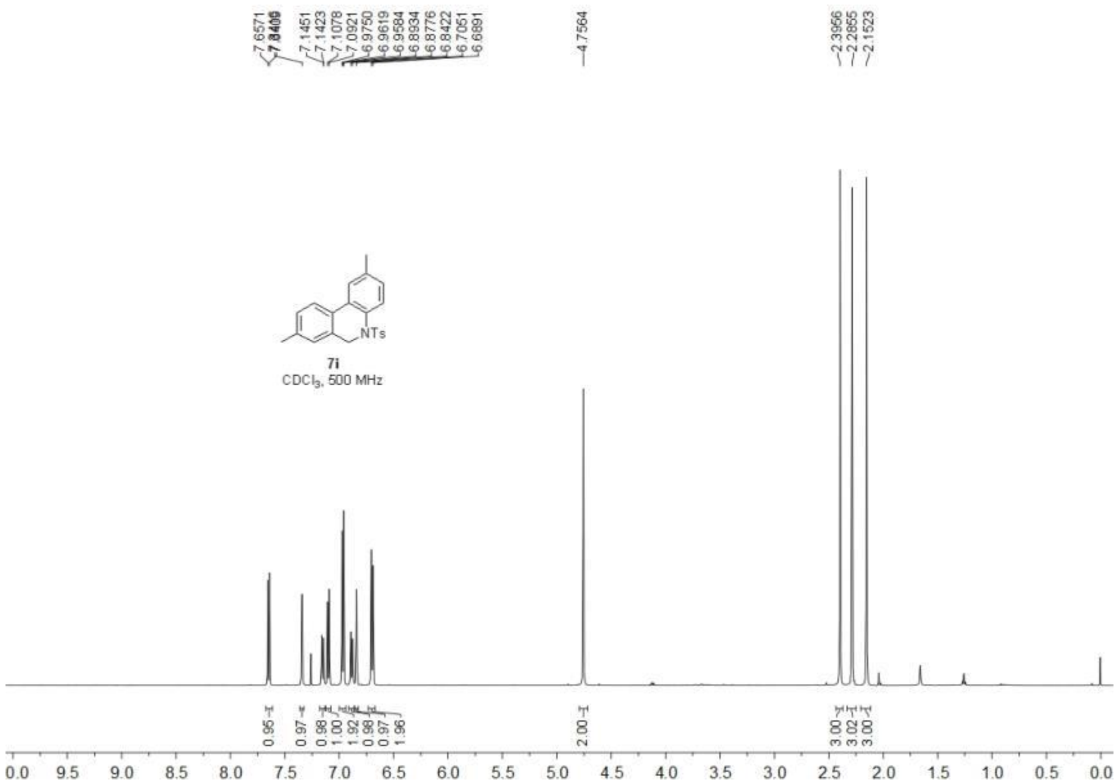

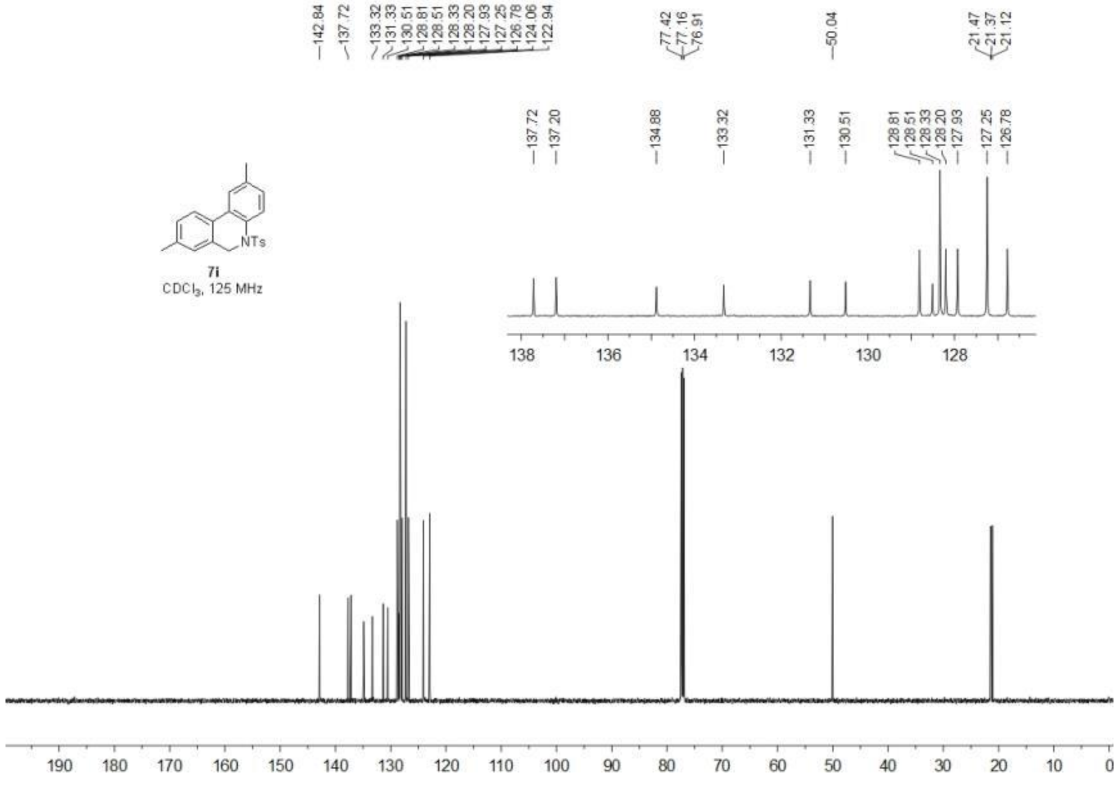

Figure S110. ^{1}H and ^{13}C NMR spectra of **7i**. Related to **Figure 5**.

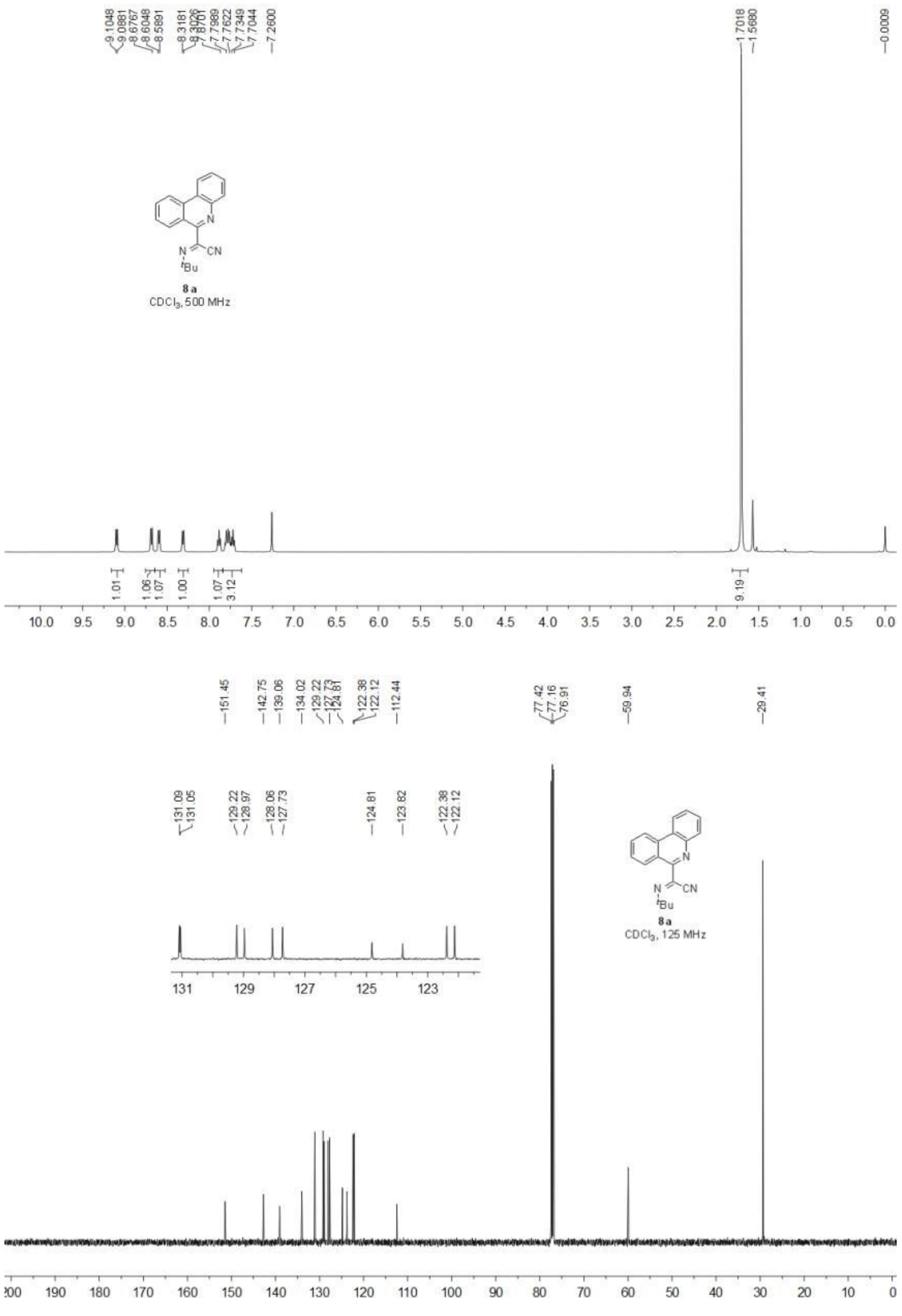

Figure S111. ^{1}H and ^{13}C NMR spectra of **8a**. Related to **Figure 5**.

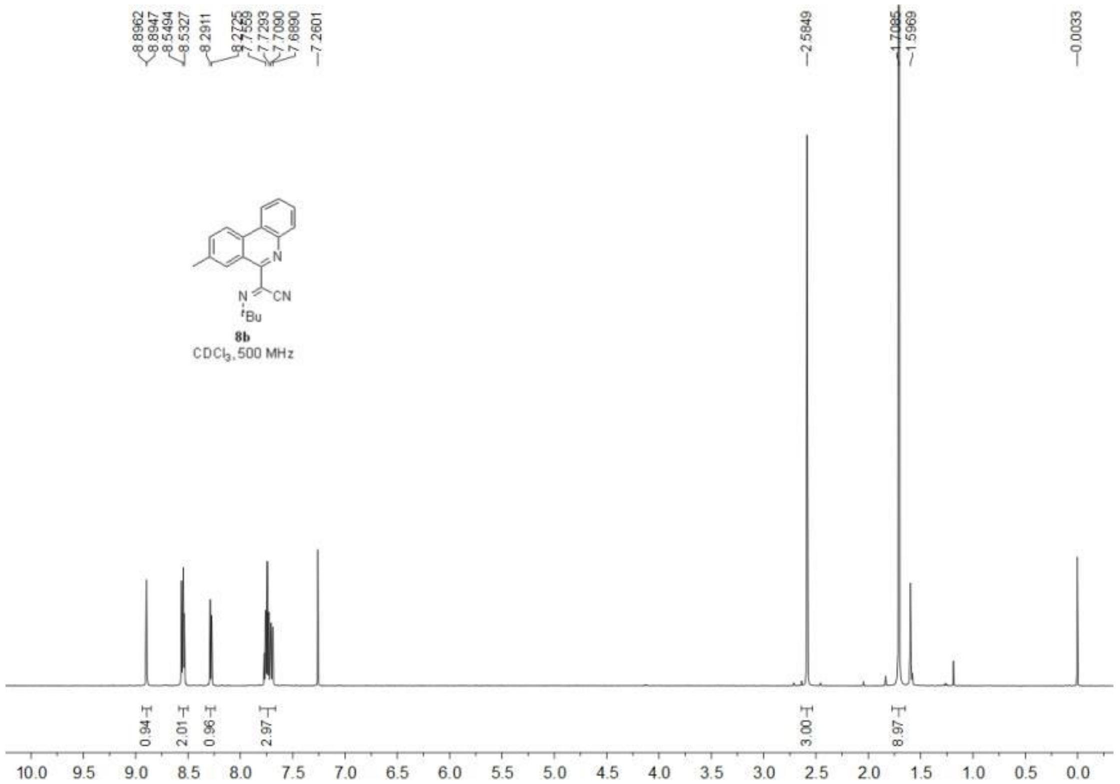

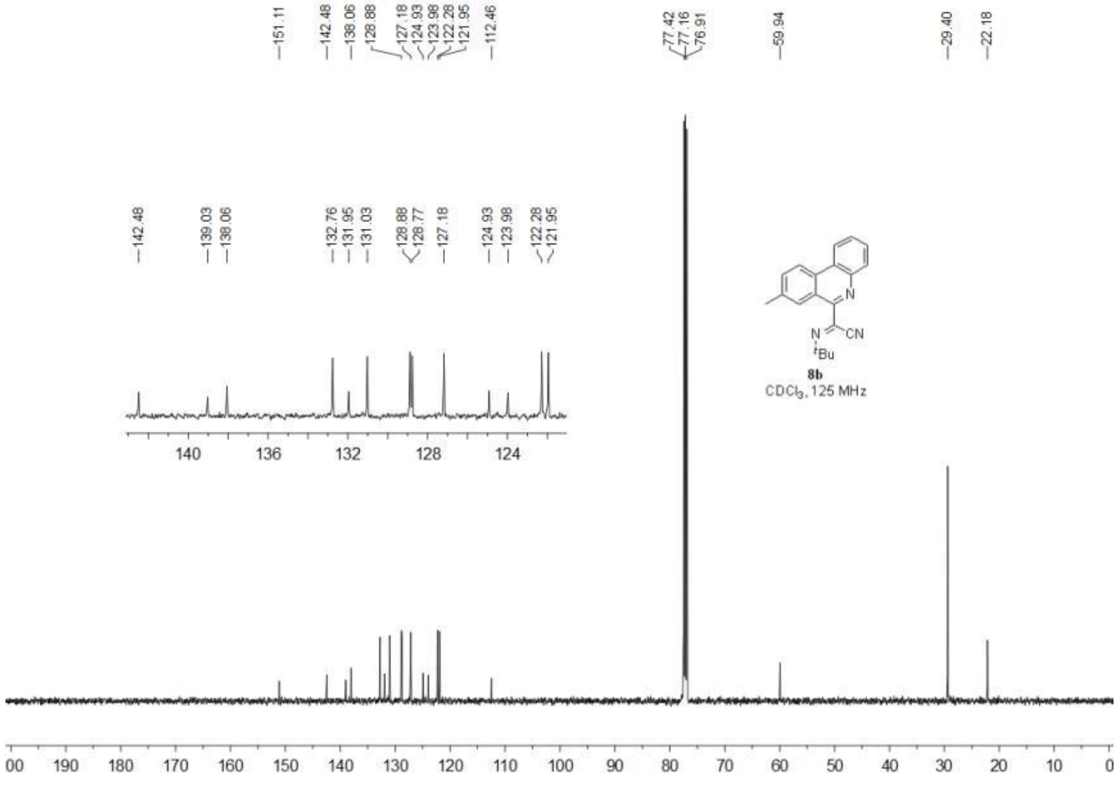

Figure S112. ^{1}H and ^{13}C NMR spectra of **8b**. Related to **Figure 5**.

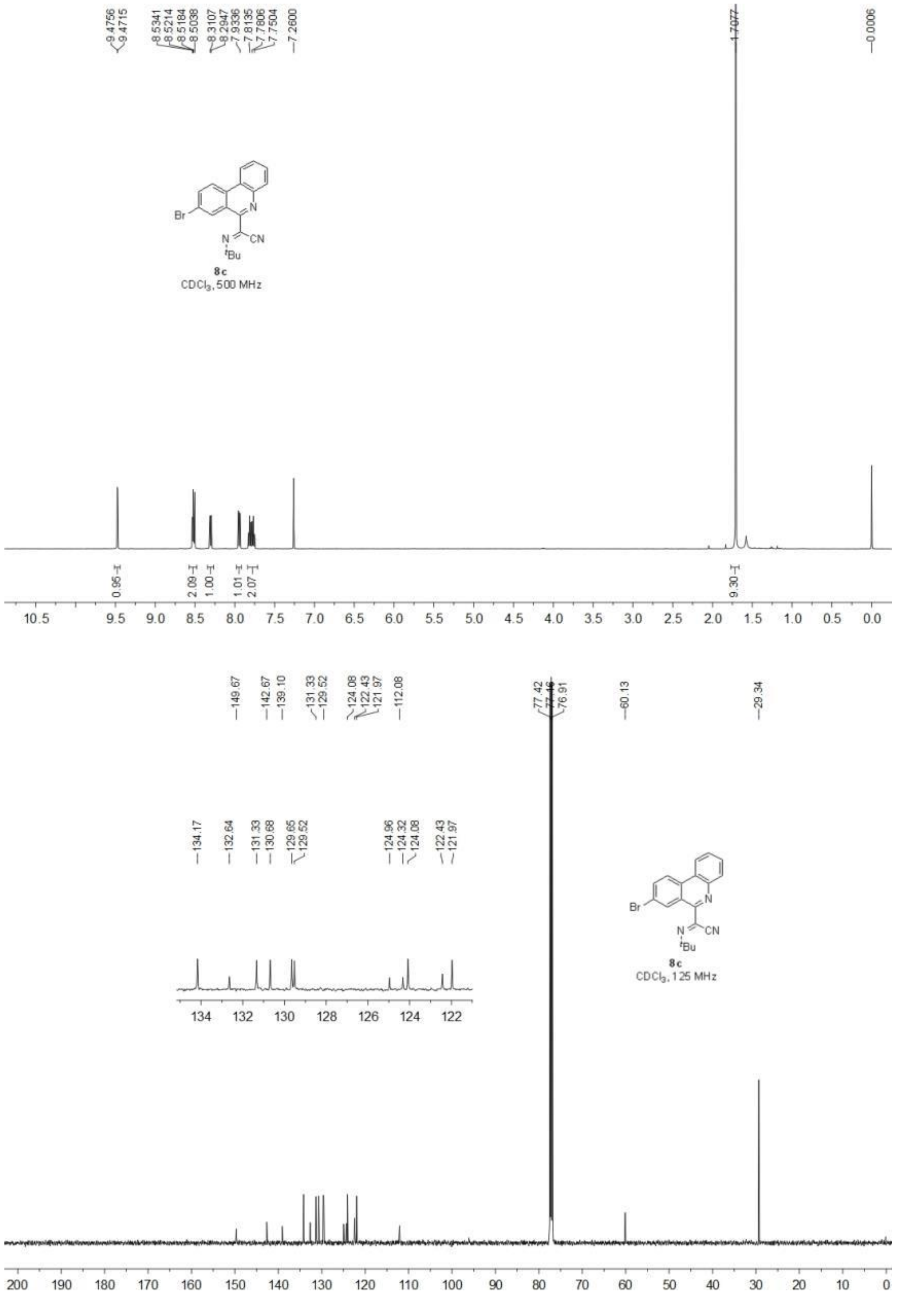

Figure S113. ^{1}H and ^{13}C NMR spectra of **8c**. Related to **Figure 5**.

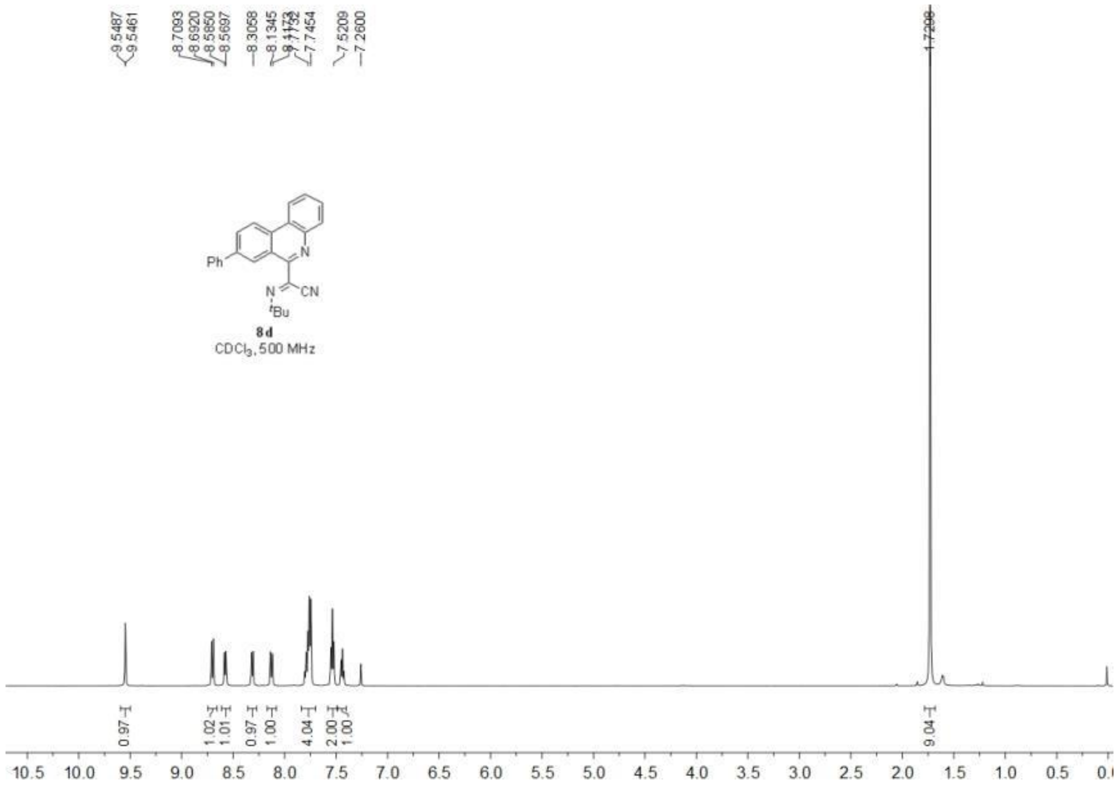

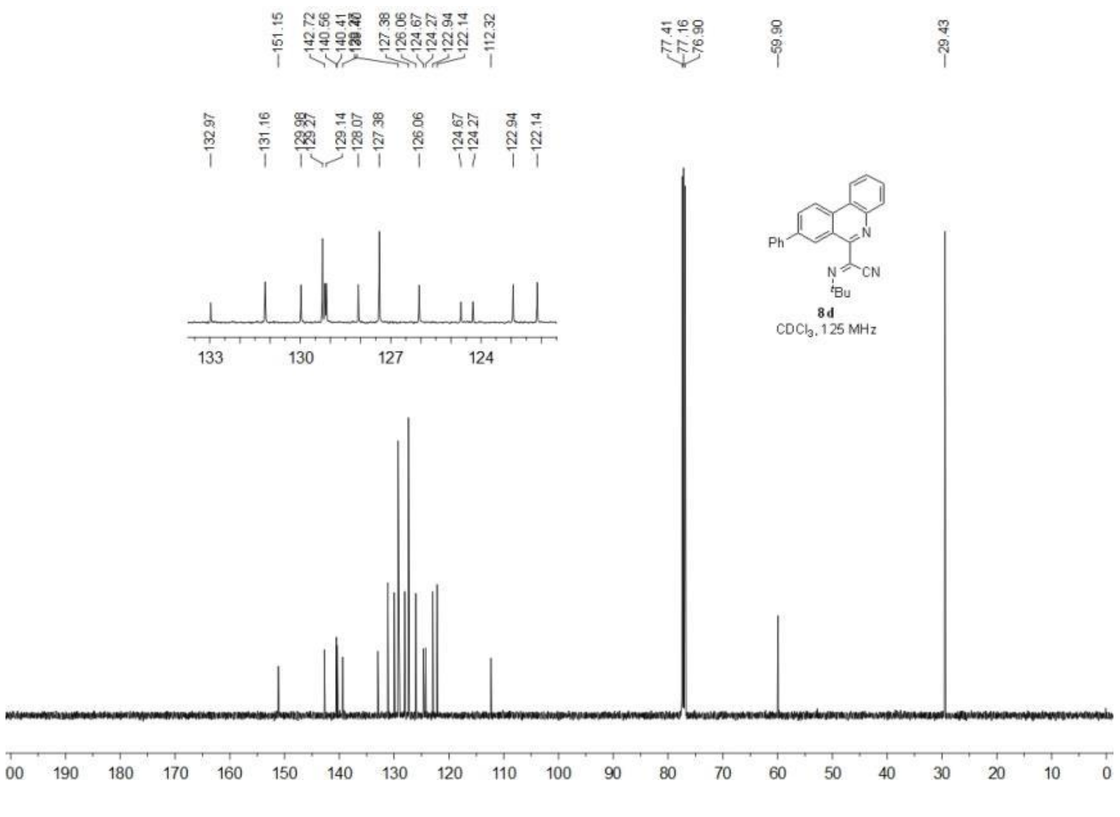

Figure S114. ^{1}H and ^{13}C NMR spectra of **8d**. Related to **Figure 5**.

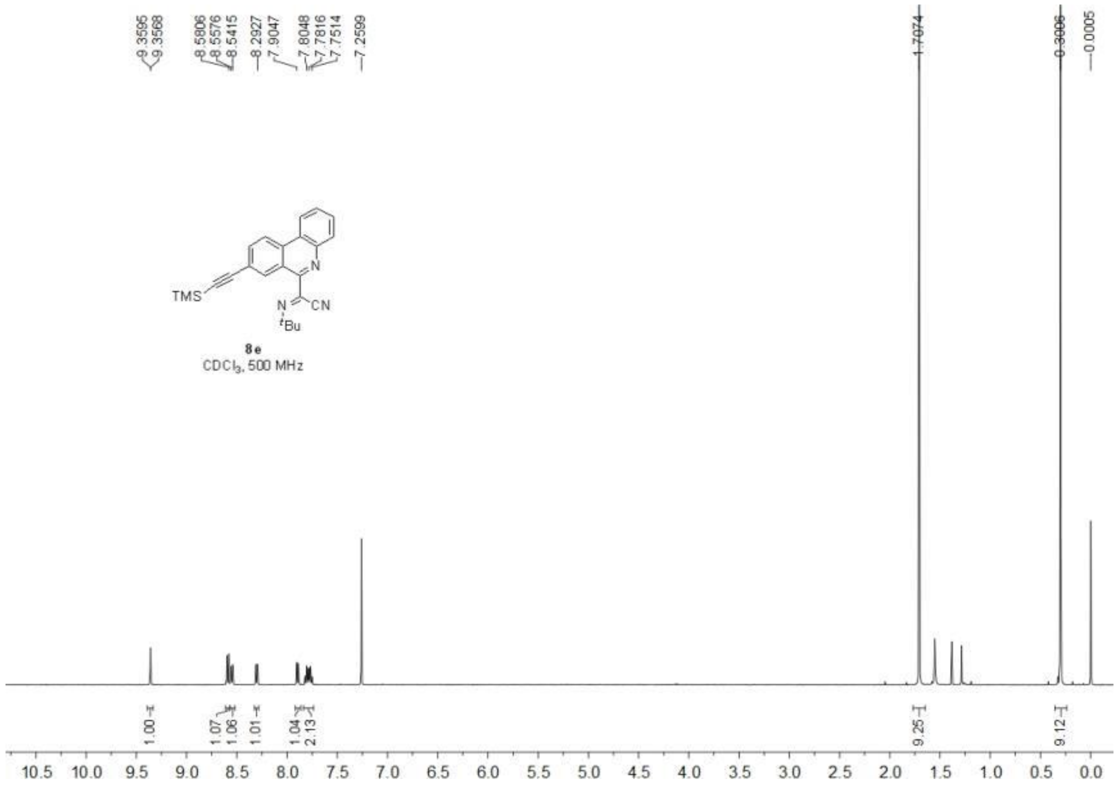

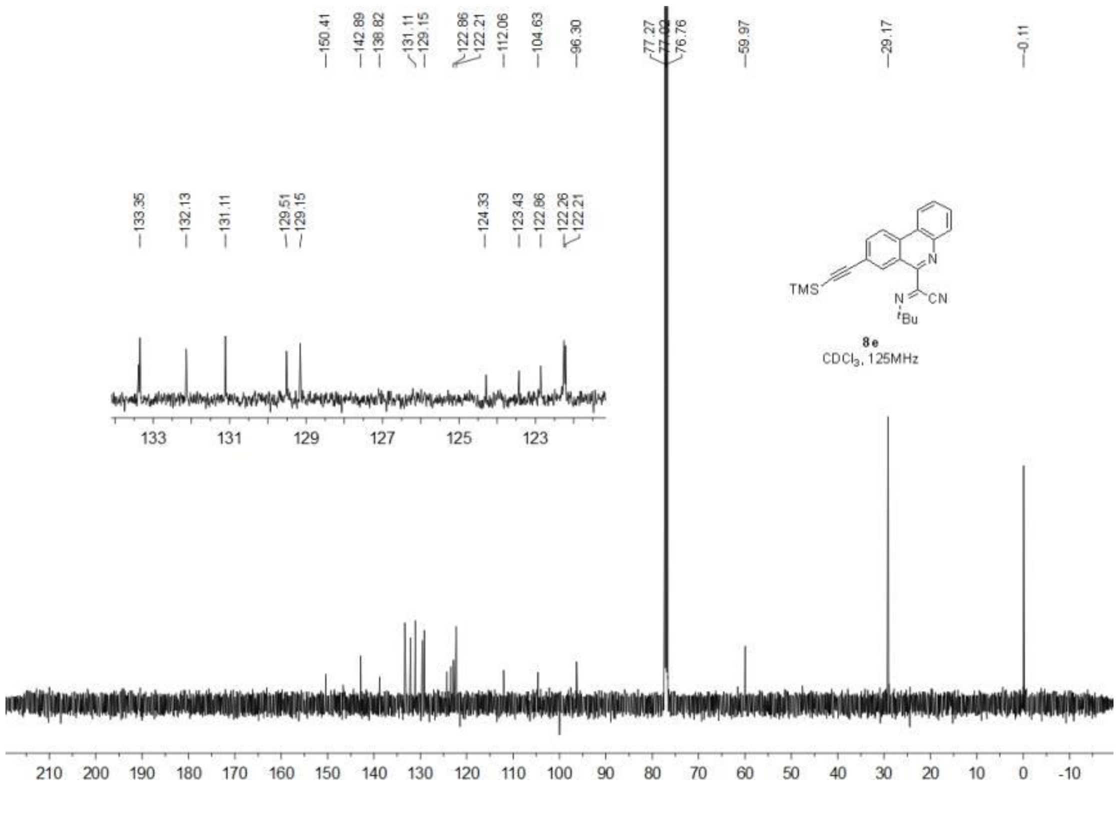

Figure S115. ^{1}H and ^{13}C NMR spectra of **8e.** Related to **Figure 5**.

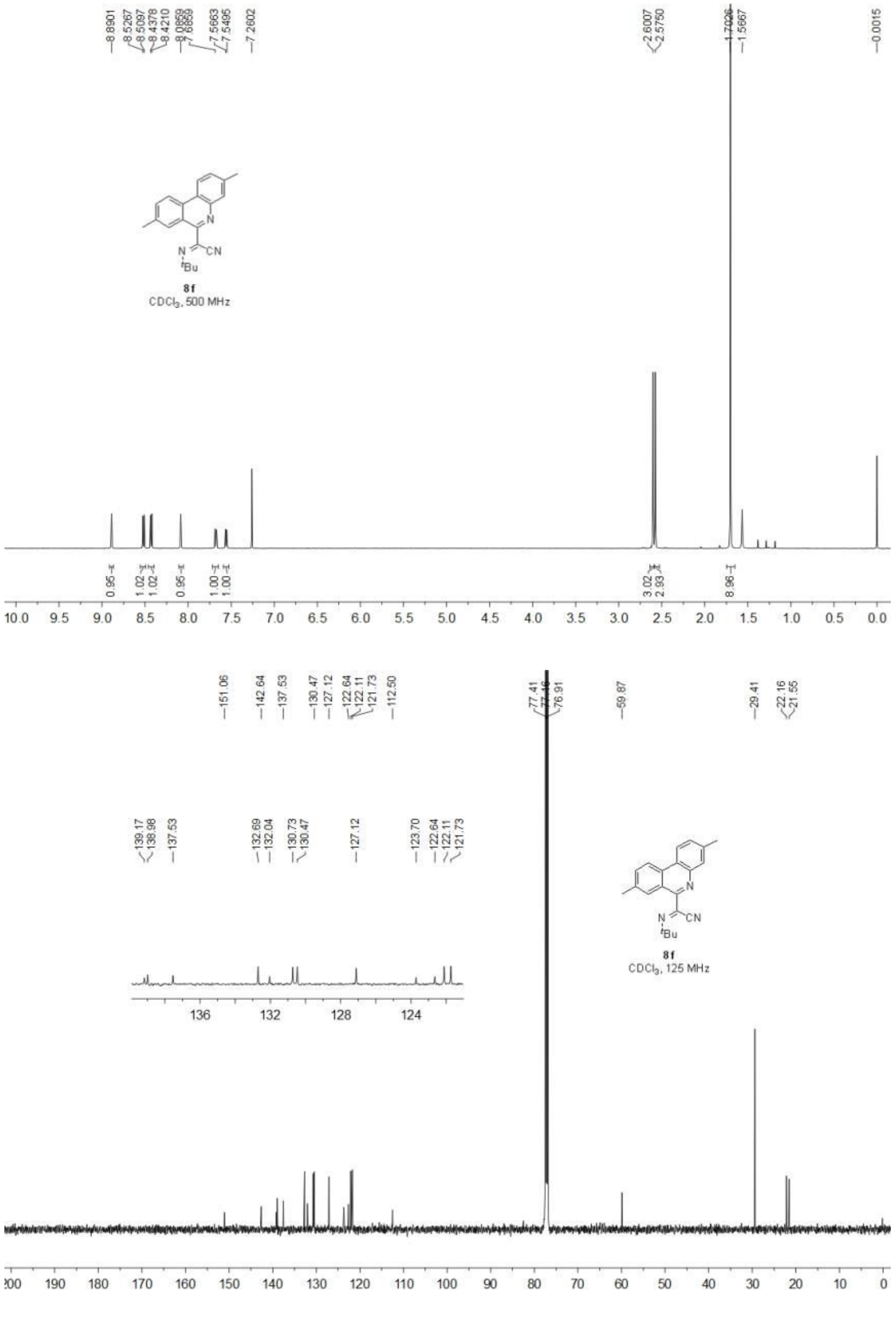

Figure S116. ^{1}H and ^{13}C NMR spectra of **8f**. Related to **Figure 5**.

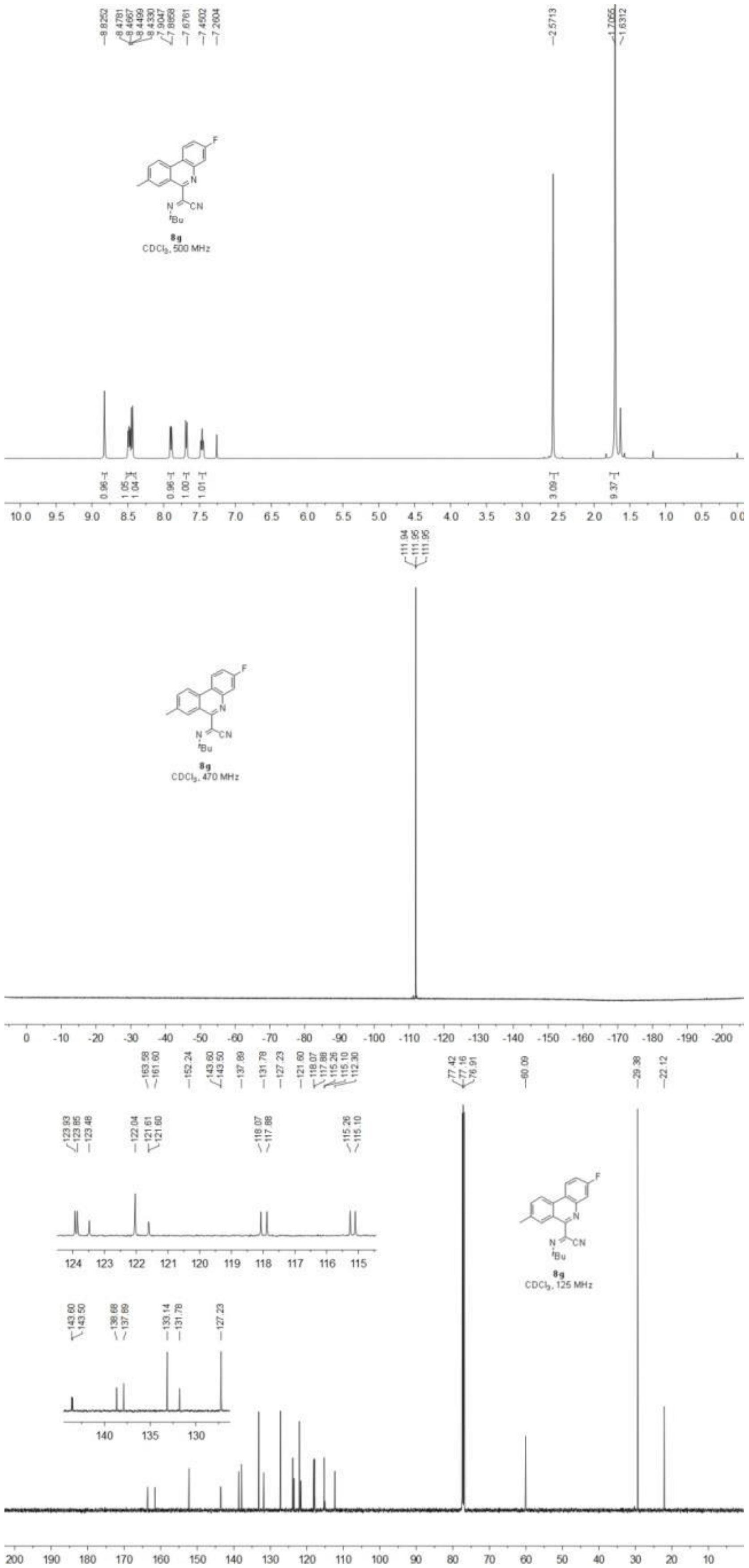

Figure S117. ^{1}H, ^{19}F and ^{13}C NMR spectra of **8g**. Related to **Figure 5**.

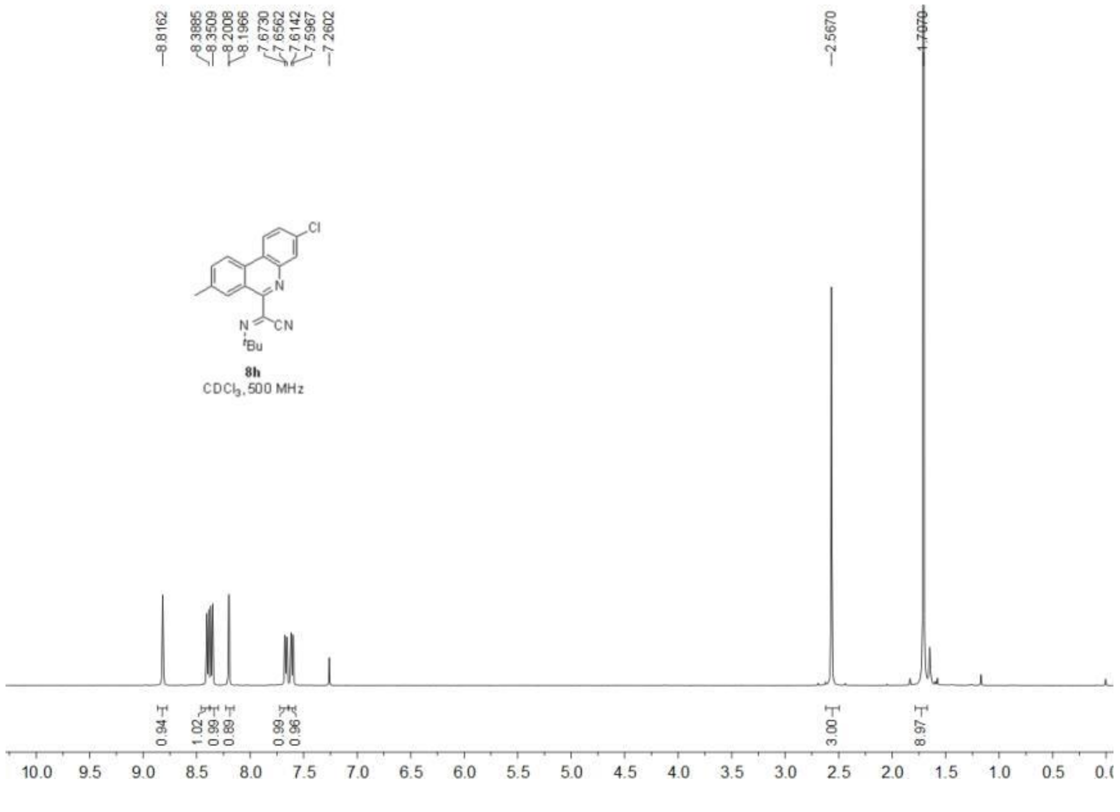

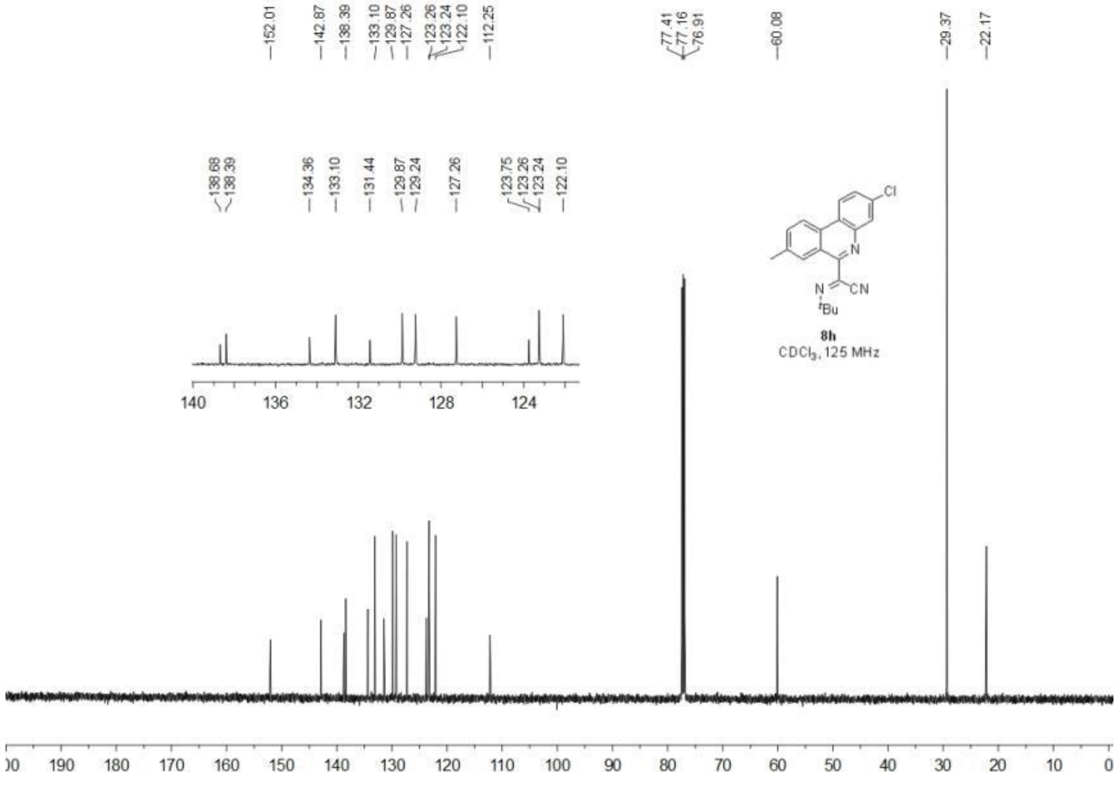

Figure S118. ^{1}H and ^{13}C NMR spectra of **8h**. Related to **Figure 5**.

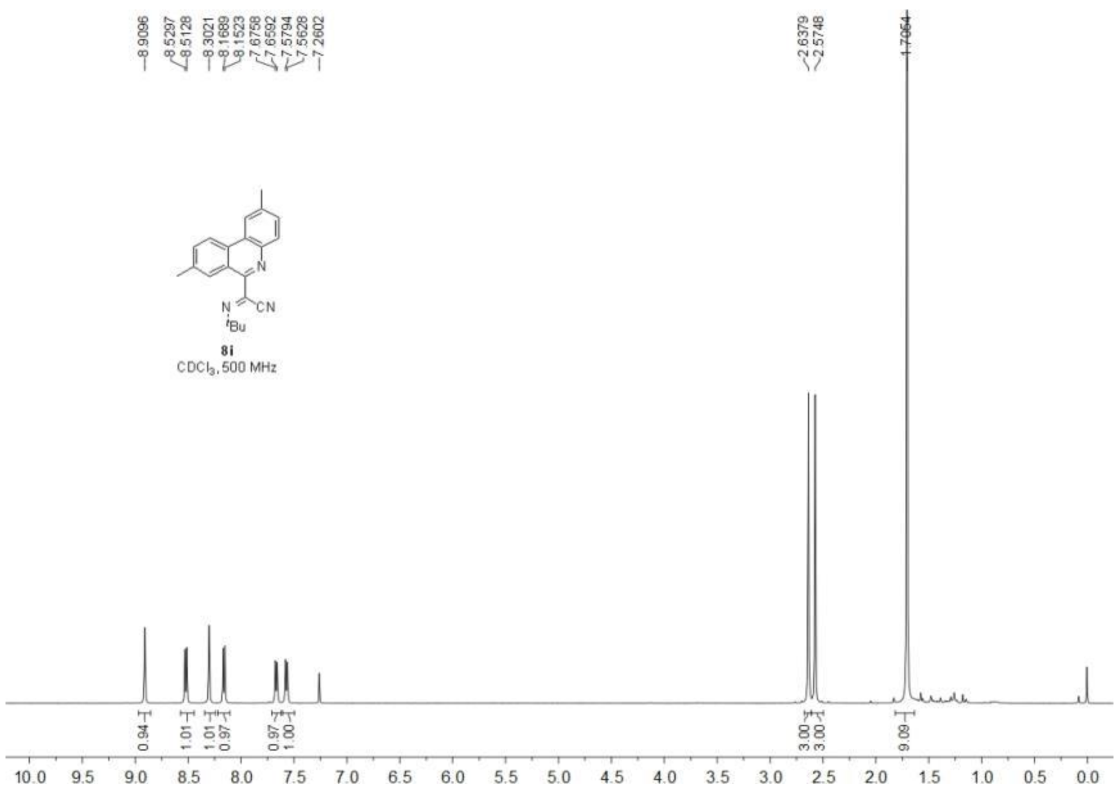

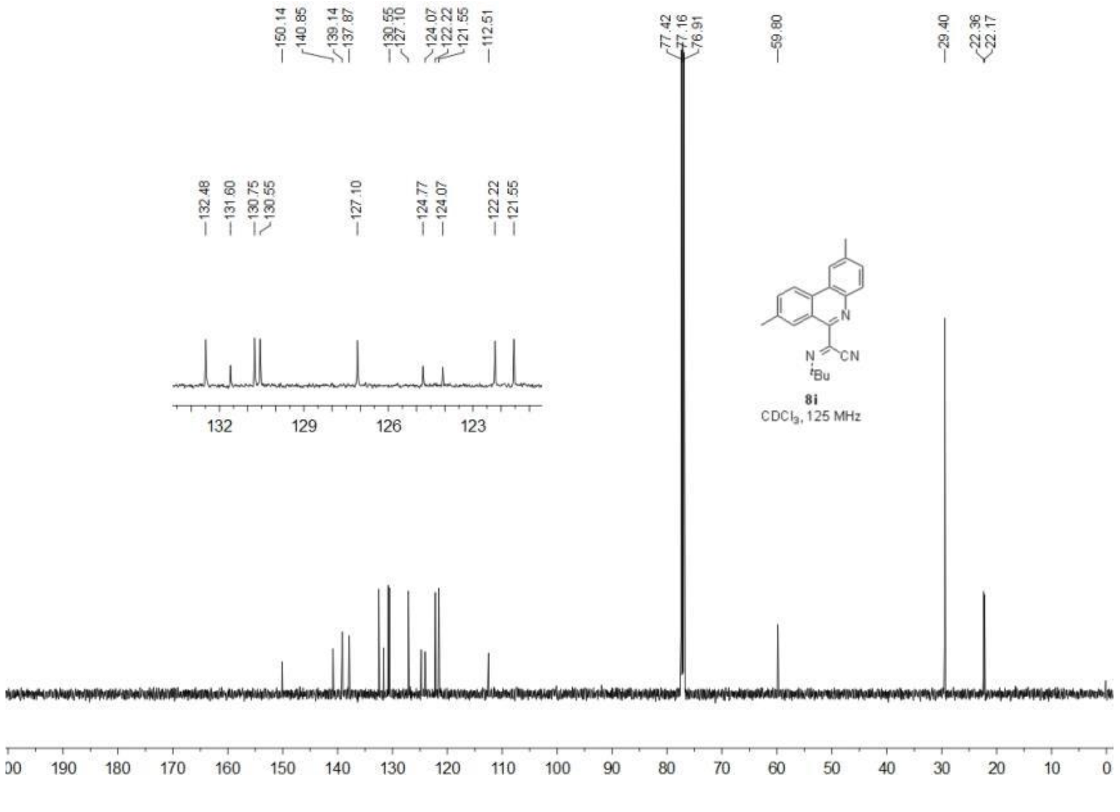

Figure S119. ^{1}H and ^{13}C NMR spectra of **8i**. Related to **Figure 5**.

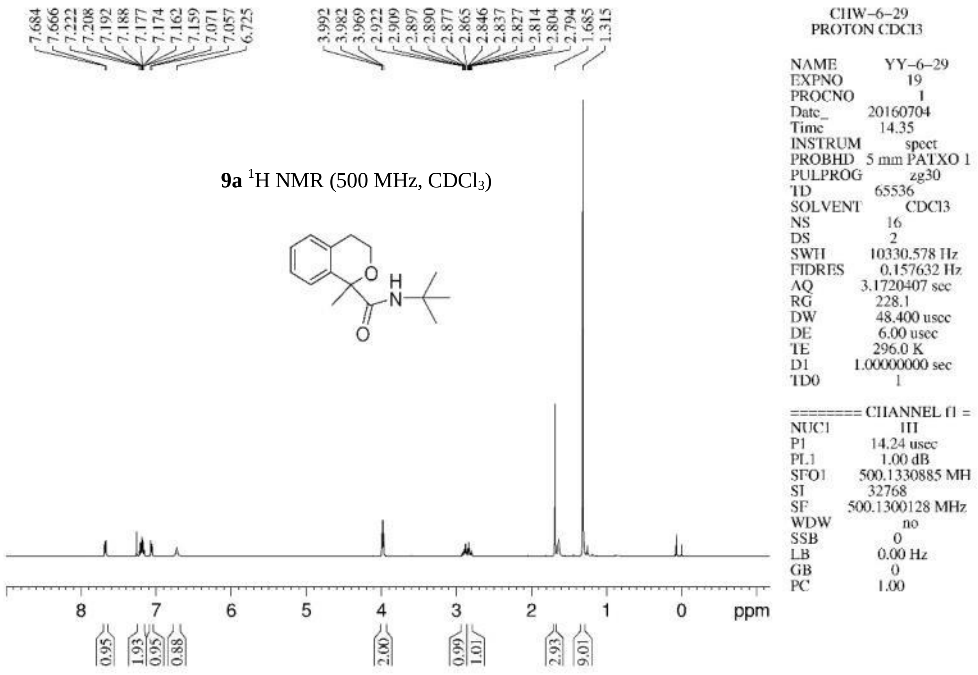

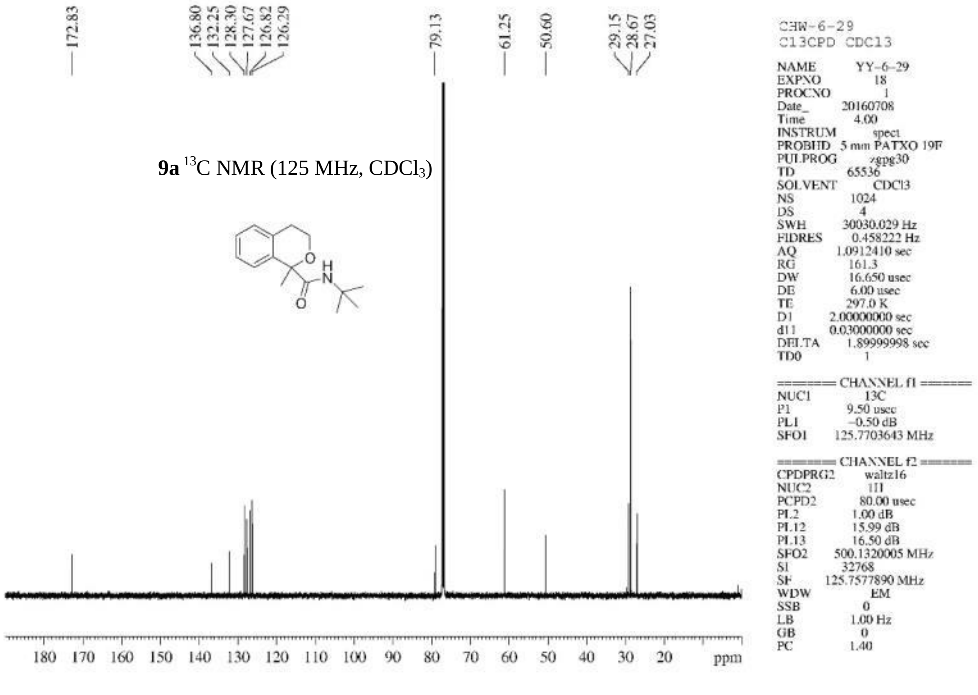

Figure S120. ^{1}H and ^{13}C NMR spectra of **9a**. Related to **Figure 6**.

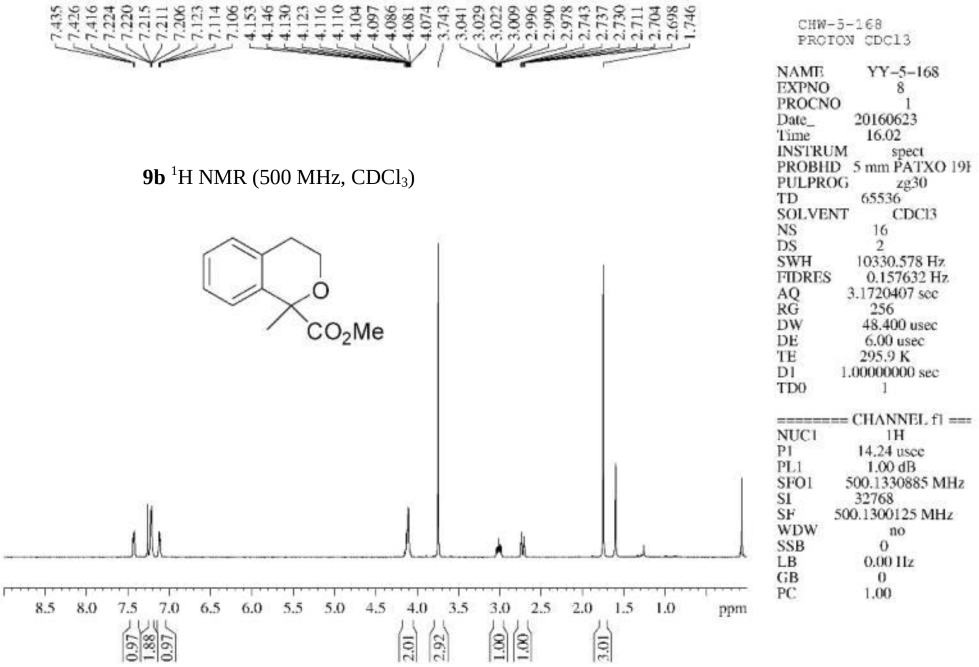

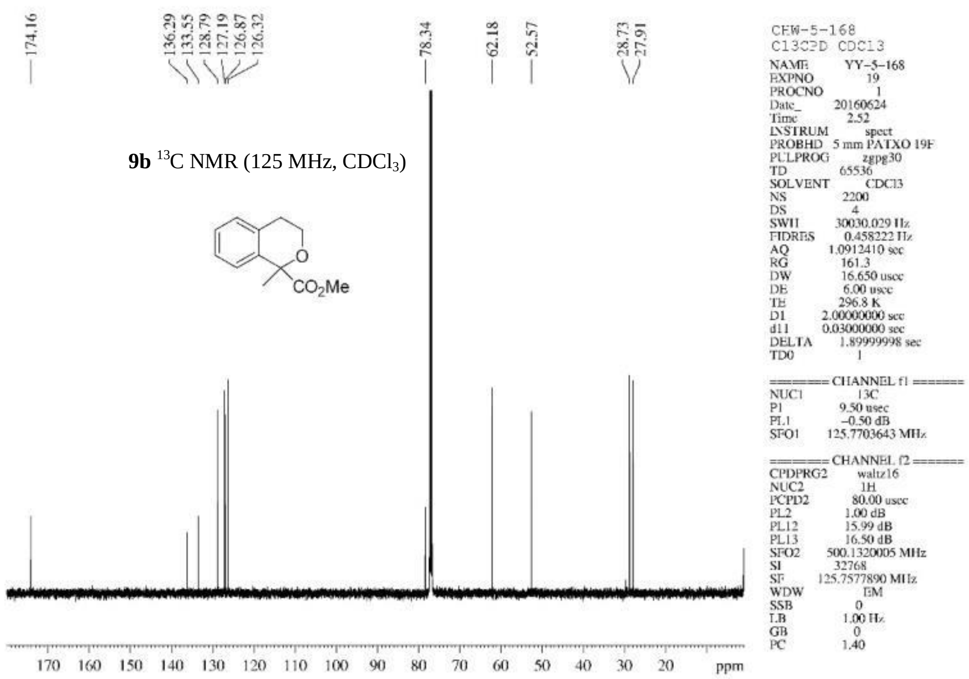

Figure S121. ^{1}H and ^{13}C NMR spectra of **9b**. Related to **Figure 6**.

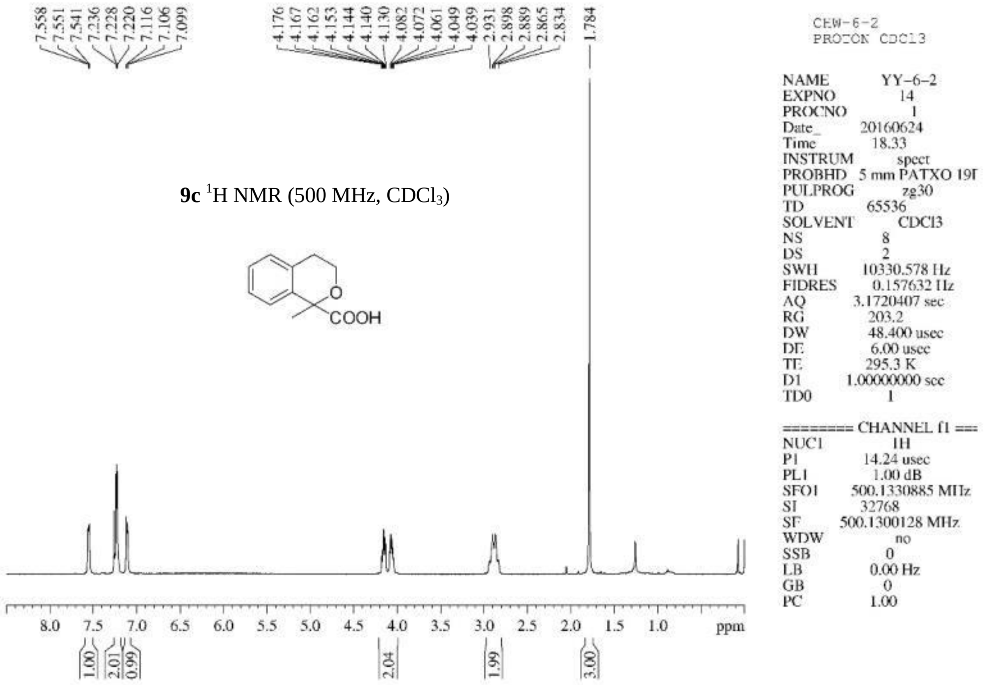

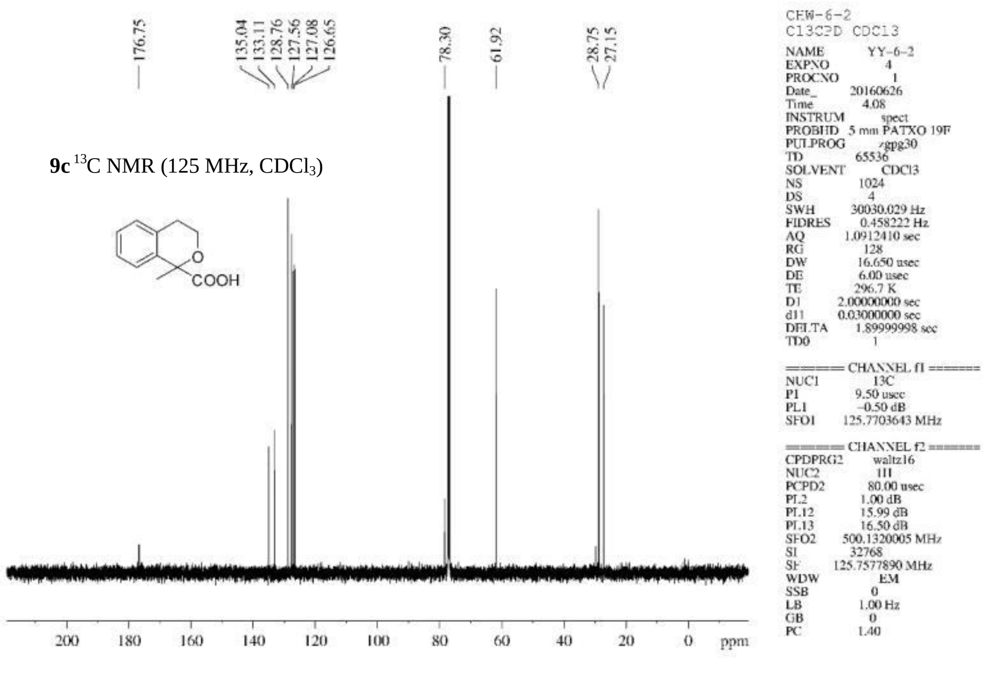

Figure S122. ^{1}H and ^{13}C NMR spectra of **9c**. Related to **Figure 6**.

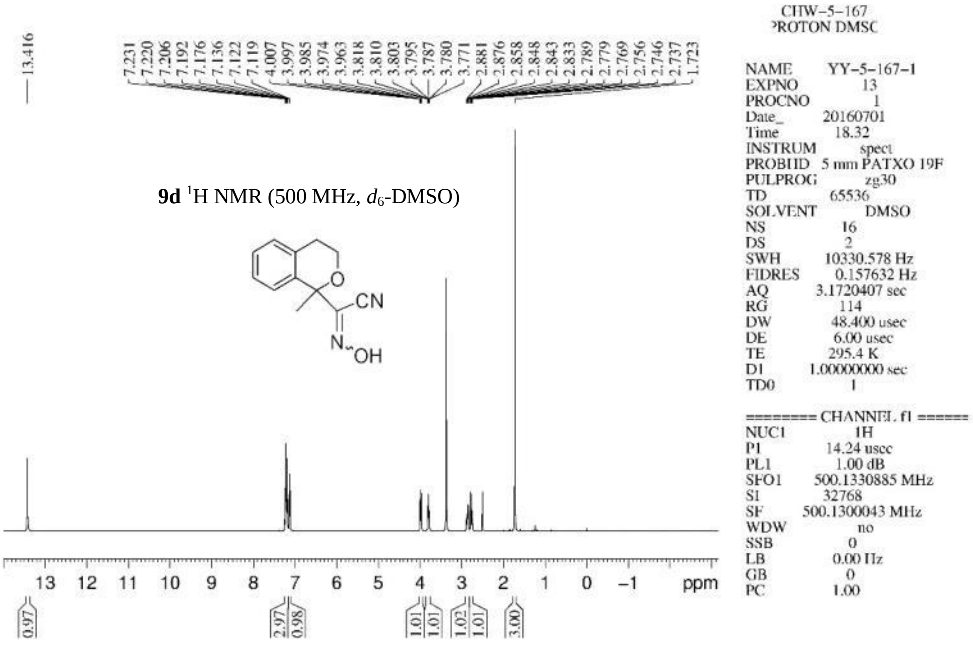

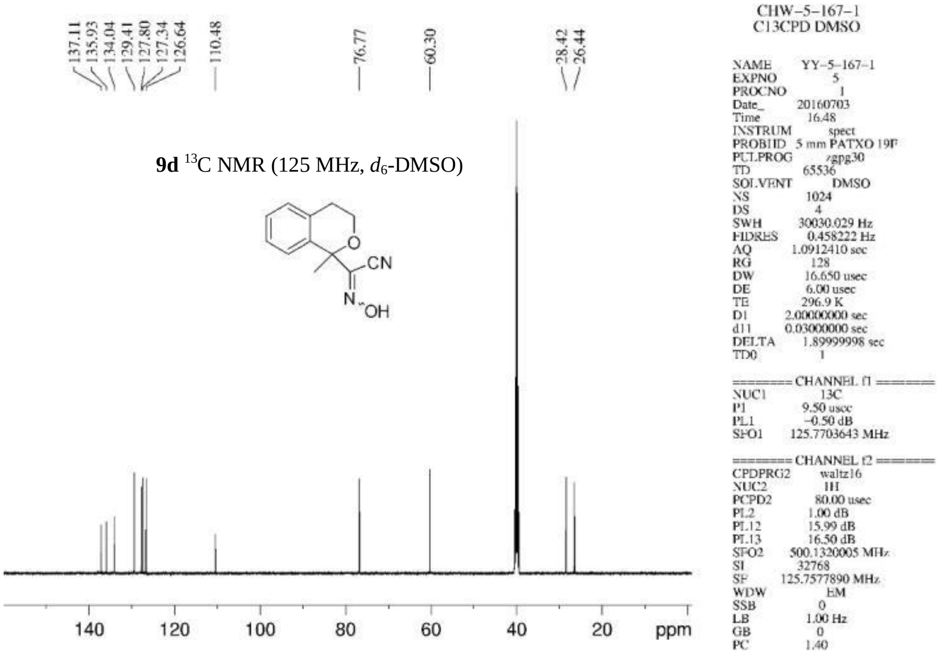

Figure S123. ^{1}H and ^{13}C NMR spectra of **9d**. Related to **Figure 6**.

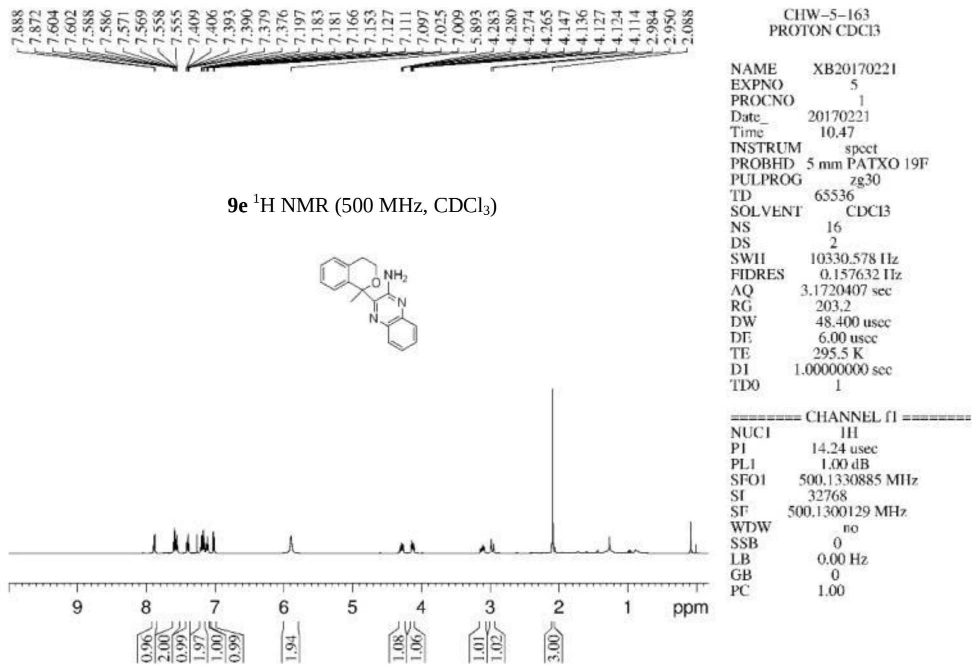

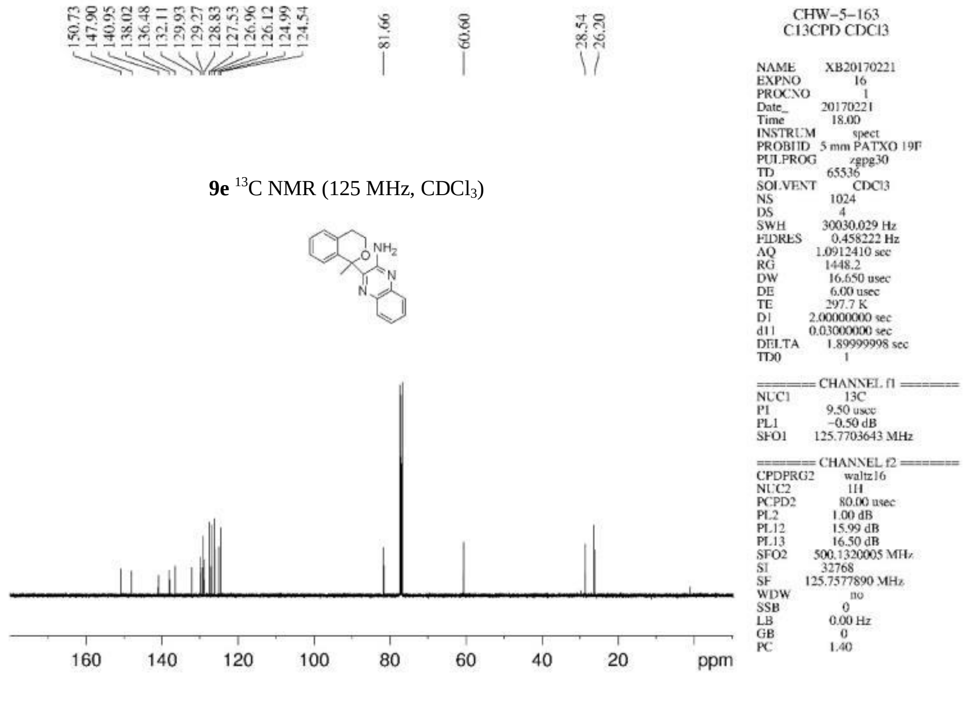

Figure S124. ^{1}H and ^{13}C NMR spectra of **9e**. Related to **Figure 6**.

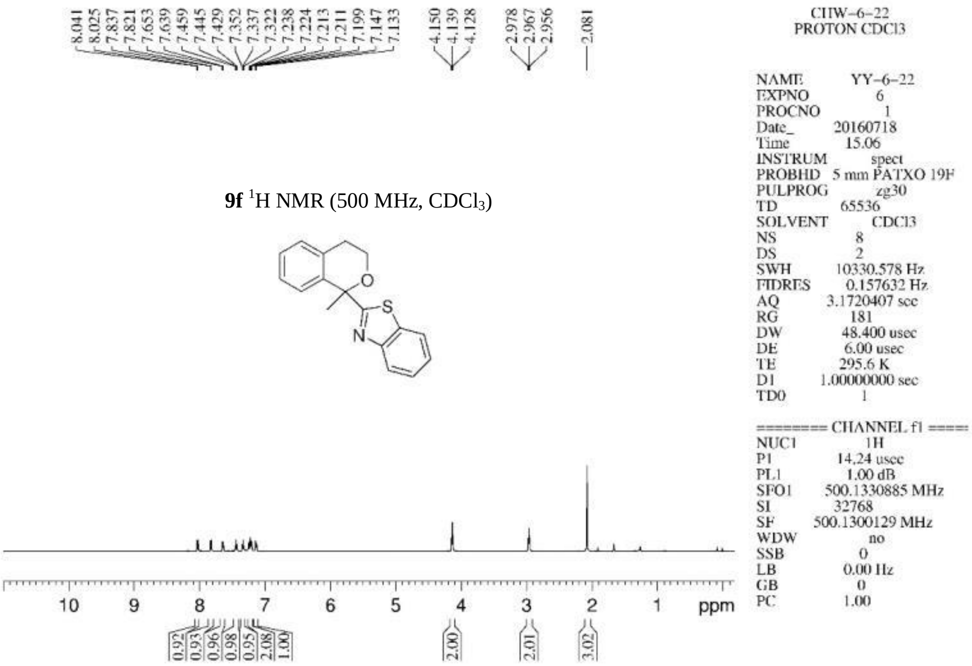

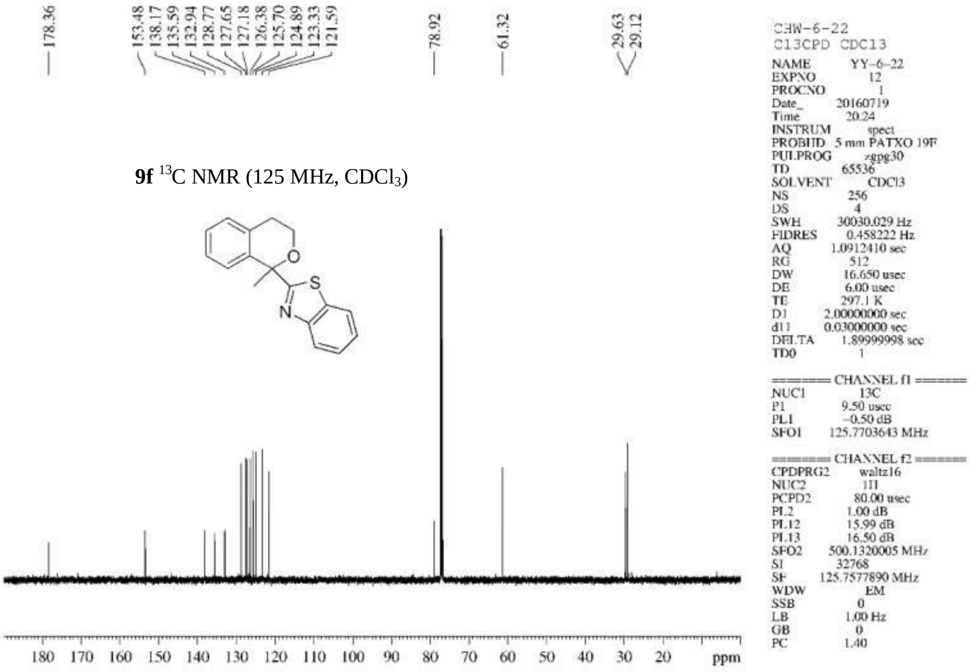

Figure S125. ^{1}H and ^{13}C NMR spectra of **9f**. Related to **Figure 6**.

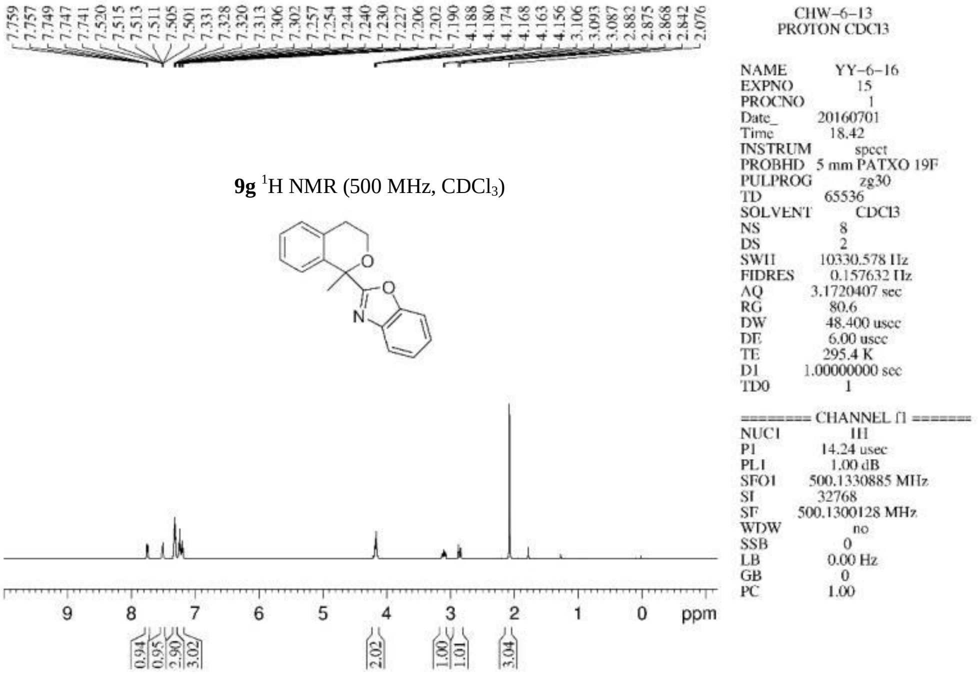

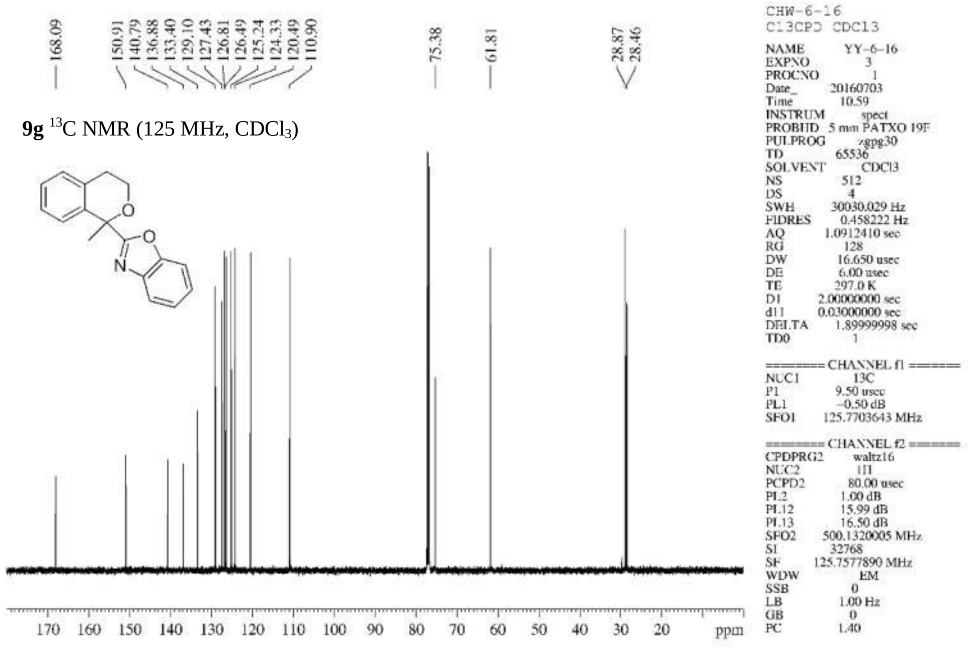

Figure S126. ^{1}H and ^{13}C NMR spectra of **9g**. Related to **Figure 6**.

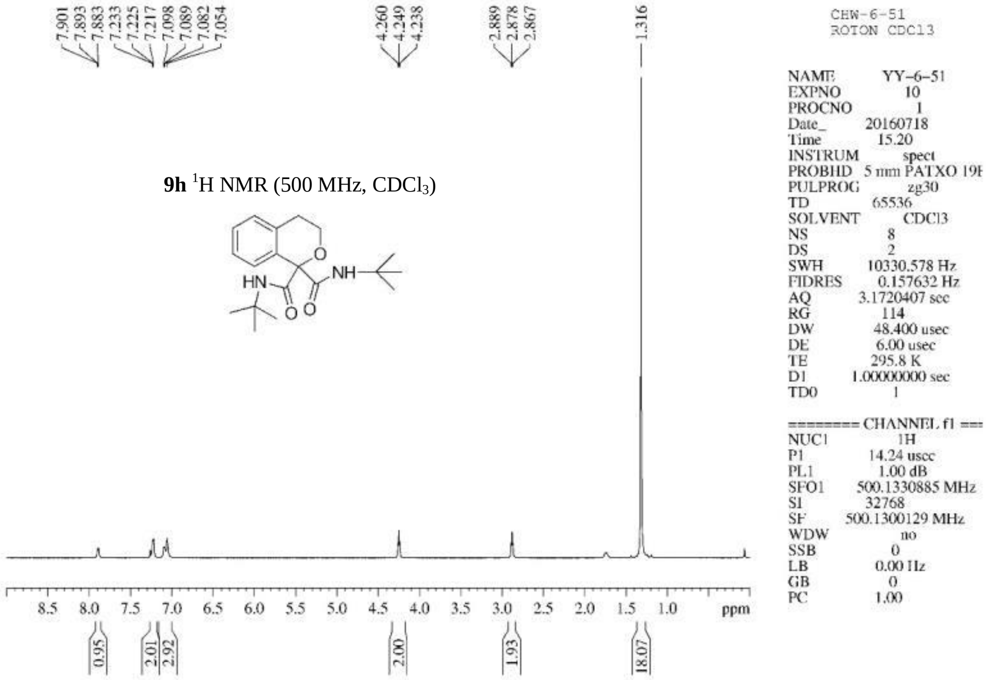

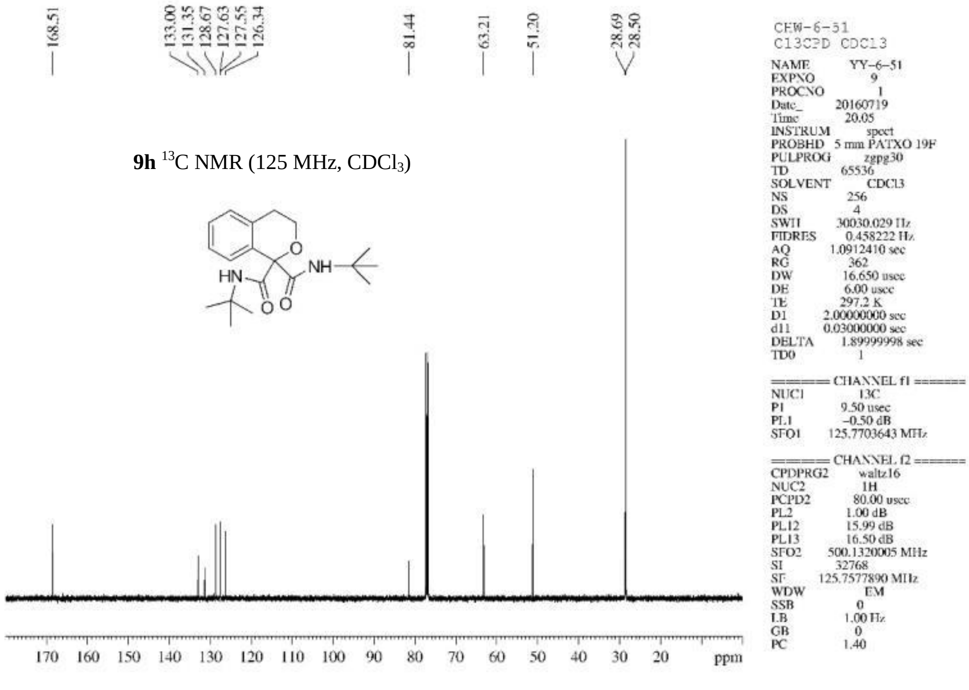

Figure S127. ^{1}H and ^{13}C NMR spectra of **9h**. Related to **Figure 6**.

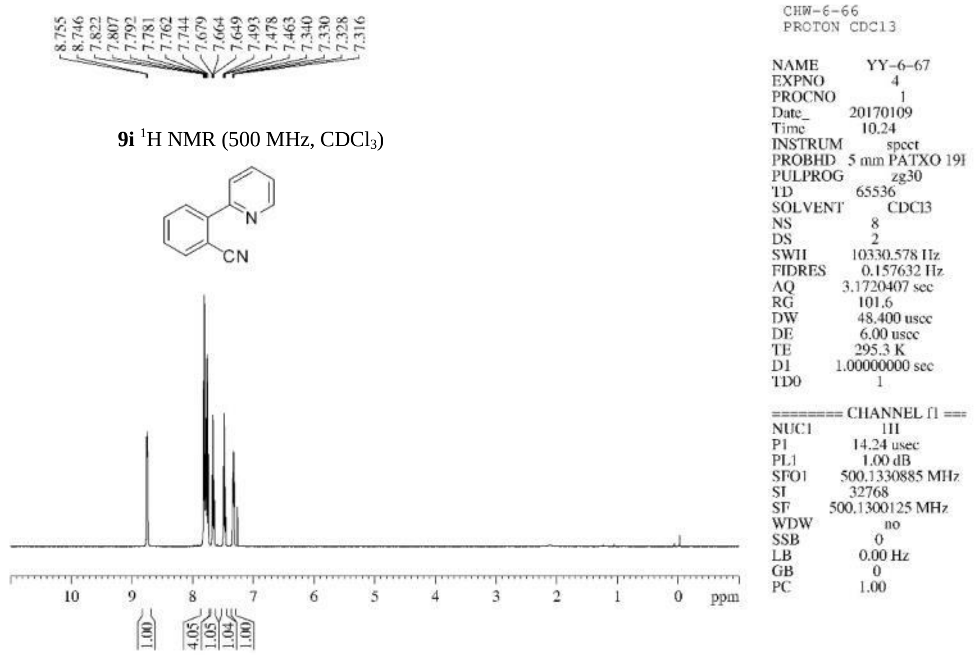

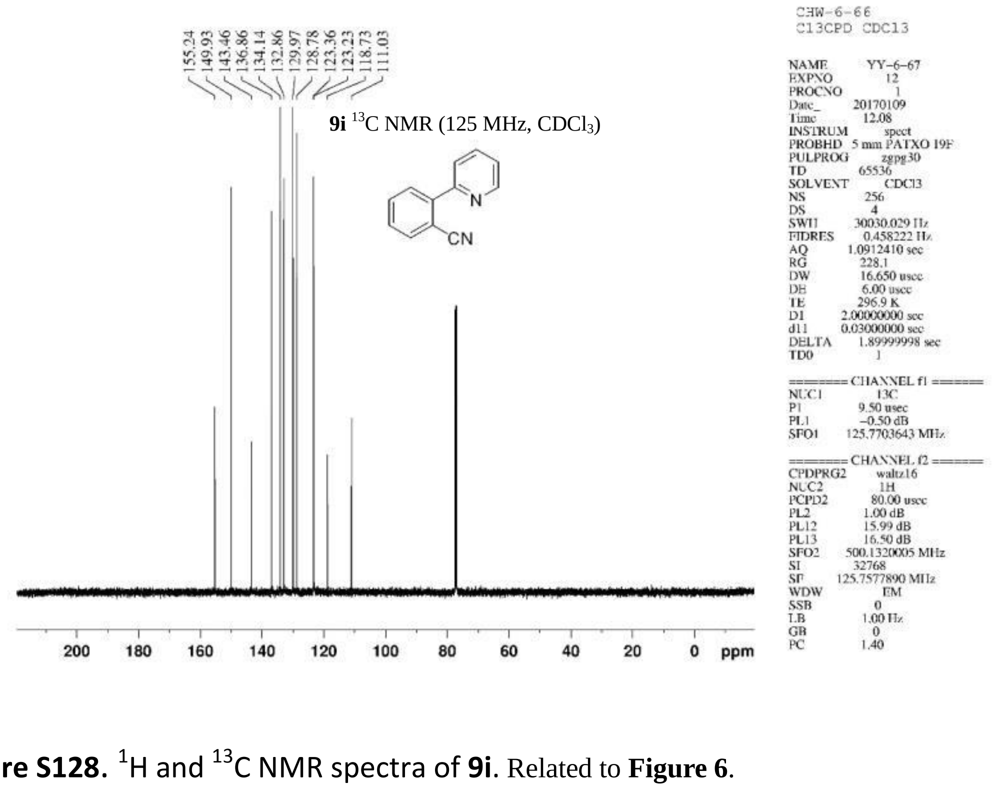

Figure S128. ^{1}H and ^{13}C NMR spectra of **9i**. Related to **Figure 6**.

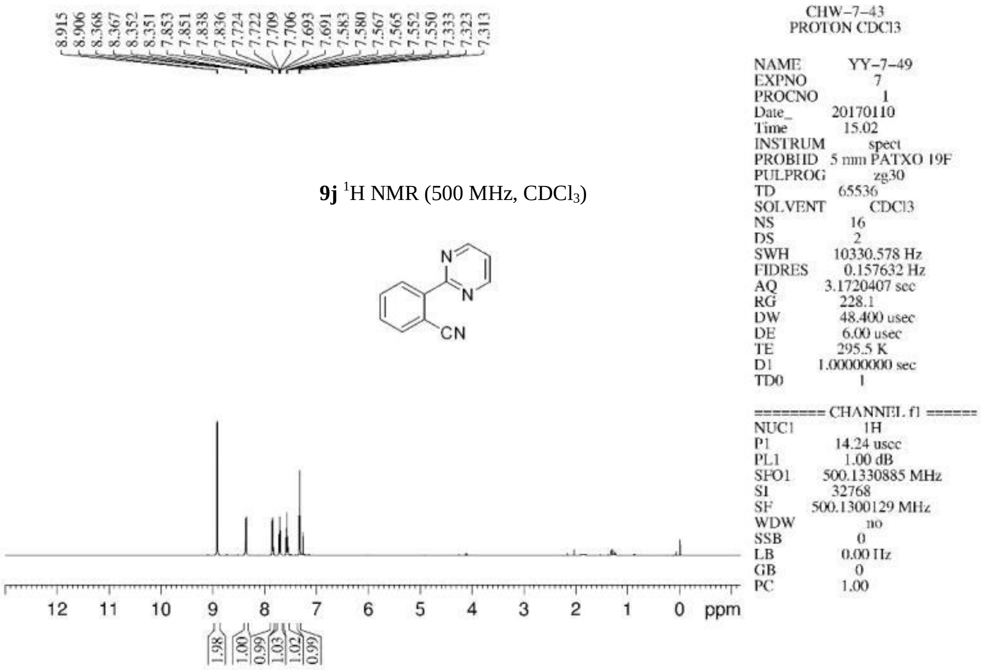

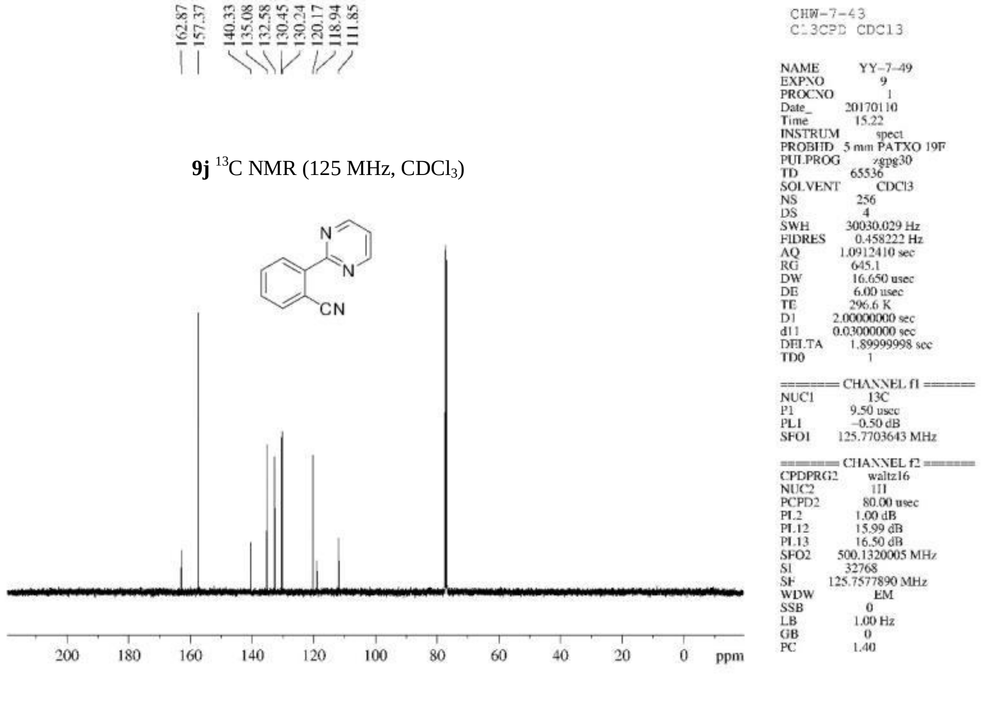

Figure S129. ^{1}H and ^{13}C NMR spectra of **9j**. Related to **Figure 6**.

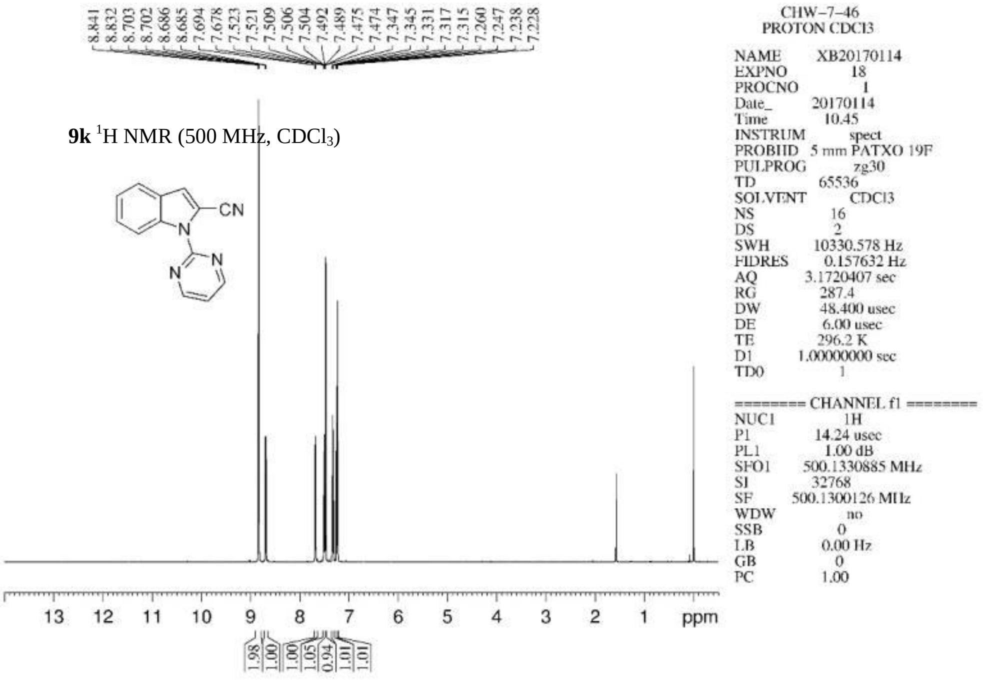

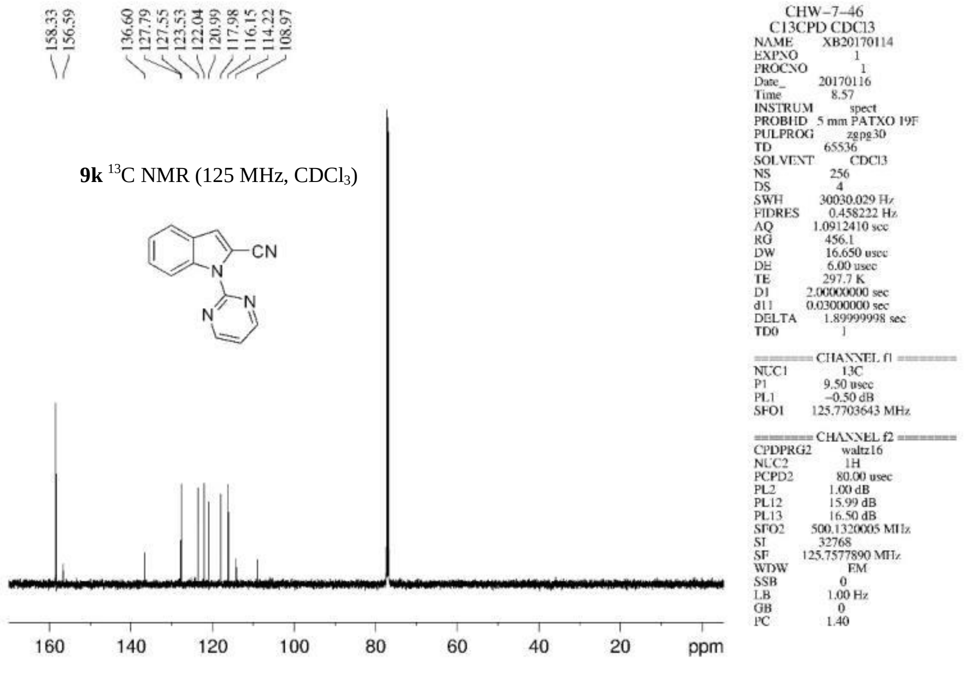

Figure S130. ¹H and ¹³C NMR spectra of **9k**. Related to **Figure 6**.

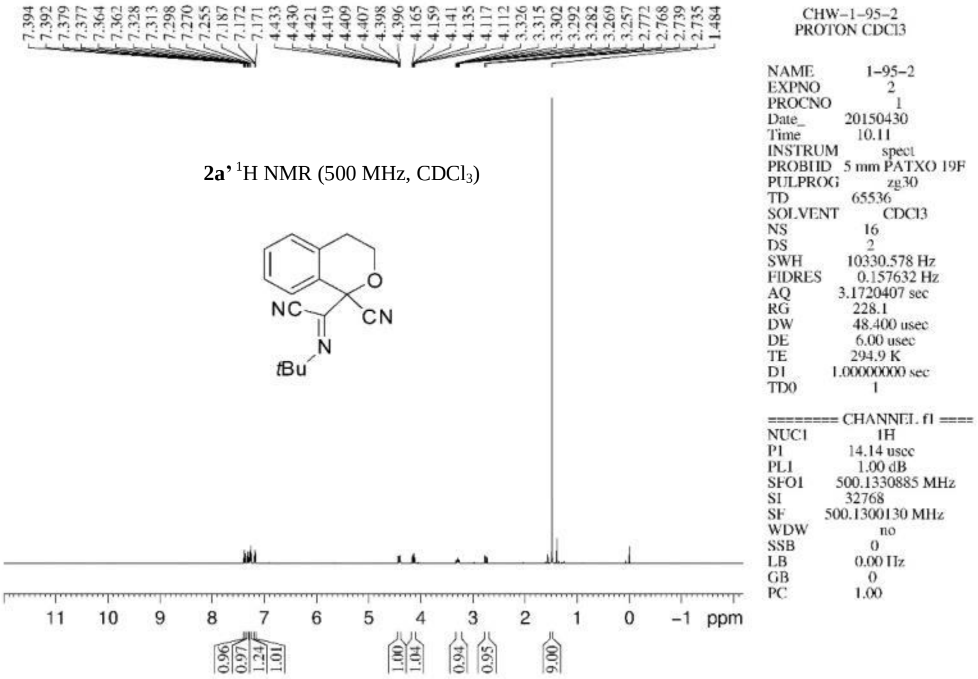

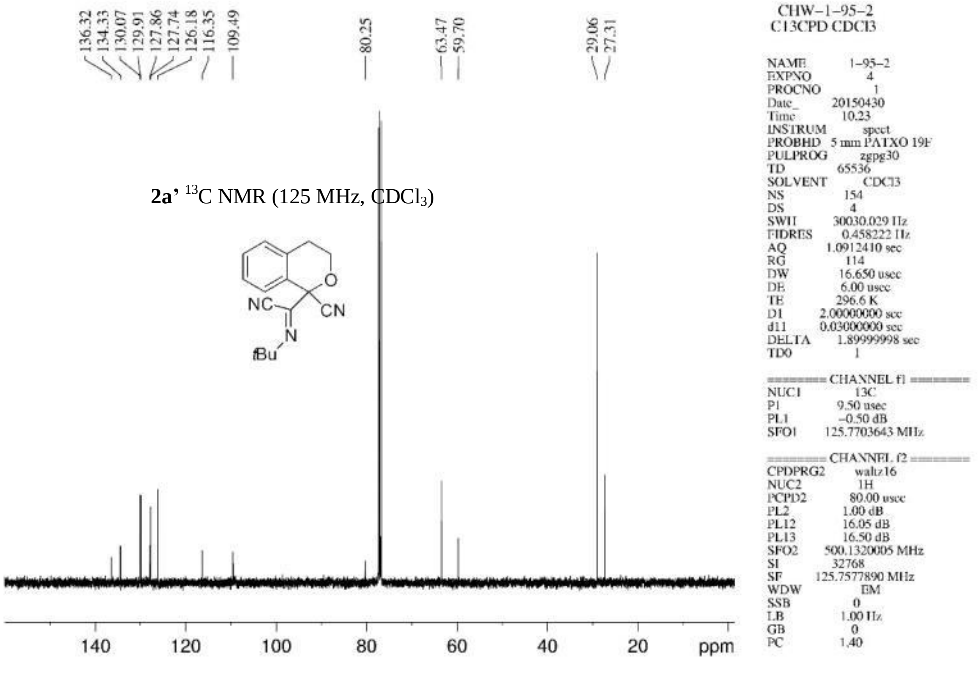

Figure S131. ^{1}H and ^{13}C NMR spectra of **2a'**. Related to **Figure 8**.

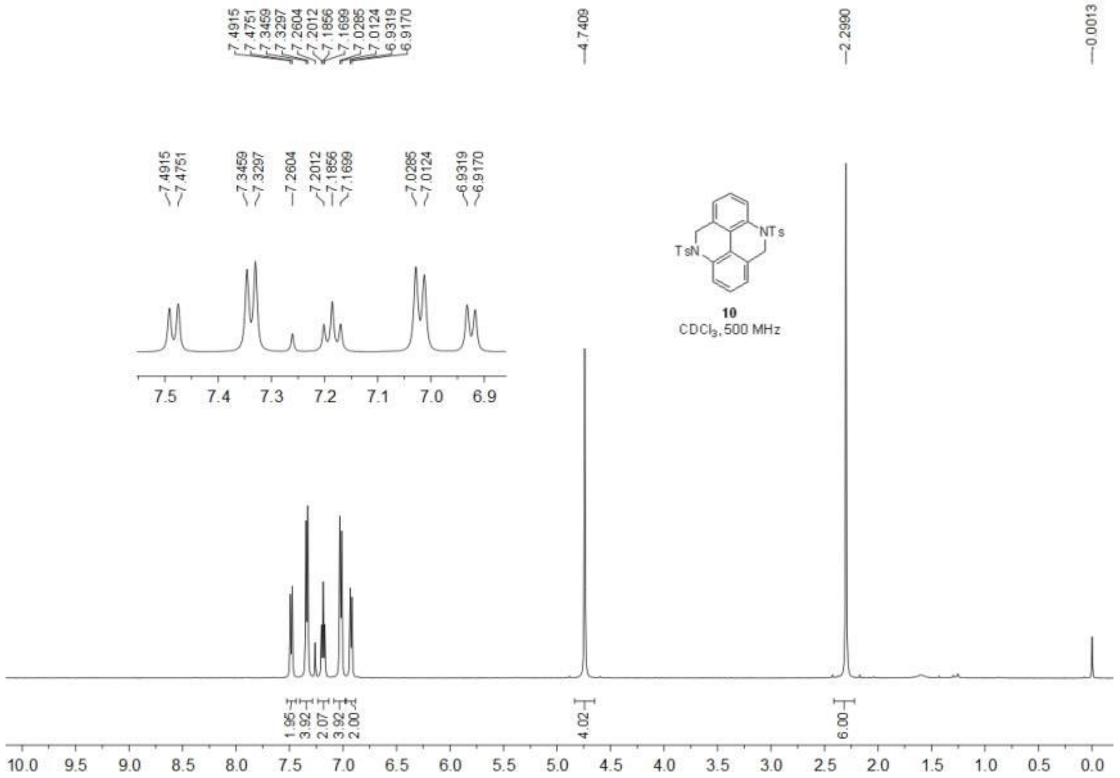

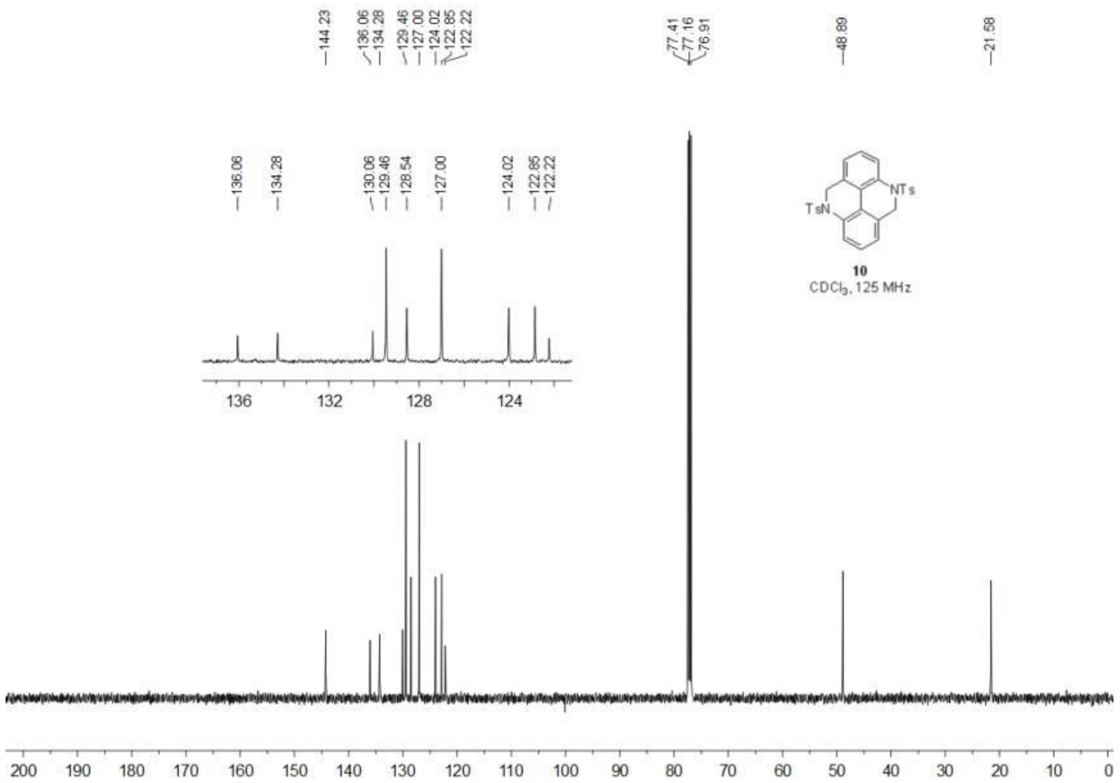

Figure S132. ^{1}H and ^{13}C NMR spectra of **10.** Related to **Figure** 7.

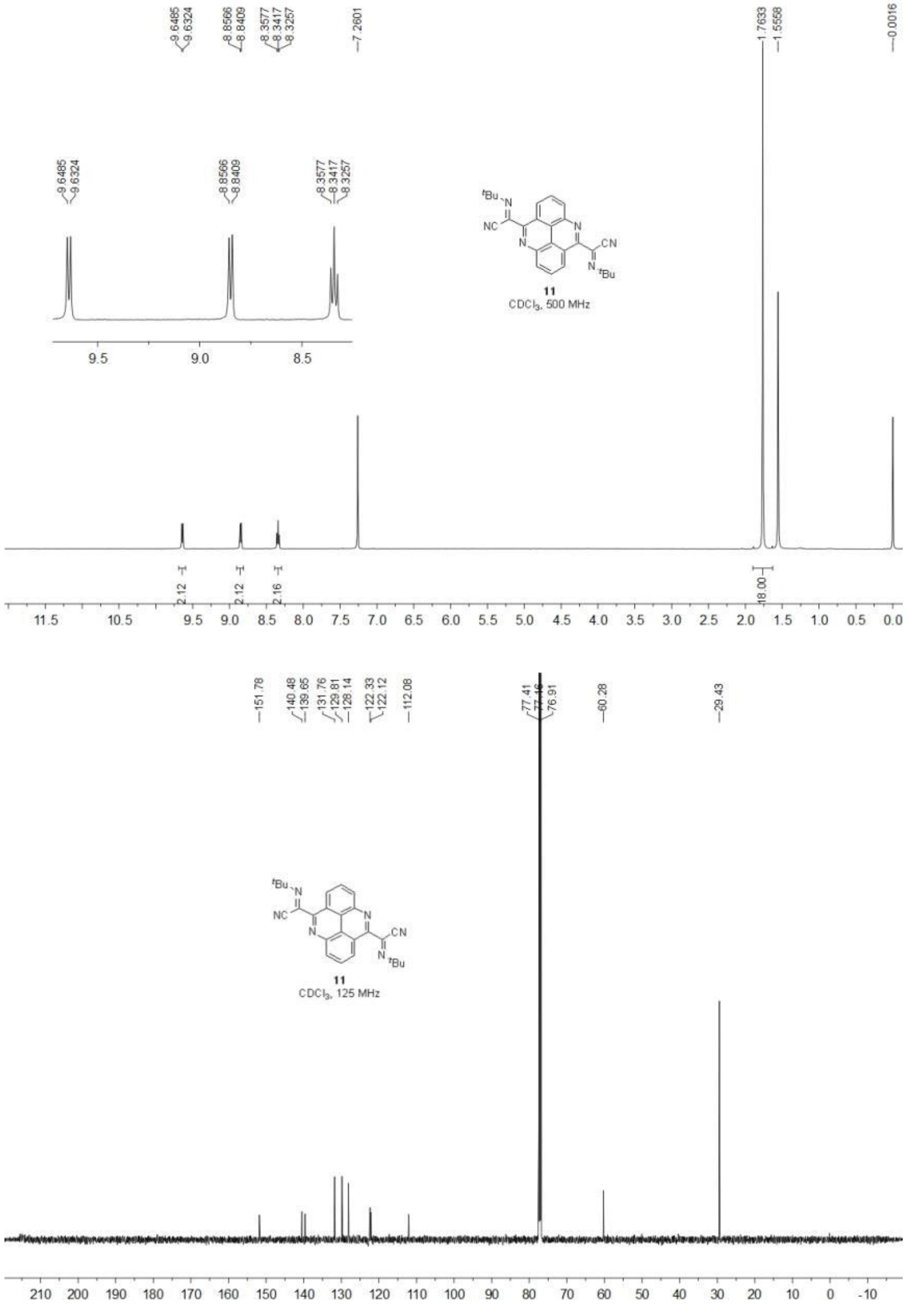

Figure S133. ^{1}H and ^{13}C NMR spectra of **11**. Related to **Figure** 7.

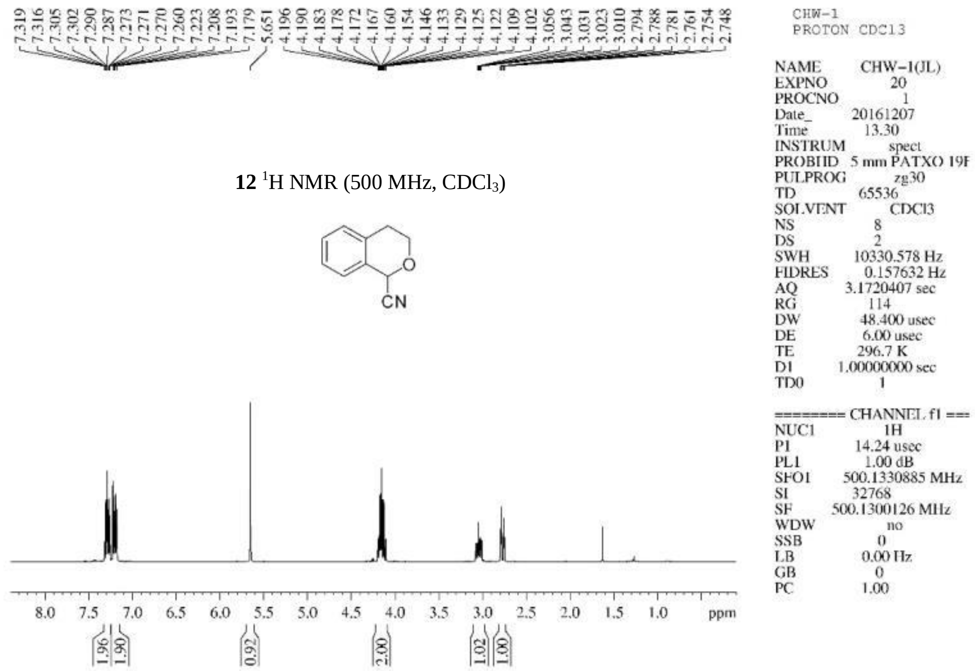

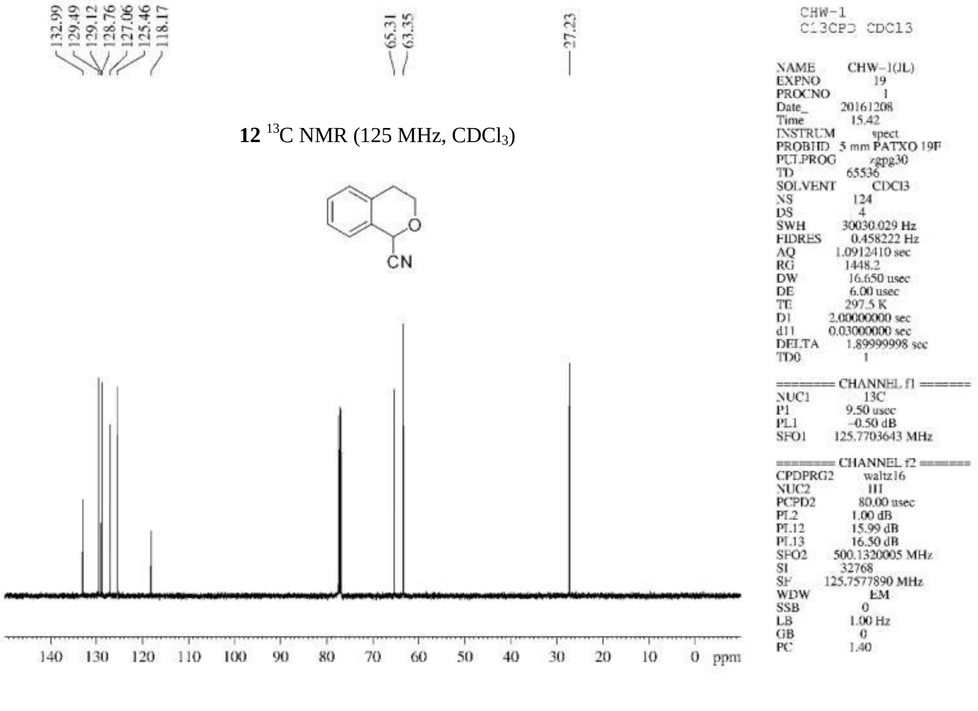

Figure S134. ¹H and ¹³C NMR spectra of **12**. Related to **Figure 8**.

Supplemental References

Cheng, X., Yang, B., Hu, X., Xu, Q., and Lu, Z. (2016). Visible–light–promoted metal–free aerobic oxidation of primary amines to acids and lactones. Chem. Eur. J. *22*, 17566-17570.

Gawlak, M., and Robbins, R. F. (1964). The peracid oxidation of 4,9-diazapyrene. J. Chem. Soc. 5135-5139.

Gonzalez-de-Castro, A., Robertson, C. M., and Xiao, J. (2014). Dehydrogenative α-oxygenation of ethers with an iron catalyst. J. Am. Chem. Soc. *136*, 8350–8360.

Michael, C., Donald, W., Marie, J., Erhu, L., and Fuye, G. Compounds and methods for treating protein folding disorders. US. 2010/0144821[P], 2010-6-10.

Muramatsu and Nakano, K. (2014). Organocatalytic approach for $C(sp^3)$–H bond arylation, alkylation, and amidation of isochromans under facile conditions. Org. Lett. *16*, 2042–2045.

Park, W. K. C., Kennedy, R. M., Larsen, S. D., Miller, S., Roth, B. D., Song, Y., Steinbaugh, B. A., Sun, K., Tait, B. D., Kowala, M. C., Trivedi, B. K., Auerbach, B., Askew, V., Dillon, L., Hanselman, J. C., Lin, Z., Lu, G. H., Robertson, A., and Sekerke, C. (2008). Hepatoselectivity of statins: design and synthesis of 4-sulfamoyl pyrroles as HMG-CoA reductase inhibitors. Bioorg. Med. Chem. Lett. *18*, 1151–1156.

Pingaew, R., Worachartcheewan, A., Nantasenamat, C., Prachayasittikul, S., Ruchirawat, S., and Prachayasittikul, V. (2013). Synthesis, cytotoxicity and QSAR study of *N*-tosyl-1,2,3,4-tetrahydroiso-petrquinoline derivatives. Arch. Pharm. Res. *36*, 1066–1077.

Sullivan, S., Doni, E., Tuttle, T., and Murphy, J. (2014). Metal–free reductive cleavage of C–N and S–N bonds by photoactivated electron transfer from a neutral organic donor. Angew. Chem. Int. Ed. *53*, 474–478.

Xu, S., Huang, X., Hong, X., and Xu, B. (2012). Palladium-assisted regioselective C–H cyanation of heteroarenes using isonitrile as cyanide source. Org. Lett. *14*, 4614–4617.

Yan, C., Liu, Y., and Wang, Q. (2014). Mild and highly efficient metal-free oxidative α-cyanation of *N*-acyl/sulfonyl tetrahydroisoquinolines. RSC Adv. *4*, 60075–60078.

Zhou, M., Kong, S., Zhang, L., Zhao, M., Duan, J., Ou-yang, Z., and Wang, M. (2013). $CuBr_2$ catalyzed bromination/oxidation of isochromans to benzaldehyde derivatives. Tetrahedron Lett. *54*, *3*962–3964.
